# Supplementary material for: The Middle Eastern Cousin: Comparative Venomics of Daboia palaestinae and Daboia russelii
Source: Toxins (Basel). 2022 Oct 23;14(11):725. doi: 10.3390/toxins14110725 (PMC9696752; doi:10.3390/toxins14110725)
Supplement: Supplementary file 1 [file toxins-14-00725-s001.zip › Supplementary figures_revised.pdf]

Supplementary Figures

Supplementary Figure S1. SDS-PAGE profile of *Daboia palaestinae* venom

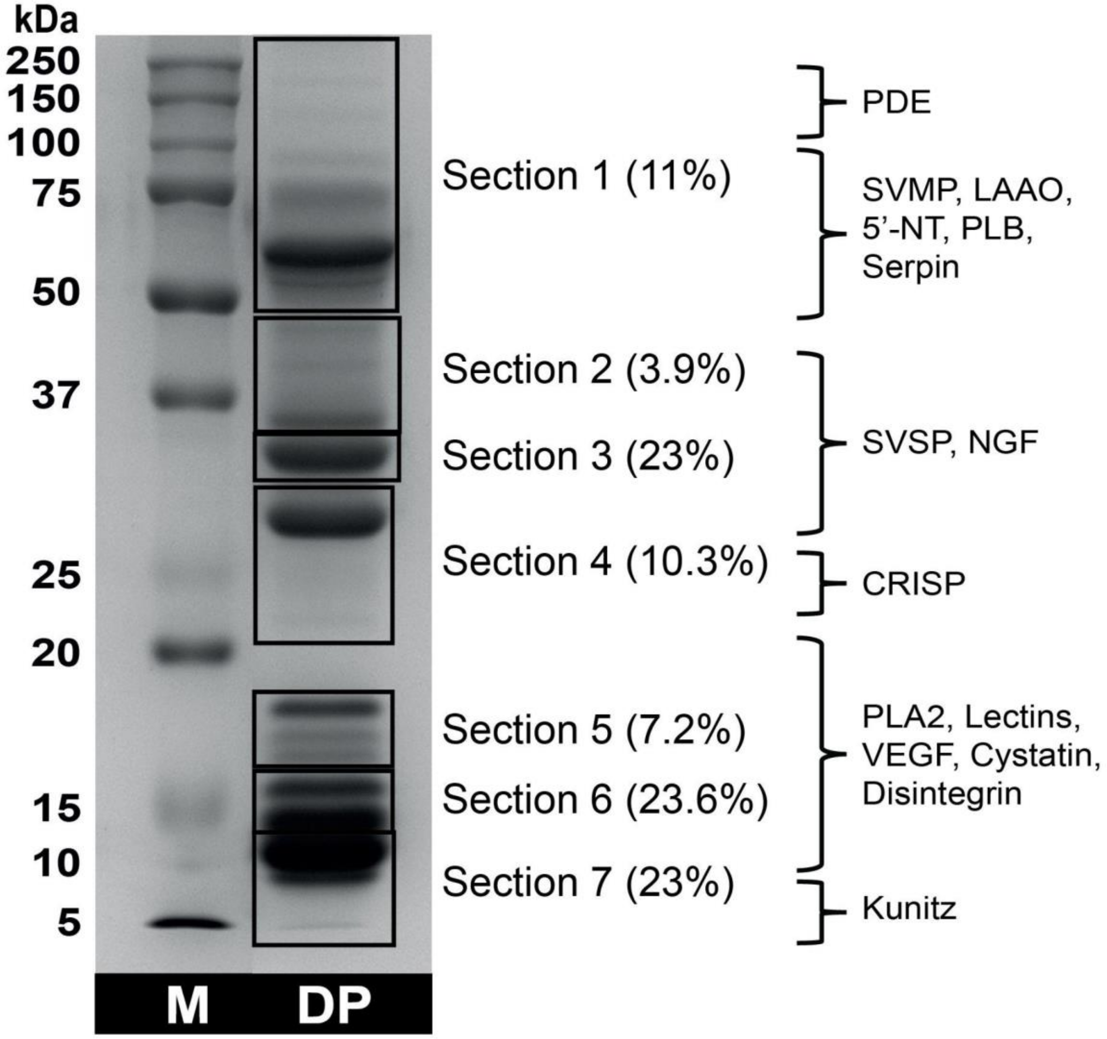

The annotated SDS-PAGE gel image *Daboia palaestinae* is shown here. This image indicates distinct bands that were excised for in-gel digestion and the toxins identified in those respective bands through mass spectrometric analysis.

Supplementary Figure S2. RP-HPLC profiles of *D. palaestinae* and *D. russelii* venoms.

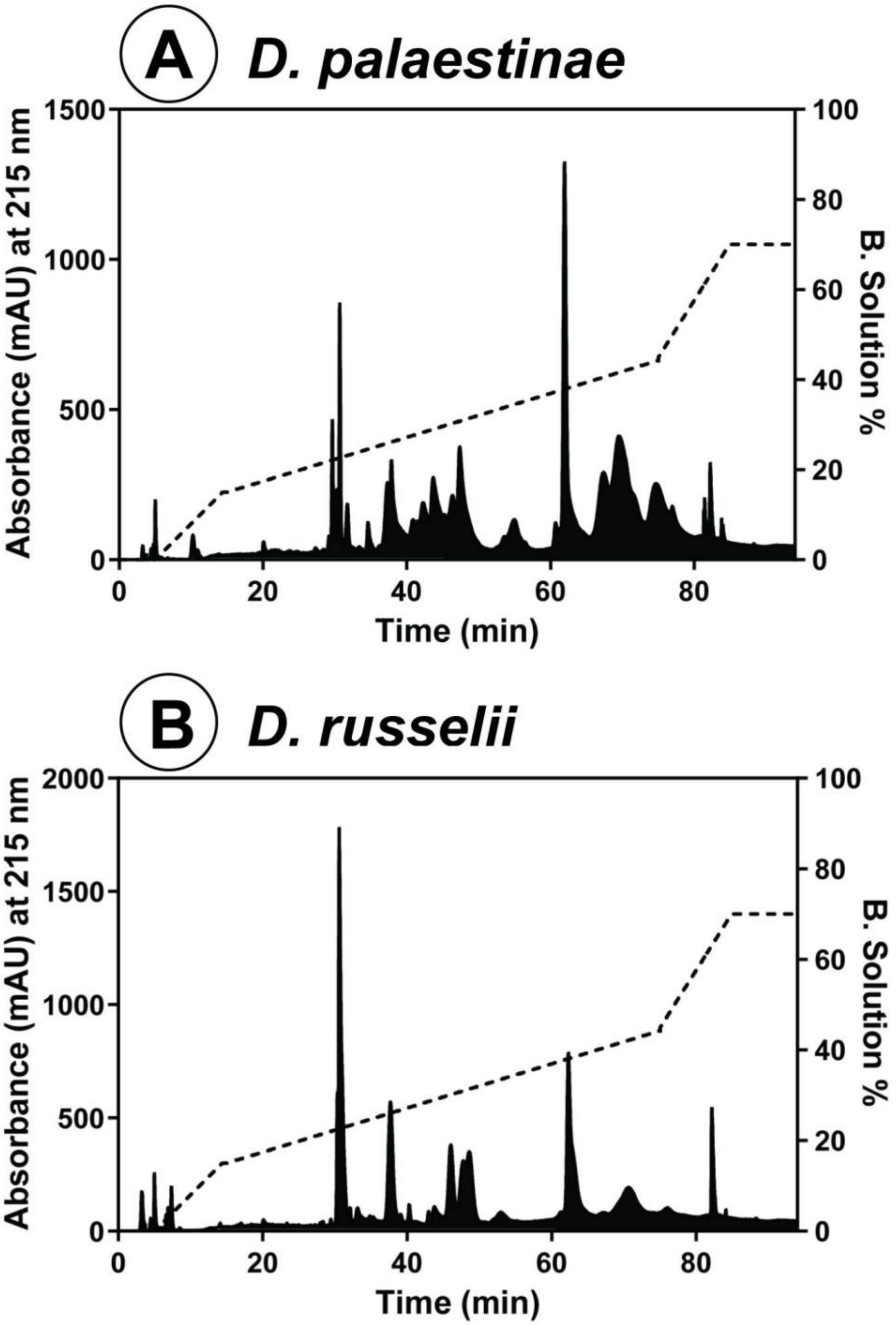

This figure illustrates the RP-HPLC profiles of (A) *D. palaestinae* and (B) *D. russelii* venoms. Here, the differences in the pattens and intensities of peaks represent the differences in venom compositions.

Supplementary Figure S3: The DNase activity of *Daboia* venoms.

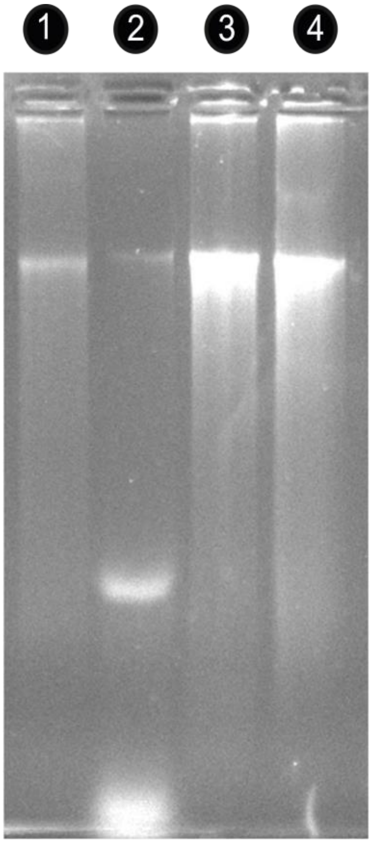

This agarose gel depicts the results of DNase assay, wherein, the DNA from calf thymus (500 ng), mixed with venoms or positive control, was subjected to horizontal electrophoresis. Lane **(1)**: purified DNA (negative control); **(2)**: DNA + bovine pancreatic DNase (positive control); **(3)**: *D. russelii*; **(4)**: *D. palaestinae*.

Supplementary Figure S4: The fibrinogenolytic activity of the *D. palaestinae* venom.

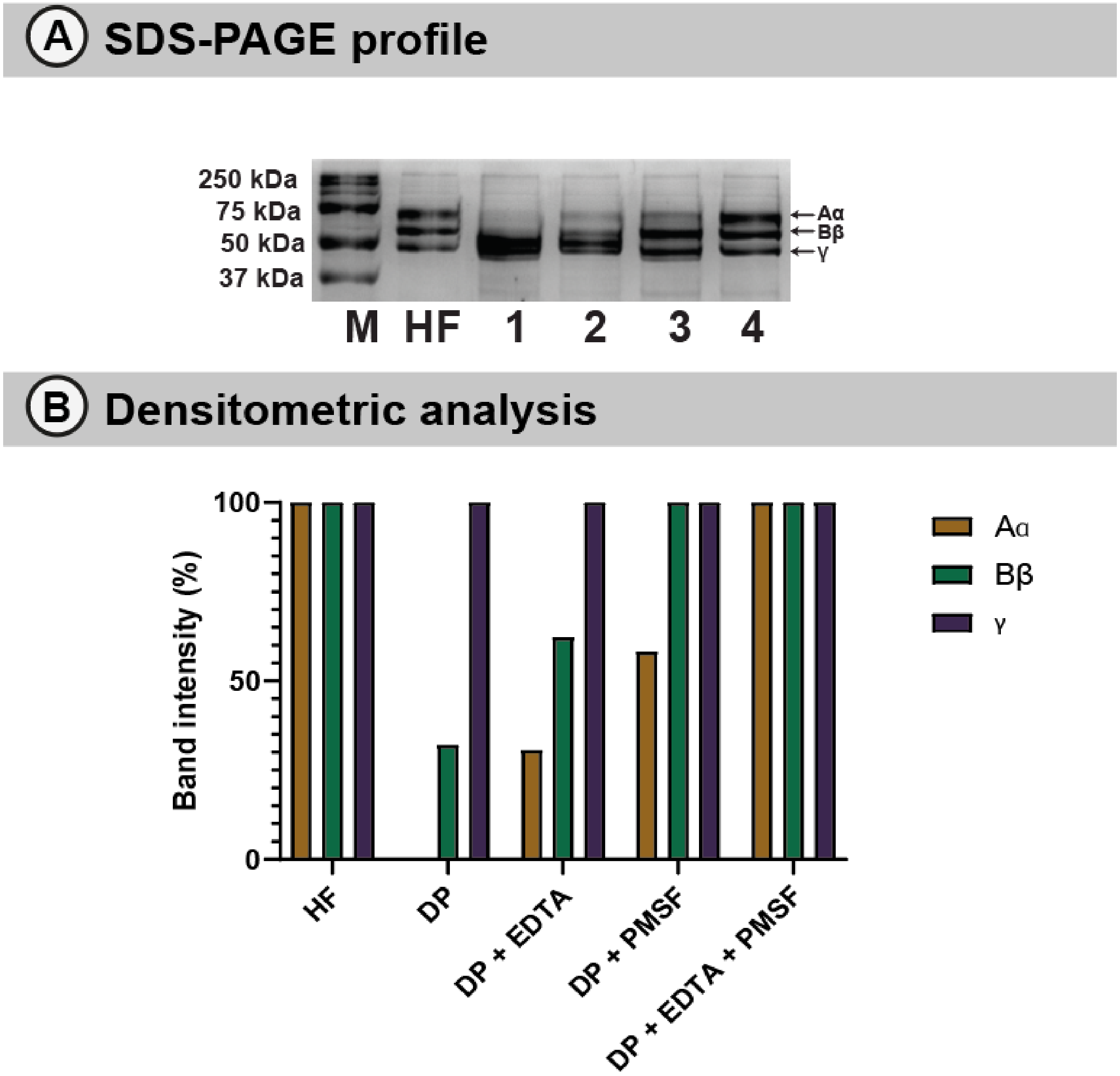

The figure highlights the fibrinogenolytic activity of *D. palaestinae* venom **(A)** The SDS-PAGE gel depicts the enzymatic cleavage of human fibrinogen Aα, Bβ and γ bands. **(M)**: Pre-stained protein ladder; **(HF)**: Human fibrinogen only (negative control); **(1)**: human fibrinogen mixed with *D. palaestinae* venom; **(2):** human fibrinogen mixed with *D. palaestinae* venom and EDTA; **(3):** human fibrinogen mixed with *D. palaestinae* venom and PMSF; **(4):** human fibrinogen mixed with *D. palaestinae* venom, EDTA and PMSF. **(B)** The intensity of SDS-PAGE bands were calculated using ImageJ. The reduction in Aα, Bβ and γ band intensities (%) due to the fibrinogenolytic activity of *D. palaestinae*

venom is indicated. The relative reduction in band intensity was calculated using human fibrinogen as control (100% intensity).

Supplementary Figure S5: The fibrinogenolytic activity of the *D. russelii* venom.

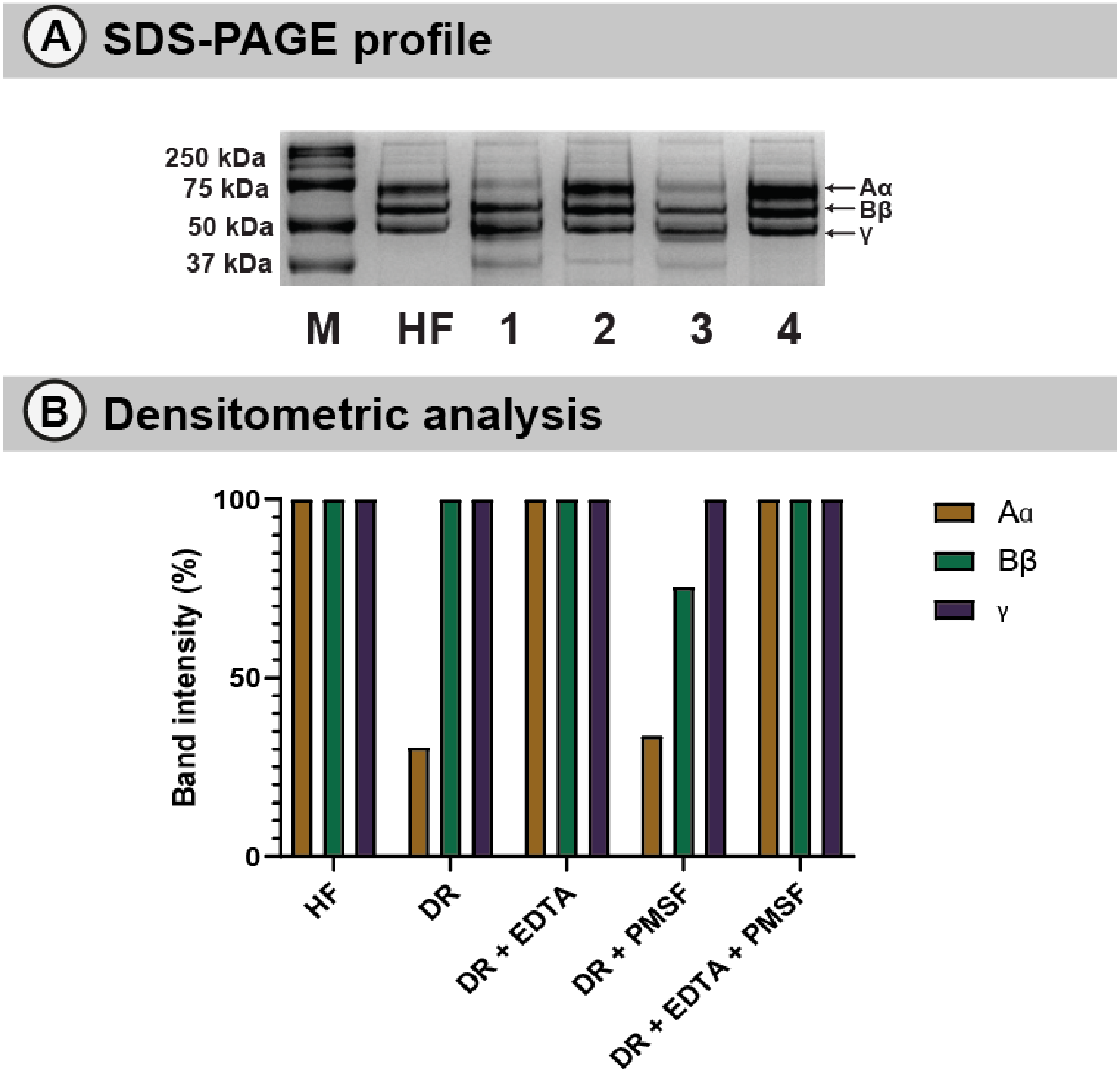

The figure highlights the fibrinogenolytic activity of *D. russelii* venom **(A)** The SDS-PAGE gel depicts the enzymatic cleavage of human fibrinogen Aα, Bβ and γ bands. **(M)**: Pre-stained protein ladder; **(HF)**: Human fibrinogen only (negative control); **(1)**: human fibrinogen mixed with *D. russelii* venom; **(2):** human fibrinogen mixed with *D. russelii* venom and EDTA; **(3):** human fibrinogen mixed with *D. russelii* venom and PMSF; **(4):** human fibrinogen mixed with *D. russelii* venom, EDTA and PMSF. **(B)** The intensity of SDS-PAGE bands were calculated using ImageJ. The reduction in Aα, Bβ and γ band intensities (%) due to the fibrinogenolytic activity of *D. russelii* venom is indicated. The

relative reduction in band intensity was calculated using human fibrinogen as control (100% intensity).

Supplementary Figure S6. The hemorrhagic potential of *Daboia* venoms.

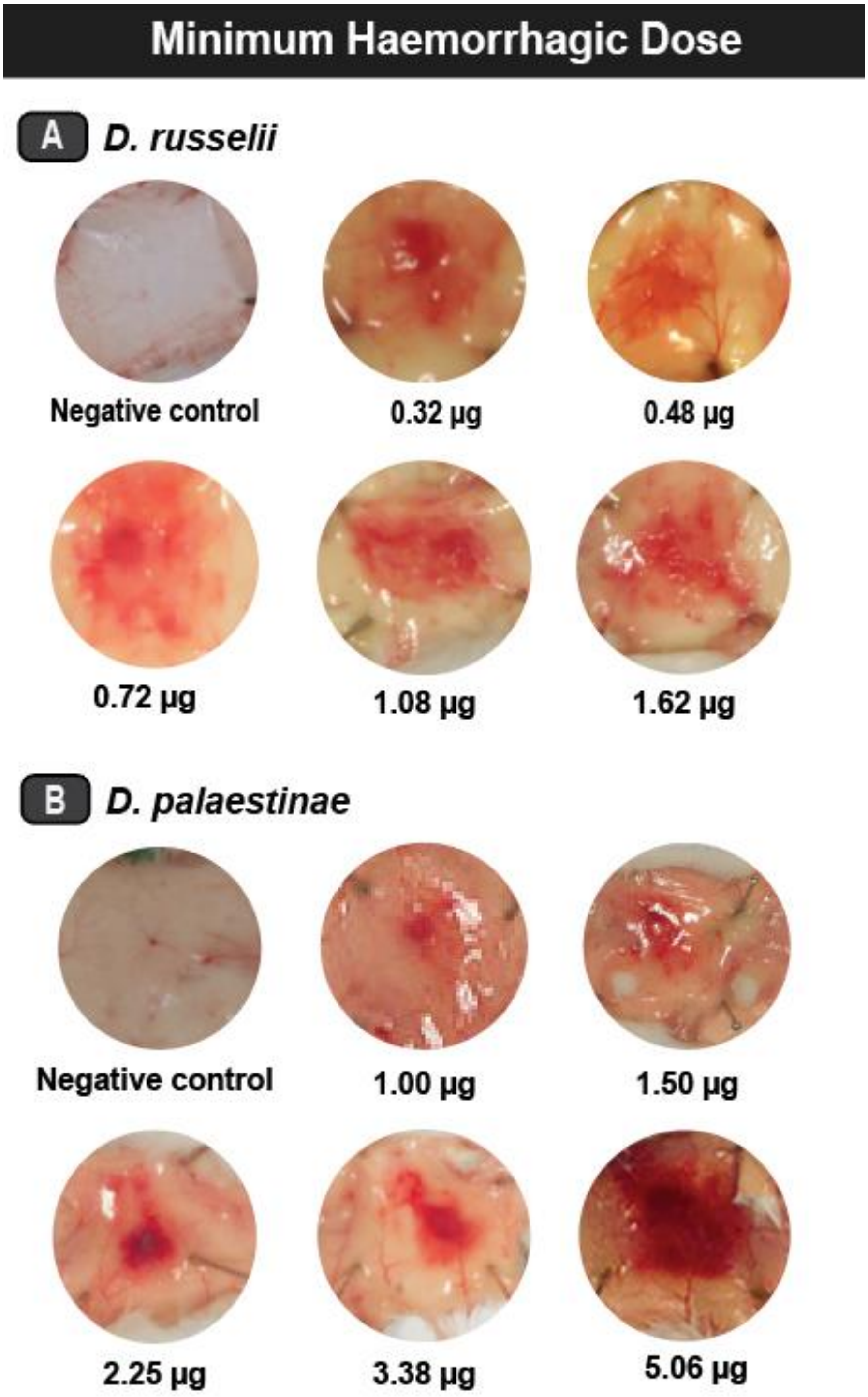

This figure illustrates the haemorrhagic potentials of **(A)** *D. russelii* and **(B)** *D. palaestinae* venoms. Here, the haemorrhagic activities are directly proportional to the increasing concentrations of venom.

Supplementary Figure S7. The necrotic potential of *D. russelii* venom.

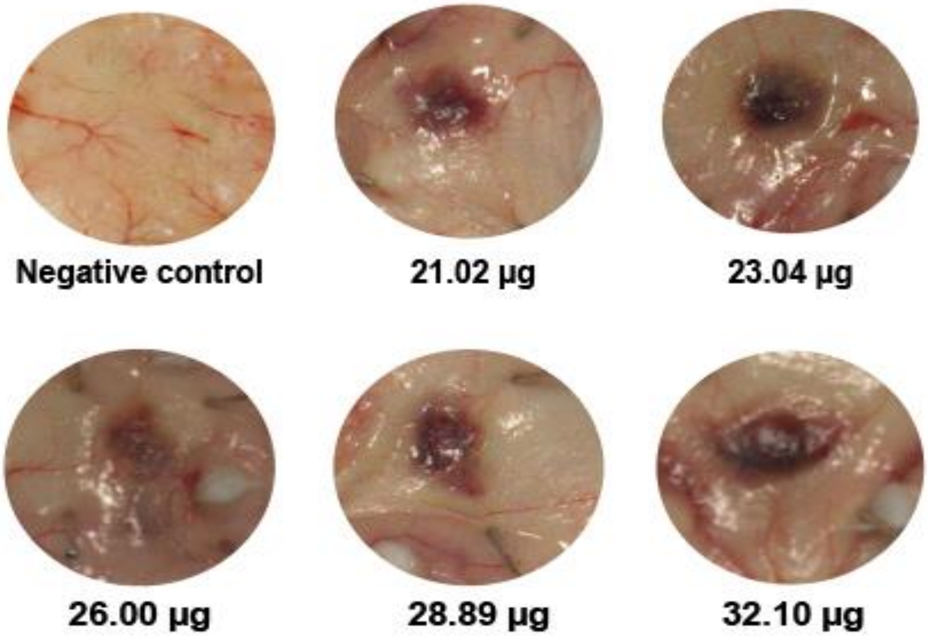

This figure illustrates the necrotizing effects of the *D. russelii* venom. Here, the necrotic lesions caused by various concentrations of the venom are shown. In these experiments, the MND was found to lie between 20 to 25 µg. In the range finding experiments with the *D. palaestinae* venom, necrotic symptoms were not observed.
