# Supplementary material for: The Middle Eastern Cousin: Comparative Venomics of Daboia palaestinae and Daboia russelii
Source: Toxins (Basel). 2022 Oct 23;14(11):725. doi: 10.3390/toxins14110725 (PMC9696752; doi:10.3390/toxins14110725)
Supplement: Supplementary file 1 [file toxins-14-00725-s001.zip › Supplementary File S1.pdf]

Summary

1. Notes

2. Result Statistics

Figure S8. False discovery rate (FDR) curve. X axis is the number of peptide-spectrum matches (PSM) being kept. Y axis is the corre sponding FDR.

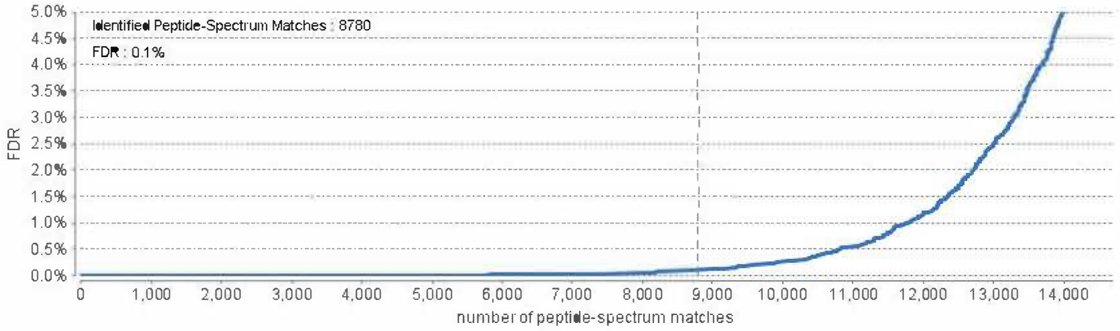

Figure S9. PSM score distribution. **(a)** Distribution of PEAKS peptide score; **(b)** Scatterplot of PEAKS peptide score versus precursor mass error.

(a) **(b)**

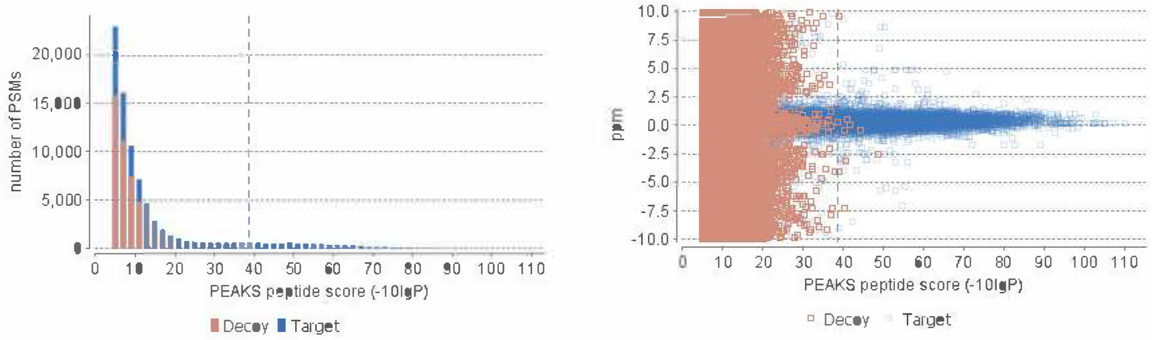

Table S5. Statistics of data.

	#Scans		#Features	Identified			#Peptides	#Sequences	#Proteins*		
	MS1	MS2		#PSMs	#Scans	#Features			Groups	All	Top
Total	61192	194255	216084	8780	8613	17484	1139	1016	164	1225	501
F1	7283	29947	27286	1302	1268	2226	443	403	82	583	228
F2	8579	27501	25645	1296	1262	1693	414	362	84	586	213
F3	7215	29271	40734	1484	1472	2985	422	374	84	578	237
F4	7830	28612	34895	1302	1292	2937	485	432	101	693	343
F5	10472	25367	27892	1009	976	2239	278	251	82	519	263
F6	10484	26040	26892	832	814	2188	293	269	74	467	280
F7	9329	27517	32740	1555	1529	3216	344	313	91	466	246

***** proteins with significant peptides are used in counts.

Figure S10. Sample overlap for Proteins and Peptides (up to 8 samples). **(a)** All Proteins; **(b)** Top Proteins; **(c)** Peptides;

(a)

	1	2	3	4	5	6	7	Unique
1	583	421	408	420	290	264	162	94
2	421	586	435	446	318	269	170	69
3	408	435	578	461	345	294	227	39
4	420	446	461	693	404	414	263	38
5	290	318	345	404	519	376	279	16
6	264	269	294	414	376	467	247	19
7	162	170	227	263	279	247	466	138

(b)

	1	2	3	4	5	6	7	Unique
1	228	168	152	192	128	148	93	27
2	168	213	165	190	128	132	90	13
3	152	165	237	220	168	169	133	8
4	192	190	220	343	226	253	159	14
5	128	128	168	226	263	228	168	4
6	148	132	169	253	228	280	159	4
7	93	90	133	159	168	159	246	54

(c)

	1	2	3	4	5	6	7	Unique
1	443	255	220	239	158	143	122	122
2	255	414	259	254	154	130	115	71
3	220	259	422	274	162	141	115	82
4	239	254	274	485	205	199	151	85
5	158	154	162	205	278	177	146	27
6	143	130	141	199	177	293	139	50
7	122	115	115	151	146	139	344	154

Figure S11. Distribution of peptide feature detection. **(a)** Feature m/z distribution; **(b)** Feature RT distribution.

(a) **(b)**

Protein List

Protein Accession Contains:

Protein Description Contains:

Protein Sample Area >=

Protein PTM Contains:

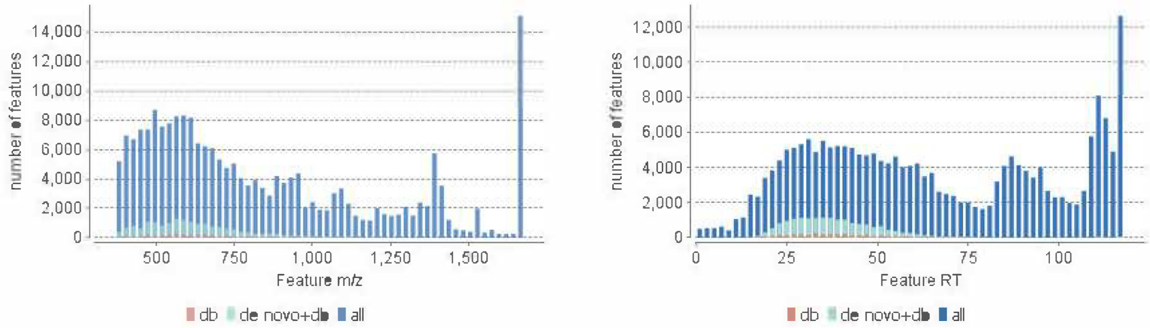

Figure S12. Distribution of identified peptide features. **(a)** Feature abundance distribution; **(b)** *De novo* sequencing validation.

(a) **(b)**

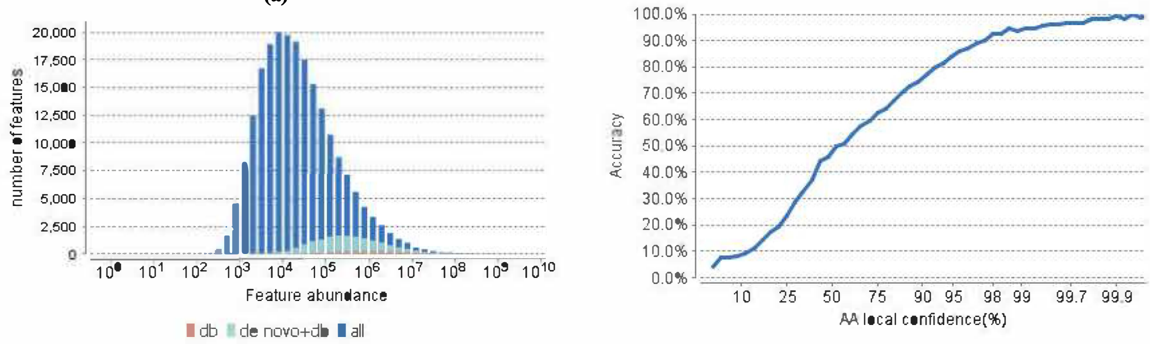

Table S6. Result filtration parameters.

Peptide -10lgP	≥38.7
PTM Ascore	≥0
Protein -10lgP	≥20
Proteins unique peptides	≥1
De novo score(%)	≥50%

Table S8. PTM profile.

Name	ΔMass	Position	#PSM	-10lgP	Abundance	AScore
Oxidation	15.99	M	1758	89.44	5.86E6	1000.00
Carbamidomethyl	57.02	C	28	60.69	1.42E6	1000.00

Table S7. Statistics of filtered result.

FDR (Peptide-Spectrum Matches)	0.1%
FDR (Peptide Sequences)	0.8%
FDR (Protein Group)	6.0%
De Novo Only Spectra	25886

3. Experiment Control

Figure S13. Precursor mass error of peptide-spectrum matches (PSM) in filtered result. **(a)** Distribution of precursor mass error in ppm; **(b)** Scatterplot of precursor m/z versus precursor mass error in ppm.

(a) **(b)**

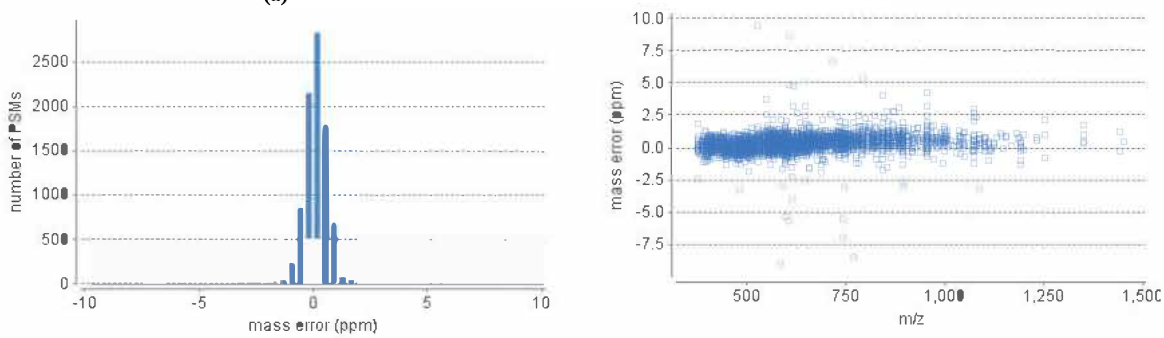

Table S9. Number of identified peptides in each sample by the number of missed cleavages.

Missed Cleavages	0	1	2	3	4+
F1	313	113	15	2	0
F2	325	78	11	0	0
F3	308	96	18	0	0
F4	381	94	9	1	0
F5	235	39	4	0	0
F6	239	49	5	0	0
F7	276	60	8	0	0

4. Other Information

Table S10. Search parameters.

Search Engine Name: PEAKS
Parent Mass Error Tolerance: 10.0 ppm
Fragment Mass Error Tolerance: 0.6 Da
Precursor Mass Search Type: monoisotopic
Enzyme: Trypsin
Max Missed Cleavages: 3
Digest Mode: Semispecific
Fixed Modifications:
  Carbamidomethylation: 57.02
Variable Modifications:
  Oxidation (M): 15.99
Max Variable PTM Per Peptide: 3
Database: Dpal_Serpent_NR
Taxon: All
Contaminant Database: cRAP_contaminants
Searched Entry: 572962
FDR Estimation: Enabled
Merge Options: no merge
Precursor Options: corrected
Charge Options: no correction
Filter m/z: 200.0 - 1800.0
Filter Charge: 1 - 8
Process: true
Associate chimera: yes

Table S11. Instrument parameters.

Fractions: 12112021_RID_2349_DP1.raw, 12112021_RID_2349_DP2.raw, 17112021_RID_2349_DP3.raw, 17112021_RID_2349_DP4.raw, 17112021_RID_2349_DP5.raw, 17112021_RID_2349_DP6.raw, 17112021_RID_2349_DP7.raw
Ion Source: ESI(nano-spray)
Fragmentation Mode: CID, CAD(y and b ions)
MS Scan Mode: FT-ICR/Orbitrap
MS/MS Scan Mode: FT-ICR/Orbitrap

Protein Group	Protein ID	Accession	-10lgP	Coverage (%)	Coverage (%) F1	Coverage (%) F2	Coverage (%) F3	Coverage (%) F4	Coverage (%) F5	Coverage (%) F6	Coverage (%) F7	Area F1	Area F2	Area F3	A
1	69771	TRINITY_DN9	397.42	65	42	40	48	38	21	15	7	6.0902E8	1.4832E8	2.8298E8	1.4
21	69773	TRINITY_DN233	359.11	51	42	45	24	26	5	4	3	1.5641E8	4.5667E7	1.4274E7	4.2
27	7	AHJ80886.1	337.24	49	38	34	36	40	16	3	9	6.4491E7	1.3431E7	2.4346E7	1.3
12	5	G8XQX1.1	332.80	56	28	25	27	23	13	9	17				
12	6	ACF70483.1	332.80	56	28	25	27	23	13	9	17				
18	69775	TRINITY_DN0	307.80	38	16	31	27	23	11	6	4	9.7514E5	9.7519E7	3.3637E7	1.3
50	69777	TRINITY_DN132	293.50	30	24	11	13	7	0	0	0	8.2659E7	8.1798E6	8.9798E6	3.8
17	69809	TRINITY_DN441	270.06	71	29	29	29	71	37	38	0	1.7543E6	2.3578E6	5.09E8	1.3
101	69780	TRINITY_DN1507	257.03	24	24	5	1	1	0	0	0	2.7944E7	1.0493E5	4.3717E4	1.4
101	69780	TRINITY_DN1507	257.03	24	24	5	1	1	0	0	0	2.7944E7	1.0493E5	4.3717E4	1.4
101	69782	TRINITY_DN1507	257.03	22	22	5	1	1	0	0	0	2.7944E7	1.0493E5	4.3717E4	1.4
101	69783	TRINITY_DN1507	257.03	21	21	4	1	1	0	0	0	2.7944E7	1.0493E5	4.3717E4	1.4
101	69784	TRINITY_DN1507	257.03	22	22	5	1	1	0	0	0	2.7944E7	1.0493E5	4.3717E4	1.4
101	69785	TRINITY_DN1507	257.03	20	20	4	1	1	0	0	0	2.7944E7	1.0493E5	4.3717E4	1.4
61	69796	TRINITY_DN30	254.87	13	10	10	13	10	6	1	0	2.1784E5	1.6365E6	7.4177E6	3.3
61	239	TRINITY_DN30	254.87	13	10	10	13	10	6	1	0	2.1784E5	1.6365E6	7.4177E6	3.3
39	70	BAN82155.1	251.91	22	6	17	12	11	5	2	0		3.5059E4		

total 523 p

Protein Group	Protein ID	Accession	-10lgP	Coverage (%)	Coverage (%) F1	Coverage (%) F2	Coverage (%) F3	Coverage (%) F4	Coverage (%) F5	Coverage (%) F6	Coverage (%) F7	Area F1	Area F2	Area F3	A
**79**	69799	TRINITY_DN266	248.99	36	22	25	16	30	18	0	4	4.5074E6	4.1083E6	2.5052E6	4.8
**5**	69839	TRINITY_DN240	245.05	44	13	13	44	28	20	8	6	2.2296E6	1.724E7	8.9727E9	2.7
**68**	69803	TRINITY_DN59	243.26	37	6	0	0	0	16	37	9	2.9622E6			
**68**	69804	TRINITY_DN59	243.26	37	6	0	0	0	16	37	9	2.9622E6			
**68**	69803	TRINITY_DN59	243.26	37	6	0	0	0	16	37	9	2.9622E6			
**43**	69825	TRINITY_DN1385	231.26	40	37	32	33	31	31	0	0	2.7681E7	2.4791E7	1.6852E8	2.7
**90**	304	QHR82712.1	229.92	32	17	25	16	24	13	0	0		6.057E5	4.6515E6	
**86**	129	AFE84762.1	229.84	35	0	35	9	10	3	0	0		3.0729E6		
**14**	69867	TRINITY_DN554	226.40	16	0	0	0	0	16	11	11				
**144**	69793	TRINITY_DN1031	219.72	19	0	6	12	6	0	0	0		1.7546E6	7.494E7	1.6
**144**	69793	TRINITY_DN1031	219.72	19	0	6	12	6	0	0	0		1.7546E6	7.494E7	1.6
**144**	69793	TRINITY_DN1031	219.72	19	0	6	12	6	0	0	0		1.7546E6	7.494E7	1.6
**32**	382	Q9PT40.1	215.17	26	8	17	23	18	11	3	0	3.788E5	1.6386E8	5.3429E8	5.4
**32**	383	CAB62591.1	215.17	26	8	17	23	18	11	3	0	3.788E5	1.6386E8	5.3429E8	5.4
**46**	505	P0DL42.1	206.88	39	0	0	0	7	0	0	39				
**75**	285	Q4VM07.1	205.78	12	9	6	9	4	2	4	1	2.3772E6	4.2446E5	8.333E6	2.5
**75**	286	AAX38182.1	205.78	12	9	6	9	4	2	4	1	2.3772E6	4.2446E5	8.333E6	2.5
**25**	70177	TRINITY_DN459	199.41	11	11	11	11	11	11	11	11	1.2856E8	3.7626E6	6.1058E7	4.1
**25**	70311	TRINITY_DN1910	199.41	23	23	23	23	23	23	23	23	1.2856E8	3.7626E6	6.1058E7	4.1
**180**	69790	TRINITY_DN509	198.91	18	0	6	0	12	3	0	0		1.3078E5		5.3
**180**	69791	TRINITY_DN509	198.91	17	0	6	0	11	2	0	0		1.3078E5		5.3
**41**	465	ADK22823.1	197.97	31	0	0	0	0	5	14	21				
**41**	466	ABA86561.1	197.97	31	0	0	0	0	5	14	21				
**41**	467	Q38L02.1	197.97	31	0	0	0	0	5	14	21				

total 523 p

Protein Group	Protein ID	Accession	-10lgP	Coverage (%)	Coverage (%) F1	Coverage (%) F2	Coverage (%) F3	Coverage (%) F4	Coverage (%) F5	Coverage (%) F6	Coverage (%) F7	Area F1	Area F2	Area F3	A
**41**	476	ADK22821.1	197.97	31	0	0	0	0	5	14	21				
**22**	442	AAZ53176.1	195.25	40	9	9	9	9	9	9	40	5.8348E5	5.2464E4	1.2575E6	2.1
**22**	443	A8CG78.1	195.25	40	9	9	9	9	9	9	40	5.8348E5	5.2464E4	1.2575E6	2.1
**22**	444	AAZ53181.1	195.25	40	9	9	9	9	9	9	40	5.8348E5	5.2464E4	1.2575E6	2.1
**22**	445	A8CG87.1	195.25	40	9	9	9	9	9	9	40	5.8348E5	5.2464E4	1.2575E6	2.1
**91**	69842	TRINITY_DN64	193.05	37	8	8	19	19	37	8	8			5.8246E5	1.5
**91**	525	B4XSY9.1	193.05	30	6	6	15	15	30	6	6			5.8246E5	1.5
**91**	526	ABW82661.1	193.05	30	6	6	15	15	30	6	6			5.8246E5	1.5
**91**	527	B4XSZ0.1	193.05	30	6	6	15	15	30	6	6			5.8246E5	1.5
**91**	528	ABW82662.1	193.05	30	6	6	15	15	30	6	6			5.8246E5	1.5
**195**	223	TRINITY_DN91	192.10	8	8	0	0	0	0	0	0	5.1733E5			
**195**	223	TRINITY_DN91	192.10	8	8	0	0	0	0	0	0	5.1733E5			
**195**	223	TRINITY_DN91	192.10	8	8	0	0	0	0	0	0	5.1733E5			
**195**	223	TRINITY_DN91	192.10	8	8	0	0	0	0	0	0	5.1733E5			
**195**	223	TRINITY_DN91	192.10	8	8	0	0	0	0	0	0	5.1733E5			
**146**	165	ETE57948.1	190.95	30	6	30	6	13	3	9	0	3.5744E5	4.2136E6	1.2599E5	5.4
**146**	166	JAC96363.1	190.95	29	6	29	6	13	3	9	0	3.5744E5	4.2136E6	1.2599E5	5.4
**146**	167	JAA97900.1	190.95	29	6	29	6	13	3	9	0	3.5744E5	4.2136E6	1.2599E5	5.4
**146**	168	LAA21099.1	190.95	29	6	29	6	13	3	9	0	3.5744E5	4.2136E6	1.2599E5	5.4
**146**	169	JAI10342.1	190.95	29	6	29	6	13	3	9	0	3.5744E5	4.2136E6	1.2599E5	5.4
**146**	170	JAG68843.1	190.95	29	6	29	6	13	3	9	0	3.5744E5	4.2136E6	1.2599E5	5.4
**146**	171	XP_013921350.1	190.95	29	6	29	6	13	3	9	0	3.5744E5	4.2136E6	1.2599E5	5.4
**146**	172	JAB54771.1	190.95	29	6	29	6	13	3	9	0	3.5744E5	4.2136E6	1.2599E5	5.4
**146**	173	JAA97901.1	190.95	29	6	29	6	13	3	9	0	3.5744E5	4.2136E6	1.2599E5	5.4
**146**	174	JAG68844.1	190.95	29	6	29	6	13	3	9	0	3.5744E5	4.2136E6	1.2599E5	5.4
**146**	175	LAA21098.1	190.95	29	6	29	6	13	3	9	0	3.5744E5	4.2136E6	1.2599E5	5.4
**146**	176	JAV51374.1	190.95	29	6	29	6	13	3	9	0	3.5744E5	4.2136E6	1.2599E5	5.4
**146**	177	JAG47580.1	190.95	29	6	29	6	13	3	9	0	3.5744E5	4.2136E6	1.2599E5	5.4
**146**	178	AFJ49303.1	190.95	29	6	29	6	13	3	9	0	3.5744E5	4.2136E6	1.2599E5	5.4
**146**	179	JAI14496.1	190.95	29	6	29	6	13	3	9	0	3.5744E5	4.2136E6	1.2599E5	5.4
**146**	180	JAI10341.1	190.95	29	6	29	6	13	3	9	0	3.5744E5	4.2136E6	1.2599E5	5.4
**146**	181	JAI14495.1	190.95	29	6	29	6	13	3	9	0	3.5744E5	4.2136E6	1.2599E5	5.4
**146**	182	JAC96364.1	190.95	29	6	29	6	13	3	9	0	3.5744E5	4.2136E6	1.2599E5	5.4

total 523 p

Protein Group	Protein ID	Accession	-10lgP	Coverage (%)	Coverage (%) F1	Coverage (%) F2	Coverage (%) F3	Coverage (%) F4	Coverage (%) F5	Coverage (%) F6	Coverage (%) F7	Area F1	Area F2	Area F3	A
**146**	183	JAG47579.1	190.95	29	6	29	6	13	3	9	0	3.5744E5	4.2136E6	1.2599E5	5.4
**146**	184	JAV51372.1	190.95	29	6	29	6	13	3	9	0	3.5744E5	4.2136E6	1.2599E5	5.4
**146**	185	AFJ49302.1	190.95	29	6	29	6	13	3	9	0	3.5744E5	4.2136E6	1.2599E5	5.4
**146**	186	BAQ22177.1	190.95	29	6	29	6	13	3	9	0	3.5744E5	4.2136E6	1.2599E5	5.4
**146**	187	JAG45996.1	190.95	29	6	29	6	13	3	9	0	3.5744E5	4.2136E6	1.2599E5	5.4
**146**	188	JAG45999.1	190.95	29	6	29	6	13	3	9	0	3.5744E5	4.2136E6	1.2599E5	5.4
**146**	189	JAB54395.1	190.95	29	6	29	6	13	3	9	0	3.5744E5	4.2136E6	1.2599E5	5.4
**146**	190	ETE74025.1	190.95	29	6	29	6	13	3	9	0	3.5744E5	4.2136E6	1.2599E5	5.4
**78**	529	ADK22827.1	188.30	28	6	6	6	13	28	13	13				1.4
**78**	530	ADK22825.1	188.30	28	6	6	6	13	28	13	13				1.4
**60**	540	ADN04916.1	186.74	21	4	21	13	12	3	0	0	5.4582E5	1.8664E8	4.7221E7	2.0
**60**	541	E0Y418.1	186.74	21	4	21	13	12	3	0	0	5.4582E5	1.8664E8	4.7221E7	2.0
**11**	394	ADK22824.1	186.62	35	0	0	0	0	5	0	35				
**11**	395	AAY63876.1	186.62	35	0	0	0	0	5	0	35				
**11**	396	ADK22822.1	186.62	35	0	0	0	0	5	0	35				
**11**	397	Q4PRC6.1	186.62	35	0	0	0	0	5	0	35				
**94**	70178	TRINITY_DN459	183.64	13	13	13	13	13	13	10	6	1.125E6	1.1654E6	6.0058E5	1.5
**155**	69806	TRINITY_DN157	183.50	36	4	4	4	4	4	4	36				
**107**	430	P18964.1	181.11	18	0	0	18	18	0	0	18			2.9668E7	1.3
**107**	439	ADP88558.1	181.11	17	0	0	17	17	0	0	17			2.9668E7	1.3
**107**	441	P18965.2	181.11	17	0	0	17	17	0	0	17			2.9668E7	1.3
**178**	295	TRINITY_DN2646	180.73	28	0	19	28	0	0	0	0		3.8106E5	7.4529E6	
**178**	295	TRINITY_DN2646	180.73	28	0	19	28	0	0	0	0		3.8106E5	7.4529E6	
**196**	69797	TRINITY_DN300	179.20	13	13	0	0	0	0	0	0	1.7343E6			
**196**	69797	TRINITY_DN300	179.20	13	13	0	0	0	0	0	0	1.7343E6			
**147**	308	B8K1W0.1	178.02	10	10	3	4	2	0	0	1	1.2744E7	1.1059E5		2.1
**147**	309	AAZ39880.1	178.02	10	10	3	4	2	0	0	1	1.2744E7	1.1059E5		2.1
**129**	410	ACE73567.1	175.01	15	4	0	0	14	10	0	15				8.1

total 523 p

Protein Group	Protein ID	Accession	-10lgP	Coverage (%)	Coverage (%) F1	Coverage (%) F2	Coverage (%) F3	Coverage (%) F4	Coverage (%) F5	Coverage (%) F6	Coverage (%) F7	Area F1	Area F2	Area F3	A
**189**	69815	TRINITY_DN324	174.06	15	0	13	6	0	0	0	0		1.5152E6	1.8195E6	
**13**	69822	TRINITY_DN441	172.25	35	11	18	26	22	16	22	0		1.2908E7	7.0313E9	2.2
**113**	348	AUF41660.1	170.03	10	6	3	7	5	4	3	4				
**28**	512	CAR40186.1	169.60	28	9	9	9	9	9	8	28	8.1854E6	6.5995E6	3.4681E7	2.1
**28**	513	B5U6Z2.1	169.60	28	9	9	9	9	9	8	28	8.1854E6	6.5995E6	3.4681E7	2.1
**125**	69855	TRINITY_DN59	169.25	23	0	0	0	7	6	23	14				9.8
**157**	69824	TRINITY_DN278	169.22	33	0	0	0	11	0	0	33				1.4
**157**	403	AJO70723.1	169.22	27	0	0	0	9	0	0	27				1.4
**64**	440	XP_034272439.1	168.51	5	3	3	2	4	4	4	5	5.1514E5	3.8767E5	3.8979E5	2.4
**93**	769	QBF53416.1	167.52	9	4	4	5	9	5	5	5				4.3
**44**	69826	TRINITY_DN59	166.98	37	0	0	0	0	6	34	15				
**44**	69826	TRINITY_DN59	166.98	37	0	0	0	0	6	34	15				
**179**	236	TRINITY_DN283	165.03	10	1	10	0	0	0	0	0	4.1868E4	2.2513E6		
**179**	236	TRINITY_DN283	165.03	10	1	10	0	0	0	0	0	4.1868E4	2.2513E6		
**179**	236	TRINITY_DN283	165.03	10	1	10	0	0	0	0	0	4.1868E4	2.2513E6		
**179**	236	TRINITY_DN283	165.03	10	1	10	0	0	0	0	0	4.1868E4	2.2513E6		
**179**	236	TRINITY_DN283	165.03	10	1	10	0	0	0	0	0	4.1868E4	2.2513E6		
**179**	236	TRINITY_DN283	165.03	10	1	10	0	0	0	0	0	4.1868E4	2.2513E6		
**134**	69816	TRINITY_DN714	161.86	15	4	4	4	5	8	7	0				2.3
**183**	69817	TRINITY_DN249	161.22	28	0	0	28	0	0	0	0			4.6706E6	
**183**	69817	TRINITY_DN249	161.22	28	0	0	28	0	0	0	0			4.6706E6	
**183**	69817	TRINITY_DN249	161.22	28	0	0	28	0	0	0	0			4.6706E6	
**173**	69821	TRINITY_DN77	157.32	24	0	0	0	24	0	0	2				1.6

total 523 p

Protein Group	Protein ID	Accession	-10lgP	Coverage (%)	Coverage (%) F1	Coverage (%) F2	Coverage (%) F3	Coverage (%) F4	Coverage (%) F5	Coverage (%) F6	Coverage (%) F7	Area F1	Area F2	Area F3	A
**173**	69823	TRINITY_DN77	157.32	31	0	0	0	31	0	0	3				1.[illegible]
**152**	69807	TRINITY_DN3925	157.17	14	10	5	10	3	0	0	0	1.8455E6	3.6002E5	4.3445E6	2.[illegible]
**152**	69808	TRINITY_DN3925	157.17	11	8	4	8	2	0	0	0	1.8455E6	3.6002E5	4.3445E6	2.[illegible]
**36**	419	APB93444.1	156.68	34	7	7	0	7	28	7	34				
**226**	69853	TRINITY_DN195	156.40	23	0	0	0	0	0	0	23				
**137**	69852	TRINITY_DN1793	155.08	21	4	0	0	21	10	0	10				1.[illegible]
**114**	69914	TRINITY_DN240	153.29	22	8	22	22	21	0	8	8		8.1563E6	4.8348E7	7.[illegible]
**114**	69914	TRINITY_DN240	153.29	22	8	22	22	21	0	8	8		8.1563E6	4.8348E7	7.[illegible]
**114**	69914	TRINITY_DN240	153.29	22	8	22	22	21	0	8	8		8.1563E6	4.8348E7	7.[illegible]
**114**	69914	TRINITY_DN240	153.29	22	8	22	22	21	0	8	8		8.1563E6	4.8348E7	7.[illegible]
**114**	69914	TRINITY_DN240	153.29	22	8	22	22	21	0	8	8		8.1563E6	4.8348E7	7.[illegible]
**114**	69919	TRINITY_DN240	153.29	22	8	22	22	21	0	8	8		8.1563E6	4.8348E7	7.[illegible]
**133**	468	ETE70217.1	151.88	1	0	0	0	1	1	1	1				
**246**	565	XP_034291429.1	148.76	5	5	0	0	0	0	0	0	6.0941E4			
**246**	566	XP_034291427.1	148.76	5	5	0	0	0	0	0	0	6.0941E4			
**246**	567	XP_034291428.1	148.76	5	5	0	0	0	0	0	0	6.0941E4			
**194**	69934	TRINITY_DN743	148.55	15	0	0	0	0	0	15	15				
**194**	69961	TRINITY_DN743	148.55	23	0	0	0	0	0	22	23				
**26**	69833	TRINITY_DN0	148.09	19	18	18	14	19	9	14	9	6.4332E6	1.8506E6	1.3941E8	5.[illegible]
**26**	69834	TRINITY_DN0	148.09	18	17	17	13	18	8	13	8	6.4332E6	1.8506E6	1.3941E8	5.[illegible]
**89**	918	CBM40646.1	146.67	12	0	5	9	7	0	3	0			0	
**89**	943	CBM40645.1	146.67	12	0	5	9	7	0	3	0			0	
**89**	944	AAR24534.1	146.67	12	0	5	9	7	0	3	0			0	
**89**	945	Q6T6S7.1	146.67	12	0	5	9	7	0	3	0			0	
**224**	69820	TRINITY_DN94	144.95	12	0	12	0	0	0	0	0		8.0435E5		
**95**	690	E5AJX2.1	137.19	13	0	4	9	8	0	0	0			5.4903E6	
**95**	691	CBW30778.1	137.19	13	0	4	9	8	0	0	0			5.4903E6	
**185**	69894	TRINITY_DN4675	137.11	13	0	0	0	0	13	0	0				

total 523 p

Protein Group	Protein ID	Accession	-10lgP	Coverage (%)	Coverage (%) F1	Coverage (%) F2	Coverage (%) F3	Coverage (%) F4	Coverage (%) F5	Coverage (%) F6	Coverage (%) F7	Area F1	Area F2	Area F3	A
**149**	890	TRINITY_DN59	137.06	22	0	0	0	0	22	0	0				
**149**	890	TRINITY_DN59	137.06	22	0	0	0	0	22	0	0				
**149**	891	AJO70724.1	137.06	18	0	0	0	0	18	0	0				
**49**	69863	TRINITY_DN588	135.41	20	12	6	6	11	6	6	14				
**49**	69863	TRINITY_DN588	135.41	20	12	6	6	11	6	6	14				
**218**	514	TRINITY_DN6726	131.08	30	0	0	0	0	6	0	30				
**201**	341	TRINITY_DN3927	130.90	5	4	0	0	2	0	0	0	9.7972E5			1.1
**167**	69841	TRINITY_DN2452	130.54	24	14	12	19	0	4	0	0	5.2396E5	1.7998E6	2.7782E6	
**48**	759	P82475.2	130.50	26	17	8	8	8	8	8	17				
**166**	69828	TRINITY_DN30	128.97	8	6	6	2	0	0	0	0		0		
**119**	844	JAS04521.1	128.01	12	0	0	5	8	12	12	3				
**119**	845	AAG12169.1	128.01	12	0	0	5	8	12	12	3				
**119**	998	JAG46131.1	128.01	12	0	0	5	8	12	12	3				
**119**	999	BAN82015.1	128.01	12	0	0	5	8	12	12	3				
**119**	1000	JAS04781.1	128.01	12	0	0	5	8	12	12	3				
**119**	1001	JAI10407.1	128.01	12	0	0	5	8	12	12	3				
**119**	1002	JAS05454.1	128.01	12	0	0	5	8	12	12	3				
**119**	1003	JAS04703.1	128.01	12	0	0	5	8	12	12	3				
**119**	1004	JAG46130.1	128.01	12	0	0	5	8	12	12	3				
**119**	1005	JAA98018.1	128.01	12	0	0	5	8	12	12	3				
**119**	1006	AAG30924.1	128.01	12	0	0	5	8	12	12	3				
**119**	1007	JAS05315.1	128.01	12	0	0	5	8	12	12	3				
**119**	1008	JAV01886.1	128.01	12	0	0	5	8	12	12	3				
**119**	1009	JAS04865.1	128.01	12	0	0	5	8	12	12	3				
**119**	1010	JAA98019.1	128.01	12	0	0	5	8	12	12	3				
**119**	1011	JAA94888.1	128.01	12	0	0	5	8	12	12	3				
**119**	1012	JAS04864.1	128.01	12	0	0	5	8	12	12	3				
**119**	1013	AFJ49196.1	128.01	12	0	0	5	8	12	12	3				
**119**	1014	JAS04866.1	128.01	12	0	0	5	8	12	12	3				

total 523 p

Protein Group	Protein ID	Accession	-10lgP	Coverage (%)	Coverage (%) F1	Coverage (%) F2	Coverage (%) F3	Coverage (%) F4	Coverage (%) F5	Coverage (%) F6	Coverage (%) F7	Area F1	Area F2	Area F3	A
**119**	1015	JAG43651.1	128.01	12	0	0	5	8	12	12	3				
**119**	1016	JAV48424.1	128.01	12	0	0	5	8	12	12	3				
**119**	1017	JAG46132.1	128.01	12	0	0	5	8	12	12	3				
**119**	1018	JAA97946.1	128.01	12	0	0	5	8	12	12	3				
**119**	982	BAP39981.1	128.01	10	0	0	4	6	10	10	2				
**119**	983	BAN82142.1	128.01	10	0	0	4	6	10	10	2				
**151**	638	CAP74088.1	127.36	14	0	0	0	14	6	0	9				3.1
**241**	518	ADJ67475.1	127.31	5	2	3	0	0	0	0	2				
**221**	69843	XP_034278116.1	126.21	5	0	0	5	2	0	0	0			2.9581E6	1.2
**138**	69858	TRINITY_DN12452	126.04	19	0	0	9	9	9	19	9			3.0778E5	5.5
**153**	515	AUS82489.1	125.09	11	0	0	7	8	4	3	0				1.(
**153**	557	JAA98031.1	125.09	11	0	0	7	8	4	3	0				1.(
**164**	1055	TRINITY_DN471	123.14	19	19	19	12	12	0	12	0	1.3286E6	4.833E6	1.0924E7	2.3
**117**	1178	ADN04917.1	122.90	8	8	8	8	4	0	4	0	6.4186E7	1.0631E7	3.0723E5	
**117**	1179	E0Y419.1	122.90	8	8	8	8	4	0	4	0	6.4186E7	1.0631E7	3.0723E5	
**123**	877	AEH59582.1	120.92	12	0	0	5	8	12	8	3				
**123**	866	QBF53420.1	120.92	13	0	0	5	8	13	8	3				
**123**	878	QHR82782.1	120.92	12	0	0	5	8	12	8	3				
**123**	882	TRINITY_DN1356	120.92	10	0	0	4	6	10	6	2				
**123**	882	TRINITY_DN1356	120.92	10	0	0	4	6	10	6	2				
**123**	882	TRINITY_DN1356	120.92	10	0	0	4	6	10	6	2				
**123**	882	TRINITY_DN1356	120.92	10	0	0	4	6	10	6	2				
**205**	69831	TRINITY_DN180	120.88	2	0	0	0	0	0	2	0		1.0785E4		1.2
**205**	69832	TRINITY_DN180	120.88	2	0	0	0	0	0	2	0		1.0785E4		1.2
**205**	454	XP_034285002.1	120.88	2	0	0	0	0	0	2	0		1.0785E4		1.2
**205**	456	XP_034285001.1	120.88	2	0	0	0	0	0	2	0		1.0785E4		1.2
**205**	453	XP_034285000.1	120.88	2	0	0	0	0	0	2	0		1.0785E4		1.2
**228**	988	JAG67762.1	120.52	20	0	0	0	0	0	0	20				
**228**	989	JAG67761.1	120.52	20	0	0	0	0	0	0	20				
**212**	928	XP_015679695.1	120.08	11	5	0	0	6	6	6	0	2.0856E5			1.(
**128**	722	ADP88559.1	117.89	11	3	3	3	8	0	3	8				1.6

total 523 p

Protein Group	Protein ID	Accession	-10lgP	Coverage (%)	Coverage (%) F1	Coverage (%) F2	Coverage (%) F3	Coverage (%) F4	Coverage (%) F5	Coverage (%) F6	Coverage (%) F7	Area F1	Area F2	Area F3	A
**128**	723	E5L0E3.1	117.89	11	3	3	3	8	0	3	8				1.6
**193**	914	P0DJL5.1	116.25	22	0	0	7	0	7	6	22			1.1179E5	
**193**	921	AMB36341.1	116.25	18	0	0	6	0	6	5	18			1.1179E5	
**92**	69869	TRINITY_DN554	114.68	20	0	7	7	12	12	20	20		9.8869E4	8.6025E5	1.4
**92**	69870	TRINITY_DN554	114.68	24	0	9	9	15	15	24	24		9.8869E4	8.6025E5	1.4
**210**	621	XP_015666085.1	114.12	8	5	3	3	3	0	0	0	3.4283E5	1.3335E5	8.9043E4	1.0
**210**	622	XP_015666086.1	114.12	8	5	3	3	3	0	0	0	3.4283E5	1.3335E5	8.9043E4	1.0
**210**	615	XP_034258446.1	114.12	8	5	3	3	3	0	0	0	3.4283E5	1.3335E5	8.9043E4	1.0
**210**	616	XP_034258445.1	114.12	8	5	3	3	3	0	0	0	3.4283E5	1.3335E5	8.9043E4	1.0
**210**	617	XP_034258444.1	114.12	8	5	3	3	3	0	0	0	3.4283E5	1.3335E5	8.9043E4	1.0
**210**	610	XP_032092626.1	114.12	8	5	3	3	3	0	0	0	3.4283E5	1.3335E5	8.9043E4	1.0
**210**	611	XP_032092625.1	114.12	8	5	3	3	3	0	0	0	3.4283E5	1.3335E5	8.9043E4	1.0
**210**	612	XP_032092628.1	114.12	8	5	3	3	3	0	0	0	3.4283E5	1.3335E5	8.9043E4	1.0
**210**	613	XP_013922774.1	114.12	8	5	3	3	3	0	0	0	3.4283E5	1.3335E5	8.9043E4	1.0
**210**	614	XP_032092627.1	114.12	8	5	3	3	3	0	0	0	3.4283E5	1.3335E5	8.9043E4	1.0
**210**	623	JAV50864.1	114.12	8	5	3	3	3	0	0	0	3.4283E5	1.3335E5	8.9043E4	1.0
**210**	624	JAI13760.1	114.12	8	5	3	3	3	0	0	0	3.4283E5	1.3335E5	8.9043E4	1.0
**210**	625	AFJ49766.1	114.12	8	5	3	3	3	0	0	0	3.4283E5	1.3335E5	8.9043E4	1.0
**210**	604	JAG47096.1	114.12	8	5	3	3	3	0	0	0	3.4283E5	1.3335E5	8.9043E4	1.0
**210**	626	TRINITY_DN8397	114.12	8	5	3	3	3	0	0	0	3.4283E5	1.3335E5	8.9043E4	1.0
**210**	609	ETE69371.1	114.12	9	6	3	3	3	0	0	0	3.4283E5	1.3335E5	8.9043E4	1.0
**210**	710	XP_026529384.1	114.12	8	5	3	3	3	0	0	0	3.4283E5	1.3335E5	8.9043E4	1.0
**210**	725	XP_026563995.1	114.12	8	5	3	3	3	0	0	0	3.4283E5	1.3335E5	8.9043E4	1.0
**88**	2092	P86497.1	114.04	15	0	15	15	15	0	0	0		1.4585E7	4.8687E7	1.4
**269**	949	JAG46182.1	109.91	6	0	6	0	0	0	0	0		1.1978E5		
**269**	950	XP_015674504.1	109.91	6	0	6	0	0	0	0	0		1.1978E5		
**269**	951	JAG43702.1	109.91	6	0	6	0	0	0	0	0		1.1978E5		
**269**	952	JAA96331.1	109.91	6	0	6	0	0	0	0	0		1.1978E5		
**269**	953	XP_015674505.1	109.91	6	0	6	0	0	0	0	0		1.1978E5		
**269**	954	JAV49839.1	109.91	6	0	6	0	0	0	0	0		1.1978E5		
**269**	69874	TRINITY_DN3815	109.91	6	0	6	0	0	0	0	0		1.1978E5		

total 523 p

Protein Group	Protein ID	Accession	-10lgP	Coverage (%)	Coverage (%) F1	Coverage (%) F2	Coverage (%) F3	Coverage (%) F4	Coverage (%) F5	Coverage (%) F6	Coverage (%) F7	Area F1	Area F2	Area F3	A[illegible]
**269**	69874	TRINITY_DN3815	109.91	6	0	6	0	0	0	0	0		1.1978E5		
**269**	69874	TRINITY_DN3815	109.91	6	0	6	0	0	0	0	0		1.1978E5		
**269**	69874	TRINITY_DN3815	109.91	6	0	6	0	0	0	0	0		1.1978E5		
**269**	69874	TRINITY_DN3815	109.91	6	0	6	0	0	0	0	0		1.1978E5		
**256**	684	TRINITY_DN28984	109.74	2	0	0	0	0	0	0	2				
**256**	69854	TRINITY_DN28984	109.74	2	0	0	0	0	0	0	2				
**280**	832	JAV50910.1	109.42	12	0	0	0	12	0	0	0				5.[illegible]
**280**	833	JAI10138.1	109.42	12	0	0	0	12	0	0	0				5.[illegible]
**280**	834	JAB54484.1	109.42	12	0	0	0	12	0	0	0				5.[illegible]
**280**	835	XP_026541233.1	109.42	12	0	0	0	12	0	0	0				5.[illegible]
**280**	836	XP_026576189.1	109.42	12	0	0	0	12	0	0	0				5.[illegible]
**280**	837	LAB45561.1	109.42	11	0	0	0	11	0	0	0				5.[illegible]
**280**	69868	TRINITY_DN178	109.42	9	0	0	0	9	0	0	0				5.[illegible]
**280**	1039	LAB63521.1	109.42	12	0	0	0	12	0	0	0				5.[illegible]
**280**	1040	JAG68347.1	109.42	12	0	0	0	12	0	0	0				5.[illegible]
**280**	1041	ETE58466.1	109.42	10	0	0	0	10	0	0	0				5.[illegible]
**258**	69851	TRINITY_DN7999	108.70	7	0	0	0	0	0	0	7				
**240**	498	JAV50725.1	105.94	6	0	0	2	0	0	0	4			9.5286E4	
**127**	1155	ADP88560.1	105.88	10	10	10	4	4	6	4	6	3.9743E5	3.772E5		
**127**	1156	E5L0E4.1	105.88	10	10	10	4	4	6	4	6	3.9743E5	3.772E5		
**262**	695	XP_026535564.1	105.27	3	0	0	0	0	0	0	3				
**262**	696	XP_026535563.1	105.27	3	0	0	0	0	0	0	3				
**262**	655	XP_013918193.1	105.27	3	0	0	0	0	0	0	3				
**262**	598	XP_034271810.1	105.27	3	0	0	0	0	0	0	3				
**262**	705	XP_013918195.1	105.27	3	0	0	0	0	0	0	3				
**262**	678	XP_013918194.1	105.27	3	0	0	0	0	0	0	3				
**262**	637	XP_034271811.1	105.27	3	0	0	0	0	0	0	3				
**262**	706	XP_029143180.1	105.27	3	0	0	0	0	0	0	3				
**242**	452	JAC95757.1	104.83	9	0	0	3	2	0	0	4			1.1168E5	8.[illegible]
**202**	1548	ADI47590.1	104.60	5	5	3	5	0	0	0	0	1.4223E6	4.9549E5	1.8109E6	

total 523 p

Protein Group	Protein ID	Accession	-10lgP	Coverage (%)	Coverage (%) F1	Coverage (%) F2	Coverage (%) F3	Coverage (%) F4	Coverage (%) F5	Coverage (%) F6	Coverage (%) F7	Area F1	Area F2	Area F3	A
**110**	1500	E0Y421.1	103.68	8	4	4	4	4	0	0	0	1.8692E7			
**110**	1501	ADN04919.1	103.68	8	4	4	4	4	0	0	0	1.8692E7			
**110**	1707	QBF53413.1	103.68	8	4	4	4	4	0	0	0	1.8692E7			
**191**	1327	XP_007435739.1	103.31	4	2	2	2	4	2	2	2	6.6638E5	2.0438E5	3.7003E5	1.4
**191**	1555	XP_015672252.1	103.31	4	2	2	2	4	2	2	2	6.6638E5	2.0438E5	3.7003E5	1.4
**191**	1528	JAG44290.1	103.31	4	2	2	2	4	2	2	2	6.6638E5	2.0438E5	3.7003E5	1.4
**191**	69929	TRINITY_DN6008	103.31	4	2	2	2	4	2	2	2	6.6638E5	2.0438E5	3.7003E5	1.4
**191**	1538	AFJ50411.1	103.31	4	2	2	2	4	2	2	2	6.6638E5	2.0438E5	3.7003E5	1.4
**192**	70234	TRINITY_DN2736	103.13	14	14	14	14	14	0	0	0	1.8674E5	1.5622E6	7.1135E5	5.8
**177**	69888	TRINITY_DN471	102.97	11	11	11	11	11	11	0	0	4.0439E6	6.1514E6	1.3556E7	4.8
**264**	69847	TRINITY_DN1497	102.43	2	2	0	0	0	0	0	0	1.3496E5			
**264**	69848	TRINITY_DN1497	102.43	3	3	0	0	0	0	0	0	1.3496E5			
**238**	639	ETE68218.1	95.05	4	1	0	0	1	0	4	0	5.5682E4			1.1
**238**	651	XP_025022318.1	95.05	4	1	0	0	1	0	4	0	5.5682E4			1.1
**238**	656	JAG67632.1	95.05	4	1	0	0	1	0	4	0	5.5682E4			1.1
**238**	664	JAG44306.1	95.05	4	1	0	0	1	0	4	0	5.5682E4			1.1
**238**	665	JAA96758.1	95.05	4	1	0	0	1	0	4	0	5.5682E4			1.1
**238**	666	JAG46538.1	95.05	4	1	0	0	1	0	4	0	5.5682E4			1.1
**238**	676	LAB43566.1	95.05	4	1	0	0	1	0	4	0	5.5682E4			1.1
**238**	654	JAV50234.1	95.05	4	1	0	0	1	0	4	0	5.5682E4			1.1
**238**	657	XP_026576301.1	95.05	4	1	0	0	1	0	4	0	5.5682E4			1.1
**238**	658	XP_026525656.1	95.05	4	1	0	0	1	0	4	0	5.5682E4			1.1
**238**	659	XP_026576299.1	95.05	4	1	0	0	1	0	4	0	5.5682E4			1.1
**238**	660	XP_026525655.1	95.05	4	1	0	0	1	0	4	0	5.5682E4			1.1
**238**	661	XP_026525654.1	95.05	4	1	0	0	1	0	4	0	5.5682E4			1.1
**238**	662	XP_026576298.1	95.05	4	1	0	0	1	0	4	0	5.5682E4			1.1
**238**	663	XP_013919280.1	95.05	4	1	0	0	1	0	4	0	5.5682E4			1.1
**238**	667	TRINITY_DN206	95.05	4	1	0	0	1	0	4	0	5.5682E4			1.1
**238**	667	TRINITY_DN206	95.05	4	1	0	0	1	0	4	0	5.5682E4			1.1
**238**	668	XP_032091066.1	95.05	4	1	0	0	1	0	4	0	5.5682E4			1.1
**238**	669	XP_032091065.1	95.05	4	1	0	0	1	0	4	0	5.5682E4			1.1
**238**	670	XP_034271617.1	95.05	4	1	0	0	1	0	4	0	5.5682E4			1.1

total 523 p

Protein Group	Protein ID	Accession	-10lgP	Coverage (%)	Coverage (%) F1	Coverage (%) F2	Coverage (%) F3	Coverage (%) F4	Coverage (%) F5	Coverage (%) F6	Coverage (%) F7	Area F1	Area F2	Area F3	A
**238**	671	XP_034271615.1	95.05	4	1	0	0	1	0	4	0	5.5682E4			1.1
**238**	672	XP_034271618.1	95.05	4	1	0	0	1	0	4	0	5.5682E4			1.1
**238**	673	XP_034271616.1	95.05	4	1	0	0	1	0	4	0	5.5682E4			1.1
**238**	674	XP_025022317.1	95.05	4	1	0	0	1	0	4	0	5.5682E4			1.1
**238**	675	XP_007428111.1	95.05	4	1	0	0	1	0	4	0	5.5682E4			1.1
**148**	70063	TRINITY_DN459	94.14	11	11	11	11	11	11	0	0	3.6789E6	3.4934E5	9.2485E5	6.1
**148**	70205	TRINITY_DN459	94.14	9	9	9	9	9	9	0	0	3.6789E6	3.4934E5	9.2485E5	6.1
**148**	2962	TRINITY_DN3041	94.14	31	31	31	31	31	31	0	0	3.6789E6	3.4934E5	9.2485E5	6.1
**274**	1102	TRINITY_DN2	93.78	5	0	0	2	3	0	0	0			7.0205E4	3.8
**257**	69921	TRINITY_DN7999	93.52	3	0	0	0	0	0	0	3				
**257**	69922	TRINITY_DN7999	93.52	3	0	0	0	0	0	0	3				
**265**	1267	TRINITY_DN13494	92.73	7	0	7	0	0	0	0	0		7.3443E5		
**254**	2971	A8Y7P1.1	92.20	12	0	0	0	0	0	0	12				
**254**	2972	CAL69604.1	92.20	12	0	0	0	0	0	0	12				
**254**	2973	A8Y7N6.1	92.20	12	0	0	0	0	0	0	12				
**254**	2974	CAL69609.1	92.20	12	0	0	0	0	0	0	12				
**254**	2975	A8Y7P5.1	92.20	11	0	0	0	0	0	0	11				
**254**	2976	AFD04724.1	92.20	11	0	0	0	0	0	0	11				
**254**	2977	CAL69613.1	92.20	11	0	0	0	0	0	0	11				
**254**	2978	A8Y7N7.1	92.20	11	0	0	0	0	0	0	11				
**254**	2979	CAL69605.1	92.20	11	0	0	0	0	0	0	11				
**233**	69924	TRINITY_DN960	90.47	8	0	0	0	0	8	4	0				
**233**	69925	TRINITY_DN960	90.47	7	0	0	0	0	7	3	0				
**233**	69926	TRINITY_DN960	90.47	6	0	0	0	0	6	3	0				

total 523 p

Protein Group	Protein ID	Accession	-10lgP	Coverage (%)	Coverage (%) F1	Coverage (%) F2	Coverage (%) F3	Coverage (%) F4	Coverage (%) F5	Coverage (%) F6	Coverage (%) F7	Area F1	Area F2	Area F3	A
**233**	69927	TRINITY_DN960	90.47	5	0	0	0	0	5	3	0				
**233**	69956	TRINITY_DN960	90.47	7	0	0	0	0	7	3	0				
**233**	69957	TRINITY_DN960	90.47	6	0	0	0	0	6	3	0				
**233**	69958	TRINITY_DN960	90.47	6	0	0	0	0	6	3	0				
**244**	69895	TRINITY_DN672	89.92	5	0	0	0	0	0	0	5				
**275**	1984	1OQS	89.80	14	0	0	0	0	0	0	14				
**275**	1984	1OQS	89.80	14	0	0	0	0	0	0	14				
**275**	1984	1OQS	89.80	14	0	0	0	0	0	0	14				
**275**	1984	1OQS	89.80	14	0	0	0	0	0	0	14				
**275**	1986	CAA48456.1	89.80	12	0	0	0	0	0	0	12				
**275**	1987	AAZ53185.1	89.80	12	0	0	0	0	0	0	12				
**275**	1988	Q02471.1	89.80	12	0	0	0	0	0	0	12				
**275**	1989	S29298	89.80	12	0	0	0	0	0	0	12				
**275**	1990	ACD43467.1	89.80	12	0	0	0	0	0	0	12				
**275**	1991	ACD43465.1	89.80	12	0	0	0	0	0	0	12				
**203**	1737	AFD04723.1	87.78	11	0	0	0	10	10	10	11				8.1
**203**	1738	AFB74191.1	87.78	11	0	0	0	10	10	10	11				8.1
**203**	1739	A8Y7N4.1	87.78	11	0	0	0	10	10	10	11				8.1
**203**	1740	H6VC05.1	87.78	11	0	0	0	10	10	10	11				8.1
**203**	1741	CAL69602.1	87.78	11	0	0	0	10	10	10	11				8.1
**207**	1970	ABD24041.1	86.71	26	26	13	0	0	0	0	11	3.3819E5	1.6087E5		
**207**	1971	Q2ES49.1	86.71	26	26	13	0	0	0	0	11	3.3819E5	1.6087E5		
**255**	842	TRINITY_DN157	86.44	13	0	0	0	0	0	0	13				
**213**	1995	AAB36402.1	85.29	13	0	0	0	0	0	0	13				

total 523 p

Protein Group	Protein ID	Accession	-10lgP	Coverage (%)	Coverage (%) F1	Coverage (%) F2	Coverage (%) F3	Coverage (%) F4	Coverage (%) F5	Coverage (%) F6	Coverage (%) F7	Area F1	Area F2	Area F3	A
**213**	1996	Q9PSM8.1	85.29	13	0	0	0	0	0	0	13				
**284**	1099	XP_029141923.1	81.62	7	0	0	0	0	0	0	7				
**267**	69887	TRINITY_DN1424	81.20	5	0	0	0	0	5	0	5				
**215**	997	ART88739.1	79.86	7	0	4	3	0	0	0	0		1.254E5		
**296**	69884	TRINITY_DN1526	79.05	7	0	0	0	0	0	0	7				
**296**	69885	TRINITY_DN1526	79.05	6	0	0	0	0	0	0	6				
**296**	69886	TRINITY_DN1526	79.05	5	0	0	0	0	0	0	5				
**236**	70327	TRINITY_DN2452	77.88	9	0	8	0	8	9	8	0		3.2643E4		4.1
**187**	3019	P00990.1	76.97	17	0	0	15	15	15	15	17			4.065E5	8.(
**187**	3020	TIVRV2	76.97	17	0	0	15	15	15	15	17			4.065E5	8.(
**187**	3028	CAL69611.1	76.97	12	0	0	11	11	11	11	12			4.065E5	8.(
**187**	3029	A8Y7P3.1	76.97	12	0	0	11	11	11	11	12			4.065E5	8.(
**187**	2948	CAL69610.1	76.97	12	0	0	11	11	11	11	12			4.065E5	8.(
**187**	2949	A8Y7P2.1	76.97	12	0	0	11	11	11	11	12			4.065E5	8.(
**187**	3030	CAL69615.1	76.97	12	0	0	11	11	11	11	12			4.065E5	8.(
**187**	3031	AFE83617.1	76.97	12	0	0	11	11	11	11	12			4.065E5	8.(
**187**	3032	A8Y7P4.1	76.97	12	0	0	11	11	11	11	12			4.065E5	8.(
**187**	3033	AFE83616.1	76.97	12	0	0	11	11	11	11	12			4.065E5	8.(
**187**	3034	AFB74192.1	76.97	12	0	0	11	11	11	11	12			4.065E5	8.(
**187**	3035	CAL69614.1	76.97	12	0	0	11	11	11	11	12			4.065E5	8.(
**187**	3036	A8Y7P6.1	76.97	12	0	0	11	11	11	11	12			4.065E5	8.(
**187**	3037	A8Y7N5.1	76.97	12	0	0	11	11	11	11	12			4.065E5	8.(
**187**	3038	CAL69608.1	76.97	12	0	0	11	11	11	11	12			4.065E5	8.(
**187**	3039	ABD24040.1	76.97	12	0	0	11	11	11	11	12			4.065E5	8.(
**187**	3040	CAL69612.1	76.97	12	0	0	11	11	11	11	12			4.065E5	8.(

total 523 p

Protein Group	Protein ID	Accession	-10lgP	Coverage (%)	Coverage (%) F1	Coverage (%) F2	Coverage (%) F3	Coverage (%) F4	Coverage (%) F5	Coverage (%) F6	Coverage (%) F7	Area F1	Area F2	Area F3	A
**187**	3041	A8Y7P0.1	76.97	12	0	0	11	11	11	11	12			4.065E5	8.[illegible]
**187**	3042	AFE83615.1	76.97	12	0	0	11	11	11	11	12			4.065E5	8.[illegible]
**187**	3043	Q2ES50.1	76.97	12	0	0	11	11	11	11	12			4.065E5	8.[illegible]
**187**	3044	CAL69603.1	76.97	12	0	0	11	11	11	11	12			4.065E5	8.[illegible]
**187**	3045	H6VC06.1	76.97	12	0	0	11	11	11	11	12			4.065E5	8.[illegible]
**307**	70026	TRINITY_DN5102	76.41	3	0	3	0	0	0	0	0		8.6583E4		
**229**	1035	CAE47141.1	75.31	12	0	0	0	0	0	0	12				
**252**	955	ABB76279.1	73.14	9	0	0	0	9	0	0	0				7.1[illegible]
**230**	1518	ADI47595.1	73.04	6	6	0	0	0	0	0	0	4.3022E6			
**230**	1676	ADI47604.1	73.04	6	6	0	0	0	0	0	0	4.3022E6			
**297**	69935	TRINITY_DN722	72.61	3	0	3	0	0	0	0	0		3.0191E5		
**297**	70165	TRINITY_DN722	72.61	11	0	11	0	0	0	0	0		3.0191E5		
**297**	70166	TRINITY_DN722	72.61	9	0	9	0	0	0	0	0		3.0191E5		
**261**	1482	TRINITY_DN86540	70.73	11	0	0	0	0	4	0	11				
**261**	1972	P0C0U6.1	70.73	16	0	0	0	0	6	0	16				
**261**	1973	XP_026532450.1	70.73	15	0	0	0	0	6	0	15				
**261**	1974	XP_026570824.1	70.73	15	0	0	0	0	6	0	15				
**261**	1975	XP_034284997.1	70.73	15	0	0	0	0	6	0	15				
**261**	1976	XP_032079120.1	70.73	15	0	0	0	0	6	0	15				
**261**	1977	JAC95434.1	70.73	15	0	0	0	0	6	0	15				
**261**	1978	LAA66838.1	70.73	14	0	0	0	0	6	0	14				
**290**	1384	XP_029140879.1	70.10	0	0	0	0	0	0	0	0				
**248**	1817	AJO70722.1	68.21	6	0	0	0	0	0	0	6				
**343**	2091	XP_029142225.1	64.45	1	0	0	1	0	0	0	0			9.5811E4	
**343**	2123	XP_015677107.1	64.45	3	0	0	3	0	0	0	0			9.5811E4	
**343**	70209	TRINITY_DN1914	64.45	3	0	0	3	0	0	0	0			9.5811E4	
**403**	2994	TRINITY_DN1889	63.54	8	0	0	0	0	0	0	8				
**200**	3016	CCH15044.1	63.13	9	9	9	9	9	9	9	9	8.2206E5	5.5535E5	2.9889E6	2.[illegible]

total 523 p

Protein Group	Protein ID	Accession	-10lgP	Coverage (%)	Coverage (%) F1	Coverage (%) F2	Coverage (%) F3	Coverage (%) F4	Coverage (%) F5	Coverage (%) F6	Coverage (%) F7	Area F1	Area F2	Area F3	A
**200**	3017	I2G9B4.1	63.13	9	9	9	9	9	9	9	9	8.2206E5	5.5535E5	2.9889E6	2.3
**200**	3010	AMH40736.1	63.13	10	10	10	10	10	10	10	10	8.2206E5	5.5535E5	2.9889E6	2.3
**200**	3011	AMH40741.1	63.13	10	10	10	10	10	10	10	10	8.2206E5	5.5535E5	2.9889E6	2.3
**200**	3012	P00991.2	63.13	10	10	10	10	10	10	10	10	8.2206E5	5.5535E5	2.9889E6	2.3
**200**	3013	AAP04484.1	63.13	10	10	10	10	10	10	10	10	8.2206E5	5.5535E5	2.9889E6	2.3
**200**	2915	AAP04485.1	63.13	10	10	10	10	10	10	10	10	8.2206E5	5.5535E5	2.9889E6	2.3
**200**	2916	P00992.2	63.13	10	10	10	10	10	10	10	10	8.2206E5	5.5535E5	2.9889E6	2.3
**200**	2917	AMH40733.1	63.13	10	10	10	10	10	10	10	10	8.2206E5	5.5535E5	2.9889E6	2.3
**200**	2918	AMH40738.1	63.13	10	10	10	10	10	10	10	10	8.2206E5	5.5535E5	2.9889E6	2.3
**200**	2919	AMH40740.1	63.13	10	10	10	10	10	10	10	10	8.2206E5	5.5535E5	2.9889E6	2.3
**200**	3014	E5AJX3.1	63.13	9	9	9	9	9	9	9	9	8.2206E5	5.5535E5	2.9889E6	2.3
**200**	3015	CBW30779.1	63.13	9	9	9	9	9	9	9	9	8.2206E5	5.5535E5	2.9889E6	2.3
**200**	70321	TRINITY_DN151	63.13	8	8	8	8	8	8	8	8	8.2206E5	5.5535E5	2.9889E6	2.3
**200**	3047	Q6T269.1	63.13	6	6	6	6	6	6	6	6	8.2206E5	5.5535E5	2.9889E6	2.3
**200**	3048	AAR19275.1	63.13	6	6	6	6	6	6	6	6	8.2206E5	5.5535E5	2.9889E6	2.3
**245**	1804	LAA93895.1	61.86	5	5	0	0	5	5	5	5	2.8419E4			7.8
**285**	2986	CAJ34939.1	61.72	19	0	0	0	0	0	19	19				
**285**	2987	Q3BK14.1	61.72	19	0	0	0	0	0	19	19				
**285**	70319	TRINITY_DN160	61.72	15	0	0	0	0	0	15	15				
**223**	1689	JAS05406.1	60.18	2	0	2	0	2	0	0	0		2.6453E5		1.0
**223**	1667	JAS05289.1	60.18	2	0	2	0	2	0	0	0		2.6453E5		1.0
**223**	1668	JAS05287.1	60.18	2	0	2	0	2	0	0	0		2.6453E5		1.0
**223**	1669	ABG26980.1	60.18	2	0	2	0	2	0	0	0		2.6453E5		1.0
**223**	1670	JAS05286.1	60.18	2	0	2	0	2	0	0	0		2.6453E5		1.0
**223**	1671	JAS05288.1	60.18	2	0	2	0	2	0	0	0		2.6453E5		1.0
**223**	1672	JAS05285.1	60.18	2	0	2	0	2	0	0	0		2.6453E5		1.0
**223**	1734	JAS05407.1	60.18	2	0	2	0	2	0	0	0		2.6453E5		1.0
**223**	1735	JAS05408.1	60.18	2	0	2	0	2	0	0	0		2.6453E5		1.0
**223**	2063	QIV64953.1	60.18	4	0	4	0	4	0	0	0		2.6453E5		1.0

total 523 p

Protein Group	Protein ID	Accession	-10lgP	Coverage (%)	Coverage (%) F1	Coverage (%) F2	Coverage (%) F3	Coverage (%) F4	Coverage (%) F5	Coverage (%) F6	Coverage (%) F7	Area F1	Area F2	Area F3	A
**303**	1894	ETE57622.1	55.32	3	0	0	0	0	0	0	3				
**376**	70075	XP_015672332.1	52.38	2	2	0	0	0	0	0	0	7.951E4			
**374**	2170	TRINITY_DN27458	51.48	2	0	0	0	2	0	0	0				1.[illegible]
**374**	3082	XP_015683432.1	51.48	2	0	0	0	2	0	0	0				1.[illegible]
**378**	2213	XP_007421015.1	50.81	1	1	0	0	0	0	0	0	1.4791E5			
**378**	70211	TRINITY_DN13676	50.81	2	2	0	0	0	0	0	0	1.4791E5			
**378**	70212	TRINITY_DN13676	50.81	1	1	0	0	0	0	0	0	1.4791E5			
**378**	70212	TRINITY_DN13676	50.81	1	1	0	0	0	0	0	0	1.4791E5			
**378**	70214	TRINITY_DN13676	50.81	1	1	0	0	0	0	0	0	1.4791E5			
**378**	70215	TRINITY_DN13676	50.81	1	1	0	0	0	0	0	0	1.4791E5			
**378**	2289	JAV50100.1	50.81	1	1	0	0	0	0	0	0	1.4791E5			
**378**	3080	LAB58574.1	50.81	9	9	0	0	0	0	0	0	1.4791E5			
**378**	2671	XP_007421016.1	50.81	1	1	0	0	0	0	0	0	1.4791E5			
**378**	2545	XP_026552070.1	50.81	1	1	0	0	0	0	0	0	1.4791E5			
**378**	2254	JAA96625.1	50.81	1	1	0	0	0	0	0	0	1.4791E5			
**378**	2546	XP_026537394.1	50.81	1	1	0	0	0	0	0	0	1.4791E5			
**378**	2255	JAG44103.1	50.81	1	1	0	0	0	0	0	0	1.4791E5			
**378**	2547	XP_026537257.1	50.81	1	1	0	0	0	0	0	0	1.4791E5			
**378**	2596	LAB27223.1	50.81	1	1	0	0	0	0	0	0	1.4791E5			
**378**	1935	XP_032065032.1	50.81	1	1	0	0	0	0	0	0	1.4791E5			
**378**	2302	XP_034262785.1	50.81	1	1	0	0	0	0	0	0	1.4791E5			
**378**	1936	XP_013919874.1	50.81	1	1	0	0	0	0	0	0	1.4791E5			
**295**	1454	JAV49704.1	50.02	2	0	0	0	0	0	0	2				
**295**	1473	JAA96208.1	50.02	2	0	0	0	0	0	0	2				
**295**	69903	TRINITY_DN159	50.02	2	0	0	0	0	0	0	2				
**295**	69904	TRINITY_DN159	50.02	2	0	0	0	0	0	0	2				
**295**	69905	TRINITY_DN159	50.02	2	0	0	0	0	0	0	2				
**295**	1488	AFJ50884.1	50.02	2	0	0	0	0	0	0	2				
**295**	1489	JAI12151.1	50.02	2	0	0	0	0	0	0	2				
**270**	2182	JAG67907.1	49.83	2	0	0	0	2	0	2	0				1.[illegible]
**270**	3150	TRINITY_DN3991	49.83	2	0	0	0	2	0	2	0				1.[illegible]
**298**	2284	CAJ01683.1	48.76	3	0	0	3	0	0	0	0			2.178E5	
**298**	2041	ADW54336.1	48.76	3	0	0	3	0	0	0	0			2.178E5	

total 523 p

Protein Group	Protein ID	Accession	-10lgP	Coverage (%)	Coverage (%) F1	Coverage (%) F2	Coverage (%) F3	Coverage (%) F4	Coverage (%) F5	Coverage (%) F6	Coverage (%) F7	Area F1	Area F2	Area F3	A
**298**	2225	ADW54337.1	48.76	4	0	0	4	0	0	0	0			2.178E5	
**298**	2593	ADW54335.1	48.76	4	0	0	4	0	0	0	0			2.178E5	
**169**	70050	P86541.2	47.76	13	13	13	0	13	13	13	13	1.9149E5	2.2353E4		3.2
**169**	2070	P01440.1	47.76	13	13	13	0	13	13	13	13	1.9149E5	2.2353E4		3.2
**169**	2071	AAC61315.1	47.76	13	13	13	0	13	13	13	13	1.9149E5	2.2353E4		3.2
**169**	2073	BAU24665.1	47.76	11	11	11	0	11	11	11	11	1.9149E5	2.2353E4		3.2
**169**	2075	Q93472.1	47.76	10	10	10	0	10	10	10	10	1.9149E5	2.2353E4		3.2
**169**	2076	Q9PST3.1	47.76	10	10	10	0	10	10	10	10	1.9149E5	2.2353E4		3.2
**169**	2077	AAK49439.1	47.76	10	10	10	0	10	10	10	10	1.9149E5	2.2353E4		3.2
**169**	2078	AAC27686.1	47.76	10	10	10	0	10	10	10	10	1.9149E5	2.2353E4		3.2
**169**	2079	Q9PST4.1	47.76	10	10	10	0	10	10	10	10	1.9149E5	2.2353E4		3.2
**169**	2080	AAC27687.1	47.76	10	10	10	0	10	10	10	10	1.9149E5	2.2353E4		3.2
**169**	2081	AAC27685.1	47.76	10	10	10	0	10	10	10	10	1.9149E5	2.2353E4		3.2
**283**	2262	ETE69494.1	47.54	2	0	0	0	2	2	2	0				4.7
**283**	3123	ETE65060.1	47.54	7	0	0	0	7	7	7	0				4.7
**283**	3124	XP_013915314.1	47.54	7	0	0	0	7	7	7	0				4.7
**283**	3102	XP_013919189.1	47.54	7	0	0	0	7	7	7	0				4.7
**283**	3103	XP_013919188.1	47.54	7	0	0	0	7	7	7	0				4.7
**283**	3104	ETE65301.1	47.54	7	0	0	0	7	7	7	0				4.7
**283**	3105	JAG46644.1	47.54	7	0	0	0	7	7	7	0				4.7
**283**	3106	JAI09932.1	47.54	7	0	0	0	7	7	7	0				4.7
**283**	70333	XP_007430054.1	47.54	7	0	0	0	7	7	7	0				4.7
**283**	3107	JAI13025.1	47.54	7	0	0	0	7	7	7	0				4.7
**283**	3108	AFJ50305.1	47.54	7	0	0	0	7	7	7	0				4.7
**283**	3109	JAG44453.1	47.54	7	0	0	0	7	7	7	0				4.7
**283**	3110	JAI09933.1	47.54	7	0	0	0	7	7	7	0				4.7
**283**	3111	JAG46643.1	47.54	7	0	0	0	7	7	7	0				4.7
**283**	3112	AFJ50304.1	47.54	7	0	0	0	7	7	7	0				4.7
**283**	3113	JAG67733.1	47.54	7	0	0	0	7	7	7	0				4.7
**283**	3114	JAB54108.1	47.54	7	0	0	0	7	7	7	0				4.7
**283**	3115	JAC95409.1	47.54	7	0	0	0	7	7	7	0				4.7
**283**	3116	JAI13026.1	47.54	7	0	0	0	7	7	7	0				4.7
**283**	3117	JAA96854.1	47.54	7	0	0	0	7	7	7	0				4.7
**283**	3118	JAG44452.1	47.54	7	0	0	0	7	7	7	0				4.7
**283**	3119	JAA96855.1	47.54	7	0	0	0	7	7	7	0				4.7
**283**	3120	XP_013930707.1	47.54	7	0	0	0	7	7	7	0				4.7

total 523 p

Protein Group	Protein ID	Accession	-10lgP	Coverage (%)	Coverage (%) F1	Coverage (%) F2	Coverage (%) F3	Coverage (%) F4	Coverage (%) F5	Coverage (%) F6	Coverage (%) F7	Area F1	Area F2	Area F3	A
**283**	3122	XP_013931116.1	47.54	7	0	0	0	7	7	7	0				4.7
**283**	3125	XP_013908942.1	47.54	7	0	0	0	7	7	7	0				4.7
**283**	3100	XP_013911300.1	47.54	6	0	0	0	6	6	6	0				4.7
**283**	3127	ETE60792.1	47.54	6	0	0	0	6	6	6	0				4.7
**283**	3129	XP_013924759.1	47.54	6	0	0	0	6	6	6	0				4.7
**283**	70335	XP_026542761.1	47.54	6	0	0	0	6	6	6	0				4.7
**283**	3130	ETE59971.1	47.54	5	0	0	0	5	5	5	0				4.7
**283**	2863	XP_013921022.1	47.54	5	0	0	0	5	5	5	0				4.7
**283**	3131	LAB40587.1	47.54	5	0	0	0	5	5	5	0				4.7
**404**	70338	TRINITY_DN1911	46.25	25	0	0	0	0	25	0	0				
**404**	70338	TRINITY_DN1911	46.25	25	0	0	0	0	25	0	0				
**305**	70262	XP_026548138.1	45.78	1	0	0	0	0	0	0	1				
**375**	2015	XP_032092456.1	42.06	5	0	0	0	0	0	0	5				
**304**	2452	XP_007437884.2	40.82	1	0	0	0	0	0	1	0				
**304**	2453	XP_007437883.2	40.82	1	0	0	0	0	0	1	0				
**304**	2910	XP_007437886.2	40.82	1	0	0	0	0	0	1	0				
**304**	2911	XP_007437885.2	40.82	1	0	0	0	0	0	1	0				
**165**	2674	XP_007421854.1	39.14	2	0	0	0	2	2	0	0				4.3

total 523 p

TRINITY_DN9
back to list

| Protein Coverage | Supporting Peptides |
Protein Coverage:

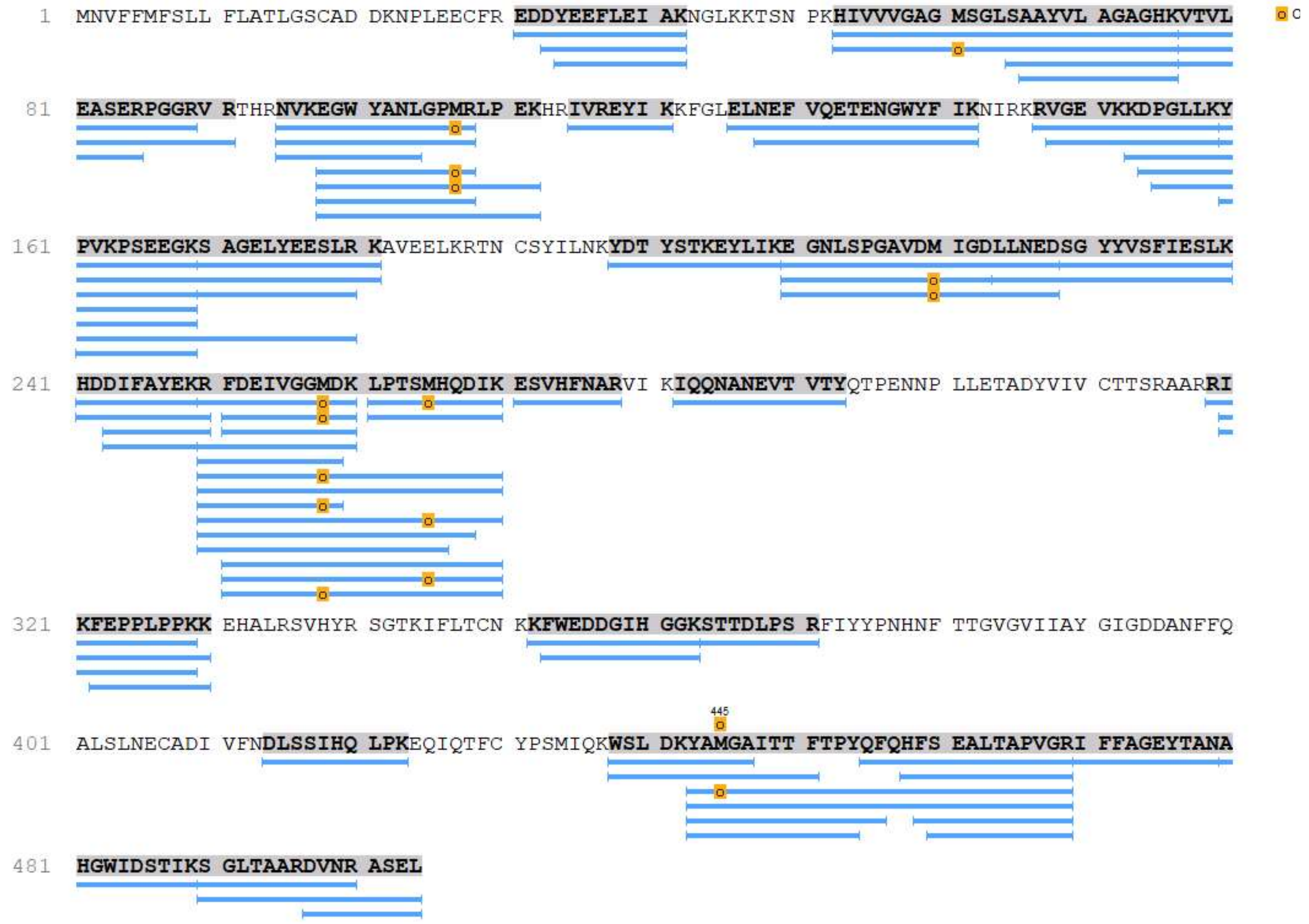

Supporting Peptides:

Peptide	Uniq	-10lgP	Mass	Length	ppm	m/z	z	RT	Fraction	Scan	Source File
K.YPVKPSEEGKSAGELYEESLRK.A	Y	103.19	2495.2544	22	0.3	624.8210	4	41.37	1	F1:12431	12112021_RID_2349_DP1.raw
K.YAMGAITTFTPYQFQHFSEALTAPVGR.I	N	93.35	3003.4587	27	0.8	1002.1609	3	68.16	3	F3:20530	17112021_RID_2349_DP3.raw
R.FDEIVGGMDKLPTSMHQDIK.E	Y	92.44	2260.0867	20	0.3	754.3698	3	47.85	3	F3:13952	17112021_RID_2349_DP3.raw
K.DPGLLKYPVKPSEEGK.S	N	90.01	1755.9407	16	1.1	586.3215	3	37.86	1	F1:11209	12112021_RID_2349_DP1.raw
R.NVKEGWYANLGPMR.L	N	89.97	1633.8035	14	1.1	817.9099	2	46.58	1	F1:14171	12112021_RID_2349_DP1.raw
K.SGLTAARDVNRASEL	N	81.95	1558.8063	15	-0.1	520.6093	3	35.16	1	F1:10323	12112021_RID_2349_DP1.raw
K.RFDEIVGGMDK.L	N	81.31	1265.6074	11	0.1	633.8110	2	38.73	1	F1:11503	12112021_RID_2349_DP1.raw
K.YDTYSTKEYLIK.E	N	80.80	1522.7555	12	0.9	762.3857	2	38.58	2	F2:10545	12112021_RID_2349_DP2.raw
K.HIVVVGAGMSGLSAAYVLAGAGHK.V	Y	80.72	2264.2100	24	-0.3	755.7437	3	58.30	3	F3:17243	17112021_RID_2349_DP3.raw
K.KDPGLLKYPVKPSEEGK.S	N	80.37	1884.0355	17	-0.1	472.0161	4	43.31	1	F1:13296	12112021_RID_2349_DP1.raw
K.YAM(+15.99)GAITTFTPYQFQHFSEALTAPVGR.I	N	80.23	3019.4539	27	0.9	1007.4928	3	64.54	3	F3:19257	17112021_RID_2349_DP3.raw
K.KFWEDDGIHGGK.S	N	78.30	1387.6520	12	0.2	694.8334	2	31.47	3	F3:8478	17112021_RID_2349_DP3.raw
K.SAGELYEESLRK.A	Y	76.14	1380.6885	12	0.1	691.3516	2	32.27	1	F1:9527	12112021_RID_2349_DP1.raw
K.RFDEIVGGMDKLPTSMHQDIK.E	Y	75.67	2416.1877	21	0.3	605.0544	4	49.40	3	F3:14259	17112021_RID_2349_DP3.raw
K.FWEDDGIHGGK.S	N	74.52	1259.5570	11	0.6	630.7861	2	33.77	2	F2:8859	12112021_RID_2349_DP2.raw
K.EGWYANLGPMR.L	N	74.35	1292.5972	11	0.2	647.3060	2	50.70	1	F1:15490	12112021_RID_2349_DP1.raw
R.EDDYEEFLEIAK.N	N	74.11	1499.6667	12	0.7	750.8412	2	59.88	5	F5:17908	17112021_RID_2349_DP5.raw
K.LPTSMHQDIK.E	Y	73.02	1168.5911	10	0.7	585.3032	2	26.70	2	F2:6395	12112021_RID_2349_DP2.raw

Peptide	Uniq	-10lgP	Mass	Length	ppm	m/z	z	RT	Fraction	Scan	Source File
R.NVKEGWYANLGPM(+15.99)R.L	N	71.22	1649.7983	14	0.4	825.9067	2	41.24	1	F1:12368	12112021_RID_2349_DP1.raw
D.PGLLKYPVKPSEEGK.S	N	71.12	1640.9137	15	0.7	547.9789	3	36.73	1	F1:10817	12112021_RID_2349_DP1.raw
K.EGWYANLGPM(+15.99)R.L	N	70.55	1308.5920	11	0.5	655.3036	2	44.48	3	F3:12782	17112021_RID_2349_DP3.raw
K.HDDIFAYEK.R	N	70.19	1136.5138	9	0.4	569.2644	2	36.68	2	F2:9942	12112021_RID_2349_DP2.raw
K.YPVKPSEEGKSAGELYEESLR.K	Y	70.02	2367.1594	21	0.8	790.0610	3	41.55	1	F1:12377	12112021_RID_2349_DP1.raw
L.ELNEFVQETENGWYFIK.N	Y	68.81	2145.0054	17	0.3	1073.5103	2	67.16	2	F2:19907	12112021_RID_2349_DP2.raw
K.SAGELYEESLR.K	Y	68.45	1252.5935	11	-0.9	627.3035	2	38.10	1	F1:11394	12112021_RID_2349_DP1.raw
K.RFDEIVGGM(+15.99)DK.L	N	67.11	1281.6023	11	0.5	641.8087	2	36.31	2	F2:10416	12112021_RID_2349_DP2.raw
K.YPVKPSEEGK.S	N	66.22	1132.5764	10	0.6	567.2958	2	19.01	5	F5:4120	17112021_RID_2349_DP5.raw
K.ESVHFNAR.V	Y	64.04	958.4620	8	-0.8	480.2379	2	23.05	1	F1:6037	12112021_RID_2349_DP1.raw
R.VGEVKKDPGLLK.Y	N	63.88	1281.7656	12	-0.3	428.2624	3	28.13	1	F1:8041	12112021_RID_2349_DP1.raw
L.NEFVQETENGWYFIK.N	N	63.17	1902.8788	15	0.5	952.4471	2	60.53	2	F2:17717	12112021_RID_2349_DP2.raw
D.DYEEFLEIAK.N	N	63.01	1255.5972	10	0.6	628.8062	2	58.64	1	F1:18007	12112021_RID_2349_DP1.raw
R.IKFEPPLPPK.K	N	62.32	1164.6907	10	0.5	583.3529	2	42.88	1	F1:12794	12112021_RID_2349_DP1.raw
D.SGYYVSFIESLK.H	N	61.77	1391.6973	12	0.1	696.8560	2	60.36	3	F3:17967	17112021_RID_2349_DP3.raw
K.EGNLSPGAVDMIGDLLNED.S	N	61.67	1957.8938	19	0.0	979.9542	2	76.89	4	F4:23445	17112021_RID_2349_DP4.raw
R.DVNRASEL	N	60.54	902.4457	8	-0.6	452.2299	2	26.74	1	F1:7420	12112021_RID_2349_DP1.raw
K.VTVLEASERPGGR.V	N	60.35	1369.7313	13	-0.1	457.5843	3	29.25	1	F1:8170	12112021_RID_2349_DP1.raw
K.HDDIFAYEKR.F	N	60.12	1292.6149	10	0.0	431.8789	3	32.95	3	F3:8849	17112021_RID_2349_DP3.raw
R.NVKEGWYANL.G	N	59.21	1192.5876	10	-0.3	597.3009	2	47.98	1	F1:14543	12112021_RID_2349_DP1.raw
K.VTVLEASER.P	N	58.47	1002.5345	9	-0.5	502.2743	2	30.13	5	F5:7870	17112021_RID_2349_DP5.raw
R.IKFEPPLPPKK.E	N	58.07	1292.7855	11	0.1	647.4001	2	39.85	2	F2:10760	12112021_RID_2349_DP2.raw
K.SGLTAARDVNR.A	N	57.18	1158.6105	11	-0.1	387.2107	3	21.39	1	F1:5502	12112021_RID_2349_DP1.raw
Y.QFQHFSEALTAPVGR.I	N	56.85	1686.8478	15	0.7	844.4318	2	42.64	4	F4:12109	17112021_RID_2349_DP4.raw
R.FDEIVGGMDK.L	N	56.21	1109.5062	10	0.9	555.7609	2	39.99	2	F2:11060	12112021_RID_2349_DP2.raw
N.AHGWIDSTIK.S	N	55.64	1126.5771	10	1.1	564.2964	2	35.13	3	F3:9624	17112021_RID_2349_DP3.raw
K.WSLDKYAMGA.I	N	55.33	1140.5273	10	0.5	571.2712	2	50.51	1	F1:15333	12112021_RID_2349_DP1.raw
K.STTDLPSR.F	N	55.31	875.4348	8	0.5	438.7249	2	22.15	1	F1:5769	12112021_RID_2349_DP1.raw
D.YEEFLEIAK.N	N	55.22	1140.5702	9	-0.1	571.2923	2	49.00	1	F1:14874	12112021_RID_2349_DP1.raw
H.FSEALTAPVGR.I	N	55.11	1146.6033	11	0.5	574.3092	2	39.16	5	F5:11001	17112021_RID_2349_DP5.raw
R.IFFAGEYTAN.A	N	54.27	1131.5237	10	0.8	1132.5319	1	55.58	1	F1:16980	12112021_RID_2349_DP1.raw
R.FDEIVGGM(+15.99)DK.L	N	53.88	1125.5012	10	-0.3	563.7577	2	37.17	1	F1:10962	12112021_RID_2349_DP1.raw
K.LPTSM(+15.99)HQDIK.E	Y	53.34	1184.5859	10	0.8	395.8696	3	26.70	2	F2:6476	12112021_RID_2349_DP2.raw
K.EGWYANLGPMRLPEK.H	N	52.68	1759.8716	15	0.9	587.6317	3	52.96	2	F2:15393	12112021_RID_2349_DP2.raw
K.RFDEIVGGMDKLPTSMHQD.I	Y	52.19	2175.0088	19	1.0	726.0109	3	48.28	3	F3:14082	17112021_RID_2349_DP3.raw
N.DLSSIHQLPK.E	N	51.71	1136.6189	10	0.9	569.3173	2	34.51	1	F1:10039	12112021_RID_2349_DP1.raw
K.FEPPLPPKK.E	N	51.31	1051.6066	9	0.2	526.8107	2	31.13	3	F3:8589	17112021_RID_2349_DP3.raw

Peptide	Uniq	-10lgP	Mass	Length	ppm	m/z	z	RT	Fraction	Scan	Source File
K.EGNLSPGAVDM(+15.99)IGDLLNED.S	N	51.30	1973.8888	19	0.5	987.9521	2	67.55	4	F4:20200	17112021_RID_2349_DP4.raw
S.AAYVLAGAGHK.V	N	50.59	1056.5715	11	1.1	529.2936	2	24.54	3	F3:6001	17112021_RID_2349_DP3.raw
D.LLNEDSGYYVSFIESLK.H	N	49.92	1975.9778	17	0.7	988.9969	2	68.81	3	F3:20708	17112021_RID_2349_DP3.raw
K.RFDEIVGGMDKLPTSM(+15.99)HQDIK.E	Y	49.70	2432.1829	21	0.4	609.0532	4	45.01	3	F3:12950	17112021_RID_2349_DP3.raw
K.RFDEIVGGM(+15.99)D.K	N	49.15	1153.5073	10	0.1	577.7610	2	38.54	3	F3:10739	17112021_RID_2349_DP3.raw
R.RIKFEPPLPPK.K	N	49.13	1320.7917	11	0.5	441.2714	3	45.59	2	F2:12721	12112021_RID_2349_DP2.raw
D.DIFAYEKR.F	N	48.19	1040.5291	8	0.1	521.2719	2	35.16	3	F3:9617	17112021_RID_2349_DP3.raw
K.HIVVVGAGM(+15.99)SGLSAAYVLAGAGHK.V	Y	47.83	2280.2048	24	-0.2	761.0754	3	51.51	3	F3:14961	17112021_RID_2349_DP3.raw
K.YAMGAITTFTPY.Q	N	46.88	1334.6217	12	0.9	668.3187	2	64.07	4	F4:19119	17112021_RID_2349_DP4.raw
R.FDEIVGGMDKLPTSM(+15.99)HQDIK.E	Y	46.36	2276.0818	20	0.4	759.7015	3	43.40	4	F4:12371	17112021_RID_2349_DP4.raw
K.EGNLSPGAVDM(+15.99)IGD.L	N	46.09	1389.6082	14	0.2	695.8115	2	46.47	4	F4:13379	17112021_RID_2349_DP4.raw
Y.PVKPSEEGK.S	N	45.74	969.5131	9	-0.5	485.7636	2	19.27	2	F2:3861	12112021_RID_2349_DP2.raw
K.IQQNANEVTVTY.Q	Y	45.71	1378.6729	12	0.8	690.3442	2	41.87	4	F4:11834	17112021_RID_2349_DP4.raw
K.RFDEIVGGMD.K	N	45.54	1137.5125	10	-0.5	569.7632	2	45.56	3	F3:13157	17112021_RID_2349_DP3.raw
K.VTVLEASERPGGRVR.T	N	45.40	1624.9009	15	0.3	542.6411	3	30.01	1	F1:8563	12112021_RID_2349_DP1.raw
Q.HFSEALTAPVGR.I	N	42.43	1283.6622	12	0.5	642.8387	2	34.80	4	F4:9467	17112021_RID_2349_DP4.raw
R.IVREYIK.K	N	41.49	919.5491	7	0.1	460.7819	2	25.34	1	F1:6965	12112021_RID_2349_DP1.raw
K.YAMGAITTFTPYQF.Q	N	41.39	1609.7487	14	-0.2	805.8814	2	71.88	3	F3:21650	17112021_RID_2349_DP3.raw
K.RFDEIVGGMDKLPTSMH.Q	Y	41.32	1931.9233	17	0.7	644.9822	3	48.99	3	F3:14232	17112021_RID_2349_DP3.raw
K.EGWYANLGPM(+15.99)RLPEK.H	N	40.61	1775.8665	15	0.9	592.9633	3	45.16	3	F3:12960	17112021_RID_2349_DP3.raw
L.SAAYVLAGAGHK.V	N	40.21	1143.6036	12	0.7	572.8095	2	26.75	5	F5:6718	17112021_RID_2349_DP5.raw
K.RFDEIVGGM(+15.99)DKLPTSMHQDIK.E	Y	40.16	2432.1829	21	1.5	811.7361	3	42.94	3	F3:12300	17112021_RID_2349_DP3.raw
F.SEALTAPVGR.I	N	40.05	999.5349	10	-0.1	500.7746	2	30.20	3	F3:7936	17112021_RID_2349_DP3.raw
D.DIFAYEK.R	N	39.75	884.4279	7	0.8	885.4359	1	41.87	4	F4:11928	17112021_RID_2349_DP4.raw
K.RVGEVKKDPGLLK.Y	N	39.62	1437.8667	13	1.0	480.2967	3	39.89	1	F1:11650	12112021_RID_2349_DP1.raw
R.FDEIVGGM(+15.99)DKLPTSMHQDIK.E	Y	38.92	2276.0818	20	0.9	759.7019	3	41.79	3	F3:11943	17112021_RID_2349_DP3.raw
K.WSLDKYAMGAITTF.T	N	38.72	1602.7751	14	1.3	802.3959	2	67.34	3	F3:20215	17112021_RID_2349_DP3.raw

TRINITY_DN233
back to list

| Protein Coverage | Supporting Peptides |
Protein Coverage:

```
1   MAPVSYLLQA WILLSLHGCI TSYPKQARSA SEDIRNCTTN PQYLPPTVVV TGERLKTLRE HMKTHRLSAY IVPNTDAHLS
81  EYVAERDMRL SWMTGFSGSE GTGVITLQKA ALFTDSRYWI QAERQMDCNW ELQKSAWINF IGQWILNEVP AGGTIGLDPF
161 LFSVDTWYNY HQVLEGANRT LDLLEVNLVD LVWGSERLPP PTNTIYRLAD DFMGGTWQEK VANARGQMEE HSKKPTAILL
241 SGLEETAWLF NLRGDDIPYT PVFYAYTLLT KTDISLFTNR SRLSKEALQM LTAGCPESPC VKVEDYDQIG ASLRKYAHQD
321 GVIVWIGTEY TTLGLYKEIP QENLLEDDFS PVMLSKAVKN SKEQKMMEAA HIRDAVALIR YLVWLEKNVP KNSVDELSGA
401 NYLDTLRSEE LHCKGPSFQT ISASGLNAAL AHYSPSSLTS RKLSLNEMYL LDSGGQYFDG TTDITRTIHW GEPTAFQKEA
481 YTRVLMGNID LSKLVFPPRM SGRLVESFAR RPLWEAGLNY GHGTGHGIGN FLSVHEWPVG FQSNNVPLDK GMFTSIEPGY
561 YHDGEFGIRI EDVALVVPAK TKYPVQEDPY LTFKVVSLVP YARNLINESL LSQDQIQYIN RYYETIRQVI GPELQRRQLE
641 EEYRWLEKNT EPFSHGSVLA ASLGTLAVST LASGLLPAAQ H
```

Supporting Peptides:

Peptide	Uniq	-10lgP	Mass	Length	ppm	m/z	z	RT	Fraction	Scan	Source File	Area F
R.NLINESLLSQDQIQYINR.Y	Y	102.57	2160.1174	18	0.2	1081.0662	2	57.94	2	F2:16943	12112021_RID_2349_DP2.raw	1.6872E
R.LADDFMGGTWQEKVANAR.G	Y	97.16	2007.9473	18	0.4	1004.9813	2	49.13	1	F1:14896	12112021_RID_2349_DP1.raw	1.1424E
K.NSVDELSGANYLDTLR.S	Y	96.75	1765.8483	16	0.7	883.9320	2	53.81	2	F2:15577	12112021_RID_2349_DP2.raw	1.6193E
K.EIPQENLLEDDFSPVMLSK.A	N	94.92	2203.0718	19	0.2	1102.5433	2	65.59	1	F1:20182	12112021_RID_2349_DP1.raw	4.4307E
K.TKYPVQEDPYLTFK.V	Y	84.98	1727.8770	14	0.9	576.9668	3	46.95	1	F1:14170	12112021_RID_2349_DP1.raw	2.0786E
K.VEDYDQIGASLR.K	Y	81.88	1364.6572	12	0.7	683.3364	2	42.99	2	F2:12000	12112021_RID_2349_DP2.raw	2.0691E
R.TIHWGEPTAFQK.E	N	81.74	1413.7041	12	0.9	707.8600	2	42.18	1	F1:12610	12112021_RID_2349_DP1.raw	4.9198E
R.LADDFM(+15.99)GGTWQEKVANAR.G	Y	80.31	2023.9421	18	0.5	675.6550	3	43.18	1	F1:12956	12112021_RID_2349_DP1.raw	6.9069E
R.IEDVALVVPAK.T	Y	80.13	1152.6754	11	-0.4	577.3447	2	45.25	1	F1:13594	12112021_RID_2349_DP1.raw	2.7331E
R.LADDFM(+15.99)GGTWQEK.V	Y	79.06	1512.6555	13	0.1	757.3351	2	41.88	1	F1:12596	12112021_RID_2349_DP1.raw	9.9489E
R.LADDFMGGTWQEK.V	Y	75.54	1496.6605	13	1.0	749.3383	2	49.98	1	F1:15195	12112021_RID_2349_DP1.raw	1.722E7
R.VLMGNIDLSK.L	N	74.32	1088.5900	10	0.9	545.3027	2	44.91	1	F1:13547	12112021_RID_2349_DP1.raw	7.3687E
R.YLVWLEKNVPK.N	N	74.01	1387.7864	11	0.1	694.9005	2	48.76	1	F1:14779	12112021_RID_2349_DP1.raw	3.2724E
K.EIPQENLLEDDFSPVM(+15.99)LSK.A	N	72.70	2219.0667	19	-0.3	1110.5403	2	61.58	2	F2:18110	12112021_RID_2349_DP2.raw	1.502E6
K.VEDYDQIGASLRK.Y	Y	72.65	1492.7521	13	1.0	747.3841	2	37.40	1	F1:11075	12112021_RID_2349_DP1.raw	2.5025E
K.YPVQEDPYLTFK.V	Y	72.14	1498.7344	12	0.1	750.3745	2	49.80	4	F4:14509	17112021_RID_2349_DP4.raw	1.1678E
K.AALFTDSR.Y	Y	69.22	879.4450	8	-0.4	440.7296	2	32.38	2	F2:8405	12112021_RID_2349_DP2.raw	3.6699E
R.VLM(+15.99)GNIDLSK.L	N	68.66	1104.5848	10	0.1	553.2997	2	44.91	1	F1:13558	12112021_RID_2349_DP1.raw	5.0787E
K.VVSLVPYAR.N	N	63.21	1002.5862	9	0.4	502.3006	2	43.57	1	F1:13098	12112021_RID_2349_DP1.raw	3.175E7
K.MMEAAHIR.D	N	61.26	957.4525	8	0.5	479.7338	2	24.46	2	F2:5605	12112021_RID_2349_DP2.raw	2.5335E
R.LPPPTNTIYR.L	N	61.17	1170.6396	10	0.3	586.3273	2	35.87	1	F1:10436	12112021_RID_2349_DP1.raw	2.508E7
K.NTEPFSHGSVLA.A	Y	60.30	1257.5989	12	0.5	629.8070	2	40.01	1	F1:11896	12112021_RID_2349_DP1.raw	8.7833E

Peptide	Uniq	-10lgP	Mass	Length	ppm	m/z	z	RT	Fraction	Scan	Source File	Area F
R.LSAYIVPNTDAHLSEYVAER.D	Y	60.08	2247.1172	20	0.6	750.0468	3	50.55	2	F2:14530	12112021_RID_2349_DP2.raw	1.5595E
D.ELSGANYLDTLR.S	Y	59.09	1350.6779	12	0.5	676.3466	2	48.45	4	F4:14024	17112021_RID_2349_DP4.raw	
R.RQLEEEYR.W	Y	56.64	1121.5465	8	0.2	561.7806	2	21.08	3	F3:4733	17112021_RID_2349_DP3.raw	
K.MM(+15.99)EAAHIR.D	N	56.35	973.4473	8	-0.3	487.7308	2	24.42	2	F2:5548	12112021_RID_2349_DP2.raw	
K.M(+15.99)MEAAHIR.D	N	55.53	973.4473	8	-0.6	487.7307	2	22.62	2	F2:5124	12112021_RID_2349_DP2.raw	
R.MSGRLVESFAR.R	Y	55.47	1251.6394	11	0.0	418.2204	3	38.02	1	F1:11242	12112021_RID_2349_DP1.raw	1.6579E
D.SGGQYFDGTTDITR.T	N	55.24	1516.6793	14	0.9	759.3477	2	39.61	4	F4:11084	17112021_RID_2349_DP4.raw	
R.QVIGPELQR.R	Y	54.67	1038.5822	9	-0.4	520.2982	2	34.14	1	F1:10002	12112021_RID_2349_DP1.raw	2.1413E
R.LSWMTGFSGSEGTGVITLQK.A	Y	53.91	2098.0405	20	0.2	1050.0277	2	62.94	2	F2:18422	12112021_RID_2349_DP2.raw	2.1277E
R.YWIQAER.Q	N	52.15	964.4766	7	-0.4	483.2454	2	36.97	2	F2:9988	12112021_RID_2349_DP2.raw	1.3881E
R.GDDIPYTPVFYAYTLLTK.T	N	51.60	2076.0454	18	0.2	1039.0302	2	78.46	1	F1:24532	12112021_RID_2349_DP1.raw	3.0975E
K.GPSFQTISASGLNAALAHYSPSSLTSR.K	Y	50.90	2719.3564	27	0.3	907.4597	3	60.52	2	F2:17711	12112021_RID_2349_DP2.raw	
K.NVPKNSVDELSGANYLDTLR.S	Y	50.02	2204.1072	20	0.7	1103.0616	2	50.58	1	F1:15379	12112021_RID_2349_DP1.raw	2.6389E
K.GMFTSIEPGYYHDGEFGIR.I	N	48.84	2174.9731	19	0.7	725.9988	3	56.28	2	F2:16416	12112021_RID_2349_DP2.raw	
K.GM(+15.99)FTSIEPGYYHDGEFGIR.I	N	46.73	2190.9680	19	0.3	731.3302	3	51.35	2	F2:14719	12112021_RID_2349_DP2.raw	
R.TIHWGEPTAFQKEAYTR.V	N	46.04	2033.9958	17	0.9	679.0065	3	45.28	1	F1:13603	12112021_RID_2349_DP1.raw	2.9881E
R.QVIGPELQRR.Q	Y	45.98	1194.6832	10	0.8	598.3494	2	28.93	1	F1:8159	12112021_RID_2349_DP1.raw	4.0244E
R.LVESFAR.R	Y	45.21	820.4443	7	-0.1	411.2294	2	31.00	4	F4:8159	17112021_RID_2349_DP4.raw	2.2969E
R.YLVWLEK.N	N	43.98	949.5273	7	-0.5	475.7707	2	51.37	1	F1:15594	12112021_RID_2349_DP1.raw	3.0018E
K.NSVDELSGANYLDTLRSEE.L	Y	41.99	2110.9653	19	0.7	1056.4907	2	55.67	1	F1:17087	12112021_RID_2349_DP1.raw	7.1446E
K.TKYPVQED.P	Y	39.43	978.4658	8	0.6	490.2405	2	23.69	1	F1:6389	12112021_RID_2349_DP1.raw	1.8089E
R.QLEEEYR.W	Y	39.22	965.4454	7	-0.3	483.7298	2	24.68	2	F2:5729	12112021_RID_2349_DP2.raw	

AHJ80886.1
back to list

| Protein Coverage | Supporting Peptides |
Protein Coverage:

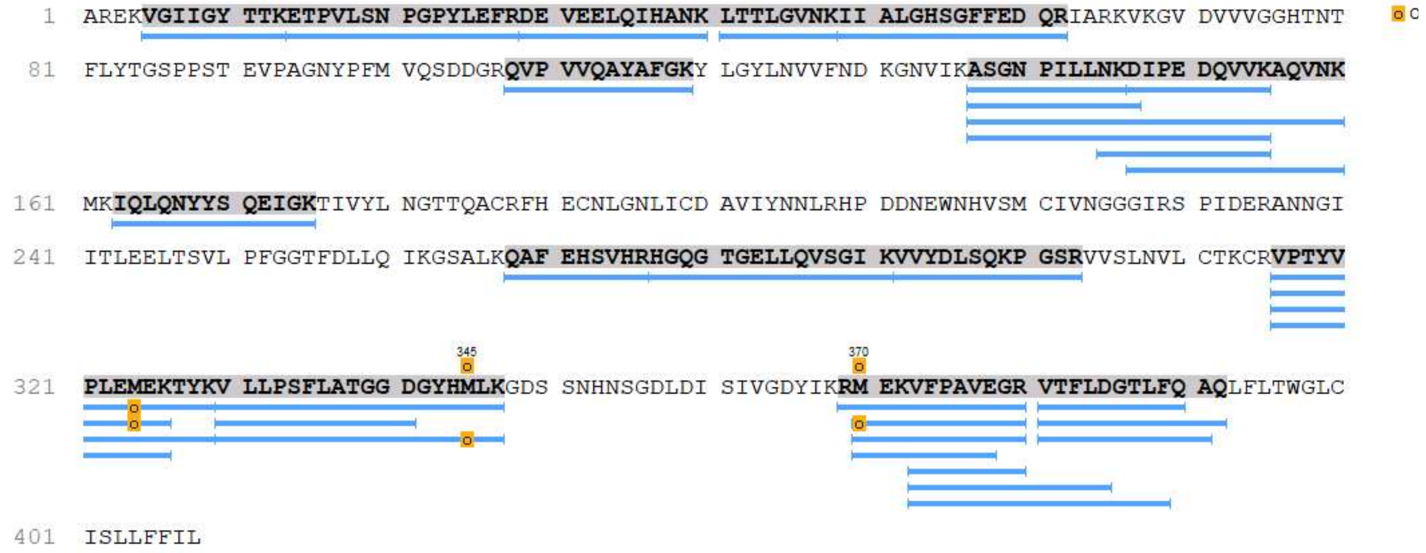

Supporting Peptides:

Peptide	Uniq	-10lgP	Mass	Length	ppm	m/z	z	RT	Fraction	Scan	Source File	Area F1

Peptide	Uniq	-10lgP	Mass	Length	ppm	m/z	z	RT	Fraction	Scan	Source File	Area F1
R.HGQGTGELLQVSGIK.V	N	95.91	1522.8103	15	0.4	762.4127	2	39.99	3	F3:11299	17112021_RID_2349_DP3.raw	2.7833E6
K.IQLQNYYSQEIGK.T	Y	93.83	1582.7991	13	0.7	792.4073	2	45.41	1	F1:13679	12112021_RID_2349_DP1.raw	8.8534E6
K.IIALGHSGFFEDQR.I	N	93.42	1588.7997	14	0.8	795.4078	2	47.13	2	F2:13392	12112021_RID_2349_DP2.raw	1.2282E6
K.VLLPSFLATGGDGYHMLK.G	Y	87.27	1918.0022	18	0.7	960.0090	2	60.01	3	F3:17866	17112021_RID_2349_DP3.raw	
R.VPTYVPLEMEKTYK.V	N	87.11	1696.8745	14	0.4	566.6323	3	45.65	1	F1:13767	12112021_RID_2349_DP1.raw	5.018E6
K.ASGNPILLNKDIPEDQVVKAQVNK.M	Y	87.11	2589.4126	24	1.2	864.1459	3	45.05	1	F1:13591	12112021_RID_2349_DP1.raw	9.398E6
K.ASGNPILLNKDIPEDQVVK.A	Y	83.55	2049.1106	19	0.8	684.0447	3	45.02	1	F1:13569	12112021_RID_2349_DP1.raw	3.5624E7
R.QVPVVQAYAFGK.Y	N	78.42	1305.7081	12	0.2	653.8615	2	46.67	2	F2:13272	12112021_RID_2349_DP2.raw	4.2275E6
K.DIPEDQVVKAQVNK.M	Y	77.75	1581.8362	14	1.0	791.9261	2	34.51	1	F1:10105	12112021_RID_2349_DP1.raw	1.479E6
K.QAFEHSVHR.H	N	75.67	1109.5366	9	0.7	555.7760	2	21.01	1	F1:5462	12112021_RID_2349_DP1.raw	1.4117E6
R.VPTYVPLEMEK.T	N	71.68	1304.6686	11	0.5	653.3419	2	47.31	2	F2:13493	12112021_RID_2349_DP2.raw	8.3497E6
R.MEKVFPAVEGR.V	N	70.69	1261.6488	11	0.2	421.5570	3	35.91	1	F1:10572	12112021_RID_2349_DP1.raw	6.3631E6
R.DEVEELQIHANK.L	N	70.10	1423.6943	12	0.6	712.8549	2	34.19	4	F4:9279	17112021_RID_2349_DP4.raw	
K.ASGNPILLNK.D	N	67.11	1025.5869	10	-0.1	513.8007	2	34.04	1	F1:9894	12112021_RID_2349_DP1.raw	2.3772E7
R.VTFLDGTLFQA.Q	Y	67.07	1210.6234	11	0.9	606.3195	2	72.56	1	F1:22584	12112021_RID_2349_DP1.raw	1.349E5
R.VPTYVPLEM(+15.99)EK.T	N	64.43	1320.6635	11	0.7	661.3395	2	41.27	1	F1:12376	12112021_RID_2349_DP1.raw	5.2823E6
K.ETPVLSNPGPYLEFR.D	N	63.99	1717.8674	15	0.3	859.9412	2	55.85	4	F4:16529	17112021_RID_2349_DP4.raw	
K.VGIIGYTTK.E	N	60.94	950.5436	9	-0.2	476.2790	2	37.93	1	F1:11149	12112021_RID_2349_DP1.raw	9.0552E6
K.RMEKVFPAVEGR.V	N	60.62	1417.7500	12	-0.5	473.5904	3	38.18	1	F1:11332	12112021_RID_2349_DP1.raw	1.0596E6
R.M(+15.99)EKVFPAVEGR.V	N	56.35	1277.6438	11	-0.1	426.8885	3	34.90	1	F1:10583	12112021_RID_2349_DP1.raw	4.4201E6
K.VFPAVEGR.V	N	54.60	873.4708	8	0.2	437.7428	2	33.73	1	F1:9818	12112021_RID_2349_DP1.raw	2.3327E7
R.VPTYVPLEM(+15.99)EKTYK.V	N	53.59	1712.8695	14	-0.2	571.9637	3	45.68	1	F1:13801	12112021_RID_2349_DP1.raw	1.6172E6
K.VVYDLSQKPGSR.V	N	53.26	1347.7146	12	0.8	674.8651	2	28.72	7	F7:7430	17112021_RID_2349_DP7.raw	
K.ASGNPILLNKD.I	N	52.23	1140.6139	11	0.6	571.3146	2	34.93	4	F4:9472	17112021_RID_2349_DP4.raw	
R.VTFLDGTLFQ.A	Y	52.01	1139.5863	10	0.1	570.8005	2	71.52	1	F1:22214	12112021_RID_2349_DP1.raw	1.731E6
K.VFPAVEGRVTFLD.G	Y	50.74	1448.7664	13	0.1	725.3905	2	58.64	1	F1:18050	12112021_RID_2349_DP1.raw	2.5813E5
K.VFPAVEGRVTFLDGTLF.Q	Y	49.96	1866.9879	17	1.5	934.5026	2	72.35	1	F1:22539	12112021_RID_2349_DP1.raw	6.8832E5
K.LTTLGVNK.I	N	48.37	844.5018	8	-0.2	423.2581	2	28.18	2	F2:6923	12112021_RID_2349_DP2.raw	1.9323E6
R.VTFLDGTLFQAQ.L	Y	48.28	1338.6819	12	1.0	670.3489	2	70.39	1	F1:21836	12112021_RID_2349_DP1.raw	2.3504E5
K.DIPEDQVVK.A	Y	47.98	1041.5342	9	0.0	521.7744	2	33.23	1	F1:9696	12112021_RID_2349_DP1.raw	6.0307E6
K.VLLPSFLATGGDGYHM(+15.99)LK.G	Y	47.16	1933.9971	18	1.6	968.0073	2	55.51	4	F4:16340	17112021_RID_2349_DP4.raw	
L.NKDIPEDQVVK.A	Y	45.22	1283.6721	11	0.5	642.8436	2	26.35	1	F1:7247	12112021_RID_2349_DP1.raw	5.8334E4
K.VLLPSFLATGGD.G	N	44.90	1188.6390	12	0.8	1189.6472	1	70.35	1	F1:21823	12112021_RID_2349_DP1.raw	1.8966E5
R.MEKVFPAVE.G	N	39.13	1048.5262	9	-0.6	525.2701	2	42.65	1	F1:12815	12112021_RID_2349_DP1.raw	2.9669E5

G8XQX1.1
back to list

| Protein Coverage | Supporting Peptides |
Protein Coverage:

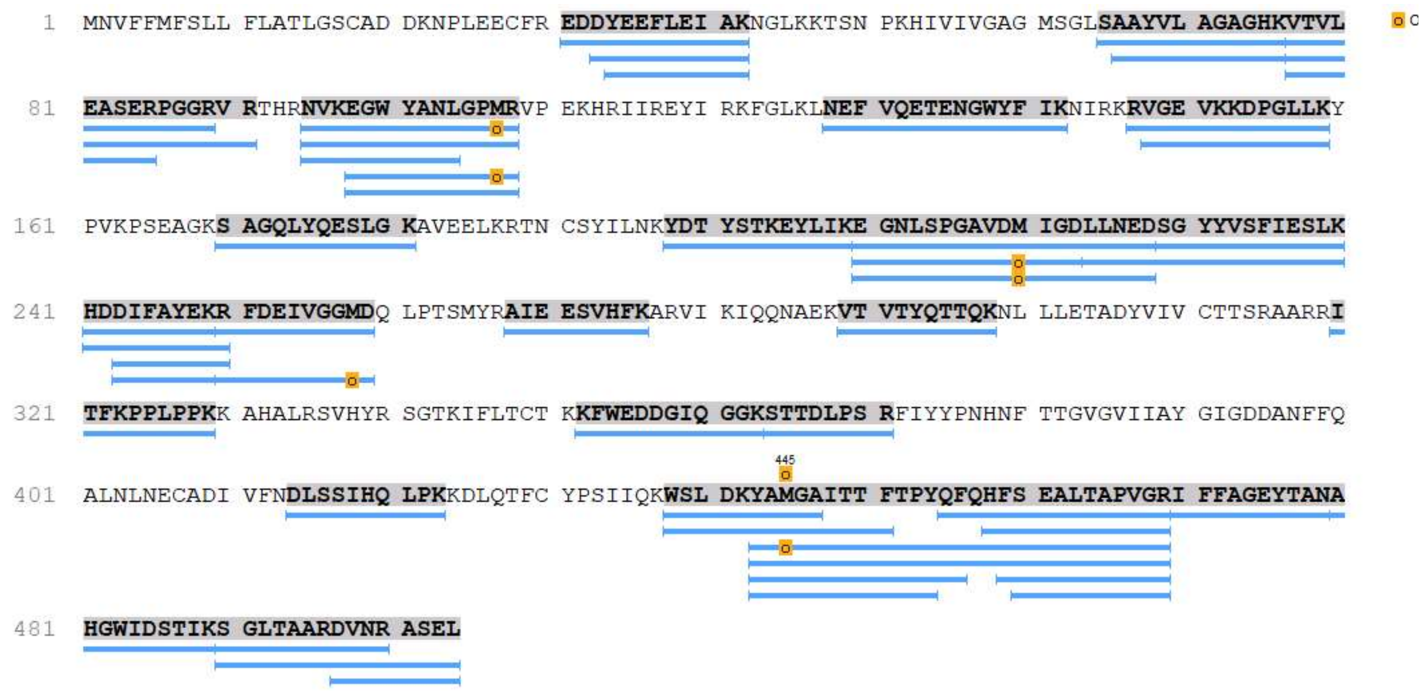

Supporting Peptides:

Peptide	Uniq	-10lgP	Mass	Length	ppm	m/z	z	RT	Fraction	Scan	Source File
K.YAMGAITTFTPYQFQHFSEALTAPVGR.I	N	93.35	3003.4587	27	0.8	1002.1609	3	68.16	3	F3:20530	17112021_RID_2349_DP3.raw
R.NVKEGWYANLGPMR.V	N	89.97	1633.8035	14	1.1	817.9099	2	46.58	1	F1:14171	12112021_RID_2349_DP1.raw
K.SGLTAARDVNRASEL	N	81.95	1558.8063	15	-0.1	520.6093	3	35.16	1	F1:10323	12112021_RID_2349_DP1.raw
K.YDTYSTKEYLIK.E	N	80.80	1522.7555	12	0.9	762.3857	2	38.58	2	F2:10545	12112021_RID_2349_DP2.raw
K.YAM(+15.99)GAITTFTPYQFQHFSEALTAPVGR.I	N	80.23	3019.4539	27	0.9	1007.4928	3	64.54	3	F3:19257	17112021_RID_2349_DP3.raw
K.SAGQLYQESLGK.A	N	80.13	1279.6407	12	0.1	640.8277	2	34.93	1	F1:10235	12112021_RID_2349_DP1.raw
K.EGWYANLGPMR.V	N	74.35	1292.5972	11	0.2	647.3060	2	50.70	1	F1:15490	12112021_RID_2349_DP1.raw
R.EDDYEEFLEIAK.N	N	74.11	1499.6667	12	0.7	750.8412	2	59.88	5	F5:17908	17112021_RID_2349_DP5.raw
R.NVKEGWYANLGPM(+15.99)R.V	N	71.22	1649.7983	14	0.4	825.9067	2	41.24	1	F1:12368	12112021_RID_2349_DP1.raw
K.EGWYANLGPM(+15.99)R.V	N	70.55	1308.5920	11	0.5	655.3036	2	44.48	3	F3:12782	17112021_RID_2349_DP3.raw
K.HDDIFAYEK.R	N	70.19	1136.5138	9	0.4	569.2644	2	36.68	2	F2:9942	12112021_RID_2349_DP2.raw
R.VGEVKKDPGLLK.Y	N	63.88	1281.7656	12	-0.3	428.2624	3	28.13	1	F1:8041	12112021_RID_2349_DP1.raw
L.NEFVQETENGWYFIK.N	N	63.17	1902.8788	15	0.5	952.4471	2	60.53	2	F2:17717	12112021_RID_2349_DP2.raw
D.DYEEFLEIAK.N	N	63.01	1255.5972	10	0.6	628.8062	2	58.64	1	F1:18007	12112021_RID_2349_DP1.raw
D.SGYYVSFIESLK.H	N	61.77	1391.6973	12	0.1	696.8560	2	60.36	3	F3:17967	17112021_RID_2349_DP3.raw
K.EGNLSPGAVDMIGDLLNED.S	N	61.67	1957.8938	19	0.0	979.9542	2	76.89	4	F4:23445	17112021_RID_2349_DP4.raw
R.DVNRASEL	N	60.54	902.4457	8	-0.6	452.2299	2	26.74	1	F1:7420	12112021_RID_2349_DP1.raw
K.VTVLEASERPGGR.V	N	60.35	1369.7313	13	-0.1	457.5843	3	29.25	1	F1:8170	12112021_RID_2349_DP1.raw
K.HDDIFAYEKR.F	N	60.12	1292.6149	10	0.0	431.8789	3	32.95	3	F3:8849	17112021_RID_2349_DP3.raw
R.NVKEGWYANL.G	N	59.21	1192.5876	10	-0.3	597.3009	2	47.98	1	F1:14543	12112021_RID_2349_DP1.raw
K.VTVLEASER.P	N	58.47	1002.5345	9	-0.5	502.2743	2	30.13	5	F5:7870	17112021_RID_2349_DP5.raw
R.AIEESVHFK.A	Y	58.30	1058.5397	9	-0.1	530.2770	2	29.87	7	F7:7843	17112021_RID_2349_DP7.raw
K.VTVTYQTTQK.N	N	57.89	1167.6135	10	0.3	584.8142	2	25.60	7	F7:6331	17112021_RID_2349_DP7.raw
K.SGLTAARDVNR.A	N	57.18	1158.6105	11	-0.1	387.2107	3	21.39	1	F1:5502	12112021_RID_2349_DP1.raw

Peptide	Uniq	-10lgP	Mass	Length	ppm	m/z	z	RT	Fraction	Scan	Source File
Y.QFQHFSEALTAPVGR.I	N	56.85	1686.8478	15	0.7	844.4318	2	42.64	4	F4:12109	17112021_RID_2349_DP4.raw
N.AHGWIDSTIK.S	N	55.64	1126.5771	10	1.1	564.2964	2	35.13	3	F3:9624	17112021_RID_2349_DP3.raw
K.WSLDKYAMGA.I	N	55.33	1140.5273	10	0.5	571.2712	2	50.51	1	F1:15333	12112021_RID_2349_DP1.raw
K.STTDLPSR.F	N	55.31	875.4348	8	0.5	438.7249	2	22.15	1	F1:5769	12112021_RID_2349_DP1.raw
D.YEEFLEIAK.N	N	55.22	1140.5702	9	-0.1	571.2923	2	49.00	1	F1:14874	12112021_RID_2349_DP1.raw
H.FSEALTAPVGR.I	N	55.11	1146.6033	11	0.5	574.3092	2	39.16	5	F5:11001	17112021_RID_2349_DP5.raw
R.IFFAGEYTAN.A	N	54.27	1131.5237	10	0.8	1132.5319	1	55.58	1	F1:16980	12112021_RID_2349_DP1.raw
R.ITFKPPLPPK.K	N	54.10	1136.6957	10	0.3	379.9059	3	40.53	7	F7:11518	17112021_RID_2349_DP7.raw
N.DLSSIHQLPK.K	N	51.71	1136.6189	10	0.9	569.3173	2	34.51	1	F1:10039	12112021_RID_2349_DP1.raw
K.EGNLSPGAVDM(+15.99)IGDLLNED.S	N	51.30	1973.8888	19	0.5	987.9521	2	67.55	4	F4:20200	17112021_RID_2349_DP4.raw
S.AAYVLAGAGHK.V	N	50.59	1056.5715	11	1.1	529.2936	2	24.54	3	F3:6001	17112021_RID_2349_DP3.raw
D.LLNEDSGYYVSFIESLK.H	N	49.92	1975.9778	17	0.7	988.9969	2	68.81	3	F3:20708	17112021_RID_2349_DP3.raw
K.RFDEIVGGM(+15.99)D.Q	N	49.15	1153.5073	10	0.1	577.7610	2	38.54	3	F3:10739	17112021_RID_2349_DP3.raw
D.DIFAYEKR.F	N	48.19	1040.5291	8	0.1	521.2719	2	35.16	3	F3:9617	17112021_RID_2349_DP3.raw
K.YAMGAITTFTPY.Q	N	46.88	1334.6217	12	0.9	668.3187	2	64.07	4	F4:19119	17112021_RID_2349_DP4.raw
K.EGNLSPGAVDM(+15.99)IGD.L	N	46.09	1389.6082	14	0.2	695.8115	2	46.47	4	F4:13379	17112021_RID_2349_DP4.raw
K.RFDEIVGGMD.Q	N	45.54	1137.5125	10	-0.5	569.7632	2	45.56	3	F3:13157	17112021_RID_2349_DP3.raw
K.VTVLEASERPGGRVR.T	N	45.40	1624.9009	15	0.3	542.6411	3	30.01	1	F1:8563	12112021_RID_2349_DP1.raw
K.KFWEDDGIQGGK.S	N	44.74	1378.6517	12	-2.0	690.3318	2	35.62	7	F7:9823	17112021_RID_2349_DP7.raw
Q.HFSEALTAPVGR.I	N	42.43	1283.6622	12	0.5	642.8387	2	34.80	4	F4:9467	17112021_RID_2349_DP4.raw
K.YAMGAITTFTPYQF.Q	N	41.39	1609.7487	14	-0.2	805.8814	2	71.88	3	F3:21650	17112021_RID_2349_DP3.raw
L.SAAYVLAGAGHK.V	N	40.21	1143.6036	12	0.7	572.8095	2	26.75	5	F5:6718	17112021_RID_2349_DP5.raw
F.SEALTAPVGR.I	N	40.05	999.5349	10	-0.1	500.7746	2	30.20	3	F3:7936	17112021_RID_2349_DP3.raw
D.DIFAYEK.R	N	39.75	884.4279	7	0.8	885.4359	1	41.87	4	F4:11928	17112021_RID_2349_DP4.raw
K.RVGEVKKDPGLLK.Y	N	39.62	1437.8667	13	1.0	480.2967	3	39.89	1	F1:11650	12112021_RID_2349_DP1.raw
K.WSLDKYAMGAITTF.T	N	38.72	1602.7751	14	1.3	802.3959	2	67.34	3	F3:20215	17112021_RID_2349_DP3.raw

ACF70483.1
back to list

| Protein Coverage | Supporting Peptides |
Protein Coverage:

1 MNVFFMFSLL FLATLGSCAD DKNPLEECFR EDDYEEFLEI AKNGLKKTSN PKHIVIVGAG MSGLSAAYVL AGAGHKVTVL

81 EASERPGGRV RTHRNVKEGW YANLGPMRVP EKHRIIREYI RKFGLKLNEF VQETENGWYF IKNIRKRVGE VKKDPGLLKY

161 PVKPSEAGKS AGQLYQESLG KAVEELKRTN CSYILNKYDT YSTKEYLIKE GNLSPGAVDM IGDLLNEDSG YYVSFIESLK

241 HDDIFAYEKR FDEIVGGMDQ LPTSMYRAIE ESVHFKARVI KIQQNAEKVT VTYQTTQKNL LLETADYVIV CTTSRAARRI

321 TFKPPLPPKK AHALRSVHYR SGTKIFLTCT KKFWEDDGIQ GGKSTTDLPS RFIYYPNHNF TTGVGVIIAY GIGDDANFFQ

401 ALNLNECADI VFNDLSSIHQ LPKKDLQTFC YPSIIQKWSL DKYAMGAITT FTPYQFQHFS EALTAPVGRI FFAGEYTANA

481 HGWIDSTIKS GLTAARDVNR ASEL

Supporting Peptides:

Peptide	Uniq	-10lgP	Mass	Length	ppm	m/z	z	RT	Fraction	Scan	Source File
K.YAMGAITTFTPYQFQHFSEALTAPVGR.I	N	93.35	3003.4587	27	0.8	1002.1609	3	68.16	3	F3:20530	17112021_RID_2349_DP3.raw
R.NVKEGWYANLGPMR.V	N	89.97	1633.8035	14	1.1	817.9099	2	46.58	1	F1:14171	12112021_RID_2349_DP1.raw
K.SGLTAARDVNRASEL	N	81.95	1558.8063	15	-0.1	520.6093	3	35.16	1	F1:10323	12112021_RID_2349_DP1.raw
K.YDTYSTKEYLIK.E	N	80.80	1522.7555	12	0.9	762.3857	2	38.58	2	F2:10545	12112021_RID_2349_DP2.raw
K.YAM(+15.99)GAITTFTPYQFQHFSEALTAPVGR.I	N	80.23	3019.4539	27	0.9	1007.4928	3	64.54	3	F3:19257	17112021_RID_2349_DP3.raw
K.SAGQLYQESLGK.A	N	80.13	1279.6407	12	0.1	640.8277	2	34.93	1	F1:10235	12112021_RID_2349_DP1.raw
K.EGWYANLGPMR.V	N	74.35	1292.5972	11	0.2	647.3060	2	50.70	1	F1:15490	12112021_RID_2349_DP1.raw
R.EDDYEEFLEIAK.N	N	74.11	1499.6667	12	0.7	750.8412	2	59.88	5	F5:17908	17112021_RID_2349_DP5.raw
R.NVKEGWYANLGPM(+15.99)R.V	N	71.22	1649.7983	14	0.4	825.9067	2	41.24	1	F1:12368	12112021_RID_2349_DP1.raw
K.EGWYANLGPM(+15.99)R.V	N	70.55	1308.5920	11	0.5	655.3036	2	44.48	3	F3:12782	17112021_RID_2349_DP3.raw
K.HDDIFAYEK.R	N	70.19	1136.5138	9	0.4	569.2644	2	36.68	2	F2:9942	12112021_RID_2349_DP2.raw
R.VGEVKKDPGLLK.Y	N	63.88	1281.7656	12	-0.3	428.2624	3	28.13	1	F1:8041	12112021_RID_2349_DP1.raw
L.NEFVQETENGWYFIK.N	N	63.17	1902.8788	15	0.5	952.4471	2	60.53	2	F2:17717	12112021_RID_2349_DP2.raw
D.DYEEFLEIAK.N	N	63.01	1255.5972	10	0.6	628.8062	2	58.64	1	F1:18007	12112021_RID_2349_DP1.raw
D.SGYYVSFIESLK.H	N	61.77	1391.6973	12	0.1	696.8560	2	60.36	3	F3:17967	17112021_RID_2349_DP3.raw
K.EGNLSPGAVDMIGDLLNED.S	N	61.67	1957.8938	19	0.0	979.9542	2	76.89	4	F4:23445	17112021_RID_2349_DP4.raw
R.DVNRASEL	N	60.54	902.4457	8	-0.6	452.2299	2	26.74	1	F1:7420	12112021_RID_2349_DP1.raw
K.VTVLEASERPGGR.V	N	60.35	1369.7313	13	-0.1	457.5843	3	29.25	1	F1:8170	12112021_RID_2349_DP1.raw
K.HDDIFAYEKR.F	N	60.12	1292.6149	10	0.0	431.8789	3	32.95	3	F3:8849	17112021_RID_2349_DP3.raw
R.NVKEGWYANL.G	N	59.21	1192.5876	10	-0.3	597.3009	2	47.98	1	F1:14543	12112021_RID_2349_DP1.raw
K.VTVLEASER.P	N	58.47	1002.5345	9	-0.5	502.2743	2	30.13	5	F5:7870	17112021_RID_2349_DP5.raw
R.AIEESVHFK.A	Y	58.30	1058.5397	9	-0.1	530.2770	2	29.87	7	F7:7843	17112021_RID_2349_DP7.raw
K.VTVTYQTTQK.N	N	57.89	1167.6135	10	0.3	584.8142	2	25.60	7	F7:6331	17112021_RID_2349_DP7.raw
K.SGLTAARDVNR.A	N	57.18	1158.6105	11	-0.1	387.2107	3	21.39	1	F1:5502	12112021_RID_2349_DP1.raw

Peptide	Uniq	-10lgP	Mass	Length	ppm	m/z	z	RT	Fraction	Scan	Source File
Y.QFQHFSEALTAPVGR.I	N	56.85	1686.8478	15	0.7	844.4318	2	42.64	4	F4:12109	17112021_RID_2349_DP4.raw
N.AHGWIDSTIK.S	N	55.64	1126.5771	10	1.1	564.2964	2	35.13	3	F3:9624	17112021_RID_2349_DP3.raw
K.WSLDKYAMGA.I	N	55.33	1140.5273	10	0.5	571.2712	2	50.51	1	F1:15333	12112021_RID_2349_DP1.raw
K.STTDLPSR.F	N	55.31	875.4348	8	0.5	438.7249	2	22.15	1	F1:5769	12112021_RID_2349_DP1.raw
D.YEEFLEIAK.N	N	55.22	1140.5702	9	-0.1	571.2923	2	49.00	1	F1:14874	12112021_RID_2349_DP1.raw
H.FSEALTAPVGR.I	N	55.11	1146.6033	11	0.5	574.3092	2	39.16	5	F5:11001	17112021_RID_2349_DP5.raw
R.IFFAGEYTAN.A	N	54.27	1131.5237	10	0.8	1132.5319	1	55.58	1	F1:16980	12112021_RID_2349_DP1.raw
R.ITFKPPLPPK.K	N	54.10	1136.6957	10	0.3	379.9059	3	40.53	7	F7:11518	17112021_RID_2349_DP7.raw
N.DLSSIHQLPK.K	N	51.71	1136.6189	10	0.9	569.3173	2	34.51	1	F1:10039	12112021_RID_2349_DP1.raw
K.EGNLSPGAVDM(+15.99)IGDLLNED.S	N	51.30	1973.8888	19	0.5	987.9521	2	67.55	4	F4:20200	17112021_RID_2349_DP4.raw
S.AAYVLAGAGHK.V	N	50.59	1056.5715	11	1.1	529.2936	2	24.54	3	F3:6001	17112021_RID_2349_DP3.raw
D.LLNEDSGYYVSFIESLK.H	N	49.92	1975.9778	17	0.7	988.9969	2	68.81	3	F3:20708	17112021_RID_2349_DP3.raw
K.RFDEIVGGM(+15.99)D.Q	N	49.15	1153.5073	10	0.1	577.7610	2	38.54	3	F3:10739	17112021_RID_2349_DP3.raw
D.DIFAYEKR.F	N	48.19	1040.5291	8	0.1	521.2719	2	35.16	3	F3:9617	17112021_RID_2349_DP3.raw
K.YAMGAITTFTPY.Q	N	46.88	1334.6217	12	0.9	668.3187	2	64.07	4	F4:19119	17112021_RID_2349_DP4.raw
K.EGNLSPGAVDM(+15.99)IGD.L	N	46.09	1389.6082	14	0.2	695.8115	2	46.47	4	F4:13379	17112021_RID_2349_DP4.raw
K.RFDEIVGGMD.Q	N	45.54	1137.5125	10	-0.5	569.7632	2	45.56	3	F3:13157	17112021_RID_2349_DP3.raw
K.VTVLEASERPGGRVR.T	N	45.40	1624.9009	15	0.3	542.6411	3	30.01	1	F1:8563	12112021_RID_2349_DP1.raw
K.KFWEDDGIQGGK.S	N	44.74	1378.6517	12	-2.0	690.3318	2	35.62	7	F7:9823	17112021_RID_2349_DP7.raw
Q.HFSEALTAPVGR.I	N	42.43	1283.6622	12	0.5	642.8387	2	34.80	4	F4:9467	17112021_RID_2349_DP4.raw
K.YAMGAITTFTPYQF.Q	N	41.39	1609.7487	14	-0.2	805.8814	2	71.88	3	F3:21650	17112021_RID_2349_DP3.raw
L.SAAYVLAGAGHK.V	N	40.21	1143.6036	12	0.7	572.8095	2	26.75	5	F5:6718	17112021_RID_2349_DP5.raw
F.SEALTAPVGR.I	N	40.05	999.5349	10	-0.1	500.7746	2	30.20	3	F3:7936	17112021_RID_2349_DP3.raw
D.DIFAYEK.R	N	39.75	884.4279	7	0.8	885.4359	1	41.87	4	F4:11928	17112021_RID_2349_DP4.raw
K.RVGEVKKDPGLLK.Y	N	39.62	1437.8667	13	1.0	480.2967	3	39.89	1	F1:11650	12112021_RID_2349_DP1.raw
K.WSLDKYAMGAITTF.T	N	38.72	1602.7751	14	1.3	802.3959	2	67.34	3	F3:20215	17112021_RID_2349_DP3.raw

TRINITY_DN0
back to list

| Protein Coverage | Supporting Peptides |
Protein Coverage:

1 MIRFGNPSSS DKRRQRCWSW YWGGLLLLWA VAETRADIRY ATVYWLEAEK SFQIKDVLDK NGDAYGYYND TIQSTGWGIL

81 EIKAGYGNQR ISNEILMYAA GFLEGYLTAS HMSDNFANLF PVMIKNVIIE QKVKDFIQKQ DEWTRQQIKN NKDDPFWRNA

161 GYVIAQLDGL YMGNVEWAKR QKRTPLTDFE ISFLNAVGDL LDLIPALNSQ LRKSDFRSMP DVSRIYQWDM GHCSALIKVL

241 PGYENIYFAH SSWFTYAATL RIYKHWDFKI TDPQTKTGRA SFSSYPGLLV SLDDFYILGS GLIMLQTTNS VFNFFLLKQV

321 VPESLFAWER VRIANMMADS GKTWAQTFEK ENSGTYNNQY MILDTKKIKL QRSLEDGTLY IIEQIPKLVK YSDQTKVLRN

401 GYWPSYNIPF HKVIYNMSGY TEYVQRYGLE FSYEMAPRAK IFRRDQGKVT DMESMKHIMR YNNYKEDPYT KHNPCNTICC

481 RQDLSHKSPV PAGCYDSKVA DISMAAKFTA YAISGPPVEK DLPVFSWVHF NQTKHQGLPE SYNFDFVTMK PVL

Supporting Peptides:

Peptide	Uniq	-10lgP	Mass	Length	ppm	m/z	z	RT	Fraction	Scan	Source File	Area F1
R.SLEDGTLYIIEQIPK.L	N	90.40	1717.9138	15	0.5	859.9646	2	67.04	2	F2:19818	12112021_RID_2349_DP2.raw	1.408E6
K.ENSGTYNNQYMILDTK.K	N	89.12	1889.8466	16	0.8	945.9313	2	45.52	2	F2:12882	12112021_RID_2349_DP2.raw	
K.FTAYAISGPPVEK.D	Y	88.93	1378.7133	13	0.3	690.3641	2	43.87	2	F2:12308	12112021_RID_2349_DP2.raw	1.5569E[illegible]
R.YGLEFSYEMAPR.A	Y	88.54	1461.6598	12	0.5	731.8375	2	55.35	2	F2:16085	12112021_RID_2349_DP2.raw	2.0566E[illegible]
R.YGLEFSYEM(+15.99)APR.A	Y	85.59	1477.6547	12	0.8	739.8352	2	48.84	2	F2:14018	12112021_RID_2349_DP2.raw	
K.QVVPESLFAWER.V	Y	81.73	1459.7460	12	0.3	730.8805	2	59.87	2	F2:17521	12112021_RID_2349_DP2.raw	8.9291E[illegible]
R.IANMM(+15.99)ADSGK.T	N	76.74	1052.4630	10	-0.3	527.2386	2	24.17	2	F2:6829	12112021_RID_2349_DP2.raw	9.0092E[illegible]
K.TWAQTFEKENSGTYNNQYMILDTK.K	Y	75.94	2881.3228	24	0.0	961.4482	3	54.25	2	F2:15686	12112021_RID_2349_DP2.raw	
R.YNNYKEDPYTK.H	Y	75.77	1433.6462	11	1.1	717.8312	2	23.44	3	F3:5597	17112021_RID_2349_DP3.raw	2.3721E[illegible]
R.IANM(+15.99)MADSGK.T	N	75.38	1052.4630	10	-0.3	527.2386	2	24.17	2	F2:6725	12112021_RID_2349_DP2.raw	
R.DQGKVTDMESMK.H	N	72.75	1367.6061	12	0.5	684.8107	2	27.78	2	F2:6747	12112021_RID_2349_DP2.raw	
R.YATVYWLEAEK.S	N	72.57	1371.6710	11	0.7	686.8433	2	51.85	5	F5:15257	17112021_RID_2349_DP5.raw	
K.HQGLPESYNFDFVTM(+15.99)KPVL	N	71.29	2237.0828	19	0.8	746.7021	3	59.87	2	F2:17492	12112021_RID_2349_DP2.raw	
K.HQGLPESYNFDFVTMKPVL	N	70.10	2221.0876	19	0.3	1111.5514	2	65.61	2	F2:19349	12112021_RID_2349_DP2.raw	
K.ENSGTYNNQYM(+15.99)ILDTK.K	N	68.81	1905.8414	16	0.6	953.9286	2	41.00	2	F2:11307	12112021_RID_2349_DP2.raw	
K.FTAYAISGPPVEKD.L	Y	67.71	1493.7401	14	0.6	747.8778	2	44.70	3	F3:12805	17112021_RID_2349_DP3.raw	
R.NGYWPSYNIPFHK.V	Y	67.54	1621.7677	13	1.2	811.8921	2	51.95	2	F2:14850	12112021_RID_2349_DP2.raw	
R.DQGKVTDMESM(+15.99)K.H	N	66.01	1383.6010	12	0.7	692.8082	2	27.71	2	F2:6798	12112021_RID_2349_DP2.raw	
K.VADISMAAK.F	Y	62.72	904.4688	9	0.4	453.2418	2	29.56	2	F2:7416	12112021_RID_2349_DP2.raw	2.873E5
K.YSDQTKVLR.N	N	60.88	1108.5876	9	-0.4	555.3009	2	23.23	1	F1:6160	12112021_RID_2349_DP1.raw	4.0413E[illegible]
R.DQGKVTDM(+15.99)ESM(+15.99)K.H	N	60.55	1399.5959	12	-0.3	700.8051	2	27.86	2	F2:6821	12112021_RID_2349_DP2.raw	
K.TWAQTFEK.E	Y	59.18	1009.4869	8	0.9	505.7512	2	37.49	2	F2:10261	12112021_RID_2349_DP2.raw	
R.DQGKVTDM(+15.99)ESMK.H	N	55.19	1383.6010	12	0.0	692.8077	2	19.49	2	F2:3974	12112021_RID_2349_DP2.raw	

Peptide	Uniq	-10lgP	Mass	Length	ppm	m/z	z	RT	Fraction	Scan	Source File	Area F1
K.ENSGTYNNQYMILDTKK.I	N	48.36	2017.9415	17	0.5	673.6548	3	39.15	2	F2:10720	12112021_RID_2349_DP2.raw	
D.GTLYIIEQIPK.L	N	47.53	1273.7281	11	0.8	637.8719	2	53.61	4	F4:15815	17112021_RID_2349_DP4.raw	
K.TWAQTFEKENSGTYNNQYMILDTKK.I	Y	47.17	3009.4177	25	0.8	1004.1473	3	51.07	2	F2:14750	12112021_RID_2349_DP2.raw	
K.ENSGTYNNQYM(+15.99)ILDTKK.I	N	47.15	2033.9364	17	0.0	678.9861	3	39.23	2	F2:10730	12112021_RID_2349_DP2.raw	
K.SFQIKDVLDK.N	Y	46.69	1191.6499	10	0.9	596.8328	2	44.01	5	F5:12690	17112021_RID_2349_DP5.raw	
K.VLPGYENIYFAH.S	N	46.58	1421.6979	12	0.6	711.8566	2	53.52	2	F2:15534	12112021_RID_2349_DP2.raw	
K.VTDM(+15.99)ESMK.H	N	45.53	955.3990	8	-0.3	478.7067	2	24.77	2	F2:5754	12112021_RID_2349_DP2.raw	
K.HQGLPESYNFD.F	N	44.27	1305.5625	11	-0.5	653.7882	2	42.77	2	F2:11932	12112021_RID_2349_DP2.raw	
K.VTDMESM(+15.99)K.H	N	43.61	955.3990	8	-0.3	478.7067	2	24.77	2	F2:5660	12112021_RID_2349_DP2.raw	
K.VTDM(+15.99)ESM(+15.99)K.H	N	42.57	971.3940	8	-0.2	486.7042	2	24.80	2	F2:5663	12112021_RID_2349_DP2.raw	
R.IYKHWDFK.I	Y	42.43	1135.5814	8	0.2	379.5345	3	39.94	2	F2:10734	12112021_RID_2349_DP2.raw	
K.NNKDDPFWR.N	N	41.45	1190.5469	9	0.5	596.2810	2	35.13	5	F5:9629	17112021_RID_2349_DP5.raw	
K.VTDMESMK.H	N	39.74	939.4042	8	0.0	470.7094	2	24.77	2	F2:5674	12112021_RID_2349_DP2.raw	

TRINITY_DN132
back to list

| Protein Coverage | Supporting Peptides |
Protein Coverage:

1 MQGMGIEDKS SKMHCMKGKH VAIICGVVIV VGLILGLGLG LGLDTEACNP PEVNEQVSTK SPISNTPDVT NPSGSSVFCS

81 AK**NDENGPWT DFR**LPNYVHP VHYDLHLTPE MEAEVYTGMV NISIRLEEQT TKHLWLHLRE TK**ITEMPQLR TSSGQVIEIK**

161 **R**CFGYEPQEY VVIEAEEDLR PSNYFLSMKF KGYLNGSLVG FYSTTYGENG KIK**YIAATDH EPTDARK**SFP CFDEPNIKAT

241 YTISITHEHD YEAISNMPVE KTISIDNKWT KTIFKKSVPM STYLVAWAVH QFKYEERISS RGIPLRIYAQ PQQINTAIYA

321 ANVTKVVFDY FENYFNMNYS LPKLDKIAIP DFGTGAMENW GLITYR**ETNL LYDSQESAAS NK**QRVATVIA HELVHQWFGN

401 IVTMDWWDDL WLNEGFASFF EYMGVNAKEE KWQMLDQILI ADVLPVLKDD SLVSSHAITV NVSSPDEITS VFDGISYSKG

481 ASILR**MLEDW ISPDHFR**AGC QKYLKTHYFK NAKTDDFWK**A MEEVSGKPVR DVMDTWTRQM GYPVLKVDLS STVTQQRFLL**

561 **D**PK**ADPSKPS SEFSYK**WNIP VKWKEGNTSN IIFYNKSELA GITITRPSDL PPNSFLK**VNK DHVGFYRVNY EPQVWRALTD**

641 **IMMK**DHQNFN LADR**AGFIDD AFALAR**AGLL KYADALNLTR **YLQNEIEYIP WQRAVVAVSY IRHMLEDDKA LYPK**FQRYFG

721 NLVKPIASEL KWENDEDHIK SLLRTTVLEF ACNMDDPEAL GNASLLFKSW TSGISLDVNL RLLVYR**FGMQ NSGDEQAWNY**

801 **MFQK**YRIATL AQEKEK**LLYG LASVK**NITLL NRFLNCIKNT SLIR**SQDVFT VLRYISFNSY GKTMAWDWVR LNWEYLVK**RY

881 TLNDRNLGRL ISSISGTFNT ELQLWQMENF FER**YPDAGAG EASR**KQALET TKSNIEWLK**Q YRDDVATWLE NSEHPK**FA

Supporting Peptides:

Peptide	Uniq	-10lgP	Mass	Length	ppm	m/z	z	RT	Fraction	Scan	Source File	Area F1	Area F
R.AGFIDDAFALAR.A	Y	82.36	1265.6404	12	0.1	633.8275	2	61.37	1	F1:18887	12112021_RID_2349_DP1.raw	5.6662E6	2.7487I
R.YLQNEIEYIPWQR.A	Y	81.85	1750.8678	13	0.8	876.4419	2	57.41	1	F1:17599	12112021_RID_2349_DP1.raw	4.2599E6	1.9498I
R.ETNLLYDSQESAASNK.Q	Y	79.61	1768.8115	16	1.0	885.4139	2	37.10	2	F2:10076	12112021_RID_2349_DP2.raw	2.2867E5	2.6048I
K.YIAATDHEPTDAR.K	Y	77.36	1458.6738	13	0.8	730.3448	2	24.63	1	F1:6617	12112021_RID_2349_DP1.raw	5.1544E5	
R.HMLEDDKALYPK.F	Y	74.41	1458.7177	12	0.1	487.2466	3	38.63	1	F1:11459	12112021_RID_2349_DP1.raw	1.8307E6	
K.NDENGPWTDFR.L	Y	73.51	1349.5636	11	0.3	675.7893	2	46.11	1	F1:13951	12112021_RID_2349_DP1.raw	3.5973E5	5.0695I

total 32

Peptide	Uniq	-10lgP	Mass	Length	ppm	m/z	z	RT	Fraction	Scan	Source File	Area F1	Area F
K.YIAATDHEPTDARK.S	Y	69.82	1586.7688	14	0.7	794.3922	2	21.59	1	F1:5582	12112021_RID_2349_DP1.raw	3.4793E6	
R.MLEDWISPDHFR.A	Y	67.50	1544.7081	12	1.1	773.3622	2	54.12	1	F1:16564	12112021_RID_2349_DP1.raw	1.5051E5	
R.FGMQNSGDEQAWNYMFQK.Y	Y	66.58	2179.9092	18	-0.2	1090.9617	2	58.16	3	F3:17233	17112021_RID_2349_DP3.raw		3.2503[illegible]
R.SQDVFTVLR.Y	Y	66.02	1063.5662	9	-0.4	532.7902	2	47.74	1	F1:14400	12112021_RID_2349_DP1.raw	1.7467E6	2.2202[illegible]
K.VDLSSTVTQQR.F	Y	65.72	1232.6361	11	4.7	617.3282	2	32.63	2	F2:8691	12112021_RID_2349_DP2.raw	4.1055E7	6.9159[illegible]
R.VNYEPQVWR.A	Y	64.09	1189.5880	9	-0.2	595.8011	2	41.81	1	F1:12508	12112021_RID_2349_DP1.raw	5.2545E6	
R.DVMDTWTR.Q	Y	63.49	1022.4492	8	0.2	512.2319	2	43.94	1	F1:13265	12112021_RID_2349_DP1.raw	3.9974E5	
R.YPDAGAGEASR.K	Y	63.05	1092.4835	11	0.3	547.2492	2	22.63	1	F1:5917	12112021_RID_2349_DP1.raw	3.2708E5	3.7776[illegible]
R.AVVAVSYIR.H	Y	61.55	976.5706	9	-0.4	489.2924	2	40.15	1	F1:11950	12112021_RID_2349_DP1.raw	5.7775E6	1.9065[illegible]
K.VNKDHVGFYR.V	Y	60.88	1233.6254	10	0.0	617.8199	2	29.25	1	F1:8313	12112021_RID_2349_DP1.raw	2.0841E6	
R.TSSGQVIEIKR.C	Y	59.17	1216.6775	11	0.0	609.3460	2	27.07	1	F1:7509	12112021_RID_2349_DP1.raw	2.1937E6	
K.AMEEVSGKPVR.D	Y	59.15	1201.6124	11	0.6	601.8138	2	21.99	1	F1:5707	12112021_RID_2349_DP1.raw	2.2834E5	
K.ITEMPQLR.T	Y	58.29	986.5219	8	-0.1	494.2682	2	37.37	1	F1:11034	12112021_RID_2349_DP1.raw	1.9443E6	
K.TMAWDWVR.L	Y	54.89	1063.4910	8	0.1	532.7528	2	53.75	3	F3:15730	17112021_RID_2349_DP3.raw		
R.TSSGQVIEIK.R	Y	54.73	1060.5764	10	-0.3	531.2953	2	31.42	4	F4:8292	17112021_RID_2349_DP4.raw	2.0041E5	
R.QMGYPVLK.V	Y	54.53	934.4946	8	-0.5	468.2543	2	38.30	1	F1:11404	12112021_RID_2349_DP1.raw	2.02E6	
K.QYRDDVATWLENSEHPK.F	Y	53.56	2086.9707	17	0.5	696.6646	3	46.31	3	F3:13434	17112021_RID_2349_DP3.raw		
R.YISFNSYGK.T	Y	51.64	1077.5131	9	-0.5	539.7635	2	39.29	1	F1:11645	12112021_RID_2349_DP1.raw	8.8725E5	
K.ITEM(+15.99)PQLR.T	Y	49.55	1002.5168	8	0.8	502.2661	2	37.31	1	F1:10990	12112021_RID_2349_DP1.raw	8.909E5	
R.M(+15.99)LEDWISPDHFR.A	Y	47.01	1560.7031	12	1.0	781.3596	2	51.01	1	F1:15476	12112021_RID_2349_DP1.raw	6.1745E4	
R.ALTDIM(+15.99)MK.D	Y	47.00	937.4613	8	0.1	469.7380	2	44.85	1	F1:13477	12112021_RID_2349_DP1.raw	6.7217E4	
R.LNWEYLVK.R	Y	44.24	1063.5702	8	0.0	532.7924	2	53.65	3	F3:15847	17112021_RID_2349_DP3.raw		
K.ADPSKPSSEFSYK.W	Y	42.02	1441.6725	13	0.0	721.8435	2	29.31	1	F1:8282	12112021_RID_2349_DP1.raw	1.8949E5	
K.VDLSSTVTQQRFLLD.P	Y	41.88	1720.8995	15	0.1	861.4572	2	57.49	1	F1:17638	12112021_RID_2349_DP1.raw	6.4276E4	
K.LLYGLASVK.N	Y	41.79	962.5800	9	0.3	482.2974	2	45.33	3	F3:13034	17112021_RID_2349_DP3.raw		
R.HM(+15.99)LEDDKALYPK.F	Y	40.30	1474.7125	12	0.3	492.5783	3	35.26	1	F1:10364	12112021_RID_2349_DP1.raw	7.7591E5	

total 32

TRINITY_DN441
back to list

| Protein Coverage | Supporting Peptides |

Protein Coverage:

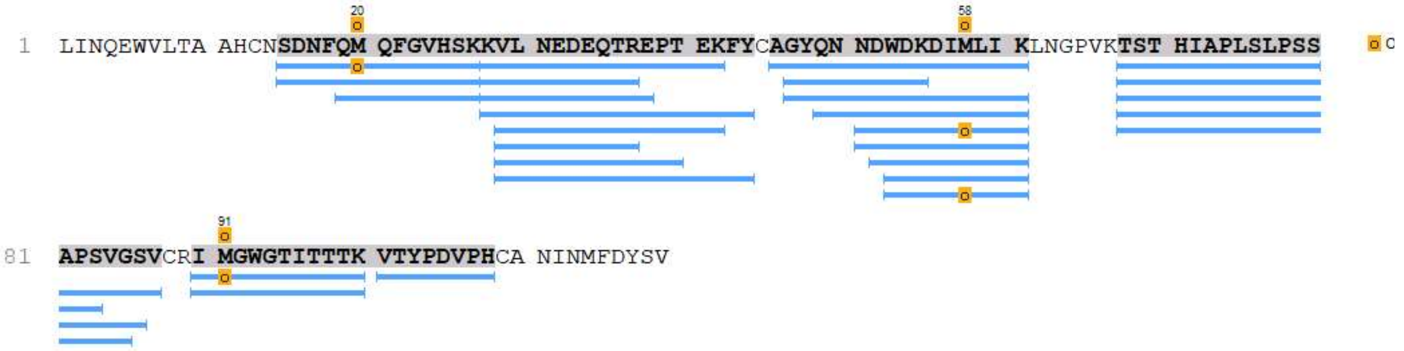

Supporting Peptides:

Peptide	Uniq	-10lgP	Mass	Length	ppm	m/z	z	RT	Fraction	Scan	Source File	Area F1	Area F2

total

Peptide	Uniq	-10lgP	Mass	Length	ppm	m/z	z	RT	Fraction	Scan	Source File	Area F1	Area F2
K.KVLNEDEQTREPTEK.F	Y	86.02	1814.9010	15	0.9	908.4586	2	20.05	3	F3:4488	17112021_RID_2349_DP3.raw	1.2849E6	1.492E6
K.VLNEDEQTREPTEK.F	Y	84.29	1686.8060	14	0.1	844.4104	2	21.98	3	F3:5114	17112021_RID_2349_DP3.raw	4.6932E5	8.6582E5
R.IMGWGTITTTK.V	N	78.23	1207.6271	11	-0.4	604.8206	2	47.11	3	F3:13741	17112021_RID_2349_DP3.raw	1.7839E6	2.7374E7
R.IM(+15.99)GWGTITTTK.V	N	67.99	1223.6221	11	0.3	612.8185	2	40.75	2	F2:11290	12112021_RID_2349_DP2.raw	7.1945E5	1.2257E7
K.TSTHIAPLSLPSSAPSVG.S	Y	64.26	1720.8995	18	0.0	861.4570	2	49.46	4	F4:14366	17112021_RID_2349_DP4.raw		
K.VLNEDEQTR.E	N	63.83	1102.5254	9	0.5	552.2703	2	19.87	4	F4:4313	17112021_RID_2349_DP4.raw		
A.GYQNNDWDKDIMLIK.L	Y	60.88	1851.8826	15	0.1	926.9486	2	52.32	4	F4:15363	17112021_RID_2349_DP4.raw		
K.TSTHIAPLSLPSS.A	N	60.21	1309.6877	13	-0.1	655.8511	2	44.76	4	F4:12858	17112021_RID_2349_DP4.raw		
K.TSTHIAPLSLPSSAPSVGSV.C	Y	59.73	1907.0000	20	-0.1	954.5072	2	53.14	4	F4:15583	17112021_RID_2349_DP4.raw		
D.WDKDIMLIK.L	N	57.12	1160.6263	9	-0.1	581.3204	2	46.64	2	F2:13338	12112021_RID_2349_DP2.raw	2.1169E5	9.915E6
N.DWDKDIMLIK.L	Y	56.94	1275.6533	10	0.0	638.8339	2	53.04	4	F4:15596	17112021_RID_2349_DP4.raw		
K.KVLNEDEQTR.E	N	55.31	1230.6204	10	0.8	616.3179	2	16.73	4	F4:3273	17112021_RID_2349_DP4.raw		
N.SDNFQMQFGVHSK.K	N	55.26	1523.6827	13	0.6	762.8491	2	41.31	4	F4:11754	17112021_RID_2349_DP4.raw		
N.NDWDKDIMLIK.L	Y	53.12	1389.6962	11	-0.9	695.8547	2	51.77	4	F4:15198	17112021_RID_2349_DP4.raw		
D.WDKDIM(+15.99)LIK.L	N	52.20	1176.6212	9	-0.2	589.3177	2	41.27	2	F2:11445	12112021_RID_2349_DP2.raw		6.6323E6
C.AGYQNNDWDKDIMLIK.L	Y	50.85	1922.9197	16	0.5	641.9808	3	52.44	4	F4:15407	17112021_RID_2349_DP4.raw		
K.TSTHIAPLSLPSSAPS.V	Y	49.77	1564.8097	16	0.1	783.4122	2	45.91	5	F5:13244	17112021_RID_2349_DP5.raw		
N.NDWDKDIM(+15.99)LIK.L	Y	48.22	1405.6912	11	0.8	703.8534	2	47.57	4	F4:13818	17112021_RID_2349_DP4.raw		
K.VLNEDEQTREPT.E	Y	47.20	1429.6685	12	0.5	715.8419	2	25.27	4	F4:6180	17112021_RID_2349_DP4.raw		
Y.QNNDWDKDIMLIK.L	Y	44.19	1631.7977	13	0.5	544.9401	3	50.35	4	F4:14806	17112021_RID_2349_DP4.raw		
K.KVLNEDEQTRE.P	N	43.42	1359.6630	11	0.2	680.8389	2	18.27	4	F4:3798	17112021_RID_2349_DP4.raw		
A.GYQNNDWDK.D	Y	42.46	1138.4679	9	-0.2	570.2411	2	26.69	4	F4:6675	17112021_RID_2349_DP4.raw		
K.VLNEDEQTREPTEKFY.C	Y	42.44	1996.9377	16	0.0	999.4762	2	36.81	4	F4:10173	17112021_RID_2349_DP4.raw		
N.SDNFQM(+15.99)QFGVHSK.K	N	42.19	1539.6776	13	1.2	770.8470	2	32.62	4	F4:8609	17112021_RID_2349_DP4.raw		
F.QMQFGVHSK.K	N	40.86	1060.5125	9	0.7	531.2639	2	24.46	4	F4:5894	17112021_RID_2349_DP4.raw		
K.KVLNEDEQTREPTEKFY.C	Y	40.79	2125.0327	17	0.6	709.3519	3	34.27	4	F4:9275	17112021_RID_2349_DP4.raw		
K.VTYPDVPH.C	N	39.33	926.4498	8	-0.6	464.2319	2	35.54	6	F6:9635	17112021_RID_2349_DP6.raw		
K.TSTHIAPLSLPSSAPSVGS.V	Y	39.31	1807.9315	19	0.9	904.9738	2	49.11	4	F4:14246	17112021_RID_2349_DP4.raw		

total

TRINITY_DN1507
back to list

| Protein Coverage | Supporting Peptides |
Protein Coverage:

1 MANVLCSCSE DCLEKKDCCT DYKSICKGET SWLKDQCASS SAAQCPAGFE QSPLILFSMD GFR**AGYLETW DSLMPNINK**L

81 KTCGTHAKYM RAVYPTKTFV NHYTIVTGLY PESHGIIDNN IYDVTLNLNF SLSAPTMTNP AWWGGQPIWH TVTYQGLK**AA**

161 **TYFWPGSEVK** INGSYPTIYK VYNK**SIPFEA R**VTEVLKWLD LPK**AERPDFV TLYIEEPDTT GHK**FGPVSGE IIMALQMADR

241 **TLGMLMEGLK** QRNLHNCVNL ILLADHGMEQ ISCNRLEYMT DYFDK**VDFFM YEGPAPR**IRS K**NVPKDFYTF DSEGIVR**NLT

321 CQKPKQYFK**A YLAKDLPKRL HYVNNIR**IDK **VNLMVDQQWM AVR**NKNYNRC NGGTHGYDNE FK**SMQAIFLA HGPGFK**GKNE

401 VTSFENIEVY NLMCDLLKLK PAPNNGTHGS LNHLLK**NPFY NPSPAK**EQSS PLLCPFGPVP SPDVSGCKCS SITDLGK**VNE**

481 **RLNLNNQAK**T ESEAHNLPYG RPQVLQNHSK YCLLHQAKYI SAYSQDILMP LWSSYTINKS PPTSVPPSAS DCLRLDVRIP

561 AAQSQTCSNY QPDLTITPGF LYPPNFGSSN FEQYDALITS NLVPMFKGFT RLWNYFHGTL LPKYARERNG LNVISGPIFD

641 YNYDGHFDSY DTIKEHVNDT KIPIPTHFFV VLTSCENQIN TPLNCPGSLK VLSFILPHRP DNSESCADTS PDNLWVEERI

721 QTHTARVRDV ELLTGLNFYS GLK**QSLPETL QLKTFLPIFV NPVN**

Supporting Peptides:

Peptide	Uniq	-10lgP	Mass	Length	ppm	m/z	z	RT	Fraction	Scan	Source File	Area F1	Area
K.DFYTFDSEGIVR.N	Y	89.31	1447.6619	12	0.7	724.8387	2	56.67	1	F1:17397	12112021_RID_2349_DP1.raw	1.3223E6	
K.NPFYNPSPAK.E	N	84.62	1133.5505	10	0.0	567.7825	2	34.93	1	F1:10324	12112021_RID_2349_DP1.raw	1.138E7	1.617
K.SMQAIFLAHGPGFK.G	Y	77.40	1502.7704	14	1.0	752.3932	2	50.76	1	F1:15524	12112021_RID_2349_DP1.raw	2.4779E5	
K.AATYFWPGSEVK.I	N	74.08	1354.6558	12	0.4	678.3354	2	50.11	1	F1:15322	12112021_RID_2349_DP1.raw	1.6628E6	1.581
K.TFLPIFVNPVN	Y	72.44	1259.6914	11	0.6	630.8533	2	74.54	1	F1:23258	12112021_RID_2349_DP1.raw	1.257E7	
R.AGYLETWDSLMPNINK.L	Y	71.20	1850.8872	16	0.6	926.4514	2	65.24	1	F1:20092	12112021_RID_2349_DP1.raw	7.1448E5	1.049
R.TLGMLMEGLK.Q	N	65.62	1091.5719	10	-0.1	546.7932	2	53.13	1	F1:16226	12112021_RID_2349_DP1.raw	2.9888E5	
K.VNERLNLNNQAK.T	Y	65.11	1411.7532	12	-0.6	471.5914	3	25.48	1	F1:6986	12112021_RID_2349_DP1.raw	2.9466E6	
K.VNLMVDQQWMAVR.N	N	63.98	1588.7854	13	0.2	795.4001	2	56.99	1	F1:17428	12112021_RID_2349_DP1.raw	1.178E5	
R.TLGMLM(+15.99)EGLK.Q	N	62.95	1107.5668	10	0.0	554.7906	2	53.06	1	F1:16245	12112021_RID_2349_DP1.raw	1.4377E5	
K.NVPKDFYTFDSEGIVR.N	Y	60.51	1885.9210	16	0.3	629.6478	3	51.20	1	F1:15596	12112021_RID_2349_DP1.raw	9.4201E5	
K.QSLPETLQLK.T	Y	58.68	1155.6499	10	0.3	578.8324	2	43.37	1	F1:13055	12112021_RID_2349_DP1.raw	7.7551E6	
R.AGYLETWDSLM(+15.99)PNINK.L	Y	57.35	1866.8822	16	0.6	934.4489	2	65.26	1	F1:20129	12112021_RID_2349_DP1.raw	2.6064E5	
K.AYLAKDLPK.R	N	54.37	1017.5858	9	-0.3	509.8000	2	31.16	1	F1:8942	12112021_RID_2349_DP1.raw	1.0616E6	
K.VDFFMYEGPAPR.I	N	48.08	1427.6543	12	0.8	714.8350	2	56.53	1	F1:17314	12112021_RID_2349_DP1.raw	2.1345E4	
K.AERPDFVTLYIEEPDTTGHK.F	Y	47.01	2317.1226	20	0.9	773.3821	3	51.14	1	F1:15544	12112021_RID_2349_DP1.raw	1.1012E6	
K.SIPFEAR.V	N	41.70	818.4286	7	-0.3	410.2214	2	34.74	1	F1:10150	12112021_RID_2349_DP1.raw	2.9409E6	
K.RLHYVNNIR.I	Y	41.43	1183.6573	9	0.5	395.5599	3	35.06	1	F1:10272	12112021_RID_2349_DP1.raw	8.4331E4	

total [illegible]

TRINITY_DN1507
back to list

| Protein Coverage | Supporting Peptides |

Protein Coverage:

```
1   MANVLCSCSE DCLEKKDCCT DYKSICKGET SWLKDQCASS SAAQCPAGFE QSPLILFSMD GFRAGYLETW DSLMPNINKL
81  KTCGTHAKYM RAVYPTKTFV NHYTIVTGLY PESHGIIDNN IYDVTLNLNF SLSAPTMTNP AWWGGQPIWH TVTYQGLKAA
161 TYFWPGSEVK INGSYPTIYK VYNKSIPFEA RVTEVLKWLD LPKAERPDFV TLYIEEPDTT GHKFGPVSGE IIMALQMADR
241 TLGMLMEGLK QRNLHNCVNL ILLADHGMEQ ISCNRLEYMT DYFDKVDFFM YEGPAPRIRS KNVPKDFYTF DSEGIVRNLT
321 CQKPKQYFKA YLAKDLPKRL HYVNNIRIDK VNLMVDQQWM AVRNKNYNRC NGGTHGYDNE FKSMQAIFLA HGPGFKGKNE
401 VTSFENIEVY NLMCDLLKLK PAPNNGTHGS LNHLLKNPFY NPSPAKEQSS PLLCPFGPVP SPDVSGCKCS SITDLGKVNE
481 RLNLNNQAKT ESEAHNLPYG RPQVLQNHSK YCLLHQAKYI SAYSQDILMP LWSSYTINKS PPTSVPPSAS DCLRLDVRIP
561 AAQSQTCSNY QPDLTITPGF LYPPNFGSSN FEQYDALITS NLVPMFKGFT RLWNYFHGTL LPKYARERNG LNVISGPIFD
641 YNYDGHFDSY DTIKEHVNDT KIPIPTHFFV VLTSCENQIN TPLNCPGSLK VLSFILPHRP DNSESCADTS PDNLWVEERI
721 QTHTARVRDV ELLTGLNFYS GLKQSLPETL QLKTFLPIFV NPVN
```

Supporting Peptides:

Peptide	Uniq	-10lgP	Mass	Length	ppm	m/z	z	RT	Fraction	Scan	Source File	Area F1	Area
K.DFYTFDSEGIVR.N	Y	89.31	1447.6619	12	0.7	724.8387	2	56.67	1	F1:17397	12112021_RID_2349_DP1.raw	1.3223E6	
K.NPFYNPSPAK.E	N	84.62	1133.5505	10	0.0	567.7825	2	34.93	1	F1:10324	12112021_RID_2349_DP1.raw	1.138E7	1.617
K.SMQAIFLAHGPGFK.G	Y	77.40	1502.7704	14	1.0	752.3932	2	50.76	1	F1:15524	12112021_RID_2349_DP1.raw	2.4779E5	
K.AATYFWPGSEVK.I	N	74.08	1354.6558	12	0.4	678.3354	2	50.11	1	F1:15322	12112021_RID_2349_DP1.raw	1.6628E6	1.581
K.TFLPIFVNPVN	Y	72.44	1259.6914	11	0.6	630.8533	2	74.54	1	F1:23258	12112021_RID_2349_DP1.raw	1.257E7	
R.AGYLETWDSLMPNINK.L	Y	71.20	1850.8872	16	0.6	926.4514	2	65.24	1	F1:20092	12112021_RID_2349_DP1.raw	7.1448E5	1.049
R.TLGMLMEGLK.Q	N	65.62	1091.5719	10	-0.1	546.7932	2	53.13	1	F1:16226	12112021_RID_2349_DP1.raw	2.9888E5	
K.VNERLNLNNQAK.T	Y	65.11	1411.7532	12	-0.6	471.5914	3	25.48	1	F1:6986	12112021_RID_2349_DP1.raw	2.9466E6	
K.VNLMVDQQWMAVR.N	N	63.98	1588.7854	13	0.2	795.4001	2	56.99	1	F1:17428	12112021_RID_2349_DP1.raw	1.178E5	
R.TLGMLM(+15.99)EGLK.Q	N	62.95	1107.5668	10	0.0	554.7906	2	53.06	1	F1:16245	12112021_RID_2349_DP1.raw	1.4377E5	
K.NVPKDFYTFDSEGIVR.N	Y	60.51	1885.9210	16	0.3	629.6478	3	51.20	1	F1:15596	12112021_RID_2349_DP1.raw	9.4201E5	
K.QSLPETLQLK.T	Y	58.68	1155.6499	10	0.3	578.8324	2	43.37	1	F1:13055	12112021_RID_2349_DP1.raw	7.7551E6	
R.AGYLETWDSLM(+15.99)PNINK.L	Y	57.35	1866.8822	16	0.6	934.4489	2	65.26	1	F1:20129	12112021_RID_2349_DP1.raw	2.6064E5	
K.AYLAKDLPK.R	N	54.37	1017.5858	9	-0.3	509.8000	2	31.16	1	F1:8942	12112021_RID_2349_DP1.raw	1.0616E6	
K.VDFFMYEGPAPR.I	N	48.08	1427.6543	12	0.8	714.8350	2	56.53	1	F1:17314	12112021_RID_2349_DP1.raw	2.1345E4	
K.AERPDFVTLYIEEPDTTGHK.F	Y	47.01	2317.1226	20	0.9	773.3821	3	51.14	1	F1:15544	12112021_RID_2349_DP1.raw	1.1012E6	
K.SIPFEAR.V	N	41.70	818.4286	7	-0.3	410.2214	2	34.74	1	F1:10150	12112021_RID_2349_DP1.raw	2.9409E6	
K.RLHYVNNIR.I	Y	41.43	1183.6573	9	0.5	395.5599	3	35.06	1	F1:10272	12112021_RID_2349_DP1.raw	8.4331E4	

total [illegible]

TRINITY_DN1507
back to list

| Protein Coverage | Supporting Peptides |
Protein Coverage:

```
1   MASGCSCDDK CTERQACCQD YEDTCVLPTQ SWSCSKLRCS EKRMANVLCS CSEDCLEKKD CCTDYKSICK GETSWLKDQC   C
                                                     117
81  ASSSAAQCPA GFEQSPLILF SMDGFRAGYL ETWDSLMPNI NKLKTCGTHA KYMRAVYPTK TFVNHYTIVT GLYPESHGII
161 DNNIYDVTLN LNFSLSAPTM TNPAWWGGQP IWHTVTYQGL KAATYFWPGS EVKINGSYPT IYKVYNKSIP FEARVTEVLK
241 WLDLPKAERP DFVTLYIEEP DTTGHKFGPV SGEIIMALQM ADRTLGMLME GLKQRNLHNC VNLILLADHG MEQISCNRLE
321 YMTDYFDKVD FFMYEGPAPR IRSKNVPKDF YTFDSEGIVR NLTCQKPKQY FKAYLAKDLP KRLHYVNNIR IDKVNLMVDQ
401 QWMAVRNKNY NRCNGGTHGY DNEFKSMQAI FLAHGPGFKG KNEVTSFENI EVYNLMCDLL KLKPAPNNGT HGSLNHLLKN
481 PFYNPSPAKE QSSPLLCPFG PVPSPDVSGC KCSSITDLGK VNERLNLNNQ AKTESEAHNL PYGRPQVLQN HSKYCLLHQA
561 KYISAYSQDI LMPLWSSYTI NKSPPTSVPP SASDCLRLDV RIPAAQSQTC SNYQPDLTIT PGFLYPPNFG SSNFEQYDAL
641 ITSNLVPMFK GFTRLWNYFH GTLLPKYARE RNGLNVISGP IFDYNYDGHF DSYDTIKEHV NDTKIPIPTH FFVVLTSCEN
721 QINTPLNCPG SLKVLSFILP HRPDNSESCA DTSPDNLWVE ERIQTHTARV RDVELLTGLN FYSGLKQSLP ETLQLKTFLP
801 IFVNPVN
```

Supporting Peptides:

Peptide	Uniq	-10lgP	Mass	Length	ppm	m/z	z	RT	Fraction	Scan	Source File	Area F1	Area
K.DFYTFDSEGIVR.N	Y	89.31	1447.6619	12	0.7	724.8387	2	56.67	1	F1:17397	12112021_RID_2349_DP1.raw	1.3223E6	
K.NPFYNPSPAK.E	N	84.62	1133.5505	10	0.0	567.7825	2	34.93	1	F1:10324	12112021_RID_2349_DP1.raw	1.138E7	1.617
K.SMQAIFLAHGPGFK.G	Y	77.40	1502.7704	14	1.0	752.3932	2	50.76	1	F1:15524	12112021_RID_2349_DP1.raw	2.4779E5	
K.AATYFWPGSEVK.I	N	74.08	1354.6558	12	0.4	678.3354	2	50.11	1	F1:15322	12112021_RID_2349_DP1.raw	1.6628E6	1.581
K.TFLPIFVNPVN	Y	72.44	1259.6914	11	0.6	630.8533	2	74.54	1	F1:23258	12112021_RID_2349_DP1.raw	1.257E7	
R.AGYLETWDSLMPNINK.L	Y	71.20	1850.8872	16	0.6	926.4514	2	65.24	1	F1:20092	12112021_RID_2349_DP1.raw	7.1448E5	1.049
R.TLGMLMEGLK.Q	N	65.62	1091.5719	10	-0.1	546.7932	2	53.13	1	F1:16226	12112021_RID_2349_DP1.raw	2.9888E5	
K.VNERLNLNNQAK.T	Y	65.11	1411.7532	12	-0.6	471.5914	3	25.48	1	F1:6986	12112021_RID_2349_DP1.raw	2.9466E6	
K.VNLMVDQQWMAVR.N	N	63.98	1588.7854	13	0.2	795.4001	2	56.99	1	F1:17428	12112021_RID_2349_DP1.raw	1.178E5	
R.TLGMLM(+15.99)EGLK.Q	N	62.95	1107.5668	10	0.0	554.7906	2	53.06	1	F1:16245	12112021_RID_2349_DP1.raw	1.4377E5	
K.NVPKDFYTFDSEGIVR.N	Y	60.51	1885.9210	16	0.3	629.6478	3	51.20	1	F1:15596	12112021_RID_2349_DP1.raw	9.4201E5	
K.QSLPETLQLK.T	Y	58.68	1155.6499	10	0.3	578.8324	2	43.37	1	F1:13055	12112021_RID_2349_DP1.raw	7.7551E6	
R.AGYLETWDSLM(+15.99)PNINK.L	Y	57.35	1866.8822	16	0.6	934.4489	2	65.26	1	F1:20129	12112021_RID_2349_DP1.raw	2.6064E5	
K.AYLAKDLPK.R	N	54.37	1017.5858	9	-0.3	509.8000	2	31.16	1	F1:8942	12112021_RID_2349_DP1.raw	1.0616E6	
K.VDFFMYEGPAPR.I	N	48.08	1427.6543	12	0.8	714.8350	2	56.53	1	F1:17314	12112021_RID_2349_DP1.raw	2.1345E4	
K.AERPDFVTLYIEEPDTTGHK.F	Y	47.01	2317.1226	20	0.9	773.3821	3	51.14	1	F1:15544	12112021_RID_2349_DP1.raw	1.1012E6	
K.SIPFEAR.V	N	41.70	818.4286	7	-0.3	410.2214	2	34.74	1	F1:10150	12112021_RID_2349_DP1.raw	2.9409E6	
K.RLHYVNNIR.I	Y	41.43	1183.6573	9	0.5	395.5599	3	35.06	1	F1:10272	12112021_RID_2349_DP1.raw	8.4331E4	

total [illegible]

TRINITY_DN1507
back to list

| Protein Coverage | Supporting Peptides |
Protein Coverage:

```
1   MIQQKVLFIS LVAVALGLGL GLGLKESVEP QVSCRYRCNE TFSKMASGCS CDDKCTERQA CCQDYEDTCV LPTQSWSCSK
81  LRCSEKRMAN VLCSCSEDCL EKKDCCTDYK SICKGETSWL KDQCASSSAA QCPAGFEQSP LILFSMDGFR AGYLETWDSL
161 MPNINKLKTC GTHAKYMRAV YPTKTFVNHY TIVTGLYPES HGIIDNNIYD VTLNLNFSLS APTMTNPAWW GGQPIWHTVT
241 YQGLKAATYF WPGSEVKING SYPTIYKVYN KSIPFEARVT EVLKWLDLPK AERPDFVTLY IEEPDTTGHK FGPVSGEIIM
321 ALQMADRTLG MLMEGLKQRN LHNCVNLILL ADHGMEQISC NRLEYMTDYF DKVDFFMYEG PAPRIRSKNV PKDFYTFDSE
401 GIVRNLTCQK PKQYFKAYLA KDLPKRLHYV NNIRIDKVNL MVDQQWMAVR NKNYNRCNGG THGYDNEFKS MQAIFLAHGP
481 GFKGKNEVTS FENIEVYNLM CDLLKLKPAP NNGTHGSLNH LLKNPFYNPS PAKEQSSPLL CPFGPVPSPD VSGCKCSSIT
561 DLGKVNERLN LNNQAKTESE AHNLPYGRPQ VLQNHSKYCL LHQAKYISAY SQDILMPLWS SYTINKSPPT SVPPSASDCL
641 RLDVRIPAAQ SQTCSNYQPD LTITPGFLYP PNFGSSNFEQ YDALITSNLV PMFKGFTRLW NYFHGTLLPK YARERNGLNV
721 ISGPIFDYNY DGHFDSYDTI KEHVNDTKIP IPTHFFVVLT SCENQINTPL NCPGSLKVLS FILPHRPDNS ESCADTSPDN
801 LWVEERIQTH TARVRDVELL TGLNFYSGLK QSLPETLQLK TFLPIFVNPV N
```

Supporting Peptides:

Peptide	Uniq	-10lgP	Mass	Length	ppm	m/z	z	RT	Fraction	Scan	Source File	Area F1	Area
K.DFYTFDSEGIVR.N	Y	89.31	1447.6619	12	0.7	724.8387	2	56.67	1	F1:17397	12112021_RID_2349_DP1.raw	1.3223E6	
K.NPFYNPSPAK.E	N	84.62	1133.5505	10	0.0	567.7825	2	34.93	1	F1:10324	12112021_RID_2349_DP1.raw	1.138E7	1.617[illegible]
K.SMQAIFLAHGPGFK.G	Y	77.40	1502.7704	14	1.0	752.3932	2	50.76	1	F1:15524	12112021_RID_2349_DP1.raw	2.4779E5	
K.AATYFWPGSEVK.I	N	74.08	1354.6558	12	0.4	678.3354	2	50.11	1	F1:15322	12112021_RID_2349_DP1.raw	1.6628E6	1.581[illegible]
K.TFLPIFVNPVN	Y	72.44	1259.6914	11	0.6	630.8533	2	74.54	1	F1:23258	12112021_RID_2349_DP1.raw	1.257E7	
R.AGYLETWDSLMPNINK.L	Y	71.20	1850.8872	16	0.6	926.4514	2	65.24	1	F1:20092	12112021_RID_2349_DP1.raw	7.1448E5	1.049[illegible]
R.TLGMLMEGLK.Q	N	65.62	1091.5719	10	-0.1	546.7932	2	53.13	1	F1:16226	12112021_RID_2349_DP1.raw	2.9888E5	
K.VNERLNLNNQAK.T	Y	65.11	1411.7532	12	-0.6	471.5914	3	25.48	1	F1:6986	12112021_RID_2349_DP1.raw	2.9466E6	
K.VNLMVDQQWMAVR.N	N	63.98	1588.7854	13	0.2	795.4001	2	56.99	1	F1:17428	12112021_RID_2349_DP1.raw	1.178E5	
R.TLGMLM(+15.99)EGLK.Q	N	62.95	1107.5668	10	0.0	554.7906	2	53.06	1	F1:16245	12112021_RID_2349_DP1.raw	1.4377E5	
K.NVPKDFYTFDSEGIVR.N	Y	60.51	1885.9210	16	0.3	629.6478	3	51.20	1	F1:15596	12112021_RID_2349_DP1.raw	9.4201E5	
K.QSLPETLQLK.T	Y	58.68	1155.6499	10	0.3	578.8324	2	43.37	1	F1:13055	12112021_RID_2349_DP1.raw	7.7551E6	
R.AGYLETWDSLM(+15.99)PNINK.L	Y	57.35	1866.8822	16	0.6	934.4489	2	65.26	1	F1:20129	12112021_RID_2349_DP1.raw	2.6064E5	
K.AYLAKDLPK.R	N	54.37	1017.5858	9	-0.3	509.8000	2	31.16	1	F1:8942	12112021_RID_2349_DP1.raw	1.0616E6	
K.VDFFMYEGPAPR.I	N	48.08	1427.6543	12	0.8	714.8350	2	56.53	1	F1:17314	12112021_RID_2349_DP1.raw	2.1345E4	
K.AERPDFVTLYIEEPDTTGHK.F	Y	47.01	2317.1226	20	0.9	773.3821	3	51.14	1	F1:15544	12112021_RID_2349_DP1.raw	1.1012E6	
K.SIPFEAR.V	N	41.70	818.4286	7	-0.3	410.2214	2	34.74	1	F1:10150	12112021_RID_2349_DP1.raw	2.9409E6	
K.RLHYVNNIR.I	Y	41.43	1183.6573	9	0.5	395.5599	3	35.06	1	F1:10272	12112021_RID_2349_DP1.raw	8.4331E4	
												**total** [illegible]	

TRINITY_DN1507
back to list

| Protein Coverage | Supporting Peptides |
Protein Coverage:

1 MIQQKVLFIS LVAVALGLGL GLGLKESVEP QAQSWSCSKL RCSEKRMANV LCSCSEDCLE KKDCCTDYKS ICKGETSWLK C

120

81 DQCASSSAAQ CPAGFEQSPL ILFSMDGFRA GYLETWDSLM PNINKLKTCG THAKYMRAVY PTKTFVNHYT IVTGLYPESH

161 GIIDNNIYDV TLNLNFSLSA PTMTNPAWWG GQPIWHTVTY QGLKAATYFW PGSEVKINGS YPTIYKVYNK SIPFEARVTE

241 VLKWLDLPKA ERPDFVTLYI EEPDTTGHKF GPVSGEIIMA LQMADRTLGM LMEGLKQRNL HNCVNLILLA DHGMEQISCN

321 RLEYMTDYFD KVDFFMYEGP APRIRSKNVP KDFYTFDSEG IVRNLTCQKP KQYFKAYLAK DLPKRLHYVN NIRIDKVNLM

401 VDQQWMAVRN KNYNRCNGGT HGYDNEFKSM QAIFLAHGPG FKGKNEVTSF ENIEVYNLMC DLLKLKPAPN NGTHGSLNHL

481 LKNPFYNPSP AKEQSSPLLC PFGPVPSPDV SGCKCSSITD LGKVNERLNL NNQAKTESEA HNLPYGRPQV LQNHSKYCLL

561 HQAKYISAYS QDILMPLWSS YTINKSPPTS VPPSASDCLR LDVRIPAAQS QTCSNYQPDL TITPGFLYPP NFGSSNFEQY

641 DALITSNLVP MFKGFTRLWN YFHGTLLPKY ARERNGLNVI SGPIFDYNYD GHFDSYDTIK EHVNDTKIPI PTHFFVVLTS

721 CENQINTPLN CPGSLKVLSF ILPHRPDNSE SCADTSPDNL WVEERIQTHT ARVRDVELLT GLNFYSGLKQ SLPETLQLKT

801 FLPIFVNPVN

Supporting Peptides:

Peptide	Uniq	-10lgP	Mass	Length	ppm	m/z	z	RT	Fraction	Scan	Source File	Area F1	Area
K.DFYTFDSEGIVR.N	Y	89.31	1447.6619	12	0.7	724.8387	2	56.67	1	F1:17397	12112021_RID_2349_DP1.raw	1.3223E6	
K.NPFYNPSPAK.E	N	84.62	1133.5505	10	0.0	567.7825	2	34.93	1	F1:10324	12112021_RID_2349_DP1.raw	1.138E7	1.617
K.SMQAIFLAHGPGFK.G	Y	77.40	1502.7704	14	1.0	752.3932	2	50.76	1	F1:15524	12112021_RID_2349_DP1.raw	2.4779E5	
K.AATYFWPGSEVK.I	N	74.08	1354.6558	12	0.4	678.3354	2	50.11	1	F1:15322	12112021_RID_2349_DP1.raw	1.6628E6	1.581
K.TFLPIFVNPVN	Y	72.44	1259.6914	11	0.6	630.8533	2	74.54	1	F1:23258	12112021_RID_2349_DP1.raw	1.257E7	
R.AGYLETWDSLMPNINK.L	Y	71.20	1850.8872	16	0.6	926.4514	2	65.24	1	F1:20092	12112021_RID_2349_DP1.raw	7.1448E5	1.049
R.TLGMLMEGLK.Q	N	65.62	1091.5719	10	-0.1	546.7932	2	53.13	1	F1:16226	12112021_RID_2349_DP1.raw	2.9888E5	
K.VNERLNLNNQAK.T	Y	65.11	1411.7532	12	-0.6	471.5914	3	25.48	1	F1:6986	12112021_RID_2349_DP1.raw	2.9466E6	
K.VNLMVDQQWMAVR.N	N	63.98	1588.7854	13	0.2	795.4001	2	56.99	1	F1:17428	12112021_RID_2349_DP1.raw	1.178E5	
R.TLGMLM(+15.99)EGLK.Q	N	62.95	1107.5668	10	0.0	554.7906	2	53.06	1	F1:16245	12112021_RID_2349_DP1.raw	1.4377E5	
K.NVPKDFYTFDSEGIVR.N	Y	60.51	1885.9210	16	0.3	629.6478	3	51.20	1	F1:15596	12112021_RID_2349_DP1.raw	9.4201E5	
K.QSLPETLQLK.T	Y	58.68	1155.6499	10	0.3	578.8324	2	43.37	1	F1:13055	12112021_RID_2349_DP1.raw	7.7551E6	
R.AGYLETWDSLM(+15.99)PNINK.L	Y	57.35	1866.8822	16	0.6	934.4489	2	65.26	1	F1:20129	12112021_RID_2349_DP1.raw	2.6064E5	
K.AYLAKDLPK.R	N	54.37	1017.5858	9	-0.3	509.8000	2	31.16	1	F1:8942	12112021_RID_2349_DP1.raw	1.0616E6	
K.VDFFMYEGPAPR.I	N	48.08	1427.6543	12	0.8	714.8350	2	56.53	1	F1:17314	12112021_RID_2349_DP1.raw	2.1345E4	
K.AERPDFVTLYIEEPDTTGHK.F	Y	47.01	2317.1226	20	0.9	773.3821	3	51.14	1	F1:15544	12112021_RID_2349_DP1.raw	1.1012E6	
K.SIPFEAR.V	N	41.70	818.4286	7	-0.3	410.2214	2	34.74	1	F1:10150	12112021_RID_2349_DP1.raw	2.9409E6	
K.RLHYVNNIR.I	Y	41.43	1183.6573	9	0.5	395.5599	3	35.06	1	F1:10272	12112021_RID_2349_DP1.raw	8.4331E4	

total 1

TRINITY_DN1507

back to list

| Protein Coverage | Supporting Peptides |

Protein Coverage:

```
1   MEMFPEDETE RGKKRTLDLI DFQTETIIKR STLRKYKILC VVLFISLVAV ALGLGLGLGL KESVEPQVSC RYRCNETFSK
81  MASGCSCDDK CTERQACCQD YEDTCVLPTQ SWSCSKLRCS EKRMANVLCS CSEDCLEKKD CCTDYKSICK GETSWLKDQC
                                197
161 ASSSAAQCPA GFEQSPLILF SMDGFRAGYL ETWDSLMPNI NKLKTCGTHA KYMRAVYPTK TFVNHYTIVT GLYPESHGII
241 DNNIYDVTLN LNFSLSAPTM TNPAWWGGQP IWHTVTYQGL KAATYFWPGS EVKINGSYPT IYKVYNKSIP FEARVTEVLK
321 WLDLPKAERP DFVTLYIEEP DTTGHKFGPV SGEIIMALQM ADRTLGMLME GLKQRNLHNC VNLILLADHG MEQISCNRLE
401 YMTDYFDKVD FFMYEGPAPR IRSKNVPKDF YTFDSEGIVR NLTCQKPKQY FKAYLAKDLP KRLHYVNNIR IDKVNLMVDQ
481 QWMAVRNKNY NRCNGGTHGY DNEFKSMQAI FLAHGPGFKG KNEVTSFENI EVYNLMCDLL KLKPAPNNGT HGSLNHLLKN
561 PFYNPSPAKE QSSPLLCPFG PVPSPDVSGC KCSSITDLGK VNERLNLNNQ AKTESEAHNL PYGRPQVLQN HSKYCLLHQA
641 KYISAYSQDI LMPLWSSYTI NKSPPTSVPP SASDCLRLDV RIPAAQSQTC SNYQPDLTIT PGFLYPPNFG SSNFEQYDAL
721 ITSNLVPMFK GFTRLWNYFH GTLLPKYARE RNGLNVISGP IFDYNYDGHF DSYDTIKEHV NDTKIPIPTH FFVVLTSCEN
801 QINTPLNCPG SLKVLSFILP HRPDNSESCA DTSPDNLWVE ERIQTHTARV RDVELLTGLN FYSGLKQSLP ETLQLKTFLP
881 IFVNPVN
```

Supporting Peptides:

Peptide	Uniq	-10lgP	Mass	Length	ppm	m/z	z	RT	Fraction	Scan	Source File	Area F1	Area
K.DFYTFDSEGIVR.N	Y	89.31	1447.6619	12	0.7	724.8387	2	56.67	1	F1:17397	12112021_RID_2349_DP1.raw	1.3223E6	
K.NPFYNPSPAK.E	N	84.62	1133.5505	10	0.0	567.7825	2	34.93	1	F1:10324	12112021_RID_2349_DP1.raw	1.138E7	1.617
K.SMQAIFLAHGPGFK.G	Y	77.40	1502.7704	14	1.0	752.3932	2	50.76	1	F1:15524	12112021_RID_2349_DP1.raw	2.4779E5	
K.AATYFWPGSEVK.I	N	74.08	1354.6558	12	0.4	678.3354	2	50.11	1	F1:15322	12112021_RID_2349_DP1.raw	1.6628E6	1.581
K.TFLPIFVNPVN	Y	72.44	1259.6914	11	0.6	630.8533	2	74.54	1	F1:23258	12112021_RID_2349_DP1.raw	1.257E7	
R.AGYLETWDSLMPNINK.L	Y	71.20	1850.8872	16	0.6	926.4514	2	65.24	1	F1:20092	12112021_RID_2349_DP1.raw	7.1448E5	1.049
R.TLGMLMEGLK.Q	N	65.62	1091.5719	10	-0.1	546.7932	2	53.13	1	F1:16226	12112021_RID_2349_DP1.raw	2.9888E5	
K.VNERLNLNNQAK.T	Y	65.11	1411.7532	12	-0.6	471.5914	3	25.48	1	F1:6986	12112021_RID_2349_DP1.raw	2.9466E6	
K.VNLMVDQQWMAVR.N	N	63.98	1588.7854	13	0.2	795.4001	2	56.99	1	F1:17428	12112021_RID_2349_DP1.raw	1.178E5	
R.TLGMLM(+15.99)EGLK.Q	N	62.95	1107.5668	10	0.0	554.7906	2	53.06	1	F1:16245	12112021_RID_2349_DP1.raw	1.4377E5	
K.NVPKDFYTFDSEGIVR.N	Y	60.51	1885.9210	16	0.3	629.6478	3	51.20	1	F1:15596	12112021_RID_2349_DP1.raw	9.4201E5	
K.QSLPETLQLK.T	Y	58.68	1155.6499	10	0.3	578.8324	2	43.37	1	F1:13055	12112021_RID_2349_DP1.raw	7.7551E6	
R.AGYLETWDSLM(+15.99)PNINK.L	Y	57.35	1866.8822	16	0.6	934.4489	2	65.26	1	F1:20129	12112021_RID_2349_DP1.raw	2.6064E5	
K.AYLAKDLPK.R	N	54.37	1017.5858	9	-0.3	509.8000	2	31.16	1	F1:8942	12112021_RID_2349_DP1.raw	1.0616E6	
K.VDFFMYEGPAPR.I	N	48.08	1427.6543	12	0.8	714.8350	2	56.53	1	F1:17314	12112021_RID_2349_DP1.raw	2.1345E4	
K.AERPDFVTLYIEEPDTTGHK.F	Y	47.01	2317.1226	20	0.9	773.3821	3	51.14	1	F1:15544	12112021_RID_2349_DP1.raw	1.1012E6	
K.SIPFEAR.V	N	41.70	818.4286	7	-0.3	410.2214	2	34.74	1	F1:10150	12112021_RID_2349_DP1.raw	2.9409E6	
K.RLHYVNNIR.I	Y	41.43	1183.6573	9	0.5	395.5599	3	35.06	1	F1:10272	12112021_RID_2349_DP1.raw	8.4331E4	
													**total** 1

TRINITY_DN30
back to list

| Protein Coverage | Supporting Peptides |
Protein Coverage:

1 MIQPLLVVTC LVVFPYQGSS IILESGNVND YEVVYPQKVT ALPKGAIQQL EQKYEDAIQY QFKVKGEPVV LHLEKNKDFF
81 PENYSETHYS PDDREITTNP PVEDHCYYHG HIQNEADSTA SISACNGLKG HFKLQGETYL IEPLKIPDSE AHAVYKYENI
161 EKEDEAPKMC GV**TQTNWESD EPIKKA**SQLV ATSEQQSYYD RFR**YIK**LVIV VDHSMVTKYN GDLTAIR**TKI YQR**VNILNEI
241 **YGPFSIR**VPL VGVEIWVNRD FISVTSASNV TLKSFGEWRK RDLLSRKRHD YAQLLTAINF DEQIVGRAYI STMCQPLRSV
321 AIVEDYSPID HAVAATMAHE MGHNLGMHHD GKQCNCGANS CIMAPEISDP PPTYFSDCSR **NYYQTFLTNY RPD**CTLIRPS
401 RTDIVSPPVC GNELLEVGEE CDCGSPANCR NPCCDAASCR LHSWVQCESG ECCQQCRFKR AGTECRPAMD ECDMAEQCTG
481 KSADCPVDGF HANGRPCLHN LGYCYNGKCP ITLYQCRALL GKNVTGVQDS CFQHNRLGYS YGYCRKENGR KIPCAPEDEK
561 CGRLYCSYNL LGNHIPCYPY YRRNREDKGM VDHGTKCGDG KVCSNRHCVD VTTAY

Supporting Peptides:

Peptide	Uniq	-10lgP	Mass	Length	ppm	m/z	z	RT	Fraction	Scan	Source File	Area F1	Area F2	A
K.LVIVVDHSMVTK.Y	N	86.31	1339.7533	12	0.8	670.8845	2	40.72	2	F2:11182	12112021_RID_2349_DP2.raw		6.7627E5	6.[illegible]
A.SQLVATSEQQSYYDR.F	N	85.45	1773.8169	15	0.1	887.9158	2	35.26	3	F3:9651	17112021_RID_2349_DP3.raw			3.[illegible]
R.VNILNEIYGPFSIR.V	Y	80.46	1633.8827	14	0.4	817.9489	2	68.10	3	F3:20508	17112021_RID_2349_DP3.raw	2.1784E5	1.6365E6	7.[illegible]
K.LVIVVDHSM(+15.99)VTK.Y	N	80.36	1355.7483	12	0.3	678.8816	2	40.62	2	F2:11272	12112021_RID_2349_DP2.raw		3.9309E5	1.[illegible]
K.YNGDLTAIR.T	N	72.64	1021.5192	9	-0.1	511.7668	2	35.19	1	F1:10334	12112021_RID_2349_DP1.raw	2.7287E6	6.3775E6	1.[illegible]
V.ATSEQQSYYDR.F	N	70.49	1346.5739	11	0.3	674.2944	2	23.13	2	F2:5110	12112021_RID_2349_DP2.raw	1.2012E5	4.4545E5	7.[illegible]
R.NYYQTFLTNYRPD.C	N	66.53	1693.7736	13	0.4	847.8944	2	54.36	2	F2:15683	12112021_RID_2349_DP2.raw	2.3745E5	9.7382E5	7.[illegible]
S.QLVATSEQQSYYDR.F	N	63.01	1686.7849	14	1.1	844.4006	2	47.86	5	F5:13912	17112021_RID_2349_DP5.raw		1.5966E5	2.[illegible]
T.SEQQSYYDR.F	N	62.38	1174.4890	9	0.7	588.2522	2	21.94	2	F2:4785	12112021_RID_2349_DP2.raw	3.2418E4	2.9845E5	2.[illegible]
A.TSEQQSYYDR.F	N	58.61	1275.5367	10	0.4	638.7759	2	22.62	5	F5:5328	17112021_RID_2349_DP5.raw	1.2834E5	4.8542E5	8.[illegible]
R.VNILNEIYGPF.S	Y	48.96	1277.6655	11	0.3	639.8402	2	74.71	3	F3:22527	17112021_RID_2349_DP3.raw			2.[illegible]
A.TSEQQSYYDRFR.Y	N	45.69	1578.7063	12	-0.5	527.2424	3	33.88	1	F1:9791	12112021_RID_2349_DP1.raw	5.1666E6		1.[illegible]
R.NYYQTFLTNY.R	N	45.63	1325.5928	10	0.5	663.8040	2	61.33	4	F4:18230	17112021_RID_2349_DP4.raw			
V.TQTNWESDEPIKK.A	N	44.98	1574.7576	13	0.5	788.3865	2	30.75	1	F1:8710	12112021_RID_2349_DP1.raw	3.3503E5		7.[illegible]
T.SEQQSYYDRFR.Y	N	42.54	1477.6586	11	0.6	739.8370	2	32.22	3	F3:8513	17112021_RID_2349_DP3.raw		1.2544E6	3.[illegible]
V.ATSEQQSYYDRFR.Y	N	41.91	1649.7434	13	0.5	550.9220	3	32.84	3	F3:8815	17112021_RID_2349_DP3.raw	1.9978E6		5.[illegible]
R.NYYQTFLTN.Y	N	40.03	1162.5295	9	0.5	582.2723	2	54.02	4	F4:15903	17112021_RID_2349_DP4.raw			
													total	

TRINITY_DN30
back to list

| Protein Coverage | Supporting Peptides |
Protein Coverage:

1 MMQVLLVTIC LAVFPYQGSS IILESGNVND YEVVYPQKVT ALPKGAIQQL EQKYEDAIQY QFKVKGEPVV LHLEKNKDFF
81 PENYSETHYS PDDREITTNP PVEDHCYYHG HIQNEADSTA SISACNGLKG HFKLQGETYL IEPLKIPDSE AHAVYKYENI
161 EKEDEAPKMC GV**TQTNWESD EPIKKASQLV ATSEQQSYYD RFR**YIK**LVIV VDHSMVTKYN GDLTAIR**TKI YQR**VNILNEI**
241 **YGPFSIR**VPL VGVEIWVNRD FISVTSASNV TLKSFGEWRK RDLLSRKRHD YAQLLTAINF DEQIVGRAYI STMCQPLRSV
321 AIVEDYSPID HAVAATMAHE MGHNLGMHHD GKQCNCGANS CIMAPEISDP PPTYFSDCSR **NYYQTFLTNY RPD**CTLIRPS
401 RTDIVSPPVC GNELLEVGEE CDCGSPANCR NPCCDAASCR LHSWVQCESG ECCQQCRFKR AGTECRPAMD ECDMAEQCTG
481 KSADCPVDGF HANGRPCLHN LGYCYNGKCP ITLYQCRALL GKNVTGVQDS CFQHNRLGYS YGYCRKENGR KIPCAPEDEK
561 CGRLYCSYNL LGNHIPCYPY YRRNREDKGM VDHGTKCGDG KVCSNRHCVD VTTAY

Supporting Peptides:

Peptide	Uniq	-10lgP	Mass	Length	ppm	m/z	z	RT	Fraction	Scan	Source File	Area F1	Area F2	A
K.LVIVVDHSMVTK.Y	N	86.31	1339.7533	12	0.8	670.8845	2	40.72	2	F2:11182	12112021_RID_2349_DP2.raw		6.7627E5	6.[illegible]
A.SQLVATSEQQSYYDR.F	N	85.45	1773.8169	15	0.1	887.9158	2	35.26	3	F3:9651	17112021_RID_2349_DP3.raw			3.[illegible]
R.VNILNEIYGPFSIR.V	Y	80.46	1633.8827	14	0.4	817.9489	2	68.10	3	F3:20508	17112021_RID_2349_DP3.raw	2.1784E5	1.6365E6	7.[illegible]
K.LVIVVDHSM(+15.99)VTK.Y	N	80.36	1355.7483	12	0.3	678.8816	2	40.62	2	F2:11272	12112021_RID_2349_DP2.raw		3.9309E5	1.[illegible]
K.YNGDLTAIR.T	N	72.64	1021.5192	9	-0.1	511.7668	2	35.19	1	F1:10334	12112021_RID_2349_DP1.raw	2.7287E6	6.3775E6	1.[illegible]
V.ATSEQQSYYDR.F	N	70.49	1346.5739	11	0.3	674.2944	2	23.13	2	F2:5110	12112021_RID_2349_DP2.raw	1.2012E5	4.4545E5	7.[illegible]
R.NYYQTFLTNYRPD.C	N	66.53	1693.7736	13	0.4	847.8944	2	54.36	2	F2:15683	12112021_RID_2349_DP2.raw	2.3745E5	9.7382E5	7.[illegible]
S.QLVATSEQQSYYDR.F	N	63.01	1686.7849	14	1.1	844.4006	2	47.86	5	F5:13912	17112021_RID_2349_DP5.raw		1.5966E5	2.[illegible]
T.SEQQSYYDR.F	N	62.38	1174.4890	9	0.7	588.2522	2	21.94	2	F2:4785	12112021_RID_2349_DP2.raw	3.2418E4	2.9845E5	2.[illegible]
A.TSEQQSYYDR.F	N	58.61	1275.5367	10	0.4	638.7759	2	22.62	5	F5:5328	17112021_RID_2349_DP5.raw	1.2834E5	4.8542E5	8.[illegible]
R.VNILNEIYGPF.S	Y	48.96	1277.6655	11	0.3	639.8402	2	74.71	3	F3:22527	17112021_RID_2349_DP3.raw			2.[illegible]
A.TSEQQSYYDRFR.Y	N	45.69	1578.7063	12	-0.5	527.2424	3	33.88	1	F1:9791	12112021_RID_2349_DP1.raw	5.1666E6		1.[illegible]
R.NYYQTFLTNY.R	N	45.63	1325.5928	10	0.5	663.8040	2	61.33	4	F4:18230	17112021_RID_2349_DP4.raw			
V.TQTNWESDEPIKK.A	N	44.98	1574.7576	13	0.5	788.3865	2	30.75	1	F1:8710	12112021_RID_2349_DP1.raw	3.3503E5		7.[illegible]
T.SEQQSYYDRFR.Y	N	42.54	1477.6586	11	0.6	739.8370	2	32.22	3	F3:8513	17112021_RID_2349_DP3.raw		1.2544E6	3.[illegible]
V.ATSEQQSYYDRFR.Y	N	41.91	1649.7434	13	0.5	550.9220	3	32.84	3	F3:8815	17112021_RID_2349_DP3.raw	1.9978E6		5.[illegible]
R.NYYQTFLTN.Y	N	40.03	1162.5295	9	0.5	582.2723	2	54.02	4	F4:15903	17112021_RID_2349_DP4.raw			
														**total**

BAN82155.1
back to list

| Protein Coverage | Supporting Peptides |
Protein Coverage:

```
1   MIRFGNPSSS VKRRQRCRSW YWGGLLLLWA VAETRADIHY ATVYWLEAEK SFQIKAVLDK NGDAYGYYND TIQSTGWGIL
81  EIKAGYGNQP ISNEILMYAA GFLEGYLTAS HMSDHFANLF PLMIKNVIIE QKVKDFIQKQ DEWTRQQIKN NKDDPFWRNA
161 GYVIVQLDGL YMGNVEWAKQ QKRTPLTNFE ISFLNVIGDL LDLIPALYSE LRKSDFRSMP DVSRIYQWDM GHCSALIKVL
241 PGYENIYFAH SSWFTYAATL RIYKHLDFKI TDPQTKTGRA SFSSYPGFLS SLDDFYILGS GLIMLQTTNS VFNLSLLKKV
321 VPESLFAWER VRIANMMADS GKTWAETFEK ENSGTYNNQY MILDTKKIKL QRSLEDGTLY IIEQIPKLVK YSDQTKVLRN
401 GYWPSYNIPF DKVIYNMSGY REYVQRHGLE FSYEMAPRAK IFRRDQGKVT DMESMKSIMR YNNYKEDPYA KRNPCNTICC
481 RQDLDRRTPV PAGCYDSKVA DISMASKFTA YAINGPPVEK GLPVFSWVHF NKTKHQGLPE SYNFDFVTMK PVL
```

Supporting Peptides:

Peptide	Uniq	-10lgP	Mass	Length	ppm	m/z	z	RT	Fraction	Scan	Source File	Area F1	A
R.SLEDGTLYIIEQIPK.L	N	90.40	1717.9138	15	0.5	859.9646	2	67.04	2	F2:19818	12112021_RID_2349_DP2.raw	1.408E6	2.
K.ENSGTYNNQYMILDTK.K	N	89.12	1889.8466	16	0.8	945.9313	2	45.52	2	F2:12882	12112021_RID_2349_DP2.raw		1.
R.IANMM(+15.99)ADSGK.T	N	76.74	1052.4630	10	-0.3	527.2386	2	24.17	2	F2:6829	12112021_RID_2349_DP2.raw	9.0092E4	1.
R.IANM(+15.99)MADSGK.T	N	75.38	1052.4630	10	-0.3	527.2386	2	24.17	2	F2:6725	12112021_RID_2349_DP2.raw		1.
R.DQGKVTDMESMK.S	N	72.75	1367.6061	12	0.5	684.8107	2	27.78	2	F2:6747	12112021_RID_2349_DP2.raw		2.
H.YATVYWLEAEK.S	N	72.57	1371.6710	11	0.7	686.8433	2	51.85	5	F5:15257	17112021_RID_2349_DP5.raw		
K.HQGLPESYNFDFVTM(+15.99)KPVL	N	71.29	2237.0828	19	0.8	746.7021	3	59.87	2	F2:17492	12112021_RID_2349_DP2.raw		1.
K.HQGLPESYNFDFVTMKPVL	N	70.10	2221.0876	19	0.3	1111.5514	2	65.61	2	F2:19349	12112021_RID_2349_DP2.raw		1.
K.ENSGTYNNQYM(+15.99)ILDTK.K	N	68.81	1905.8414	16	0.6	953.9286	2	41.00	2	F2:11307	12112021_RID_2349_DP2.raw		9.
R.DQGKVTDMESM(+15.99)K.S	N	66.01	1383.6010	12	0.7	692.8082	2	27.71	2	F2:6798	12112021_RID_2349_DP2.raw		1.
K.YSDQTKVLR.N	N	60.88	1108.5876	9	-0.4	555.3009	2	23.23	1	F1:6160	12112021_RID_2349_DP1.raw	4.0413E5	
R.DQGKVTDM(+15.99)ESM(+15.99)K.S	N	60.55	1399.5959	12	-0.3	700.8051	2	27.86	2	F2:6821	12112021_RID_2349_DP2.raw		7.
R.DQGKVTDM(+15.99)ESMK.S	N	55.19	1383.6010	12	0.0	692.8077	2	19.49	2	F2:3974	12112021_RID_2349_DP2.raw		2.
K.ENSGTYNNQYMILDTKK.I	N	48.36	2017.9415	17	0.5	673.6548	3	39.15	2	F2:10720	12112021_RID_2349_DP2.raw		2.
D.GTLYIIEQIPK.L	N	47.53	1273.7281	11	0.8	637.8719	2	53.61	4	F4:15815	17112021_RID_2349_DP4.raw		
K.ENSGTYNNQYM(+15.99)ILDTKK.I	N	47.15	2033.9364	17	0.0	678.9861	3	39.23	2	F2:10730	12112021_RID_2349_DP2.raw		1.
K.VLPGYENIYFAH.S	N	46.58	1421.6979	12	0.6	711.8566	2	53.52	2	F2:15534	12112021_RID_2349_DP2.raw		1.
K.VTDM(+15.99)ESMK.S	N	45.53	955.3990	8	-0.3	478.7067	2	24.77	2	F2:5754	12112021_RID_2349_DP2.raw		1.
K.HQGLPESYNFD.F	N	44.27	1305.5625	11	-0.5	653.7882	2	42.77	2	F2:11932	12112021_RID_2349_DP2.raw		1.
K.VADISM(+15.99)ASK.F	Y	43.92	936.4586	9	-0.6	469.2363	2	29.56	2	F2:7390	12112021_RID_2349_DP2.raw		3.
K.VTDMESM(+15.99)K.S	N	43.61	955.3990	8	-0.3	478.7067	2	24.77	2	F2:5660	12112021_RID_2349_DP2.raw		1.
K.VTDM(+15.99)ESM(+15.99)K.S	N	42.57	971.3940	8	-0.2	486.7042	2	24.80	2	F2:5663	12112021_RID_2349_DP2.raw		8.
K.NNKDDPFWR.N	N	41.45	1190.5469	9	0.5	596.2810	2	35.13	5	F5:9629	17112021_RID_2349_DP5.raw		
K.VTDMESMK.S	N	39.74	939.4042	8	0.0	470.7094	2	24.77	2	F2:5674	12112021_RID_2349_DP2.raw		7.

TRINITY_DN266
back to list

| Protein Coverage | Supporting Peptides |
Protein Coverage:

```
  1 MQTPKRRRGA QGCPRSSLSP PPPLLLLVGA VWFGAALSVA AGSFELTILH TNDVHARVEQ TSRDSGKCTG QDCYGGVARR
 81 ATKIRELRAK HRHVLLLDAG DQYQGTVWFS FFKGREVLKF MKSLRYDAMA LGNHEFDNGL AGLLDPLLKN ANFPILSANI
161 RPKGPIASTI SGYILPYKII KVGSEKVGII GYTTKETPVL SNPGPYLEFR DEVEELQIHA NKLTTLGVNK IIALGHSGFF
241 EDQRIAQKVK GVDVVVGGHT NTFLYTGMLF LHKIGFLYNL AIRVPSILPT T
```

Supporting Peptides:

Peptide	Uniq	-10lgP	Mass	Length	ppm	m/z	z	RT	Fraction	Scan	Source File	Area F1	Area F2	Area I
K.IIALGHSGFFEDQR.I	N	93.42	1588.7997	14	0.8	795.4078	2	47.13	2	F2:13392	12112021_RID_2349_DP2.raw	1.2282E6	4.8241E6	2.973E
K.NANFPILSANIRPK.G	N	87.65	1553.8678	14	0.6	777.9417	2	47.24	1	F1:14300	12112021_RID_2349_DP1.raw	2.5082E7	3.0757E6	
K.GPIASTISGYILPYK.I	Y	85.78	1578.8657	15	0.7	790.4407	2	58.67	2	F2:17142	12112021_RID_2349_DP2.raw	4.5074E6	4.1083E6	2.5052I
K.VGSEKVGIIGYTTK.E	N	74.20	1450.8031	14	0.9	726.4095	2	36.48	1	F1:10777	12112021_RID_2349_DP1.raw	2.8559E6		
R.DEVEELQIHANK.L	N	70.10	1423.6943	12	0.6	712.8549	2	34.19	4	F4:9279	17112021_RID_2349_DP4.raw		2.6545E4	
K.ETPVLSNPGPYLEFR.D	N	63.99	1717.8674	15	0.3	859.9412	2	55.85	4	F4:16529	17112021_RID_2349_DP4.raw			
K.VGIIGYTTK.E	N	60.94	950.5436	9	-0.2	476.2790	2	37.93	1	F1:11149	12112021_RID_2349_DP1.raw	9.0552E6	3.6938E6	2.4626I
R.YDAMALGNHEFD.N	N	53.56	1381.5608	12	1.0	691.7883	2	46.21	7	F7:13460	17112021_RID_2349_DP7.raw			
K.LTTLGVNK.I	N	48.37	844.5018	8	-0.2	423.2581	2	28.18	2	F2:6923	12112021_RID_2349_DP2.raw	1.9323E6	3.4738E6	2.4241I

total 9 pepti

TRINITY_DN240
back to list

| Protein Coverage | Supporting Peptides |
Protein Coverage:

```
 1 RLDRPVTNST HIAPLSLPSS PPIVGSVCRI MGWGTISTTK VILSDVPRCA NIEILKYSKC QEVHPELPAK GRVVCAGVWQ
81 GGKDTCKGDS GGPLICNGQF QGLLSWGGDP CAQPLQPGLY TDIFDYSDWI KSIIAGNTAV TCPP
```

Supporting Peptides:

Peptide	Uniq	-10lgP	Mass	Length	ppm	m/z	z	RT	Fraction	Scan	Source File	Area F1	A
R.IMGWGTISTTKVILSDVPR.C	Y	96.96	2073.1292	19	0.2	692.0505	3	59.98	3	F3:17898	17112021_RID_2349_DP3.raw		
R.IMGWGTISTTK.V	N	74.23	1193.6115	11	0.6	597.8134	2	46.31	2	F2:13130	12112021_RID_2349_DP2.raw	1.0272E6	8
G.LYTDIFDYSDWIK.S	N	70.95	1677.7926	13	1.1	839.9045	2	72.40	3	F3:21807	17112021_RID_2349_DP3.raw		
P.GLYTDIFDYSDWIK.S	N	70.77	1734.8141	14	0.1	868.4144	2	74.37	3	F3:22479	17112021_RID_2349_DP3.raw		
L.YTDIFDYSDWIK.S	N	69.44	1564.7085	12	0.3	783.3618	2	68.09	3	F3:20399	17112021_RID_2349_DP3.raw		
Y.TDIFDYSDWIK.S	N	66.08	1401.6453	11	0.8	701.8304	2	66.55	4	F4:19959	17112021_RID_2349_DP4.raw		
R.IM(+15.99)GWGTISTTKVILSDVPR.C	Y	65.54	2089.1240	19	0.7	697.3824	3	56.07	3	F3:16589	17112021_RID_2349_DP3.raw		
R.IM(+15.99)GWGTISTTK.V	N	65.43	1209.6063	11	0.2	605.8105	2	41.63	3	F3:12035	17112021_RID_2349_DP3.raw		4
K.SIIAGNTAVTC(+57.02)PP	N	58.39	1299.6493	13	0.5	650.8323	2	45.43	3	F3:13053	17112021_RID_2349_DP3.raw		
K.VILSDVPR.C	Y	53.60	897.5283	8	0.4	449.7716	2	36.96	1	F1:10936	12112021_RID_2349_DP1.raw	2.2296E6	1
L.QPGLYTDIFDYSDWIK.S	N	51.31	1959.9254	16	0.7	980.9707	2	73.83	3	F3:22207	17112021_RID_2349_DP3.raw		

Peptide	Uniq	-10lgP	Mass	Length	ppm	m/z	z	RT	Fraction	Scan	Source File	Area F1	A
K.SIIAGNTAVT.C	N	48.53	945.5131	10	0.0	473.7639	2	39.62	3	F3:11085	17112021_RID_2349_DP3.raw		
K.C(+57.02)QEVHPELPAK.G	Y	46.74	1306.6339	11	0.6	654.3246	2	27.17	3	F3:6836	17112021_RID_2349_DP3.raw		
M.GWGTISTTK.V	N	46.73	949.4869	9	-0.6	475.7504	2	32.18	3	F3:8600	17112021_RID_2349_DP3.raw		
T.KVILSDVPR.C	Y	42.92	1025.6233	9	0.3	513.8191	2	30.86	3	F3:8089	17112021_RID_2349_DP3.raw		
C.AQPLQPGLYTDIFDYSDWIK.S	N	41.95	2369.1580	20	0.7	1185.5870	2	75.98	3	F3:23000	17112021_RID_2349_DP3.raw		
C.QEVHPELPAK.G	Y	38.96	1146.6033	10	0.2	574.3090	2	26.03	3	F3:6524	17112021_RID_2349_DP3.raw		

TRINITY_DN59
back to list

| Protein Coverage | Supporting Peptides |
Protein Coverage:

1 MGRFISVSFG WLVVFLSPSG TRADCPSDWS SHEGNCYKVF NLRKTWEEAE KFCTKQANGV HLVSIDSKKE ANFVAKLVSQ

81 NIKESRGIEF VWIGLRDQSE RQHCSSEWSD GSSINYQNWI EAESRKCLGL EKETGYRKWV NIYCGLRNPF TCEI

Supporting Peptides:

Peptide	Uniq	-10lgP	Mass	Length	ppm	m/z	z	RT	Fraction	Scan	Source File	Area F1	Area F2	Area F3	A
K.QANGVHLVSIDSK.K	Y	81.82	1366.7205	13	0.6	456.5811	3	32.64	5	F5:8755	17112021_RID_2349_DP5.raw				
K.QANGVHLVSIDSKK.E	Y	77.07	1494.8154	14	0.1	499.2791	3	28.38	5	F5:7341	17112021_RID_2349_DP5.raw				
D.GSSINYQNWIEAESR.K	Y	62.77	1752.8066	15	0.2	877.4108	2	50.91	6	F6:14917	17112021_RID_2349_DP6.raw				
S.SINYQNWIEAESR.K	Y	61.57	1608.7532	13	0.1	805.3839	2	51.12	6	F6:14982	17112021_RID_2349_DP6.raw				
K.LVSQNIKESR.G	Y	59.45	1172.6514	10	0.9	587.3335	2	20.11	1	F1:5091	12112021_RID_2349_DP1.raw	2.9622E6			
N.GVHLVSIDSK.K	Y	56.37	1053.5818	10	-0.5	527.7979	2	31.63	6	F6:8277	17112021_RID_2349_DP6.raw				
N.YQNWIEAESR.K	Y	55.48	1294.5942	10	0.8	648.3049	2	40.44	6	F6:11179	17112021_RID_2349_DP6.raw				
W.SDGSSINYQNWIEAESR.K	Y	51.52	1954.8657	17	0.3	978.4404	2	52.50	6	F6:15474	17112021_RID_2349_DP6.raw				
G.SSINYQNWIEAESR.K	Y	51.20	1695.7853	14	0.8	848.9006	2	50.80	6	F6:14944	17112021_RID_2349_DP6.raw				
Y.QNWIEAESR.K	Y	48.46	1131.5309	9	-0.5	566.7725	2	35.70	6	F6:9679	17112021_RID_2349_DP6.raw				
K.TWEEAEKF.C	N	47.94	1038.4658	8	0.3	520.2404	2	39.87	6	F6:11136	17112021_RID_2349_DP6.raw				
N.GVHLVSIDSKK.E	Y	47.32	1181.6768	11	-0.8	394.8992	3	27.85	6	F6:6922	17112021_RID_2349_DP6.raw				
A.NGVHLVSIDSK.K	Y	47.22	1167.6248	11	-0.5	584.8193	2	32.17	6	F6:8523	17112021_RID_2349_DP6.raw				
K.EANFVAKLVSQN.I	Y	45.69	1318.6881	12	0.1	660.3514	2	48.74	6	F6:14195	17112021_RID_2349_DP6.raw				
R.KTWEEAEK.F	N	45.16	1019.4923	8	0.3	510.7536	2	17.57	6	F6:3741	17112021_RID_2349_DP6.raw				
K.QANGVHLVSID.S	Y	43.90	1151.5935	11	0.4	576.8043	2	40.57	6	F6:11384	17112021_RID_2349_DP6.raw				
K.KEANFVAK.L	Y	42.38	905.4970	8	-0.4	453.7556	2	16.96	6	F6:3318	17112021_RID_2349_DP6.raw				
S.DGSSINYQNWIEAESR.K	Y	42.17	1867.8336	16	0.4	934.9245	2	54.93	6	F6:16268	17112021_RID_2349_DP6.raw				
K.EANFVAK.L	Y	41.77	777.4021	7	0.2	389.7084	2	22.39	6	F6:5158	17112021_RID_2349_DP6.raw				
V.HLVSIDSK.K	N	41.41	897.4919	8	-0.6	449.7530	2	23.13	6	F6:5416	17112021_RID_2349_DP6.raw				
S.INYQNWIEAESR.K	Y	39.32	1521.7212	12	0.5	761.8683	2	49.89	6	F6:14631	17112021_RID_2349_DP6.raw				

total 21 peptid

TRINITY_DN59
back to list

| Protein Coverage | Supporting Peptides |
Protein Coverage:

1 MGRFISVSFG WLVVFLSLSG TGADCPSDWS SHEGNCYKVF NLRKTWEEAE KFCTKQANGV HLVSIDSKKE ANFVAKLVSQ

81 NIKESRGIEF VWIGLRDQSE RQHCSSEWSD GSSINYQNWI EAESRKCLGL EKETGYRKWV NIYCGLRNPF TCEI

Supporting Peptides:

Peptide	Uniq	-10lgP	Mass	Length	ppm	m/z	z	RT	Fraction	Scan	Source File	Area F1	Area F2	Area F3	A 1
K.QANGVHLVSIDSK.K	Y	81.82	1366.7205	13	0.6	456.5811	3	32.64	5	F5:8755	17112021_RID_2349_DP5.raw				
K.QANGVHLVSIDSKK.E	Y	77.07	1494.8154	14	0.1	499.2791	3	28.38	5	F5:7341	17112021_RID_2349_DP5.raw				
D.GSSINYQNWIEAESR.K	Y	62.77	1752.8066	15	0.2	877.4108	2	50.91	6	F6:14917	17112021_RID_2349_DP6.raw				
S.SINYQNWIEAESR.K	Y	61.57	1608.7532	13	0.1	805.3839	2	51.12	6	F6:14982	17112021_RID_2349_DP6.raw				
K.LVSQNIKESR.G	Y	59.45	1172.6514	10	0.9	587.3335	2	20.11	1	F1:5091	12112021_RID_2349_DP1.raw	2.9622E6			
N.GVHLVSIDSK.K	Y	56.37	1053.5818	10	-0.5	527.7979	2	31.63	6	F6:8277	17112021_RID_2349_DP6.raw				
N.YQNWIEAESR.K	Y	55.48	1294.5942	10	0.8	648.3049	2	40.44	6	F6:11179	17112021_RID_2349_DP6.raw				
W.SDGSSINYQNWIEAESR.K	Y	51.52	1954.8657	17	0.3	978.4404	2	52.50	6	F6:15474	17112021_RID_2349_DP6.raw				
G.SSINYQNWIEAESR.K	Y	51.20	1695.7853	14	0.8	848.9006	2	50.80	6	F6:14944	17112021_RID_2349_DP6.raw				
Y.QNWIEAESR.K	Y	48.46	1131.5309	9	-0.5	566.7725	2	35.70	6	F6:9679	17112021_RID_2349_DP6.raw				
K.TWEEAEKF.C	N	47.94	1038.4658	8	0.3	520.2404	2	39.87	6	F6:11136	17112021_RID_2349_DP6.raw				
N.GVHLVSIDSKK.E	Y	47.32	1181.6768	11	-0.8	394.8992	3	27.85	6	F6:6922	17112021_RID_2349_DP6.raw				
A.NGVHLVSIDSK.K	Y	47.22	1167.6248	11	-0.5	584.8193	2	32.17	6	F6:8523	17112021_RID_2349_DP6.raw				
K.EANFVAKLVSQN.I	Y	45.69	1318.6881	12	0.1	660.3514	2	48.74	6	F6:14195	17112021_RID_2349_DP6.raw				
R.KTWEEAEK.F	N	45.16	1019.4923	8	0.3	510.7536	2	17.57	6	F6:3741	17112021_RID_2349_DP6.raw				
K.QANGVHLVSID.S	Y	43.90	1151.5935	11	0.4	576.8043	2	40.57	6	F6:11384	17112021_RID_2349_DP6.raw				
K.KEANFVAK.L	Y	42.38	905.4970	8	-0.4	453.7556	2	16.96	6	F6:3318	17112021_RID_2349_DP6.raw				
S.DGSSINYQNWIEAESR.K	Y	42.17	1867.8336	16	0.4	934.9245	2	54.93	6	F6:16268	17112021_RID_2349_DP6.raw				
K.EANFVAK.L	Y	41.77	777.4021	7	0.2	389.7084	2	22.39	6	F6:5158	17112021_RID_2349_DP6.raw				
V.HLVSIDSK.K	N	41.41	897.4919	8	-0.6	449.7530	2	23.13	6	F6:5416	17112021_RID_2349_DP6.raw				
S.INYQNWIEAESR.K	Y	39.32	1521.7212	12	0.5	761.8683	2	49.89	6	F6:14631	17112021_RID_2349_DP6.raw				

total 21 peptide

TRINITY_DN59
back to list

| Protein Coverage | Supporting Peptides |
Protein Coverage:

1 MGRFISVSFG WLVVFLSPSG TRADCPSDWS SHEGNCYKVF NLRKTWEEAE KFCTKQANGV HLVSIDSKKE ANFVAKLVSQ

81 NIKESRGIEF VWIGLRDQSE RQHCSSEWSD GSSINYQNWI EAESRKCLGL EKETGYRKWV NIYCGLRNPF TCEI

Supporting Peptides:

Peptide	Uniq	-10lgP	Mass	Length	ppm	m/z	z	RT	Fraction	Scan	Source File	Area F1	Area F2	Area F3	A l
K.QANGVHLVSIDSK.K	Y	81.82	1366.7205	13	0.6	456.5811	3	32.64	5	F5:8755	17112021_RID_2349_DP5.raw				
K.QANGVHLVSIDSKK.E	Y	77.07	1494.8154	14	0.1	499.2791	3	28.38	5	F5:7341	17112021_RID_2349_DP5.raw				
D.GSSINYQNWIEAESR.K	Y	62.77	1752.8066	15	0.2	877.4108	2	50.91	6	F6:14917	17112021_RID_2349_DP6.raw				
S.SINYQNWIEAESR.K	Y	61.57	1608.7532	13	0.1	805.3839	2	51.12	6	F6:14982	17112021_RID_2349_DP6.raw				
K.LVSQNIKESR.G	Y	59.45	1172.6514	10	0.9	587.3335	2	20.11	1	F1:5091	12112021_RID_2349_DP1.raw	2.9622E6			
N.GVHLVSIDSK.K	Y	56.37	1053.5818	10	-0.5	527.7979	2	31.63	6	F6:8277	17112021_RID_2349_DP6.raw				
N.YQNWIEAESR.K	Y	55.48	1294.5942	10	0.8	648.3049	2	40.44	6	F6:11179	17112021_RID_2349_DP6.raw				
W.SDGSSINYQNWIEAESR.K	Y	51.52	1954.8657	17	0.3	978.4404	2	52.50	6	F6:15474	17112021_RID_2349_DP6.raw				
G.SSINYQNWIEAESR.K	Y	51.20	1695.7853	14	0.8	848.9006	2	50.80	6	F6:14944	17112021_RID_2349_DP6.raw				
Y.QNWIEAESR.K	Y	48.46	1131.5309	9	-0.5	566.7725	2	35.70	6	F6:9679	17112021_RID_2349_DP6.raw				
K.TWEEAEKF.C	N	47.94	1038.4658	8	0.3	520.2404	2	39.87	6	F6:11136	17112021_RID_2349_DP6.raw				
N.GVHLVSIDSKK.E	Y	47.32	1181.6768	11	-0.8	394.8992	3	27.85	6	F6:6922	17112021_RID_2349_DP6.raw				
A.NGVHLVSIDSK.K	Y	47.22	1167.6248	11	-0.5	584.8193	2	32.17	6	F6:8523	17112021_RID_2349_DP6.raw				
K.EANFVAKLVSQN.I	Y	45.69	1318.6881	12	0.1	660.3514	2	48.74	6	F6:14195	17112021_RID_2349_DP6.raw				
R.KTWEEAEK.F	N	45.16	1019.4923	8	0.3	510.7536	2	17.57	6	F6:3741	17112021_RID_2349_DP6.raw				
K.QANGVHLVSID.S	Y	43.90	1151.5935	11	0.4	576.8043	2	40.57	6	F6:11384	17112021_RID_2349_DP6.raw				
K.KEANFVAK.L	Y	42.38	905.4970	8	-0.4	453.7556	2	16.96	6	F6:3318	17112021_RID_2349_DP6.raw				
S.DGSSINYQNWIEAESR.K	Y	42.17	1867.8336	16	0.4	934.9245	2	54.93	6	F6:16268	17112021_RID_2349_DP6.raw				
K.EANFVAK.L	Y	41.77	777.4021	7	0.2	389.7084	2	22.39	6	F6:5158	17112021_RID_2349_DP6.raw				
V.HLVSIDSK.K	N	41.41	897.4919	8	-0.6	449.7530	2	23.13	6	F6:5416	17112021_RID_2349_DP6.raw				
S.INYQNWIEAESR.K	Y	39.32	1521.7212	12	0.5	761.8683	2	49.89	6	F6:14631	17112021_RID_2349_DP6.raw				

total 21 peptide

TRINITY_DN1385
back to list

| Protein Coverage | Supporting Peptides |
Protein Coverage:

1 SQLVATSEQQ SYYDRFRYIK LAIVADHSMV EKYKGNLKKL RKRIYRTVNT VNEMYLPLNI RVPLVHVEIW DDRDLINVTS C

81 SGGATLKSFG KWRGSDLLSR KRHDNAQLLT GIDLNGQTLG IAPNSGMCHP NRSV

Supporting Peptides:

Peptide	Uniq	-10lgP	Mass	Length	ppm	m/z	z	RT	Fraction	Scan	Source File	Area F1	Area F
K.LAIVADHSMVEK.Y	Y	88.02	1311.6857	12	0.2	656.8502	2	34.96	2	F2:9288	12112021_RID_2349_DP2.raw	4.5317E6	5.8499E

total

Peptide	Uniq	-10lgP	Mass	Length	ppm	m/z	z	RT	Fraction	Scan	Source File	Area F1	Area F
SQLVATSEQQSYYDR.F	N	85.45	1773.8169	15	0.1	887.9158	2	35.26	3	F3:9651	17112021_RID_2349_DP3.raw		
K.LAIVADHSM(+15.99)VEK.Y	Y	78.52	1327.6805	12	0.4	664.8478	2	35.33	1	F1:10345	12112021_RID_2349_DP1.raw	2.4019E6	2.9689E
R.TVNTVNEM(+15.99)YLPLNIR.V	Y	72.02	1791.9189	15	0.5	896.9672	2	56.15	2	F2:16974	12112021_RID_2349_DP2.raw	7.951E6	6.1112E
V.ATSEQQSYYDR.F	N	70.49	1346.5739	11	0.3	674.2944	2	23.13	2	F2:5110	12112021_RID_2349_DP2.raw	1.2012E5	4.4545E
R.TVNTVNEMYLPLNIR.V	Y	66.01	1775.9240	15	0.2	888.9694	2	57.79	4	F4:17087	17112021_RID_2349_DP4.raw	8.7541E6	9.8614E
S.QLVATSEQQSYYDR.F	N	63.01	1686.7849	14	1.1	844.4006	2	47.86	5	F5:13912	17112021_RID_2349_DP5.raw		1.5966E
T.SEQQSYYDR.F	N	62.38	1174.4890	9	0.7	588.2522	2	21.94	2	F2:4785	12112021_RID_2349_DP2.raw	3.2418E4	2.9845E
A.TSEQQSYYDR.F	N	58.61	1275.5367	10	0.4	638.7759	2	22.62	5	F5:5328	17112021_RID_2349_DP5.raw	1.2834E5	4.8542E
K.WRGSDLLSR.K	Y	50.67	1088.5726	9	-0.1	545.2935	2	37.47	1	F1:11043	12112021_RID_2349_DP1.raw	4.0422E6	
A.TSEQQSYYDRFR.Y	N	45.69	1578.7063	12	-0.5	527.2424	3	33.88	1	F1:9791	12112021_RID_2349_DP1.raw	5.1666E6	
T.SEQQSYYDRFR.Y	N	42.54	1477.6586	11	0.6	739.8370	2	32.22	3	F3:8513	17112021_RID_2349_DP3.raw		1.2544E
V.ATSEQQSYYDRFR.Y	N	41.91	1649.7434	13	0.5	550.9220	3	32.84	3	F3:8815	17112021_RID_2349_DP3.raw	1.9978E6	

total

QHR82712.1
back to list

| Protein Coverage | Supporting Peptides |
Protein Coverage:

1 ALGNHEFDNG LDGLLDPLLK **NANFPILSAN IRPK**GPIASN ISGYILPYKI IN**VGSEKVGI IGYTTKETPV LSNPGPYLEF**

81 **RDEVEELQIH ANK**LTTLGIN KIIALGHSGF LEDQRIAQKV KGVDVVVGGH TNTFLYSGSP PSTEVPAGNY PFMVQSDDGR

161 **QVPVVQAYAF GKYLGYLNVV FDDKGNVIKA SGNPILLNKD** IPEDQDLKAE VNKMKIQLQS YSSQEIGKTI VYLNGTTQAC

241 RFQECNLGNL ICDAVIYNNL RHPDDNEWNH VSMCIVNGGG IRSPIDERAN NGNKEGPF

Supporting Peptides:

Peptide	Uniq	-10lgP	Mass	Length	ppm	m/z	z	RT	Fraction	Scan	Source File	Area F1	Area F2	A
K.NANFPILSANIRPK.G	N	87.65	1553.8678	14	0.6	777.9417	2	47.24	1	F1:14300	12112021_RID_2349_DP1.raw	2.5082E7	3.0757E6	
R.QVPVVQAYAFGK.Y	N	78.42	1305.7081	12	0.2	653.8615	2	46.67	2	F2:13272	12112021_RID_2349_DP2.raw	4.2275E6	4.4501E6	1.
N.VGSEKVGIIGYTTK.E	N	74.20	1450.8031	14	0.9	726.4095	2	36.48	1	F1:10777	12112021_RID_2349_DP1.raw	2.8559E6		
K.YLGYLNVVFDDKGNVIK.A	Y	73.37	1956.0356	17	0.4	979.0255	2	58.81	3	F3:17436	17112021_RID_2349_DP3.raw		6.057E5	4.
R.DEVEELQIHANK.L	N	70.10	1423.6943	12	0.6	712.8549	2	34.19	4	F4:9279	17112021_RID_2349_DP4.raw		2.6545E4	
K.ASGNPILLNK.D	N	67.11	1025.5869	10	-0.1	513.8007	2	34.04	1	F1:9894	12112021_RID_2349_DP1.raw	2.3772E7	7.6734E6	2.
K.ETPVLSNPGPYLEFR.D	N	63.99	1717.8674	15	0.3	859.9412	2	55.85	4	F4:16529	17112021_RID_2349_DP4.raw			
K.VGIIGYTTK.E	N	60.94	950.5436	9	-0.2	476.2790	2	37.93	1	F1:11149	12112021_RID_2349_DP1.raw	9.0552E6	3.6938E6	2.
K.ASGNPILLNKD.I	N	52.23	1140.6139	11	0.6	571.3146	2	34.93	4	F4:9472	17112021_RID_2349_DP4.raw			

total 9 pepti

AFE84762.1
back to list

| Protein Coverage | Supporting Peptides |
Protein Coverage:

1 MARERRDSKA AAFFCLAWAL GLPLLGFPQH VGGREDRADW TQEKYSHRPT ILNATSILQV TSQTNVSR**MW QNDLHPIMIE** C

81 **RYPGSPGSYA VR**QHIKHR**LQ GLQAGWLVEE DTFQSHTPYG YR**TFSNIIST LNPLAKRHLV IACHYDSK**YF PPQLDGK**VFV

161 GATDSAVPCA MMLELARSLD RQLSFLKQSS LPTKADLSLK **LIFFDGEEAF VRWSPSDSLY GSR**SLAQK**MS STPHPPGAR**N

241 TYQTQGIDLF VLLDLIGAR**N PVFPVYFLNT AR**WFGRLEAI EQNLHDLGLL NNYSSERQYF RSNLRQ**HPVE DDHIPFLR**R**G**

321 **VPILHLIPSP FPR**VWHVMED NEENLDKPTI DNLSKILQIF VLEYLNLG

Supporting Peptides:

Peptide	Uniq	-10lgP	Mass	Length	ppm	m/z	z	RT	Fraction	Scan	Source File	Area F1	Area
R.MWQNDLHPIMIER.Y	N	92.13	1681.8069	13	0.1	841.9108	2	49.27	2	F2:14104	12112021_RID_2349_DP2.raw		1.384
R.NPVFPVYFLNTAR.W	N	83.53	1536.8088	13	0.7	769.4122	2	63.32	2	F2:18598	12112021_RID_2349_DP2.raw		7.791
K.LIFFDGEEAFVR.W	N	83.44	1441.7241	12	0.9	721.8700	2	63.41	2	F2:18652	12112021_RID_2349_DP2.raw		4.154
R.WSPSDSLYGSR.S	N	76.49	1253.5676	11	-0.1	627.7910	2	42.62	2	F2:11930	12112021_RID_2349_DP2.raw		4.928
R.YPGSPGSYAVR.Q	N	69.52	1152.5563	11	0.6	577.2858	2	32.51	2	F2:8432	12112021_RID_2349_DP2.raw		8.721
K.M(+15.99)SSTPHPPGAR.N	Y	65.00	1152.5345	11	0.0	577.2745	2	18.26	2	F2:4172	12112021_RID_2349_DP2.raw		9.335
K.MSSTPHPPGAR.N	Y	61.11	1136.5397	11	-0.9	569.2766	2	19.88	5	F5:4334	17112021_RID_2349_DP5.raw		2.139
R.GVPILHLIPSPFPR.V	N	55.94	1541.9082	14	0.5	771.9617	2	63.34	2	F2:18577	12112021_RID_2349_DP2.raw		4.547
Q.HPVEDDHIPFLR.R	N	53.57	1473.7365	12	-0.1	492.2527	3	46.37	2	F2:13060	12112021_RID_2349_DP2.raw		7.548
K.YFPPQLDGK.V	N	51.22	1063.5338	9	0.4	532.7744	2	39.95	2	F2:11061	12112021_RID_2349_DP2.raw		3.278
R.M(+15.99)WQNDLHPIMIER.Y	N	44.14	1697.8018	13	2.2	849.9100	2	47.25	2	F2:13379	12112021_RID_2349_DP2.raw		1.065
R.LQGLQAGWLVEEDTFQSHTPYGYR.T	N	38.86	2794.3350	24	0.8	932.4530	3	60.00	2	F2:17677	12112021_RID_2349_DP2.raw		1.957

total 1

TRINITY_DN554
back to list

| Protein Coverage | Supporting Peptides |
Protein Coverage:

1 LSAGKEGRKT MGRFISVSFS LLVVFLSLSG TEAGFCCPNG WSSFGQHCYK VIEPLKNWTD AEKFCTEQHK **GSHLASIHSS**

81 **EEEAFVSK**VA SKVLKFGSVW IGLNDPWNNC HWEWSDNAK**F DYKAMTR**RPY CTVMVLKPDK IFWFNRGCEK SVSFVCKFLA

Supporting Peptides:

Peptide	Uniq	-10lgP	Mass	Length	ppm	m/z	z	RT	Fraction	Scan	Source File	Area F1	Area F2	Area F3	Area F4	[illegible]
K.GSHLASIHSSEEEAFVSK.V	Y	110.07	1913.9119	18	0.2	957.9634	2	35.00	5	F5:9814	17112021_RID_2349_DP5.raw					2
H.LASIHSSEEEAFVSK.V	Y	75.78	1632.7994	15	0.6	545.2741	3	35.12	5	F5:9694	17112021_RID_2349_DP5.raw					2
A.SIHSSEEEAFVSK.V	Y	74.21	1448.6783	13	0.6	725.3469	2	28.87	5	F5:7463	17112021_RID_2349_DP5.raw					3
S.IHSSEEEAFVSK.V	Y	64.59	1361.6462	12	0.3	681.8306	2	27.01	5	F5:6866	17112021_RID_2349_DP5.raw					1
H.SSEEEAFVSK.V	Y	58.46	1111.5033	10	0.4	556.7592	2	28.18	5	F5:7267	17112021_RID_2349_DP5.raw					7
L.ASIHSSEEEAFVSK.V	Y	54.72	1519.7155	14	0.6	507.5794	3	30.04	5	F5:7917	17112021_RID_2349_DP5.raw					1
S.SEEEAFVSK.V	Y	47.15	1024.4713	9	1.0	513.2435	2	27.17	5	F5:6857	17112021_RID_2349_DP5.raw					2
K.FDYKAMTR.R	Y	46.61	1030.4906	8	0.0	516.2526	2	31.54	5	F5:8441	17112021_RID_2349_DP5.raw					1

total 8 peptides

TRINITY_DN1031
back to list

| Protein Coverage | Supporting Peptides |

Protein Coverage:

```
  1 MEKLILLTWA TGFLVVNCVQ LHQQLQTLPM LGDVYHVTGI LKLPYAEIEE PFEAWFNKTG GKSRIQYYDG QVITYQLGYI  C
 81 KPFGASFKVT PETTETEVNI KECFQFNGTS ENPVRPQSLF PNLTNFQPVR HEHYNGQHCT VLESVSYWGK KKNTYKFWVA
161 DGSRGPLPVH YEMKGYNTLL GSHYDKYEID YISYSHSFSP SIFNLPPGIK CAHWPKGGPE HRILANPMQE FVGGTGEVSQ
241 HHEHLFHHYK KKFRKTYKNK KEIEHRQHNF IHNMRYIHSK NRANLSFKLA ANHLADRTPE ELAVLRGRLQ TDEPRKAGAF
321 PSDSYRGLTL PDSLDWRLYG AVTRVKDQAV CGSCWSFATT GALEGAIFLK TGVLTPLSQQ VLIDCSWGFG NRACDGGQEP
401 QAFEWIMKHG GIASAEAYGP YMGQNGYCHY NQSELIAPIA GYIVITSQNI TDLKAALYKH GPIAVNVDAS HKSFAFYSSG
481 IYYEPNCGNT TMELDHAVLA VGYGDLQGEP YWLIKNSWST YWGNDGYILM SMKENNCGVA SSATYPILA
```

Supporting Peptides:

Peptide	Uniq	-10lgP	Mass	Length	ppm	m/z	z	RT	Fraction	Scan	Source File	Area F1	Area
R.IQYYDGQVITYQLGYIKPFGASFK.V	Y	89.84	2798.4319	24	0.6	933.8185	3	64.33	3	F3:19267	17112021_RID_2349_DP3.raw		
K.VTPETTETEVNIK.E	Y	80.09	1459.7406	13	-0.1	730.8775	2	33.29	3	F3:8992	17112021_RID_2349_DP3.raw		
K.HGPIAVNVDASHK.S	Y	76.92	1343.6946	13	0.3	672.8547	2	28.24	2	F2:6976	12112021_RID_2349_DP2.raw	1.321	
R.GPLPVHYEMK.G	Y	72.68	1169.5903	10	0.8	585.8029	2	35.42	3	F3:9693	17112021_RID_2349_DP3.raw		
R.IQYYDGQVITYQLGYIKPF.G	Y	66.66	2308.1780	19	0.9	1155.0973	2	67.58	3	F3:20288	17112021_RID_2349_DP3.raw		
R.GLTLPDSLDWR.L	Y	63.18	1271.6510	11	0.0	636.8328	2	58.34	2	F2:17069	12112021_RID_2349_DP2.raw	1.203	
R.GPLPVHYEM(+15.99)K.G	Y	57.86	1185.5852	10	-0.2	396.2023	3	29.39	3	F3:7623	17112021_RID_2349_DP3.raw		
R.KAGAFPSDSYR.G	Y	51.41	1197.5778	11	0.2	599.7963	2	27.19	2	F2:6677	12112021_RID_2349_DP2.raw	4.193	
K.FWVADGSR.G	Y	49.35	936.4453	8	0.1	469.2300	2	37.70	3	F3:10542	17112021_RID_2349_DP3.raw		
F.SPSIFNLPPGIK.C	Y	45.13	1268.7128	12	0.8	635.3642	2	55.02	3	F3:16255	17112021_RID_2349_DP3.raw		

total 1

TRINITY_DN1031

back to list

| Protein Coverage | Supporting Peptides |

Protein Coverage:

```
  1 MEKLILLTWA TGFLVVNCVQ LHQQLQTLPM LGDVYHVTGI LKLPYAEIEE PFEAWFNKTG GKSRIQYYDG QVITYQLGYI  C
 81 KPFGASFKVT PETTETEVNI KECFQFNGTS ENPVRPQSLF PNLTNFQPVR HEHYNGQHCT VLESVSYWGK KKNTYKFWVA
161 DGSRGPLPVH YEMKGYNTLL GSHYDKYEID YISYSHSFSP SIFNLPPGIK CAHWPKGGPE HRILANPMQE FVGGTGEVSQ
241 HHEHLFHHYK KKFRKTYKNK KEIEHRQHNF IHNMRYIHSK NRANLSFKLA ANHLADRTPE ELAVLRGRLQ TDEPRKAGAF
321 PSDSYRGLTL PDSLDWRLYG AVTRVKDQAV CGSCWSFATT GALEGAIFLK TGVLTPLSQQ VLIDCSWGFG NRACDGGQEP
401 QAFEWIMKHG GIASAEAYGP YMGQNGYCHY NQSELIAPIA GYIVITSQNI TDLKAALYKH GPIAVNVDAS HKSFAFYSSG
481 IYYEPNCGNT TMELDHAVLA VGYGDLQGEP YWLIKNSWST YWGNDGYILM SMKENNCGVA SSATYPILA
```

Supporting Peptides:

Peptide	Uniq	-10lgP	Mass	Length	ppm	m/z	z	RT	Fraction	Scan	Source File	Area F1	Area
R.IQYYDGQVITYQLGYIKPFGASFK.V	Y	89.84	2798.4319	24	0.6	933.8185	3	64.33	3	F3:19267	17112021_RID_2349_DP3.raw		
K.VTPETTETEVNIK.E	Y	80.09	1459.7406	13	-0.1	730.8775	2	33.29	3	F3:8992	17112021_RID_2349_DP3.raw		
K.HGPIAVNVDASHK.S	Y	76.92	1343.6946	13	0.3	672.8547	2	28.24	2	F2:6976	12112021_RID_2349_DP2.raw	1.321	
R.GPLPVHYEMK.G	Y	72.68	1169.5903	10	0.8	585.8029	2	35.42	3	F3:9693	17112021_RID_2349_DP3.raw		
R.IQYYDGQVITYQLGYIKPF.G	Y	66.66	2308.1780	19	0.9	1155.0973	2	67.58	3	F3:20288	17112021_RID_2349_DP3.raw		
R.GLTLPDSLDWR.L	Y	63.18	1271.6510	11	0.0	636.8328	2	58.34	2	F2:17069	12112021_RID_2349_DP2.raw	1.203	

total 1

Peptide	Uniq	-10lgP	Mass	Length	ppm	m/z	z	RT	Fraction	Scan	Source File	Area F1	Area
R.GPLPVHYEM(+15.99)K.G	Y	57.86	1185.5852	10	-0.2	396.2023	3	29.39	3	F3:7623	17112021_RID_2349_DP3.raw		
R.KAGAFPSDSYR.G	Y	51.41	1197.5778	11	0.2	599.7963	2	27.19	2	F2:6677	12112021_RID_2349_DP2.raw		4.193
K.FWVADGSR.G	Y	49.35	936.4453	8	0.1	469.2300	2	37.70	3	F3:10542	17112021_RID_2349_DP3.raw		
F.SPSIFNLPPGIK.C	Y	45.13	1268.7128	12	0.8	635.3642	2	55.02	3	F3:16255	17112021_RID_2349_DP3.raw		
													**total 1**

TRINITY_DN1031
back to list

| Protein Coverage | Supporting Peptides |
Protein Coverage:

1 MEKLILLTWA TGFLVVNCVQ LHQQLQTLPM LGDVYHVTGI LKLPYAEIEE PFEAWFNKTG GKSR**IQYYDG QVITYQLGYI**

81 **KPFGASFKVT PETTETEVNI K**ECFQFNGTS ENPVRPQSLF PNLTNFQPVR HEHYNGQHCT VLESVSYWGK KKNTYK**FWVA**

161 **DGSRGPLPVH YEMK**GYNTLL GSHYDKYEID YISYSHSF**SP SIFNLPPGIK** CAHWPKGGPE HRILANPMQE FVGGTGEVSQ

241 HHEHLFHHYK KKFRKTYKNK KEIEHRQHNF IHNMRYIHSK NRANLSFKLA ANHLADRTPE ELAVLRGRLQ TDEPR**KAGAF**

321 **PSDSYRGLTL PDSLDWR**LYG AVTRVKDQAV CGSCWSFATT GALEGAIFLK TGVLTPLSQQ VLIDCSWGFG NRACDGGQEP

401 QAFEWIMKHG GIASAEAYGP YMGQNGYCHY NQSELIAPIA GYIVITSQNI TDLKAALYK**H GPIAVNVDAS HK**SFAFYSSG

481 IYYEPNCGNT TMELDHAVLA VGYGDLQGEP YWLIKNSWST YWGNDGYILM SMKENNCGVA SSATYPILA

Supporting Peptides:

Peptide	Uniq	-10lgP	Mass	Length	ppm	m/z	z	RT	Fraction	Scan	Source File	Area F1	Area
R.IQYYDGQVITYQLGYIKPFGASFK.V	Y	89.84	2798.4319	24	0.6	933.8185	3	64.33	3	F3:19267	17112021_RID_2349_DP3.raw		
K.VTPETTETEVNIK.E	Y	80.09	1459.7406	13	-0.1	730.8775	2	33.29	3	F3:8992	17112021_RID_2349_DP3.raw		
K.HGPIAVNVDASHK.S	Y	76.92	1343.6946	13	0.3	672.8547	2	28.24	2	F2:6976	12112021_RID_2349_DP2.raw		1.321
R.GPLPVHYEMK.G	Y	72.68	1169.5903	10	0.8	585.8029	2	35.42	3	F3:9693	17112021_RID_2349_DP3.raw		
R.IQYYDGQVITYQLGYIKPF.G	Y	66.66	2308.1780	19	0.9	1155.0973	2	67.58	3	F3:20288	17112021_RID_2349_DP3.raw		
R.GLTLPDSLDWR.L	Y	63.18	1271.6510	11	0.0	636.8328	2	58.34	2	F2:17069	12112021_RID_2349_DP2.raw		1.203
R.GPLPVHYEM(+15.99)K.G	Y	57.86	1185.5852	10	-0.2	396.2023	3	29.39	3	F3:7623	17112021_RID_2349_DP3.raw		
R.KAGAFPSDSYR.G	Y	51.41	1197.5778	11	0.2	599.7963	2	27.19	2	F2:6677	12112021_RID_2349_DP2.raw		4.193
K.FWVADGSR.G	Y	49.35	936.4453	8	0.1	469.2300	2	37.70	3	F3:10542	17112021_RID_2349_DP3.raw		
F.SPSIFNLPPGIK.C	Y	45.13	1268.7128	12	0.8	635.3642	2	55.02	3	F3:16255	17112021_RID_2349_DP3.raw		
													**total 1**

Q9PT40.1
back to list

| Protein Coverage | Supporting Peptides |
Protein Coverage:

1 MVLIRVLANL LVLQLSYAQK SSELVIGGDE CNINEHPFPV ALHTARSKRF YCA**GTLINQE WVLTAAR**CDR KNIR**IILGVH**

114 147

81 **SKNVPNEDQQ IRVPK**EKFFC LSSK**TYTRWD KDIMLIR**LKK PVNDSTHIVP LSLPSSPPSV GSVCR**IMGWG TITTTKVTYP**

161 **DVPH**CANINM FDYSVCRKVY RKLPEKSRTL CAGILQGGID SCKVDNGGPL ICNGQIQGIV SWGGHPCAQP HKPALYTNVF

241 DYTDWIQSII AGNITATCPP

Supporting Peptides:

Peptide	Uniq	-10lgP	Mass	Length	ppm	m/z	z	RT	Fraction	Scan	Source File	Area F1	Area F2	Are
K.TYTRWDKDIMLIR.L	N	82.41	1709.8923	13	0.4	570.9716	3	49.33	3	F3:14370	17112021_RID_2349_DP3.raw		1.4395E6	1.73
R.IMGWGTITTTK.V	N	78.23	1207.6271	11	-0.4	604.8206	2	47.11	3	F3:13741	17112021_RID_2349_DP3.raw	1.7839E6	2.7374E7	1.67
														**total 1**

Peptide	Uniq	-10lgP	Mass	Length	ppm	m/z	z	RT	Fraction	Scan	Source File	Area F1	Area F2	Are
K.NVPNEDQQIRVPK.E	Y	74.15	1535.8055	13	0.1	768.9101	2	30.88	2	F2:7842	12112021_RID_2349_DP2.raw		1.5351E7	1.45
K.NVPNEDQQIR.V	Y	72.95	1211.5895	10	0.4	606.8022	2	24.54	2	F2:5672	12112021_RID_2349_DP2.raw	3.788E5	1.1029E8	3.95
R.IM(+15.99)GWGTITTTK.V	N	67.99	1223.6221	11	0.3	612.8185	2	40.75	2	F2:11290	12112021_RID_2349_DP2.raw	7.1945E5	1.2257E7	3.31
R.WDKDIMLIR.L	N	59.98	1188.6324	9	0.8	595.3240	2	47.61	2	F2:13483	12112021_RID_2349_DP2.raw		2.0105E7	1.37
N.VPNEDQQIR.V	Y	58.49	1097.5465	9	1.3	549.7812	2	22.54	3	F3:5342	17112021_RID_2349_DP3.raw		1.7116E5	6.42
R.WDKDIM(+15.99)LIR.L	N	54.14	1204.6274	9	0.6	603.3214	2	42.22	2	F2:11819	12112021_RID_2349_DP2.raw		1.4264E7	3.00
A.GTLINQEWVLTAAR.C	N	48.88	1570.8467	14	0.5	786.4310	2	56.07	3	F3:16574	17112021_RID_2349_DP3.raw			2.60
V.PNEDQQIRVPK.E	Y	48.77	1322.6942	11	0.1	662.3544	2	30.86	2	F2:7883	12112021_RID_2349_DP2.raw		2.0109E5	
W.DKDIMLIR.L	N	44.22	1002.5532	8	-0.5	502.2836	2	37.38	3	F3:10341	17112021_RID_2349_DP3.raw			5.86
R.IILGVHSK.N	Y	43.01	865.5385	8	0.7	433.7769	2	26.46	2	F2:6227	12112021_RID_2349_DP2.raw		3.7847E7	1.23
W.DKDIM(+15.99)LIR.L	N	39.37	1018.5481	8	0.3	510.2815	2	31.54	3	F3:8358	17112021_RID_2349_DP3.raw			1.70
K.VTYPDVPH.C	N	39.33	926.4498	8	-0.6	464.2319	2	35.54	6	F6:9635	17112021_RID_2349_DP6.raw			
														total 1

CAB62591.1
back to list

| Protein Coverage | Supporting Peptides |
Protein Coverage:

1 MVLIRVLANL LVLQLSYAQK SSELVIGGDE CNINEHPFPV ALHTARSKRF YCAGTLINQE WVLTAARCDR KNIRIILGVH C

114 147

81 SKNVPNEDQQ IRVPKEKFFC LSSKTYTRWD KDIMLIRLKK PVNDSTHIVP LSLPSSPPSV GSVCRIMGWG TITTTKVTYP

161 DVPHCANINM FDYSVCRKVY RKLPEKSRTL CAGILQGGID SCKVDNGGPL ICNGQIQGIV SWGGHPCAQP HKPALYTNVF

241 DYTDWIQSII AGNITATCPP

Supporting Peptides:

Peptide	Uniq	-10lgP	Mass	Length	ppm	m/z	z	RT	Fraction	Scan	Source File	Area F1	Area F2	Are
K.TYTRWDKDIMLIR.L	N	82.41	1709.8923	13	0.4	570.9716	3	49.33	3	F3:14370	17112021_RID_2349_DP3.raw		1.4395E6	1.73
R.IMGWGTITTTK.V	N	78.23	1207.6271	11	-0.4	604.8206	2	47.11	3	F3:13741	17112021_RID_2349_DP3.raw	1.7839E6	2.7374E7	1.67
K.NVPNEDQQIRVPK.E	Y	74.15	1535.8055	13	0.1	768.9101	2	30.88	2	F2:7842	12112021_RID_2349_DP2.raw		1.5351E7	1.45
K.NVPNEDQQIR.V	Y	72.95	1211.5895	10	0.4	606.8022	2	24.54	2	F2:5672	12112021_RID_2349_DP2.raw	3.788E5	1.1029E8	3.95
R.IM(+15.99)GWGTITTTK.V	N	67.99	1223.6221	11	0.3	612.8185	2	40.75	2	F2:11290	12112021_RID_2349_DP2.raw	7.1945E5	1.2257E7	3.31
R.WDKDIMLIR.L	N	59.98	1188.6324	9	0.8	595.3240	2	47.61	2	F2:13483	12112021_RID_2349_DP2.raw		2.0105E7	1.37
N.VPNEDQQIR.V	Y	58.49	1097.5465	9	1.3	549.7812	2	22.54	3	F3:5342	17112021_RID_2349_DP3.raw		1.7116E5	6.42
R.WDKDIM(+15.99)LIR.L	N	54.14	1204.6274	9	0.6	603.3214	2	42.22	2	F2:11819	12112021_RID_2349_DP2.raw		1.4264E7	3.00
A.GTLINQEWVLTAAR.C	N	48.88	1570.8467	14	0.5	786.4310	2	56.07	3	F3:16574	17112021_RID_2349_DP3.raw			2.60
V.PNEDQQIRVPK.E	Y	48.77	1322.6942	11	0.1	662.3544	2	30.86	2	F2:7883	12112021_RID_2349_DP2.raw		2.0109E5	
W.DKDIMLIR.L	N	44.22	1002.5532	8	-0.5	502.2836	2	37.38	3	F3:10341	17112021_RID_2349_DP3.raw			5.86
R.IILGVHSK.N	Y	43.01	865.5385	8	0.7	433.7769	2	26.46	2	F2:6227	12112021_RID_2349_DP2.raw		3.7847E7	1.23
W.DKDIM(+15.99)LIR.L	N	39.37	1018.5481	8	0.3	510.2815	2	31.54	3	F3:8358	17112021_RID_2349_DP3.raw			1.70
K.VTYPDVPH.C	N	39.33	926.4498	8	-0.6	464.2319	2	35.54	6	F6:9635	17112021_RID_2349_DP6.raw			
														total 1

P0DL42.1
back to list

| Protein Coverage | Supporting Peptides |

Protein Coverage:

1 EVRPFLDVYQ RSACQTRETL VSILQEHPDE ISDIFRPSCV AVLRCSGCCT DESMKCTPVG KHTADIQIMR MNPRTHSSKM

81 EVMKFMEHTA CECRPRWKQG EPEGPKEPR

Supporting Peptides:

Peptide	Uniq	-10lgP	Mass	Length	ppm	m/z	z	RT	Fraction	Scan	Source File	Area F1	Area F2	Area F3	Area F4	Are F5
R.WKQGEPEGPKEPR	Y	78.94	1536.7684	13	-0.2	769.3914	2	21.61	7	F7:5034	17112021_RID_2349_DP7.raw					
K.HTADIQIMR.M	Y	70.22	1083.5496	9	0.5	542.7823	2	29.75	7	F7:8013	17112021_RID_2349_DP7.raw					
K.QGEPEGPKEPR	Y	69.86	1222.5941	11	-0.4	612.3041	2	17.34	7	F7:3495	17112021_RID_2349_DP7.raw					
K.HTADIQIM(+15.99)R.M	Y	62.06	1099.5444	9	0.2	550.7796	2	27.83	7	F7:7145	17112021_RID_2349_DP7.raw					
R.WKQGEPEGPK.E	Y	53.45	1154.5720	10	0.0	578.2933	2	19.26	7	F7:4324	17112021_RID_2349_DP7.raw					
R.PFLDVYQR.S	N	49.73	1036.5342	8	-0.1	519.2743	2	44.38	7	F7:12800	17112021_RID_2349_DP7.raw				0	
H.TADIQIMR.M	Y	49.17	946.4906	8	-0.5	474.2523	2	35.36	7	F7:9694	17112021_RID_2349_DP7.raw					
R.ETLVSILQEHPD.E	Y	48.06	1379.6932	12	0.5	690.8542	2	54.14	7	F7:16136	17112021_RID_2349_DP7.raw					
R.ETLVSILQEHP.D	Y	47.00	1264.6663	11	0.4	633.3406	2	55.20	7	F7:16471	17112021_RID_2349_DP7.raw					
V.RPFLDVYQR.S	N	45.90	1192.6353	9	1.7	597.3259	2	38.63	7	F7:10909	17112021_RID_2349_DP7.raw					
K.QGEPEGPKEP.R	Y	45.10	1066.4930	10	-0.3	534.2537	2	21.94	7	F7:5155	17112021_RID_2349_DP7.raw					
R.WKQGEPEGP.K	Y	44.23	1026.4771	9	0.6	514.2461	2	25.35	7	F7:6256	17112021_RID_2349_DP7.raw					
K.QGEPEGPK.E	Y	43.80	840.3977	8	0.3	421.2063	2	14.33	7	F7:2604	17112021_RID_2349_DP7.raw					
R.WKQGEPEGPKE.P	Y	42.99	1283.6145	11	0.6	642.8149	2	21.26	7	F7:4833	17112021_RID_2349_DP7.raw					
H.TADIQIM(+15.99)R.M	Y	40.76	962.4855	8	-0.7	482.2497	2	28.10	7	F7:7206	17112021_RID_2349_DP7.raw					

total 15 peptides

Q4VM07.1

back to list

| Protein Coverage | Supporting Peptides |

Protein Coverage:

1 MMQVLLVTIC LAVFPYQGSS IILESGNVND YEVVYPQKIT ALPKGAIQQP EQKYEDAIKY EFKVNGKPVV LHLEKNKGLF
81 SEDYSETHYT PDGREITINP PVEDHCYYHG RIQNDADSTA SISACNGLKG HFKLQGEMYL IEPLRIPDSE AHAIYKYENI
161 EKEDEAPKMC GVTQTNWESD EPIKASQLNL TPEQRTYLKS KKYVELVIVA DYIMFWKYDR SLSTIRTRIY EIVNTLNVIY

241 RFLNIYIALV AVEIWSKGDL INVTSSAYDT LDSFGEWRER DLLNRKRHDN AQLLTGINFN GPSAGRGFVG RMCQPKYSVG

321 IVQDHSKIYL LVASAMAHEM GHNLGMDHDR IDCTCGAKSC IMSGILRCET SYLFSDCSRE EHRKYLINKM PQCILNKPLK

401 TDIVSPAVCG NYFVEVGEEC DCGSPANCQD RCCDAATCKL RPGAQCGDGV CCYQCKFRRA GTVCRPANGE CDVSDLCTGQ
481 SAECPTDQFQ RNGQPCQNNK GYCYNGTCPI MEKQCISLFG ASATVAQDSC FQFNRRGNHY GYCRKENNTK IACAPEDVKC
561 GRLYCLDNSS GHKNPCQIYY IPSDENKGMV DPGTKCGDGM VCSNGKCVDV TIAY

Supporting Peptides:

Peptide	Uniq	-10lgP	Mass	Length	ppm	m/z	z	RT	Fraction	Scan	Source File	Area F1	Area F2
R.IYEIVNTLNVIYR.F	N	74.30	1608.8875	13	0.4	805.4514	2	61.67	3	F3:18319	17112021_RID_2349_DP3.raw		
R.HDNAQLLTGINFNGPSAGR.G	Y	73.01	1980.9766	19	0.3	991.4958	2	49.32	1	F1:15000	12112021_RID_2349_DP1.raw	2.0256E6	
K.YSVGIVQDHSK.I	Y	72.55	1231.6196	11	0.5	616.8174	2	28.91	3	F3:7512	17112021_RID_2349_DP3.raw	3.3389E5	4.2446E5
I.KASQLNLTPEQR.T	N	68.58	1383.7469	12	0.5	692.8811	2	28.68	1	F1:8063	12112021_RID_2349_DP1.raw	1.981E6	1.3728E5

total 12

Peptide	Uniq	-10lgP	Mass	Length	ppm	m/z	z	RT	Fraction	Scan	Source File	Area F1	Area F2
A.SQLNLTPEQR.T	N	61.22	1184.6150	10	0.3	593.3149	2	32.62	1	F1:9376	12112021_RID_2349_DP1.raw	1.0663E7	3.1363E6
K.ASQLNLTPEQR.T	N	61.13	1255.6521	11	0.9	628.8339	2	32.86	2	F2:8565	12112021_RID_2349_DP2.raw	5.7571E7	1.162E7
D.SFGEWRER.D	N	55.07	1065.4991	8	0.1	533.7569	2	31.26	2	F2:7954	12112021_RID_2349_DP2.raw	6.0265E6	2.4122E6
D.TLDSFGEWR.E	N	52.86	1109.5142	9	0.7	555.7648	2	47.54	2	F2:13529	12112021_RID_2349_DP2.raw		8.0796E4
R.HDNAQLLTGINFN.G	N	48.53	1455.7106	13	1.2	728.8634	2	53.68	3	F3:15808	17112021_RID_2349_DP3.raw		
R.ERDLLNR.K	N	42.02	914.4933	7	-0.1	458.2539	2	23.23	1	F1:6203	12112021_RID_2349_DP1.raw	1.8049E7	3.2804E6
Q.LNLTPEQR.T	N	41.26	969.5243	8	0.0	485.7694	2	30.89	7	F7:8269	17112021_RID_2349_DP7.raw	4.4645E5	
D.NAQLLTGINFNGPSAGR.G	Y	40.64	1728.8907	17	0.3	865.4529	2	51.82	1	F1:15761	12112021_RID_2349_DP1.raw	1.7741E4	

total 12

AAX38182.1
back to list

| Protein Coverage | Supporting Peptides |
Protein Coverage:

```
  1 MMQVLLVTIC LAVFPYQGSS IILESGNVND YEVVYPQKIT ALPKGAIQQP EQKYEDAIKY EFKVNGKPVV LHLEKNKGLF
 81 SEDYSETHYT PDGREITINP PVEDHCYYHG RIQNDADSTA SISACNGLKG HFKLQGEMYL IEPLRIPDSE AHAIYKYENI
161 EKEDEAPKMC GVTQTNWESD EPIKASQLNL TPEQRTYLKS KKYVELVIVA DYIMFWKYDR SLSTIRTRIY EIVNTLNVIY
241 RFLNIYIALV AVEIWSKGDL INVTSSAYDT LDSFGEWRER DLLNRKRHDN AQLLTGINFN GPSAGRGFVG RMCQPKYSVG
321 IVQDHSKIYL LVASAMAHEM GHNLGMDHDR IDCTCGAKSC IMSGILRCET SYLFSDCSRE EHRKYLINKM PQCILNKPLK
401 TDIVSPAVCG NYFVEVGEEC DCGSPANCQD RCCDAATCKL RPGAQCGDGV CCYQCKFRRA GTVCRPANGE CDVSDLCTGQ
481 SAECPTDQFQ RNGQPCQNNK GYCYNGTCPI MEKQCISLFG ASATVAQDSC FQFNRRGNHY GYCRKENNTK IACAPEDVKC
561 GRLYCLDNSS GHKNPCQIYY IPSDENKGMV DPGTKCGDGM VCSNGKCVDV TIAY
```

Supporting Peptides:

Peptide	Uniq	-10lgP	Mass	Length	ppm	m/z	z	RT	Fraction	Scan	Source File	Area F1	Area F2
R.IYEIVNTLNVIYR.F	N	74.30	1608.8875	13	0.4	805.4514	2	61.67	3	F3:18319	17112021_RID_2349_DP3.raw		
R.HDNAQLLTGINFNGPSAGR.G	Y	73.01	1980.9766	19	0.3	991.4958	2	49.32	1	F1:15000	12112021_RID_2349_DP1.raw	2.0256E6	
K.YSVGIVQDHSK.I	Y	72.55	1231.6196	11	0.5	616.8174	2	28.91	3	F3:7512	17112021_RID_2349_DP3.raw	3.3389E5	4.2446E5
I.KASQLNLTPEQR.T	N	68.58	1383.7469	12	0.5	692.8811	2	28.68	1	F1:8063	12112021_RID_2349_DP1.raw	1.981E6	1.3728E5
A.SQLNLTPEQR.T	N	61.22	1184.6150	10	0.3	593.3149	2	32.62	1	F1:9376	12112021_RID_2349_DP1.raw	1.0663E7	3.1363E6
K.ASQLNLTPEQR.T	N	61.13	1255.6521	11	0.9	628.8339	2	32.86	2	F2:8565	12112021_RID_2349_DP2.raw	5.7571E7	1.162E7
D.SFGEWRER.D	N	55.07	1065.4991	8	0.1	533.7569	2	31.26	2	F2:7954	12112021_RID_2349_DP2.raw	6.0265E6	2.4122E6
D.TLDSFGEWR.E	N	52.86	1109.5142	9	0.7	555.7648	2	47.54	2	F2:13529	12112021_RID_2349_DP2.raw		8.0796E4
R.HDNAQLLTGINFN.G	N	48.53	1455.7106	13	1.2	728.8634	2	53.68	3	F3:15808	17112021_RID_2349_DP3.raw		
R.ERDLLNR.K	N	42.02	914.4933	7	-0.1	458.2539	2	23.23	1	F1:6203	12112021_RID_2349_DP1.raw	1.8049E7	3.2804E6
Q.LNLTPEQR.T	N	41.26	969.5243	8	0.0	485.7694	2	30.89	7	F7:8269	17112021_RID_2349_DP7.raw	4.4645E5	
D.NAQLLTGINFNGPSAGR.G	Y	40.64	1728.8907	17	0.3	865.4529	2	51.82	1	F1:15761	12112021_RID_2349_DP1.raw	1.7741E4	

total 12

TRINITY_DN459
back to list

| Protein Coverage | Supporting Peptides |
Protein Coverage:

1 GQSAECPTDV FQRNGQPCQN NKGYCYNGDC PILTNQCISL FGSSVTVAPD VCFDYNLQGT EDYHCRRENG RYIKCEPQDK

81 KCGRLFCVKP PTGNTISCKF SRSKDDPDYG MVDLGTKCAD GKVCNSNRQC VDVNTA

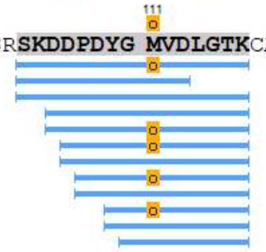

Supporting Peptides:

Peptide	Uniq	-10lgP	Mass	Length	ppm	m/z	z	RT	Fraction	Scan	Source File	Area F1	Area
R.SKDDPDYGM(+15.99)VDLGTK.C	Y	89.44	1655.7349	15	0.4	552.9191	3	38.10	1	F1:11983	12112021_RID_2349_DP1.raw	4.6116E7	1.206E
R.SKDDPDYGMVDLGTK.C	Y	87.92	1639.7399	15	-0.1	820.8771	2	39.20	3	F3:10983	17112021_RID_2349_DP3.raw	8.1952E7	2.5565
K.DDPDYGMVDLGTK.C	N	86.80	1424.6129	13	0.9	713.3144	2	48.02	1	F1:14542	12112021_RID_2349_DP1.raw	3.1015E5	
D.PDYGMVDLGTK.C	N	73.86	1194.5591	11	-0.1	598.2867	2	43.94	1	F1:13219	12112021_RID_2349_DP1.raw	7.4048E6	4.1966
D.PDYGM(+15.99)VDLGTK.C	N	71.86	1210.5540	11	0.4	606.2845	2	40.64	1	F1:13230	12112021_RID_2349_DP1.raw	6.7718E6	3.5085
K.DDPDYGM(+15.99)VDLGTK.C	N	69.19	1440.6078	13	0.3	721.3114	2	40.35	6	F6:11298	17112021_RID_2349_DP6.raw	7.0367E4	
D.DPDYGMVDLGTK.C	N	68.28	1309.5859	12	1.2	655.8010	2	47.41	1	F1:14315	12112021_RID_2349_DP1.raw	7.5385E5	
D.DPDYGM(+15.99)VDLGTK.C	N	61.75	1325.5809	12	0.8	663.7983	2	39.96	1	F1:11872	12112021_RID_2349_DP1.raw	2.7576E5	
D.YGM(+15.99)VDLGTK.C	N	54.16	998.4742	9	0.8	500.2448	2	39.32	1	F1:11674	12112021_RID_2349_DP1.raw	1.0157E5	
R.SKDDPDYGMVD.L	Y	46.37	1240.4917	11	0.7	621.2535	2	34.22	1	F1:10024	12112021_RID_2349_DP1.raw	4.9296E5	
D.YGMVDLGTK.C	N	45.13	982.4794	9	-0.5	492.2467	2	39.32	1	F1:11699	12112021_RID_2349_DP1.raw	1.3963E5	
Y.GMVDLGTK.C	N	44.20	819.4160	8	0.0	410.7153	2	30.74	7	F7:8124	17112021_RID_2349_DP7.raw		

TRINITY_DN1910
back to list

| Protein Coverage | Supporting Peptides |
Protein Coverage:

1 TISCKFSRSK DDPDYGMVDL GTKCADGKNS ANQCFDPIMC ERREEETCAS GPCCRRCKVR LAHF

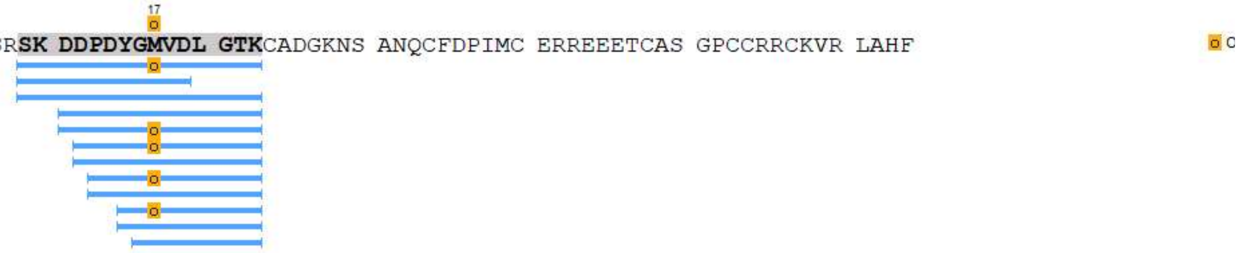

Supporting Peptides:

Peptide	Uniq	-10lgP	Mass	Length	ppm	m/z	z	RT	Fraction	Scan	Source File	Area F1	Area
R.SKDDPDYGM(+15.99)VDLGTK.C	Y	89.44	1655.7349	15	0.4	552.9191	3	38.10	1	F1:11983	12112021_RID_2349_DP1.raw	4.6116E7	1.206E
R.SKDDPDYGMVDLGTK.C	Y	87.92	1639.7399	15	-0.1	820.8771	2	39.20	3	F3:10983	17112021_RID_2349_DP3.raw	8.1952E7	2.5565
K.DDPDYGMVDLGTK.C	N	86.80	1424.6129	13	0.9	713.3144	2	48.02	1	F1:14542	12112021_RID_2349_DP1.raw	3.1015E5	
D.PDYGMVDLGTK.C	N	73.86	1194.5591	11	-0.1	598.2867	2	43.94	1	F1:13219	12112021_RID_2349_DP1.raw	7.4048E6	4.1966
D.PDYGM(+15.99)VDLGTK.C	N	71.86	1210.5540	11	0.4	606.2845	2	40.64	1	F1:13230	12112021_RID_2349_DP1.raw	6.7718E6	3.5085
K.DDPDYGM(+15.99)VDLGTK.C	N	69.19	1440.6078	13	0.3	721.3114	2	40.35	6	F6:11298	17112021_RID_2349_DP6.raw	7.0367E4	
D.DPDYGMVDLGTK.C	N	68.28	1309.5859	12	1.2	655.8010	2	47.41	1	F1:14315	12112021_RID_2349_DP1.raw	7.5385E5	
D.DPDYGM(+15.99)VDLGTK.C	N	61.75	1325.5809	12	0.8	663.7983	2	39.96	1	F1:11872	12112021_RID_2349_DP1.raw	2.7576E5	
D.YGM(+15.99)VDLGTK.C	N	54.16	998.4742	9	0.8	500.2448	2	39.32	1	F1:11674	12112021_RID_2349_DP1.raw	1.0157E5	
R.SKDDPDYGMVD.L	Y	46.37	1240.4917	11	0.7	621.2535	2	34.22	1	F1:10024	12112021_RID_2349_DP1.raw	4.9296E5	

Peptide	Uniq	-10lgP	Mass	Length	ppm	m/z	z	RT	Fraction	Scan	Source File	Area F1	Area
D.YGMVDLGTK.C	N	45.13	982.4794	9	-0.5	492.2467	2	39.32	1	F1:11699	12112021_RID_2349_DP1.raw	1.3963E5	
Y.GMVDLGTK.C	N	44.20	819.4160	8	0.0	410.7153	2	30.74	7	F7:8124	17112021_RID_2349_DP7.raw		

t

TRINITY_DN509
back to list

| Protein Coverage | Supporting Peptides |
Protein Coverage:

```
  1 MKYAIVCLLL VGSVFAAEDD DKKEDVGTVV GIDLGTTYSC VGVFKNGRVE IIANDQGNRI TPSYVAFTPE GERLIGDAAK
 81 NQLTSNPENT VFDAKRLIGR TWNDPSVQQD IKYLPFKVIE KKTKPYIQVD VGGGQTKTFA PEEISAMVLT KMKETAEAYL
161 GKKVTHAVVT VPAYFNDAQR QATKDAGTIA GLNVMRIINE PTAAAIAYGL DKREGEKNIL VFDLGGGTFD VSLLTIDNGV
241 FEVVATNGDT HLGGEDFDQR VMEHFIKLYK KKTGKDVRKD NRAVQKLRRE VEKAKRALSS QHQARIEIES FFEGEDFSET
321 LTRAKFEELN MDLFRSTMKP VQKVLEDADL KKPDIDEIVL VGGSTRIPKI QQLVKEFFNG KEPSRGINPD EAVAYGAAVQ
401 AGVLSGDQDT GDLVLLDVCP LTLGIETVGG VMTKLIPRNT VVPTKKSQIF STASDNQPTV TIKVYEGERP LTKDNHLLGT
481 FDLTGIPPAP RGVPQIEVTF EIDVNGILRV TAEDKGTGNK NKITITNDQN RLTPEEIERM VNDAEKFAEE DKKLKERIDA
561 RNELESYAYS LKNQIGDKEK LGGKLSTEDK ETIEKAVEEK IEWLESHQEA DIEDFKAQKK ELEEVVQPIV GKLYGGAGPP
641 PGGEEPGEKD EL
```

Supporting Peptides:

Peptide	Uniq	-10lgP	Mass	Length	ppm	m/z	z	RT	Fraction	Scan	Source File	Area F1	Area F2	Area F3
K.SQIFSTASDNQPTVTIK.V	Y	78.43	1835.9265	17	0.7	918.9712	2	43.86	4	F4:12557	17112021_RID_2349_DP4.raw			
K.ELEEVVQPIVGK.L	Y	68.74	1338.7394	12	0.1	670.3770	2	46.15	4	F4:13270	17112021_RID_2349_DP4.raw			
R.NELESYAYSLK.N	Y	67.75	1315.6295	11	0.6	658.8224	2	45.74	4	F4:13114	17112021_RID_2349_DP4.raw			
R.ITPSYVAFTPEGER.L	Y	63.57	1565.7725	14	0.5	783.8939	2	46.47	2	F2:13149	12112021_RID_2349_DP2.raw		8.8984E4	
K.VYEGERPLTK.D	Y	55.73	1190.6295	10	0.6	596.3224	2	22.63	4	F4:5257	17112021_RID_2349_DP4.raw			
R.LTPEEIER.M	Y	49.69	985.5080	8	-0.1	493.7612	2	31.13	4	F4:8215	17112021_RID_2349_DP4.raw			
K.NQLTSNPENTVFDAK.R	Y	48.37	1676.8005	15	1.1	839.4084	2	40.91	2	F2:11316	12112021_RID_2349_DP2.raw		1.9104E4	
K.LYGGAGPPPGGEEPGEKDEL	Y	45.98	1967.9111	20	0.8	984.9636	2	40.85	4	F4:11534	17112021_RID_2349_DP4.raw			
R.TWNDPSVQQDIK.Y	Y	45.02	1429.6837	12	0.3	715.8494	2	37.71	2	F2:10252	12112021_RID_2349_DP2.raw		2.2693E4	
K.LYGGAGPPPGGEEPGEKDE.L	Y	42.79	1854.8271	19	1.1	928.4219	2	33.35	4	F4:8886	17112021_RID_2349_DP4.raw			
K.LYGGAGPPPGGEEPGEK.D	Y	38.90	1610.7576	17	0.9	806.3868	2	31.58	4	F4:8348	17112021_RID_2349_DP4.raw			

total 11 peptide

TRINITY_DN509
back to list

| Protein Coverage | Supporting Peptides |
Protein Coverage:

```
  1 PREAAGGYIS ADGRPGLNTR SGETSAWEGS ERRSAAAGSP SRRDRVLLLV WCVREHPPSK SNMKYAIVCL LLVGSVFAAE
 81 DDDKKEDVGT VVGIDLGTTY SCVGVFKNGR VEIIANDQGN RITPSYVAFT PEGERLIGDA AKNQLTSNPE NTVFDAKRLI
161 GRTWNDPSVQ QDIKYLPFKV IEKKTKPYIQ VDVGGGQTKT FAPEEISAMV LTKMKETAEA YLGKKVTHAV VTVPAYFNDA
241 QRQATKDAGT IAGLNVMRII NEPTAAAIAY GLDKREGEKN ILVFDLGGGT FDVSLLTIDN GVFEVVATNG DTHLGGEDFD
321 QRVMEHFIKL YKKKTGKDVR KDNRAVQKLR REVEKAKRAL SSQHQARIEI ESFFEGEDFS ETLTRAKFEE LNMDLFRSTM
401 KPVQKVLEDA DLKKPDIDEI VLVGGSTRIP KIQQLVKEFF NGKEPSRGIN PDEAVAYGAA VQAGVLSGDQ DTGDLVLLDV
481 CPLTLGIETV GGVMTKLIPR NTVVPTKKSQ IFSTASDNQP TVTIKVYEGE RPLTKDNHLL GTFDLTGIPP APRGVPQIEV
561 TFEIDVNGIL RVTAEDKGTG NKNKITITND QNRLTPEEIE RMVNDAEKFA EEDKKLKERI DARNELESYA YSLKNQIGDK
641 EKLGGKLSTE DKETIEKAVE EKIEWLESHQ EADIEDFKAQ KKELEEVVQP IVGKLYGGAG PPPGGEEPGE KDEL
```

Supporting Peptides:

Peptide	Uniq	-10lgP	Mass	Length	ppm	m/z	z	RT	Fraction	Scan	Source File	Area F1	Area F2	Area F3
K.SQIFSTASDNQPTVTIK.V	Y	78.43	1835.9265	17	0.7	918.9712	2	43.86	4	F4:12557	17112021_RID_2349_DP4.raw			
K.ELEEVVQPIVGK.L	Y	68.74	1338.7394	12	0.1	670.3770	2	46.15	4	F4:13270	17112021_RID_2349_DP4.raw			
R.NELESYAYSLK.N	Y	67.75	1315.6295	11	0.6	658.8224	2	45.74	4	F4:13114	17112021_RID_2349_DP4.raw			
R.ITPSYVAFTPEGER.L	Y	63.57	1565.7725	14	0.5	783.8939	2	46.47	2	F2:13149	12112021_RID_2349_DP2.raw		8.8984E4	
K.VYEGERPLTK.D	Y	55.73	1190.6295	10	0.6	596.3224	2	22.63	4	F4:5257	17112021_RID_2349_DP4.raw			
R.LTPEEIER.M	Y	49.69	985.5080	8	-0.1	493.7612	2	31.13	4	F4:8215	17112021_RID_2349_DP4.raw			
K.NQLTSNPENTVFDAK.R	Y	48.37	1676.8005	15	1.1	839.4084	2	40.91	2	F2:11316	12112021_RID_2349_DP2.raw		1.9104E4	
K.LYGGAGPPPGGEEPGEKDEL	Y	45.98	1967.9111	20	0.8	984.9636	2	40.85	4	F4:11534	17112021_RID_2349_DP4.raw			
R.TWNDPSVQQDIK.Y	Y	45.02	1429.6837	12	0.3	715.8494	2	37.71	2	F2:10252	12112021_RID_2349_DP2.raw		2.2693E4	
K.LYGGAGPPPGGEEPGEKDE.L	Y	42.79	1854.8271	19	1.1	928.4219	2	33.35	4	F4:8886	17112021_RID_2349_DP4.raw			
K.LYGGAGPPPGGEEPGEK.D	Y	38.90	1610.7576	17	0.9	806.3868	2	31.58	4	F4:8348	17112021_RID_2349_DP4.raw			

total 11 peptide

ADK22823.1
back to list

| Protein Coverage | Supporting Peptides |
Protein Coverage:

```
 1 MGRFISVSFG LLVVFLSLSG TGADCPSEWS SHEGHCYKVF KLLKTWEDAE KFCTQQANGW HLASIESVEE ANFVAQLASE

81 TLTKSKYHAW IGLRDQSKRQ QCSSHWTDGS AVSYETVTKY TKCFGLNKET KYHEWITLPC GDKNPFICKS WVLH
```

Supporting Peptides:

Peptide	Uniq	-10lgP	Mass	Length	ppm	m/z	z	RT	Fraction	Scan	Source File	Area F1	Area F2	Area F3	Area F4	Ar F
K.LLKTWEDAEK.F	N	73.82	1231.6448	10	0.3	616.8298	2	32.46	7	F7:8767	17112021_RID_2349_DP7.raw					
F.VAQLASETLTK.S	Y	64.28	1159.6448	11	0.4	580.8299	2	32.58	7	F7:8832	17112021_RID_2349_DP7.raw					
N.FVAQLASETLTK.S	Y	63.48	1306.7133	12	0.5	654.3643	2	44.09	7	F7:12823	17112021_RID_2349_DP7.raw					
K.YHAWIGLR.D	N	61.14	1014.5399	8	-0.1	508.2772	2	40.32	7	F7:11939	17112021_RID_2349_DP7.raw					3.38
D.GSAVSYETVTK.Y	N	57.05	1140.5663	11	0.1	571.2905	2	28.66	6	F6:7260	17112021_RID_2349_DP6.raw					
V.AQLASETLTK.S	Y	56.80	1060.5764	10	0.2	531.2956	2	28.42	7	F7:7389	17112021_RID_2349_DP7.raw					
K.SKYHAWIGLR.D	N	51.39	1229.6669	10	0.3	615.8409	2	41.34	7	F7:11753	17112021_RID_2349_DP7.raw					
Y.HAWIGLR.D	N	46.00	851.4766	7	0.5	426.7458	2	35.11	7	F7:9748	17112021_RID_2349_DP7.raw					
H.WTDGSAVSYETVTK.Y	N	45.93	1542.7202	14	0.5	772.3678	2	39.78	6	F6:11115	17112021_RID_2349_DP6.raw					
A.QLASETLTK.S	Y	44.28	989.5393	9	-0.3	495.7768	2	26.35	7	F7:6702	17112021_RID_2349_DP7.raw					
K.TWEDAEK.F	N	42.92	877.3817	7	0.0	439.6982	2	23.16	7	F7:5532	17112021_RID_2349_DP7.raw					
K.TWEDAEKF.C	N	40.38	1024.4502	8	-0.9	513.2319	2	40.86	7	F7:11654	17112021_RID_2349_DP7.raw					

total 12 peptides

ABA86561.1
back to list

| Protein Coverage | Supporting Peptides |
Protein Coverage:

```
 1 MGRFISVSFG LLVVFLSLSG TGADCPSEWS SHEGHCYKVF KLLKTWEDAE KFCTQQANGW HLASIESVEE ANFVAQLASE

81 TLTKSKYHAW IGLRDQSKRQ QCSSHWTDGS AVSYETVTKY TKCFGLNKET KYHEWITLPC GDKNPFICKS WVLH
```

Supporting Peptides:

Peptide	Uniq	-10lgP	Mass	Length	ppm	m/z	z	RT	Fraction	Scan	Source File	Area F1	Area F2	Area F3	Area F4	Ar F
K.LLKTWEDAEK.F	N	73.82	1231.6448	10	0.3	616.8298	2	32.46	7	F7:8767	17112021_RID_2349_DP7.raw					
F.VAQLASETLTK.S	Y	64.28	1159.6448	11	0.4	580.8299	2	32.58	7	F7:8832	17112021_RID_2349_DP7.raw					
N.FVAQLASETLTK.S	Y	63.48	1306.7133	12	0.5	654.3643	2	44.09	7	F7:12823	17112021_RID_2349_DP7.raw					
K.YHAWIGLR.D	N	61.14	1014.5399	8	-0.1	508.2772	2	40.32	7	F7:11939	17112021_RID_2349_DP7.raw					3.38
D.GSAVSYETVTK.Y	N	57.05	1140.5663	11	0.1	571.2905	2	28.66	6	F6:7260	17112021_RID_2349_DP6.raw					
V.AQLASETLTK.S	Y	56.80	1060.5764	10	0.2	531.2956	2	28.42	7	F7:7389	17112021_RID_2349_DP7.raw					
K.SKYHAWIGLR.D	N	51.39	1229.6669	10	0.3	615.8409	2	41.34	7	F7:11753	17112021_RID_2349_DP7.raw					
Y.HAWIGLR.D	N	46.00	851.4766	7	0.5	426.7458	2	35.11	7	F7:9748	17112021_RID_2349_DP7.raw					
H.WTDGSAVSYETVTK.Y	N	45.93	1542.7202	14	0.5	772.3678	2	39.78	6	F6:11115	17112021_RID_2349_DP6.raw					
A.QLASETLTK.S	Y	44.28	989.5393	9	-0.3	495.7768	2	26.35	7	F7:6702	17112021_RID_2349_DP7.raw					
K.TWEDAEK.F	N	42.92	877.3817	7	0.0	439.6982	2	23.16	7	F7:5532	17112021_RID_2349_DP7.raw					
K.TWEDAEKF.C	N	40.38	1024.4502	8	-0.9	513.2319	2	40.86	7	F7:11654	17112021_RID_2349_DP7.raw					

total 12 peptides

Q38L02.1
back to list

| Protein Coverage | Supporting Peptides |
Protein Coverage:

1 MGRFISVSFG LLVVFLSLSG TGADCPSEWS SHEGHCYKVF K**LLKTWEDAE KF**CTQQANGW HLASIESVEE AN**FVAQLASE**

81 **TLTKSKYHAW IGLR**DQSKRQ QCSSH**WTDGS AVSYETVTK**Y TKCFGLNKET KYHEWITLPC GDKNPFICKS WVLH

Supporting Peptides:

Peptide	Uniq	-10lgP	Mass	Length	ppm	m/z	z	RT	Fraction	Scan	Source File	Area F1	Area F2	Area F3	Area F4	Ar F
K.LLKTWEDAEK.F	N	73.82	1231.6448	10	0.3	616.8298	2	32.46	7	F7:8767	17112021_RID_2349_DP7.raw					
F.VAQLASETLTK.S	Y	64.28	1159.6448	11	0.4	580.8299	2	32.58	7	F7:8832	17112021_RID_2349_DP7.raw					
N.FVAQLASETLTK.S	Y	63.48	1306.7133	12	0.5	654.3643	2	44.09	7	F7:12823	17112021_RID_2349_DP7.raw					
K.YHAWIGLR.D	N	61.14	1014.5399	8	-0.1	508.2772	2	40.32	7	F7:11939	17112021_RID_2349_DP7.raw					3.38
D.GSAVSYETVTK.Y	N	57.05	1140.5663	11	0.1	571.2905	2	28.66	6	F6:7260	17112021_RID_2349_DP6.raw					
V.AQLASETLTK.S	Y	56.80	1060.5764	10	0.2	531.2956	2	28.42	7	F7:7389	17112021_RID_2349_DP7.raw					
K.SKYHAWIGLR.D	N	51.39	1229.6669	10	0.3	615.8409	2	41.34	7	F7:11753	17112021_RID_2349_DP7.raw					
Y.HAWIGLR.D	N	46.00	851.4766	7	0.5	426.7458	2	35.11	7	F7:9748	17112021_RID_2349_DP7.raw					
H.WTDGSAVSYETVTK.Y	N	45.93	1542.7202	14	0.5	772.3678	2	39.78	6	F6:11115	17112021_RID_2349_DP6.raw					
A.QLASETLTK.S	Y	44.28	989.5393	9	-0.3	495.7768	2	26.35	7	F7:6702	17112021_RID_2349_DP7.raw					
K.TWEDAEK.F	N	42.92	877.3817	7	0.0	439.6982	2	23.16	7	F7:5532	17112021_RID_2349_DP7.raw					
K.TWEDAEKF.C	N	40.38	1024.4502	8	-0.9	513.2319	2	40.86	7	F7:11654	17112021_RID_2349_DP7.raw					

total 12 peptides

ADK22821.1
back to list

| Protein Coverage | Supporting Peptides |
Protein Coverage:

1 MGRFISVSFG LLVVFLSLSG TGADCPSDWS SHEGHCYKVF KLLKTWEDAE KFCTQQANGW HLASIESVEE ANFVAQLASE

81 TLTKSKYHAW IGLRDQSQRQ QCSSHWTDGS AVSYETVTKY TKCFGLNKET KYHEWITLPC GDKNPFICKS WVLH

Supporting Peptides:

Peptide	Uniq	-10lgP	Mass	Length	ppm	m/z	z	RT	Fraction	Scan	Source File	Area F1	Area F2	Area F3	Area F4	Ar F
K.LLKTWEDAEK.F	N	73.82	1231.6448	10	0.3	616.8298	2	32.46	7	F7:8767	17112021_RID_2349_DP7.raw					
F.VAQLASETLTK.S	Y	64.28	1159.6448	11	0.4	580.8299	2	32.58	7	F7:8832	17112021_RID_2349_DP7.raw					
N.FVAQLASETLTK.S	Y	63.48	1306.7133	12	0.5	654.3643	2	44.09	7	F7:12823	17112021_RID_2349_DP7.raw					
K.YHAWIGLR.D	N	61.14	1014.5399	8	-0.1	508.2772	2	40.32	7	F7:11939	17112021_RID_2349_DP7.raw					3.38[illegible]
D.GSAVSYETVTK.Y	N	57.05	1140.5663	11	0.1	571.2905	2	28.66	6	F6:7260	17112021_RID_2349_DP6.raw					
V.AQLASETLTK.S	Y	56.80	1060.5764	10	0.2	531.2956	2	28.42	7	F7:7389	17112021_RID_2349_DP7.raw					
K.SKYHAWIGLR.D	N	51.39	1229.6669	10	0.3	615.8409	2	41.34	7	F7:11753	17112021_RID_2349_DP7.raw					
Y.HAWIGLR.D	N	46.00	851.4766	7	0.5	426.7458	2	35.11	7	F7:9748	17112021_RID_2349_DP7.raw					
H.WTDGSAVSYETVTK.Y	N	45.93	1542.7202	14	0.5	772.3678	2	39.78	6	F6:11115	17112021_RID_2349_DP6.raw					
A.QLASETLTK.S	Y	44.28	989.5393	9	-0.3	495.7768	2	26.35	7	F7:6702	17112021_RID_2349_DP7.raw					
K.TWEDAEK.F	N	42.92	877.3817	7	0.0	439.6982	2	23.16	7	F7:5532	17112021_RID_2349_DP7.raw					
K.TWEDAEKF.C	N	40.38	1024.4502	8	-0.9	513.2319	2	40.86	7	F7:11654	17112021_RID_2349_DP7.raw					

total 12 peptides

AAZ53176.1
back to list

| Protein Coverage | Supporting Peptides |
Protein Coverage:

1 MRTLWIVAVC LIGVEGNLYQ FGEMINQKTG NFGLLSYVYY GCYCGWGGKG KPQDATDRCC FVHDCCYGRV KGCDPKTATY

81 SYSFENGDIV CGGDDPCLRA VCECDRVAAI CFRENMNTYD KKYMLYSIFD CKEESDQC

Supporting Peptides:

Peptide	Uniq	-10lgP	Mass	Length	ppm	m/z	z	RT	Fraction	Scan	Source File	Area F1	Area F2	A
G.NLYQFGEM(+15.99)INQK.T	Y	70.27	1499.7079	12	-0.1	750.8611	2	48.50	7	F7:14280	17112021_RID_2349_DP7.raw	1.5509E5	5.2464E4	2.[illegible]
G.NLYQFGEMINQK.T	Y	69.45	1483.7129	12	0.2	742.8639	2	50.67	7	F7:15227	17112021_RID_2349_DP7.raw	4.284E5		1.[illegible]
N.LYQFGEM(+15.99)INQK.T	Y	66.62	1385.6649	11	0.4	693.8400	2	39.94	7	F7:11327	17112021_RID_2349_DP7.raw			
N.LYQFGEMINQK.T	Y	66.11	1369.6700	11	0.2	685.8424	2	47.17	7	F7:13715	17112021_RID_2349_DP7.raw			
E.GNLYQFGEMINQK.T	Y	61.39	1540.7344	13	0.5	771.3749	2	54.08	7	F7:16198	17112021_RID_2349_DP7.raw			
L.YQFGEMINQK.T	Y	61.03	1256.5859	10	0.0	629.3002	2	42.06	7	F7:12071	17112021_RID_2349_DP7.raw			
K.TATYSYSFENGDIV.C	Y	54.07	1565.6885	14	0.6	783.8520	2	57.78	7	F7:17385	17112021_RID_2349_DP7.raw			
R.ENMNTYDK.K	N	52.21	1013.4124	8	0.7	507.7138	2	19.60	7	F7:4282	17112021_RID_2349_DP7.raw			
K.GKPQDATDR.C	N	48.61	986.4781	9	-0.5	494.2461	2	11.50	7	F7:1839	17112021_RID_2349_DP7.raw			
C.FRENMNTYDKK.Y	Y	45.46	1444.6769	11	-0.4	482.5660	3	21.59	7	F7:4995	17112021_RID_2349_DP7.raw			
R.ENMNTYDKK.Y	N	43.49	1141.5073	9	-0.1	571.7609	2	15.11	7	F7:2868	17112021_RID_2349_DP7.raw			

tota

Peptide	Uniq	-10lgP	Mass	Length	ppm	m/z	z	RT	Fraction	Scan	Source File	Area F1	Area F2	A
K.KYMLYSIFD.C	N	43.04	1178.5681	9	0.2	590.2914	2	60.72	7	F7:18383	17112021_RID_2349_DP7.raw			
R.ENMNTYDKKY.M	Y	42.65	1304.5707	10	-0.7	653.2922	2	23.23	7	F7:5555	17112021_RID_2349_DP7.raw			
Q.FGEMINQK.T	Y	39.53	965.4641	8	-0.3	483.7391	2	31.06	7	F7:8248	17112021_RID_2349_DP7.raw			
C.FRENMNTYDK.K	Y	39.33	1316.5819	10	0.4	659.2985	2	24.51	7	F7:6037	17112021_RID_2349_DP7.raw			

tota

A8CG78.1
back to list

| Protein Coverage | Supporting Peptides |
Protein Coverage:

24

1 MRTLWIVAVC LIGVE**GNLYQ FGEMINQK**TG NFGLLSYVYY GCYCGWGGK**G KPQDATDR**CC FVHDCCYGRV KGCDPK**TATY** C

81 **SYSFENGDIV** CGGDDPCLRA VCECDRVAAI C**FRENMNTYD KKYMLYSIFD** CKEESDQC

Supporting Peptides:

Peptide	Uniq	-10lgP	Mass	Length	ppm	m/z	z	RT	Fraction	Scan	Source File	Area F1	Area F2	A
G.NLYQFGEM(+15.99)INQK.T	Y	70.27	1499.7079	12	-0.1	750.8611	2	48.50	7	F7:14280	17112021_RID_2349_DP7.raw	1.5509E5	5.2464E4	2.[illegible]
G.NLYQFGEMINQK.T	Y	69.45	1483.7129	12	0.2	742.8639	2	50.67	7	F7:15227	17112021_RID_2349_DP7.raw	4.284E5		1.[illegible]
N.LYQFGEM(+15.99)INQK.T	Y	66.62	1385.6649	11	0.4	693.8400	2	39.94	7	F7:11327	17112021_RID_2349_DP7.raw			
N.LYQFGEMINQK.T	Y	66.11	1369.6700	11	0.2	685.8424	2	47.17	7	F7:13715	17112021_RID_2349_DP7.raw			
E.GNLYQFGEMINQK.T	Y	61.39	1540.7344	13	0.5	771.3749	2	54.08	7	F7:16198	17112021_RID_2349_DP7.raw			
L.YQFGEMINQK.T	Y	61.03	1256.5859	10	0.0	629.3002	2	42.06	7	F7:12071	17112021_RID_2349_DP7.raw			
K.TATYSYSFENGDIV.C	Y	54.07	1565.6885	14	0.6	783.8520	2	57.78	7	F7:17385	17112021_RID_2349_DP7.raw			
R.ENMNTYDK.K	N	52.21	1013.4124	8	0.7	507.7138	2	19.60	7	F7:4282	17112021_RID_2349_DP7.raw			
K.GKPQDATDR.C	N	48.61	986.4781	9	-0.5	494.2461	2	11.50	7	F7:1839	17112021_RID_2349_DP7.raw			
C.FRENMNTYDKK.Y	Y	45.46	1444.6769	11	-0.4	482.5660	3	21.59	7	F7:4995	17112021_RID_2349_DP7.raw			
R.ENMNTYDKK.Y	N	43.49	1141.5073	9	-0.1	571.7609	2	15.11	7	F7:2868	17112021_RID_2349_DP7.raw			
K.KYMLYSIFD.C	N	43.04	1178.5681	9	0.2	590.2914	2	60.72	7	F7:18383	17112021_RID_2349_DP7.raw			
R.ENMNTYDKKY.M	Y	42.65	1304.5707	10	-0.7	653.2922	2	23.23	7	F7:5555	17112021_RID_2349_DP7.raw			
Q.FGEMINQK.T	Y	39.53	965.4641	8	-0.3	483.7391	2	31.06	7	F7:8248	17112021_RID_2349_DP7.raw			
C.FRENMNTYDK.K	Y	39.33	1316.5819	10	0.4	659.2985	2	24.51	7	F7:6037	17112021_RID_2349_DP7.raw			

tota

AAZ53181.1
back to list

| Protein Coverage | Supporting Peptides |
Protein Coverage:

24

1 MRTLWIVAVC LIGVE**GNLYQ FGEMINQK**TG NFGLLSYVYY GCYCGWGGK**G KPQDATDR**CC FVHDCCYGRV KGCDPK**TATY** C

81 **SYSFENGDIV** CGGDDPCLRA VCECDRVAAI C**FRENMNTYD KKYMLYSIFD** CKEESDQC

Supporting Peptides:

Peptide	Uniq	-10lgP	Mass	Length	ppm	m/z	z	RT	Fraction	Scan	Source File	Area F1	Area F2	A
G.NLYQFGEM(+15.99)INQK.T	Y	70.27	1499.7079	12	-0.1	750.8611	2	48.50	7	F7:14280	17112021_RID_2349_DP7.raw	1.5509E5	5.2464E4	2.[illegible]
G.NLYQFGEMINQK.T	Y	69.45	1483.7129	12	0.2	742.8639	2	50.67	7	F7:15227	17112021_RID_2349_DP7.raw	4.284E5		1.[illegible]
N.LYQFGEM(+15.99)INQK.T	Y	66.62	1385.6649	11	0.4	693.8400	2	39.94	7	F7:11327	17112021_RID_2349_DP7.raw			
N.LYQFGEMINQK.T	Y	66.11	1369.6700	11	0.2	685.8424	2	47.17	7	F7:13715	17112021_RID_2349_DP7.raw			
E.GNLYQFGEMINQK.T	Y	61.39	1540.7344	13	0.5	771.3749	2	54.08	7	F7:16198	17112021_RID_2349_DP7.raw			
L.YQFGEMINQK.T	Y	61.03	1256.5859	10	0.0	629.3002	2	42.06	7	F7:12071	17112021_RID_2349_DP7.raw			
K.TATYSYSFENGDIV.C	Y	54.07	1565.6885	14	0.6	783.8520	2	57.78	7	F7:17385	17112021_RID_2349_DP7.raw			
R.ENMNTYDK.K	N	52.21	1013.4124	8	0.7	507.7138	2	19.60	7	F7:4282	17112021_RID_2349_DP7.raw			
K.GKPQDATDR.C	N	48.61	986.4781	9	-0.5	494.2461	2	11.50	7	F7:1839	17112021_RID_2349_DP7.raw			
C.FRENMNTYDKK.Y	Y	45.46	1444.6769	11	-0.4	482.5660	3	21.59	7	F7:4995	17112021_RID_2349_DP7.raw			
R.ENMNTYDKK.Y	N	43.49	1141.5073	9	-0.1	571.7609	2	15.11	7	F7:2868	17112021_RID_2349_DP7.raw			
K.KYMLYSIFD.C	N	43.04	1178.5681	9	0.2	590.2914	2	60.72	7	F7:18383	17112021_RID_2349_DP7.raw			
R.ENMNTYDKKY.M	Y	42.65	1304.5707	10	-0.7	653.2922	2	23.23	7	F7:5555	17112021_RID_2349_DP7.raw			
Q.FGEMINQK.T	Y	39.53	965.4641	8	-0.3	483.7391	2	31.06	7	F7:8248	17112021_RID_2349_DP7.raw			
C.FRENMNTYDK.K	Y	39.33	1316.5819	10	0.4	659.2985	2	24.51	7	F7:6037	17112021_RID_2349_DP7.raw			

tota

A8CG87.1
back to list

| Protein Coverage | Supporting Peptides |
Protein Coverage:

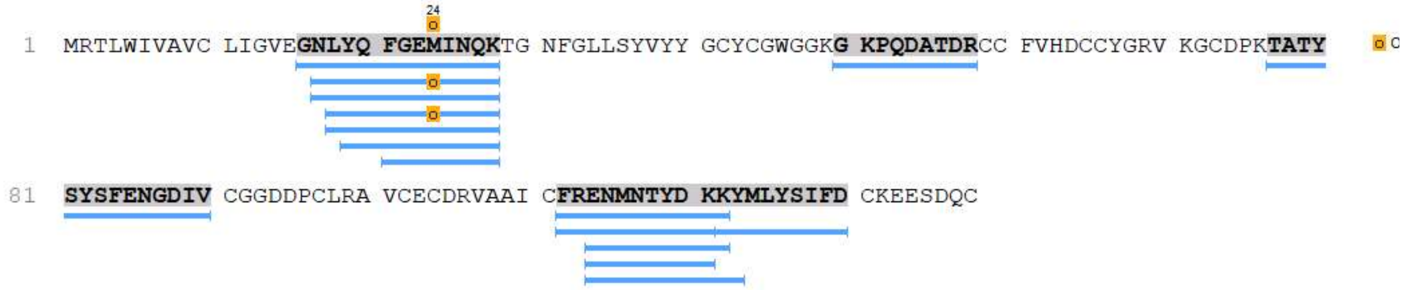

Supporting Peptides:

Peptide	Uniq	-10lgP	Mass	Length	ppm	m/z	z	RT	Fraction	Scan	Source File	Area F1	Area F2	A
G.NLYQFGEM(+15.99)INQK.T	Y	70.27	1499.7079	12	-0.1	750.8611	2	48.50	7	F7:14280	17112021_RID_2349_DP7.raw	1.5509E5	5.2464E4	2.[illegible]
G.NLYQFGEMINQK.T	Y	69.45	1483.7129	12	0.2	742.8639	2	50.67	7	F7:15227	17112021_RID_2349_DP7.raw	4.284E5		1.[illegible]
N.LYQFGEM(+15.99)INQK.T	Y	66.62	1385.6649	11	0.4	693.8400	2	39.94	7	F7:11327	17112021_RID_2349_DP7.raw			
N.LYQFGEMINQK.T	Y	66.11	1369.6700	11	0.2	685.8424	2	47.17	7	F7:13715	17112021_RID_2349_DP7.raw			
E.GNLYQFGEMINQK.T	Y	61.39	1540.7344	13	0.5	771.3749	2	54.08	7	F7:16198	17112021_RID_2349_DP7.raw			
L.YQFGEMINQK.T	Y	61.03	1256.5859	10	0.0	629.3002	2	42.06	7	F7:12071	17112021_RID_2349_DP7.raw			
K.TATYSYSFENGDIV.C	Y	54.07	1565.6885	14	0.6	783.8520	2	57.78	7	F7:17385	17112021_RID_2349_DP7.raw			
R.ENMNTYDK.K	N	52.21	1013.4124	8	0.7	507.7138	2	19.60	7	F7:4282	17112021_RID_2349_DP7.raw			
K.GKPQDATDR.C	N	48.61	986.4781	9	-0.5	494.2461	2	11.50	7	F7:1839	17112021_RID_2349_DP7.raw			
C.FRENMNTYDKK.Y	Y	45.46	1444.6769	11	-0.4	482.5660	3	21.59	7	F7:4995	17112021_RID_2349_DP7.raw			
R.ENMNTYDKK.Y	N	43.49	1141.5073	9	-0.1	571.7609	2	15.11	7	F7:2868	17112021_RID_2349_DP7.raw			
K.KYMLYSIFD.C	N	43.04	1178.5681	9	0.2	590.2914	2	60.72	7	F7:18383	17112021_RID_2349_DP7.raw			

tota

Peptide	Uniq	-10lgP	Mass	Length	ppm	m/z	z	RT	Fraction	Scan	Source File	Area F1	Area F2	A
R.ENMNTYDKKY.M	Y	42.65	1304.5707	10	-0.7	653.2922	2	23.23	7	F7:5555	17112021_RID_2349_DP7.raw			
Q.FGEMINQK.T	Y	39.53	965.4641	8	-0.3	483.7391	2	31.06	7	F7:8248	17112021_RID_2349_DP7.raw			
C.FRENMNTYDK.K	Y	39.33	1316.5819	10	0.4	659.2985	2	24.51	7	F7:6037	17112021_RID_2349_DP7.raw			

tota

TRINITY_DN64
back to list

| Protein Coverage | Supporting Peptides |
Protein Coverage:

1 WSAYDQHCYK **AFDEPKR**SGD AEKFCTEQAN GGHLVSIESV EEAE**FVAQLI SENIKTSADY VWIGLWNQR**K AQYCISK**WTD**

81 **GSSVIYKNVI ER**FIKNCFGL EKQTNYRTWF NLSCGDDYPF VCKFPPRC

Supporting Peptides:

Peptide	Uniq	-10lgP	Mass	Length	ppm	m/z	z	RT	Fraction	Scan	Source File	Area F1	Area F2	Area
K.TSADYVWIGLWNQR.K	Y	81.64	1707.8369	14	0.9	854.9265	2	65.28	5	F5:19507	17112021_RID_2349_DP5.raw			5.8246
K.WTDGSSVIYK.N	N	77.27	1154.5608	10	0.0	578.2877	2	37.40	5	F5:10403	17112021_RID_2349_DP5.raw	2.1576E5	2.5151E5	1.7167
K.WTDGSSVIYKNVIER.F	N	71.65	1765.8999	15	0.3	883.9575	2	44.92	5	F5:12969	17112021_RID_2349_DP5.raw			
F.VAQLISENIK.T	N	48.99	1113.6394	10	-0.4	557.8268	2	37.53	5	F5:10425	17112021_RID_2349_DP5.raw			
W.TDGSSVIYK.N	N	44.54	968.4814	9	0.1	485.2480	2	26.37	5	F5:6664	17112021_RID_2349_DP5.raw			
E.FVAQLISENIK.T	N	44.08	1260.7078	11	0.3	631.3613	2	49.39	5	F5:14370	17112021_RID_2349_DP5.raw			
K.AFDEPKR.S	N	40.54	861.4344	7	-0.5	431.7243	2	18.04	5	F5:3641	17112021_RID_2349_DP5.raw			
V.AQLISENIK.T	N	39.19	1014.5709	9	0.0	508.2927	2	33.55	5	F5:9140	17112021_RID_2349_DP5.raw			

total 8 pe

B4XSY9.1
back to list

| Protein Coverage | Supporting Peptides |
Protein Coverage:

1 MGRFIFVRVG LLVVFLSLSG TGADFDCPPD WSAYDQHCYK **AFDEPKR**SGD AEKFCTQQAN GGHLVSIESV EEAE**FVAQLI**

81 **SENIKTSADY VWIGLWNQR**K APYCVSK**WTD GSSVIYKNVI ER**FIKNCFGL EKETNYRTWF NLSCGDDYPF VCKSPA

Supporting Peptides:

Peptide	Uniq	-10lgP	Mass	Length	ppm	m/z	z	RT	Fraction	Scan	Source File	Area F1	Area F2	Area
K.TSADYVWIGLWNQR.K	Y	81.64	1707.8369	14	0.9	854.9265	2	65.28	5	F5:19507	17112021_RID_2349_DP5.raw			5.8246
K.WTDGSSVIYK.N	N	77.27	1154.5608	10	0.0	578.2877	2	37.40	5	F5:10403	17112021_RID_2349_DP5.raw	2.1576E5	2.5151E5	1.7167
K.WTDGSSVIYKNVIER.F	N	71.65	1765.8999	15	0.3	883.9575	2	44.92	5	F5:12969	17112021_RID_2349_DP5.raw			
F.VAQLISENIK.T	N	48.99	1113.6394	10	-0.4	557.8268	2	37.53	5	F5:10425	17112021_RID_2349_DP5.raw			
W.TDGSSVIYK.N	N	44.54	968.4814	9	0.1	485.2480	2	26.37	5	F5:6664	17112021_RID_2349_DP5.raw			
E.FVAQLISENIK.T	N	44.08	1260.7078	11	0.3	631.3613	2	49.39	5	F5:14370	17112021_RID_2349_DP5.raw			
K.AFDEPKR.S	N	40.54	861.4344	7	-0.5	431.7243	2	18.04	5	F5:3641	17112021_RID_2349_DP5.raw			
V.AQLISENIK.T	N	39.19	1014.5709	9	0.0	508.2927	2	33.55	5	F5:9140	17112021_RID_2349_DP5.raw			

total 8 pe

ABW82661.1
back to list

| Protein Coverage | Supporting Peptides |
Protein Coverage:

1 MGRFIFVRVG LLVVFLSLSG TGADFDCPPD WSAYDQHCYK **AFDEPKR**SGD AEKFCTQQAN GGHLVSIESV EEAE**FVAQLI**

81 **SENIKTSADY VWIGLWNQR**K APYCVSK**WTD GSSVIYKNVI ER**FIKNCFGL EKETNYRTWF NLSCGDDYPF VCKSPA

Supporting Peptides:

Peptide	Uniq	-10lgP	Mass	Length	ppm	m/z	z	RT	Fraction	Scan	Source File	Area F1	Area F2	Area
K.TSADYVWIGLWNQR.K	Y	81.64	1707.8369	14	0.9	854.9265	2	65.28	5	F5:19507	17112021_RID_2349_DP5.raw			5.8246
K.WTDGSSVIYK.N	N	77.27	1154.5608	10	0.0	578.2877	2	37.40	5	F5:10403	17112021_RID_2349_DP5.raw	2.1576E5	2.5151E5	1.7167
K.WTDGSSVIYKNVIER.F	N	71.65	1765.8999	15	0.3	883.9575	2	44.92	5	F5:12969	17112021_RID_2349_DP5.raw			
F.VAQLISENIK.T	N	48.99	1113.6394	10	-0.4	557.8268	2	37.53	5	F5:10425	17112021_RID_2349_DP5.raw			
W.TDGSSVIYK.N	N	44.54	968.4814	9	0.1	485.2480	2	26.37	5	F5:6664	17112021_RID_2349_DP5.raw			
E.FVAQLISENIK.T	N	44.08	1260.7078	11	0.3	631.3613	2	49.39	5	F5:14370	17112021_RID_2349_DP5.raw			
K.AFDEPKR.S	N	40.54	861.4344	7	-0.5	431.7243	2	18.04	5	F5:3641	17112021_RID_2349_DP5.raw			
V.AQLISENIK.T	N	39.19	1014.5709	9	0.0	508.2927	2	33.55	5	F5:9140	17112021_RID_2349_DP5.raw			

total 8 pe

B4XSZ0.1
back to list

| Protein Coverage | Supporting Peptides |
Protein Coverage:

1 MGRFIFVRFG LLVVFLSLSG TGADFDCPPD WSAYDQHCYK **AFDEPKR**SGD AEKFCTQQAN GGHLVSIESV EEAE**FVAQLI**

81 **SENIKTSADY VWIGLWNQR**K APYCVSK**WTD GSSVIYKNVI ER**FIKNCFGL EKETNYRTWF NLSCGDDYPF VCKSPA

Supporting Peptides:

Peptide	Uniq	-10lgP	Mass	Length	ppm	m/z	z	RT	Fraction	Scan	Source File	Area F1	Area F2	Area
K.TSADYVWIGLWNQR.K	Y	81.64	1707.8369	14	0.9	854.9265	2	65.28	5	F5:19507	17112021_RID_2349_DP5.raw			5.8246
K.WTDGSSVIYK.N	N	77.27	1154.5608	10	0.0	578.2877	2	37.40	5	F5:10403	17112021_RID_2349_DP5.raw	2.1576E5	2.5151E5	1.7167
K.WTDGSSVIYKNVIER.F	N	71.65	1765.8999	15	0.3	883.9575	2	44.92	5	F5:12969	17112021_RID_2349_DP5.raw			
F.VAQLISENIK.T	N	48.99	1113.6394	10	-0.4	557.8268	2	37.53	5	F5:10425	17112021_RID_2349_DP5.raw			
W.TDGSSVIYK.N	N	44.54	968.4814	9	0.1	485.2480	2	26.37	5	F5:6664	17112021_RID_2349_DP5.raw			
E.FVAQLISENIK.T	N	44.08	1260.7078	11	0.3	631.3613	2	49.39	5	F5:14370	17112021_RID_2349_DP5.raw			
K.AFDEPKR.S	N	40.54	861.4344	7	-0.5	431.7243	2	18.04	5	F5:3641	17112021_RID_2349_DP5.raw			
V.AQLISENIK.T	N	39.19	1014.5709	9	0.0	508.2927	2	33.55	5	F5:9140	17112021_RID_2349_DP5.raw			

total 8 pe

ABW82662.1
back to list

| Protein Coverage | Supporting Peptides |
Protein Coverage:

1 MGRFIFVRFG LLVVFLSLSG TGADFDCPPD WSAYDQHCYK **AFDEPKR**SGD AEKFCTQQAN GGHLVSIESV EEAE**FVAQLI**

81 **SENIKTSADY VWIGLWNQR**K APYCVSK**WTD GSSVIYKNVI ER**FIKNCFGL EKETNYRTWF NLSCGDDYPF VCKSPA

Supporting Peptides:

Peptide	Uniq	-10lgP	Mass	Length	ppm	m/z	z	RT	Fraction	Scan	Source File	Area F1	Area F2	Area
K.TSADYVWIGLWNQR.K	Y	81.64	1707.8369	14	0.9	854.9265	2	65.28	5	F5:19507	17112021_RID_2349_DP5.raw			5.8246
K.WTDGSSVIYK.N	N	77.27	1154.5608	10	0.0	578.2877	2	37.40	5	F5:10403	17112021_RID_2349_DP5.raw	2.1576E5	2.5151E5	1.7167
K.WTDGSSVIYKNVIER.F	N	71.65	1765.8999	15	0.3	883.9575	2	44.92	5	F5:12969	17112021_RID_2349_DP5.raw			

total 8 pe

Peptide	Uniq	-10lgP	Mass	Length	ppm	m/z	z	RT	Fraction	Scan	Source File	Area F1	Area F2	Area
F.VAQLISENIK.T	N	48.99	1113.6394	10	-0.4	557.8268	2	37.53	5	F5:10425	17112021_RID_2349_DP5.raw			
W.TDGSSVIYK.N	N	44.54	968.4814	9	0.1	485.2480	2	26.37	5	F5:6664	17112021_RID_2349_DP5.raw			
E.FVAQLISENIK.T	N	44.08	1260.7078	11	0.3	631.3613	2	49.39	5	F5:14370	17112021_RID_2349_DP5.raw			
K.AFDEPKR.S	N	40.54	861.4344	7	-0.5	431.7243	2	18.04	5	F5:3641	17112021_RID_2349_DP5.raw			
V.AQLISENIK.T	N	39.19	1014.5709	9	0.0	508.2927	2	33.55	5	F5:9140	17112021_RID_2349_DP5.raw			

total 8 pe[illegible]

TRINITY_DN91
back to list

| Protein Coverage | Supporting Peptides |
Protein Coverage:

```
  1 MAKGFFISKS LGVVGIVLGL GAVATIIALS VVYSQEKQRA DELANQAPSS TSVAGTPSAT IAGTAQATTT NGTIQATTVK
 81 PLEPWDHMRL PKTLMPTFYT ISLQPFLIPD LYIFKGNSTV EFQCKQPTDL ILIHSKKLNY TIQGPFLVSL TGTKVVAPAI
161 DSTWLEDKTE YLVVKLKENL QQNENYQLHA SFTGELADDL AGFYRSEYTE GVARKVLATT QMQAADARKA FPCFDEPAMK
241 ANFSITLIHL PTYKALSNMP IKKAEEVKMP DGSTWVQTEF HPTLKMSTYL LGFIVSEFEN VSTIENNTLI QIWGRPTAIR
321 EGQGNYALNA TWPILSFFEQ QYSVPYPLTR LDQVALPDFN AGAMENWGLI TYRESALLFD EKYSSIGNKE RIVTVIAHEV
401 AHQWFGNLVT LEWWNELWLN EGFASYVEYL GAHEAESTWN IRDLIVPNDV YRVMAIDALA SSHPLSSPAE EINTPAQISE
481 VFDAISYSKG AAVIRMLAEF LTDAVFREGL QTYFETYQYG NTVCDDLWEQ LQKAVSKKNV YLPSTVKTIM DRWTLQMGFP
561 VLTVDTSKGI ITQKHFLLDP ESPVERPSQF NYVWIVPVSW LSKGKEAEMY WLTDILAENA NFSTSTDPAQ WLLLNVNVTG
641 YFRVNYDLGN WQRLMNQLNE DLQTIPVLNR AQIIDDAFNL ARAKHVGTDL ALNTTRYLAQ ERDYLPWDAA LDNLDYFRLM
721 FDRSEVYGPM QKYIRKQVTP LFDHFRNLTH NWTQIPDGLM NQYNQILAIR TACSHGVPGC NELASSWFEA WKNNPNVNDI
801 SPNLRSAVYC SAIRTGSLED WDFAWKMFRQ APVISEADKL RSALTCSQTP WILQRYLQYT LDPSMIRRQD AMSTINSIAS
881 NVVGQSLAWD FVRMNWKTLF QQFGRSSFSF SSLIQSVTQR FASPFELQQL EQFKADNADV GFGSATRALE QALERTKANI
961 KWVAENKALV LKWFQDNS
```

Supporting Peptides:

Peptide	Uniq	-10lgP	Mass	Length	ppm	m/z	z	RT	Fraction	Scan	Source File	Area F1	Area F2	Area F3	Area F4
R.AQIIDDAFNLAR.A	N	93.46	1345.6990	12	0.6	673.8572	2	54.45	1	F1:16660	12112021_RID_2349_DP1.raw	1.3295E6			
R.VNYDLGNWQR.L	Y	60.42	1263.5996	10	1.0	632.8077	2	44.31	1	F1:13320	12112021_RID_2349_DP1.raw	6.6887E4			
R.YLQYTLDPSMIR.R	Y	59.84	1498.7490	12	0.1	750.3818	2	54.59	1	F1:16715	12112021_RID_2349_DP1.raw	2.8801E5			
K.ADNADVGFGSATR.A	N	56.38	1279.5792	13	0.7	640.7974	2	31.66	1	F1:9065	12112021_RID_2349_DP1.raw	7.2844E4			
K.TLFQQFGR.S	Y	50.21	995.5189	8	0.2	498.7668	2	44.91	1	F1:13507	12112021_RID_2349_DP1.raw	1.1534E5			
K.NNPNVNDISPNLR.S	Y	47.74	1465.7273	13	1.2	733.8718	2	37.43	1	F1:11020	12112021_RID_2349_DP1.raw	4.7098E4			
R.LMFDRSEVYGPMQK.Y	N	39.50	1699.8062	14	0.1	567.6094	3	44.88	1	F1:13512	12112021_RID_2349_DP1.raw	8.2001E4			

total 7 peptides

TRINITY_DN91
back to list

| Protein Coverage | Supporting Peptides |
Protein Coverage:

1 MAKGFFISKS LGVVGIVLGL GAVATIIALS VVYSQEKQRA DELANQAPSS TSVAGTPSAT IAGTAQATTT NGTIQATTVK
81 PLEPWDHMRL PKTLMPTFYT ISLQPFLIPD LYIFKGNSTV EFQCKQPTDL ILIHSKKLNY TIQGPFLVSL TGTKVVAPAI
161 DSTWLEDKTE YLVVKLKENL QQNENYQLHA SFTGELADDL AGFYRSEYTE GVARKVLATT QMQAADARKA FPCFDEPAMK
241 ANFSITLIHL PTYKALSNMP IKKAEEVKMP DGSTWVQTEF HPTLKMSTYL LGFIVSEFEN VSTIENNTLI QIWGRPTAIR
321 EGQGNYALNA TWPILSFFEQ QYSVPYPLTR LDQVALPDFN AGAMENWGLI TYRESALLFD EKYSSIGNKE RIVTVIAHEV
401 AHQWFGNLVT LEWWNELWLN EGFASYVEYL GAHEAESTWN IRDLIVPNDV YRVMAIDALA SSHPLSSPAE EINTPAQISE
481 VFDAISYSKG AAVIRMLAEF LTDAVFREGL QTYFETYQYG NTVCDDLWEQ LQKAVSKKNV YLPSTVKTIM DRWTLQMGFP
561 VLTVDTSKGI ITQKHFLLDP ESPVERPSQF NYVWIVPVSW LSKGKEAEMY WLTDILAENA NFSTSTDPAQ WLLLNVNVTG
641 YFR**VNYDLGN WQR**LMNQLNE DLQTIPVLNR **AQIIDDAFNL AR**AKHVGTDL ALNTTRYLAQ ERDYLPWDAA LDNLDYFR**LM**
721 **FDRSEVYGPM QK**YIRKQVTP LFDHFRNLTH NWTQIPDGLM NQYNQILAIR TACSHGVPGC NELASSWFEA WK**NNPNVNDI**
801 **SPNLR**SAVYC SAIRTGSLED WDFAWKMFRQ APVISEADKL RSALTCSQTP WILQR**YLQYT LDPSMIR**RQD AMSTINSIAS
881 NVVGQSLAWD FVRMNWK**TLF QQFGR**SSFSF SSLIQSVTQR FASPFELQQL EQFK**ADNADV GFGSATR**ALE QALERTKANI
961 KWVAENKALV LKWFQDNS

Supporting Peptides:

Peptide	Uniq	-10lgP	Mass	Length	ppm	m/z	z	RT	Fraction	Scan	Source File	Area F1	Area F2	Area F3	Area F4
R.AQIIDDAFNLAR.A	N	93.46	1345.6990	12	0.6	673.8572	2	54.45	1	F1:16660	12112021_RID_2349_DP1.raw	1.3295E6			
R.VNYDLGNWQR.L	Y	60.42	1263.5996	10	1.0	632.8077	2	44.31	1	F1:13320	12112021_RID_2349_DP1.raw	6.6887E4			
R.YLQYTLDPSMIR.R	Y	59.84	1498.7490	12	0.1	750.3818	2	54.59	1	F1:16715	12112021_RID_2349_DP1.raw	2.8801E5			
K.ADNADVGFGSATR.A	N	56.38	1279.5792	13	0.7	640.7974	2	31.66	1	F1:9065	12112021_RID_2349_DP1.raw	7.2844E4			
K.TLFQQFGR.S	Y	50.21	995.5189	8	0.2	498.7668	2	44.91	1	F1:13507	12112021_RID_2349_DP1.raw	1.1534E5			
K.NNPNVNDISPNLR.S	Y	47.74	1465.7273	13	1.2	733.8718	2	37.43	1	F1:11020	12112021_RID_2349_DP1.raw	4.7098E4			
R.LMFDRSEVYGPMQK.Y	N	39.50	1699.8062	14	0.1	567.6094	3	44.88	1	F1:13512	12112021_RID_2349_DP1.raw	8.2001E4			

total 7 peptides

TRINITY_DN91
back to list

| Protein Coverage | Supporting Peptides |
Protein Coverage:

1 MAKGFFISKS LGVVGIVLGL GAVATIIALS VVYSQEKQRA DELANQAPSS TSVAGTPSAT IAGTAQATTT NGTIQATTVK
81 PLEPWDHMRL PKTLMPTFYT ISLQPFLIPD LYIFKGNSTV EFQCKQPTDL ILIHSKKLNY TIQGPFLVSL TGTKVVAPAI
161 DSTWLEDKTE YLVVKLKENL QQNENYQLHA SFTGELADDL AGFYRSEYTE GVARKVLATT QMQAADARKA FPCFDEPAMK
241 ANFSITLIHL PTYKALSNMP IKKAEEVKMP DGSTWVQTEF HPTLKMSTYL LGFIVSEFEN VSTIENNTLI QIWGRPTAIR
321 EGQGNYALNA TWPILSFFEQ QYSVPYPLTR LDQVALPDFN AGAMENWGLI TYRESALLFD EKYSSIGNKE RIVTVIAHEV
401 AHQWFGNLVT LEWWNELWLN EGFASYVEYL GAHEAESTWN IRDLIVPNDV YRVMAIDALA SSHPLSSPAE EINTPAQISE
481 VFDAISYSKG AAVIRMLAEF LTDAVFREGL QTYFETYQYG NTVCDDLWEQ LQKAVSKKNV YLPSTVKTIM DRWTLQMGFP
561 VLTVDTSKGI ITQKHFLLDP ESPVERPSQF NYVWIVPVSW LSKGKEAEMY WLTDILAENA NFSTSTDPAQ WLLLNVNVTG
641 YFR**VNYDLGN WQR**LMNQLNE DLQTIPVLNR **AQIIDDAFNL AR**AKHVGTDL ALNTTRYLAQ ERDYLPWDAA LDNLDYFR**LM**
721 **FDRSEVYGPM QK**YIRKQVTP LFDHFRNLTH NWTQIPDGLM NQYNQILAIR TACSHGVPGC NELASSWFEA WK**NNPNVNDI**
801 **SPNLR**SAVYC SAIRTGSLED WDFAWKMFRQ APVISEADKL RSALTCSQTP WILQR**YLQYT LDPSMIR**RQD AMSTINSIAS
881 NVVGQSLAWD FVRMNWK**TLF QQFGR**SSFSF SSLIQSVTQR FASPFELQQL EQFK**ADNADV GFGSATR**ALE QALERTKANI
961 KWVAENKALV LKWFQDNS

Supporting Peptides:

Peptide	Uniq	-10lgP	Mass	Length	ppm	m/z	z	RT	Fraction	Scan	Source File	Area F1	Area F2	Area F3	Area F4
R.AQIIDDAFNLAR.A	N	93.46	1345.6990	12	0.6	673.8572	2	54.45	1	F1:16660	12112021_RID_2349_DP1.raw	1.3295E6			
R.VNYDLGNWQR.L	Y	60.42	1263.5996	10	1.0	632.8077	2	44.31	1	F1:13320	12112021_RID_2349_DP1.raw	6.6887E4			
R.YLQYTLDPSMIR.R	Y	59.84	1498.7490	12	0.1	750.3818	2	54.59	1	F1:16715	12112021_RID_2349_DP1.raw	2.8801E5			
K.ADNADVGFGSATR.A	N	56.38	1279.5792	13	0.7	640.7974	2	31.66	1	F1:9065	12112021_RID_2349_DP1.raw	7.2844E4			
K.TLFQQFGR.S	Y	50.21	995.5189	8	0.2	498.7668	2	44.91	1	F1:13507	12112021_RID_2349_DP1.raw	1.1534E5			

total 7 peptides

Peptide	Uniq	-10lgP	Mass	Length	ppm	m/z	z	RT	Fraction	Scan	Source File	Area F1	Area F2	Area F3	Area F4
K.NNPNVNDISPNLR.S	Y	47.74	1465.7273	13	1.2	733.8718	2	37.43	1	F1:11020	12112021_RID_2349_DP1.raw	4.7098E4			
R.LMFDRSEVYGPMQK.Y	N	39.50	1699.8062	14	0.1	567.6094	3	44.88	1	F1:13512	12112021_RID_2349_DP1.raw	8.2001E4			

total 7 peptides

TRINITY_DN91
back to list

| Protein Coverage | Supporting Peptides |
Protein Coverage:

1 MAKGFFISKS LGVVGIVLGL GAVATIIALS VVYSQEKQRA DELANQAPSS TSVAGTPSAT IAGTAQATTT NGTIQATTVK
81 PLEPWDHMRL PKTLMPTFYT ISLQPFLIPD LYIFKGNSTV EFQCKQPTDL ILIHSKKLNY TIQGPFLVSL TGTKVVAPAI
161 DSTWLEDKTE YLVVKLKENL QQNENYQLHA SFTGELADDL AGFYRSEYTE GVARKVLATT QMQAADARKA FPCFDEPAMK
241 ANFSITLIHL PTYKALSNMP IKKAEEVKMP DGSTWVQTEF HPTLKMSTYL LGFIVSEFEN VSTIENNTLI QIWGRPTAIR
321 EGQGNYALNA TWPILSFFEQ QYSVPYPLTR LDQVALPDFN AGAMENWGLI TYRESALLFD EKYSSIGNKE RIVTVIAHEV
401 AHQWFGNLVT LEWWNELWLN EGFASYVEYL GAHEAESTWN IRDLIVPNDV YRVMAIDALA SSHPLSSPAE EINTPAQISE
481 VFDAISYSKG AAVIRMLAEF LTDAVFREGL QTYFETYQYG NTVCDDLWEQ LQKAVSKKNV YLPSTVKTIM DRWTLQMGFP
561 VLTVDTSKGI ITQKHFLLDP ESPVERPSQF NYVWIVPVSW LSKGKEAEMY WLTDILAENA NFSTSTDPAQ WLLLNVNVTG
641 YFR**VNYDLGN WQR**LMNQLNE DLQTIPVLNR **AQIIDDAFNL AR**AKHVGTDL ALNTTRYLAQ ERDYLPWDAA LDNLDYFR**LM**
721 **FDRSEVYGPM QK**YIRKQVTP LFDHFRNLTH NWTQIPDGLM NQYNQILAIR TACSHGVPGC NELASSWFEA WK**NNPNVNDI**
801 **SPNLR**SAVYC SAIRTGSLED WDFAWKMFRQ APVISEADKL RSALTCSQTP WILQR**YLQYT LDPSMIR**RQD AMSTINSIAS
881 NVVGQSLAWD FVRMNWK**TLF QQFGR**SSFSF SSLIQSVTQR FASPFELQQL EQFK**ADNADV GFGSATR**ALE QALERTKANI
961 KWVAENKALV LKWFQDNS

Supporting Peptides:

Peptide	Uniq	-10lgP	Mass	Length	ppm	m/z	z	RT	Fraction	Scan	Source File	Area F1	Area F2	Area F3	Area F4
R.AQIIDDAFNLAR.A	N	93.46	1345.6990	12	0.6	673.8572	2	54.45	1	F1:16660	12112021_RID_2349_DP1.raw	1.3295E6			
R.VNYDLGNWQR.L	Y	60.42	1263.5996	10	1.0	632.8077	2	44.31	1	F1:13320	12112021_RID_2349_DP1.raw	6.6887E4			
R.YLQYTLDPSMIR.R	Y	59.84	1498.7490	12	0.1	750.3818	2	54.59	1	F1:16715	12112021_RID_2349_DP1.raw	2.8801E5			
K.ADNADVGFGSATR.A	N	56.38	1279.5792	13	0.7	640.7974	2	31.66	1	F1:9065	12112021_RID_2349_DP1.raw	7.2844E4			
K.TLFQQFGR.S	Y	50.21	995.5189	8	0.2	498.7668	2	44.91	1	F1:13507	12112021_RID_2349_DP1.raw	1.1534E5			
K.NNPNVNDISPNLR.S	Y	47.74	1465.7273	13	1.2	733.8718	2	37.43	1	F1:11020	12112021_RID_2349_DP1.raw	4.7098E4			
R.LMFDRSEVYGPMQK.Y	N	39.50	1699.8062	14	0.1	567.6094	3	44.88	1	F1:13512	12112021_RID_2349_DP1.raw	8.2001E4			

total 7 peptides

TRINITY_DN91
back to list

| Protein Coverage | Supporting Peptides |
Protein Coverage:

1 MAKGFFISKS LGVVGIVLGL GAVATIIALS VVYSQEKQRA DELANQAPSS TSVAGTPSAT IAGTAQATTT NGTIQATTVK
81 PLEPWDHMRL PKTLMPTFYT ISLQPFLIPD LYIFKGNSTV EFQCKQPTDL ILIHSKKLNY TIQGPFLVSL TGTKVVAPAI
161 DSTWLEDKTE YLVVKLKENL QQNENYQLHA SFTGELADDL AGFYRSEYTE GVARKVLATT QMQAADARKA FPCFDEPAMK
241 ANFSITLIHL PTYKALSNMP IKKAEEVKMP DGSTWVQTEF HPTLKMSTYL LGFIVSEFEN VSTIENNTLI QIWGRPTAIR
321 EGQGNYALNA TWPILSFFEQ QYSVPYPLTR LDQVALPDFN AGAMENWGLI TYRESALLFD EKYSSIGNKE RIVTVIAHEV
401 AHQWFGNLVT LEWWNELWLN EGFASYVEYL GAHEAESTWN IRDLIVPNDV YRVMAIDALA SSHPLSSPAE EINTPAQISE
481 VFDAISYSKG AAVIRMLAEF LTDAVFREGL QTYFETYQYG NTVCDDLWEQ LQKAVSKKNV YLPSTVKTIM DRWTLQMGFP
561 VLTVDTSKGI ITQKHFLLDP ESPVERPSQF NYVWIVPVSW LSKGKEAEMY WLTDILAENA NFSTSTDPAQ WLLLNVNVTG
641 YFR**VNYDLGN WQR**LMNQLNE DLQTIPVLNR **AQIIDDAFNL AR**AKHVGTDL ALNTTRYLAQ ERDYLPWDAA LDNLDYFR**LM**
721 **FDRSEVYGPM QK**YIRKQVTP LFDHFRNLTH NWTQIPDGLM NQYNQILAIR TACSHGVPGC NELASSWFEA WK**NNPNVNDI**
801 **SPNLR**SAVYC SAIRTGSLED WDFAWKMFRQ APVISEADKL RSALTCSQTP WILQR**YLQYT LDPSMIR**RQD AMSTINSIAS
881 NVVGQSLAWD FVRMNWK**TLF QQFGR**SSFSF SSLIQSVTQR FASPFELQQL EQFK**ADNADV GFGSATR**ALE QALERTKANI
961 KWVAENKALV LKWFQDNS

Supporting Peptides:

Peptide	Uniq	-10lgP	Mass	Length	ppm	m/z	z	RT	Fraction	Scan	Source File	Area F1	Area F2	Area F3	Area F4

Peptide	Uniq	-10lgP	Mass	Length	ppm	m/z	z	RT	Fraction	Scan	Source File	Area F1	Area F2	Area F3	Area F4
R.AQIIDDAFNLAR.A	N	93.46	1345.6990	12	0.6	673.8572	2	54.45	1	F1:16660	12112021_RID_2349_DP1.raw	1.3295E6			
R.VNYDLGNWQR.L	Y	60.42	1263.5996	10	1.0	632.8077	2	44.31	1	F1:13320	12112021_RID_2349_DP1.raw	6.6887E4			
R.YLQYTLDPSMIR.R	Y	59.84	1498.7490	12	0.1	750.3818	2	54.59	1	F1:16715	12112021_RID_2349_DP1.raw	2.8801E5			
K.ADNADVGFGSATR.A	N	56.38	1279.5792	13	0.7	640.7974	2	31.66	1	F1:9065	12112021_RID_2349_DP1.raw	7.2844E4			
K.TLFQQFGR.S	Y	50.21	995.5189	8	0.2	498.7668	2	44.91	1	F1:13507	12112021_RID_2349_DP1.raw	1.1534E5			
K.NNPNVNDISPNLR.S	Y	47.74	1465.7273	13	1.2	733.8718	2	37.43	1	F1:11020	12112021_RID_2349_DP1.raw	4.7098E4			
R.LMFDRSEVYGPMQK.Y	N	39.50	1699.8062	14	0.1	567.6094	3	44.88	1	F1:13512	12112021_RID_2349_DP1.raw	8.2001E4			

total 7 peptides

ETE57948.1
back to list

| Protein Coverage | Supporting Peptides |
Protein Coverage:

```
  1 MCKAGFAGDD APRAVFPSIV GRPRHQGVMV GMGQKDSYVG DEAQSKRGIL TLKYPIEHGI VTNWDDMEKI WHHTFYNELR
 81 VAPEEHPVLL TEAPLNPKAN REKMTQIMFE TFNTPAMYVA IQAVLSLYAS GRTTGIVMDS GDGVTHTVPI YEGYALPHAI
161 LRLDLAGRDL TDYLMKILTE RGYSFTTTAE REIVRDIKEK LCYVALDFEQ EMATAASSSS LEKSYELPDG QVITIGNERF
241 RCPEALFQPS FLGMESCGIH ETTFNSIMKC DVDIRKDLYA NTVLSGGTTM YPGIADRMQK EITALAPSTM KIKIIAPPER
321 KYSVWIGGSI LASLSTFQQM WISKQEYDES GPSIVHRKCF
```

Supporting Peptides:

Peptide	Uniq	-10lgP	Mass	Length	ppm	m/z	z	RT	Fraction	Scan	Source File	Area
R.GYSFTTTAER.E	Y	72.34	1131.5197	10	1.1	566.7677	2	33.52	2	F2:8772	12112021_RID_2349_DP2.raw	
K.SYELPDGQVITIGNER.F	Y	71.69	1789.8846	16	0.3	895.9499	2	53.88	2	F2:15567	12112021_RID_2349_DP2.raw	
K.DSYVGDEAQSK.R	Y	67.44	1197.5149	11	1.1	599.7654	2	23.86	2	F2:5370	12112021_RID_2349_DP2.raw	2.1509
R.VAPEEHPVLLTEAPLNPK.A	Y	63.91	1953.0570	18	0.6	977.5364	2	45.29	2	F2:12822	12112021_RID_2349_DP2.raw	
K.AGFAGDDAPR.A	Y	60.62	975.4410	10	0.5	488.7280	2	26.11	1	F1:7167	12112021_RID_2349_DP1.raw	3.3593
K.EITALAPSTMK.I	Y	52.44	1160.6111	11	0.0	581.3128	2	38.85	2	F2:10632	12112021_RID_2349_DP2.raw	
K.DSYVGDEAQSKR.G	Y	50.46	1353.6160	12	0.4	677.8156	2	20.64	2	F2:4244	12112021_RID_2349_DP2.raw	
K.EITALAPSTM(+15.99)K.I	Y	47.42	1176.6060	11	0.9	589.3108	2	32.91	2	F2:8537	12112021_RID_2349_DP2.raw	
R.AVFPSIVGRPR.H	Y	44.25	1197.6981	11	1.0	599.8569	2	40.62	2	F2:11273	12112021_RID_2349_DP2.raw	
K.DLYANTVLSGGTTM(+15.99)YPGIADR.M	Y	41.30	2230.0576	21	-0.2	1116.0359	2	56.05	2	F2:16292	12112021_RID_2349_DP2.raw	

JAC96363.1
back to list

| Protein Coverage | Supporting Peptides |
Protein Coverage:

```
  1 MEEEIAALVI DNGSGMCKAG FAGDDAPRAV FPSIVGRPRH QGVMVGMGQK DSYVGDEAQS KRGILTLKYP IEHGIVTNWD
 81 DMEKIWHHTF YNELRVAPEE HPVLLTEAPL NPKANREKMT QIMFETFNTP AMYVAIQAVL SLYASGRTTG IVMDSGDGVT
161 HTVPIYEGYA LPHAILRLDL AGRDLTDYLM KILTERGYSF TTTAEREIVR DIKEKLCYVA LDFEQEMATA ASSSSLEKSY
241 ELPDGQVITI GNERFRCPEA LFQPSFLGME SCGIHETTFN SIMKCDVDIR KDLYANTVLS GGTTMYPGIA DRMQKEITAL
321 APSTMKIKII APPERKYSVW IGGSILASLS TFQQMWISKQ EYDESGPSIV HRKCF
```

Supporting Peptides:

Peptide	Uniq	-10lgP	Mass	Length	ppm	m/z	z	RT	Fraction	Scan	Source File	Area

Peptide	Uniq	-10lgP	Mass	Length	ppm	m/z	z	RT	Fraction	Scan	Source File	Area
R.GYSFTTTAER.E	Y	72.34	1131.5197	10	1.1	566.7677	2	33.52	2	F2:8772	12112021_RID_2349_DP2.raw	
K.SYELPDGQVITIGNER.F	Y	71.69	1789.8846	16	0.3	895.9499	2	53.88	2	F2:15567	12112021_RID_2349_DP2.raw	
K.DSYVGDEAQSK.R	Y	67.44	1197.5149	11	1.1	599.7654	2	23.86	2	F2:5370	12112021_RID_2349_DP2.raw	2.1509
R.VAPEEHPVLLTEAPLNPK.A	Y	63.91	1953.0570	18	0.6	977.5364	2	45.29	2	F2:12822	12112021_RID_2349_DP2.raw	
K.AGFAGDDAPR.A	Y	60.62	975.4410	10	0.5	488.7280	2	26.11	1	F1:7167	12112021_RID_2349_DP1.raw	3.3593
K.EITALAPSTMK.I	Y	52.44	1160.6111	11	0.0	581.3128	2	38.85	2	F2:10632	12112021_RID_2349_DP2.raw	
K.DSYVGDEAQSKR.G	Y	50.46	1353.6160	12	0.4	677.8156	2	20.64	2	F2:4244	12112021_RID_2349_DP2.raw	
K.EITALAPSTM(+15.99)K.I	Y	47.42	1176.6060	11	0.9	589.3108	2	32.91	2	F2:8537	12112021_RID_2349_DP2.raw	
R.AVFPSIVGRPR.H	Y	44.25	1197.6981	11	1.0	599.8569	2	40.62	2	F2:11273	12112021_RID_2349_DP2.raw	
K.DLYANTVLSGGTTM(+15.99)YPGIADR.M	Y	41.30	2230.0576	21	-0.2	1116.0359	2	56.05	2	F2:16292	12112021_RID_2349_DP2.raw	

JAA97900.1
back to list

| Protein Coverage | Supporting Peptides |
Protein Coverage:

1 MEEEIAALVI DNGSGMCKAG FAGDDAPRAV FPSIVGRPRH QGVMVGMGQK DSYVGDEAQS KRGILTLKYP IEHGIVTNWD

81 DMEKIWHHTF YNELRVAPEE HPVLLTEAPL NPKANREKMT QIMFETFNTP AMYVAIQAVL SLYASGRTTG IVMDSGDGVT

161 HTVPIYEGYA LPHAILRLDL AGRDLTDYLM KILTERGYSF TTTAEREIVR DIKEKLCYVA LDFEQEMATA ASSSSLEKSY

241 ELPDGQVITI GNERFRCPEA LFQPSFLGME SCGIHETTFN SIMKCDVDIR KDLYANTVLS GGTTMYPGIA DRMQKEITAL

321 APSTMKIKII APPERKYSVW IGGSILASLS TFQQMWISKQ EYDESGPSIV HRKCF

Supporting Peptides:

Peptide	Uniq	-10lgP	Mass	Length	ppm	m/z	z	RT	Fraction	Scan	Source File	Area
R.GYSFTTTAER.E	Y	72.34	1131.5197	10	1.1	566.7677	2	33.52	2	F2:8772	12112021_RID_2349_DP2.raw	
K.SYELPDGQVITIGNER.F	Y	71.69	1789.8846	16	0.3	895.9499	2	53.88	2	F2:15567	12112021_RID_2349_DP2.raw	
K.DSYVGDEAQSK.R	Y	67.44	1197.5149	11	1.1	599.7654	2	23.86	2	F2:5370	12112021_RID_2349_DP2.raw	2.1509
R.VAPEEHPVLLTEAPLNPK.A	Y	63.91	1953.0570	18	0.6	977.5364	2	45.29	2	F2:12822	12112021_RID_2349_DP2.raw	
K.AGFAGDDAPR.A	Y	60.62	975.4410	10	0.5	488.7280	2	26.11	1	F1:7167	12112021_RID_2349_DP1.raw	3.3593
K.EITALAPSTMK.I	Y	52.44	1160.6111	11	0.0	581.3128	2	38.85	2	F2:10632	12112021_RID_2349_DP2.raw	
K.DSYVGDEAQSKR.G	Y	50.46	1353.6160	12	0.4	677.8156	2	20.64	2	F2:4244	12112021_RID_2349_DP2.raw	
K.EITALAPSTM(+15.99)K.I	Y	47.42	1176.6060	11	0.9	589.3108	2	32.91	2	F2:8537	12112021_RID_2349_DP2.raw	
R.AVFPSIVGRPR.H	Y	44.25	1197.6981	11	1.0	599.8569	2	40.62	2	F2:11273	12112021_RID_2349_DP2.raw	
K.DLYANTVLSGGTTM(+15.99)YPGIADR.M	Y	41.30	2230.0576	21	-0.2	1116.0359	2	56.05	2	F2:16292	12112021_RID_2349_DP2.raw	

LAA21099.1
back to list

| Protein Coverage | Supporting Peptides |
Protein Coverage:

1 MEEEIAALVI DNGSGMCKAG FAGDDAPRAV FPSIVGRPRH QGVMVGMGQK DSYVGDEAQS KRGILTLKYP IEHGIVTNWD

81 DMEKIWHHTF YNELRVAPEE HPVLLTEAPL NPKANREKMT QIMFETFNTP AMYVAIQAVL SLYASGRTTG IVMDSGDGVT

161 HTVPIYEGYA LPHAILRLDL AGRDLTDYLM KILTERGYSF TTTAEREIVR DIKEKLCYVA LDFEQEMATA ASSSSLEKSY

241 ELPDGQVITI GNERFRCPEA LFQPSFLGME SCGIHETTFN SIMKCDVDIR KDLYANTVLS GGTTMYPGIA DRMQKEITAL

321 APSTMKIKII APPERKYSVW IGGSILASLS TFQQMWISKQ EYDESGPSIV HRKCF

Supporting Peptides:

Peptide	Uniq	-10lgP	Mass	Length	ppm	m/z	z	RT	Fraction	Scan	Source File	Area
R.GYSFTTTAER.E	Y	72.34	1131.5197	10	1.1	566.7677	2	33.52	2	F2:8772	12112021_RID_2349_DP2.raw	
K.SYELPDGQVITIGNER.F	Y	71.69	1789.8846	16	0.3	895.9499	2	53.88	2	F2:15567	12112021_RID_2349_DP2.raw	
K.DSYVGDEAQSK.R	Y	67.44	1197.5149	11	1.1	599.7654	2	23.86	2	F2:5370	12112021_RID_2349_DP2.raw	2.1509
R.VAPEEHPVLLTEAPLNPK.A	Y	63.91	1953.0570	18	0.6	977.5364	2	45.29	2	F2:12822	12112021_RID_2349_DP2.raw	
K.AGFAGDDAPR.A	Y	60.62	975.4410	10	0.5	488.7280	2	26.11	1	F1:7167	12112021_RID_2349_DP1.raw	3.3593
K.EITALAPSTMK.I	Y	52.44	1160.6111	11	0.0	581.3128	2	38.85	2	F2:10632	12112021_RID_2349_DP2.raw	
K.DSYVGDEAQSKR.G	Y	50.46	1353.6160	12	0.4	677.8156	2	20.64	2	F2:4244	12112021_RID_2349_DP2.raw	
K.EITALAPSTM(+15.99)K.I	Y	47.42	1176.6060	11	0.9	589.3108	2	32.91	2	F2:8537	12112021_RID_2349_DP2.raw	
R.AVFPSIVGRPR.H	Y	44.25	1197.6981	11	1.0	599.8569	2	40.62	2	F2:11273	12112021_RID_2349_DP2.raw	
K.DLYANTVLSGGTTM(+15.99)YPGIADR.M	Y	41.30	2230.0576	21	-0.2	1116.0359	2	56.05	2	F2:16292	12112021_RID_2349_DP2.raw	

JAI10342.1
back to list

| Protein Coverage | Supporting Peptides |
Protein Coverage:

1 MDDDIAALVV DNGSGMCKAG FAGDDAPRAV FPSIVGRPRH QGVMVGMGQK DSYVGDEAQS KRGILTLKYP IEHGIVTNWD

81 DMEKIWHHTF YNELRVAPEE HPVLLTEAPL NPKANREKMT QIMFETFNTP AMYVAIQAVL SLYASGRTTG IVMDSGDGVT

161 HTVPIYEGYA LPHAILRLDL AGRDLTDYLM KILTERGYSF TTTAEREIVR DIKEKLCYVA LDFEQEMATA ASSSSLEKSY

241 ELPDGQVITI GNERFRCPEA LFQPSFLGME SCGIHETTFN SIMKCDVDIR KDLYANTVLS GGTTMYPGIA DRMQKEITAL

321 APSTMKIKII APPERKYSVW IGGSILASLS TFQQMWISKQ EYDESGPSIV HRKCF

Supporting Peptides:

Peptide	Uniq	-10lgP	Mass	Length	ppm	m/z	z	RT	Fraction	Scan	Source File	Area
R.GYSFTTTAER.E	Y	72.34	1131.5197	10	1.1	566.7677	2	33.52	2	F2:8772	12112021_RID_2349_DP2.raw	
K.SYELPDGQVITIGNER.F	Y	71.69	1789.8846	16	0.3	895.9499	2	53.88	2	F2:15567	12112021_RID_2349_DP2.raw	
K.DSYVGDEAQSK.R	Y	67.44	1197.5149	11	1.1	599.7654	2	23.86	2	F2:5370	12112021_RID_2349_DP2.raw	2.1509
R.VAPEEHPVLLTEAPLNPK.A	Y	63.91	1953.0570	18	0.6	977.5364	2	45.29	2	F2:12822	12112021_RID_2349_DP2.raw	
K.AGFAGDDAPR.A	Y	60.62	975.4410	10	0.5	488.7280	2	26.11	1	F1:7167	12112021_RID_2349_DP1.raw	3.3593
K.EITALAPSTMK.I	Y	52.44	1160.6111	11	0.0	581.3128	2	38.85	2	F2:10632	12112021_RID_2349_DP2.raw	
K.DSYVGDEAQSKR.G	Y	50.46	1353.6160	12	0.4	677.8156	2	20.64	2	F2:4244	12112021_RID_2349_DP2.raw	
K.EITALAPSTM(+15.99)K.I	Y	47.42	1176.6060	11	0.9	589.3108	2	32.91	2	F2:8537	12112021_RID_2349_DP2.raw	
R.AVFPSIVGRPR.H	Y	44.25	1197.6981	11	1.0	599.8569	2	40.62	2	F2:11273	12112021_RID_2349_DP2.raw	
K.DLYANTVLSGGTTM(+15.99)YPGIADR.M	Y	41.30	2230.0576	21	-0.2	1116.0359	2	56.05	2	F2:16292	12112021_RID_2349_DP2.raw	

JAG68843.1
back to list

| Protein Coverage | Supporting Peptides |

Protein Coverage:

```
1   MEEEIAALVI DNGSGMCKAG FAGDDAPRAV FPSIVGRPRH QGVMVGMGQK DSYVGDEAQS KRGILTLKYP IEHGIVTNWD
81  DMEKIWHHTF YNELRVAPEE HPVLLTEAPL NPKANREKMT QIMFETFNTP AMYVAIQAVL SLYASGRTTG IVMDSGDGVT
161 HTVPIYEGYA LPHAILRLDL AGRDLTDYLM KILTERGYSF TTTAEREIVR DIKEKLCYVA LDFEQEMATA ASSSSLEKSY
241 ELPDGQVITI GNERFRCPEA LFQPSFLGME SCGIHETTFN SIMKCDVDIR KDLYANTVLS GGTTMYPGIA DRMQKEITAL
321 APSTMKIKII APPERKYSVW IGGSILASLS TFQQMWISKQ EYDESGPSIV HRKCF
```

Supporting Peptides:

Peptide	Uniq	-10lgP	Mass	Length	ppm	m/z	z	RT	Fraction	Scan	Source File	Area
R.GYSFTTTAER.E	Y	72.34	1131.5197	10	1.1	566.7677	2	33.52	2	F2:8772	12112021_RID_2349_DP2.raw	
K.SYELPDGQVITIGNER.F	Y	71.69	1789.8846	16	0.3	895.9499	2	53.88	2	F2:15567	12112021_RID_2349_DP2.raw	
K.DSYVGDEAQSK.R	Y	67.44	1197.5149	11	1.1	599.7654	2	23.86	2	F2:5370	12112021_RID_2349_DP2.raw	2.1509
R.VAPEEHPVLLTEAPLNPK.A	Y	63.91	1953.0570	18	0.6	977.5364	2	45.29	2	F2:12822	12112021_RID_2349_DP2.raw	
K.AGFAGDDAPR.A	Y	60.62	975.4410	10	0.5	488.7280	2	26.11	1	F1:7167	12112021_RID_2349_DP1.raw	3.3593
K.EITALAPSTMK.I	Y	52.44	1160.6111	11	0.0	581.3128	2	38.85	2	F2:10632	12112021_RID_2349_DP2.raw	
K.DSYVGDEAQSKR.G	Y	50.46	1353.6160	12	0.4	677.8156	2	20.64	2	F2:4244	12112021_RID_2349_DP2.raw	
K.EITALAPSTM(+15.99)K.I	Y	47.42	1176.6060	11	0.9	589.3108	2	32.91	2	F2:8537	12112021_RID_2349_DP2.raw	
R.AVFPSIVGRPR.H	Y	44.25	1197.6981	11	1.0	599.8569	2	40.62	2	F2:11273	12112021_RID_2349_DP2.raw	
K.DLYANTVLSGGTTM(+15.99)YPGIADR.M	Y	41.30	2230.0576	21	-0.2	1116.0359	2	56.05	2	F2:16292	12112021_RID_2349_DP2.raw	

XP_013921350.1

back to list

| Protein Coverage | Supporting Peptides |

Protein Coverage:

```
1   MEEEIAALVI DNGSGMCKAG FAGDDAPRAV FPSIVGRPRH QGVMVGMGQK DSYVGDEAQS KRGILTLKYP IEHGIVTNWD
81  DMEKIWHHTF YNELRVAPEE HPVLLTEAPL NPKANREKMT QIMFETFNTP AMYVAIQAVL SLYASGRTTG IVMDSGDGVT
161 HTVPIYEGYA LPHAILRLDL AGRDLTDYLM KILTERGYSF TTTAEREIVR DIKEKLCYVA LDFEQEMATA ASSSSLEKSY
241 ELPDGQVITI GNERFRCPEA LFQPSFLGME SCGIHETTFN SIMKCDVDIR KDLYANTVLS GGTTMYPGIA DRMQKEITAL
321 APSTMKIKII APPERKYSVW IGGSILASLS TFQQMWISKQ EYDESGPSIV HRKCF
```

Supporting Peptides:

Peptide	Uniq	-10lgP	Mass	Length	ppm	m/z	z	RT	Fraction	Scan	Source File	Area
R.GYSFTTTAER.E	Y	72.34	1131.5197	10	1.1	566.7677	2	33.52	2	F2:8772	12112021_RID_2349_DP2.raw	
K.SYELPDGQVITIGNER.F	Y	71.69	1789.8846	16	0.3	895.9499	2	53.88	2	F2:15567	12112021_RID_2349_DP2.raw	
K.DSYVGDEAQSK.R	Y	67.44	1197.5149	11	1.1	599.7654	2	23.86	2	F2:5370	12112021_RID_2349_DP2.raw	2.1509
R.VAPEEHPVLLTEAPLNPK.A	Y	63.91	1953.0570	18	0.6	977.5364	2	45.29	2	F2:12822	12112021_RID_2349_DP2.raw	
K.AGFAGDDAPR.A	Y	60.62	975.4410	10	0.5	488.7280	2	26.11	1	F1:7167	12112021_RID_2349_DP1.raw	3.3593
K.EITALAPSTMK.I	Y	52.44	1160.6111	11	0.0	581.3128	2	38.85	2	F2:10632	12112021_RID_2349_DP2.raw	
K.DSYVGDEAQSKR.G	Y	50.46	1353.6160	12	0.4	677.8156	2	20.64	2	F2:4244	12112021_RID_2349_DP2.raw	
K.EITALAPSTM(+15.99)K.I	Y	47.42	1176.6060	11	0.9	589.3108	2	32.91	2	F2:8537	12112021_RID_2349_DP2.raw	
R.AVFPSIVGRPR.H	Y	44.25	1197.6981	11	1.0	599.8569	2	40.62	2	F2:11273	12112021_RID_2349_DP2.raw	

Peptide	Uniq	-10lgP	Mass	Length	ppm	m/z	z	RT	Fraction	Scan	Source File	Area
K.DLYANTVLSGGTTM(+15.99)YPGIADR.M	Y	41.30	2230.0576	21	-0.2	1116.0359	2	56.05	2	F2:16292	12112021_RID_2349_DP2.raw	

JAB54771.1
back to list

| Protein Coverage | Supporting Peptides |
Protein Coverage:

```
  1 MDDDIAALVV DNGSGMCKAG FAGDDAPRAV FPSIVGRPRH QGVMVGMGQK DSYVGDEAQS KRGILTLKYP IEHGIVTNWD
 81 DMEKIWHHTF YNELRVAPEE HPVLLTEAPL NPKANREKMT QIMFETFNTP AMYVAIQAVL SLYASGRTTG IVMDSGDGVT
161 HTVPIYEGYA LPHAILRLDL AGRDLTDYLM KILTERGYSF TTTAEREIVR DIKEKLCYVA LDFEQEMATA ASSSSLEKSY
241 ELPDGQVITI GNERFRCPEA LFQPSFLGME SCGIHETTFN SIMKCDVDIR KDLYANTVLS GGTTMYPGIA DRMQKEITAL
321 APSTMKIKII APPERKYSVW IGGSILASLS TFQQMWISKQ EYDESGPSIV HRKCF
```

Supporting Peptides:

Peptide	Uniq	-10lgP	Mass	Length	ppm	m/z	z	RT	Fraction	Scan	Source File	Area
R.GYSFTTTAER.E	Y	72.34	1131.5197	10	1.1	566.7677	2	33.52	2	F2:8772	12112021_RID_2349_DP2.raw	
K.SYELPDGQVITIGNER.F	Y	71.69	1789.8846	16	0.3	895.9499	2	53.88	2	F2:15567	12112021_RID_2349_DP2.raw	
K.DSYVGDEAQSK.R	Y	67.44	1197.5149	11	1.1	599.7654	2	23.86	2	F2:5370	12112021_RID_2349_DP2.raw	2.1509
R.VAPEEHPVLLTEAPLNPK.A	Y	63.91	1953.0570	18	0.6	977.5364	2	45.29	2	F2:12822	12112021_RID_2349_DP2.raw	
K.AGFAGDDAPR.A	Y	60.62	975.4410	10	0.5	488.7280	2	26.11	1	F1:7167	12112021_RID_2349_DP1.raw	3.3593
K.EITALAPSTMK.I	Y	52.44	1160.6111	11	0.0	581.3128	2	38.85	2	F2:10632	12112021_RID_2349_DP2.raw	
K.DSYVGDEAQSKR.G	Y	50.46	1353.6160	12	0.4	677.8156	2	20.64	2	F2:4244	12112021_RID_2349_DP2.raw	
K.EITALAPSTM(+15.99)K.I	Y	47.42	1176.6060	11	0.9	589.3108	2	32.91	2	F2:8537	12112021_RID_2349_DP2.raw	
R.AVFPSIVGRPR.H	Y	44.25	1197.6981	11	1.0	599.8569	2	40.62	2	F2:11273	12112021_RID_2349_DP2.raw	
K.DLYANTVLSGGTTM(+15.99)YPGIADR.M	Y	41.30	2230.0576	21	-0.2	1116.0359	2	56.05	2	F2:16292	12112021_RID_2349_DP2.raw	

JAA97901.1
back to list

| Protein Coverage | Supporting Peptides |
Protein Coverage:

```
  1 MDDDIAALVV DNGSGMCKAG FAGDDAPRAV FPSIVGRPRH QGVMVGMGQK DSYVGDEAQS KRGILTLKYP IEHGIVTNWD
 81 DMEKIWHHTF YNELRVAPEE HPVLLTEAPL NPKANREKMT QIMFETFNTP AMYVAIQAVL SLYASGRTTG IVMDSGDGVT
161 HTVPIYEGYA LPHAILRLDL AGRDLTDYLM KILTERGYSF TTTAEREIVR DIKEKLCYVA LDFEQEMATA ASSSSLEKSY
241 ELPDGQVITI GNERFRCPEA LFQPSFLGME SCGIHETTFN SIMKCDVDIR KDLYANTVLS GGTTMYPGIA DRMQKEITAL
321 APSTMKIKII APPERKYSVW IGGSILASLS TFQQMWISKQ EYDESGPSIV HRKCF
```

Supporting Peptides:

Peptide	Uniq	-10lgP	Mass	Length	ppm	m/z	z	RT	Fraction	Scan	Source File	Area
R.GYSFTTTAER.E	Y	72.34	1131.5197	10	1.1	566.7677	2	33.52	2	F2:8772	12112021_RID_2349_DP2.raw	
K.SYELPDGQVITIGNER.F	Y	71.69	1789.8846	16	0.3	895.9499	2	53.88	2	F2:15567	12112021_RID_2349_DP2.raw	
K.DSYVGDEAQSK.R	Y	67.44	1197.5149	11	1.1	599.7654	2	23.86	2	F2:5370	12112021_RID_2349_DP2.raw	2.1509
R.VAPEEHPVLLTEAPLNPK.A	Y	63.91	1953.0570	18	0.6	977.5364	2	45.29	2	F2:12822	12112021_RID_2349_DP2.raw	
K.AGFAGDDAPR.A	Y	60.62	975.4410	10	0.5	488.7280	2	26.11	1	F1:7167	12112021_RID_2349_DP1.raw	3.3593
K.EITALAPSTMK.I	Y	52.44	1160.6111	11	0.0	581.3128	2	38.85	2	F2:10632	12112021_RID_2349_DP2.raw	

Peptide	Uniq	-10lgP	Mass	Length	ppm	m/z	z	RT	Fraction	Scan	Source File	Area
K.DSYVGDEAQSKR.G	Y	50.46	1353.6160	12	0.4	677.8156	2	20.64	2	F2:4244	12112021_RID_2349_DP2.raw	
K.EITALAPSTM(+15.99)K.I	Y	47.42	1176.6060	11	0.9	589.3108	2	32.91	2	F2:8537	12112021_RID_2349_DP2.raw	
R.AVFPSIVGRPR.H	Y	44.25	1197.6981	11	1.0	599.8569	2	40.62	2	F2:11273	12112021_RID_2349_DP2.raw	
K.DLYANTVLSGGTTM(+15.99)YPGIADR.M	Y	41.30	2230.0576	21	-0.2	1116.0359	2	56.05	2	F2:16292	12112021_RID_2349_DP2.raw	

JAG68844.1
back to list

| Protein Coverage | Supporting Peptides |
Protein Coverage:

1 MDDDIAALVV DNGSGMCK**AG FAGDDAPRAV FPSIVGRPR**H QGVMVGMGQK **DSYVGDEAQS KR**GILTLKYP IEHGIVTNWD

81 DMEKIWHHTF YNELR**VAPEE HPVLLTEAPL NPK**ANREKMT QIMFETFNTP AMYVAIQAVL SLYASGRTTG IVMDSGDGVT

161 HTVPIYEGYA LPHAILRLDL AGRDLTDYLM KILTER**GYSF TTTAER**EIVR DIKEKLCYVA LDFEQEMATA ASSSSLEK**SY**

241 **ELPDGQVITI GNER**FRCPEA LFQPSFLGME SCGIHETTFN SIMKCDVDIR K**DLYANTVLS GGTTMYPGIA DR**MQK**EITAL**

321 **APSTMK**IKII APPERKYSVW IGGSILASLS TFQQMWISKQ EYDESGPSIV HRKCF

Supporting Peptides:

Peptide	Uniq	-10lgP	Mass	Length	ppm	m/z	z	RT	Fraction	Scan	Source File	Area
R.GYSFTTTAER.E	Y	72.34	1131.5197	10	1.1	566.7677	2	33.52	2	F2:8772	12112021_RID_2349_DP2.raw	
K.SYELPDGQVITIGNER.F	Y	71.69	1789.8846	16	0.3	895.9499	2	53.88	2	F2:15567	12112021_RID_2349_DP2.raw	
K.DSYVGDEAQSK.R	Y	67.44	1197.5149	11	1.1	599.7654	2	23.86	2	F2:5370	12112021_RID_2349_DP2.raw	2.1509
R.VAPEEHPVLLTEAPLNPK.A	Y	63.91	1953.0570	18	0.6	977.5364	2	45.29	2	F2:12822	12112021_RID_2349_DP2.raw	
K.AGFAGDDAPR.A	Y	60.62	975.4410	10	0.5	488.7280	2	26.11	1	F1:7167	12112021_RID_2349_DP1.raw	3.3593
K.EITALAPSTMK.I	Y	52.44	1160.6111	11	0.0	581.3128	2	38.85	2	F2:10632	12112021_RID_2349_DP2.raw	
K.DSYVGDEAQSKR.G	Y	50.46	1353.6160	12	0.4	677.8156	2	20.64	2	F2:4244	12112021_RID_2349_DP2.raw	
K.EITALAPSTM(+15.99)K.I	Y	47.42	1176.6060	11	0.9	589.3108	2	32.91	2	F2:8537	12112021_RID_2349_DP2.raw	
R.AVFPSIVGRPR.H	Y	44.25	1197.6981	11	1.0	599.8569	2	40.62	2	F2:11273	12112021_RID_2349_DP2.raw	
K.DLYANTVLSGGTTM(+15.99)YPGIADR.M	Y	41.30	2230.0576	21	-0.2	1116.0359	2	56.05	2	F2:16292	12112021_RID_2349_DP2.raw	

LAA21098.1
back to list

| Protein Coverage | Supporting Peptides |
Protein Coverage:

1 MEEEIAALVI DNGSGMCK**AG FAGDDAPRAV FPSIVGRPR**H QGVMVGMGQK **DSYVGDEAQS KR**GILTLKYP IEHGIVTNWD

81 DMEKIWHHTF YNELR**VAPEE HPVLLTEAPL NPK**ANREKMT QIMFETFNTP AMYVAIQAVL SLYASGRTTG IVMDSGDGVT

161 HTVPIYEGYA LPHAILRLDL AGRDLTDYLM KILTER**GYSF TTTAER**EIVR DIKEKLCYVA LDFEQEMATA ASSSSLEK**SY**

241 **ELPDGQVITI GNER**FRCPEA LFQPSFLGME SCGIHETTFN SIMKCDVDIR K**DLYANTVLS GGTTMYPGIA DR**MQK**EITAL**

321 **APSTMK**IKII APPERKYSVW IGGSILASLS TFQQMWISKQ EYDESGPSIV HRKCF

Supporting Peptides:

Peptide	Uniq	-10lgP	Mass	Length	ppm	m/z	z	RT	Fraction	Scan	Source File	Area
R.GYSFTTTAER.E	Y	72.34	1131.5197	10	1.1	566.7677	2	33.52	2	F2:8772	12112021_RID_2349_DP2.raw	
K.SYELPDGQVITIGNER.F	Y	71.69	1789.8846	16	0.3	895.9499	2	53.88	2	F2:15567	12112021_RID_2349_DP2.raw	
K.DSYVGDEAQSK.R	Y	67.44	1197.5149	11	1.1	599.7654	2	23.86	2	F2:5370	12112021_RID_2349_DP2.raw	2.1509

Peptide	Uniq	-10lgP	Mass	Length	ppm	m/z	z	RT	Fraction	Scan	Source File	Area
R.VAPEEHPVLLTEAPLNPK.A	Y	63.91	1953.0570	18	0.6	977.5364	2	45.29	2	F2:12822	12112021_RID_2349_DP2.raw	
K.AGFAGDDAPR.A	Y	60.62	975.4410	10	0.5	488.7280	2	26.11	1	F1:7167	12112021_RID_2349_DP1.raw	3.3593
K.EITALAPSTMK.I	Y	52.44	1160.6111	11	0.0	581.3128	2	38.85	2	F2:10632	12112021_RID_2349_DP2.raw	
K.DSYVGDEAQSKR.G	Y	50.46	1353.6160	12	0.4	677.8156	2	20.64	2	F2:4244	12112021_RID_2349_DP2.raw	
K.EITALAPSTM(+15.99)K.I	Y	47.42	1176.6060	11	0.9	589.3108	2	32.91	2	F2:8537	12112021_RID_2349_DP2.raw	
R.AVFPSIVGRPR.H	Y	44.25	1197.6981	11	1.0	599.8569	2	40.62	2	F2:11273	12112021_RID_2349_DP2.raw	
K.DLYANTVLSGGTTM(+15.99)YPGIADR.M	Y	41.30	2230.0576	21	-0.2	1116.0359	2	56.05	2	F2:16292	12112021_RID_2349_DP2.raw	

JAV51374.1
back to list

| Protein Coverage | Supporting Peptides |
Protein Coverage:

1 MDDDIAALVV DNGSGMCK**AG FAGDDAPRAV FPSIVGRPR**H QGVMVGMGQK **DSYVGDEAQS KR**GILTLKYP IEHGIVTNWD

81 DMEKIWHHTF YNELR**VAPEE HPVLLTEAPL NPK**ANREKMT QIMFETFNTP AMYVAIQAVL SLYASGRTTG IVMDSGDGVT

161 HTVPIYEGYA LPHAILRLDL AGRDLTDYLM KILTER**GYSF TTTAER**EIVR DIKEKLCYVA LDFEQEMATA ASSSSLEK**SY**

241 **ELPDGQVITI GNER**FRCPEA LFQPSFLGME SCGIHETTFN SIMKCDVDIR K**DLYANTVLS GGTTMYPGIA DR**MQK**EITAL**

321 **APSTMK**IKII APPERKYSVW IGGSILASLS TFQQMWISKQ EYDESGPSIV HRKCF

Supporting Peptides:

Peptide	Uniq	-10lgP	Mass	Length	ppm	m/z	z	RT	Fraction	Scan	Source File	Area
R.GYSFTTTAER.E	Y	72.34	1131.5197	10	1.1	566.7677	2	33.52	2	F2:8772	12112021_RID_2349_DP2.raw	
K.SYELPDGQVITIGNER.F	Y	71.69	1789.8846	16	0.3	895.9499	2	53.88	2	F2:15567	12112021_RID_2349_DP2.raw	
K.DSYVGDEAQSK.R	Y	67.44	1197.5149	11	1.1	599.7654	2	23.86	2	F2:5370	12112021_RID_2349_DP2.raw	2.1509
R.VAPEEHPVLLTEAPLNPK.A	Y	63.91	1953.0570	18	0.6	977.5364	2	45.29	2	F2:12822	12112021_RID_2349_DP2.raw	
K.AGFAGDDAPR.A	Y	60.62	975.4410	10	0.5	488.7280	2	26.11	1	F1:7167	12112021_RID_2349_DP1.raw	3.3593
K.EITALAPSTMK.I	Y	52.44	1160.6111	11	0.0	581.3128	2	38.85	2	F2:10632	12112021_RID_2349_DP2.raw	
K.DSYVGDEAQSKR.G	Y	50.46	1353.6160	12	0.4	677.8156	2	20.64	2	F2:4244	12112021_RID_2349_DP2.raw	
K.EITALAPSTM(+15.99)K.I	Y	47.42	1176.6060	11	0.9	589.3108	2	32.91	2	F2:8537	12112021_RID_2349_DP2.raw	
R.AVFPSIVGRPR.H	Y	44.25	1197.6981	11	1.0	599.8569	2	40.62	2	F2:11273	12112021_RID_2349_DP2.raw	
K.DLYANTVLSGGTTM(+15.99)YPGIADR.M	Y	41.30	2230.0576	21	-0.2	1116.0359	2	56.05	2	F2:16292	12112021_RID_2349_DP2.raw	

JAG47580.1
back to list

| Protein Coverage | Supporting Peptides |
Protein Coverage:

1 MDDDIAALVV DNGSGMCK**AG FAGDDAPRAV FPSIVGRPR**H QGVMVGMGQK **DSYVGDEAQS KR**GILTLKYP IEHGIVTNWD

81 DMEKIWHHTF YNELR**VAPEE HPVLLTEAPL NPK**ANREKMT QIMFETFNTP AMYVAIQAVL SLYASGRTTG IVMDSGDGVT

161 HTVPIYEGYA LPHAILRLDL AGRDLTDYLM KILTER**GYSF TTTAER**EIVR DIKEKLCYVA LDFEQEMATA ASSSSLEK**SY**

241 **ELPDGQVITI GNER**FRCPEA LFQPSFLGME SCGIHETTFN SIMKCDVDIR K**DLYANTVLS GGTTMYPGIA DR**MQK**EITAL**

321 **APSTMK**IKII APPERKYSVW IGGSILASLS TFQQMWISKQ EYDESGPSIV HRKCF

Supporting Peptides:

Peptide	Uniq	-10lgP	Mass	Length	ppm	m/z	z	RT	Fraction	Scan	Source File	Area

Peptide	Uniq	-10lgP	Mass	Length	ppm	m/z	z	RT	Fraction	Scan	Source File	Area
R.GYSFTTTAER.E	Y	72.34	1131.5197	10	1.1	566.7677	2	33.52	2	F2:8772	12112021_RID_2349_DP2.raw	
K.SYELPDGQVITIGNER.F	Y	71.69	1789.8846	16	0.3	895.9499	2	53.88	2	F2:15567	12112021_RID_2349_DP2.raw	
K.DSYVGDEAQSK.R	Y	67.44	1197.5149	11	1.1	599.7654	2	23.86	2	F2:5370	12112021_RID_2349_DP2.raw	2.1509
R.VAPEEHPVLLTEAPLNPK.A	Y	63.91	1953.0570	18	0.6	977.5364	2	45.29	2	F2:12822	12112021_RID_2349_DP2.raw	
K.AGFAGDDAPR.A	Y	60.62	975.4410	10	0.5	488.7280	2	26.11	1	F1:7167	12112021_RID_2349_DP1.raw	3.3593
K.EITALAPSTMK.I	Y	52.44	1160.6111	11	0.0	581.3128	2	38.85	2	F2:10632	12112021_RID_2349_DP2.raw	
K.DSYVGDEAQSKR.G	Y	50.46	1353.6160	12	0.4	677.8156	2	20.64	2	F2:4244	12112021_RID_2349_DP2.raw	
K.EITALAPSTM(+15.99)K.I	Y	47.42	1176.6060	11	0.9	589.3108	2	32.91	2	F2:8537	12112021_RID_2349_DP2.raw	
R.AVFPSIVGRPR.H	Y	44.25	1197.6981	11	1.0	599.8569	2	40.62	2	F2:11273	12112021_RID_2349_DP2.raw	
K.DLYANTVLSGGTTM(+15.99)YPGIADR.M	Y	41.30	2230.0576	21	-0.2	1116.0359	2	56.05	2	F2:16292	12112021_RID_2349_DP2.raw	

AFJ49303.1
back to list

| Protein Coverage | Supporting Peptides |
Protein Coverage:

```
  1 MEEEIAALVI DNGSGMCKAG FAGDDAPRAV FPSIVGRPRH QGVMVGMGQK DSYVGDEAQS KRGILTLKYP IEHGIVTNWD C
 81 DMEKIWHHTF YNELRVAPEE HPVLLTEAPL NPKANREKMT QIMFETFNTP AMYVAIQAVL SLYASGRTTG IVMDSGDGVT
161 HTVPIYEGYA LPHAILRLDL AGRDLTDYLM KILTERGYSF TTTAEREIVR DIKEKLCYVA LDFEQEMATA ASSSSLEKSY
241 ELPDGQVITI GNERFRCPEA LFQPSFLGME SCGIHETTFN SIMKCDVDIR KDLYANTVLS GGTTMYPGIA DRMQKEITAL
321 APSTMKIKII APPERKYSVW IGGSILASLS TFQQMWISKQ EYDESGPSIV HRKCF
```

Supporting Peptides:

Peptide	Uniq	-10lgP	Mass	Length	ppm	m/z	z	RT	Fraction	Scan	Source File	Area
R.GYSFTTTAER.E	Y	72.34	1131.5197	10	1.1	566.7677	2	33.52	2	F2:8772	12112021_RID_2349_DP2.raw	
K.SYELPDGQVITIGNER.F	Y	71.69	1789.8846	16	0.3	895.9499	2	53.88	2	F2:15567	12112021_RID_2349_DP2.raw	
K.DSYVGDEAQSK.R	Y	67.44	1197.5149	11	1.1	599.7654	2	23.86	2	F2:5370	12112021_RID_2349_DP2.raw	2.1509
R.VAPEEHPVLLTEAPLNPK.A	Y	63.91	1953.0570	18	0.6	977.5364	2	45.29	2	F2:12822	12112021_RID_2349_DP2.raw	
K.AGFAGDDAPR.A	Y	60.62	975.4410	10	0.5	488.7280	2	26.11	1	F1:7167	12112021_RID_2349_DP1.raw	3.3593
K.EITALAPSTMK.I	Y	52.44	1160.6111	11	0.0	581.3128	2	38.85	2	F2:10632	12112021_RID_2349_DP2.raw	
K.DSYVGDEAQSKR.G	Y	50.46	1353.6160	12	0.4	677.8156	2	20.64	2	F2:4244	12112021_RID_2349_DP2.raw	
K.EITALAPSTM(+15.99)K.I	Y	47.42	1176.6060	11	0.9	589.3108	2	32.91	2	F2:8537	12112021_RID_2349_DP2.raw	
R.AVFPSIVGRPR.H	Y	44.25	1197.6981	11	1.0	599.8569	2	40.62	2	F2:11273	12112021_RID_2349_DP2.raw	
K.DLYANTVLSGGTTM(+15.99)YPGIADR.M	Y	41.30	2230.0576	21	-0.2	1116.0359	2	56.05	2	F2:16292	12112021_RID_2349_DP2.raw	

JAI14496.1
back to list

| Protein Coverage | Supporting Peptides |
Protein Coverage:

1 MDDDIAALVV DNGSGMCKAG FAGDDAPRAV FPSIVGRPRH QGVMVGMGQK DSYVGDEAQS KRGILTLKYP IEHGIVTNWD

81 DMEKIWHHTF YNELRVAPEE HPVLLTEAPL NPKANREKMT QIMFETFNTP AMYVAIQAVL SLYASGRTTG IVMDSGDGVT

161 HTVPIYEGYA LPHAILRLDL AGRDLTDYLM KILTERGYSF TTTAEREIVR DIKEKLCYVA LDFEQEMATA ASSSSLEKSY

241 ELPDGQVITI GNERFRCPEA LFQPSFLGME SCGIHETTFN SIMKCDVDIR KDLYANTVLS GGTTMYPGIA DRMQKEITAL

321 APSTMKIKII APPERKYSVW IGGSILASLS TFQQMWISKQ EYDESGPSIV HRKCF

Supporting Peptides:

Peptide	Uniq	-10lgP	Mass	Length	ppm	m/z	z	RT	Fraction	Scan	Source File	Area
R.GYSFTTTAER.E	Y	72.34	1131.5197	10	1.1	566.7677	2	33.52	2	F2:8772	12112021_RID_2349_DP2.raw	
K.SYELPDGQVITIGNER.F	Y	71.69	1789.8846	16	0.3	895.9499	2	53.88	2	F2:15567	12112021_RID_2349_DP2.raw	
K.DSYVGDEAQSK.R	Y	67.44	1197.5149	11	1.1	599.7654	2	23.86	2	F2:5370	12112021_RID_2349_DP2.raw	2.1509
R.VAPEEHPVLLTEAPLNPK.A	Y	63.91	1953.0570	18	0.6	977.5364	2	45.29	2	F2:12822	12112021_RID_2349_DP2.raw	
K.AGFAGDDAPR.A	Y	60.62	975.4410	10	0.5	488.7280	2	26.11	1	F1:7167	12112021_RID_2349_DP1.raw	3.3593
K.EITALAPSTMK.I	Y	52.44	1160.6111	11	0.0	581.3128	2	38.85	2	F2:10632	12112021_RID_2349_DP2.raw	
K.DSYVGDEAQSKR.G	Y	50.46	1353.6160	12	0.4	677.8156	2	20.64	2	F2:4244	12112021_RID_2349_DP2.raw	
K.EITALAPSTM(+15.99)K.I	Y	47.42	1176.6060	11	0.9	589.3108	2	32.91	2	F2:8537	12112021_RID_2349_DP2.raw	
R.AVFPSIVGRPR.H	Y	44.25	1197.6981	11	1.0	599.8569	2	40.62	2	F2:11273	12112021_RID_2349_DP2.raw	
K.DLYANTVLSGGTTM(+15.99)YPGIADR.M	Y	41.30	2230.0576	21	-0.2	1116.0359	2	56.05	2	F2:16292	12112021_RID_2349_DP2.raw	

JAI10341.1
back to list

| Protein Coverage | Supporting Peptides |
Protein Coverage:

1 MEEEIAALVI DNGSGMCKAG FAGDDAPRAV FPSIVGRPRH QGVMVGMGQK DSYVGDEAQS KRGILTLKYP IEHGIVTNWD

81 DMEKIWHHTF YNELRVAPEE HPVLLTEAPL NPKANREKMT QIMFETFNTP AMYVAIQAVL SLYASGRTTG IVMDSGDGVT

161 HTVPIYEGYA LPHAILRLDL AGRDLTDYLM KILTERGYSF TTTAEREIVR DIKEKLCYVA LDFEQEMATA ASSSSLEKSY

241 ELPDGQVITI GNERFRCPEA LFQPSFLGME SCGIHETTFN SIMKCDVDIR KDLYANTVLS GGTTMYPGIA DRMQKEITAL

321 APSTMKIKII APPERKYSVW IGGSILASLS TFQQMWISKQ EYDESGPSIV HRKCF

Supporting Peptides:

Peptide	Uniq	-10lgP	Mass	Length	ppm	m/z	z	RT	Fraction	Scan	Source File	Area
R.GYSFTTTAER.E	Y	72.34	1131.5197	10	1.1	566.7677	2	33.52	2	F2:8772	12112021_RID_2349_DP2.raw	
K.SYELPDGQVITIGNER.F	Y	71.69	1789.8846	16	0.3	895.9499	2	53.88	2	F2:15567	12112021_RID_2349_DP2.raw	
K.DSYVGDEAQSK.R	Y	67.44	1197.5149	11	1.1	599.7654	2	23.86	2	F2:5370	12112021_RID_2349_DP2.raw	2.1509
R.VAPEEHPVLLTEAPLNPK.A	Y	63.91	1953.0570	18	0.6	977.5364	2	45.29	2	F2:12822	12112021_RID_2349_DP2.raw	
K.AGFAGDDAPR.A	Y	60.62	975.4410	10	0.5	488.7280	2	26.11	1	F1:7167	12112021_RID_2349_DP1.raw	3.3593
K.EITALAPSTMK.I	Y	52.44	1160.6111	11	0.0	581.3128	2	38.85	2	F2:10632	12112021_RID_2349_DP2.raw	
K.DSYVGDEAQSKR.G	Y	50.46	1353.6160	12	0.4	677.8156	2	20.64	2	F2:4244	12112021_RID_2349_DP2.raw	
K.EITALAPSTM(+15.99)K.I	Y	47.42	1176.6060	11	0.9	589.3108	2	32.91	2	F2:8537	12112021_RID_2349_DP2.raw	
R.AVFPSIVGRPR.H	Y	44.25	1197.6981	11	1.0	599.8569	2	40.62	2	F2:11273	12112021_RID_2349_DP2.raw	
K.DLYANTVLSGGTTM(+15.99)YPGIADR.M	Y	41.30	2230.0576	21	-0.2	1116.0359	2	56.05	2	F2:16292	12112021_RID_2349_DP2.raw	

JAI14495.1
back to list

| Protein Coverage | Supporting Peptides |

Protein Coverage:

```
1   MEEEIAALVI DNGSGMCKAG FAGDDAPRAV FPSIVGRPRH QGVMVGMGQK DSYVGDEAQS KRGILTLKYP IEHGIVTNWD
81  DMEKIWHHTF YNELRVAPEE HPVLLTEAPL NPKANREKMT QIMFETFNTP AMYVAIQAVL SLYASGRTTG IVMDSGDGVT
161 HTVPIYEGYA LPHAILRLDL AGRDLTDYLM KILTERGYSF TTTAEREIVR DIKEKLCYVA LDFEQEMATA ASSSSLEKSY
241 ELPDGQVITI GNERFRCPEA LFQPSFLGME SCGIHETTFN SIMKCDVDIR KDLYANTVLS GGTTMYPGIA DRMQKEITAL
321 APSTMKIKII APPERKYSVW IGGSILASLS TFQQMWISKQ EYDESGPSIV HRKCF
```

Supporting Peptides:

Peptide	Uniq	-10lgP	Mass	Length	ppm	m/z	z	RT	Fraction	Scan	Source File	Area
R.GYSFTTTAER.E	Y	72.34	1131.5197	10	1.1	566.7677	2	33.52	2	F2:8772	12112021_RID_2349_DP2.raw	
K.SYELPDGQVITIGNER.F	Y	71.69	1789.8846	16	0.3	895.9499	2	53.88	2	F2:15567	12112021_RID_2349_DP2.raw	
K.DSYVGDEAQSK.R	Y	67.44	1197.5149	11	1.1	599.7654	2	23.86	2	F2:5370	12112021_RID_2349_DP2.raw	2.1509
R.VAPEEHPVLLTEAPLNPK.A	Y	63.91	1953.0570	18	0.6	977.5364	2	45.29	2	F2:12822	12112021_RID_2349_DP2.raw	
K.AGFAGDDAPR.A	Y	60.62	975.4410	10	0.5	488.7280	2	26.11	1	F1:7167	12112021_RID_2349_DP1.raw	3.3593
K.EITALAPSTMK.I	Y	52.44	1160.6111	11	0.0	581.3128	2	38.85	2	F2:10632	12112021_RID_2349_DP2.raw	
K.DSYVGDEAQSKR.G	Y	50.46	1353.6160	12	0.4	677.8156	2	20.64	2	F2:4244	12112021_RID_2349_DP2.raw	
K.EITALAPSTM(+15.99)K.I	Y	47.42	1176.6060	11	0.9	589.3108	2	32.91	2	F2:8537	12112021_RID_2349_DP2.raw	
R.AVFPSIVGRPR.H	Y	44.25	1197.6981	11	1.0	599.8569	2	40.62	2	F2:11273	12112021_RID_2349_DP2.raw	
K.DLYANTVLSGGTTM(+15.99)YPGIADR.M	Y	41.30	2230.0576	21	-0.2	1116.0359	2	56.05	2	F2:16292	12112021_RID_2349_DP2.raw	

JAC96364.1

back to list

| Protein Coverage | Supporting Peptides |

Protein Coverage:

```
1   MDDDIAALVV DNGSGMCKAG FAGDDAPRAV FPSIVGRPRH QGVMVGMGQK DSYVGDEAQS KRGILTLKYP IEHGIVTNWD
81  DMEKIWHHTF YNELRVAPEE HPVLLTEAPL NPKANREKMT QIMFETFNTP AMYVAIQAVL SLYASGRTTG IVMDSGDGVT
161 HTVPIYEGYA LPHAILRLDL AGRDLTDYLM KILTERGYSF TTTAEREIVR DIKEKLCYVA LDFEQEMATA ASSSSLEKSY
241 ELPDGQVITI GNERFRCPEA LFQPSFLGME SCGIHETTFN SIMKCDVDIR KDLYANTVLS GGTTMYPGIA DRMQKEITAL
321 APSTMKIKII APPERKYSVW IGGSILASLS TFQQMWISKQ EYDESGPSIV HRKCF
```

Supporting Peptides:

Peptide	Uniq	-10lgP	Mass	Length	ppm	m/z	z	RT	Fraction	Scan	Source File	Area
R.GYSFTTTAER.E	Y	72.34	1131.5197	10	1.1	566.7677	2	33.52	2	F2:8772	12112021_RID_2349_DP2.raw	
K.SYELPDGQVITIGNER.F	Y	71.69	1789.8846	16	0.3	895.9499	2	53.88	2	F2:15567	12112021_RID_2349_DP2.raw	
K.DSYVGDEAQSK.R	Y	67.44	1197.5149	11	1.1	599.7654	2	23.86	2	F2:5370	12112021_RID_2349_DP2.raw	2.1509
R.VAPEEHPVLLTEAPLNPK.A	Y	63.91	1953.0570	18	0.6	977.5364	2	45.29	2	F2:12822	12112021_RID_2349_DP2.raw	
K.AGFAGDDAPR.A	Y	60.62	975.4410	10	0.5	488.7280	2	26.11	1	F1:7167	12112021_RID_2349_DP1.raw	3.3593
K.EITALAPSTMK.I	Y	52.44	1160.6111	11	0.0	581.3128	2	38.85	2	F2:10632	12112021_RID_2349_DP2.raw	
K.DSYVGDEAQSKR.G	Y	50.46	1353.6160	12	0.4	677.8156	2	20.64	2	F2:4244	12112021_RID_2349_DP2.raw	
K.EITALAPSTM(+15.99)K.I	Y	47.42	1176.6060	11	0.9	589.3108	2	32.91	2	F2:8537	12112021_RID_2349_DP2.raw	
R.AVFPSIVGRPR.H	Y	44.25	1197.6981	11	1.0	599.8569	2	40.62	2	F2:11273	12112021_RID_2349_DP2.raw	

Peptide	Uniq	-10lgP	Mass	Length	ppm	m/z	z	RT	Fraction	Scan	Source File	Area
K.DLYANTVLSGGTTM(+15.99)YPGIADR.M	Y	41.30	2230.0576	21	-0.2	1116.0359	2	56.05	2	F2:16292	12112021_RID_2349_DP2.raw	

JAG47579.1
back to list

| Protein Coverage | Supporting Peptides |
Protein Coverage:

1 MEEEIAALVI DNGSGMCK**AG FAGDDAPRAV FPSIVGRPR**H QGVMVGMGQK **DSYVGDEAQS KR**GILTLKYP IEHGIVTNWD

81 DMEKIWHHTF YNELR**VAPEE HPVLLTEAPL NPK**ANREKMT QIMFETFNTP AMYVAIQAVL SLYASGRTTG IVMDSGDGVT

161 HTVPIYEGYA LPHAILRLDL AGRDLTDYLM KILTER**GYSF TTTAER**EIVR DIKEKLCYVA LDFEQEMATA ASSSSLEK**SY**

241 **ELPDGQVITI GNER**FRCPEA LFQPSFLGME SCGIHETTFN SIMKCDVDIR K**DLYANTVLS GGTTMYPGIA DR**MQK**EITAL**

321 **APSTMK**IKII APPERKYSVW IGGSILASLS TFQQMWISKQ EYDESGPSIV HRKCF

Supporting Peptides:

Peptide	Uniq	-10lgP	Mass	Length	ppm	m/z	z	RT	Fraction	Scan	Source File	Area
R.GYSFTTTAER.E	Y	72.34	1131.5197	10	1.1	566.7677	2	33.52	2	F2:8772	12112021_RID_2349_DP2.raw	
K.SYELPDGQVITIGNER.F	Y	71.69	1789.8846	16	0.3	895.9499	2	53.88	2	F2:15567	12112021_RID_2349_DP2.raw	
K.DSYVGDEAQSK.R	Y	67.44	1197.5149	11	1.1	599.7654	2	23.86	2	F2:5370	12112021_RID_2349_DP2.raw	2.1509
R.VAPEEHPVLLTEAPLNPK.A	Y	63.91	1953.0570	18	0.6	977.5364	2	45.29	2	F2:12822	12112021_RID_2349_DP2.raw	
K.AGFAGDDAPR.A	Y	60.62	975.4410	10	0.5	488.7280	2	26.11	1	F1:7167	12112021_RID_2349_DP1.raw	3.3593
K.EITALAPSTMK.I	Y	52.44	1160.6111	11	0.0	581.3128	2	38.85	2	F2:10632	12112021_RID_2349_DP2.raw	
K.DSYVGDEAQSKR.G	Y	50.46	1353.6160	12	0.4	677.8156	2	20.64	2	F2:4244	12112021_RID_2349_DP2.raw	
K.EITALAPSTM(+15.99)K.I	Y	47.42	1176.6060	11	0.9	589.3108	2	32.91	2	F2:8537	12112021_RID_2349_DP2.raw	
R.AVFPSIVGRPR.H	Y	44.25	1197.6981	11	1.0	599.8569	2	40.62	2	F2:11273	12112021_RID_2349_DP2.raw	
K.DLYANTVLSGGTTM(+15.99)YPGIADR.M	Y	41.30	2230.0576	21	-0.2	1116.0359	2	56.05	2	F2:16292	12112021_RID_2349_DP2.raw	

JAV51372.1
back to list

| Protein Coverage | Supporting Peptides |
Protein Coverage:

1 MEEEIAALVI DNGSGMCK**AG FAGDDAPRAV FPSIVGRPR**H QGVMVGMGQK **DSYVGDEAQS KR**GILTLKYP IEHGIVTNWD

81 DMEKIWHHTF YNELR**VAPEE HPVLLTEAPL NPK**ANREKMT QIMFETFNTP AMYVAIQAVL SLYASGRTTG IVMDSGDGVT

161 HTVPIYEGYA LPHAILRLDL AGRDLTDYLM KILTER**GYSF TTTAER**EIVR DIKEKLCYVA LDFEQEMATA ASSSSLEK**SY**

241 **ELPDGQVITI GNER**FRCPEA LFQPSFLGME SCGIHETTFN SIMKCDVDIR K**DLYANTVLS GGTTMYPGIA DR**MQK**EITAL**

321 **APSTMK**IKII APPERKYSVW IGGSILASLS TFQQMWISKQ EYDESGPSIV HRKCF

Supporting Peptides:

Peptide	Uniq	-10lgP	Mass	Length	ppm	m/z	z	RT	Fraction	Scan	Source File	Area
R.GYSFTTTAER.E	Y	72.34	1131.5197	10	1.1	566.7677	2	33.52	2	F2:8772	12112021_RID_2349_DP2.raw	
K.SYELPDGQVITIGNER.F	Y	71.69	1789.8846	16	0.3	895.9499	2	53.88	2	F2:15567	12112021_RID_2349_DP2.raw	
K.DSYVGDEAQSK.R	Y	67.44	1197.5149	11	1.1	599.7654	2	23.86	2	F2:5370	12112021_RID_2349_DP2.raw	2.1509
R.VAPEEHPVLLTEAPLNPK.A	Y	63.91	1953.0570	18	0.6	977.5364	2	45.29	2	F2:12822	12112021_RID_2349_DP2.raw	
K.AGFAGDDAPR.A	Y	60.62	975.4410	10	0.5	488.7280	2	26.11	1	F1:7167	12112021_RID_2349_DP1.raw	3.3593
K.EITALAPSTMK.I	Y	52.44	1160.6111	11	0.0	581.3128	2	38.85	2	F2:10632	12112021_RID_2349_DP2.raw	

Peptide	Uniq	-10lgP	Mass	Length	ppm	m/z	z	RT	Fraction	Scan	Source File	Area
K.DSYVGDEAQSKR.G	Y	50.46	1353.6160	12	0.4	677.8156	2	20.64	2	F2:4244	12112021_RID_2349_DP2.raw	
K.EITALAPSTM(+15.99)K.I	Y	47.42	1176.6060	11	0.9	589.3108	2	32.91	2	F2:8537	12112021_RID_2349_DP2.raw	
R.AVFPSIVGRPR.H	Y	44.25	1197.6981	11	1.0	599.8569	2	40.62	2	F2:11273	12112021_RID_2349_DP2.raw	
K.DLYANTVLSGGTTM(+15.99)YPGIADR.M	Y	41.30	2230.0576	21	-0.2	1116.0359	2	56.05	2	F2:16292	12112021_RID_2349_DP2.raw	

AFJ49302.1
back to list

| Protein Coverage | Supporting Peptides |
Protein Coverage:

1 MDDDIAALVV DNGSGMCK**AG FAGDDAPRAV FPSIVGRPR**H QGVMVGMGQK **DSYVGDEAQS KR**GILTLKYP IEHGIVTNWD

81 DMEKIWHHTF YNELR**VAPEE HPVLLTEAPL NPK**ANREKMT QIMFETFNTP AMYVAIQAVL SLYASGRTTG IVMDSGDGVT

161 HTVPIYEGYA LPHAILRLDL AGRDLTDYLM KILTER**GYSF TTTAER**EIVR DIKEKLCYVA LDFEQEMATA ASSSSLEK**SY**

241 **ELPDGQVITI GNER**FRCPEA LFQPSFLGME SCGIHETTFN SIMKCDVDIR K**DLYANTVLS GGTTMYPGIA DR**MQK**EITAL**

321 **APSTMK**IKII APPERKYSVW IGGSILASLS TFQQMWISKQ EYDESGPSIV HRKCF

Supporting Peptides:

Peptide	Uniq	-10lgP	Mass	Length	ppm	m/z	z	RT	Fraction	Scan	Source File	Area
R.GYSFTTTAER.E	Y	72.34	1131.5197	10	1.1	566.7677	2	33.52	2	F2:8772	12112021_RID_2349_DP2.raw	
K.SYELPDGQVITIGNER.F	Y	71.69	1789.8846	16	0.3	895.9499	2	53.88	2	F2:15567	12112021_RID_2349_DP2.raw	
K.DSYVGDEAQSK.R	Y	67.44	1197.5149	11	1.1	599.7654	2	23.86	2	F2:5370	12112021_RID_2349_DP2.raw	2.1509
R.VAPEEHPVLLTEAPLNPK.A	Y	63.91	1953.0570	18	0.6	977.5364	2	45.29	2	F2:12822	12112021_RID_2349_DP2.raw	
K.AGFAGDDAPR.A	Y	60.62	975.4410	10	0.5	488.7280	2	26.11	1	F1:7167	12112021_RID_2349_DP1.raw	3.3593
K.EITALAPSTMK.I	Y	52.44	1160.6111	11	0.0	581.3128	2	38.85	2	F2:10632	12112021_RID_2349_DP2.raw	
K.DSYVGDEAQSKR.G	Y	50.46	1353.6160	12	0.4	677.8156	2	20.64	2	F2:4244	12112021_RID_2349_DP2.raw	
K.EITALAPSTM(+15.99)K.I	Y	47.42	1176.6060	11	0.9	589.3108	2	32.91	2	F2:8537	12112021_RID_2349_DP2.raw	
R.AVFPSIVGRPR.H	Y	44.25	1197.6981	11	1.0	599.8569	2	40.62	2	F2:11273	12112021_RID_2349_DP2.raw	
K.DLYANTVLSGGTTM(+15.99)YPGIADR.M	Y	41.30	2230.0576	21	-0.2	1116.0359	2	56.05	2	F2:16292	12112021_RID_2349_DP2.raw	

BAQ22177.1
back to list

| Protein Coverage | Supporting Peptides |
Protein Coverage:

1 MDDDIAALVV DNGSGMCK**AG FAGDDAPRAV FPSIVGRPR**H QGVMVGMGQK **DSYVGDEAQS KR**GILTLKYP IEHGIVTNWD

81 DMEKIWHHTF YNELR**VAPEE HPVLLTEAPL NPK**ANREKMT QIMFETFNTP AMYVAIQAVL SLYASGRTTG IVMDSGDGVT

161 HTVPIYEGYA LPHAILRLDL AGRDLTDYLM KILTER**GYSF TTTAER**EIVR DIKEKLCYVA LDFEQEMATA ASSSSLEK**SY**

241 **ELPDGQVITI GNER**FRCPEA LFQPSFLGME SCGIHETTFN SIMKCDVDIR K**DLYANTVLS GGTTMYPGIA DR**MQK**EITAL**

321 **APSTMK**IKII APPERKYSVW IGGSILASLS TFQQMWISKQ EYDESGPSIV HRKCF

Supporting Peptides:

Peptide	Uniq	-10lgP	Mass	Length	ppm	m/z	z	RT	Fraction	Scan	Source File	Area
R.GYSFTTTAER.E	Y	72.34	1131.5197	10	1.1	566.7677	2	33.52	2	F2:8772	12112021_RID_2349_DP2.raw	
K.SYELPDGQVITIGNER.F	Y	71.69	1789.8846	16	0.3	895.9499	2	53.88	2	F2:15567	12112021_RID_2349_DP2.raw	
K.DSYVGDEAQSK.R	Y	67.44	1197.5149	11	1.1	599.7654	2	23.86	2	F2:5370	12112021_RID_2349_DP2.raw	2.1509

Peptide	Uniq	-10lgP	Mass	Length	ppm	m/z	z	RT	Fraction	Scan	Source File	Area
R.VAPEEHPVLLTEAPLNPK.A	Y	63.91	1953.0570	18	0.6	977.5364	2	45.29	2	F2:12822	12112021_RID_2349_DP2.raw	
K.AGFAGDDAPR.A	Y	60.62	975.4410	10	0.5	488.7280	2	26.11	1	F1:7167	12112021_RID_2349_DP1.raw	3.3593
K.EITALAPSTMK.I	Y	52.44	1160.6111	11	0.0	581.3128	2	38.85	2	F2:10632	12112021_RID_2349_DP2.raw	
K.DSYVGDEAQSKR.G	Y	50.46	1353.6160	12	0.4	677.8156	2	20.64	2	F2:4244	12112021_RID_2349_DP2.raw	
K.EITALAPSTM(+15.99)K.I	Y	47.42	1176.6060	11	0.9	589.3108	2	32.91	2	F2:8537	12112021_RID_2349_DP2.raw	
R.AVFPSIVGRPR.H	Y	44.25	1197.6981	11	1.0	599.8569	2	40.62	2	F2:11273	12112021_RID_2349_DP2.raw	
K.DLYANTVLSGGTTM(+15.99)YPGIADR.M	Y	41.30	2230.0576	21	-0.2	1116.0359	2	56.05	2	F2:16292	12112021_RID_2349_DP2.raw	

JAG45996.1
back to list

| Protein Coverage | Supporting Peptides |
Protein Coverage:

1 MEEEIAALVI DNGSGMCK**AG FAGDDAPRAV FPSIVGRPR**H QGVMVGMGQK **DSYVGDEAQS KR**GILTLKYP IEHGIVTNWD

81 DMEKIWHHTF YNELR**VAPEE HPVLLTEAPL NPK**ANREKMT QIMFETFNTP AMYVAIQAVL SLYASGRTTG IVMDSGDGVT

161 HTVPIYEGYA LPHAILRLDL AGRDLTDYLM KILTER**GYSF TTTAER**EIVR DIKEKLCYVA LDFEQEMATA ASSSSLEK**SY**

241 **ELPDGQVITI GNER**FRCPEA LFQPSFLGME SCGIHETTFN SIMKCDVDIR K**DLYANTVLS GGTTMYPGIA DR**MQK**EITAL**

321 **APSTMK**IKII APPERKYSVW IGGSILASLS TFQQMWISKQ EYDESGPSIV HRKCF

Supporting Peptides:

Peptide	Uniq	-10lgP	Mass	Length	ppm	m/z	z	RT	Fraction	Scan	Source File	Area
R.GYSFTTTAER.E	Y	72.34	1131.5197	10	1.1	566.7677	2	33.52	2	F2:8772	12112021_RID_2349_DP2.raw	
K.SYELPDGQVITIGNER.F	Y	71.69	1789.8846	16	0.3	895.9499	2	53.88	2	F2:15567	12112021_RID_2349_DP2.raw	
K.DSYVGDEAQSK.R	Y	67.44	1197.5149	11	1.1	599.7654	2	23.86	2	F2:5370	12112021_RID_2349_DP2.raw	2.1509
R.VAPEEHPVLLTEAPLNPK.A	Y	63.91	1953.0570	18	0.6	977.5364	2	45.29	2	F2:12822	12112021_RID_2349_DP2.raw	
K.AGFAGDDAPR.A	Y	60.62	975.4410	10	0.5	488.7280	2	26.11	1	F1:7167	12112021_RID_2349_DP1.raw	3.3593
K.EITALAPSTMK.I	Y	52.44	1160.6111	11	0.0	581.3128	2	38.85	2	F2:10632	12112021_RID_2349_DP2.raw	
K.DSYVGDEAQSKR.G	Y	50.46	1353.6160	12	0.4	677.8156	2	20.64	2	F2:4244	12112021_RID_2349_DP2.raw	
K.EITALAPSTM(+15.99)K.I	Y	47.42	1176.6060	11	0.9	589.3108	2	32.91	2	F2:8537	12112021_RID_2349_DP2.raw	
R.AVFPSIVGRPR.H	Y	44.25	1197.6981	11	1.0	599.8569	2	40.62	2	F2:11273	12112021_RID_2349_DP2.raw	
K.DLYANTVLSGGTTM(+15.99)YPGIADR.M	Y	41.30	2230.0576	21	-0.2	1116.0359	2	56.05	2	F2:16292	12112021_RID_2349_DP2.raw	

JAG45999.1
back to list

| Protein Coverage | Supporting Peptides |
Protein Coverage:

1 MDDDIAALVV DNGSGMCK**AG FAGDDAPRAV FPSIVGRPR**H QGVMVGMGQK **DSYVGDEAQS KR**GILTLKYP IEHGIVTNWD

81 DMEKIWHHTF YNELR**VAPEE HPVLLTEAPL NPK**ANREKMT QIMFETFNTP AMYVAIQAVL SLYASGRTTG IVMDSGDGVT

161 HTVPIYEGYA LPHAILRLDL AGRDLTDYLM KILTER**GYSF TTTAER**EIVR DIKEKLCYVA LDFEQEMATA ASSSSLEK**SY**

241 **ELPDGQVITI GNER**FRCPEA LFQPSFLGME SCGIHETTFN SIMKCDVDIR K**DLYANTVLS GGTTMYPGIA DR**MQK**EITAL**

321 **APSTMK**IKII APPERKYSVW IGGSILASLS TFQQMWISKQ EYDESGPSIV HRKCF

Supporting Peptides:

Peptide	Uniq	-10lgP	Mass	Length	ppm	m/z	z	RT	Fraction	Scan	Source File	Area

Peptide	Uniq	-10lgP	Mass	Length	ppm	m/z	z	RT	Fraction	Scan	Source File	Area
R.GYSFTTTAER.E	Y	72.34	1131.5197	10	1.1	566.7677	2	33.52	2	F2:8772	12112021_RID_2349_DP2.raw	
K.SYELPDGQVITIGNER.F	Y	71.69	1789.8846	16	0.3	895.9499	2	53.88	2	F2:15567	12112021_RID_2349_DP2.raw	
K.DSYVGDEAQSK.R	Y	67.44	1197.5149	11	1.1	599.7654	2	23.86	2	F2:5370	12112021_RID_2349_DP2.raw	2.1509
R.VAPEEHPVLLTEAPLNPK.A	Y	63.91	1953.0570	18	0.6	977.5364	2	45.29	2	F2:12822	12112021_RID_2349_DP2.raw	
K.AGFAGDDAPR.A	Y	60.62	975.4410	10	0.5	488.7280	2	26.11	1	F1:7167	12112021_RID_2349_DP1.raw	3.3593
K.EITALAPSTMK.I	Y	52.44	1160.6111	11	0.0	581.3128	2	38.85	2	F2:10632	12112021_RID_2349_DP2.raw	
K.DSYVGDEAQSKR.G	Y	50.46	1353.6160	12	0.4	677.8156	2	20.64	2	F2:4244	12112021_RID_2349_DP2.raw	
K.EITALAPSTM(+15.99)K.I	Y	47.42	1176.6060	11	0.9	589.3108	2	32.91	2	F2:8537	12112021_RID_2349_DP2.raw	
R.AVFPSIVGRPR.H	Y	44.25	1197.6981	11	1.0	599.8569	2	40.62	2	F2:11273	12112021_RID_2349_DP2.raw	
K.DLYANTVLSGGTTM(+15.99)YPGIADR.M	Y	41.30	2230.0576	21	-0.2	1116.0359	2	56.05	2	F2:16292	12112021_RID_2349_DP2.raw	

JAB54395.1
back to list

| Protein Coverage | Supporting Peptides |
Protein Coverage:

1 MEEEIAALVI DNGSGMCK**AG FAGDDAPRAV FPSIVGRPR**H QGVMVGMGQK **DSYVGDEAQS KR**GILTLKYP IEHGIVTNWD

81 DMEKIWHHTF YNELR**VAPEE HPVLLTEAPL NPK**ANREKMT QIMFETFNTP AMYVAIQAVL SLYASGRTTG IVMDSGDGVT

161 HTVPIYEGYA LPHAILRLDL AGRDLTDYLM KILTER**GYSF TTTAER**EIVR DIKEKLCYVA LDFEQEMATA ASSSSLEK**SY**

241 **ELPDGQVITI GNER**FRCPEA LFQPSFLGME SCGIHETTFN SIMKCDVDIR K**DLYANTVLS GGTTMYPGIA DR**MQK**EITAL**

321 **APSTMK**IKII APPERKYSVW IGGSILASLS TFQQMWISKQ EYDESGPSIV HRKCF

Supporting Peptides:

Peptide	Uniq	-10lgP	Mass	Length	ppm	m/z	z	RT	Fraction	Scan	Source File	Area
R.GYSFTTTAER.E	Y	72.34	1131.5197	10	1.1	566.7677	2	33.52	2	F2:8772	12112021_RID_2349_DP2.raw	
K.SYELPDGQVITIGNER.F	Y	71.69	1789.8846	16	0.3	895.9499	2	53.88	2	F2:15567	12112021_RID_2349_DP2.raw	
K.DSYVGDEAQSK.R	Y	67.44	1197.5149	11	1.1	599.7654	2	23.86	2	F2:5370	12112021_RID_2349_DP2.raw	2.1509
R.VAPEEHPVLLTEAPLNPK.A	Y	63.91	1953.0570	18	0.6	977.5364	2	45.29	2	F2:12822	12112021_RID_2349_DP2.raw	
K.AGFAGDDAPR.A	Y	60.62	975.4410	10	0.5	488.7280	2	26.11	1	F1:7167	12112021_RID_2349_DP1.raw	3.3593
K.EITALAPSTMK.I	Y	52.44	1160.6111	11	0.0	581.3128	2	38.85	2	F2:10632	12112021_RID_2349_DP2.raw	
K.DSYVGDEAQSKR.G	Y	50.46	1353.6160	12	0.4	677.8156	2	20.64	2	F2:4244	12112021_RID_2349_DP2.raw	
K.EITALAPSTM(+15.99)K.I	Y	47.42	1176.6060	11	0.9	589.3108	2	32.91	2	F2:8537	12112021_RID_2349_DP2.raw	
R.AVFPSIVGRPR.H	Y	44.25	1197.6981	11	1.0	599.8569	2	40.62	2	F2:11273	12112021_RID_2349_DP2.raw	
K.DLYANTVLSGGTTM(+15.99)YPGIADR.M	Y	41.30	2230.0576	21	-0.2	1116.0359	2	56.05	2	F2:16292	12112021_RID_2349_DP2.raw	

ETE74025.1
back to list

| Protein Coverage | Supporting Peptides |
Protein Coverage:

1 MEEEIAALVI DNGSGMCKAG FAGDDAPRAV FPSIVGRPRH QGVMVGMGQK DSYVGDEAQS KRGILTLKYP IEHGIVTNWD

81 DMEKIWHHTF YNELRVAPEE HPVLLTEAPL NPKANREKMT QIMFETFNTP AMYVAIQAVL SLYASGRTTG IVMDSGDGVT

161 HTVPIYEGYA LPHAILRLDL AGRDLTDYLM KILTERGYSF TTTAEREIVR DIKEKLCYVA LDFEQEMATA ASSSSLEKSY

241 ELPDGQVITI GNERFRCPEA LFQPSFLGME SCGIHETTFN SIMKCDVDIR KDLYANTVLS GGTTMYPGIA DRMQKEITAL

321 APSTMKIKII APPERKYSVW IGGSILASLS TFQQMWISKQ EYDESGPSIV HRKCF

Supporting Peptides:

Peptide	Uniq	-10lgP	Mass	Length	ppm	m/z	z	RT	Fraction	Scan	Source File	Area
R.GYSFTTTAER.E	Y	72.34	1131.5197	10	1.1	566.7677	2	33.52	2	F2:8772	12112021_RID_2349_DP2.raw	
K.SYELPDGQVITIGNER.F	Y	71.69	1789.8846	16	0.3	895.9499	2	53.88	2	F2:15567	12112021_RID_2349_DP2.raw	
K.DSYVGDEAQSK.R	Y	67.44	1197.5149	11	1.1	599.7654	2	23.86	2	F2:5370	12112021_RID_2349_DP2.raw	2.1509
R.VAPEEHPVLLTEAPLNPK.A	Y	63.91	1953.0570	18	0.6	977.5364	2	45.29	2	F2:12822	12112021_RID_2349_DP2.raw	
K.AGFAGDDAPR.A	Y	60.62	975.4410	10	0.5	488.7280	2	26.11	1	F1:7167	12112021_RID_2349_DP1.raw	3.3593
K.EITALAPSTMK.I	Y	52.44	1160.6111	11	0.0	581.3128	2	38.85	2	F2:10632	12112021_RID_2349_DP2.raw	
K.DSYVGDEAQSKR.G	Y	50.46	1353.6160	12	0.4	677.8156	2	20.64	2	F2:4244	12112021_RID_2349_DP2.raw	
K.EITALAPSTM(+15.99)K.I	Y	47.42	1176.6060	11	0.9	589.3108	2	32.91	2	F2:8537	12112021_RID_2349_DP2.raw	
R.AVFPSIVGRPR.H	Y	44.25	1197.6981	11	1.0	599.8569	2	40.62	2	F2:11273	12112021_RID_2349_DP2.raw	
K.DLYANTVLSGGTTM(+15.99)YPGIADR.M	Y	41.30	2230.0576	21	-0.2	1116.0359	2	56.05	2	F2:16292	12112021_RID_2349_DP2.raw	

ADK22827.1
back to list

| Protein Coverage | Supporting Peptides |
Protein Coverage:

1 MGRFISVSFR LLVVFLSLSG TRADFDCPSG WSAHDQHCYK AFDEPKRSGD AETFCTEQAN SGHLVSIESV EEAEFVAQLI

81 SENIKTPADY VWIGLRNQRK AQYCISKWTD GSSVIYKNVI ERFIKNCFGL EKQTDYRTWF NLSCGDDYPF VCKFPPRC

Supporting Peptides:

Peptide	Uniq	-10lgP	Mass	Length	ppm	m/z	z	RT	Fraction	Scan	Source File	Area F1	Area F2	Area
K.WTDGSSVIYK.N	N	77.27	1154.5608	10	0.0	578.2877	2	37.40	5	F5:10403	17112021_RID_2349_DP5.raw	2.1576E5	2.5151E5	1.7167
K.TPADYVWIGLR.N	Y	76.52	1289.6768	11	0.6	645.8461	2	57.87	7	F7:17384	17112021_RID_2349_DP7.raw			
K.WTDGSSVIYKNVIER.F	N	71.65	1765.8999	15	0.3	883.9575	2	44.92	5	F5:12969	17112021_RID_2349_DP5.raw			
F.VAQLISENIK.T	N	48.99	1113.6394	10	-0.4	557.8268	2	37.53	5	F5:10425	17112021_RID_2349_DP5.raw			
W.TDGSSVIYK.N	N	44.54	968.4814	9	0.1	485.2480	2	26.37	5	F5:6664	17112021_RID_2349_DP5.raw			
E.FVAQLISENIK.T	N	44.08	1260.7078	11	0.3	631.3613	2	49.39	5	F5:14370	17112021_RID_2349_DP5.raw			
K.AFDEPKR.S	N	40.54	861.4344	7	-0.5	431.7243	2	18.04	5	F5:3641	17112021_RID_2349_DP5.raw			
V.AQLISENIK.T	N	39.19	1014.5709	9	0.0	508.2927	2	33.55	5	F5:9140	17112021_RID_2349_DP5.raw			

total 8 pep

ADK22825.1
back to list

| Protein Coverage | Supporting Peptides |
Protein Coverage:

1 MGRFISVSFG LLVVFLSLSG TRADFDCPSG WSAHDQHCYK AFDEPKRSGD AETFCTEQAN SGHLVSIESV EEAEFVAQLI

81 SENIKTPADY VWIGLRNQRK AQYCISKWTD GSSVIYKNVI ERFIKNCFGL EKESDYRTWF NLSCGDDYPF VCKFPPRC

Supporting Peptides:

Peptide	Uniq	-10lgP	Mass	Length	ppm	m/z	z	RT	Fraction	Scan	Source File	Area F1	Area F2	Area I
K.WTDGSSVIYK.N	N	77.27	1154.5608	10	0.0	578.2877	2	37.40	5	F5:10403	17112021_RID_2349_DP5.raw	2.1576E5	2.5151E5	1.7167
K.TPADYVWIGLR.N	Y	76.52	1289.6768	11	0.6	645.8461	2	57.87	7	F7:17384	17112021_RID_2349_DP7.raw			
K.WTDGSSVIYKNVIER.F	N	71.65	1765.8999	15	0.3	883.9575	2	44.92	5	F5:12969	17112021_RID_2349_DP5.raw			
F.VAQLISENIK.T	N	48.99	1113.6394	10	-0.4	557.8268	2	37.53	5	F5:10425	17112021_RID_2349_DP5.raw			
W.TDGSSVIYK.N	N	44.54	968.4814	9	0.1	485.2480	2	26.37	5	F5:6664	17112021_RID_2349_DP5.raw			
E.FVAQLISENIK.T	N	44.08	1260.7078	11	0.3	631.3613	2	49.39	5	F5:14370	17112021_RID_2349_DP5.raw			
K.AFDEPKR.S	N	40.54	861.4344	7	-0.5	431.7243	2	18.04	5	F5:3641	17112021_RID_2349_DP5.raw			
V.AQLISENIK.T	N	39.19	1014.5709	9	0.0	508.2927	2	33.55	5	F5:9140	17112021_RID_2349_DP5.raw			

total 8 pep

ADN04916.1
back to list

| Protein Coverage | Supporting Peptides |
Protein Coverage:

```
  1 MVLIRVLANL LVLHLSYAQK SSELVIGGDE CNINEHPFLA LMYNSTSMKF HCSGTLLNEE WVLTAAHCDM ENMQIYLGVH
                                                  114
 81 DKNPNKDQQ TRVPKEMFFC LSNKSYTPWD KDIMLIRLNS PVTYSTHIAP FSLPSSPPTV GSVCRIMGWG AITSPNETYP
161 DVPHCANIEI YDYSVCRKAY GGLPEKSRTL CAGVLQGGID TCLADSGGPL ICNGQFQGIV AWGRHPCAQP QLPAFYTKVF
241 DYSDWIQSII AGNTAATCPS
```

Supporting Peptides:

Peptide	Uniq	-10lgP	Mass	Length	ppm	m/z	z	RT	Fraction	Scan	Source File	Area F1	Are
R.KAYGGLPEKSR.T	Y	66.51	1204.6564	11	-0.1	603.3354	2	21.80	2	F2:4728	12112021_RID_2349_DP2.raw	5.4582E5	2.00
K.AYGGLPEKSR.T	N	64.80	1076.5614	10	0.4	539.2882	2	22.20	2	F2:4846	12112021_RID_2349_DP2.raw		5.68
P.WDKDIMLIR.L	N	59.98	1188.6324	9	0.8	595.3240	2	47.61	2	F2:13483	12112021_RID_2349_DP2.raw		2.01
R.LNSPVTYSTH.I	Y	58.68	1117.5404	10	0.6	559.7778	2	32.48	2	F2:8433	12112021_RID_2349_DP2.raw		5.06
R.KAYGGLPEK.S	Y	58.02	961.5233	9	0.5	481.7692	2	22.09	2	F2:4938	12112021_RID_2349_DP2.raw		1.43
K.AYGGLPEK.S	N	56.12	833.4283	8	0.6	417.7217	2	26.79	2	F2:6388	12112021_RID_2349_DP2.raw	3.9735E5	1.69
R.LNSPVTYSTHIAPF.S	Y	54.49	1545.7827	14	0.3	773.8989	2	53.38	2	F2:15391	12112021_RID_2349_DP2.raw		3.47
P.WDKDIM(+15.99)LIR.L	N	54.14	1204.6274	9	0.6	603.3214	2	42.22	2	F2:11819	12112021_RID_2349_DP2.raw		1.42
R.LNSPVTYSTHIAPFSLPSSPPTVG.S	Y	49.67	2468.2588	24	0.8	1235.1377	2	61.33	2	F2:18069	12112021_RID_2349_DP2.raw		1.89
W.DKDIMLIR.L	N	44.22	1002.5532	8	-0.5	502.2836	2	37.38	3	F3:10341	17112021_RID_2349_DP3.raw		
C.AQPQLPAFYTK.V	Y	42.49	1262.6659	11	0.8	632.3407	2	45.16	2	F2:12753	12112021_RID_2349_DP2.raw		8.03
W.DKDIM(+15.99)LIR.L	N	39.37	1018.5481	8	0.3	510.2815	2	31.54	3	F3:8358	17112021_RID_2349_DP3.raw		

tota

E0Y418.1
back to list

| Protein Coverage | Supporting Peptides |
Protein Coverage:

1 MVLIRVLANL LVLHLSYAQK SSELVIGGDE CNINEHPFLA LMYNSTSMKF HCSGTLLNEE WVLTAAHCDM ENMQIYLGVH

114

81 DKKNPNKDQQ TRVPKEMFFC LSNKSYTPWD KDIMLIRLNS PVTYSTHIAP FSLPSSPPTV GSVCRIMGWG AITSPNETYP

161 DVPHCANIEI YDYSVCRKAY GGLPEKSRTL CAGVLQGGID TCLADSGGPL ICNGQFQGIV AWGRHPCAQP QLPAFYTKVF

241 DYSDWIQSII AGNTAATCPS

Supporting Peptides:

Peptide	Uniq	-10lgP	Mass	Length	ppm	m/z	z	RT	Fraction	Scan	Source File	Area F1	Are
R.KAYGGLPEKSR.T	Y	66.51	1204.6564	11	-0.1	603.3354	2	21.80	2	F2:4728	12112021_RID_2349_DP2.raw	5.4582E5	2.0
K.AYGGLPEKSR.T	N	64.80	1076.5614	10	0.4	539.2882	2	22.20	2	F2:4846	12112021_RID_2349_DP2.raw		5.6
P.WDKDIMLIR.L	N	59.98	1188.6324	9	0.8	595.3240	2	47.61	2	F2:13483	12112021_RID_2349_DP2.raw		2.01
R.LNSPVTYSTH.I	Y	58.68	1117.5404	10	0.6	559.7778	2	32.48	2	F2:8433	12112021_RID_2349_DP2.raw		5.0
R.KAYGGLPEK.S	Y	58.02	961.5233	9	0.5	481.7692	2	22.09	2	F2:4938	12112021_RID_2349_DP2.raw		1.4
K.AYGGLPEK.S	N	56.12	833.4283	8	0.6	417.7217	2	26.79	2	F2:6388	12112021_RID_2349_DP2.raw	3.9735E5	1.6
R.LNSPVTYSTHIAPF.S	Y	54.49	1545.7827	14	0.3	773.8989	2	53.38	2	F2:15391	12112021_RID_2349_DP2.raw		3.4
P.WDKDIM(+15.99)LIR.L	N	54.14	1204.6274	9	0.6	603.3214	2	42.22	2	F2:11819	12112021_RID_2349_DP2.raw		1.4
R.LNSPVTYSTHIAPFSLPSSPPTVG.S	Y	49.67	2468.2588	24	0.8	1235.1377	2	61.33	2	F2:18069	12112021_RID_2349_DP2.raw		1.8
W.DKDIMLIR.L	N	44.22	1002.5532	8	-0.5	502.2836	2	37.38	3	F3:10341	17112021_RID_2349_DP3.raw		
C.AQPQLPAFYTK.V	Y	42.49	1262.6659	11	0.8	632.3407	2	45.16	2	F2:12753	12112021_RID_2349_DP2.raw		8.0
W.DKDIM(+15.99)LIR.L	N	39.37	1018.5481	8	0.3	510.2815	2	31.54	3	F3:8358	17112021_RID_2349_DP3.raw		

tota

ADK22824.1
back to list

| Protein Coverage | Supporting Peptides |

Protein Coverage:

73

1 MGRFISISFG LLVVFLSLSG TGAKQDCLSD WSFYEGYCYK VFNEKKTWED AEKFCNEQVN GGYLVSFRSS EEMDFVIRMT

81 FPIFRFDFFW IGLRDFWRDC YWRWSDGVNL DYKAWSREPN CFVSKTTDNQ WLRWNCNDPR YFVCKSRVSC

Supporting Peptides:

Peptide	Uniq	-10lgP	Mass	Length	ppm	m/z	z	RT	Fraction	Scan	Source File	Area F1	Area F2	Area F3	Area F4	Ar
R.WSDGVNLDYK.A	Y	76.23	1195.5509	10	-0.2	598.7826	2	42.63	7	F7:12181	17112021_RID_2349_DP7.raw					
R.SSEEMDFVIR.M	Y	63.84	1211.5492	10	-0.1	606.7818	2	46.89	7	F7:13707	17112021_RID_2349_DP7.raw					
K.TTDNQWLR.W	N	59.51	1032.4989	8	0.4	517.2570	2	33.81	7	F7:9330	17112021_RID_2349_DP7.raw					8.7
R.FDFFWIGLR.D	Y	57.64	1199.6127	9	-0.2	600.8135	2	74.61	7	F7:23057	17112021_RID_2349_DP7.raw					
R.SSEEM(+15.99)DFVIR.M	Y	57.28	1227.5441	10	0.3	614.7795	2	44.29	7	F7:12932	17112021_RID_2349_DP7.raw					
W.SDGVNLDYK.A	Y	51.62	1009.4716	9	-0.2	505.7430	2	32.58	7	F7:8732	17112021_RID_2349_DP7.raw					
R.MTFPIFR.F	Y	47.81	910.4734	7	0.7	456.2443	2	54.20	7	F7:16176	17112021_RID_2349_DP7.raw					
K.TWEDAEK.F	N	42.92	877.3817	7	0.0	439.6982	2	23.16	7	F7:5532	17112021_RID_2349_DP7.raw					
S.SEEM(+15.99)DFVIR.M	Y	42.14	1140.5121	9	0.0	571.2633	2	38.69	7	F7:10887	17112021_RID_2349_DP7.raw					
K.KTWEDAEKF.C	N	40.81	1152.5450	9	0.6	577.2802	2	34.65	7	F7:9456	17112021_RID_2349_DP7.raw					
R.M(+15.99)TFPIFR.F	Y	40.76	926.4684	7	-0.1	464.2414	2	47.07	7	F7:13700	17112021_RID_2349_DP7.raw					

total 12 peptide

Peptide	Uniq	-10lgP	Mass	Length	ppm	m/z	z	RT	Fraction	Scan	Source File	Area F1	Area F2	Area F3	Area F4	Ar
K.TWEDAEKF.C	N	40.38	1024.4502	8	-0.9	513.2319	2	40.86	7	F7:11654	17112021_RID_2349_DP7.raw					

total 12 peptide

AAY63876.1
back to list

| Protein Coverage | Supporting Peptides |
Protein Coverage:

1 MGRFISISFG LLVVFLSLSG TGAKQDCLSD WSFYEGYCYK VFNEKKTWED AEKFCNEQVN GGYLVSFRSS EEMDFVIRMT C

81 FPIFRFDFFW IGLRDFWRDC YWRWSDGVNL DYKAWSREPN CFVSKTTDNQ WLRWNCNDPR YFVCKSRVSC

Supporting Peptides:

Peptide	Uniq	-10lgP	Mass	Length	ppm	m/z	z	RT	Fraction	Scan	Source File	Area F1	Area F2	Area F3	Area F4	Ar
R.WSDGVNLDYK.A	Y	76.23	1195.5509	10	-0.2	598.7826	2	42.63	7	F7:12181	17112021_RID_2349_DP7.raw					
R.SSEEMDFVIR.M	Y	63.84	1211.5492	10	-0.1	606.7818	2	46.89	7	F7:13707	17112021_RID_2349_DP7.raw					
K.TTDNQWLR.W	N	59.51	1032.4989	8	0.4	517.2570	2	33.81	7	F7:9330	17112021_RID_2349_DP7.raw					8.7
R.FDFFWIGLR.D	Y	57.64	1199.6127	9	-0.2	600.8135	2	74.61	7	F7:23057	17112021_RID_2349_DP7.raw					
R.SSEEM(+15.99)DFVIR.M	Y	57.28	1227.5441	10	0.3	614.7795	2	44.29	7	F7:12932	17112021_RID_2349_DP7.raw					
W.SDGVNLDYK.A	Y	51.62	1009.4716	9	-0.2	505.7430	2	32.58	7	F7:8732	17112021_RID_2349_DP7.raw					
R.MTFPIFR.F	Y	47.81	910.4734	7	0.7	456.2443	2	54.20	7	F7:16176	17112021_RID_2349_DP7.raw					
K.TWEDAEK.F	N	42.92	877.3817	7	0.0	439.6982	2	23.16	7	F7:5532	17112021_RID_2349_DP7.raw					
S.SEEM(+15.99)DFVIR.M	Y	42.14	1140.5121	9	0.0	571.2633	2	38.69	7	F7:10887	17112021_RID_2349_DP7.raw					
K.KTWEDAEKF.C	N	40.81	1152.5450	9	0.6	577.2802	2	34.65	7	F7:9456	17112021_RID_2349_DP7.raw					
R.M(+15.99)TFPIFR.F	Y	40.76	926.4684	7	-0.1	464.2414	2	47.07	7	F7:13700	17112021_RID_2349_DP7.raw					
K.TWEDAEKF.C	N	40.38	1024.4502	8	-0.9	513.2319	2	40.86	7	F7:11654	17112021_RID_2349_DP7.raw					

total 12 peptide

ADK22822.1
back to list

| Protein Coverage | Supporting Peptides |
Protein Coverage:

1 MGRFISVGFG LLVVFLSLSG TGAKQDCLSD WSFYEGYCYK VFNEKKTWED AEKFCNEQVN GGYLVSFRSS EEMDFVIRMT C

81 FPIFRFDFFW IGLRDFWRDC YWRWSDGVNL DYKAWSREPN CFVSKTTDNQ WLRWNCNDPR YFVCKSRVSC

Supporting Peptides:

Peptide	Uniq	-10lgP	Mass	Length	ppm	m/z	z	RT	Fraction	Scan	Source File	Area F1	Area F2	Area F3	Area F4	Ar
R.WSDGVNLDYK.A	Y	76.23	1195.5509	10	-0.2	598.7826	2	42.63	7	F7:12181	17112021_RID_2349_DP7.raw					
R.SSEEMDFVIR.M	Y	63.84	1211.5492	10	-0.1	606.7818	2	46.89	7	F7:13707	17112021_RID_2349_DP7.raw					
K.TTDNQWLR.W	N	59.51	1032.4989	8	0.4	517.2570	2	33.81	7	F7:9330	17112021_RID_2349_DP7.raw					8.7
R.FDFFWIGLR.D	Y	57.64	1199.6127	9	-0.2	600.8135	2	74.61	7	F7:23057	17112021_RID_2349_DP7.raw					
R.SSEEM(+15.99)DFVIR.M	Y	57.28	1227.5441	10	0.3	614.7795	2	44.29	7	F7:12932	17112021_RID_2349_DP7.raw					
W.SDGVNLDYK.A	Y	51.62	1009.4716	9	-0.2	505.7430	2	32.58	7	F7:8732	17112021_RID_2349_DP7.raw					
R.MTFPIFR.F	Y	47.81	910.4734	7	0.7	456.2443	2	54.20	7	F7:16176	17112021_RID_2349_DP7.raw					
K.TWEDAEK.F	N	42.92	877.3817	7	0.0	439.6982	2	23.16	7	F7:5532	17112021_RID_2349_DP7.raw					
S.SEEM(+15.99)DFVIR.M	Y	42.14	1140.5121	9	0.0	571.2633	2	38.69	7	F7:10887	17112021_RID_2349_DP7.raw					

total 12 peptide

Peptide	Uniq	-10lgP	Mass	Length	ppm	m/z	z	RT	Fraction	Scan	Source File	Area F1	Area F2	Area F3	Area F4	Ar
K.KTWEDAEKF.C	N	40.81	1152.5450	9	0.6	577.2802	2	34.65	7	F7:9456	17112021_RID_2349_DP7.raw					
R.M(+15.99)TFPIFR.F	Y	40.76	926.4684	7	-0.1	464.2414	2	47.07	7	F7:13700	17112021_RID_2349_DP7.raw					
K.TWEDAEKF.C	N	40.38	1024.4502	8	-0.9	513.2319	2	40.86	7	F7:11654	17112021_RID_2349_DP7.raw					

total 12 peptide

Q4PRC6.1
back to list

| Protein Coverage | Supporting Peptides |
Protein Coverage:

1 MGRFISISFG LLVVFLSLSG TGAKQDCLSD WSFYEGYCYK VFNEKKTWED AEKFCNEQVN GGYLVSFRSS EEMDFVIRMT

81 FPIFRFDFFW IGLRDFWRDC YWRWSDGVNL DYKAWSREPN CFVSKTTDNQ WLRWNCNDPR YFVCKSRVSC

Supporting Peptides:

Peptide	Uniq	-10lgP	Mass	Length	ppm	m/z	z	RT	Fraction	Scan	Source File	Area F1	Area F2	Area F3	Area F4	Ar
R.WSDGVNLDYK.A	Y	76.23	1195.5509	10	-0.2	598.7826	2	42.63	7	F7:12181	17112021_RID_2349_DP7.raw					
R.SSEEMDFVIR.M	Y	63.84	1211.5492	10	-0.1	606.7818	2	46.89	7	F7:13707	17112021_RID_2349_DP7.raw					
K.TTDNQWLR.W	N	59.51	1032.4989	8	0.4	517.2570	2	33.81	7	F7:9330	17112021_RID_2349_DP7.raw					8.7
R.FDFFWIGLR.D	Y	57.64	1199.6127	9	-0.2	600.8135	2	74.61	7	F7:23057	17112021_RID_2349_DP7.raw					
R.SSEEM(+15.99)DFVIR.M	Y	57.28	1227.5441	10	0.3	614.7795	2	44.29	7	F7:12932	17112021_RID_2349_DP7.raw					
W.SDGVNLDYK.A	Y	51.62	1009.4716	9	-0.2	505.7430	2	32.58	7	F7:8732	17112021_RID_2349_DP7.raw					
R.MTFPIFR.F	Y	47.81	910.4734	7	0.7	456.2443	2	54.20	7	F7:16176	17112021_RID_2349_DP7.raw					
K.TWEDAEK.F	N	42.92	877.3817	7	0.0	439.6982	2	23.16	7	F7:5532	17112021_RID_2349_DP7.raw					
S.SEEM(+15.99)DFVIR.M	Y	42.14	1140.5121	9	0.0	571.2633	2	38.69	7	F7:10887	17112021_RID_2349_DP7.raw					
K.KTWEDAEKF.C	N	40.81	1152.5450	9	0.6	577.2802	2	34.65	7	F7:9456	17112021_RID_2349_DP7.raw					
R.M(+15.99)TFPIFR.F	Y	40.76	926.4684	7	-0.1	464.2414	2	47.07	7	F7:13700	17112021_RID_2349_DP7.raw					
K.TWEDAEKF.C	N	40.38	1024.4502	8	-0.9	513.2319	2	40.86	7	F7:11654	17112021_RID_2349_DP7.raw					

total 12 peptide

TRINITY_DN459
back to list

| Protein Coverage | Supporting Peptides |
Protein Coverage:

1 GQSAECPTDV FQRNGQPCQN NKGYCYNGDC PILTNQCISL FGSSVTVAPD VCFDYNLQGT EDYHCRRENG RYIKCEPQDK

81 KCGRLFCVKA PTGNAIKCKI PYSPDDPDYG MVDLGTKCAD GKVCNSNRQC VDVNTA

Supporting Peptides:

Peptide	Uniq	-10lgP	Mass	Length	ppm	m/z	z	RT	Fraction	Scan	Source File	Area F1	Ar
P.DDDPDYGMVDLGTK.C	N	86.80	1424.6129	13	0.9	713.3144	2	48.02	1	F1:14542	12112021_RID_2349_DP1.raw	3.1015E5	
K.IPYSPDDPDYGMVDLGTK.C	Y	73.90	1981.8978	18	1.1	991.9573	2	53.28	3	F3:15640	17112021_RID_2349_DP3.raw	9.0784E5	1.1
D.PDYGMVDLGTK.C	N	73.86	1194.5591	11	-0.1	598.2867	2	43.94	1	F1:13219	12112021_RID_2349_DP1.raw	7.4048E6	4.1
D.PDYGM(+15.99)VDLGTK.C	N	71.86	1210.5540	11	0.4	606.2845	2	40.64	1	F1:13230	12112021_RID_2349_DP1.raw	6.7718E6	3.5
P.DDPDYGM(+15.99)VDLGTK.C	N	69.19	1440.6078	13	0.3	721.3114	2	40.35	6	F6:11298	17112021_RID_2349_DP6.raw	7.0367E4	

Peptide	Uniq	-10lgP	Mass	Length	ppm	m/z	z	RT	Fraction	Scan	Source File	Area F1
D.DPDYGMVDLGTK.C	N	68.28	1309.5859	12	1.2	655.8010	2	47.41	1	F1:14315	12112021_RID_2349_DP1.raw	7.5385E5
D.DPDYGM(+15.99)VDLGTK.C	N	61.75	1325.5809	12	0.8	663.7983	2	39.96	1	F1:11872	12112021_RID_2349_DP1.raw	2.7576E5
K.IPYSPDDPDYGM(+15.99)VDLGTK.C	Y	56.68	1997.8928	18	0.4	999.9541	2	47.71	4	F4:13784	17112021_RID_2349_DP4.raw	2.172E5
D.YGM(+15.99)VDLGTK.C	N	54.16	998.4742	9	0.8	500.2448	2	39.32	1	F1:11674	12112021_RID_2349_DP1.raw	1.0157E5
D.YGMVDLGTK.C	N	45.13	982.4794	9	-0.5	492.2467	2	39.32	1	F1:11699	12112021_RID_2349_DP1.raw	1.3963E5
Y.GMVDLGTK.C	N	44.20	819.4160	8	0.0	410.7153	2	30.74	7	F7:8124	17112021_RID_2349_DP7.raw	

TRINITY_DN157
back to list

| Protein Coverage | Supporting Peptides |
Protein Coverage:

```
  1 ALQATGPAGA LLRPGSRSRT GKSDLLRPPP DRGRGGAAGR GAGQARRRVR ARPAPGRRGC RARGIKVGPE VRAQLPPSFS
 81 PTASRALRPP SASMVHWSAE EKQLITSLWS KVDVPEVGGA TLGKLLVVYP WTQRFFAHFG NLSGPSAICG NPLVKAHGKK
161 VLTSFGEAIK HLDNIKETFA KLSELHCDKL HVDPENFKLL GNILIIVLAG HHGKEFTPST HAAFQKLTHV VAHSLALRYH
```

Supporting Peptides:

Peptide	Uniq	-10lgP	Mass	Length	ppm	m/z	z	RT	Fraction	Scan	Source File	Area F1	Area F2	Area F3
K.VDVPEVGGATLGK.L	Y	80.51	1240.6663	13	0.6	621.3408	2	42.05	7	F7:11994	17112021_RID_2349_DP7.raw			
K.LLVVYPWTQR.F	N	62.42	1273.7183	10	0.7	637.8668	2	56.17	4	F4:16617	17112021_RID_2349_DP4.raw	5.7141E5	2.1702E5	1.0063E6
K.HLDNIKETFAK.L	Y	61.67	1314.6931	11	0.6	439.2386	3	30.74	7	F7:8165	17112021_RID_2349_DP7.raw			
K.VLTSFGEAIK.H	N	61.56	1063.5913	10	-0.1	532.8029	2	41.86	7	F7:11920	17112021_RID_2349_DP7.raw			
M.VHWSAEEK.Q	N	52.31	984.4665	8	0.7	493.2408	2	21.02	7	F7:4832	17112021_RID_2349_DP7.raw			
K.EFTPSTHAAFQK.L	N	50.12	1362.6567	12	0.3	682.3358	2	30.43	7	F7:8112	17112021_RID_2349_DP7.raw			
K.KVLTSFGEAIK.H	N	48.51	1191.6863	11	1.5	596.8513	2	36.27	7	F7:10106	17112021_RID_2349_DP7.raw			
K.QLITSLWSK.V	Y	42.95	1074.6073	9	-0.1	538.3109	2	50.81	7	F7:15061	17112021_RID_2349_DP7.raw			
K.LTHVVAHSLALR.Y	Y	42.42	1315.7725	12	0.2	439.5982	3	32.34	7	F7:8705	17112021_RID_2349_DP7.raw			

total 9 pept

P18964.1
back to list

| Protein Coverage | Supporting Peptides |
Protein Coverage:

```
  1 VVGGDECNIN EHPFLVALYT STSSTIHCGG ALINREWVLT AAHCDRRNIR IKLGMHSKNI RNEDEQIRVP RGKYFCLNTK
 81 FPNGLDKDIM LIRLRRPVTY STHIAPVSLP SRSRGVGSRC RIMGWGKIST TEDTYPDVPH CTNIFIVKHK WCEPLYPWVP
161 ADSRTLCAGI LKGGRDTCHG DSGGPLICNG QIQGIVAGGS EPCGQHLKPA VYTKVFDYNN WIQNIIAGNR TVTCPP
```

Supporting Peptides:

Peptide	Uniq	-10lgP	Mass	Length	ppm	m/z	z	RT	Fraction	Scan	Source File	Area F1	Area F2	Area F3
K.FPNGLDKDIMLIR.L	Y	66.83	1530.8228	13	0.2	511.2816	3	54.26	3	F3:15907	17112021_RID_2349_DP3.raw			2.7289E6
Y.STHIAPVSLPSR.S	Y	63.76	1263.6935	12	0.2	632.8541	2	34.00	7	F7:9308	17112021_RID_2349_DP7.raw			
R.NEDEQIRVPR.G	Y	62.99	1254.6316	10	0.2	628.3232	2	27.46	7	F7:7050	17112021_RID_2349_DP7.raw			7.022E6
K.NIRNEDEQIRVPR.G	Y	61.00	1637.8597	13	0.5	546.9608	3	29.37	7	F7:7736	17112021_RID_2349_DP7.raw			2.8569E6
R.RPVTYSTHIAPVSLPSR.S	Y	57.58	1880.0267	17	0.6	627.6832	3	39.74	4	F4:11137	17112021_RID_2349_DP4.raw			1.6115E7
K.NIRNEDEQIR.V	Y	56.31	1285.6375	10	0.5	643.8263	2	20.61	7	F7:4677	17112021_RID_2349_DP7.raw			7.3183E5

total 10 pe

Peptide	Uniq	-10lgP	Mass	Length	ppm	m/z	z	RT	Fraction	Scan	Source File	Area F1	Area F2	Area F3
K.FPNGLDKDIM(+15.99)LIR.L	Y	47.93	1546.8177	13	0.2	516.6133	3	50.38	3	F3:14752	17112021_RID_2349_DP3.raw			2.1323E5
L.DKDIMLIR.L	N	44.22	1002.5532	8	-0.5	502.2836	2	37.38	3	F3:10341	17112021_RID_2349_DP3.raw			5.8616E6
K.FPNGLDK.D	Y	41.98	789.4021	7	1.3	395.7088	2	29.86	7	F7:7802	17112021_RID_2349_DP7.raw			
L.DKDIM(+15.99)LIR.L	N	39.37	1018.5481	8	0.3	510.2815	2	31.54	3	F3:8358	17112021_RID_2349_DP3.raw			1.7097E6

total 10 pe[illegible]

ADP88558.1
back to list

| Protein Coverage | Supporting Peptides |
Protein Coverage:

1 MVLIKVLANL LVLQLSYAQK SSELVVGGDE CNINEHPFLV ALYTSASSTI HCAGALINRE WVLTAAHCDR RNIRIKLGMH

114
81 SK**NIRNEDEQ IRVPR**GKYFC LNTK**FPNGLD KDIMLIR**LR**R PVTYSTHIAP VSLPSR**SRGV GSRCRIMGWG KISTTEDTYP

161 DVPHCTNIFI VKHKWCEPLY PWVPADSRTL CAGILKGGRD TCKGDSGGPL ICNGEMHGIV AGGSEPCGQH LKPAVYTKVF

241 DYNNWIQSII AGNRTVTCPP

Supporting Peptides:

Peptide	Uniq	-10lgP	Mass	Length	ppm	m/z	z	RT	Fraction	Scan	Source File	Area F1	Area F2	Area F3
K.FPNGLDKDIMLIR.L	Y	66.83	1530.8228	13	0.2	511.2816	3	54.26	3	F3:15907	17112021_RID_2349_DP3.raw			2.7289E6
Y.STHIAPVSLPSR.S	Y	63.76	1263.6935	12	0.2	632.8541	2	34.00	7	F7:9308	17112021_RID_2349_DP7.raw			
R.NEDEQIRVPR.G	Y	62.99	1254.6316	10	0.2	628.3232	2	27.46	7	F7:7050	17112021_RID_2349_DP7.raw			7.022E6
K.NIRNEDEQIRVPR.G	Y	61.00	1637.8597	13	0.5	546.9608	3	29.37	7	F7:7736	17112021_RID_2349_DP7.raw			2.8569E6
R.RPVTYSTHIAPVSLPSR.S	Y	57.58	1880.0267	17	0.6	627.6832	3	39.74	4	F4:11137	17112021_RID_2349_DP4.raw			1.6115E7
K.NIRNEDEQIR.V	Y	56.31	1285.6375	10	0.5	643.8263	2	20.61	7	F7:4677	17112021_RID_2349_DP7.raw			7.3183E5
K.FPNGLDKDIM(+15.99)LIR.L	Y	47.93	1546.8177	13	0.2	516.6133	3	50.38	3	F3:14752	17112021_RID_2349_DP3.raw			2.1323E5
L.DKDIMLIR.L	N	44.22	1002.5532	8	-0.5	502.2836	2	37.38	3	F3:10341	17112021_RID_2349_DP3.raw			5.8616E6
K.FPNGLDK.D	Y	41.98	789.4021	7	1.3	395.7088	2	29.86	7	F7:7802	17112021_RID_2349_DP7.raw			
L.DKDIM(+15.99)LIR.L	N	39.37	1018.5481	8	0.3	510.2815	2	31.54	3	F3:8358	17112021_RID_2349_DP3.raw			1.7097E6

total 10 pe[illegible]

P18965.2
back to list

| Protein Coverage | Supporting Peptides |
Protein Coverage:

1 MVLIKVLANL LVLQLSYAQK SSELVVGGDE CNINEHPFLV ALYTSASSTI HCAGALINRE WVLTAAHCDR RNIRIKLGMH

114
81 SK**NIRNEDEQ IRVPR**GKYFC LNTK**FPNGLD KDIMLIR**LR**R PVTYSTHIAP VSLPSR**SRGV GSRCRIMGWG KISTTEDTYP

161 DVPHCTNIFI VKHKWCEPLY PWVPADSRTL CAGILKGGRD TCHGDSGGPL ICNGEMHGIV AGGSEPCGQH LKPAVYTKVF

241 DYNNWIQSII AGNRTVTCPP

Supporting Peptides:

Peptide	Uniq	-10lgP	Mass	Length	ppm	m/z	z	RT	Fraction	Scan	Source File	Area F1	Area F2	Area F3
K.FPNGLDKDIMLIR.L	Y	66.83	1530.8228	13	0.2	511.2816	3	54.26	3	F3:15907	17112021_RID_2349_DP3.raw			2.7289E6
Y.STHIAPVSLPSR.S	Y	63.76	1263.6935	12	0.2	632.8541	2	34.00	7	F7:9308	17112021_RID_2349_DP7.raw			
R.NEDEQIRVPR.G	Y	62.99	1254.6316	10	0.2	628.3232	2	27.46	7	F7:7050	17112021_RID_2349_DP7.raw			7.022E6
K.NIRNEDEQIRVPR.G	Y	61.00	1637.8597	13	0.5	546.9608	3	29.37	7	F7:7736	17112021_RID_2349_DP7.raw			2.8569E6
R.RPVTYSTHIAPVSLPSR.S	Y	57.58	1880.0267	17	0.6	627.6832	3	39.74	4	F4:11137	17112021_RID_2349_DP4.raw			1.6115E7

total 10 pe[illegible]

Peptide	Uniq	-10lgP	Mass	Length	ppm	m/z	z	RT	Fraction	Scan	Source File	Area F1	Area F2	Area F3
K.NIRNEDEQIR.V	Y	56.31	1285.6375	10	0.5	643.8263	2	20.61	7	F7:4677	17112021_RID_2349_DP7.raw			7.3183E5 [illegible]
K.FPNGLDKDIM(+15.99)LIR.L	Y	47.93	1546.8177	13	0.2	516.6133	3	50.38	3	F3:14752	17112021_RID_2349_DP3.raw			2.1323E5
L.DKDIMLIR.L	N	44.22	1002.5532	8	-0.5	502.2836	2	37.38	3	F3:10341	17112021_RID_2349_DP3.raw			5.8616E6
K.FPNGLDK.D	Y	41.98	789.4021	7	1.3	395.7088	2	29.86	7	F7:7802	17112021_RID_2349_DP7.raw			[illegible]
L.DKDIM(+15.99)LIR.L	N	39.37	1018.5481	8	0.3	510.2815	2	31.54	3	F3:8358	17112021_RID_2349_DP3.raw			1.7097E6

total 10 pe[illegible]

TRINITY_DN2646
back to list

| Protein Coverage | Supporting Peptides |
Protein Coverage:

1 MAPGGWMLLA GLLLLLGGLD RPSSGSAAGP VPLVIWHGMG DSCCNPLSMG YIKKIVEEK**I PGIYVLSLK**I GNTLIDDMEN

110

81 SFFMDVNNQV KKVCDQLSKD PK**LQGGYNAM GFSQGGQFLR** AVVQRCPSPP MFNLISIGGQ HQGVFGFPHC PGESSHLCDW

161 LRKVLNIAYT DKIQK**HLVQA QYWHDPVNEQ QYR**R**SSLFLA DINQER**VINE TYKKNLMTLK KFVMVRFLND TMVDPPASEW

241 FGYYRSGQTK **ETIPLQETTL YTEDR**LGLKE MDQAGKLVFL GAEGDHLRFS KEWFFK**NIVP FLSDAAGDDS QA**VLS

Supporting Peptides:

Peptide	Uniq	-10lgP	Mass	Length	ppm	m/z	z	RT	Fraction	Scan	Source File	Area F1	Area
K.LQGGYNAMGFSQGGQFLR.A	Y	84.07	1929.9155	18	0.4	965.9654	2	52.55	3	F3:15482	17112021_RID_2349_DP3.raw		1.037
K.ETIPLQETTLYTEDR.L	Y	70.72	1807.8839	15	0.1	904.9493	2	48.94	2	F2:13963	12112021_RID_2349_DP2.raw		9.553
R.SSLFLADINQER.V	Y	67.59	1391.7045	12	0.3	696.8597	2	50.21	3	F3:14633	17112021_RID_2349_DP3.raw		1.288
K.IPGIYVLSLK.I	Y	62.71	1101.6797	10	0.6	551.8475	2	57.36	3	F3:16947	17112021_RID_2349_DP3.raw		
K.NIVPFLSDAAGDDSQA.V	Y	54.50	1618.7474	16	0.8	810.3817	2	64.67	3	F3:19306	17112021_RID_2349_DP3.raw		5.298
K.HLVQAQYWHDPVNEQQYR.R	Y	46.16	2310.0930	18	0.6	771.0388	3	40.68	3	F3:11579	17112021_RID_2349_DP3.raw		
K.LQGGYNAM(+15.99)GFSQGGQFLR.A	Y	39.61	1945.9104	18	0.7	973.9631	2	47.17	3	F3:13705	17112021_RID_2349_DP3.raw		

total 7 [illegible]

TRINITY_DN2646
back to list

| Protein Coverage | Supporting Peptides |
Protein Coverage:

1 MAPGGWMLLA GLLLLLGGLD RPSSGSAAGP VPLVIWHGMG DSCCNPLSMG YIKKIVEEK**I PGIYVLSLK**I GNTLIDDMEN

110

81 SFFMDVNNQV KKVCDQLSKD PK**LQGGYNAM GFSQGGQFLR** AVVQRCPSPP MFNLISIGGQ HQGVFGFPHC PGESSHLCDW

161 LRKVLNIAYT DKIQK**HLVQA QYWHDPVNEQ QYR**R**SSLFLA DINQER**VINE TYKKNLMTLK KFVMVRFLND TMVDPPASEW

241 FGYYRSGQTK **ETIPLQETTL YTEDR**LGLKE MDQAGKLVFL GAEGDHLRFS KEWFFK**NIVP FLSDAAGDDS QA**VLS

Supporting Peptides:

Peptide	Uniq	-10lgP	Mass	Length	ppm	m/z	z	RT	Fraction	Scan	Source File	Area F1	Area
K.LQGGYNAMGFSQGGQFLR.A	Y	84.07	1929.9155	18	0.4	965.9654	2	52.55	3	F3:15482	17112021_RID_2349_DP3.raw		1.037
K.ETIPLQETTLYTEDR.L	Y	70.72	1807.8839	15	0.1	904.9493	2	48.94	2	F2:13963	12112021_RID_2349_DP2.raw		9.553
R.SSLFLADINQER.V	Y	67.59	1391.7045	12	0.3	696.8597	2	50.21	3	F3:14633	17112021_RID_2349_DP3.raw		1.288
K.IPGIYVLSLK.I	Y	62.71	1101.6797	10	0.6	551.8475	2	57.36	3	F3:16947	17112021_RID_2349_DP3.raw		
K.NIVPFLSDAAGDDSQA.V	Y	54.50	1618.7474	16	0.8	810.3817	2	64.67	3	F3:19306	17112021_RID_2349_DP3.raw		5.298
K.HLVQAQYWHDPVNEQQYR.R	Y	46.16	2310.0930	18	0.6	771.0388	3	40.68	3	F3:11579	17112021_RID_2349_DP3.raw		
K.LQGGYNAM(+15.99)GFSQGGQFLR.A	Y	39.61	1945.9104	18	0.7	973.9631	2	47.17	3	F3:13705	17112021_RID_2349_DP3.raw		

total 7 [illegible]

TRINITY_DN300
back to list

| Protein Coverage | Supporting Peptides |

Protein Coverage:

```
  1 MAKSESQMDI TEMNSPKPKR KLRWSPLEIG LAAVVLLLAI VAITMIILYA TYDDGICKTS DCIKSAARII ENMDTTAEPC
 81 SNFYQYACGG WLKKHIIPET SSRYSNFDIL RDELEVVLKD VLEKKDSNDI DAVAKAKTLY RSCINETAIN SKKGKPLINL
161 MTNISEWPIA AESWDGSTWS AEKSIAELNF QFGRKVIINM FVGTDDKNSS QHVLHVDQPR LGLPSRDYYE CTGLYKEACS
241 AYESFMTSVA KLIRQERNLP INDTEISIEA ERIMALEKEI ANVTTKDEDR NDPLLLYHKM KLSDLQKNFS MEINNKTFDW
321 IKFVNNIMTT VAIEVKNTED IIVYAPEYLI KLKSVISKYS AREIQNYISW RYIMDMVSSL SREYKDTRNN FRKVLYGTTS
401 DAAIWRRCAN YVNSIMGNAV GRLYVQEAFA GESKHVVQEM ITQIREVFIK TLEELSWMDA KTKKKAEEKA VAIKERIGYP
481 DEILTNPVKL NQEYKGLNYK EEEYFENLIE NLKFGQKERL KKLREKVDKD EWITGAAVVN AFYSPNRNQI VFPAGILQPP
561 FFSASQPKSL NFGGIGMVIG HEITHGFDDN GRNFNAQGDL VDWWSQESAN NFKSQSRCMI YQYGNFTWDL AGGQNLSGIN
641 TLGENIADNG GIRQAYRAYE SYVQKNGEEK RLPGLDLNHK QLFFLNFAQI WCGTYRPEYA VNSIKTDVHS PGKFRVLGSL
721 QNNLEFSEAF KCTDKDYMDP VKKCRVW
```

Supporting Peptides:

Peptide	Uniq	-10lgP	Mass	Length	ppm	m/z	z	RT	Fraction	Scan	Source File	Area F1	Area F2	Ar F
K.SIAELNFQFGR.K	Y	73.70	1280.6512	11	0.1	641.3329	2	55.39	1	F1:16912	12112021_RID_2349_DP1.raw	5.654E4		
R.IGYPDEILTNPVK.L	Y	70.66	1457.7766	13	0.2	729.8957	2	50.37	1	F1:15355	12112021_RID_2349_DP1.raw	5.763E5		
R.YIMDMVSSLSR.E	Y	62.87	1300.6155	11	0.5	651.3153	2	54.99	1	F1:16794	12112021_RID_2349_DP1.raw	6.491E4		
R.AYESYVQK.N	Y	61.21	986.4709	8	-0.6	494.2424	2	26.11	1	F1:7228	12112021_RID_2349_DP1.raw	5.2803E5		
R.EIQNYISWR.Y	Y	55.38	1207.5985	9	0.2	604.8066	2	48.42	1	F1:14642	12112021_RID_2349_DP1.raw	5.0876E4		
R.NQIVFPAGILQPPFFSASQPK.S	Y	53.84	2285.2207	21	1.1	1143.6189	2	71.77	1	F1:22334	12112021_RID_2349_DP1.raw	1.8483E5		
R.LYVQEAFAGESK.H	Y	53.64	1340.6611	12	0.4	671.3381	2	42.18	1	F1:12626	12112021_RID_2349_DP1.raw	1.0438E5		
K.TDVHSPGKFR.V	Y	49.57	1142.5833	10	-0.4	381.8682	3	22.83	1	F1:6017	12112021_RID_2349_DP1.raw	1.6839E5		

total 8 peptides

TRINITY_DN300
back to list

| Protein Coverage | Supporting Peptides |

Protein Coverage:

```
  1 MAKSESQMDI TEMNSPKPKR KLRWSPLEIG LAAVVLLLAI VAITMIILYA TYDDGICKTS DCIKSAARII ENMDTTAEPC
 81 SNFYQYACGG WLKKHIIPET SSRYSNFDIL RDELEVVLKD VLEKKDSNDI DAVAKAKTLY RSCINETAIN SKKGKPLINL
161 MTNISEWPIA AESWDGSTWS AEKSIAELNF QFGRKVIINM FVGTDDKNSS QHVLHVDQPR LGLPSRDYYE CTGLYKEACS
241 AYESFMTSVA KLIRQERNLP INDTEISIEA ERIMALEKEI ANVTTKDEDR NDPLLLYHKM KLSDLQKNFS MEINNKTFDW
321 IKFVNNIMTT VAIEVKNTED IIVYAPEYLI KLKSVISKYS AREIQNYISW RYIMDMVSSL SREYKDTRNN FRKVLYGTTS
401 DAAIWRRCAN YVNSIMGNAV GRLYVQEAFA GESKHVVQEM ITQIREVFIK TLEELSWMDA KTKKKAEEKA VAIKERIGYP
481 DEILTNPVKL NQEYKGLNYK EEEYFENLIE NLKFGQKERL KKLREKVDKD EWITGAAVVN AFYSPNRNQI VFPAGILQPP
561 FFSASQPKSL NFGGIGMVIG HEITHGFDDN GRNFNAQGDL VDWWSQESAN NFKSQSRCMI YQYGNFTWDL AGGQNLSGIN
641 TLGENIADNG GIRQAYRAYE SYVQKNGEEK RLPGLDLNHK QLFFLNFAQI WCGTYRPEYA VNSIKTDVHS PGKFRVLGSL
721 QNNLEFSEAF KCTDKDYMDP VKKCRVW
```

Supporting Peptides:

Peptide	Uniq	-10lgP	Mass	Length	ppm	m/z	z	RT	Fraction	Scan	Source File	Area F1	Area F2	Ar F
K.SIAELNFQFGR.K	Y	73.70	1280.6512	11	0.1	641.3329	2	55.39	1	F1:16912	12112021_RID_2349_DP1.raw	5.654E4		
R.IGYPDEILTNPVK.L	Y	70.66	1457.7766	13	0.2	729.8957	2	50.37	1	F1:15355	12112021_RID_2349_DP1.raw	5.763E5		
R.YIMDMVSSLSR.E	Y	62.87	1300.6155	11	0.5	651.3153	2	54.99	1	F1:16794	12112021_RID_2349_DP1.raw	6.491E4		
R.AYESYVQK.N	Y	61.21	986.4709	8	-0.6	494.2424	2	26.11	1	F1:7228	12112021_RID_2349_DP1.raw	5.2803E5		
R.EIQNYISWR.Y	Y	55.38	1207.5985	9	0.2	604.8066	2	48.42	1	F1:14642	12112021_RID_2349_DP1.raw	5.0876E4		

total 8 peptides

Peptide	Uniq	-10lgP	Mass	Length	ppm	m/z	z	RT	Fraction	Scan	Source File	Area F1	Area F2	Ar F
R.NQIVFPAGILQPPFFSASQPK.S	Y	53.84	2285.2207	21	1.1	1143.6189	2	71.77	1	F1:22334	12112021_RID_2349_DP1.raw	1.8483E5		
R.LYVQEAFAGESK.H	Y	53.64	1340.6611	12	0.4	671.3381	2	42.18	1	F1:12626	12112021_RID_2349_DP1.raw	1.0438E5		
K.TDVHSPGKFR.V	Y	49.57	1142.5833	10	-0.4	381.8682	3	22.83	1	F1:6017	12112021_RID_2349_DP1.raw	1.6839E5		

total 8 peptides

B8K1W0.1
back to list

| Protein Coverage | Supporting Peptides |
Protein Coverage:

1 MMQVLLVTIC LAVFPYHGSS IILESGNVND YEVVYPQKVT AMPKEAVKQP EQKYEDAMQY KFEVNGEPVL LHLEKNKDLF
81 SEDYSETHYS PDGREITTKP LVQDHCYYHG HIQNDAHSSA SISACNGLKG HFKLRGEMYL IEPLKLSDSE AHAVYKYENV
161 EKEDEALKMC GV**TQTNWESD EPIKKASLLV ATSER**NRYFN PYSYVELIIT VDHSMVTK**YK NDLTAIR**TWV FELVNTINEI
241 FKYLYIR**VPL VGLEIWK**NRD LINVTSAANV TLDLFGEWR**K SYLLPRK**IHD NSQLLTAIDL NGLTIGMAYV STMCQ**SKYSV**
321 **GVVQDHSK**IN LRVAVTMAHE IGHNLGLTHD GVYCTCGGYS CIMSAVLGDQ PSKYFSNCSY NQYRRFLTEH NPECIINPPL
401 RTDIVSPPAC GNELLERGEE CDCGSPENCR DPCCDAASCK LHSWVECESG KCCNQCRFKR AGTECRPARD ECDKAEQCTG
481 RSANCPVDEF HENGRPCLHN FGYCYNGKCP IMYHQCHALF GQNVTGVQDS CFQYNRLGVY YAYCRKENGR KIPCAPKDEK
561 CGRLYCSYKS PGNQIPCLPY YIPSDENKGM VDHGTKCGDG KVCSNGQCVD LNIAY

Supporting Peptides:

Peptide	Uniq	-10lgP	Mass	Length	ppm	m/z	z	RT	Fraction	Scan	Source File	Area F1	Area F2	Area F3
K.YKNDLTAIR.T	N	69.36	1092.5928	9	0.5	547.3040	2	30.48	1	F1:8695	12112021_RID_2349_DP1.raw	1.0161E6	3.056E5	9.9469E5
K.ASLLVATSER.N	Y	65.12	1045.5768	10	-0.4	523.7955	2	35.52	1	F1:10437	12112021_RID_2349_DP1.raw	8.3446E5		
R.VPLVGLEIWK.N	Y	64.06	1152.6907	10	-0.3	577.3524	2	61.59	1	F1:19008	12112021_RID_2349_DP1.raw	1.8214E6		
R.KSYLLPR.K	Y	52.02	875.5228	7	0.7	438.7690	2	31.78	1	F1:9234	12112021_RID_2349_DP1.raw	7.7747E6		
A.SLLVATSER.N	Y	50.58	974.5396	9	-0.5	488.2769	2	33.39	1	F1:9720	12112021_RID_2349_DP1.raw	6.4327E5		
V.TQTNWESDEPIKK.A	N	44.98	1574.7576	13	0.5	788.3865	2	30.75	1	F1:8710	12112021_RID_2349_DP1.raw	3.3503E5		7.2185E4
K.NDLTAIR.T	N	44.81	801.4344	7	0.3	401.7246	2	29.16	7	F7:7605	17112021_RID_2349_DP7.raw	1.0951E5	4.7012E4	
Q.SKYSVGVVQDHSK.I	Y	43.60	1432.7310	13	-0.6	478.5840	3	27.74	1	F1:7696	12112021_RID_2349_DP1.raw	1.9427E5		
K.SYLLPRK.I	Y	42.55	875.5228	7	0.2	438.7688	2	28.14	1	F1:7822	12112021_RID_2349_DP1.raw	1.2424E6		
K.YSVGVVQDHSK.I	Y	42.32	1217.6040	11	0.5	406.8755	3	26.34	1	F1:7230	12112021_RID_2349_DP1.raw	2.3403E5	1.1059E5	

total 10 peptides

AAZ39880.1
back to list

| Protein Coverage | Supporting Peptides |
Protein Coverage:

1 MMQVLLVTIC LAVFPYHGSS IILESGNVND YEVVYPQKVT AMPKEAVKQP EQKYEDAMQY KFEVNGEPVL LHLEKNKDLF
81 SEDYSETHYS PDGREITTKP LVQDHCYYHG HIQNDAHSSA SISACNGLKG HFKLRGEMYL IEPLKLSDSE AHAVYKYENV
161 EKEDEALKMC GV**TQTNWESD EPIKKASLLV ATSER**NRYFN PYSYVELIIT VDHSMVTK**YK NDLTAIR**TWV FELVNTINEI
241 FKYLYIR**VPL VGLEIWK**NRD LINVTSAANV TLDLFGEWR**K SYLLPRK**IHD NSQLLTAIDL NGLTIGMAYV STMCQ**SKYSV**
321 **GVVQDHSK**IN LRVAVTMAHE IGHNLGLTHD GVYCTCGGYS CIMSAVLGDQ PSKYFSNCSY NQYRRFLTEH NPECIINPPL
401 RTDIVSPPAC GNELLERGEE CDCGSPENCR DPCCDAASCK LHSWVECESG KCCNQCRFKR AGTECRPARD ECDKAEQCTG
481 RSANCPVDEF HENGRPCLHN FGYCYNGKCP IMYHQCHALF GQNVTGVQDS CFQYNRLGVY YAYCRKENGR KIPCAPKDEK
561 CGRLYCSYKS PGNQIPCLPY YIPSDENKGM VDHGTKCGDG KVCSNGQCVD LNIAY

Supporting Peptides:

Peptide	Uniq	-10lgP	Mass	Length	ppm	m/z	z	RT	Fraction	Scan	Source File	Area F1	Area F2	Area F3
K.YKNDLTAIR.T	N	69.36	1092.5928	9	0.5	547.3040	2	30.48	1	F1:8695	12112021_RID_2349_DP1.raw	1.0161E6	3.056E5	9.9469E5

total 10 peptides

Peptide	Uniq	-10lgP	Mass	Length	ppm	m/z	z	RT	Fraction	Scan	Source File	Area F1	Area F2	Area F3
K.ASLLVATSER.N	Y	65.12	1045.5768	10	-0.4	523.7955	2	35.52	1	F1:10437	12112021_RID_2349_DP1.raw	8.3446E5		
R.VPLVGLEIWK.N	Y	64.06	1152.6907	10	-0.3	577.3524	2	61.59	1	F1:19008	12112021_RID_2349_DP1.raw	1.8214E6		
R.KSYLLPR.K	Y	52.02	875.5228	7	0.7	438.7690	2	31.78	1	F1:9234	12112021_RID_2349_DP1.raw	7.7747E6		
A.SLLVATSER.N	Y	50.58	974.5396	9	-0.5	488.2769	2	33.39	1	F1:9720	12112021_RID_2349_DP1.raw	6.4327E5		
V.TQTNWESDEPIKK.A	N	44.98	1574.7576	13	0.5	788.3865	2	30.75	1	F1:8710	12112021_RID_2349_DP1.raw	3.3503E5		7.2185E4
K.NDLTAIR.T	N	44.81	801.4344	7	0.3	401.7246	2	29.16	7	F7:7605	17112021_RID_2349_DP7.raw	1.0951E5	4.7012E4	
Q.SKYSVGVVQDHSK.I	Y	43.60	1432.7310	13	-0.6	478.5840	3	27.74	1	F1:7696	12112021_RID_2349_DP1.raw	1.9427E5		
K.SYLLPRK.I	Y	42.55	875.5228	7	0.2	438.7688	2	28.14	1	F1:7822	12112021_RID_2349_DP1.raw	1.2424E6		
K.YSVGVVQDHSK.I	Y	42.32	1217.6040	11	0.5	406.8755	3	26.34	1	F1:7230	12112021_RID_2349_DP1.raw	2.3403E5	1.1059E5	

total 10 peptides

ACE73567.1
back to list

| Protein Coverage | Supporting Peptides |
Protein Coverage:

1 MIAFIVLPIL AAVLQQSSGS VDFDSESPRR PEIQNEIVDL HNSLRRSVTP TASNMLKMEW YPEAAANAER WAFRCILNHS C

81 PYNSRVIGGI KCGENIYMSP YPMKWTAIIH EWHKEKKDFV YGQGASPANA VVGHYTQIVW YKSYRSGCAA AYCPSSEYNY

161 FYVCQYCPAG NIIGKIATPY TSGPPCGDCP SACDNGLCTN PCSHHDEFTN CKDLVKQGCH SNYLKTKCPA SCFCHNEII

Supporting Peptides:

Peptide	Uniq	-10lgP	Mass	Length	ppm	m/z	z	RT	Fraction	Scan	Source File	Area F1	Area F2	Ar F.
K.MEWYPEAAANAER.W	N	85.56	1536.6667	13	0.5	769.3411	2	45.12	7	F7:13118	17112021_RID_2349_DP7.raw			
K.M(+15.99)EWYPEAAANAER.W	N	83.36	1552.6616	13	0.4	777.3384	2	40.79	7	F7:11501	17112021_RID_2349_DP7.raw			
G.SVDFDSESPR.R	N	67.58	1137.4938	10	-0.1	569.7541	2	30.74	7	F7:8132	17112021_RID_2349_DP7.raw	4.0226E4		
R.SVTPTASNMLK.M	Y	66.97	1147.5907	11	0.0	574.8026	2	33.55	7	F7:9166	17112021_RID_2349_DP7.raw			
R.RSVTPTASNMLK.M	Y	56.72	1303.6918	12	0.4	652.8535	2	28.75	7	F7:7373	17112021_RID_2349_DP7.raw			
R.SVTPTASNM(+15.99)LK.M	Y	41.99	1163.5856	11	0.4	582.8003	2	24.98	4	F4:6074	17112021_RID_2349_DP4.raw			

total 6 |

TRINITY_DN324
back to list

| Protein Coverage | Supporting Peptides |
Protein Coverage:

1 MAEAAAAETA ALFGLLLVLL LAPAALAAPG WAVPVDVRGD SVREAARFAL AEAFPEGHPR GLWALCGART QVVNGIKHYL

81 EVVLLQSPCR RGEMADCDSP SGSLPLGKPE HVSCHFEVWS QPWASSKNLL KQDCHPTEPL TEIQAASLGP WETFPLNSPF

161 VDVAEASLRQ EESLQNVSAH LVSLFKDFLI TYRKSYLDDR ETQRRFQIFA ENLERARLYQ ELDQGTAEYG VTKFSDLTEE

241 EFHSTYLNPL LAKLPGRPMK EASIPNGSFP EEWDWRDHGA VTEVKNQGEC GSCWAFSVTG NIEGQWFLHK GTLLSLSEQE

321 LVDCDVIDKA CGGGLPSNAY EAIEKLGGLE TERDYSYQGF EQNCGFSREK VAVYINSSVG VSRDESEIAA WLAKNGPISI

401 ALNAFAMQFY RKGVSHPFFM LCNPWMIDHA VLLVGYGTRG QIPFWAIKNS WGEDWGEKGY YYLYRGKHAC GMNTMCSSAV

481 IN

Supporting Peptides:

Peptide	Uniq	-10lgP	Mass	Length	ppm	m/z	z	RT	Fraction	Scan	Source File	Area F1	Area F2	Arc
R.LYQELDQGTAEYGVTK.F	Y	91.90	1813.8734	16	0.9	907.9448	2	42.13	2	F2:11774	12112021_RID_2349_DP2.raw		6.0013E5	
R.DESEIAAWLAK.N	Y	64.98	1231.6084	11	0.0	616.8115	2	58.67	2	F2:17147	12112021_RID_2349_DP2.raw		5.887E4	3.37
R.GQIPFWAIK.N	Y	50.17	1058.5913	9	0.3	530.3031	2	54.72	3	F3:16090	17112021_RID_2349_DP3.raw			8.69
K.LGGLETER.D	Y	50.13	873.4556	8	-0.2	437.7350	2	26.62	2	F2:6331	12112021_RID_2349_DP2.raw		2.0992E5	6.12
R.FQIFAENLER.A	Y	44.23	1265.6404	10	0.2	633.8276	2	51.63	2	F2:14817	12112021_RID_2349_DP2.raw		2.5367E5	

total 6 peptides

Peptide	Uniq	-10lgP	Mass	Length	ppm	m/z	z	RT	Fraction	Scan	Source File	Area F1	Area F2	Arc
K.FSDLTEEEFHSTYLNPLLAK.L	Y	41.38	2353.1477	20	0.6	785.3903	3	58.68	2	F2:17169	12112021_RID_2349_DP2.raw		3.9261E5	

total 6 peptides

TRINITY_DN441
back to list

| Protein Coverage | Supporting Peptides |
Protein Coverage:

```
  1 TGILQGNILP RRVEAMVLIR VLANLLVLQL SYAQKSSELV VGGHRCNINE HRFLALVNSD KKQCGGTLIN QEWVLTAAHC   o C

                                                    126
 81 DGKIMKIQLG VHSKKVPNKD KQTRVPKQKF FCLSSKNYTM WDKDIMLIKL NGPVKTSTHI APLSLPSS
```

Supporting Peptides:

Peptide	Uniq	-10lgP	Mass	Length	ppm	m/z	z	RT	Fraction	Scan	Source File	Area F1	Area F2	Area I
K.TSTHIAPLSLPSS	N	60.21	1309.6877	13	-0.1	655.8511	2	44.76	4	F4:12858	17112021_RID_2349_DP4.raw			
R.FLALVNSDKK.Q	Y	57.18	1133.6444	10	0.1	567.8295	2	34.21	2	F2:9023	12112021_RID_2349_DP2.raw		8.9311E6	4.923E
M.WDKDIMLIK.L	N	57.12	1160.6263	9	-0.1	581.3204	2	46.64	2	F2:13338	12112021_RID_2349_DP2.raw	2.1169E5	9.915E6	1.5104
K.SSELVVGGHR.C	Y	56.70	1039.5410	10	-0.1	520.7777	2	21.39	3	F3:4900	17112021_RID_2349_DP3.raw			2.3028
R.FLALVNSDK.K	Y	53.75	1005.5494	9	-0.6	503.7817	2	41.29	5	F5:11774	17112021_RID_2349_DP5.raw		3.9766E6	2.0733
M.WDKDIM(+15.99)LIK.L	N	52.20	1176.6212	9	-0.2	589.3177	2	41.27	2	F2:11445	12112021_RID_2349_DP2.raw		6.6323E6	2.1157
K.IQLGVHSKK.V	N	42.78	1008.6080	9	0.2	505.3113	2	18.69	3	F3:4026	17112021_RID_2349_DP3.raw			5.2609
K.IQLGVHSK.K	N	40.36	880.5131	8	-0.5	441.2636	2	23.29	1	F1:6181	12112021_RID_2349_DP1.raw	3.6053E6	1.5875E7	1.7023
R.FLALVNSDKKQ.C	Y	39.83	1261.7030	11	-0.2	631.8586	2	33.21	3	F3:8944	17112021_RID_2349_DP3.raw			7.979E
F.LALVNSDKK.Q	Y	38.72	986.5760	9	-0.8	494.2949	2	33.30	3	F3:8929	17112021_RID_2349_DP3.raw			2.4679

total 10

AUF41660.1
back to list

| Protein Coverage | Supporting Peptides |
Protein Coverage:

```
  1 MMQVLLVTIS LAVFPYQGSS IILESGNVND YEVVYPQKVT ALPKGAVQQP EQKYEDTMQY EFEVNGEPVV LHLEKNKILF
 81 SEDYSETHYS PDGREITTNP PVEDHCYYHG RIQNDAHSSA SISACNGLKG HFKLRGEMYF IEPLKLSNNE AHAVYKYENI
161 EKEDETPKMC GVTQTNWESD EPIKKASLLN LTPEQRTYLK SKKYIEVIII ADYIIYLKYG RNLFTIRTRI YEIVNILNVI

241 YRVLNIYIAL LGLEIWNNGD KINVLPEAKV TLDLFGKWKA SDLLNHRNHD NAYLLTGINF NGPTAGLGYL GGMCHPEYSA

321 GIVQDHNKLN YLVALAMAHE MGHNLGMEHD KIHCTCGAKS CIMSGTLSCE ASNRFSDCSR EEHQKYLIDK MPQCILNKPL
401 KTDIVSPAVC GNYLVELGED CDCGSPSVCQ NPCCNAATCK LTQGSQCADE ECCDQCKFRR AGTVCRAANG ECDVSDLCTG
481 QSAECPTDQF QRNGHPCQNN NGYCYNGKCP IMGKQCISLF GSRATVAEDA CFNFNSLGNA YGYCRKENGR KIPCAPQDVK
561 CGRLYCFDNL PEHKNPCQIY YTPSDENKGM VDLGTKCGDG KACSSNRQCV DVNTAYQST
```

Supporting Peptides:

Peptide	Uniq	-10lgP	Mass	Length	ppm	m/z	z	RT	Fraction	Scan	Source File	Area F1	Area F2	Area F3
R.IYEIVNILNVIYR.V	N	76.22	1620.9238	13	0.7	811.4697	2	75.23	4	F4:22905	17112021_RID_2349_DP4.raw			1.5149E6
K.VTLDLFGK.W	N	54.13	891.5065	8	-0.2	446.7604	2	51.79	1	F1:15754	12112021_RID_2349_DP1.raw	8.9865E6	9.162E6	4.6551E7
K.ASLLNLTPEQR.T	Y	49.74	1240.6775	11	-0.1	621.3459	2	44.70	7	F7:12940	17112021_RID_2349_DP7.raw			
K.INVLPEAK.V	N	47.44	882.5175	8	0.8	442.2664	2	35.58	1	F1:10479	12112021_RID_2349_DP1.raw	8.9247E6	1.1229E7	4.4088E7
V.TQTNWESDEPIKK.A	N	44.98	1574.7576	13	0.5	788.3865	2	30.75	1	F1:8710	12112021_RID_2349_DP1.raw	3.3503E5		7.2185E4
K.GMVDLGTK.C	N	44.20	819.4160	8	0.0	410.7153	2	30.74	7	F7:8124	17112021_RID_2349_DP7.raw			
L.LNLTPEQR.T	N	41.26	969.5243	8	0.0	485.7694	2	30.89	7	F7:8269	17112021_RID_2349_DP7.raw	4.4645E5		

total 7 pept

CAR40186.1

back to list

| Protein Coverage | Supporting Peptides |

Protein Coverage:

1 MRTLWIVAVC LMGVEGHLTQ FGDMINKKTG TFGLLSYVYY GCYCGLGGKG KPQDATDRCC FVHDCCYGTV NGCDPKLSTY

81 SYSFQNGDIV CGDDDPCLRA VCECDRVAAI CFGENMNTYD KKYMLYSLFD CMEESEKC

Supporting Peptides:

Peptide	Uniq	-10lgP	Mass	Length	ppm	m/z	z	RT	Fraction	Scan	Source File	Area F1	Area F2	A
G.HLTQFGDMINKK.T	Y	78.52	1430.7340	12	0.0	477.9186	3	34.29	7	F7:9506	17112021_RID_2349_DP7.raw	2.9174E6	3.5177E6	2.[illegible]
G.HLTQFGDMINK.K	Y	74.36	1302.6390	11	-0.1	652.3267	2	37.85	7	F7:10612	17112021_RID_2349_DP7.raw	1.8546E6	6.6203E5	4.[illegible]
G.HLTQFGDM(+15.99)INK.K	Y	69.44	1318.6339	11	-0.1	660.3242	2	37.85	7	F7:10613	17112021_RID_2349_DP7.raw	4.5507E5	3.4443E5	1.[illegible]
G.HLTQFGDM(+15.99)INKK.T	Y	68.29	1446.7289	12	-0.4	483.2501	3	26.61	7	F7:6724	17112021_RID_2349_DP7.raw	2.9583E6	2.0754E6	8.[illegible]
G.ENMNTYDK.K	N	52.21	1013.4124	8	0.7	507.7138	2	19.60	7	F7:4282	17112021_RID_2349_DP7.raw			
K.GKPQDATDR.C	N	48.61	986.4781	9	-0.5	494.2461	2	11.50	7	F7:1839	17112021_RID_2349_DP7.raw			
G.ENMNTYDKK.Y	N	43.49	1141.5073	9	-0.1	571.7609	2	15.11	7	F7:2868	17112021_RID_2349_DP7.raw			
K.KYMLYSLFD.C	N	43.04	1178.5681	9	0.2	590.2914	2	60.72	7	F7:18383	17112021_RID_2349_DP7.raw			

tot

B5U6Z2.1

back to list

| Protein Coverage | Supporting Peptides |

Protein Coverage:

1 MRTLWIVAVC LMGVEGHLTQ FGDMINKKTG TFGLLSYVYY GCYCGLGGKG KPQDATDRCC FVHDCCYGTV NGCDPKLSTY

81 SYSFQNGDIV CGDDDPCLRA VCECDRVAAI CFGENMNTYD KKYMLYSLFD CMEESEKC

Supporting Peptides:

Peptide	Uniq	-10lgP	Mass	Length	ppm	m/z	z	RT	Fraction	Scan	Source File	Area F1	Area F2	A
G.HLTQFGDMINKK.T	Y	78.52	1430.7340	12	0.0	477.9186	3	34.29	7	F7:9506	17112021_RID_2349_DP7.raw	2.9174E6	3.5177E6	2.[illegible]
G.HLTQFGDMINK.K	Y	74.36	1302.6390	11	-0.1	652.3267	2	37.85	7	F7:10612	17112021_RID_2349_DP7.raw	1.8546E6	6.6203E5	4.[illegible]
G.HLTQFGDM(+15.99)INK.K	Y	69.44	1318.6339	11	-0.1	660.3242	2	37.85	7	F7:10613	17112021_RID_2349_DP7.raw	4.5507E5	3.4443E5	1.[illegible]
G.HLTQFGDM(+15.99)INKK.T	Y	68.29	1446.7289	12	-0.4	483.2501	3	26.61	7	F7:6724	17112021_RID_2349_DP7.raw	2.9583E6	2.0754E6	8.[illegible]
G.ENMNTYDK.K	N	52.21	1013.4124	8	0.7	507.7138	2	19.60	7	F7:4282	17112021_RID_2349_DP7.raw			
K.GKPQDATDR.C	N	48.61	986.4781	9	-0.5	494.2461	2	11.50	7	F7:1839	17112021_RID_2349_DP7.raw			
G.ENMNTYDKK.Y	N	43.49	1141.5073	9	-0.1	571.7609	2	15.11	7	F7:2868	17112021_RID_2349_DP7.raw			
K.KYMLYSLFD.C	N	43.04	1178.5681	9	0.2	590.2914	2	60.72	7	F7:18383	17112021_RID_2349_DP7.raw			

tot

TRINITY_DN59

back to list

| Protein Coverage | Supporting Peptides |

Protein Coverage:

1 MGRFISVSFG WLVVFLSLSG TGADQDCLPG WSFYEGNCYK AFDEPKSWVD AEKFCQKQSN GKHLATIEWL GKANFVAELV

81 TLREPETHVW IGLRVEDKRQ QCSSHWTDGS AVSYETVTKY TKCFGLNKDK GYLEWVTLPC EDNIPFICKS WVPH

Supporting Peptides:

Peptide	Uniq	-10lgP	Mass	Length	ppm	m/z	z	RT	Fraction	Scan	Source File	Area F1	Area F2	Area F3	Area F4

total 5 peptides

Peptide	Uniq	-10lgP	Mass	Length	ppm	m/z	z	RT	Fraction	Scan	Source File	Area F1	Area F2	Area F3	Area F4
K.HLATIEWLGK.A	N	75.80	1166.6448	10	0.6	584.3300	2	45.31	7	F7:13096	17112021_RID_2349_DP7.raw				
K.ANFVAELVTLR.E	Y	67.48	1231.6924	11	0.3	616.8536	2	60.68	6	F6:18179	17112021_RID_2349_DP6.raw				9.8423E4
D.GSAVSYETVTK.Y	N	57.05	1140.5663	11	0.1	571.2905	2	28.66	6	F6:7260	17112021_RID_2349_DP6.raw				
H.LATIEWLGK.A	N	49.74	1029.5858	9	-0.3	515.8000	2	50.89	7	F7:15056	17112021_RID_2349_DP7.raw				
H.WTDGSAVSYETVTK.Y	N	45.93	1542.7202	14	0.5	772.3678	2	39.78	6	F6:11115	17112021_RID_2349_DP6.raw				

total 5 peptides

TRINITY_DN278
back to list

| Protein Coverage | Supporting Peptides |
Protein Coverage:

1 SGWSAYDQNC YK**VFKEEMNW ADAEK**FCTEQ HK**GSHLVSLH N**IAEADFVVK **KTLAMLK**DGV IWMGLNDVWN ECNWGWTDGA

81 K**LDYKAWNEG TN**CFVFKIAK NHWSHMDCSR THNFVCKFRV

Supporting Peptides:

Peptide	Uniq	-10lgP	Mass	Length	ppm	m/z	z	RT	Fraction	Scan	Source File	Area F1	Area F2	Area F3	A
K.VFKEEMNWADAEK.F	Y	78.10	1595.7290	13	0.4	798.8721	2	40.09	7	F7:11338	17112021_RID_2349_DP7.raw				1.[illegible]
K.EEMNWADAEK.F	N	64.38	1221.4972	10	0.6	611.7562	2	36.04	7	F7:10022	17112021_RID_2349_DP7.raw				
K.VFKEEM(+15.99)NWADAEK.F	Y	62.41	1611.7239	13	0.5	806.8696	2	33.88	7	F7:9194	17112021_RID_2349_DP7.raw				
K.LDYKAWNEGTN.C	N	59.40	1309.5939	11	-0.2	655.8041	2	39.53	7	F7:11171	17112021_RID_2349_DP7.raw				
K.KTLAMLK.D	N	50.24	803.4939	7	0.4	402.7544	2	24.60	7	F7:5931	17112021_RID_2349_DP7.raw				
K.EEM(+15.99)NWADAEK.F	N	48.33	1237.4921	10	0.6	619.7537	2	28.66	7	F7:7336	17112021_RID_2349_DP7.raw				
K.GSHLVSLHN.I	N	46.52	962.4933	9	-0.1	482.2539	2	23.67	7	F7:5711	17112021_RID_2349_DP7.raw				
K.GSHLVSLH.N	N	45.55	848.4504	8	0.0	425.2325	2	23.74	7	F7:5731	17112021_RID_2349_DP7.raw				

total 8 pepti

AJO70723.1
back to list

| Protein Coverage | Supporting Peptides |
Protein Coverage:

1 MGRFISVSFG WLVVFLSLSG TEAAFCCPSG WSAYDQNCYK **VFKEEMNWAD AEK**FCTEQHK **GSHLVSLHN**I AEADFVVK**KT**

81 **LAMLK**DGVIW MGLNDVWNEC NWGWTDGAK**L DYKAWNEGTN** CFVFKIAKNH WSRMDCSKTH NFVCKFRV

Supporting Peptides:

Peptide	Uniq	-10lgP	Mass	Length	ppm	m/z	z	RT	Fraction	Scan	Source File	Area F1	Area F2	Area F3	A
K.VFKEEMNWADAEK.F	Y	78.10	1595.7290	13	0.4	798.8721	2	40.09	7	F7:11338	17112021_RID_2349_DP7.raw				1.[illegible]
K.EEMNWADAEK.F	N	64.38	1221.4972	10	0.6	611.7562	2	36.04	7	F7:10022	17112021_RID_2349_DP7.raw				
K.VFKEEM(+15.99)NWADAEK.F	Y	62.41	1611.7239	13	0.5	806.8696	2	33.88	7	F7:9194	17112021_RID_2349_DP7.raw				
K.LDYKAWNEGTN.C	N	59.40	1309.5939	11	-0.2	655.8041	2	39.53	7	F7:11171	17112021_RID_2349_DP7.raw				
K.KTLAMLK.D	N	50.24	803.4939	7	0.4	402.7544	2	24.60	7	F7:5931	17112021_RID_2349_DP7.raw				
K.EEM(+15.99)NWADAEK.F	N	48.33	1237.4921	10	0.6	619.7537	2	28.66	7	F7:7336	17112021_RID_2349_DP7.raw				
K.GSHLVSLHN.I	N	46.52	962.4933	9	-0.1	482.2539	2	23.67	7	F7:5711	17112021_RID_2349_DP7.raw				
K.GSHLVSLH.N	N	45.55	848.4504	8	0.0	425.2325	2	23.74	7	F7:5731	17112021_RID_2349_DP7.raw				

total 8 pepti

XP_034272439.1
back to list

| Protein Coverage | Supporting Peptides |

Protein Coverage:

```
1   MNTYSFRQSS SNIGGGGFGG SSSFRTPSIH GGSGGYGISV SSARLASSGF GGSLGGGYGG GYGGGSFNFG SGCFGGAGGA
81  GGNINASDGL LSGNEKVTMQ NLNDRLANYL DKVRALEEAN TELEVKIREW YQKQGPTATR DYSPYYKTIE DLRDKILVAT
161 IENNKMILQI DNARLAADDF KTKFETEQAL RMSVEADING LRRVLDELTL ARTDLELQIE SLKEELAYLK KNHEEEMSAL
241 SGQLGGQVSV EVDSAPGIDL TKILADMRDQ YELLADKNRR DAEAWFTSKT EELTKEVAIN TEQLQTSKTE ITDLRRTLQG
321 LEIELQSQLS MKSALEGTLG DTEARYGAQL CQIQSLISNL EAQLVDVRSD MERQNNDYKM LMDIKSRLEQ EIETYRQLLD
401 GQDFNFSGNA SGMYGKTKQL LATSLEDYPL RYAQIFNTQI SMAGIIEWTQ LELDNLDQKT RKLMTMHCAL HPKSDIDQLY
481 LPRSQDGKGL LQVKQTVEEE KHSLNDYIQK EKEPLLKETK PSNSLASELS FTMSHYYQSS SSSYGGGGGG GSGRVASVRH
561 GPSSIHGGAG GRVSASSARF VSSGSGYGGG YGYGSGLGGG CAIGYGSVGG LGYGNYFGSS GGYGGGVGGG YGCGVGGGYG
641 GGVGGGYGGG LDFVVGGGGG GDGGLLTGNE KITMQNLNDR LANYLDKVRA LEEANTELEI KIRDWHQKQA PSSPAKDYSH
721 YYKIISDLRD KIHDATIDNS RTILEIDNAR LAADDFRLKY ENELHLRQGV ESDINGLRRV LDELTLFKSD MEMQIEEKKE
801 DLAYLKKNHE ELMKEFSTQL TGKVSVEMDA APGVDLTKIL ADMREQYEQM AERNRKDAEA WFFTKTEELN REVATHTEQL
881 QTSKTEISEL RRTIQSLEIE LQSQLSMKAG LETSLAETEG RYCAQLAQIQ NLITNVEEQL AELRNDMERQ NHEYRMLMDI
961 KTRLEQEIAT YRNLLEGQDS KLPGWSPKDA TYGGSSTINY SSGGSGSGGG GAISGSGYSS GGSSVTTSKV RSGNF
```

Supporting Peptides:

Peptide	Uniq	-10lgP	Mass	Length	ppm	m/z	z	RT	Fraction	Scan	Source File	Area F1	Area F2	Area
K.VTMQNLNDR.L	N	68.95	1089.5237	9	0.0	545.7691	2	27.78	6	F6:7010	17112021_RID_2349_DP6.raw	1.3099E6	6.7229E5	5.351[illegible]
R.ALEEANTELEVK.I	N	67.86	1344.6772	12	0.3	673.3461	2	36.35	6	F6:9844	17112021_RID_2349_DP6.raw			
K.VTM(+15.99)QNLNDR.L	N	67.75	1105.5186	9	-0.5	553.7663	2	28.09	1	F1:7841	12112021_RID_2349_DP1.raw	7.8553E5	3.4355E5	1.125[illegible]
R.VLDELTLAR.T	Y	63.40	1028.5865	9	1.0	515.3011	2	44.76	7	F7:12937	17112021_RID_2349_DP7.raw	5.1514E5	3.8767E5	3.897[illegible]
K.TRLEQEIATYR.N	N	60.70	1378.7205	11	-0.1	690.3674	2	36.59	4	F4:9988	17112021_RID_2349_DP4.raw		4.6159E4	
R.LEQEIATYR.N	N	59.72	1121.5717	9	-0.1	561.7930	2	32.42	6	F6:8521	17112021_RID_2349_DP6.raw	2.3192E4		
R.LAADDFRLK.Y	N	56.97	1047.5713	9	0.6	524.7932	2	38.21	1	F1:11342	12112021_RID_2349_DP1.raw	4.5823E5		
R.LAADDFR.L	N	55.06	806.3922	7	0.0	404.2034	2	31.43	2	F2:8068	12112021_RID_2349_DP2.raw	2.1578E6	1.4275E6	3.776[illegible]
														total

QBF53416.1

back to list

| Protein Coverage | Supporting Peptides |

Protein Coverage:

```
1   MIQVLLVIIC LAVFPYQVSS IILESGNINN YEVVYPQKVT ALPKGAIQQL EQKYEDAMQY QFKVKGEPVV LHLEKNKDFF
81  PEDYSETHYS PDDREITTNP PVEDHCYYYG HIQNDADSTA SISACNGLKG YFTLRGVTYL IEPLKIPESE AHAIYKYENV
161 EKEDEDPKKC EFRRAGTECR PARSECDVAE YCTGQSAECP TDVFHSNGKP CLNNFGYCYN GNCPIMYHQC YALFGPNATV
241 GQDGCFEWNK KGESYFYCRK ENDVPIPCAP EDIKCGRLFC ELIKNTCKYD YSEDPDYGMV DHGTKCGDGK VCINRHCVDV
321 TTAY
```

Supporting Peptides:

Peptide	Uniq	-10lgP	Mass	Length	ppm	m/z	z	RT	Fraction	Scan	Source File	Area F1	A[illegible]
K.YDYSEDPDYGMVDHGTK.C	N	86.45	1990.7891	17	0.5	996.4023	2	37.41	4	F4:10370	17112021_RID_2349_DP4.raw		
E.DPDYGM(+15.99)VDHGTK.C	N	72.65	1349.5558	12	0.3	675.7854	2	22.45	4	F4:5264	17112021_RID_2349_DP4.raw		1.1[illegible]
E.DPDYGMVDHGTK.C	N	64.82	1333.5608	12	0.6	667.7881	2	28.50	3	F3:7288	17112021_RID_2349_DP3.raw	1.1371E6	
K.YDYSEDPDYGM(+15.99)VDHGTK.C	N	61.79	2006.7839	17	0.2	669.9354	3	32.76	4	F4:8703	17112021_RID_2349_DP4.raw		
D.PDYGMVDHGTK.C	N	55.63	1218.5339	11	0.9	407.1856	3	24.91	5	F5:6050	17112021_RID_2349_DP5.raw		

Peptide	Uniq	-10lgP	Mass	Length	ppm	m/z	z	RT	Fraction	Scan	Source File	Area F1	Ar
K.YENVEKEDEDPKK.C	Y	51.92	1621.7471	13	-0.1	541.5896	3	17.62	4	F4:3552	17112021_RID_2349_DP4.raw		

TRINITY_DN59
back to list

| Protein Coverage | Supporting Peptides |
Protein Coverage:

1 ADCPSDWCSH EGHCYKVFRL FKTWEEAEKF CMQQVNGWHL ASIESVEEAN FVAELVSKTL IKSKYHAWIG LRDQSERQQC

81 SSHWTDGSAV SYETVTKYTK CFGLNKDKGY LEWVTLPCED NIPFICKSWV PH

Supporting Peptides:

Peptide	Uniq	-10lgP	Mass	Length	ppm	m/z	z	RT	Fraction	Scan	Source File	Area F1	Area F2	Area F3	Area F4	Ar F
E.SVEEANFVAELVSK.T	Y	64.69	1520.7722	14	0.7	761.3939	2	57.97	6	F6:17304	17112021_RID_2349_DP6.raw					
K.YHAWIGLR.D	N	61.14	1014.5399	8	-0.1	508.2772	2	40.32	7	F7:11939	17112021_RID_2349_DP7.raw					3.38
D.GSAVSYETVTK.Y	N	57.05	1140.5663	11	0.1	571.2905	2	28.66	6	F6:7260	17112021_RID_2349_DP6.raw					
R.LFKTWEEAEK.F	N	55.19	1279.6448	10	0.9	640.8303	2	35.62	7	F7:9816	17112021_RID_2349_DP7.raw					
K.SKYHAWIGLR.D	N	51.39	1229.6669	10	0.3	615.8409	2	41.34	7	F7:11753	17112021_RID_2349_DP7.raw					
K.TWEEAEKF.C	N	47.94	1038.4658	8	0.3	520.2404	2	39.87	6	F6:11136	17112021_RID_2349_DP6.raw					
Y.HAWIGLR.D	N	46.00	851.4766	7	0.5	426.7458	2	35.11	7	F7:9748	17112021_RID_2349_DP7.raw					
H.WTDGSAVSYETVTK.Y	N	45.93	1542.7202	14	0.5	772.3678	2	39.78	6	F6:11115	17112021_RID_2349_DP6.raw					
F.KTWEEAEK.F	N	45.16	1019.4923	8	0.3	510.7536	2	17.57	6	F6:3741	17112021_RID_2349_DP6.raw					

total 9 peptides

TRINITY_DN59
back to list

| Protein Coverage | Supporting Peptides |
Protein Coverage:

1 ADCPSDWCSH EGHCYKVFRL FKTWEEAEKF CMQQVNGWHL ASIESVEEAN FVAELVSKTL IKSKYHAWIG LRDQSERQQC

81 SSHWTDGSAV SYETVTKYTK CFGLNKDKGY LEWVTLPCED NIPFICKSWV PH

Supporting Peptides:

Peptide	Uniq	-10lgP	Mass	Length	ppm	m/z	z	RT	Fraction	Scan	Source File	Area F1	Area F2	Area F3	Area F4	Ar F
E.SVEEANFVAELVSK.T	Y	64.69	1520.7722	14	0.7	761.3939	2	57.97	6	F6:17304	17112021_RID_2349_DP6.raw					
K.YHAWIGLR.D	N	61.14	1014.5399	8	-0.1	508.2772	2	40.32	7	F7:11939	17112021_RID_2349_DP7.raw					3.38
D.GSAVSYETVTK.Y	N	57.05	1140.5663	11	0.1	571.2905	2	28.66	6	F6:7260	17112021_RID_2349_DP6.raw					
R.LFKTWEEAEK.F	N	55.19	1279.6448	10	0.9	640.8303	2	35.62	7	F7:9816	17112021_RID_2349_DP7.raw					
K.SKYHAWIGLR.D	N	51.39	1229.6669	10	0.3	615.8409	2	41.34	7	F7:11753	17112021_RID_2349_DP7.raw					
K.TWEEAEKF.C	N	47.94	1038.4658	8	0.3	520.2404	2	39.87	6	F6:11136	17112021_RID_2349_DP6.raw					
Y.HAWIGLR.D	N	46.00	851.4766	7	0.5	426.7458	2	35.11	7	F7:9748	17112021_RID_2349_DP7.raw					
H.WTDGSAVSYETVTK.Y	N	45.93	1542.7202	14	0.5	772.3678	2	39.78	6	F6:11115	17112021_RID_2349_DP6.raw					
F.KTWEEAEK.F	N	45.16	1019.4923	8	0.3	510.7536	2	17.57	6	F6:3741	17112021_RID_2349_DP6.raw					

total 9 peptides

TRINITY_DN283
back to list

| Protein Coverage | Supporting Peptides |
Protein Coverage:

1 MLKKSKMFLL LSILSFLCAT SCSFARDESN LFPWDKIRLP KYVVPVHYDL LIHPNLTTLS FTGLSKIEIL ITEQTSSIIL
81 HSKYLQITKA TIHNTKDSAD TAKGMMVLEH PPFEQIALVS TEPLQDGQSY IISIEYSANL SESFHGFYKS TYRTLEGETR
161 VLASTQFEAT AARMAFPCFD EPALKATFSI KIRRSPKHLA LSNMPLVKSV NINEWLIEDQ FDISVKMSTY LVAFIISDFK
241 SVTKRTLRGI KVSVYTVPHK IKQAEYALDA AVKLLDFFEG YFGIAYPLPK QDLAAIPDFQ SGAMENWGLT TYRESALLYD
321 SEKSSVSKKL VITMTVAHEL AHQWFGNLVT MEWWNDLWLN EGFAKFMEFV SVRKTHPELK VEDHFLNKYF NAMEVDALNS
401 SHPISTPVED PAQILEMFDE VSYDKGACIL NMLQDYLSPE VFQAGLAKYL LRFSYQNTKN KDLWDSLSDV CPYADGSHIQ
481 NGVCIRNKKK VLNSHWTKAV LQDVQAVMNT WTLQKGFPLV TVTIKGKNIH LQQEHYSKKA TLSSAGSLWH IPLTYITNKC
561 NDVRRFLLTT KTNNIVLPEE VEWIKFNVGM NGYYIVHYGD HGWDSLIR**LL KDNHETISSN DRASLINNAF QLVSAGK**LSI
641 EKALDLTLYL KHESEIAPVY QGLNELIPLY KLLEKTNISD TEQQLK**AYIV NLFK**NMIDKQ SWDDEGTVSE QMLRNSLLTF
721 ACVRKYKPCV DK**AQEYFMK**W KNSDGTLKLP NNIKMAVYAV GIQTDENWDF LFSKYQLPEF DTEKNQIEVV LCLSQNKEK**L**
801 **QWLMDQALQG DIIKTQELPS ILVSVGRNPY GYQLAWK**FLK QNWQKLVKKF DFGSHSLAQM ITGITNQYST KEQLAEVK**NY**
881 **FSSMDKK**TSE LRAVQQAIET IEENIQWMDK NLEKLKAWLQ INSKV

Supporting Peptides:

Peptide	Uniq	-10lgP	Mass	Length	ppm	m/z	z	RT	Fraction	Scan	Source File	Area F1	Area F2	Area F3
R.ASLINNAFQLVSAGK.L	Y	67.88	1531.8358	15	-0.2	766.9250	2	59.92	2	F2:17595	12112021_RID_2349_DP2.raw		2.8511E5	
K.TQELPSILVSVGR.N	Y	67.46	1397.7878	13	0.4	699.9015	2	55.31	2	F2:16054	12112021_RID_2349_DP2.raw	4.1868E4	3.6255E5	
K.AYIVNLFK.N	Y	58.66	966.5538	8	0.5	484.2844	2	53.23	2	F2:15426	12112021_RID_2349_DP2.raw		2.4274E5	
K.LQWLMDQALQGDIIK.T	Y	56.30	1770.9338	15	-0.1	886.4741	2	65.16	2	F2:19201	12112021_RID_2349_DP2.raw		7.7937E4	
K.NYFSSM(+15.99)DKK.T	Y	40.82	1134.5016	9	-0.6	568.2578	2	29.43	2	F2:7291	12112021_RID_2349_DP2.raw		3.9503E4	
R.NPYGYQLAWK.F	Y	40.39	1238.6084	10	-0.4	620.3112	2	47.34	2	F2:13416	12112021_RID_2349_DP2.raw		1.0503E6	
K.AQEYFMK.W	Y	39.57	915.4160	7	0.6	458.7155	2	32.48	2	F2:8440	12112021_RID_2349_DP2.raw		3.5139E4	
R.LLKDNHETISSNDR.A	Y	39.52	1640.8118	14	0.6	547.9449	3	22.93	2	F2:5192	12112021_RID_2349_DP2.raw		1.5804E5	

total 8 peptide

TRINITY_DN283
back to list

| Protein Coverage | Supporting Peptides |

Protein Coverage:

1 MLKKSKMFLL LSILSFLCAT SCSFARDESN LFPWDKIRLP KYVVPVHYDL LIHPNLTTLS FTGLSKIEIL ITEQTSSIIL
81 HSKYLQITKA TIHNTKDSAD TAKGMMVLEH PPFEQIALVS TEPLQDGQSY IISIEYSANL SESFHGFYKS TYRTLEGETR
161 VLASTQFEAT AARMAFPCFD EPALKATFSI KIRRSPKHLA LSNMPLVKSV NINEWLIEDQ FDISVKMSTY LVAFIISDFK
241 SVTKRTLRGI KVSVYTVPHK IKQAEYALDA AVKLLDFFEG YFGIAYPLPK QDLAAIPDFQ SGAMENWGLT TYRESALLYD
321 SEKSSVSKKL VITMTVAHEL AHQWFGNLVT MEWWNDLWLN EGFAKFMEFV SVRKTHPELK VEDHFLNKYF NAMEVDALNS
401 SHPISTPVED PAQILEMFDE VSYDKGACIL NMLQDYLSPE VFQAGLAKYL LRFSYQNTKN KDLWDSLSDV CPYADGSHIQ
481 NGVCIRNKKK VLNSHWTKAV LQDVQAVMNT WTLQKGFPLV TVTIKGKNIH LQQEHYSKKA TLSSAGSLWH IPLTYITNKC
561 NDVRRFLLTT KTNNIVLPEE VEWIKFNVGM NGYYIVHYGD HGWDSLIR**LL KDNHETISSN DRASLINNAF QLVSAGK**LSI
641 EKALDLTLYL KHESEIAPVY QGLNELIPLY KLLEKTNISD TEQQLK**AYIV NLFK**NMIDKQ SWDDEGTVSE QMLRNSLLTF
721 ACVRKYKPCV DK**AQEYFMK**W KNSDGTLKLP NNIKMAVYAV GIQTDENWDF LFSKYQLPEF DTEKNQIEVV LCLSQNKEK**L**
801 **QWLMDQALQG DIIKTQELPS ILVSVGRNPY GYQLAWK**FLK QNWQKLVKKF DFGSHSLAQM ITGITNQYST KEQLAEVK**NY**
881 **FSSMDKK**TSE LRAVQQAIET IEENIQWMDK NLEKLKAWLQ INSKV

Supporting Peptides:

Peptide	Uniq	-10lgP	Mass	Length	ppm	m/z	z	RT	Fraction	Scan	Source File	Area F1	Area F2	Area F3
R.ASLINNAFQLVSAGK.L	Y	67.88	1531.8358	15	-0.2	766.9250	2	59.92	2	F2:17595	12112021_RID_2349_DP2.raw		2.8511E5	
K.TQELPSILVSVGR.N	Y	67.46	1397.7878	13	0.4	699.9015	2	55.31	2	F2:16054	12112021_RID_2349_DP2.raw	4.1868E4	3.6255E5	
K.AYIVNLFK.N	Y	58.66	966.5538	8	0.5	484.2844	2	53.23	2	F2:15426	12112021_RID_2349_DP2.raw		2.4274E5	
K.LQWLMDQALQGDIIK.T	Y	56.30	1770.9338	15	-0.1	886.4741	2	65.16	2	F2:19201	12112021_RID_2349_DP2.raw		7.7937E4	
K.NYFSSM(+15.99)DKK.T	Y	40.82	1134.5016	9	-0.6	568.2578	2	29.43	2	F2:7291	12112021_RID_2349_DP2.raw		3.9503E4	

total 8 peptide

Peptide	Uniq	-10lgP	Mass	Length	ppm	m/z	z	RT	Fraction	Scan	Source File	Area F1	Area F2	Area F3
R.NPYGYQLAWK.F	Y	40.39	1238.6084	10	-0.4	620.3112	2	47.34	2	F2:13416	12112021_RID_2349_DP2.raw		1.0503E6	
K.AQEYFMK.W	Y	39.57	915.4160	7	0.6	458.7155	2	32.48	2	F2:8440	12112021_RID_2349_DP2.raw		3.5139E4	
R.LLKDNHETISSNDR.A	Y	39.52	1640.8118	14	0.6	547.9449	3	22.93	2	F2:5192	12112021_RID_2349_DP2.raw		1.5804E5	

total 8 peptides

TRINITY_DN283
back to list

| Protein Coverage | Supporting Peptides |
Protein Coverage:

1 MLKKSKMFLL LSILSFLCAT SCSFARDESN LFPWDKIRLP KYVVPVHYDL LIHPNLTTLS FTGLSKIEIL ITEQTSSIIL
81 HSKYLQITKA TIHNTKDSAD TAKGMMVLEH PPFEQIALVS TEPLQDGQSY IISIEYSANL SESFHGFYKS TYRTLEGETR
161 VLASTQFEAT AARMAFPCFD EPALKATFSI KIRRSPKHLA LSNMPLVKSV NINEWLIEDQ FDISVKMSTY LVAFIISDFK
241 SVTKRTLRGI KVSVYTVPHK IKQAEYALDA AVKLLDFFEG YFGIAYPLPK QDLAAIPDFQ SGAMENWGLT TYRESALLYD
321 SEKSSVSKKL VITMTVAHEL AHQWFGNLVT MEWWNDLWLN EGFAKFMEFV SVRKTHPELK VEDHFLNKYF NAMEVDALNS
401 SHPISTPVED PAQILEMFDE VSYDKGACIL NMLQDYLSPE VFQAGLAKYL LRFSYQNTKN KDLWDSLSDV CPYADGSHIQ
481 NGVCIRNKKK VLNSHWTKAV LQDVQAVMNT WTLQKGFPLV TVTIKGKNIH LQQEHYSKKA TLSSAGSLWH IPLTYITNKC
561 NDVRRFLLTT KTNNIVLPEE VEWIKFNVGM NGYYIVHYGD HGWDSLIR**LL KDNHETISSN DRASLINNAF QLVSAGK**LSI
641 EKALDLTLYL KHESEIAPVY QGLNELIPLY KLLEKTNISD TEQQLK**AYIV NLFK**NMIDKQ SWDDEGTVSE QMLRNSLLTF
721 ACVRKYKPCV DK**AQEYFMK**W KNSDGTLKLP NNIKMAVYAV GIQTDENWDF LFSKYQLPEF DTEKNQIEVV LCLSQNKEK**L**
801 **QWLMDQALQG DIIKTQELPS ILVSVGRNPY GYQLAWK**FLK QNWQKLVKKF DFGSHSLAQM ITGITNQYST KEQLAEVK**NY**
881 **FSSMDKK**TSE LRAVQQAIET IEENIQWMDK NLEKLKAWLQ INSKV

Supporting Peptides:

Peptide	Uniq	-10lgP	Mass	Length	ppm	m/z	z	RT	Fraction	Scan	Source File	Area F1	Area F2	Area F3
R.ASLINNAFQLVSAGK.L	Y	67.88	1531.8358	15	-0.2	766.9250	2	59.92	2	F2:17595	12112021_RID_2349_DP2.raw		2.8511E5	
K.TQELPSILVSVGR.N	Y	67.46	1397.7878	13	0.4	699.9015	2	55.31	2	F2:16054	12112021_RID_2349_DP2.raw	4.1868E4	3.6255E5	
K.AYIVNLFK.N	Y	58.66	966.5538	8	0.5	484.2844	2	53.23	2	F2:15426	12112021_RID_2349_DP2.raw		2.4274E5	
K.LQWLMDQALQGDIIK.T	Y	56.30	1770.9338	15	-0.1	886.4741	2	65.16	2	F2:19201	12112021_RID_2349_DP2.raw		7.7937E4	
K.NYFSSM(+15.99)DKK.T	Y	40.82	1134.5016	9	-0.6	568.2578	2	29.43	2	F2:7291	12112021_RID_2349_DP2.raw		3.9503E4	
R.NPYGYQLAWK.F	Y	40.39	1238.6084	10	-0.4	620.3112	2	47.34	2	F2:13416	12112021_RID_2349_DP2.raw		1.0503E6	
K.AQEYFMK.W	Y	39.57	915.4160	7	0.6	458.7155	2	32.48	2	F2:8440	12112021_RID_2349_DP2.raw		3.5139E4	
R.LLKDNHETISSNDR.A	Y	39.52	1640.8118	14	0.6	547.9449	3	22.93	2	F2:5192	12112021_RID_2349_DP2.raw		1.5804E5	

total 8 peptides

TRINITY_DN283
back to list

| Protein Coverage | Supporting Peptides |
Protein Coverage:

1 MLKKSKMFLL LSILSFLCAT SCSFARDESN LFPWDKIRLP KYVVPVHYDL LIHPNLTTLS FTGLSKIEIL ITEQTSSIIL
81 HSKYLQITKA TIHNTKDSAD TAKGMMVLEH PPFEQIALVS TEPLQDGQSY IISIEYSANL SESFHGFYKS TYRTLEGETR
161 VLASTQFEAT AARMAFPCFD EPALKATFSI KIRRSPKHLA LSNMPLVKSV NINEWLIEDQ FDISVKMSTY LVAFIISDFK
241 SVTKRTLRGI KVSVYTVPHK IKQAEYALDA AVKLLDFFEG YFGIAYPLPK QDLAAIPDFQ SGAMENWGLT TYRESALLYD
321 SEKSSVSKKL VITMTVAHEL AHQWFGNLVT MEWWNDLWLN EGFAKFMEFV SVRKTHPELK VEDHFLNKYF NAMEVDALNS
401 SHPISTPVED PAQILEMFDE VSYDKGACIL NMLQDYLSPE VFQAGLAKYL LRFSYQNTKN KDLWDSLSDV CPYADGSHIQ
481 NGVCIRNKKK VLNSHWTKAV LQDVQAVMNT WTLQKGFPLV TVTIKGKNIH LQQEHYSKKA TLSSAGSLWH IPLTYITNKC
561 NDVRRFLLTT KTNNIVLPEE VEWIKFNVGM NGYYIVHYGD HGWDSLIR**LL KDNHETISSN DRASLINNAF QLVSAGK**LSI
641 EKALDLTLYL KHESEIAPVY QGLNELIPLY KLLEKTNISD TEQQLK**AYIV NLFK**NMIDKQ SWDDEGTVSE QMLRNSLLTF
721 ACVRKYKPCV DK**AQEYFMK**W KNSDGTLKLP NNIKMAVYAV GIQTDENWDF LFSKYQLPEF DTEKNQIEVV LCLSQNKEK**L**
801 **QWLMDQALQG DIIKTQELPS ILVSVGRNPY GYQLAWK**FLK QNWQKLVKKF DFGSHSLAQM ITGITNQYST KEQLAEVK**NY**
881 **FSSMDKK**TSE LRAVQQAIET IEENIQWMDK NLEKLKAWLQ INSKV

Supporting Peptides:

Peptide	Uniq	-10lgP	Mass	Length	ppm	m/z	z	RT	Fraction	Scan	Source File	Area F1	Area F2	Area F3
R.ASLINNAFQLVSAGK.L	Y	67.88	1531.8358	15	-0.2	766.9250	2	59.92	2	F2:17595	12112021_RID_2349_DP2.raw		2.8511E5	
K.TQELPSILVSVGR.N	Y	67.46	1397.7878	13	0.4	699.9015	2	55.31	2	F2:16054	12112021_RID_2349_DP2.raw	4.1868E4	3.6255E5	
K.AYIVNLFK.N	Y	58.66	966.5538	8	0.5	484.2844	2	53.23	2	F2:15426	12112021_RID_2349_DP2.raw		2.4274E5	
K.LQWLMDQALQGDIIK.T	Y	56.30	1770.9338	15	-0.1	886.4741	2	65.16	2	F2:19201	12112021_RID_2349_DP2.raw		7.7937E4	
K.NYFSSM(+15.99)DKK.T	Y	40.82	1134.5016	9	-0.6	568.2578	2	29.43	2	F2:7291	12112021_RID_2349_DP2.raw		3.9503E4	
R.NPYGYQLAWK.F	Y	40.39	1238.6084	10	-0.4	620.3112	2	47.34	2	F2:13416	12112021_RID_2349_DP2.raw		1.0503E6	
K.AQEYFMK.W	Y	39.57	915.4160	7	0.6	458.7155	2	32.48	2	F2:8440	12112021_RID_2349_DP2.raw		3.5139E4	
R.LLKDNHETISSNDR.A	Y	39.52	1640.8118	14	0.6	547.9449	3	22.93	2	F2:5192	12112021_RID_2349_DP2.raw		1.5804E5	

total 8 peptide

TRINITY_DN283
back to list

| Protein Coverage | Supporting Peptides |
Protein Coverage:

1 MLKKSKMFLL LSILSFLCAT SCSFARDESN LFPWDKIRLP KYVVPVHYDL LIHPNLTTLS FTGLSKIEIL ITEQTSSIIL
81 HSKYLQITKA TIHNTKDSAD TAKGMMVLEH PPFEQIALVS TEPLQDGQSY IISIEYSANL SESFHGFYKS TYRTLEGETR
161 VLASTQFEAT AARMAFPCFD EPALKATFSI KIRRSPKHLA LSNMPLVKSV NINEWLIEDQ FDISVKMSTY LVAFIISDFK
241 SVTKRTLRGI KVSVYTVPHK IKQAEYALDA AVKLLDFFEG YFGIAYPLPK QDLAAIPDFQ SGAMENWGLT TYRESALLYD
321 SEKSSVSKKL VITMTVAHEL AHQWFGNLVT MEWWNDLWLN EGFAKFMEFV SVRKTHPELK VEDHFLNKYF NAMEVDALNS
401 SHPISTPVED PAQILEMFDE VSYDKGACIL NMLQDYLSPE VFQAGLAKYL LRFSYQNTKN KDLWDSLSDV CPYADGSHIQ
481 NGVCIRNKKK VLNSHWTKAV LQDVQAVMNT WTLQKGFPLV TVTIKGKNIH LQQEHYSKKA TLSSAGSLWH IPLTYITNKC
561 NDVRRFLLTT KTNNIVLPEE VEWIKFNVGM NGYYIVHYGD HGWDSLIR**LL KDNHETISSN DRASLINNAF QLVSAGK**LSI
641 EKALDLTLYL KHESEIAPVY QGLNELIPLY KLLEKTNISD TEQQLK**AYIV NLFK**NMIDKQ SWDDEGTVSE QMLRNSLLTF
721 ACVRKYKPCV DK**AQEYFMK**W KNSDGTLKLP NNIKMAVYAV GIQTDENWDF LFSKYQLPEF DTEKNQIEVV LCLSQNKEK**L**
801 **QWLMDQALQG DIIKTQELPS ILVSVGRNPY GYQLAWK**FLK QNWQKLVKKF DFGSHSLAQM ITGITNQYST KEQLAEVK**NY**
881 **FSSMDKK**TSE LRAVQQAIET IEENIQWMDK NLEKLKAWLQ INSKV

Supporting Peptides:

Peptide	Uniq	-10lgP	Mass	Length	ppm	m/z	z	RT	Fraction	Scan	Source File	Area F1	Area F2	Area F3
R.ASLINNAFQLVSAGK.L	Y	67.88	1531.8358	15	-0.2	766.9250	2	59.92	2	F2:17595	12112021_RID_2349_DP2.raw		2.8511E5	
K.TQELPSILVSVGR.N	Y	67.46	1397.7878	13	0.4	699.9015	2	55.31	2	F2:16054	12112021_RID_2349_DP2.raw	4.1868E4	3.6255E5	
K.AYIVNLFK.N	Y	58.66	966.5538	8	0.5	484.2844	2	53.23	2	F2:15426	12112021_RID_2349_DP2.raw		2.4274E5	
K.LQWLMDQALQGDIIK.T	Y	56.30	1770.9338	15	-0.1	886.4741	2	65.16	2	F2:19201	12112021_RID_2349_DP2.raw		7.7937E4	
K.NYFSSM(+15.99)DKK.T	Y	40.82	1134.5016	9	-0.6	568.2578	2	29.43	2	F2:7291	12112021_RID_2349_DP2.raw		3.9503E4	
R.NPYGYQLAWK.F	Y	40.39	1238.6084	10	-0.4	620.3112	2	47.34	2	F2:13416	12112021_RID_2349_DP2.raw		1.0503E6	
K.AQEYFMK.W	Y	39.57	915.4160	7	0.6	458.7155	2	32.48	2	F2:8440	12112021_RID_2349_DP2.raw		3.5139E4	
R.LLKDNHETISSNDR.A	Y	39.52	1640.8118	14	0.6	547.9449	3	22.93	2	F2:5192	12112021_RID_2349_DP2.raw		1.5804E5	

total 8 peptide

TRINITY_DN283
back to list

| Protein Coverage | Supporting Peptides |
Protein Coverage:

```
  1 MLKKSKMFLL LSILSFLCAT SCSFARDESN LFPWDKIRLP KYVVPVHYDL LIHPNLTTLS FTGLSKIEIL ITEQTSSIIL
 81 HSKYLQITKA TIHNTKDSAD TAKGMMVLEH PPFEQIALVS TEPLQDGQSY IISIEYSANL SESFHGFYKS TYRTLEGETR
161 VLASTQFEAT AARMAFPCFD EPALKATFSI KIRRSPKHLA LSNMPLVKSV NINEWLIEDQ FDISVKMSTY LVAFIISDFK
241 SVTKRTLRGI KVSVYTVPHK IKQAEYALDA AVKLLDFFEG YFGIAYPLPK QDLAAIPDFQ SGAMENWGLT TYRESALLYD
321 SEKSSVSKKL VITMTVAHEL AHQWFGNLVT MEWWNDLWLN EGFAKFMEFV SVRKTHPELK VEDHFLNKYF NAMEVDALNS
401 SHPISTPVED PAQILEMFDE VSYDKGACIL NMLQDYLSPE VFQAGLAKYL LRFSYQNTKN KDLWDSLSDV CPYADGSHIQ
481 NGVCIRNKKK VLNSHWTKAV LQDVQAVMNT WTLQKGFPLV TVTIKGKNIH LQQEHYSKKA TLSSAGSLWH IPLTYITNKC
561 NDVRRFLLTT KTNNIVLPEE VEWIKFNVGM NGYYIVHYGD HGWDSLIRLL KDNHETISSN DRASLINNAF QLVSAGKLSI
641 EKALDLTLYL KHESEIAPVY QGLNELIPLY KLLEKTNISD TEQQLKAYIV NLFKNMIDKQ SWDDEGTVSE QMLRNSLLTF
721 ACVRKYKPCV DKAQEYFMKW KNSDGTLKLP NNIKMAVYAV GIQTDENWDF LFSKYQLPEF DTEKNQIEVV LCLSQNKEKL
801 QWLMDQALQG DIIKTQELPS ILVSVGRNPY GYQLAWKFLK QNWQKLVKKF DFGSHSLAQM ITGITNQYST KEQLAEVKNY
881 FSSMDKKTSE LRAVQQAIET IEENIQWMDK NLEKLKAWLQ INSKV
```

Supporting Peptides:

Peptide	Uniq	-10lgP	Mass	Length	ppm	m/z	z	RT	Fraction	Scan	Source File	Area F1	Area F2	Area F3
R.ASLINNAFQLVSAGK.L	Y	67.88	1531.8358	15	-0.2	766.9250	2	59.92	2	F2:17595	12112021_RID_2349_DP2.raw		2.8511E5	
K.TQELPSILVSVGR.N	Y	67.46	1397.7878	13	0.4	699.9015	2	55.31	2	F2:16054	12112021_RID_2349_DP2.raw	4.1868E4	3.6255E5	
K.AYIVNLFK.N	Y	58.66	966.5538	8	0.5	484.2844	2	53.23	2	F2:15426	12112021_RID_2349_DP2.raw		2.4274E5	
K.LQWLMDQALQGDIIK.T	Y	56.30	1770.9338	15	-0.1	886.4741	2	65.16	2	F2:19201	12112021_RID_2349_DP2.raw		7.7937E4	
K.NYFSSM(+15.99)DKK.T	Y	40.82	1134.5016	9	-0.6	568.2578	2	29.43	2	F2:7291	12112021_RID_2349_DP2.raw		3.9503E4	
R.NPYGYQLAWK.F	Y	40.39	1238.6084	10	-0.4	620.3112	2	47.34	2	F2:13416	12112021_RID_2349_DP2.raw		1.0503E6	
K.AQEYFMK.W	Y	39.57	915.4160	7	0.6	458.7155	2	32.48	2	F2:8440	12112021_RID_2349_DP2.raw		3.5139E4	
R.LLKDNHETISSNDR.A	Y	39.52	1640.8118	14	0.6	547.9449	3	22.93	2	F2:5192	12112021_RID_2349_DP2.raw		1.5804E5	

total 8 peptide

TRINITY_DN714
back to list

| Protein Coverage | Supporting Peptides |
Protein Coverage:

```
  1 VVDVTLNSFG EWRERDLLNR KKHDNAQLLT GINFNGDTVG FGFVGSICMP KESVGIVQDH SKTHLLVATT MAHELGHNLG
 81 INHDEKQCNC GANSCIMAAM VSDPPPLYFS DCSRDQYQKF LTNFKPDCTL IRPSRTDIVS PPACGNELLE KGEECDCGSL
161 WYCRNPCCDA ATCKLKPGAQ CGDGMCCYQC RFVTAGTVCR PARDECDIPE YCSGQSVECP MDHFQKNGKP CLMNYGYCYN
241 GRCPIMIHQC IALWGPGTTV SPDVCFQKNK SGQDYYYCRR ENNKNIPCAP QDVKCGRLYC KLHNVDTFSC NIRYSDVALD
    323
321 YGMVDHGTKC GDGKVCNSNR QCVDVNTAY
```

Supporting Peptides:

Peptide	Uniq	-10lgP	Mass	Length	ppm	m/z	z	RT	Fraction	Scan	Source File	Area F1	Are
R.YSDVALDYGMVDHGTK.C	Y	87.37	1769.7930	16	0.7	885.9044	2	41.97	4	F4:11928	17112021_RID_2349_DP4.raw		
R.YSDVALDYGM(+15.99)VDHGTK.C	Y	69.95	1785.7880	16	0.5	893.9017	2	37.05	5	F5:10247	17112021_RID_2349_DP5.raw		
K.ESVGIVQDHSK.T	N	58.55	1197.5989	11	0.9	599.8073	2	22.46	5	F5:5229	17112021_RID_2349_DP5.raw		
N.SFGEWRER.D	N	55.07	1065.4991	8	0.1	533.7569	2	31.26	2	F2:7954	12112021_RID_2349_DP2.raw	6.0265E6	2.41
K.HDNAQLLTGINFN.G	N	48.53	1455.7106	13	1.2	728.8634	2	53.68	3	F3:15808	17112021_RID_2349_DP3.raw		
R.ERDLLNR.K	N	42.02	914.4933	7	-0.1	458.2539	2	23.23	1	F1:6203	12112021_RID_2349_DP1.raw	1.8049E7	3.28

t

TRINITY_DN249
back to list

| Protein Coverage | Supporting Peptides |
Protein Coverage:

1 MLLLLFLLSA SWSNVPTNGE VVDSFEANCP QFFLRETPPV IRHPPRSVRI CQRYQNLYRF ATLYNEDNCI PVYSAYIYNP
81 GKAKRPK**KWM IEPQLINSSF QPEMETEGEF LNK**KGSKEAL QESQAILQDY KNLTDCNR**GH LNPNGHQPDY SAKSSTFTLT**
161 **NIVPQFIK**LN GGAWNNYEQS TMVQMTKGCQ ETFAVVGAVP GDTSVSGGRV NRPSHLWSAA CCVIDNNHLR SWAILAR**NDQ**
241 **NVVETFTLGQ LEYQLAR**LYN VNRVSLFHSD CPRQ

Supporting Peptides:

Peptide	Uniq	-10lgP	Mass	Length	ppm	m/z	z	RT	Fraction	Scan	Source File	Area F1	Ar F
R.NDQNVVETFTLGQLEYQLAR.L	Y	78.63	2337.1602	20	0.1	1169.5875	2	68.98	3	F3:20748	17112021_RID_2349_DP3.raw		
R.GHLNPNGHQPDYSAK.S	Y	73.72	1633.7596	15	1.0	545.5944	3	20.98	3	F3:4807	17112021_RID_2349_DP3.raw		
K.KWMIEPQLINSSFQPEMETEGEFLNK.K	Y	56.53	3124.4885	26	-0.5	1042.5029	3	65.30	3	F3:19500	17112021_RID_2349_DP3.raw		
K.SSTFTLTNIVPQFIK.L	Y	54.61	1694.9243	15	1.1	848.4703	2	67.54	3	F3:20255	17112021_RID_2349_DP3.raw		

total 4 peptid

TRINITY_DN249
back to list

| Protein Coverage | Supporting Peptides |
Protein Coverage:

1 MLLLLFLLSA SWSNVPTNGE VVDSFEANCP QFFLRETPPV IRHPPRSVRI CQRYQNLYRF ATLYNEDNCI PVYSAYIYNP
81 GKAKRPK**KWM IEPQLINSSF QPEMETEGEF LNK**KGSKEAL QESQAILQDY KNLTDCNR**GH LNPNGHQPDY SAKSSTFTLT**
161 **NIVPQFIK**LN GGAWNNYEQS TMVQMTKGCQ ETFAVVGAVP GDTSVSGGRV NRPSHLWSAA CCVIDNNHLR SWAILAR**NDQ**
241 **NVVETFTLGQ LEYQLAR**LYN VNRVSLFHSD CPRQ

Supporting Peptides:

Peptide	Uniq	-10lgP	Mass	Length	ppm	m/z	z	RT	Fraction	Scan	Source File	Area F1	Ar F
R.NDQNVVETFTLGQLEYQLAR.L	Y	78.63	2337.1602	20	0.1	1169.5875	2	68.98	3	F3:20748	17112021_RID_2349_DP3.raw		
R.GHLNPNGHQPDYSAK.S	Y	73.72	1633.7596	15	1.0	545.5944	3	20.98	3	F3:4807	17112021_RID_2349_DP3.raw		
K.KWMIEPQLINSSFQPEMETEGEFLNK.K	Y	56.53	3124.4885	26	-0.5	1042.5029	3	65.30	3	F3:19500	17112021_RID_2349_DP3.raw		
K.SSTFTLTNIVPQFIK.L	Y	54.61	1694.9243	15	1.1	848.4703	2	67.54	3	F3:20255	17112021_RID_2349_DP3.raw		

total 4 peptid

TRINITY_DN249
back to list

| Protein Coverage | Supporting Peptides |
Protein Coverage:

1 MLLLLFLLSA SWSNVPTNGE VVDSFEANCP QFFLRETPPV IRHPPRSVRI CQRYQNLYRF ATLYNEDNCI PVYSAYIYNP
81 GKAKRPK**KWM IEPQLINSSF QPEMETEGEF LNK**KGSKEAL QESQAILQDY KNLTDCNR**GH LNPNGHQPDY SAKSSTFTLT**
161 **NIVPQFIK**LN GGAWNNYEQS TMVQMTKGCQ ETFAVVGAVP GDTSVSGGRV NRPSHLWSAA CCVIDNNHLR SWAILAR**NDQ**
241 **NVVETFTLGQ LEYQLAR**LYN VNRVSLFHSD CPRQ

Supporting Peptides:

Peptide	Uniq	-10lgP	Mass	Length	ppm	m/z	z	RT	Fraction	Scan	Source File	Area F1	Ar F
R.NDQNVVETFTLGQLEYQLAR.L	Y	78.63	2337.1602	20	0.1	1169.5875	2	68.98	3	F3:20748	17112021_RID_2349_DP3.raw		
R.GHLNPNGHQPDYSAK.S	Y	73.72	1633.7596	15	1.0	545.5944	3	20.98	3	F3:4807	17112021_RID_2349_DP3.raw		
K.KWMIEPQLINSSFQPEMETEGEFLNK.K	Y	56.53	3124.4885	26	-0.5	1042.5029	3	65.30	3	F3:19500	17112021_RID_2349_DP3.raw		
K.SSTFTLTNIVPQFIK.L	Y	54.61	1694.9243	15	1.1	848.4703	2	67.54	3	F3:20255	17112021_RID_2349_DP3.raw		

total 4 peptid

TRINITY_DN77
back to list

| Protein Coverage | Supporting Peptides |
Protein Coverage:

1 MAAHGWLWGC LLFAVWGSLV LGQEK**IEVLE NK**AVSLPCKA IRSQPNPTRQ EWKFQK**DGTT LLFYHDNK**FT DNYKDR**ATFT**

81 **PYEIHLASVT R**KDSGLYTCE VLGESFSQSQ VQLVVQVPAA K**PTAHVPSVV TIGNK**VVLRC EETEANPRPT FKWFK**NDIQM**

161 **PLDPK**TSTIF KNSSYTIDAD TGVLAFEPAT ALDTGDYSCE AENKVGPPQR **SEVIHMETS**E LNVGGIVAAV VVLLIILGLV

241 IFGIWFAYNR GYFSKRTTSG SKKVIYSQPS NRSDGDFKQT SSFLV

Supporting Peptides:

Peptide	Uniq	-10lgP	Mass	Length	ppm	m/z	z	RT	Fraction	Scan	Source File	Area F1	Area F2	Area F3	Area F4
R.ATFTPYEIHLASVTR.K	Y	73.13	1704.8835	15	0.6	853.4496	2	49.18	4	F4:14269	17112021_RID_2349_DP4.raw				3.0429E6
K.NDIQMPLDPK.T	Y	67.48	1169.5751	10	0.6	585.7952	2	41.29	4	F4:11774	17112021_RID_2349_DP4.raw				7.7188E6
R.SEVIHMETS.E	Y	61.05	1031.4594	9	0.4	516.7372	2	31.03	4	F4:8170	17112021_RID_2349_DP4.raw				1.6121E6
K.DGTTLLFYHDNK.F	Y	55.18	1422.6780	12	0.9	712.3469	2	45.59	4	F4:13096	17112021_RID_2349_DP4.raw				1.0471E6
K.PTAHVPSVVTIGNK.V	Y	48.30	1418.7881	14	0.6	473.9369	3	35.53	4	F4:9662	17112021_RID_2349_DP4.raw				9.978E5
R.SEVIHM(+15.99)ETS.E	Y	43.01	1047.4542	9	-0.1	524.7343	2	31.06	4	F4:8181	17112021_RID_2349_DP4.raw				4.2082E5
K.IEVLENK.A	Y	39.82	843.4702	7	0.1	422.7424	2	28.48	4	F4:7332	17112021_RID_2349_DP4.raw				1.5645E6

total 7 peptides

TRINITY_DN77
back to list

| Protein Coverage | Supporting Peptides |

Protein Coverage:

1 MAAHGWLWGC LLFAVWGSLV LGQEK**IEVLE NK**AVSLPCKA IRSQPNPTRQ EWKFQK**DGTT LLFYHDNK**FT DNYKDR**ATFT**

81 **PYEIHLASVT R**KDSGLYTCE VLGESFSQSQ VQLVVQVPAA K**PTAHVPSVV TIGNK**VVLRC EETEANPRPT FKWFK**NDIQM**

161 **PLDPK**TSTIF KNSSYTIDAD TGVLAFEPAT ALDTGDYSCE AENKVGPPQR **SEVIHMETS**

Supporting Peptides:

Peptide	Uniq	-10lgP	Mass	Length	ppm	m/z	z	RT	Fraction	Scan	Source File	Area F1	Area F2	Area F3	Area F4
R.ATFTPYEIHLASVTR.K	Y	73.13	1704.8835	15	0.6	853.4496	2	49.18	4	F4:14269	17112021_RID_2349_DP4.raw				3.0429E6
K.NDIQMPLDPK.T	Y	67.48	1169.5751	10	0.6	585.7952	2	41.29	4	F4:11774	17112021_RID_2349_DP4.raw				7.7188E6
R.SEVIHMETS	Y	61.05	1031.4594	9	0.4	516.7372	2	31.03	4	F4:8170	17112021_RID_2349_DP4.raw				1.6121E6
K.DGTTLLFYHDNK.F	Y	55.18	1422.6780	12	0.9	712.3469	2	45.59	4	F4:13096	17112021_RID_2349_DP4.raw				1.0471E6
K.PTAHVPSVVTIGNK.V	Y	48.30	1418.7881	14	0.6	473.9369	3	35.53	4	F4:9662	17112021_RID_2349_DP4.raw				9.978E5
R.SEVIHM(+15.99)ETS	Y	43.01	1047.4542	9	-0.1	524.7343	2	31.06	4	F4:8181	17112021_RID_2349_DP4.raw				4.2082E5
K.IEVLENK.A	Y	39.82	843.4702	7	0.1	422.7424	2	28.48	4	F4:7332	17112021_RID_2349_DP4.raw				1.5645E6

total 7 peptides

TRINITY_DN3925
back to list

| Protein Coverage | Supporting Peptides |

Protein Coverage:

1 MYQLWIKCLA AWIFLKRFNG VHVMQAKAPM YPNEPFLVFW NAPTTQCRLR YKVDLDLKTF HIVTNANDSL SGSAVTIFYS

81 NHLGVYPHID DRGHFFHGII PQNESLTK**HL DKSKSDINR**M IPLKTFHGLG VIDWENWRPQ WDRNWGSKNV YRNRSVQFAK

161 **DLHPELSEDK IR**RLAKKQYE KAAKSFMRDT LLLAEEMRPD GYWGYYLYPD CQNYDYKTKG DQYTGKCPEI EMSR**NDQLLW**

241 **LWR**DSTALFP NVYLEIILRS SENALKFVHH RLKESMRIAS MAR**EDYALPV FAYAR**PFYAY TFEPLTQEDL VTTVGESAAM

321 GAAGIVFWGS MQYASTVDSC QKVKK**YMNGP LGR**YIVNVTT AAKICSRVLC RKNGRCVRKH SDSK**AFLHLF PESFR**IMVHA

401 NATEKKVIVK GKLELENLIY LRENFMCHCY QGWKGLYCEE DSINDIRKI

Supporting Peptides:

Peptide	Uniq	-10lgP	Mass	Length	ppm	m/z	z	RT	Fraction	Scan	Source File	Area F1	Area F2	Area F3	A
K.DLHPELSEDKIR.R	Y	69.19	1450.7416	12	0.1	484.5879	3	33.81	1	F1:9852	12112021_RID_2349_DP1.raw	1.1766E6	1.5248E5	1.0234E6	2.

total 6 peptides

Peptide	Uniq	-10lgP	Mass	Length	ppm	m/z	z	RT	Fraction	Scan	Source File	Area F1	Area F2	Area F3	A
R.EDYALPVFAYAR.P	Y	58.82	1413.6927	12	0.2	707.8538	2	59.74	3	F3:17800	17112021_RID_2349_DP3.raw	3.0486E5	2.0754E5	1.9353E6	
R.NDQLLWLWR.D	Y	55.03	1242.6509	9	0.4	622.3329	2	65.33	3	F3:19554	17112021_RID_2349_DP3.raw			6.8841E5	
K.AFLHLFPESFR.I	Y	54.74	1362.7084	11	-0.4	455.2432	3	56.66	3	F3:16716	17112021_RID_2349_DP3.raw			6.974E5	
K.HLDKSKSDINR.M	Y	48.25	1311.6895	11	0.3	438.2372	3	26.05	1	F1:7151	12112021_RID_2349_DP1.raw	0			
K.YMNGPLGR.Y	Y	45.34	906.4382	8	0.1	454.2264	2	31.12	1	F1:8845	12112021_RID_2349_DP1.raw	3.6398E5			

total 6 peptides

TRINITY_DN3925
back to list

| Protein Coverage | Supporting Peptides |
Protein Coverage:

```
  1 MGSQSLPGTI ICFGLYSPGC YSWLLLFALP HLEGRSPHFV LDLILLWRST FGLCFTEALE RLLFWDLFRL PLSCAWTLPG
 81 GLDLEPWNLP CERTPTLGAF LELTVPEKLH IMYQLWIKCL AAWIFLKRFN GVHVMQAKAP MYPNEPFLVF WNAPTTQCRL
161 RYKVDLDLKT FHIVTNANDS LSGSAVTIFY SNHLGVYPHI DDRGHFFHGI IPQNESLTKH LDKSKSDINR MIPLKTFHGL
241 GVIDWENWRP QWDRNWGSKN VYRNRSVQFA KDLHPELSED KIRRLAKKQY EKAAKSFMRD TLLLAEEMRP DGYWGYYLYP
321 DCQNYDYKTK GDQYTGKCPE IEMSRNDQLL WLWRDSTALF PNVYLEIILR SSENALKFVH HRLKESMRIA SMAREDYALP
401 VFAYARPFYA YTFEPLTQED LVTTVGESAA MGAAGIVFWG SMQYASTVDS CQKVKKYMNG PLGRYIVNVT TAAKICSRVL
481 CRKNGRCVRK HSDSKAFLHL FPESFRIMVH ANATEKKVIV KGKLELENLI YLRENFMCHC YQGWKGLYCE EDSINDIRKI
```

Supporting Peptides:

Peptide	Uniq	-10lgP	Mass	Length	ppm	m/z	z	RT	Fraction	Scan	Source File	Area F1	Area F2	Area F3	A
K.DLHPELSEDKIR.R	Y	69.19	1450.7416	12	0.1	484.5879	3	33.81	1	F1:9852	12112021_RID_2349_DP1.raw	1.1766E6	1.5248E5	1.0234E6	2.
R.EDYALPVFAYAR.P	Y	58.82	1413.6927	12	0.2	707.8538	2	59.74	3	F3:17800	17112021_RID_2349_DP3.raw	3.0486E5	2.0754E5	1.9353E6	
R.NDQLLWLWR.D	Y	55.03	1242.6509	9	0.4	622.3329	2	65.33	3	F3:19554	17112021_RID_2349_DP3.raw			6.8841E5	
K.AFLHLFPESFR.I	Y	54.74	1362.7084	11	-0.4	455.2432	3	56.66	3	F3:16716	17112021_RID_2349_DP3.raw			6.974E5	
K.HLDKSKSDINR.M	Y	48.25	1311.6895	11	0.3	438.2372	3	26.05	1	F1:7151	12112021_RID_2349_DP1.raw	0			
K.YMNGPLGR.Y	Y	45.34	906.4382	8	0.1	454.2264	2	31.12	1	F1:8845	12112021_RID_2349_DP1.raw	3.6398E5			

total 6 peptides

APB93444.1
back to list

| Protein Coverage | Supporting Peptides |
Protein Coverage:

```
                                                                      74
  1 MGRFISVSFS FLVVFLSLSG TGADCPSDWS SHEGHCYKVF KERKTWEDAE KFCTEQARGG HLISLKSTEE VDFMIKLAYP  o C
 81 ILKADLVWIG LRDFWRDCHM GWRDHAQLLY KAWSDEPNCF AAKTTDNQWL RRKCNSSQYF VCQSRVPR
```

Supporting Peptides:

Peptide	Uniq	-10lgP	Mass	Length	ppm	m/z	z	RT	Fraction	Scan	Source File	Area F1	Area F2	Area F3
K.STEEVDFMIK.L	N	71.04	1197.5587	10	0.3	599.7868	2	46.71	5	F5:13738	17112021_RID_2349_DP5.raw		4.9086E5	[illegible]
K.STEEVDFM(+15.99)IK.L	N	68.50	1213.5536	10	1.2	607.7848	2	41.28	7	F7:11785	17112021_RID_2349_DP7.raw	2.9758E5	1.0635E5	
K.TTDNQWLR.R	N	59.51	1032.4989	8	0.4	517.2570	2	33.81	7	F7:9330	17112021_RID_2349_DP7.raw			
K.ADLVWIGLR.D	Y	57.52	1041.5970	9	0.0	521.8058	2	58.17	5	F5:17416	17112021_RID_2349_DP5.raw			
K.TWEDAEK.F	N	42.92	877.3817	7	0.0	439.6982	2	23.16	7	F7:5532	17112021_RID_2349_DP7.raw			
R.GGHLISLK.S	N	41.82	823.4916	8	0.0	412.7531	2	27.22	7	F7:6877	17112021_RID_2349_DP7.raw			
R.KTWEDAEKF.C	N	40.81	1152.5450	9	0.6	577.2802	2	34.65	7	F7:9456	17112021_RID_2349_DP7.raw			
K.TWEDAEKF.C	N	40.38	1024.4502	8	-0.9	513.2319	2	40.86	7	F7:11654	17112021_RID_2349_DP7.raw			

total 9

Peptide	Uniq	-10lgP	Mass	Length	ppm	m/z	z	RT	Fraction	Scan	Source File	Area F1	Area F2	Area F3
K.LAYPILK.A	N	40.30	816.5109	7	0.3	409.2628	2	42.38	5	F5:12212	17112021_RID_2349_DP5.raw			

total 9

TRINITY_DN195

back to list

| Protein Coverage | Supporting Peptides |

Protein Coverage:

```
  1 LNSHAEAEQP AGAGLLGSGR GLGQVGGALR LRTWGICVKS APERRLALLA LLTCLPACLP AGPIMHSRLP VAAPLCLLLL
 81 LLLPWALPAI MPGGLSPRDV TDPEVQEAAV FAVEKYNARS ANDNYFKALR VVEAQSQVVS GVKYYLKMEL VKTTCRKTVG
161 KPKLYQEIQN CNLPPENQQE ELTCQFEVWS RPWLQSTLLT KMSCN
```

Supporting Peptides:

Peptide	Uniq	-10lgP	Mass	Length	ppm	m/z	z	RT	Fraction	Scan	Source File	Area F1	Area F2	Area F3	Area F4
R.DVTDPEVQEAAVFAVEK.Y	Y	82.05	1845.8995	17	0.6	923.9576	2	55.65	7	F7:16625	17112021_RID_2349_DP7.raw				
R.VVEAQSQVVSGVK.Y	Y	75.55	1328.7300	13	0.3	665.3724	2	29.88	7	F7:7845	17112021_RID_2349_DP7.raw				
R.SANDNYFK.A	Y	48.33	957.4192	8	0.0	479.7169	2	26.12	7	F7:6538	17112021_RID_2349_DP7.raw				
P.AIMPGGLSPR.D	Y	46.28	997.5378	10	0.8	499.7766	2	38.34	7	F7:10743	17112021_RID_2349_DP7.raw				
I.MPGGLSPR.D	Y	44.41	813.4167	8	-0.3	407.7155	2	28.28	7	F7:7342	17112021_RID_2349_DP7.raw				

total 5 peptides

TRINITY_DN1793

back to list

| Protein Coverage | Supporting Peptides |

Protein Coverage:

```
  1 MIAFIVLPIL AAVLQQSSGS VDFDSESPRK PEIQNEIVDL HNTLRRSVNP TASNMLKMEW YPEAAANAER WAFRCIYDHS
 81 PRDSRVIGGI KCGENIYMSP YPMKWTAIIH EWHDEKKNFV YGQGASPANA VVGHYTQVVW YKSYRSGCAA AYCPSSKYNY
161 FYVCQYCPAG NIIGKTATPY TSGPPCGDCP SACDNGLCTN PCRREDEFTN CKDLVKQGCH SNYLKTNCPA SCFCHNEII
```

Supporting Peptides:

Peptide	Uniq	-10lgP	Mass	Length	ppm	m/z	z	RT	Fraction	Scan	Source File	Area F1	Area F2	Ar[illegible] F[illegible]
K.MEWYPEAAANAER.W	N	85.56	1536.6667	13	0.5	769.3411	2	45.12	7	F7:13118	17112021_RID_2349_DP7.raw			
K.M(+15.99)EWYPEAAANAER.W	N	83.36	1552.6616	13	0.4	777.3384	2	40.79	7	F7:11501	17112021_RID_2349_DP7.raw			
G.SVDFDSESPR.K	N	67.58	1137.4938	10	-0.1	569.7541	2	30.74	7	F7:8132	17112021_RID_2349_DP7.raw	4.0226E4		
R.KPEIQNEIVDLHNTLR.R	Y	67.27	1918.0272	16	0.5	640.3500	3	44.88	4	F4:12951	17112021_RID_2349_DP4.raw			
R.SVNPTASNMLK.M	N	53.18	1160.5859	11	0.3	581.3004	2	32.34	4	F4:8661	17112021_RID_2349_DP4.raw			
R.SVNPTASNM(+15.99)LK.M	N	48.77	1176.5808	11	0.8	589.2982	2	24.27	4	F4:5833	17112021_RID_2349_DP4.raw			

total 6 [illegible]

TRINITY_DN240

back to list

| Protein Coverage | Supporting Peptides |

Protein Coverage:

```
  1 ICGGTLINQQ WVLSAAHCDM ENMQIYLGLH NFRLPNKDQK RRVAEEKFFC LSNKSYTKWD KDIMLIKLNR RVKTSTHITP
 81 LSLPSNPPRL RSVCRIMGWG SITSPNGRDH CLYSQTQLLM
```

Supporting Peptides:

Peptide	Uniq	-10lgP	Mass	Length	ppm	m/z	z	RT	Fraction	Scan	Source File	Area F1	Area F2	Ar[illegible]
K.TSTHITPLSLPSNPPR.L	Y	84.36	1716.9159	16	0.1	859.4653	2	41.01	3	F3:11600	17112021_RID_2349_DP3.raw		7.6443E6	4.73[illegible]
K.WDKDIMLIK.L	N	57.12	1160.6263	9	-0.1	581.3204	2	46.64	2	F2:13338	12112021_RID_2349_DP2.raw	2.1169E5	9.915E6	1.51[illegible]
K.WDKDIM(+15.99)LIK.L	N	52.20	1176.6212	9	-0.2	589.3177	2	41.27	2	F2:11445	12112021_RID_2349_DP2.raw		6.6323E6	2.11[illegible]
R.VKTSTHITPLSLPSNPPR.L	Y	50.77	1944.0792	18	0.2	649.0338	3	40.72	2	F2:11199	12112021_RID_2349_DP2.raw		5.1195E5	1.00[illegible]

total [illegible]

Peptide	Uniq	-10lgP	Mass	Length	ppm	m/z	z	RT	Fraction	Scan	Source File	Area F1	Area F2	Arc
P.LSLPSNPPR.L	N	41.33	979.5450	9	-0.2	490.7797	2	34.94	7	F7:9564	17112021_RID_2349_DP7.raw			

total

TRINITY_DN240
back to list

| Protein Coverage | Supporting Peptides |
Protein Coverage:

64

1 ICGGTLINQQ WVLSAAHCDM ENMQIYLGLH NFRLPNKDQK RRVAEEKFFC LSNKSYTKWD KDIMLIKLNR RVKTSTHITP C

81 LSLPSNPPRL RSVCRIMGWG SITSPNGRDH CLYSQTQLLM

Supporting Peptides:

Peptide	Uniq	-10lgP	Mass	Length	ppm	m/z	z	RT	Fraction	Scan	Source File	Area F1	Area F2	Arc
K.TSTHITPLSLPSNPPR.L	Y	84.36	1716.9159	16	0.1	859.4653	2	41.01	3	F3:11600	17112021_RID_2349_DP3.raw		7.6443E6	4.73
K.WDKDIMLIK.L	N	57.12	1160.6263	9	-0.1	581.3204	2	46.64	2	F2:13338	12112021_RID_2349_DP2.raw	2.1169E5	9.915E6	1.51
K.WDKDIM(+15.99)LIK.L	N	52.20	1176.6212	9	-0.2	589.3177	2	41.27	2	F2:11445	12112021_RID_2349_DP2.raw		6.6323E6	2.11
R.VKTSTHITPLSLPSNPPR.L	Y	50.77	1944.0792	18	0.2	649.0338	3	40.72	2	F2:11199	12112021_RID_2349_DP2.raw		5.1195E5	1.00
P.LSLPSNPPR.L	N	41.33	979.5450	9	-0.2	490.7797	2	34.94	7	F7:9564	17112021_RID_2349_DP7.raw			

total

TRINITY_DN240
back to list

| Protein Coverage | Supporting Peptides |
Protein Coverage:

64

1 ICGGTLINQQ WVLSAAHCDM ENMQIYLGLH NFRLPNKDQK RRVAEEKFFC LSNKSYTKWD KDIMLIKLNR RVKTSTHITP C

81 LSLPSNPPRL RSVCRIMGWG SITSPNGRDH CLYSQTQLLM

Supporting Peptides:

Peptide	Uniq	-10lgP	Mass	Length	ppm	m/z	z	RT	Fraction	Scan	Source File	Area F1	Area F2	Arc
K.TSTHITPLSLPSNPPR.L	Y	84.36	1716.9159	16	0.1	859.4653	2	41.01	3	F3:11600	17112021_RID_2349_DP3.raw		7.6443E6	4.73
K.WDKDIMLIK.L	N	57.12	1160.6263	9	-0.1	581.3204	2	46.64	2	F2:13338	12112021_RID_2349_DP2.raw	2.1169E5	9.915E6	1.51
K.WDKDIM(+15.99)LIK.L	N	52.20	1176.6212	9	-0.2	589.3177	2	41.27	2	F2:11445	12112021_RID_2349_DP2.raw		6.6323E6	2.11
R.VKTSTHITPLSLPSNPPR.L	Y	50.77	1944.0792	18	0.2	649.0338	3	40.72	2	F2:11199	12112021_RID_2349_DP2.raw		5.1195E5	1.00
P.LSLPSNPPR.L	N	41.33	979.5450	9	-0.2	490.7797	2	34.94	7	F7:9564	17112021_RID_2349_DP7.raw			

total

TRINITY_DN240
back to list

| Protein Coverage | Supporting Peptides |
Protein Coverage:

64

1 ICGGTLINQQ WVLSAAHCDM ENMQIYLGLH NFRLPNKDQK RRVAEEKFFC LSNKSYTKWD KDIMLIKLNR RVKTSTHITP C

81 LSLPSNPPRL RSVCRIMGWG SITSPNGRDH CLYSQTQLLM

Supporting Peptides:

Peptide	Uniq	-10lgP	Mass	Length	ppm	m/z	z	RT	Fraction	Scan	Source File	Area F1	Area F2	Arc
K.TSTHITPLSLPSNPPR.L	Y	84.36	1716.9159	16	0.1	859.4653	2	41.01	3	F3:11600	17112021_RID_2349_DP3.raw		7.6443E6	4.73
K.WDKDIMLIK.L	N	57.12	1160.6263	9	-0.1	581.3204	2	46.64	2	F2:13338	12112021_RID_2349_DP2.raw	2.1169E5	9.915E6	1.51
K.WDKDIM(+15.99)LIK.L	N	52.20	1176.6212	9	-0.2	589.3177	2	41.27	2	F2:11445	12112021_RID_2349_DP2.raw		6.6323E6	2.11

total

Peptide	Uniq	-10lgP	Mass	Length	ppm	m/z	z	RT	Fraction	Scan	Source File	Area F1	Area F2	Ar[illegible]
R.VKTSTHITPLSLPSNPPR.L	Y	50.77	1944.0792	18	0.2	649.0338	3	40.72	2	F2:11199	12112021_RID_2349_DP2.raw		5.1195E5	1.00
P.LSLPSNPPR.L	N	41.33	979.5450	9	-0.2	490.7797	2	34.94	7	F7:9564	17112021_RID_2349_DP7.raw			

total [illegible]

TRINITY_DN240
back to list

| Protein Coverage | Supporting Peptides |
Protein Coverage:

1 ICGGTLINQQ WVLSAAHCDM ENMQIYLGLH NFRLPNKDQK RRVAEEKFFC LSNKSYTK**WD KDIMLIK**LNR R**VKTSTHITP**

81 **LSLPSNPPR**L RSVCRIMGWG SITSPNGRDH CLYSQTQLLM

Supporting Peptides:

Peptide	Uniq	-10lgP	Mass	Length	ppm	m/z	z	RT	Fraction	Scan	Source File	Area F1	Area F2	Ar[illegible]
K.TSTHITPLSLPSNPPR.L	Y	84.36	1716.9159	16	0.1	859.4653	2	41.01	3	F3:11600	17112021_RID_2349_DP3.raw		7.6443E6	4.73
K.WDKDIMLIK.L	N	57.12	1160.6263	9	-0.1	581.3204	2	46.64	2	F2:13338	12112021_RID_2349_DP2.raw	2.1169E5	9.915E6	1.51
K.WDKDIM(+15.99)LIK.L	N	52.20	1176.6212	9	-0.2	589.3177	2	41.27	2	F2:11445	12112021_RID_2349_DP2.raw		6.6323E6	2.11
R.VKTSTHITPLSLPSNPPR.L	Y	50.77	1944.0792	18	0.2	649.0338	3	40.72	2	F2:11199	12112021_RID_2349_DP2.raw		5.1195E5	1.00
P.LSLPSNPPR.L	N	41.33	979.5450	9	-0.2	490.7797	2	34.94	7	F7:9564	17112021_RID_2349_DP7.raw			

total [illegible]

TRINITY_DN240
back to list

| Protein Coverage | Supporting Peptides |
Protein Coverage:

1 ICGGTLINQQ WVLSAAHCDM ENMQIYLGLH NFRLPNKDQK RRVAEEKFFC LSNKSYTK**WD KDIMLIK**LNR R**VKTSTHITP**

81 **LSLPSNPPR**L RSVCRIMGWG SITSPNGRDL CLYSQTQFFM

Supporting Peptides:

Peptide	Uniq	-10lgP	Mass	Length	ppm	m/z	z	RT	Fraction	Scan	Source File	Area F1	Area F2	Ar[illegible]
K.TSTHITPLSLPSNPPR.L	Y	84.36	1716.9159	16	0.1	859.4653	2	41.01	3	F3:11600	17112021_RID_2349_DP3.raw		7.6443E6	4.73
K.WDKDIMLIK.L	N	57.12	1160.6263	9	-0.1	581.3204	2	46.64	2	F2:13338	12112021_RID_2349_DP2.raw	2.1169E5	9.915E6	1.51
K.WDKDIM(+15.99)LIK.L	N	52.20	1176.6212	9	-0.2	589.3177	2	41.27	2	F2:11445	12112021_RID_2349_DP2.raw		6.6323E6	2.11
R.VKTSTHITPLSLPSNPPR.L	Y	50.77	1944.0792	18	0.2	649.0338	3	40.72	2	F2:11199	12112021_RID_2349_DP2.raw		5.1195E5	1.00
P.LSLPSNPPR.L	N	41.33	979.5450	9	-0.2	490.7797	2	34.94	7	F7:9564	17112021_RID_2349_DP7.raw			

total [illegible]

ETE70217.1
back to list

| Protein Coverage | Supporting Peptides |
Protein Coverage:

1 MPMFFFPEGS HISRLDGQLP QQYRIKETGR GENCSYCLQS TWLPRAGLRY PFIMNTYSFR QSSSTISGGG FGGSSSFRTP

81 SIHGGSGGYG ISVSSARLGS SPGFGGGLGS GLGGGSFSYG SGCFGGAGGA GGVFGGNLNV SDSLLSGNEK ITMQNLNDRL

161 ANYLDKVR**AL EEANTELEVK** IREWYQKQGP TATRDYSPYY KTIEELRDKI LVETIENNKM ILQIDNARLA ADDFKTKFET

241 EQALRMSVEA DINGLRRVLD ELTLSRADLE MQIESLKEEL AYLKKNHEEE MSALSGQLGG QVSVEVDSAP GIDLTKILAD

321 MRDQYELLAE KNRRDAEAWF SSKTEELNKE VAINTEQLQT SKTEITDLRR TLQGLEIELQ SQLSMKTALE GTLADTEGRY

401 GSQLCQIQSL ISNIEAQLVE VRSDMERQNN DYKMLMDIKT RLEQEIGTYR QLLDGQDFNL ASELSFTMSY YYQSSSSSYG

481 GGGGGGSGRV ASVRLGPSSI HGGAGGRVSA SSARFVSSGS GYGGGYGYGS GLGGGCGIGY GSGGGVGYGS CFGGGVGGGY

561 GGGLDFAVCS GGGGDGGLLT GNEKITMQNL NDRLANYLDK VRALEEANTE LEIKIRDWHQ RQAPSSPTKD YSHYYKTISE

641 LRDKIHDTTI DNSRIILEID NAR**LAADDFR LK**YENELHLR QGVESDINGL RRVLDELTLF KSDMEMQIEE KKEDLAYLKK

721 NHEELMKEYG SQLTGKVSVE MDAAPGVDLT KILADMREQY EQMAERNRRD AEAWFFTKTE ELNREVATHT EQLQTSKTEI

801 SELRRTIQSL EIELQSQLSM KAGLETSLAE TEGRYCAQLA QIQTLITGVE EQLADLRNDM ERQNHEYRML MDIK**TRLEQE**

881 **IATYR**NLLEG QDSKGSKQLK LVKMKNQTKL KSADYFAGLG SPCVPSPSNC SFPHHEEESF SCNVKEMIEF SNSHFSNYLK

961 GVHTLECENA GLECKIREWF EMNSSHGFYH LSSGYLGGPS GCLLQPIGDC YRTSNFHGVS SSLPMPLSFG SPIWLASDAE

1041 TSWGTFSGSQ GNLFMGGNEK RTMQELNDRL ASYLEKVRSL EEANTQLEGC IREWHQKRSH GTQRDFKDYE HNISDMHERI

1121 ETGRVTNAGL VLQIDNAKMA TEDFRLKYEA EKALRQNVQN DVENIRKELD NMTIIITDLE MEIEALREDH ILKKKEHETN

1201 MQAHGSPKDF KVNVKVNKTP QEDLSKILAG IRADYEAIIE KNRRSLDSWF QQQIGNESSE GQHNQEELQN SQKEICELNR

1281 TLQTLEIDFQ TELNKKFALE NNMGETKARY AFQLQSIQNL IKKCEEELSK FRHDIKYQNN QYKVLFGIKT RLEREISTYR

1361 QLLDGNVDGK TSTSFREHRS SNQNLKKIVQ ETVDGEEEEE EDSKDSVNSE THIYTLPMAR SGSVNLKNVF FESGGPSAAG

1441 SIYRMSSIKK PRTPSVHGGA GGFGTRISFG SNYSNTFGSN LLRSTSINEL LLTSNEKTTM QNLNDRLAAY LEKVHSLETS

1521 NSVLEKQIRE WYEKSSPIVK QDYNAFFKVI EELQDKITKA RIDNTRIILQ IDNSKLAADD FRVKYETELS LRLNVENDIT

1601 GLQGLIDELT LVRTDLEHQI ESLKEELAYL KKQHEEETAG LRRQLSGNVS VEVDAAPGID LAQAMENMRQ QYEILAEKNR

1681 QEAKDRFDKL IEQLNVEVTT TTHQLETHRS EITDRRNTLQ SLEIELQSQL SWKTERENAV AEIEAHYRTI LTQIQMAIGN

1761 IEAQLMQLRS DMEQQSIEYS ILLDIKVKLE VEIATYRRLL EGENWRMITS DLETSKEAEK GGEGGRLSGD EKQTMQNLND

1841 RLATYLGNVQ ALEEANSELE SKIAEWYEMF AAGNQAGQQQ DYSEYYAIID DLRNQIVATS IDNGTMILQI DNARLAGADF

1921 KLKYDNELAL HQSVEMDTNG LRKALEEIEI TSSDLKVQIN SFTEELEYMK KNHEEDMGSM NGSTAAGGIN VEMNVTPGVD

2001 LTTLLNEMRG EYEVLAEQNR EDIETWFNEQ SQELNKQINT SVEETSTNKS EITELKRALQ SLEIELQSQQ SLKQSLEATL

2081 VETESQYCGQ LSQIQEVIGS VEGQVQQIRD DMECQNREYE QLLDVKIRLE NEIDTYRSLL DGSERESSSG IQSRSGESAE

2161 QRFKIQEYAT SQSQSKGSGS GDSKQTDKLG SGNNPRDPQK TRVIRTIVED RIGDQVVSTR RGSSTGSFSS GVVWGFRSGW

2241 GKTGGGGGGA TGSCFHIYNS SPAGGDVGLL AGGDKETMQN LNDRLASYLA KVHALEEANG DLEQKIRNWY QKSATHINEG

2321 LHNDSKYNSS IDDLRNKIIA ASIRNASMIL QIDNARLAAD DFRMKYENEL FLHQNVEADI NGLKKVLDEL SMSKSDLEIQ

2401 IESLIEELAF LKKNHEEEMK NWQGTVAGDI NVEINAAPGV DLTAILNKMR TEYEALAEQN RKDAETWFVE KSKELNMTIN

2481 MSAKETTTNK KEITDLRHTL QSLEIDLKTQ LSLRQSLEST LAETEGQYCA QLLQIQGTIS NVENQVQQIR EDMECQNADY

2561 RCLLDIKIR**L ENEIETYR**HL IDGEALGKGM KELSGKRGES VQRSSKDLGS QNLRSEKGSG LRASGSDSRS TKTGTETSTR

2641 KSEARNVEAT TKDKEIKKTR VIKTIIEELD EYGRVTSSKV QSLACWHSTG LPSCSNTMAL SVLTSGSSRQ ISSYSMGGGG

2721 SVRLSSGVGF SGGSKFTGGY SGGGFGGGSL AGGYGTGMGG GFASGSFAGG FGGGFGVGGG DGGLLTGDEK QTMQNLNDRL

2801 ANYLGKVHAL EEANAELEVK IKEWYAKFST PDTDRDYSKY FNIIEDLRNQ ICRHTIENAG IVLQVDNARL AADDFKLKVL

2881 DDLTMNKSDL ELQIESLNEE LLYLKKNHEE ELMSFRGVTS GDVTVEMDAA PGVDLTKLLN DMRAQYEELA DKNRREAEEQ

2961 FNKQARTIYL NVGALRQEIS ANVDQLSSSK SEVTELRRTM QGLELELQAQ IAMKQSLEGT LAETERNYCD QLGQIQIQIS

3041 SLEEQLLQIR SDMENQNAEY QQLLGIKTRL EKEIETYHRL LEGEGGSALN EASQTGVPQP FPPPRHSPWV DDGIEAREQL

3121 AAESANSGDS KVEDRISEKQ IILFMSKSFK KISSYEGGAK MSGIGGGSLY GFGGGSMGGL GSSYGGAICS GYGGSGFGGG

3201 VGFGSASVAG GSYGANYRSS SSVSMSGAYG AGDSVFLAGA EKETMQNLNS RLANYLEKVR SLEEANAELE RKIRHWYEKN

3281 GPGAPGVTRD YSKYHHMIDE LRNQIVNVTI ENANVVLQID NARLAADDFR MKFENELFLR QSVEGDINGL RRVLDDLTMN

3361 RSDLEAQLES LTEELAYLKK NHEEEMTLLR SSSTGDVTVE MNAAPGIDLT KLLNDMRAQY EDLAEQNRRE AEEQFIKMSQ

3441 PLQQQIHDDA GAASTARNEL IELKRSFQSL EIELQSLLAK KASLEGTLAE TEGNYSTQLS QLQLQVSSLE EQLQQIRAET

3521 ECQNSEYQQL LGIKTRLEME IETYRRLLDG ESIRTAYETK VQIREPTKTR VVKTIVEELV DGKVISSQVK SAGNVLQQSS

3601 HPINKVQLAK VEDGSSMELS QLLNEIRANY ETLFSRSQIE TILSAGTQVI YSLLQLQEQT TLQKEQMNDN LFQKVGGSLT

3681 QVKFKFSLFL TMLTWHCKYS LQLEKVITAR MNKDEEILKA ARAELNEARR QWHHMQTEID SLHAMERGLE NSLQATELRY

3761 QTQLKHLETE IEDLEKELQE VRQDIEKQLQ QHEILLNSKM RLEQEIATYR SLLEKEEKRY LCLNRLRLTE KVDEVIKEWE

3841 GSFFKDNPHL RKKSVSLRFD LHLAATDEDC LHMKQKSRPD IEVRLVMRRS CSIPSIKT

Supporting Peptides:

Peptide	Uniq	-10lgP	Mass	Length	ppm	m/z	z	RT	Fraction	Scan	Source File	Area F1	Area F2	Area F3	A
R.ALEEANTELEVK.I	N	67.86	1344.6772	12	0.3	673.3461	2	36.35	6	F6:9844	17112021_RID_2349_DP6.raw				

total 6 pepti

Peptide	Uniq	-10lgP	Mass	Length	ppm	m/z	z	RT	Fraction	Scan	Source File	Area F1	Area F2	Area F3	A
K.TRLEQEIATYR.N	N	60.70	1378.7205	11	-0.1	690.3674	2	36.59	4	F4:9988	17112021_RID_2349_DP4.raw		4.6159E4		9.
R.LEQEIATYR.N	N	59.72	1121.5717	9	-0.1	561.7930	2	32.42	6	F6:8521	17112021_RID_2349_DP6.raw	2.3192E4			
R.LAADDFRLK.Y	N	56.97	1047.5713	9	0.6	524.7932	2	38.21	1	F1:11342	12112021_RID_2349_DP1.raw	4.5823E5			8.
R.LAADDFR.L	N	55.06	806.3922	7	0.0	404.2034	2	31.43	2	F2:8068	12112021_RID_2349_DP2.raw	2.1578E6	1.4275E6	3.7768E6	7.
R.LENEIETYR.H	Y	50.96	1165.5615	9	-0.2	583.7879	2	33.11	6	F6:8783	17112021_RID_2349_DP6.raw				

total 6 pepti

XP_034291429.1
back to list

| Protein Coverage | Supporting Peptides |
Protein Coverage:

```
  1 MAKGFFISKS LGIVGIVLGL GAVATIIALS VVYTQEKQRA DNALANQGPS STITAGATIA GTVQATTAGA TRATTASTTR
 81 ATTASTTRAT TAGANSTITT KPLEPWDHMR LPKTLMPTFY TISLQPFLKE VSPDFYIFKG NSTVEFQCKQ PTDLILIHSK
161 KLNYTLQETF LASLTGINVV APAIERTWLV EKTEYLVVKL KEKLQQDKAY RLHAEFTGEL ADDLAGFYRS AYMEEGKSKL
241 VATTQMQAAD ARKAFPCFDE PAMKANFSIT LIHLPAYKAL SNMPINKTEE VAMSDGSIWT RTEFEPTLKM STYLLAFIVS
321 EFENVSAIQH NTLIQIWGRP KSIKEGQGDY ALNVSWPILS FFEQQYNVPY PLKRLDQVAL PDFNAGAMEN WGLITYRESA
401 LLFDPEFSSI GNKERIVTVI AHEVAHQWFG NLVTLEWWNE LWLNEGFASY VEYLGANEAE PTWNIKDLIV PNDVYRVMAI
481 DALASSHPLS SPAEEINTPA QISEVFDSIS YSKGASVIRM LSEFLTEPVF REGLQSYFET YQYENTVCDD LWKQLQLVVN
561 KKNMSLPDTV KTIMDRWTLQ MGFPVLTVNT TTGIITQKHF LLDPESPVER PSQFNYTWIV PVSWLSKGKE AEMYWLTDTS
641 AENANFSTSA NPAQWLLLNI NVVGYFRVNY DLENWERLMN QLNDDLQKIP VLNRAQIIDD AFNLARAKHV GSDLALNTTR
721 YLAKEREYLP WDTALDNLDY FRLMFDRSEV YGPMQKYIQK QVTPLFDHFK KITLNWTQIP DGLMDQYNQI LAIRTACSYG
801 IPECNELASS WFKAWKANPS INRIPPNLRS AIYCSAIRTG GVEAWDFAWQ MFQQAQVISE ADKLRGALTC SQTPWILQRY
881 LQYTLDSTLI RRQDATSTIN SIASNVVGQP LAWDFVRMNW RTLFQQFGGS SFSFSSLIQS VTQRFASPFE LQQLEQFKAD
961 NADVGFGSAT RALEQALERT KANIKWVAEN QPLVLQWFKE NS
```

Supporting Peptides:

Peptide	Uniq	-10lgP	Mass	Length	ppm	m/z	z	RT	Fraction	Scan	Source File	Area F1	Area F2	Area F3	Area F4
R.AQIIDDAFNLAR.A	N	93.46	1345.6990	12	0.6	673.8572	2	54.45	1	F1:16660	12112021_RID_2349_DP1.raw	1.3295E6			
K.ADNADVGFGSATR.A	N	56.38	1279.5792	13	0.7	640.7974	2	31.66	1	F1:9065	12112021_RID_2349_DP1.raw	7.2844E4			
K.LVATTQMQAADAR.K	Y	51.71	1374.6925	13	0.4	688.3538	2	31.31	1	F1:8938	12112021_RID_2349_DP1.raw	6.0941E4			
R.LMFDRSEVYGPMQK.Y	N	39.50	1699.8062	14	0.1	567.6094	3	44.88	1	F1:13512	12112021_RID_2349_DP1.raw	8.2001E4			

total 4 peptides

XP_034291427.1
back to list

| Protein Coverage | Supporting Peptides |
Protein Coverage:

```
  1 MAKGFFISKS LGIVGIVLGL GAVATIIALS VVYTQEKQRA DNALANQGPS STITAGATIA GTVQATTAGA TRATTASTTR
 81 ATTASTTRAT TAGANSTITT KPLEPWDHMR LPKTLMPTFY TISLQPFLKE VSPDFYIFKG NSTVEFQCKQ PTDLILIHSK
161 KLNYTLQETF LASLTGINVV APAIERTWLV EKTEYLVVKL KEKLQQDKAY RLHAEFTGEL ADDLAGFYRS AYMEEGKSKL
241 VATTQMQAAD ARKAFPCFDE PAMKANFSIT LIHLPAYKAL SNMPINKTEE VAMSDGSIWT RTEFEPTLKM STYLLAFIVS
321 EFENVSAIQH NTLIQIWGRP KSIKEGQGDY ALNVSWPILS FFEQQYNVPY PLKRLDQVAL PDFNAGAMEN WGLITYRESA
401 LLFDPEFSSI GNKERIVTVI AHEVAHQWFG NLVTLEWWNE LWLNEGFASY VEYLGANEAE PTWNIKDLIV PNDVYRVMAI
481 DALASSHPLS SPAEEINTPA QISEVFDSIS YSKGASVIRM LSEFLTEPVF REGLQSYFET YQYENTVCDD LWKQLQLVVN
561 KKNMSLPDTV KTIMDRWTLQ MGFPVLTVNT TTGIITQKHF LLDPESPVER PSQFNYTWIV PVSWLSKGKE AEMYWLTDTS
641 AENANFSTSA NPAQWLLLNI NVVGYFRVNY DLENWERLMN QLNDDLQKIP VLNRAQIIDD AFNLARAKHV GSDLALNTTR
721 YLAKEREYLP WDTALDNLDY FRLMFDRSEV YGPMQKYIQK QVTPLFDHFK KITLNWTQIP DGLMDQYNQI LAIRTACSYG
801 IPECNELASS WFKAWKANPS INRIPPNLRS AIYCSAIRTG GVEAWDFAWQ MFQQAQVISE ADKLRGALTC SQTPWILQRY
881 LQYTLDSTLI RRQDATSTIN SIASNVVGQP LAWDFVRMNW RTLFQQFGGS SFSFSSLIQS VTQRFASPFE LQQLEQFKAD
961 NADVGFGSAT RALEQALERT KANIKWVAEN QPLVLQWFKE NS
```

Supporting Peptides:

Peptide	Uniq	-10lgP	Mass	Length	ppm	m/z	z	RT	Fraction	Scan	Source File	Area F1	Area F2	Area F3	Area F4
R.AQIIDDAFNLAR.A	N	93.46	1345.6990	12	0.6	673.8572	2	54.45	1	F1:16660	12112021_RID_2349_DP1.raw	1.3295E6			
K.ADNADVGFGSATR.A	N	56.38	1279.5792	13	0.7	640.7974	2	31.66	1	F1:9065	12112021_RID_2349_DP1.raw	7.2844E4			
K.LVATTQMQAADAR.K	Y	51.71	1374.6925	13	0.4	688.3538	2	31.31	1	F1:8938	12112021_RID_2349_DP1.raw	6.0941E4			
R.LMFDRSEVYGPMQK.Y	N	39.50	1699.8062	14	0.1	567.6094	3	44.88	1	F1:13512	12112021_RID_2349_DP1.raw	8.2001E4			

total 4 peptides

XP_034291428.1
back to list

| Protein Coverage | Supporting Peptides |
Protein Coverage:

1 MAKGFFISKS LGIVGIVLGL GAVATIIALS VVYTQEKQRA DNALANQGPS STITAGATIA GTVQATTAGA TRATTASTTR
81 ATTASTTRAT TAGANSTITT KPLEPWDHMR LPKTLMPTFY TISLQPFLKE VSPDFYIFKG NSTVEFQCKQ PTDLILIHSK
161 KLNYTLQETF LASLTGINVV APAIERTWLV EKTEYLVVKL KEKLQQDKAY RLHAEFTGEL ADDLAGFYRS AYMEEGKSK**L**
241 **VATTQMQAAD AR**KAFPCFDE PAMKANFSIT LIHLPAYKAL SNMPINKTEE VAMSDGSIWT RTEFEPTLKM STYLLAFIVS
321 EFENVSAIQH NTLIQIWGRP KSIKEGQGDY ALNVSWPILS FFEQQYNVPY PLKRLDQVAL PDFNAGAMEN WGLITYRESA
401 LLFDPEFSSI GNKERIVTVI AHEVAHQWFG NLVTLEWWNE LWLNEGFASY VEYLGANEAE PTWNIKDLIV PNDVYRVMAI
481 DALASSHPLS SPAEEINTPA QISEVFDSIS YSKGASVIRM LSEFLTEPVF REGLQSYFET YQYENTVCDD LWKQLQLVVN
561 KKNMSLPDTV KTIMDRWTLQ MGFPVLTVNT TTGIITQKHF LLDPESPVER PSQFNYTWIV PVSWLSKGKE AEMYWLTDTS
641 AENANFSTSA NPAQWLLLNI NVVGYFRVNY DLENWERLMN QLNDDLQKIP VLNR**AQIIDD AFNLAR**AKHV GSDLALNTTR
721 YLAKEREYLP WDTALDNLDY FR**LMFDRSEV YGPMQK**YIQK QVTPLFDHFK KITLNWTQIP DGLMDQYNQI LAIRTACSYG
801 IPECNELASS WFKAWKANPS INRIPPNLRS AIYCSAIRTG GVEAWDFAWQ MFQQAQVISE ADKLRGALTC SQTPWILQRY
881 LQYTLDSTLI RRQDATSTIN SIASNVVGQP LAWDFVRMNW RTLFQQFGGS SFSFSSLIQS VTQRFASPFE LQQLEQFK**AD**
961 **NADVGFGSAT R**ALEQALERT KANIKWVAEN QPLVLQWFKE NS

Supporting Peptides:

Peptide	Uniq	-10lgP	Mass	Length	ppm	m/z	z	RT	Fraction	Scan	Source File	Area F1	Area F2	Area F3	Area F4
R.AQIIDDAFNLAR.A	N	93.46	1345.6990	12	0.6	673.8572	2	54.45	1	F1:16660	12112021_RID_2349_DP1.raw	1.3295E6			
K.ADNADVGFGSATR.A	N	56.38	1279.5792	13	0.7	640.7974	2	31.66	1	F1:9065	12112021_RID_2349_DP1.raw	7.2844E4			
K.LVATTQMQAADAR.K	Y	51.71	1374.6925	13	0.4	688.3538	2	31.31	1	F1:8938	12112021_RID_2349_DP1.raw	6.0941E4			
R.LMFDRSEVYGPMQK.Y	N	39.50	1699.8062	14	0.1	567.6094	3	44.88	1	F1:13512	12112021_RID_2349_DP1.raw	8.2001E4			

total 4 peptides

TRINITY_DN743
back to list

| Protein Coverage | Supporting Peptides |
Protein Coverage:

1 VASEQSCYLW RLKDSSLCRE GRKTMGRSIS VSFGLLVVFL SPSGAKGSTC PFNWLPMYGL CYKVFNEPKT WDDAEMFCRK
81 YKPGCHLASL HSK**GDSTEFA EYIADYRK**GW GNIWIGLWGK KK**GFSWEWTD GSSTK**YLPWK KNQPNHYLNK EFCAEIVYFT
161 GYRLWNDQDC KVKNAFLCQC RF

Supporting Peptides:

Peptide	Uniq	-10lgP	Mass	Length	ppm	m/z	z	RT	Fraction	Scan	Source File	Area F1	Area F2	Area F3	Area F4	Ar F5
K.GDSTEFAEYIADYRK.G	Y	82.76	1763.8002	15	0.8	882.9081	2	48.40	7	F7:14317	17112021_RID_2349_DP7.raw					
K.GFSWEWTDGSSTK.Y	Y	80.18	1486.6365	13	0.1	744.3256	2	52.71	7	F7:15715	17112021_RID_2349_DP7.raw					
K.GDSTEFAEYIADYR.K	Y	77.07	1635.7052	14	0.6	818.8604	2	55.01	7	F7:16481	17112021_RID_2349_DP7.raw					

total 3 peptides

TRINITY_DN743
back to list

| Protein Coverage | Supporting Peptides |
Protein Coverage:

1 THTHTHTRFF SAGQMPFFVT AVSQMFCRKY KPGCHLASLH SK**GDSTEFAE YIADYRK**GWG NIWIGLWGKK K**GFSWEWTDG**
81 **SSTK**YLPWKK NQPNHYLNKE FCAEIVYFTA TSNFGEEMVG

Supporting Peptides:

Peptide	Uniq	-10lgP	Mass	Length	ppm	m/z	z	RT	Fraction	Scan	Source File	Area F1	Area F2	Area F3	Area F4	Area F5
K.GDSTEFAEYIADYRK.G	Y	82.76	1763.8002	15	0.8	882.9081	2	48.40	7	F7:14317	17112021_RID_2349_DP7.raw					
K.GFSWEWTDGSSTK.Y	Y	80.18	1486.6365	13	0.1	744.3256	2	52.71	7	F7:15715	17112021_RID_2349_DP7.raw					
K.GDSTEFAEYIADYR.K	Y	77.07	1635.7052	14	0.6	818.8604	2	55.01	7	F7:16481	17112021_RID_2349_DP7.raw					

total 3 peptides

TRINITY_DN0
back to list

| Protein Coverage | Supporting Peptides |
Protein Coverage:

1 NKDKQTRVPK QKFFCLSSKN YTMWDKDIML IKLNRPVTYS THIAPLSLPS SPPTVGSVCR IMGWGAITSP NETYPDVPHC

81 ANINVLDHTA CQKAYPWLLA KSRTLCAGIL QGGIDTCHAD SGGPLICNGQ LQGIVSWGYP CGQPRMPGVY TKVFDYTDWI

161 QSIIAGNTAA TCPP

Supporting Peptides:

Peptide	Uniq	-10lgP	Mass	Length	ppm	m/z	z	RT	Fraction	Scan	Source File	Area F1	Area F2	Area l
M.WDKDIMLIK.L	N	57.12	1160.6263	9	-0.1	581.3204	2	46.64	2	F2:13338	12112021_RID_2349_DP2.raw	2.1169E5	9.915E6	1.5104
K.AYPWLLAKSR.T	Y	56.89	1203.6764	10	-0.7	602.8450	2	44.38	4	F4:12697	17112021_RID_2349_DP4.raw			
R.MPGVYTK.V	N	56.65	794.3997	7	0.2	398.2072	2	26.42	4	F4:6530	17112021_RID_2349_DP4.raw	1.5867E7	1.6435E6	9.2719
K.AYPWLLAK.S	Y	56.25	960.5433	8	-0.5	481.2787	2	51.66	4	F4:15033	17112021_RID_2349_DP4.raw	6.4332E6	1.8506E6	1.3941
M.WDKDIM(+15.99)LIK.L	N	52.20	1176.6212	9	-0.2	589.3177	2	41.27	2	F2:11445	12112021_RID_2349_DP2.raw		6.6323E6	2.1157
K.LNRPVTY.S	N	50.91	861.4708	7	0.0	431.7427	2	27.71	2	F2:6756	12112021_RID_2349_DP2.raw	1.1931E5	4.6213E5	
R.M(+15.99)PGVYTK.V	N	49.06	810.3946	7	1.2	811.4028	1	23.10	3	F3:5226	17112021_RID_2349_DP3.raw	6.2476E6	4.4572E5	2.9093

total

TRINITY_DN0
back to list

| Protein Coverage | Supporting Peptides |
Protein Coverage:

1 GVHSKKVPNK DKQTRVPKQK FFCLSSKNYT MWDKDIMLIK LNRPVTYSTH IAPLSLPSSP PTVGSVCRIM GWGAITSPNE

81 TYPDVPHCAN INVLDHTACQ KAYPWLLAKS RTLCAGILQG GIDTCHADSG GPLICNGQLQ GIVSWGYPCG QPRMPGVYTK

161 VFDYTDWIQS IIAGNTAATC PP

Supporting Peptides:

Peptide	Uniq	-10lgP	Mass	Length	ppm	m/z	z	RT	Fraction	Scan	Source File	Area F1	Area F2	Area l
M.WDKDIMLIK.L	N	57.12	1160.6263	9	-0.1	581.3204	2	46.64	2	F2:13338	12112021_RID_2349_DP2.raw	2.1169E5	9.915E6	1.5104
K.AYPWLLAKSR.T	Y	56.89	1203.6764	10	-0.7	602.8450	2	44.38	4	F4:12697	17112021_RID_2349_DP4.raw			
R.MPGVYTK.V	N	56.65	794.3997	7	0.2	398.2072	2	26.42	4	F4:6530	17112021_RID_2349_DP4.raw	1.5867E7	1.6435E6	9.2719
K.AYPWLLAK.S	Y	56.25	960.5433	8	-0.5	481.2787	2	51.66	4	F4:15033	17112021_RID_2349_DP4.raw	6.4332E6	1.8506E6	1.3941
M.WDKDIM(+15.99)LIK.L	N	52.20	1176.6212	9	-0.2	589.3177	2	41.27	2	F2:11445	12112021_RID_2349_DP2.raw		6.6323E6	2.1157
K.LNRPVTY.S	N	50.91	861.4708	7	0.0	431.7427	2	27.71	2	F2:6756	12112021_RID_2349_DP2.raw	1.1931E5	4.6213E5	
R.M(+15.99)PGVYTK.V	N	49.06	810.3946	7	1.2	811.4028	1	23.10	3	F3:5226	17112021_RID_2349_DP3.raw	6.2476E6	4.4572E5	2.9093

total

CBM40646.1
back to list

| Protein Coverage | Supporting Peptides |
Protein Coverage:

1 VLIRVLANLL LLQLSYAQES SELVIGGDEC DINEHPFLVA LHTARSKRFH CAGTLLNKEW VLTAARCDME NMQIYLGLHN

81 ISRPNQDQKR RVPKEKHFCV SSKTYTRWDK DIMLIRLKRP VNDGTHIAPL SLPSNPPSVG SVCRIMGWGS ITTTKVTYPD

161 VPHCANIKLF DYSVCRDAYK GLPEKSRTLC AGILEGGIDS CKVDNGGPLI CNGQFQGIGS WEGHPCAQPL KPALYTNVFE

241 YTDWIEGIIA RNTTVTCPP

Supporting Peptides:

Peptide	Uniq	-10lgP	Mass	Length	ppm	m/z	z	RT	Fraction	Scan	Source File	Area F1	Area F2	Area F3
K.TYTRWDKDIMLIR.L	N	82.41	1709.8923	13	0.4	570.9716	3	49.33	3	F3:14370	17112021_RID_2349_DP3.raw		1.4395E6	1.7394E[illegible]
R.WDKDIMLIR.L	N	59.98	1188.6324	9	0.8	595.3240	2	47.61	2	F2:13483	12112021_RID_2349_DP2.raw		2.0105E7	1.3706E[illegible]
R.WDKDIM(+15.99)LIR.L	N	54.14	1204.6274	9	0.6	603.3214	2	42.22	2	F2:11819	12112021_RID_2349_DP2.raw		1.4264E7	3.0085E[illegible]
R.IM(+15.99)GWGSITTTK.V	Y	46.02	1209.6063	11	-0.6	605.8101	2	67.06	3	F3:20099	17112021_RID_2349_DP3.raw			0
W.DKDIMLIR.L	N	44.22	1002.5532	8	-0.5	502.2836	2	37.38	3	F3:10341	17112021_RID_2349_DP3.raw			5.8616E[illegible]
W.DKDIM(+15.99)LIR.L	N	39.37	1018.5481	8	0.3	510.2815	2	31.54	3	F3:8358	17112021_RID_2349_DP3.raw			1.7097E[illegible]
K.VTYPDVPH.C	N	39.33	926.4498	8	-0.6	464.2319	2	35.54	6	F6:9635	17112021_RID_2349_DP6.raw			

total 7 pep[illegible]

CBM40645.1
back to list

| Protein Coverage | Supporting Peptides |

Protein Coverage:

1 VLIRVLANLL LLQLSYAQES SELVIGGDEC DINEHPFLVA LHTARSKRFH CAGTLLNKEW VLTAARCDRK NIRIKFGVHN

81 KNVQNEDEEM RVPKEKHFCV SSKTYTRWDK DIMLIRLKRP VNDGTHIAPL SLPSNPPSVG SVCRIMGWGS ITTTKVTYPD

161 VPHCANIKLF DYSVCRDAYK GLPEKSRTLC AGILEGGIDS CKVDNGGPLI CNGQFQGIGS WEGHPCAQPL KPALYTNVFE

241 YTDWIEGIIA RNTTVTCPP

Supporting Peptides:

Peptide	Uniq	-10lgP	Mass	Length	ppm	m/z	z	RT	Fraction	Scan	Source File	Area F1	Area F2	Area F3
K.TYTRWDKDIMLIR.L	N	82.41	1709.8923	13	0.4	570.9716	3	49.33	3	F3:14370	17112021_RID_2349_DP3.raw		1.4395E6	1.7394E[illegible]
R.WDKDIMLIR.L	N	59.98	1188.6324	9	0.8	595.3240	2	47.61	2	F2:13483	12112021_RID_2349_DP2.raw		2.0105E7	1.3706E[illegible]
R.WDKDIM(+15.99)LIR.L	N	54.14	1204.6274	9	0.6	603.3214	2	42.22	2	F2:11819	12112021_RID_2349_DP2.raw		1.4264E7	3.0085E[illegible]
R.IM(+15.99)GWGSITTTK.V	Y	46.02	1209.6063	11	-0.6	605.8101	2	67.06	3	F3:20099	17112021_RID_2349_DP3.raw			0
W.DKDIMLIR.L	N	44.22	1002.5532	8	-0.5	502.2836	2	37.38	3	F3:10341	17112021_RID_2349_DP3.raw			5.8616E[illegible]
W.DKDIM(+15.99)LIR.L	N	39.37	1018.5481	8	0.3	510.2815	2	31.54	3	F3:8358	17112021_RID_2349_DP3.raw			1.7097E[illegible]
K.VTYPDVPH.C	N	39.33	926.4498	8	-0.6	464.2319	2	35.54	6	F6:9635	17112021_RID_2349_DP6.raw			

total 7 pep[illegible]

AAR24534.1
back to list

| Protein Coverage | Supporting Peptides |

Protein Coverage:

1 MVLIRVLANL LLLQLSYAQE SSELVIGGDE CDINEHPFLV ALHTARSKRF HCAGTLLNKE WVLTAARCDR KNIRIKFGVH

81 NKNVQNEDEE MRVPKEKHFC VSSKTYTRWD KDIMLIRLKR PVNDGTHIAP LSLPSNPPSV GSVCRIMGWG SITTTKVTYP

161 DVPHCANIKL FDYSVCRDAY KGLPEKSRTL CAGILEGGID SCKVDNGGPL ICNGQFQGIG SWEGHPCAQP LKPALYTNVF

241 EYTDWIEGII ARNTTVTCPP

Supporting Peptides:

Peptide	Uniq	-10lgP	Mass	Length	ppm	m/z	z	RT	Fraction	Scan	Source File	Area F1	Area F2	Area F3
K.TYTRWDKDIMLIR.L	N	82.41	1709.8923	13	0.4	570.9716	3	49.33	3	F3:14370	17112021_RID_2349_DP3.raw		1.4395E6	1.7394E[illegible]
R.WDKDIMLIR.L	N	59.98	1188.6324	9	0.8	595.3240	2	47.61	2	F2:13483	12112021_RID_2349_DP2.raw		2.0105E7	1.3706E[illegible]
R.WDKDIM(+15.99)LIR.L	N	54.14	1204.6274	9	0.6	603.3214	2	42.22	2	F2:11819	12112021_RID_2349_DP2.raw		1.4264E7	3.0085E[illegible]
R.IM(+15.99)GWGSITTTK.V	Y	46.02	1209.6063	11	-0.6	605.8101	2	67.06	3	F3:20099	17112021_RID_2349_DP3.raw			0
W.DKDIMLIR.L	N	44.22	1002.5532	8	-0.5	502.2836	2	37.38	3	F3:10341	17112021_RID_2349_DP3.raw			5.8616E[illegible]
W.DKDIM(+15.99)LIR.L	N	39.37	1018.5481	8	0.3	510.2815	2	31.54	3	F3:8358	17112021_RID_2349_DP3.raw			1.7097E[illegible]
K.VTYPDVPH.C	N	39.33	926.4498	8	-0.6	464.2319	2	35.54	6	F6:9635	17112021_RID_2349_DP6.raw			

total 7 pep

Q6T6S7.1
back to list

| Protein Coverage | Supporting Peptides |
Protein Coverage:

```
  1 MVLIRVLANL LLLQLSYAQE SSELVIGGDE CDINEHPFLV ALHTARSKRF HCAGTLLNKE WVLTAARCDR KNIRIKFGVH
                                          114                                 147
 81 NKNVQNEDEE MRVPKEKHFC VSSKTYTRWD KDIMLIRLKR PVNDGTHIAP LSLPSNPPSV GSVCRIMGWG SITTTKVTYP
161 DVPHCANIKL FDYSVCRDAY KGLPEKSRTL CAGILEGGID SCKVDNGGPL ICNGQFQGIG SWEGHPCAQP LKPALYTNVF
241 EYTDWIEGII ARNTTVTCPP
```

Supporting Peptides:

Peptide	Uniq	-10lgP	Mass	Length	ppm	m/z	z	RT	Fraction	Scan	Source File	Area F1	Area F2	Area F3
K.TYTRWDKDIMLIR.L	N	82.41	1709.8923	13	0.4	570.9716	3	49.33	3	F3:14370	17112021_RID_2349_DP3.raw		1.4395E6	1.7394E[illegible]
R.WDKDIMLIR.L	N	59.98	1188.6324	9	0.8	595.3240	2	47.61	2	F2:13483	12112021_RID_2349_DP2.raw		2.0105E7	1.3706E[illegible]
R.WDKDIM(+15.99)LIR.L	N	54.14	1204.6274	9	0.6	603.3214	2	42.22	2	F2:11819	12112021_RID_2349_DP2.raw		1.4264E7	3.0085E[illegible]
R.IM(+15.99)GWGSITTTK.V	Y	46.02	1209.6063	11	-0.6	605.8101	2	67.06	3	F3:20099	17112021_RID_2349_DP3.raw			0
W.DKDIMLIR.L	N	44.22	1002.5532	8	-0.5	502.2836	2	37.38	3	F3:10341	17112021_RID_2349_DP3.raw			5.8616E[illegible]
W.DKDIM(+15.99)LIR.L	N	39.37	1018.5481	8	0.3	510.2815	2	31.54	3	F3:8358	17112021_RID_2349_DP3.raw			1.7097E[illegible]
K.VTYPDVPH.C	N	39.33	926.4498	8	-0.6	464.2319	2	35.54	6	F6:9635	17112021_RID_2349_DP6.raw			

total 7 pep

TRINITY_DN94
back to list

| Protein Coverage | Supporting Peptides |
Protein Coverage:

```
  1 MMRLRDLILL ACLVAACSAL IRIPLKKFPS MRSIYNEYGT NIQDLTELGQ MLKYKFGGPG AEIPTPEALK NYMDAQYYGE
 81 IGIGTPPQKF TVVFDTGSSN LWVPSVHCSL LDIACLLHHK YDSSKSSTYV KNGTDFAIHY GTGSLSGYLS QDTVTIGDIS
161 VKNQLFGEAT KQPGITFIAA KFDGILGMAY PKISVDKVAP FFDNVMEQGL LEKNIFSFYL NRDPKGETGG ELLFGGTDSQ
241 YYTGDFHWNN VTRQAYWQVH MDKIDVANGL TVCKDGCEAI IDTGTSLITG PTKEIKELQK AIGAKPIIKG QYILQCDKLS
321 TLPTVSLVLG GKSYVLTPDQ YALKVTVQGE TLCLSGFSGL DVPPPGGPLW ILGDVLIGAY YTVFDRDNDS VGFAKCTSKV
```

Supporting Peptides:

Peptide	Uniq	-10lgP	Mass	Length	ppm	m/z	z	RT	Fraction	Scan	Source File	Area F1	Area F2	Area F3	Ar[illegible] F[illegible]
K.SYVLTPDQYALK.V	Y	68.30	1396.7238	12	0.7	699.3696	2	47.84	2	F2:13587	12112021_RID_2349_DP2.raw		3.5687E5		
K.VAPFFDNVMEQGLLEK.N	Y	60.45	1835.9127	16	0.0	918.9636	2	64.86	2	F2:19175	12112021_RID_2349_DP2.raw		3.9332E5		
K.FDGILGM(+15.99)AYPK.I	Y	49.32	1226.6005	11	0.6	614.3079	2	55.72	2	F2:16218	12112021_RID_2349_DP2.raw		5.4166E4		
K.NIFSFYLNR.D	Y	46.37	1172.5978	9	2.2	587.3074	2	59.79	2	F2:17511	12112021_RID_2349_DP2.raw		0		

total 4 peptides

E5AJX2.1
back to list

| Protein Coverage | Supporting Peptides |

Protein Coverage:

```
  1 MVLIRVLANL LLLQLSYAQK SSELVIGGDE CNINEHPFLA FVTSDRRRCA GTLINQEWVL TAAHCNGKYM KIELGVHDKM
 81 VRNEDKQTRV PKQKFFCLSS KEYTMWDKDI MLIRLNTPVN NSTHIAPVSL ASRPPVVGSV CRIMGWGTIS SPKVILPDVP
161 HCANIEIIKY SKCQGVHPEL PAKGRVVCAG IWQGGKDSCH GDSGAPLICN GQLQGLLSWG GDPCAQPLQP GLYTDIFDYS
241 DWIQSIIAGN TTATCPP
```

Supporting Peptides:

Peptide	Uniq	-10lgP	Mass	Length	ppm	m/z	z	RT	Fraction	Scan	Source File	Area F1	Area F2	Area F
M.WDKDIMLIR.L	N	59.98	1188.6324	9	0.8	595.3240	2	47.61	2	F2:13483	12112021_RID_2349_DP2.raw		2.0105E7	1.3706
M.WDKDIM(+15.99)LIR.L	N	54.14	1204.6274	9	0.6	603.3214	2	42.22	2	F2:11819	12112021_RID_2349_DP2.raw		1.4264E7	3.0085
Y.TMWDKDIMLIR.L	N	50.40	1420.7207	11	0.6	711.3680	2	53.82	3	F3:15851	17112021_RID_2349_DP3.raw			1.1002
R.IMGWGTISSPK.V	Y	46.07	1175.6008	11	0.7	588.8081	2	66.82	3	F3:20035	17112021_RID_2349_DP3.raw			5.4903
W.DKDIMLIR.L	N	44.22	1002.5532	8	-0.5	502.2836	2	37.38	3	F3:10341	17112021_RID_2349_DP3.raw			5.8616
W.DKDIM(+15.99)LIR.L	N	39.37	1018.5481	8	0.3	510.2815	2	31.54	3	F3:8358	17112021_RID_2349_DP3.raw			1.7097
R.VVC(+57.02)AGIWQGGK.D	N	39.18	1173.5964	11	0.7	587.8059	2	34.83	4	F4:9494	17112021_RID_2349_DP4.raw			

CBW30778.1
back to list

| Protein Coverage | Supporting Peptides |

Protein Coverage:

```
  1 MVLIRVLANL LLLQLSYAQK SSELVIGGDE CNINEHPFLA FVTSDRRRCA GTLINQEWVL TAAHCNGKYM KIELGVHDKM
 81 VRNEDKQTRV PKQKFFCLSS KEYTMWDKDI MLIRLNTPVN NSTHIAPVSL ASRPPVVGSV CRIMGWGTIS SPKVILPDVP
161 HCANIEIIKY SKCQGVHPEL PAKGRVVCAG IWQGGKDSCH GDSGAPLICN GQLQGLLSWG GDPCAQPLQP GLYTDIFDYS
241 DWIQSIIAGN TTATCPP
```

Supporting Peptides:

Peptide	Uniq	-10lgP	Mass	Length	ppm	m/z	z	RT	Fraction	Scan	Source File	Area F1	Area F2	Area F
M.WDKDIMLIR.L	N	59.98	1188.6324	9	0.8	595.3240	2	47.61	2	F2:13483	12112021_RID_2349_DP2.raw		2.0105E7	1.3706
M.WDKDIM(+15.99)LIR.L	N	54.14	1204.6274	9	0.6	603.3214	2	42.22	2	F2:11819	12112021_RID_2349_DP2.raw		1.4264E7	3.0085
Y.TMWDKDIMLIR.L	N	50.40	1420.7207	11	0.6	711.3680	2	53.82	3	F3:15851	17112021_RID_2349_DP3.raw			1.1002
R.IMGWGTISSPK.V	Y	46.07	1175.6008	11	0.7	588.8081	2	66.82	3	F3:20035	17112021_RID_2349_DP3.raw			5.4903
W.DKDIMLIR.L	N	44.22	1002.5532	8	-0.5	502.2836	2	37.38	3	F3:10341	17112021_RID_2349_DP3.raw			5.8616
W.DKDIM(+15.99)LIR.L	N	39.37	1018.5481	8	0.3	510.2815	2	31.54	3	F3:8358	17112021_RID_2349_DP3.raw			1.7097
R.VVC(+57.02)AGIWQGGK.D	N	39.18	1173.5964	11	0.7	587.8059	2	34.83	4	F4:9494	17112021_RID_2349_DP4.raw			

TRINITY_DN4675
back to list

| Protein Coverage | Supporting Peptides |

Protein Coverage:

```
  1 VASEQSCYLW RPRDSSLCRE GRKTMGRSIS VSFGCLVVFL SLSGTGAGLK CPPDSSPYRY FCYRVFKEQK TWEDAERFCV
 81 EHPNDGHLVS IESMEEAEFV AQLISNITGK FITHFWIGLR IEDKKQQCRS EWSDGSSVSY DNLLKREFRK CFGLEKGTGY
161 RMWFNLNCEE TYPFICKVPP YC
```

Supporting Peptides:

Peptide	Uniq	-10lgP	Mass	Length	ppm	m/z	z	RT	Fraction	Scan	Source File	Area F1	Area F2	Area F3	Area F4
R.SEWSDGSSVSYDNLLK.R	Y	83.22	1785.8057	16	0.7	893.9108	2	51.99	5	F5:15311	17112021_RID_2349_DP5.raw				

total 3 peptides

Peptide	Uniq	-10lgP	Mass	Length	ppm	m/z	z	RT	Fraction	Scan	Source File	Area F1	Area F2	Area F3	Area F4
R.SEWSDGSSVSYDNLLKR.E	Y	79.37	1941.9067	17	0.4	971.9610	2	46.71	5	F5:13597	17112021_RID_2349_DP5.raw				
K.TWEDAER.F	N	42.62	905.3879	7	0.3	453.7014	2	24.88	5	F5:6121	17112021_RID_2349_DP5.raw				

total 3 peptides

TRINITY_DN59
back to list

| Protein Coverage | Supporting Peptides |
Protein Coverage:

```
 1 GWSAYDQHCY QAVDEPKTWA DAEKFCTEQA NGGHLVSIEN VGEANFVAQL ASGFIQKPGI YVWIGLRDRR KEQQCRSEWN
81 DGSKIIYVNW KEGESKMCQG LAKWTDFHKW DYVNCAERYR FVCKFPPQC
```

Supporting Peptides:

Peptide	Uniq	-10lgP	Mass	Length	ppm	m/z	z	RT	Fraction	Scan	Source File	Area F1	Area F2	Area F3	Area F4	Area F
K.IIYVNWKEGESK.M	Y	79.43	1464.7612	12	0.4	733.3882	2	38.97	5	F5:10919	17112021_RID_2349_DP5.raw					2.829E7
C.YQAVDEPK.T	N	56.88	948.4552	8	0.4	475.2351	2	22.42	5	F5:5214	17112021_RID_2349_DP5.raw					4.8543E
K.TWADAEKF.C	N	39.07	966.4446	8	0.5	484.2298	2	41.22	5	F5:11753	17112021_RID_2349_DP5.raw					2.1565E

total 3 peptides

TRINITY_DN59
back to list

| Protein Coverage | Supporting Peptides |
Protein Coverage:

```
 1 GWSAYDQHCY QAVDEPKTWA DAEKFCTEQA NGGHLVSIEN VGEANFVAQL ASGFIQKPGI YVWIGLRDRR KEQQCRSEWN
81 DGSKIIYVNW KEGESKMCQG LAKWTDFHKW DYVNCAERYR FVCKFPPQC
```

Supporting Peptides:

Peptide	Uniq	-10lgP	Mass	Length	ppm	m/z	z	RT	Fraction	Scan	Source File	Area F1	Area F2	Area F3	Area F4	Area F
K.IIYVNWKEGESK.M	Y	79.43	1464.7612	12	0.4	733.3882	2	38.97	5	F5:10919	17112021_RID_2349_DP5.raw					2.829E7
C.YQAVDEPK.T	N	56.88	948.4552	8	0.4	475.2351	2	22.42	5	F5:5214	17112021_RID_2349_DP5.raw					4.8543E
K.TWADAEKF.C	N	39.07	966.4446	8	0.5	484.2298	2	41.22	5	F5:11753	17112021_RID_2349_DP5.raw					2.1565E

total 3 peptides

AJO70724.1
back to list

| Protein Coverage | Supporting Peptides |
Protein Coverage:

```
 1 MGRFISVSFG LLVVFLSLSG TGADFDCPSG WSAYDQHCYQ AVDEPKTWAD AEKFCTEQAN GGHLVSIENV GEANFVAQLA
81 SGFIQKPGIY VWIGLRDRRK EQQCRSEWND GSKIIYVNWK EGESKMCQGL AKWTDFHKWD YVNCAERYRF VCKFPPQC
```

Supporting Peptides:

Peptide	Uniq	-10lgP	Mass	Length	ppm	m/z	z	RT	Fraction	Scan	Source File	Area F1	Area F2	Area F3	Area F4	Area F
K.IIYVNWKEGESK.M	Y	79.43	1464.7612	12	0.4	733.3882	2	38.97	5	F5:10919	17112021_RID_2349_DP5.raw					2.829E7
C.YQAVDEPK.T	N	56.88	948.4552	8	0.4	475.2351	2	22.42	5	F5:5214	17112021_RID_2349_DP5.raw					4.8543E
K.TWADAEKF.C	N	39.07	966.4446	8	0.5	484.2298	2	41.22	5	F5:11753	17112021_RID_2349_DP5.raw					2.1565E

total 3 peptides

TRINITY_DN588
back to list

| Protein Coverage | Supporting Peptides |
Protein Coverage:

```
 1 MAAYLLAVAI LFCIQGWPSG TVQGQVRPFL DVYQRSACQA RETLVSILQE YPDEISDIFR PSCVAVLRCS GCCTDESLKC
81 TPVGKHTVDM QIMRVNPRTQ SSKMEVMKFT EHTACECRPR RKQGEPDGPK EKPRRGGVRA KFPFDQLLTP GAL
```

Supporting Peptides:

Peptide	Uniq	-10lgP	Mass	Length	ppm	m/z	z	RT	Fraction	Scan	Source File	Area F1	Area F
K.HTVDMQIMR.V	N	70.60	1129.5372	9	-0.2	565.7758	2	32.19	7	F7:8643	17112021_RID_2349_DP7.raw	1.1569E6	7.4694E
K.HTVDMQIM(+15.99)R.V	N	68.64	1145.5321	9	1.0	573.7739	2	24.76	7	F7:6460	17112021_RID_2349_DP7.raw	5.9884E5	4.6869E
K.HTVDM(+15.99)QIM(+15.99)R.V	N	62.07	1161.5271	9	0.3	581.7710	2	18.23	7	F7:3828	17112021_RID_2349_DP7.raw	7.9377E4	3.9021E
K.HTVDM(+15.99)QIMR.V	N	58.98	1145.5321	9	0.3	573.7735	2	23.27	7	F7:5513	17112021_RID_2349_DP7.raw	7.1174E5	
Q.GQVRPFLDVYQR.S	Y	55.17	1476.7837	12	-0.3	493.2684	3	42.18	7	F7:12014	17112021_RID_2349_DP7.raw		
R.PFLDVYQR.S	N	49.73	1036.5342	8	-0.1	519.2743	2	44.38	7	F7:12800	17112021_RID_2349_DP7.raw		
V.RPFLDVYQR.S	N	45.90	1192.6353	9	1.7	597.3259	2	38.63	7	F7:10909	17112021_RID_2349_DP7.raw		
R.TQSSKMEVMK.F	N	45.83	1167.5627	10	-0.2	584.7885	2	20.39	1	F1:5202	12112021_RID_2349_DP1.raw	1.9831E5	

TRINITY_DN588
back to list

| Protein Coverage | Supporting Peptides |
Protein Coverage:

1 MAAYLLAVAI LFCIQGWPSG TVQ**GQVRPFL DVYQR**SACQA RETLVSILQE YPDEISDIFR PSCVAVLRCS GCCTDESLKC C

90
81 TPVGK**HTVDM QIMR**VNPR**TQ SSKMEVMK**FT EHTACECRPR RKQGEPDGPK EKPRRGGVRA KFPFDQLLTP GAL

Supporting Peptides:

Peptide	Uniq	-10lgP	Mass	Length	ppm	m/z	z	RT	Fraction	Scan	Source File	Area F1	Area F
K.HTVDMQIMR.V	N	70.60	1129.5372	9	-0.2	565.7758	2	32.19	7	F7:8643	17112021_RID_2349_DP7.raw	1.1569E6	7.4694E
K.HTVDMQIM(+15.99)R.V	N	68.64	1145.5321	9	1.0	573.7739	2	24.76	7	F7:6460	17112021_RID_2349_DP7.raw	5.9884E5	4.6869E
K.HTVDM(+15.99)QIM(+15.99)R.V	N	62.07	1161.5271	9	0.3	581.7710	2	18.23	7	F7:3828	17112021_RID_2349_DP7.raw	7.9377E4	3.9021E
K.HTVDM(+15.99)QIMR.V	N	58.98	1145.5321	9	0.3	573.7735	2	23.27	7	F7:5513	17112021_RID_2349_DP7.raw	7.1174E5	
Q.GQVRPFLDVYQR.S	Y	55.17	1476.7837	12	-0.3	493.2684	3	42.18	7	F7:12014	17112021_RID_2349_DP7.raw		
R.PFLDVYQR.S	N	49.73	1036.5342	8	-0.1	519.2743	2	44.38	7	F7:12800	17112021_RID_2349_DP7.raw		
V.RPFLDVYQR.S	N	45.90	1192.6353	9	1.7	597.3259	2	38.63	7	F7:10909	17112021_RID_2349_DP7.raw		
R.TQSSKMEVMK.F	N	45.83	1167.5627	10	-0.2	584.7885	2	20.39	1	F1:5202	12112021_RID_2349_DP1.raw	1.9831E5	

TRINITY_DN6726
back to list

| Protein Coverage | Supporting Peptides |
Protein Coverage:

1 MVLTAEDRRL LQASVAKLGC R**LEDIGADAL NRLLIVYPSS KTYFSHYNLS PGSK**DIIHQG EKVGKALDSA LKHLDDIRGT

81 LSQLSDLHAY N**LRVDPVNFK** LLSKCFHVSL ATHLRNEYNA CCCLAWDKFL EQVSDMLCEK YR

Supporting Peptides:

Peptide	Uniq	-10lgP	Mass	Length	ppm	m/z	z	RT	Fraction	Scan	Source File	Area F1	Area F2	Area F3	Area F4	Area
R.LEDIGADALNR.L	Y	73.37	1185.5989	11	0.8	593.8072	2	39.17	7	F7:11029	17112021_RID_2349_DP7.raw					
K.TYFSHYNLSPGSK.D	Y	66.43	1499.7045	13	1.3	750.8605	2	36.62	7	F7:10149	17112021_RID_2349_DP7.raw					
N.LRVDPVNFK.L	N	42.14	1086.6185	9	0.0	544.3165	2	36.89	7	F7:10234	17112021_RID_2349_DP7.raw					4.6379
R.LLIVYPSSK.T	Y	41.76	1018.6063	9	-0.6	510.3101	2	43.65	7	F7:12634	17112021_RID_2349_DP7.raw					

total 4 peptides

TRINITY_DN3927
back to list

| Protein Coverage | Supporting Peptides |

Protein Coverage:

```
   1 MTPVAGFFLS LSVGVALALD ASLMPPNYNM TEEGARLFAN DYNTTGEIVF FESISASWNY NTNLTNHNAQ KQIAAFLKEQ
  81 EFTELWGGKA KENYGKIWQN FTDPQLKKII SSIQTLGPSN LPVQKREEYN TILSEMDSIY STATVCLPNQ NSHCWALEPD
 161 ITDIMATSHS YRKLLYAWEG WHNSAGNPLR SKYEKFVSLS NEAYKMDGFQ DTGAYWRSWY DSPTFKEDLE QLYQQLEPLY
 241 LNLHAYVRRK LYNQYGSKYI NLKGPIPAHL LGNMWAQQWN NIYDIMVPFA NKSHLDVTNT MKQKNWNTTH MFRVSEEFFT
 321 SLGMFGMPPE FWEKSMLEKP TDGRDVVCHA SAWDFFNRKD FRIKQCTTVT MEQLFTVHHE MGHVQYYLQY KNQPISFRSG
 401 ANPGFHEAIG DVMSLSVSTP IHLKKIELLT HVTEDKESDI NYLLKMALEK IAFLPFGYLI DQWRWDVFSG RTPPSRYNFD
 481 WWYLRTKYQG ICPPIARNET NFDPGAKYHI PGNTPYIRYF VSFILQFQFH KALCQAANHT GDLHTCDIYN SKDAGQKLSE
 561 AMKAGSSQPW QDILQQLTGT NKMDASALLE YFKPVMEWLE KENGKNNETL GWPEFDWRPP VPEGYPEGLD KITDEAEAKA
 641 FLEKYNSTAE VVWNAYTEAS WTYNVNITEH NKQIMLEKNF AMSNHTLQNG LKARQFDSTD FKDPKIKRIL KKLSDIERSA
 721 LPEAELKEYN QLLSEMETIY SVAKICGKDT DCKALDPDLT DTMAKSRDYD ELLNTWKGWR DASGKKIRRH YERYVQLSNK
 801 AAKLNGHADN GAYWRSLYET PTFETDLENI WYQLQPLYFN LHAYVRRALY KKYGEKRINL KGPIPAHLLG NMWAQSWANI
 881 FDLVMPFPDA TKVDATPAMI QQGWTPTKMF EQSDSFFKSL GLIPMPPEFW EKSMLEKPSD GREVVCHASA WDFYNRKDFR
 961 IKQCTVVNMD DLITVHHEMG HVQYFLQYKD QPISFREGAN PGFHEAIGDV MALSVSTPKH LKNIKLLEHL EDNIESDINY
1041 LMSIALDKIA FLPFGYLIDQ WRWKVFDGRI PKKEYNQQWW NLRLKYQGLC PPTPRSEEDF DPGAKFHIPA NVPYVRYFVS
1121 FIIQFQFHQA LCKAAGHTGP LYKCDIYQSK EAGKILGDAL KLGFSRPWPE VMQNITGQPN MSAEGLLTYF EPLTKWLIEK
1201 NRQNNETLGW PEYTWQAYAG GSEVNFLGMS LSSSQIETGQ WILLAIGLML LITTIALSVI YFRRRRKGFK SSSEMELK
```

Supporting Peptides:

Peptide	Uniq	-10lgP	Mass	Length	ppm	m/z	z	RT	Fraction	Scan	Source File	Area F1	Area F2	Area F3	A
K.MFEQSDSFFK.S	Y	55.53	1264.5433	10	0.0	633.2789	2	49.45	1	F1:15052	12112021_RID_2349_DP1.raw	2.2329E5			7.[illegible]
K.VDATPAMIQQGWTPTK.M	Y	54.44	1742.8661	16	1.2	872.4414	2	48.04	1	F1:14537	12112021_RID_2349_DP1.raw	9.7075E4			
K.IISSIQTLGPSNLPVQK.R	Y	50.12	1794.0250	17	1.2	898.0209	2	50.02	1	F1:15184	12112021_RID_2349_DP1.raw	4.8193E5			
R.SEEDFDPGAK.F	Y	47.52	1093.4563	10	0.2	547.7355	2	27.98	1	F1:7775	12112021_RID_2349_DP1.raw	1.7742E5			
K.ALDPDLTDTMAK.S	Y	45.71	1289.6173	12	1.4	645.8168	2	42.38	4	F4:12047	17112021_RID_2349_DP4.raw				4.[illegible]

total 5 peptides

TRINITY_DN2452

back to list

| Protein Coverage | Supporting Peptides |

Protein Coverage:

```
   1 SETHYSPDGR EITTNPPVED HCYYYGRIQN DADSTASISA CNGLKGHFQL QGKTYLIEPL KIPESEAHAI YKYENIEKED
  81 EGPKKCGVTQ TNWESDEPIK KASQLVATSE QRRFPKRYIK LVVVADHRMF TKYNNDSTAI RTWIYEMINT VNEVFLPLNI
 161 HVPLVGVEIW CKEDLISVVS SSGDTLDSFG EWRKRDLLNR KRHDNAYLLT AIDFHG
```

Supporting Peptides:

Peptide	Uniq	-10lgP	Mass	Length	ppm	m/z	z	RT	Fraction	Scan	Source File	Area F1	Area
A.SQLVATSEQR.R	N	61.23	1117.5728	10	0.3	559.7938	2	23.26	1	F1:6173	12112021_RID_2349_DP1.raw	8.3452E4	1.575[illegible]
D.TLDSFGEWR.K	N	52.86	1109.5142	9	0.7	555.7648	2	47.54	2	F2:13529	12112021_RID_2349_DP2.raw		8.079[illegible]
K.EDLISVVSSSGDTLDSFGEWR.K	Y	47.78	2298.0652	21	-0.1	1150.0398	2	71.06	3	F3:21425	17112021_RID_2349_DP3.raw		
K.LVVVADHR.M	Y	47.56	907.5239	8	-0.4	454.7690	2	23.50	2	F2:5404	12112021_RID_2349_DP2.raw	5.2396E5	1.799[illegible]
V.TQTNWESDEPIKK.A	N	44.98	1574.7576	13	0.5	788.3865	2	30.75	1	F1:8710	12112021_RID_2349_DP1.raw	3.3503E5	

total 5 pept

P82475.2

back to list

| Protein Coverage | Supporting Peptides |

Protein Coverage:

```
   1 QVRPFPDVYQ RSACQARETL VSILQEYPDE ISDIFRPSCV AVLRCSGCCT DESLKCTPVG KHTVDMQIMR VNPRTQSSKM  C
  81 EVMKFTEHTA CECRPRRKQG EPDGPKEKPR
```

Supporting Peptides:

Peptide	Uniq	-10lgP	Mass	Length	ppm	m/z	z	RT	Fraction	Scan	Source File	Area F1	Area F

Peptide	Uniq	-10lgP	Mass	Length	ppm	m/z	z	RT	Fraction	Scan	Source File	Area F1	Area F
K.HTVDMQIMR.V	N	70.60	1129.5372	9	-0.2	565.7758	2	32.19	7	F7:8643	17112021_RID_2349_DP7.raw	1.1569E6	7.4694E
K.HTVDMQIM(+15.99)R.V	N	68.64	1145.5321	9	1.0	573.7739	2	24.76	7	F7:6460	17112021_RID_2349_DP7.raw	5.9884E5	4.6869E
K.HTVDM(+15.99)QIM(+15.99)R.V	N	62.07	1161.5271	9	0.3	581.7710	2	18.23	7	F7:3828	17112021_RID_2349_DP7.raw	7.9377E4	3.9021E
K.HTVDM(+15.99)QIMR.V	N	58.98	1145.5321	9	0.3	573.7735	2	23.27	7	F7:5513	17112021_RID_2349_DP7.raw	7.1174E5	
R.PFPDVYQR.S	Y	50.92	1020.5028	8	0.4	511.2589	2	39.38	7	F7:11284	17112021_RID_2349_DP7.raw		
R.TQSSKMEVMK.F	N	45.83	1167.5627	10	-0.2	584.7885	2	20.39	1	F1:5202	12112021_RID_2349_DP1.raw	1.9831E5	
V.RPFPDVYQR.S	Y	42.95	1176.6040	9	0.6	589.3096	2	33.46	7	F7:9070	17112021_RID_2349_DP7.raw		
Q.VRPFPDVYQR.S	Y	42.10	1275.6724	10	0.8	638.8440	2	37.19	7	F7:10314	17112021_RID_2349_DP7.raw		

TRINITY_DN30
back to list

| Protein Coverage | Supporting Peptides |
Protein Coverage:

```
  1 MMQVLLVTIC LAVFPYQGSS IILESGNVND YEVVYPQKVT ALPKGAIQQL EQKYEDAIQY QFKVKGEPVV LHLEKNKDFF
 81 PENYSETHYS PDDREITTNP PVEDHCYYHG HIQNEADSTA SISACNGLKG HFKLQGETYL IEPLKIPDSE AHAVYKYENI
161 EKEDEAPKMC GVTQTNWESD EPIKKASQLV ATSEQRRNFQ KYIELVIVVD NVMFRKYTGN STAIRTRIYE IVNTLNLIYT
241 ILNIHIALVY LEIWSKGDLI NVQSVVDVTL NSFGEWRERD LLNRKRHDNA QLLTGMTFDL STLGITFFAG MCQAYRSVGV
321 IQYYSTTNLR IAVTMAHEIG HNVGMKHDTY YCTCGGNSCI MSAVLSNQPS KPFSNCSNHD YQRYLTRYKP KCIFNPPLRT
401 DIVSRAVCGN EIWEEGEECD CGSPGNCQNP CCNAATCKLT PGSQCADGEC CDQCRIQTAG TVCRRARDEC DVPEHCTGQS
481 AECPVDGFHA NGQPCQNNNG YCYNGNCPIL TDQCISLFGS RATVAEDSCF QENQKGSYYG YCRKENGRKI PCAPEDEKCG
561 RLYCSYNLLG NHIPCYPYYR RNREDKGMVD HGTKCGDGKV CSNRHCVDVT TAY
```

Supporting Peptides:

Peptide	Uniq	-10lgP	Mass	Length	ppm	m/z	z	RT	Fraction	Scan	Source File	Area F1	Area F2	Area F3
A.SQLVATSEQR.R	N	61.23	1117.5728	10	0.3	559.7938	2	23.26	1	F1:6173	12112021_RID_2349_DP1.raw	8.3452E4	1.5759E4	
N.SFGEWRER.D	N	55.07	1065.4991	8	0.1	533.7569	2	31.26	2	F2:7954	12112021_RID_2349_DP2.raw	6.0265E6	2.4122E6	
V.TQTNWESDEPIKK.A	N	44.98	1574.7576	13	0.5	788.3865	2	30.75	1	F1:8710	12112021_RID_2349_DP1.raw	3.3503E5		7.2185E4
R.SVGVIQYYSTTNLR.I	Y	42.94	1599.8257	14	1.7	800.9214	2	48.04	2	F2:13703	12112021_RID_2349_DP2.raw		0	
R.ERDLLNR.K	N	42.02	914.4933	7	-0.1	458.2539	2	23.23	1	F1:6203	12112021_RID_2349_DP1.raw	1.8049E7	3.2804E6	

total 5 peptides

JAS04521.1
back to list

| Protein Coverage | Supporting Peptides |
Protein Coverage:

```
  1 MSMLCYTLII AFLIGIWAAP KSEDNVPLGS PATSDLSDTS CAKTHEALKT SRNTDQHYPA PKKAEDQEFG SAANIIVDPK
 81 LFQKRRFQSP RVLFSTQPPP LSRDEQNVDN ANSLNRNIRA KREDHPVHNR GEYSVCDSVN VWVANKTTAT DIRGNVVTVM
161 VDVNINNNVY KQYFFETKCR NPNPVPTGCR GIDARHWNSY CTTTNTFVKA LTMEGNQASW RFIRIDSACV CVISRKNENF
241 G
```

Supporting Peptides:

Peptide	Uniq	-10lgP	Mass	Length	ppm	m/z	z	RT	Fraction	Scan	Source File	Area F1	Area F2	Area F3
K.ALTMEGNQASWR.F	N	80.22	1362.6350	12	0.4	682.3251	2	38.77	5	F5:10781	17112021_RID_2349_DP5.raw			4.2979E5
K.ALTM(+15.99)EGNQASWR.F	N	77.11	1378.6299	12	0.1	690.3223	2	36.09	5	F5:10910	17112021_RID_2349_DP5.raw			
R.NPNPVPTGC(+57.02)R.G	Y	58.52	1110.5240	10	0.3	556.2695	2	23.20	5	F5:5451	17112021_RID_2349_DP5.raw			
K.QYFFETK.C	N	55.57	961.4545	7	0.5	481.7347	2	40.77	5	F5:11527	17112021_RID_2349_DP5.raw			

AAG12169.1
back to list

| Protein Coverage | Supporting Peptides |

Protein Coverage:

```
  1  MSMLCYTLII TLLIGIWAAP KSEDNVPLGS PATSDLSVTS CTKTHEALKT SRNTDQHYPA PKKEEDQEFG SAANIIVDPK
 81  LFQKRRFQSP RVLFSTQPPP LSRDEQSVDD ANSLNRNIRA KREDHPVHNR GEYSVCDSVN VWVANKTTAT DIRGNVVTVM
                                                                                 213
161  VDVNINNNVY KQYFFETKCR NPNPVPTGCR GIDARHWNSY CTTTNTFVKA LTMEGNQASW RFIRIDTACV CVISRKNENF
241  G
```

Supporting Peptides:

Peptide	Uniq	-10lgP	Mass	Length	ppm	m/z	z	RT	Fraction	Scan	Source File	Area F1	Area F2	Area F3
K.ALTMEGNQASWR.F	N	80.22	1362.6350	12	0.4	682.3251	2	38.77	5	F5:10781	17112021_RID_2349_DP5.raw			4.2979E5
K.ALTM(+15.99)EGNQASWR.F	N	77.11	1378.6299	12	0.1	690.3223	2	36.09	5	F5:10910	17112021_RID_2349_DP5.raw			
R.NPNPVPTGC(+57.02)R.G	Y	58.52	1110.5240	10	0.3	556.2695	2	23.20	5	F5:5451	17112021_RID_2349_DP5.raw			
K.QYFFETK.C	N	55.57	961.4545	7	0.5	481.7347	2	40.77	5	F5:11527	17112021_RID_2349_DP5.raw			

JAG46131.1

back to list

| Protein Coverage | Supporting Peptides |

Protein Coverage:

```
  1  MSMLCYTLII AFLIGIWAAP KSEDNVPLGS PATSDLSDTS CAKTHEALKT SRNTDQHYPA PKKAEDQEFG SAANIIVDPK
 81  LFQKRRFQSP RVLFSTQPPP LSRDEQSVDN ANSFNRNIRA KREDHPVHKR GEYSVCDSVN VWVANKTTAT DIRGNLVTVM
                                                                                 213
161  VDVNINNNVY KQYFFETKCR NPNPVPTGCR GIDARHWNSY CTTTNTFVKA LTMEGNQASW RFIRIDSACV CVISRKNENF
241  G
```

Supporting Peptides:

Peptide	Uniq	-10lgP	Mass	Length	ppm	m/z	z	RT	Fraction	Scan	Source File	Area F1	Area F2	Area F3
K.ALTMEGNQASWR.F	N	80.22	1362.6350	12	0.4	682.3251	2	38.77	5	F5:10781	17112021_RID_2349_DP5.raw			4.2979E5
K.ALTM(+15.99)EGNQASWR.F	N	77.11	1378.6299	12	0.1	690.3223	2	36.09	5	F5:10910	17112021_RID_2349_DP5.raw			
R.NPNPVPTGC(+57.02)R.G	Y	58.52	1110.5240	10	0.3	556.2695	2	23.20	5	F5:5451	17112021_RID_2349_DP5.raw			
K.QYFFETK.C	N	55.57	961.4545	7	0.5	481.7347	2	40.77	5	F5:11527	17112021_RID_2349_DP5.raw			

BAN82015.1

back to list

| Protein Coverage | Supporting Peptides |

Protein Coverage:

```
  1  MSMLCYTLII AFLIGIWAAP KSEDNVPLGS PATSDLSDTS CAKTHEALKT SRNTDQHYPA PKKAEDQEFG SAANIIVDPK
 81  LFQKRRFQSP RVLFSTQPPP LSRDEQSVDN ANSLNRNIRS KREDHPVHNR GEYSVCDSVS VWVANKTTAT DIRGNLVTVM
                                                                                 213
161  VDVNLNNNVY KQYFFETKCR NPNPVPTGCR GIDSRHWNSY CTTTHTFVRA LTMEGNQASW RFIRIDTACV CVISRKNENF
241  G
```

Supporting Peptides:

Peptide	Uniq	-10lgP	Mass	Length	ppm	m/z	z	RT	Fraction	Scan	Source File	Area F1	Area F2	Area F3
R.ALTMEGNQASWR.F	N	80.22	1362.6350	12	0.4	682.3251	2	38.77	5	F5:10781	17112021_RID_2349_DP5.raw			4.2979E5
R.ALTM(+15.99)EGNQASWR.F	N	77.11	1378.6299	12	0.1	690.3223	2	36.09	5	F5:10910	17112021_RID_2349_DP5.raw			
R.NPNPVPTGC(+57.02)R.G	Y	58.52	1110.5240	10	0.3	556.2695	2	23.20	5	F5:5451	17112021_RID_2349_DP5.raw			
K.QYFFETK.C	N	55.57	961.4545	7	0.5	481.7347	2	40.77	5	F5:11527	17112021_RID_2349_DP5.raw			

JAS04781.1

back to list

| Protein Coverage | Supporting Peptides |

Protein Coverage:

1 MSMLCYTLII AFLIGIWAAP KSEDNVPLGS PATSDLSDTS CAKTHEALKT SRNTDQHYPA PKKAEDQEFG SAANIIVDPK
81 LFQKRRFQSP RVLFSTQPPP LSRDEQSVDN ANSFNRNIRA KREDHPVHKR GEYSVCDSVN VWVANKTTAT DIRGNLVTVM
161 VDVNINNNVY KQYFFETKCR NPNPVPTGCR GIDARHWNSY CTTTNTFVKA LTMEGNQASW RFIRIDSACV CVISRKNENF
241 G

Supporting Peptides:

Peptide	Uniq	-10lgP	Mass	Length	ppm	m/z	z	RT	Fraction	Scan	Source File	Area F1	Area F2	Area F3
K.ALTMEGNQASWR.F	N	80.22	1362.6350	12	0.4	682.3251	2	38.77	5	F5:10781	17112021_RID_2349_DP5.raw			4.2979E5
K.ALTM(+15.99)EGNQASWR.F	N	77.11	1378.6299	12	0.1	690.3223	2	36.09	5	F5:10910	17112021_RID_2349_DP5.raw			
R.NPNPVPTGC(+57.02)R.G	Y	58.52	1110.5240	10	0.3	556.2695	2	23.20	5	F5:5451	17112021_RID_2349_DP5.raw			
K.QYFFETK.C	N	55.57	961.4545	7	0.5	481.7347	2	40.77	5	F5:11527	17112021_RID_2349_DP5.raw			

JAI10407.1
back to list

| Protein Coverage | Supporting Peptides |
Protein Coverage:

1 MSMLCYTLII AFLIGIWAAP KSEDNVPLGS PATSDLSDTS CAKTHEALKT SRNTDQHYPA PKKAEDQEFG SAANIIVDPK
81 LFQKRRFQSP RVLFSTQPPP LSRDEQSVDN ANSLNRNIRA KREDHPVHKR GEYSVCDSVN VWVANKTTAT DIRGNLVTVM
161 VDVNINNNVY KQYFFETKCR NPNPVPTGCR GIDARHWNSY CTTTNTFVKA LTMEGNQASW RFIRIDSACV CVISRKNENF
241 G

Supporting Peptides:

Peptide	Uniq	-10lgP	Mass	Length	ppm	m/z	z	RT	Fraction	Scan	Source File	Area F1	Area F2	Area F3
K.ALTMEGNQASWR.F	N	80.22	1362.6350	12	0.4	682.3251	2	38.77	5	F5:10781	17112021_RID_2349_DP5.raw			4.2979E5
K.ALTM(+15.99)EGNQASWR.F	N	77.11	1378.6299	12	0.1	690.3223	2	36.09	5	F5:10910	17112021_RID_2349_DP5.raw			
R.NPNPVPTGC(+57.02)R.G	Y	58.52	1110.5240	10	0.3	556.2695	2	23.20	5	F5:5451	17112021_RID_2349_DP5.raw			
K.QYFFETK.C	N	55.57	961.4545	7	0.5	481.7347	2	40.77	5	F5:11527	17112021_RID_2349_DP5.raw			

JAS05454.1
back to list

| Protein Coverage | Supporting Peptides |
Protein Coverage:

1 MSMLCYTLII AFLIGIWAAP KSEDNVPLGS PATSDLSDTS CAKTHEALKT SRNTDQHYPA PKKAEDQEFG SAANIIVDPK
81 LFQKRRFQSP RVLFSTQPPP LSRDEQSVDN ANSLNRNIRA KREDHPVHNQ GEYSVCDSVD VWVANKTTAT DIRGNLVTVM
161 VDVNINNNVY KQYFFETKCR NPNPVPTGCR GIDARHWNSY CTTTETFVKA LTMEGNQASW RFIRIDSACV CVISRKNENF
241 G

Supporting Peptides:

Peptide	Uniq	-10lgP	Mass	Length	ppm	m/z	z	RT	Fraction	Scan	Source File	Area F1	Area F2	Area F3
K.ALTMEGNQASWR.F	N	80.22	1362.6350	12	0.4	682.3251	2	38.77	5	F5:10781	17112021_RID_2349_DP5.raw			4.2979E5
K.ALTM(+15.99)EGNQASWR.F	N	77.11	1378.6299	12	0.1	690.3223	2	36.09	5	F5:10910	17112021_RID_2349_DP5.raw			
R.NPNPVPTGC(+57.02)R.G	Y	58.52	1110.5240	10	0.3	556.2695	2	23.20	5	F5:5451	17112021_RID_2349_DP5.raw			
K.QYFFETK.C	N	55.57	961.4545	7	0.5	481.7347	2	40.77	5	F5:11527	17112021_RID_2349_DP5.raw			

JAS04703.1
back to list

| Protein Coverage | Supporting Peptides |
Protein Coverage:

1 MSMLCYTLII AFLIGIWAAP KSEDNVPLGS PATSDLSDTS CAKTHEALKT SRNTDQHYPA PKKAEDQEFG SAANIIVDPK
81 LFQKRRFQSP RVLFSTQPPP LSRDEQSVDN ANSLNRNIRA KREDHPVHKR GEYSVCDSVN VWVANKTTAT DIRGNLVTVM
213
161 VDVNINNNVY KQYFFETKCR NPNPVPTGCR GIDARHWNSY CTTTNTFVKA LTMEGNQASW RFIRIDSACV CVISRKNENF
241 G

Supporting Peptides:

Peptide	Uniq	-10lgP	Mass	Length	ppm	m/z	z	RT	Fraction	Scan	Source File	Area F1	Area F2	Area F3
K.ALTMEGNQASWR.F	N	80.22	1362.6350	12	0.4	682.3251	2	38.77	5	F5:10781	17112021_RID_2349_DP5.raw			4.2979E5
K.ALTM(+15.99)EGNQASWR.F	N	77.11	1378.6299	12	0.1	690.3223	2	36.09	5	F5:10910	17112021_RID_2349_DP5.raw			
R.NPNPVPTGC(+57.02)R.G	Y	58.52	1110.5240	10	0.3	556.2695	2	23.20	5	F5:5451	17112021_RID_2349_DP5.raw			
K.QYFFETK.C	N	55.57	961.4545	7	0.5	481.7347	2	40.77	5	F5:11527	17112021_RID_2349_DP5.raw			

JAG46130.1
back to list

| Protein Coverage | Supporting Peptides |
Protein Coverage:

1 MSMLCYTLII AFLIGIWAAP KSEDNVPLGS PATSDLSDTS CAKTHEALKT SRNTDQHYPA PKKAEDQEFG SAANIIVDPK
81 LFQKRRFQSP RVLFSTQPPP LSRDEQSVDN ANSFNRNIRA KREDHPVHKR GEYSVCDSVN VWVANKTTAT DIRGNLVTVM
213
161 VDVNINNNVY KQYFFETKCR NPNPVPTGCR GIDARHWNSY CTTTNTFVKA LTMEGNQASW RFIRIDSACV CVISRKNENF
241 G

Supporting Peptides:

Peptide	Uniq	-10lgP	Mass	Length	ppm	m/z	z	RT	Fraction	Scan	Source File	Area F1	Area F2	Area F3
K.ALTMEGNQASWR.F	N	80.22	1362.6350	12	0.4	682.3251	2	38.77	5	F5:10781	17112021_RID_2349_DP5.raw			4.2979E5
K.ALTM(+15.99)EGNQASWR.F	N	77.11	1378.6299	12	0.1	690.3223	2	36.09	5	F5:10910	17112021_RID_2349_DP5.raw			
R.NPNPVPTGC(+57.02)R.G	Y	58.52	1110.5240	10	0.3	556.2695	2	23.20	5	F5:5451	17112021_RID_2349_DP5.raw			
K.QYFFETK.C	N	55.57	961.4545	7	0.5	481.7347	2	40.77	5	F5:11527	17112021_RID_2349_DP5.raw			

JAA98018.1
back to list

| Protein Coverage | Supporting Peptides |
Protein Coverage:

1 MSMLCYTLII AFLIGIWAAP KSEDNVPLGS PATSDLSDTS YAKTHEALKT SRNTDQHYPA PKKAEDQEFG SAANIIVDPK
81 LFQKRRFQSP RVLFSTQPPP LSRDEQSVDN ANSLNRNIRA KREDHPVHKR GEYSVCDSVD VWVANKTTAT DIRGNLVTVM
213
161 VDVNINNNVY KQYFFETKCR NPNPVPTGCR GIDARHWNSY CTTTNTFVKA LTMEGNQASW RFIRIDSACV CVISRKNENF
241 G

Supporting Peptides:

Peptide	Uniq	-10lgP	Mass	Length	ppm	m/z	z	RT	Fraction	Scan	Source File	Area F1	Area F2	Area F3
K.ALTMEGNQASWR.F	N	80.22	1362.6350	12	0.4	682.3251	2	38.77	5	F5:10781	17112021_RID_2349_DP5.raw			4.2979E5
K.ALTM(+15.99)EGNQASWR.F	N	77.11	1378.6299	12	0.1	690.3223	2	36.09	5	F5:10910	17112021_RID_2349_DP5.raw			
R.NPNPVPTGC(+57.02)R.G	Y	58.52	1110.5240	10	0.3	556.2695	2	23.20	5	F5:5451	17112021_RID_2349_DP5.raw			
K.QYFFETK.C	N	55.57	961.4545	7	0.5	481.7347	2	40.77	5	F5:11527	17112021_RID_2349_DP5.raw			

AAG30924.1
back to list

| Protein Coverage | Supporting Peptides |
Protein Coverage:

1 MSMLCYTLII AFLIGIWAAP KSEDNVPLGS PATSDLSDTS CAKTHEALKT SRNIDQHYPA PKKAEDQEFG SAANIIVDPK
81 LFQKRRFQSP RVLFSTQPPP LSRDEQSVDN ANSLNRNIRA KREDHPVHKR GEYSVCDSVN VWVANKTTAT DIRGNLVTVM
161 VDVNINNNVY KQYFFETKCR NPNPVPTGCR GIDARHWNSY CTTTNTFVKA LTMEGNQASW RFIRIDSACV CVISRKNENF
241 G

Supporting Peptides:

Peptide	Uniq	-10lgP	Mass	Length	ppm	m/z	z	RT	Fraction	Scan	Source File	Area F1	Area F2	Area F3
K.ALTMEGNQASWR.F	N	80.22	1362.6350	12	0.4	682.3251	2	38.77	5	F5:10781	17112021_RID_2349_DP5.raw			4.2979E5
K.ALTM(+15.99)EGNQASWR.F	N	77.11	1378.6299	12	0.1	690.3223	2	36.09	5	F5:10910	17112021_RID_2349_DP5.raw			
R.NPNPVPTGC(+57.02)R.G	Y	58.52	1110.5240	10	0.3	556.2695	2	23.20	5	F5:5451	17112021_RID_2349_DP5.raw			
K.QYFFETK.C	N	55.57	961.4545	7	0.5	481.7347	2	40.77	5	F5:11527	17112021_RID_2349_DP5.raw			

JAS05315.1
back to list

| Protein Coverage | Supporting Peptides |
Protein Coverage:

1 MSMLCYTLII AFLIGIWAAP KSEDNVPLGS PATSDLSDTS CAKTHEALKT SRNTDQHYPA PKKAEDQEFV SAANIIVDPK
81 LFQKRRFQSP RVLFSTQPPP LSRDEQSVDN ANSLNRNIRA KREDHPVHNR GEYSVCDSVN VWVANKTTAT DIRGNLVTVM
161 VNVNINNNVY KQYFFETKCR NPNPVPTGCR GIDARHWNSY CTTTETFVKA LTMEGNQASW RFIRIDSACV CVISRKNENF
241 G

Supporting Peptides:

Peptide	Uniq	-10lgP	Mass	Length	ppm	m/z	z	RT	Fraction	Scan	Source File	Area F1	Area F2	Area F3
K.ALTMEGNQASWR.F	N	80.22	1362.6350	12	0.4	682.3251	2	38.77	5	F5:10781	17112021_RID_2349_DP5.raw			4.2979E5
K.ALTM(+15.99)EGNQASWR.F	N	77.11	1378.6299	12	0.1	690.3223	2	36.09	5	F5:10910	17112021_RID_2349_DP5.raw			
R.NPNPVPTGC(+57.02)R.G	Y	58.52	1110.5240	10	0.3	556.2695	2	23.20	5	F5:5451	17112021_RID_2349_DP5.raw			
K.QYFFETK.C	N	55.57	961.4545	7	0.5	481.7347	2	40.77	5	F5:11527	17112021_RID_2349_DP5.raw			

JAV01886.1
back to list

| Protein Coverage | Supporting Peptides |
Protein Coverage:

1 MSMLCYTLII TFLTGIWAAP KSEDNVPLGS PATSDLSVTS CTKTHEALKT SRNTDQHYPA PKKEEDQEFG SAANIIVDPK
81 LFQKRRFQSP RVLFSTQPPP LSRDEQSVDN ANSLNRNIRA KREDHPVHNR GEYSVCDSVN VWVANKTTAT DIRGNLVTVM
161 VDVNINNNVY KQYFFETKCR NPNPVPTGCR GIDARHWNSY CTTTNTFVKA LTMEGNQASW RFIRIDTACV CVISRKNENF
241 G

Supporting Peptides:

Peptide	Uniq	-10lgP	Mass	Length	ppm	m/z	z	RT	Fraction	Scan	Source File	Area F1	Area F2	Area F3
K.ALTMEGNQASWR.F	N	80.22	1362.6350	12	0.4	682.3251	2	38.77	5	F5:10781	17112021_RID_2349_DP5.raw			4.2979E5
K.ALTM(+15.99)EGNQASWR.F	N	77.11	1378.6299	12	0.1	690.3223	2	36.09	5	F5:10910	17112021_RID_2349_DP5.raw			
R.NPNPVPTGC(+57.02)R.G	Y	58.52	1110.5240	10	0.3	556.2695	2	23.20	5	F5:5451	17112021_RID_2349_DP5.raw			
K.QYFFETK.C	N	55.57	961.4545	7	0.5	481.7347	2	40.77	5	F5:11527	17112021_RID_2349_DP5.raw			

JAS04865.1
back to list

| Protein Coverage | Supporting Peptides |
Protein Coverage:

1 MSMLCYTLII AFLIGIWAAP KSEDNVPLGS PATSDLSDTS CAKTHEALKT SRNTDQHYPA PKKAEDQEFG SAANIIVDPK
81 LFQKRRFQSP RVLFSTQPPP LSRDEQSVDN ANSFNRNIRA KREDHPVHKR GEYSVCDSVN VWVANKTTAT DIRGNLVTVM
161 VDVNINNNVY KQYFFETKCR NPNPVPTGCR GIDARHWNSY CTTTNTFVKA LTMEGNQASW RFIRIDSACV CVISRKNENF
241 G

Supporting Peptides:

Peptide	Uniq	-10lgP	Mass	Length	ppm	m/z	z	RT	Fraction	Scan	Source File	Area F1	Area F2	Area F3
K.ALTMEGNQASWR.F	N	80.22	1362.6350	12	0.4	682.3251	2	38.77	5	F5:10781	17112021_RID_2349_DP5.raw			4.2979E5
K.ALTM(+15.99)EGNQASWR.F	N	77.11	1378.6299	12	0.1	690.3223	2	36.09	5	F5:10910	17112021_RID_2349_DP5.raw			
R.NPNPVPTGC(+57.02)R.G	Y	58.52	1110.5240	10	0.3	556.2695	2	23.20	5	F5:5451	17112021_RID_2349_DP5.raw			
K.QYFFETK.C	N	55.57	961.4545	7	0.5	481.7347	2	40.77	5	F5:11527	17112021_RID_2349_DP5.raw			

JAA98019.1
back to list

| Protein Coverage | Supporting Peptides |
Protein Coverage:

1 MSMLCYTLII AFLIGIWAAP KSEDNVPLGS PATSDLSDTS YAKTHEALKT SRNTDQHYPA PKKAEDQEFG SAANIIVDPK
81 LFQKRRFQSP RVLFSTQPPP LSRDEQSVDN ANSLNRNIRA KREDHPVHKR GEYSVCDSVN VWVANKTTAT DIRGNLVTVM
161 VDVNINNNVY KQYFFETKCR NPNPVPTGCR GIDARHWNSY CTTTNTFVKA LTMEGNQASW RFIRIDSACV CVISRKNENF
241 G

Supporting Peptides:

Peptide	Uniq	-10lgP	Mass	Length	ppm	m/z	z	RT	Fraction	Scan	Source File	Area F1	Area F2	Area F3
K.ALTMEGNQASWR.F	N	80.22	1362.6350	12	0.4	682.3251	2	38.77	5	F5:10781	17112021_RID_2349_DP5.raw			4.2979E5
K.ALTM(+15.99)EGNQASWR.F	N	77.11	1378.6299	12	0.1	690.3223	2	36.09	5	F5:10910	17112021_RID_2349_DP5.raw			
R.NPNPVPTGC(+57.02)R.G	Y	58.52	1110.5240	10	0.3	556.2695	2	23.20	5	F5:5451	17112021_RID_2349_DP5.raw			
K.QYFFETK.C	N	55.57	961.4545	7	0.5	481.7347	2	40.77	5	F5:11527	17112021_RID_2349_DP5.raw			

JAA94888.1
back to list

| Protein Coverage | Supporting Peptides |
Protein Coverage:

1 MSMLCYTLII AFLIGIWAAP KSEDNVPLGS PATSDLSDTS CAKTHEALKT SRNTDQHYPA PKKAEDQEFG SAANIIVDPK
81 LFQKRRFQSP RVLFSTQPPP LSRDEQSVDN ANSFNRNIRA KREDHPVHKR GEYSVCDSVN VWVANKTTAT DIRGNLVTVM
161 VDVNINNNVY KQYFFETKCR NPNPVPTGCR GIDARHWNSY CTTTNTFVKA LTMEGNQASW RFIRIDSACV CVISRKNENF
241 G

Supporting Peptides:

Peptide	Uniq	-10lgP	Mass	Length	ppm	m/z	z	RT	Fraction	Scan	Source File	Area F1	Area F2	Area F3
K.ALTMEGNQASWR.F	N	80.22	1362.6350	12	0.4	682.3251	2	38.77	5	F5:10781	17112021_RID_2349_DP5.raw			4.2979E5
K.ALTM(+15.99)EGNQASWR.F	N	77.11	1378.6299	12	0.1	690.3223	2	36.09	5	F5:10910	17112021_RID_2349_DP5.raw			
R.NPNPVPTGC(+57.02)R.G	Y	58.52	1110.5240	10	0.3	556.2695	2	23.20	5	F5:5451	17112021_RID_2349_DP5.raw			
K.QYFFETK.C	N	55.57	961.4545	7	0.5	481.7347	2	40.77	5	F5:11527	17112021_RID_2349_DP5.raw			

JAS04864.1
back to list

| Protein Coverage | Supporting Peptides |
Protein Coverage:

1 MSMLCYTLII AFLIGIWAAP KSEDNVPLGS PATSDLSDTS CAKTHEALKT SRNTDQHYPA PKKAEDQEFG SAANIIVDPK
81 LFQKRRFQSP RVLFSTQPPP LSRDEQSVDN ANSFNRNIRA KREDHPVHKR GEYSVCDSVN VWVANKTTAT DIRGNLVTVM
161 VDVNINNNVY KQYFFETKCR NPNPVPTGCR GIDARHWNSY CTTTNTFVKA LTMEGNQASW RFIRIDSACV CVISRKNENF
241 G

Supporting Peptides:

Peptide	Uniq	-10lgP	Mass	Length	ppm	m/z	z	RT	Fraction	Scan	Source File	Area F1	Area F2	Area F3
K.ALTMEGNQASWR.F	N	80.22	1362.6350	12	0.4	682.3251	2	38.77	5	F5:10781	17112021_RID_2349_DP5.raw			4.2979E5
K.ALTM(+15.99)EGNQASWR.F	N	77.11	1378.6299	12	0.1	690.3223	2	36.09	5	F5:10910	17112021_RID_2349_DP5.raw			
R.NPNPVPTGC(+57.02)R.G	Y	58.52	1110.5240	10	0.3	556.2695	2	23.20	5	F5:5451	17112021_RID_2349_DP5.raw			
K.QYFFETK.C	N	55.57	961.4545	7	0.5	481.7347	2	40.77	5	F5:11527	17112021_RID_2349_DP5.raw			

AFJ49196.1
back to list

| Protein Coverage | Supporting Peptides |
Protein Coverage:

1 MSMLCYTLII AFLIGIWAAP KSEDNVPLGS PATSDLSDTS CAKTHEALKT SRNTDQHYPA PKKAEDQEFG SAANIIVDPK
81 LFQKRRFQSP RVLFSTQPPP LSRDEQSVDN ANSLNRNIRA KREDHPVHKR GEYSVCDSVN VWVANKTTAT DIRGNLVTVM
161 VDVNINNNVY KQYFFETKCR NPNPVPTGCR GIDARHWNSY CTTTNTFVKA LTMEGNQASW RFIRIDSACV CVISRKNENF
241 G

Supporting Peptides:

Peptide	Uniq	-10lgP	Mass	Length	ppm	m/z	z	RT	Fraction	Scan	Source File	Area F1	Area F2	Area F3
K.ALTMEGNQASWR.F	N	80.22	1362.6350	12	0.4	682.3251	2	38.77	5	F5:10781	17112021_RID_2349_DP5.raw			4.2979E5
K.ALTM(+15.99)EGNQASWR.F	N	77.11	1378.6299	12	0.1	690.3223	2	36.09	5	F5:10910	17112021_RID_2349_DP5.raw			
R.NPNPVPTGC(+57.02)R.G	Y	58.52	1110.5240	10	0.3	556.2695	2	23.20	5	F5:5451	17112021_RID_2349_DP5.raw			
K.QYFFETK.C	N	55.57	961.4545	7	0.5	481.7347	2	40.77	5	F5:11527	17112021_RID_2349_DP5.raw			

JAS04866.1
back to list

| Protein Coverage | Supporting Peptides |
Protein Coverage:

1 MSMLCYTLII AFLIGIWAAP KSEDNVPLGS PATSDLSDTS CAKTHEALKT SRNTDQHYPA PKKAEDQEFG SAANIIVDPK
81 LFQKRRFQSP RVLFSTQPPP LSRDEQSVDN ANSFNRNIRA KREDHPVHKR GEYSVCDSVN VWVANKTTAT DIRGNLVTVM
161 VDVNINNNVY KQYFFETKCR NPNPVPTGCR GIDARHWNSY CTTTNTFVKA LTMEGNQASW RFIRIDSACV CVISRKNENF
241 G

Supporting Peptides:

Peptide	Uniq	-10lgP	Mass	Length	ppm	m/z	z	RT	Fraction	Scan	Source File	Area F1	Area F2	Area F3
K.ALTMEGNQASWR.F	N	80.22	1362.6350	12	0.4	682.3251	2	38.77	5	F5:10781	17112021_RID_2349_DP5.raw			4.2979E5
K.ALTM(+15.99)EGNQASWR.F	N	77.11	1378.6299	12	0.1	690.3223	2	36.09	5	F5:10910	17112021_RID_2349_DP5.raw			
R.NPNPVPTGC(+57.02)R.G	Y	58.52	1110.5240	10	0.3	556.2695	2	23.20	5	F5:5451	17112021_RID_2349_DP5.raw			
K.QYFFETK.C	N	55.57	961.4545	7	0.5	481.7347	2	40.77	5	F5:11527	17112021_RID_2349_DP5.raw			

JAG43651.1
back to list

| Protein Coverage | Supporting Peptides |
Protein Coverage:

1 MSMLCYTLII AFLIGIWAAP KSEDNVPLGS PATSDLSDTS CAKTHEALKT SRNTDQHYPA PKKAEDQEFG SAANIIVDPK
81 LFQKRRFQSP RVLFSTQPPP LSRDEQSVDN ANSFNRNIRA KREDHPVHKR GEYSVCDSVN VWVANKTTAT DIRGNLVTVM
161 VDVNINNNVY KQYFFETKCR NPNPVPTGCR GIDARHWNSY CTTTNTFVKA LTMEGNQASW RFIRIDSACV CVISRKNENF
241 G

Supporting Peptides:

Peptide	Uniq	-10lgP	Mass	Length	ppm	m/z	z	RT	Fraction	Scan	Source File	Area F1	Area F2	Area F3
K.ALTMEGNQASWR.F	N	80.22	1362.6350	12	0.4	682.3251	2	38.77	5	F5:10781	17112021_RID_2349_DP5.raw			4.2979E5
K.ALTM(+15.99)EGNQASWR.F	N	77.11	1378.6299	12	0.1	690.3223	2	36.09	5	F5:10910	17112021_RID_2349_DP5.raw			
R.NPNPVPTGC(+57.02)R.G	Y	58.52	1110.5240	10	0.3	556.2695	2	23.20	5	F5:5451	17112021_RID_2349_DP5.raw			
K.QYFFETK.C	N	55.57	961.4545	7	0.5	481.7347	2	40.77	5	F5:11527	17112021_RID_2349_DP5.raw			

JAV48424.1
back to list

| Protein Coverage | Supporting Peptides |
Protein Coverage:

1 MSMLCYTLII AFLIGIWAAP KSEDNVPLGS PATSDLSDTS CAKTHEALKT SRNTDQHYPA PKKAEDQEFG SAANIIVDPK
81 LFQKRRFQSP RVLFSTQPPP LSRDEQSVDN ANSLNRNIRA KREDHPVHKR GEYSVCDSVN VWVANKTTAT DIRGNLVTVM
161 VDVNINNNVY KQYFFETKCR NPNPVPTGCR GIDARHWNSY CTTTNTFVKA LTMEGNQASW RFIRIDSACV CVISRKNENF
241 G

Supporting Peptides:

Peptide	Uniq	-10lgP	Mass	Length	ppm	m/z	z	RT	Fraction	Scan	Source File	Area F1	Area F2	Area F3
K.ALTMEGNQASWR.F	N	80.22	1362.6350	12	0.4	682.3251	2	38.77	5	F5:10781	17112021_RID_2349_DP5.raw			4.2979E5
K.ALTM(+15.99)EGNQASWR.F	N	77.11	1378.6299	12	0.1	690.3223	2	36.09	5	F5:10910	17112021_RID_2349_DP5.raw			
R.NPNPVPTGC(+57.02)R.G	Y	58.52	1110.5240	10	0.3	556.2695	2	23.20	5	F5:5451	17112021_RID_2349_DP5.raw			
K.QYFFETK.C	N	55.57	961.4545	7	0.5	481.7347	2	40.77	5	F5:11527	17112021_RID_2349_DP5.raw			

JAG46132.1
back to list

| Protein Coverage | Supporting Peptides |
Protein Coverage:

1 MSMLCYTLII AFLIGIWAAP KSEDNVPLGS PATSDLSDTS CAKTHEALKT SRNTDQHYPA PKKAEDQEFG SAANIIVDPK
81 LFQKRRFQSP RVLFSTQPPP LSRDEQSVDN ANSFNRNIRA KREDHPVHKR GEYSVCDSVN VWVANKTTAT DIRGNLVTVM
161 VDVNINNNVY KQYFFETKCR NPNPVPTGCR GIDARHWNSY CTTTNTFVKA LTMEGNQASW RFIRIDSACV CVISRKNENF
241 G

Supporting Peptides:

Peptide	Uniq	-10lgP	Mass	Length	ppm	m/z	z	RT	Fraction	Scan	Source File	Area F1	Area F2	Area F3
K.ALTMEGNQASWR.F	N	80.22	1362.6350	12	0.4	682.3251	2	38.77	5	F5:10781	17112021_RID_2349_DP5.raw			4.2979E5
K.ALTM(+15.99)EGNQASWR.F	N	77.11	1378.6299	12	0.1	690.3223	2	36.09	5	F5:10910	17112021_RID_2349_DP5.raw			
R.NPNPVPTGC(+57.02)R.G	Y	58.52	1110.5240	10	0.3	556.2695	2	23.20	5	F5:5451	17112021_RID_2349_DP5.raw			
K.QYFFETK.C	N	55.57	961.4545	7	0.5	481.7347	2	40.77	5	F5:11527	17112021_RID_2349_DP5.raw			

JAA97946.1
back to list

| Protein Coverage | Supporting Peptides |
Protein Coverage:

1 MSMLCYTLII AFLIGIWAAP KSEDNVPLGS PATSDLSDTS YAKTHEALKT SRNTDQHYPA PKKAEDQEFG SAANIIVDPK
81 LFQKRRFQSP RVLFSTQPPP LSRDEQSVDN ANSLNRNIRA KREDHPVHKR GEYSVCDSVN VWVANKTTAT DIRGNLVTVM
161 VDVNINNNVY K**QYFFETK**CR **NPNPVPTGCR** GIDARHWNSY CTTTNTFVK**A LTMEGNQASW R**FIRIDSACV CVISRKNENF
241 G

Supporting Peptides:

Peptide	Uniq	-10lgP	Mass	Length	ppm	m/z	z	RT	Fraction	Scan	Source File	Area F1	Area F2	Area F3
K.ALTMEGNQASWR.F	N	80.22	1362.6350	12	0.4	682.3251	2	38.77	5	F5:10781	17112021_RID_2349_DP5.raw			4.2979E5
K.ALTM(+15.99)EGNQASWR.F	N	77.11	1378.6299	12	0.1	690.3223	2	36.09	5	F5:10910	17112021_RID_2349_DP5.raw			
R.NPNPVPTGC(+57.02)R.G	Y	58.52	1110.5240	10	0.3	556.2695	2	23.20	5	F5:5451	17112021_RID_2349_DP5.raw			
K.QYFFETK.C	N	55.57	961.4545	7	0.5	481.7347	2	40.77	5	F5:11527	17112021_RID_2349_DP5.raw			

BAP39981.1
back to list

| Protein Coverage | Supporting Peptides |
Protein Coverage:

1 NQYRGGAVLQ ATSTDCQLPV VQQFDPDWLQ VDSAFHPSEN LALLENIKVH SVMSMLCYTL IIAFLIGIWA APKSEDNVPL
81 GSPATSDLSD TSCAKTHEAL KTSRNTDQHY PAPKKAEDQE FGSAANIIVD PKLFQKRRFQ SPRVLFSTQP PPLSRDEQSV
161 DNANSLNRNI RSKREDHPVH NRGEYSVCDS VSVWVANKTT ATDIRGNLVT VMVDVNLNNN VYK**QYFFETK** CR**NPNPVPTG**
241 **CR**GIDSRHWN SYCTTTHTFV R**ALTMEGNQA SWR**FIRIDTA CVCVISRKNE NFG

Supporting Peptides:

Peptide	Uniq	-10lgP	Mass	Length	ppm	m/z	z	RT	Fraction	Scan	Source File	Area F1	Area F2	Area F3
R.ALTMEGNQASWR.F	N	80.22	1362.6350	12	0.4	682.3251	2	38.77	5	F5:10781	17112021_RID_2349_DP5.raw			4.2979E5
R.ALTM(+15.99)EGNQASWR.F	N	77.11	1378.6299	12	0.1	690.3223	2	36.09	5	F5:10910	17112021_RID_2349_DP5.raw			
R.NPNPVPTGC(+57.02)R.G	Y	58.52	1110.5240	10	0.3	556.2695	2	23.20	5	F5:5451	17112021_RID_2349_DP5.raw			
K.QYFFETK.C	N	55.57	961.4545	7	0.5	481.7347	2	40.77	5	F5:11527	17112021_RID_2349_DP5.raw			

BAN82142.1
back to list

| Protein Coverage | Supporting Peptides |
Protein Coverage:

1 MGNQYRGGAV LQATSTDCQL PVVQQFDPDW LQVDSAFHPS ENLALLENIK VHSVMSMLCY TLIIAFLIGI WAAPKSEDNV
81 PLGSPATSDL SDTSCAKTYE ALKTSRNTDQ HYPAPKKAED QEFGSAANII VDPKLFQKRR FQSPRVLFST QPPPLSRDEQ
161 SVDNANSLNR NIRAKREDHP VHNRGEYSVC DSVNVWVANK TTATDIRGNL VTVMVDVNLN NNVYK**QYFFE TK**CR**NPNPVP**
241 **TGCR**GIDSRH WNSYCTTTHT FVK**ALTMEGN QASWR**FIRID AACVCVISRK NENFG

Supporting Peptides:

Peptide	Uniq	-10lgP	Mass	Length	ppm	m/z	z	RT	Fraction	Scan	Source File	Area F1	Area F2	Area F3
K.ALTMEGNQASWR.F	N	80.22	1362.6350	12	0.4	682.3251	2	38.77	5	F5:10781	17112021_RID_2349_DP5.raw			4.2979E5
K.ALTM(+15.99)EGNQASWR.F	N	77.11	1378.6299	12	0.1	690.3223	2	36.09	5	F5:10910	17112021_RID_2349_DP5.raw			
R.NPNPVPTGC(+57.02)R.G	Y	58.52	1110.5240	10	0.3	556.2695	2	23.20	5	F5:5451	17112021_RID_2349_DP5.raw			
K.QYFFETK.C	N	55.57	961.4545	7	0.5	481.7347	2	40.77	5	F5:11527	17112021_RID_2349_DP5.raw			

CAP74088.1
back to list

| Protein Coverage | Supporting Peptides |
Protein Coverage:

1 GNVDFDSESP RKPEIQNEII DLHNSLRRSV NPTASNMLKM EWYPEAAANA ERWAFRCILS HSPRDSRVIG GIKCGENIYM

81 STSPMKWTAI IHKWHGEEKD FVYGQGASPA NAVVGHYTQI VWYKSYRSGC AAAYCPSSEY KYFYVCQYCP AGNMQGKTAT

161 PYTSGPPCGD CPSACDNGLC TNPCTHEDKF TNCKDLVKQG CNNNYLKTNC PASCSCHNEI I

Supporting Peptides:

Peptide	Uniq	-10lgP	Mass	Length	ppm	m/z	z	RT	Fraction	Scan	Source File	Area F1	Area F2	Area F3	A
K.MEWYPEAAANAER.W	N	85.56	1536.6667	13	0.5	769.3411	2	45.12	7	F7:13118	17112021_RID_2349_DP7.raw				4
K.M(+15.99)EWYPEAAANAER.W	N	83.36	1552.6616	13	0.4	777.3384	2	40.79	7	F7:11501	17112021_RID_2349_DP7.raw				1
R.SVNPTASNMLK.M	N	53.18	1160.5859	11	0.3	581.3004	2	32.34	4	F4:8661	17112021_RID_2349_DP4.raw				1
R.SVNPTASNM(+15.99)LK.M	N	48.77	1176.5808	11	0.8	589.2982	2	24.27	4	F4:5833	17112021_RID_2349_DP4.raw				9
K.WTAIIHK.W	Y	45.59	867.4966	7	-0.1	434.7556	2	29.95	7	F7:7835	17112021_RID_2349_DP7.raw				3

total 5 pe

ADJ67475.1

back to list

| Protein Coverage | Supporting Peptides |

Protein Coverage:

1 MMQVLLVTIS LAVFPYQGSS IILESGNVND YEVVYPQKVT AMPKGAVKQP EQKYEDTMQY EFEVNGEPVV LHLEKNKILF

81 SEDYSETHYY PDGREITTNP PVEDHCYYHG HIQNDGHSSA SISACNGLKG HFKLRGEMYF IEPLKLSNNE AHAVYKYENI

161 EKEDETPKMC GVTQTNWESD KPIKKASQLV STSAQFNKAF IELIIIVDHS MAKKCNSTAT NTKIYEIVNS ANEIFNPLNI

241 HVTLIGVEFW CDRDLINVTS SADETLDSFG EWRASDLMTR KSHDNALLFT DMRFDLNTLG ITFLAGMCQA YRSVGIVQVQ

321 GNRNFKTAVI MAHELSHNLG MYHDGKNCIC NDSSCVMSPV LSDQPSKLFS NCSIHDYQRY LTRYKPKCIL YPPLRKDIVS

401 PPVCGNEIWE EGEECDCGSP ADCQNPCCDA ATCKLKPGAE CGNGLCCYQC KIKTAGTVCR RARNECDVPE HCTGQSAECP

481 RDQLQQNGQP CQNNRGYCYN GDCPIMRNQC ISLFGSRATV AKDSCFQENL KGSYYGYCRK ENGRKIPCAP QDVKCGRLFC

561 LNNSPRNKNP CNMHYSCMDQ HKGMVDPGTK CEDGKVCNNK RQCVDVNTAY QSTTGFSQI

Supporting Peptides:

Peptide	Uniq	-10lgP	Mass	Length	ppm	m/z	z	RT	Fraction	Scan	Source File	Area F1	Area F2	Area F3	Area F4
R.SVGIVQVQGNR.N	N	67.43	1155.6360	11	0.4	578.8255	2	32.21	1	F1:9249	12112021_RID_2349_DP1.raw	4.3558E5	7.7637E4		
A.SQLVSTSAQFNK.A	Y	53.22	1308.6674	12	1.0	655.3416	2	32.83	7	F7:8869	17112021_RID_2349_DP7.raw				
E.TLDSFGEWR.A	N	52.86	1109.5142	9	0.7	555.7648	2	47.54	2	F2:13529	12112021_RID_2349_DP2.raw		8.0796E4		

total 3 peptides

XP_034278116.1

back to list

| Protein Coverage | Supporting Peptides |

Protein Coverage:

1 MCPRLVTSLL GLVLGINVCA GFTAHRVRRS GEFGEEGPDS VLSDSPWIST YGSCKNRCFE LDEAEPPVCR CDNLCKSYSS

81 CCVDFDELCL KTAGGWECTK ERCGETRNED HACHCSEDCL ARGDCCSNYQ VVCKGDTPWV MDDCEDIRTP VCPAGFLHPP

161 LIIFSVDGFR ASYMKKGEKV MRNIEKLRSC GTHAPYMRPV YPTKTFPNLY TLATGLYPES HGIIGNSMYD PVFDAIFNLR

241 GREKFNHRWW GGQPIWITAA KQGVKAGTFF WSNVIPYERR ILTILQWLTL ADNERVYTRP YAYAFYSDQP DMAGHRYGPF

321 SSEMLGPLRD IDKILGQLMD GLKQLNLHRC ANIIFVGDHG MEDVTCERTE YLSNYLSNVD DITLIPGTLG RIRAKANSNV

401 KFDPKAIVAN LTCKKPDQHF KPYLKQHLPK RLHYANNRRI EDIHLLVERR WHVAKRPGDV FKKITGKCYF HGDHGYDNKI

481 NSMQTVFLGY GPSFKYKTKV PPFENIELYN LMCDLLGLKP APNNGTHGSL NHLLRSSTYR PTMPEEVARP LHPVATTPSS

561 DYDLGCSCDD KNRLDELSRR PYSKGTEART YTGGEGFYTN DPENVLPEDG CVPPPEKHLL YGRPAVLFRI KYSLLHHHDF

641 ESGYSETFQM PLWTSYTISK QAETSGVPDQ VISCVRPDLR ISPSNSQSCT AYRVDRQMSY GFLFPPQLSS SVETKYDAFL

721 ITNVIPMYPA FKKVWNYFQR ILVKRYAIER NGVNVISGPI FDYDFDGLHD TPDKIKLYVE GSSIPVPTHY YSIITSCLDF

801 TQPADKCDGP LSVLAYIFPH RPDNDESCNN SSEDESRWVE ELLKMHTARV RDIEQITGLD FYRKTSRSYS EILSLKTYLH

881 TFESEI

Supporting Peptides:

Peptide	Uniq	-10lgP	Mass	Length	ppm	m/z	z	RT	Fraction	Scan	Source File	Area F1	Area F2	Area F3	A
R.DIEQITGLDFYR.K	Y	62.33	1468.7197	12	1.2	735.3680	2	63.03	3	F3:18811	17112021_RID_2349_DP3.raw			1.9643E5	5

total 5 peptides

Peptide	Uniq	-10lgP	Mass	Length	ppm	m/z	z	RT	Fraction	Scan	Source File	Area F1	Area F2	Area F3	A
R.VRDIEQITGLDFYR.K	Y	47.90	1723.8893	14	-0.3	575.6368	3	54.41	3	F3:16066	17112021_RID_2349_DP3.raw			2.9338E5	
K.VWNYFQR.I	Y	44.68	1011.4926	7	0.6	506.7539	2	44.05	4	F4:12592	17112021_RID_2349_DP4.raw			5.0789E5	7
K.TYLHTFESEI	Y	43.72	1238.5819	10	0.0	620.2982	2	49.94	3	F3:14629	17112021_RID_2349_DP3.raw			1.7866E6	
K.YDAFLITNVIPMYPAFK.K	Y	43.12	2002.0273	17	1.4	1002.0223	2	75.89	3	F3:22897	17112021_RID_2349_DP3.raw			1.7377E5	

total 5 peptides

TRINITY_DN12452
back to list

| Protein Coverage | Supporting Peptides |
Protein Coverage:

1 CLPGWSFYEG NCYKAFDEPK SWVDAEKFCQ KQSNGK**HLAS IEGLGK**ANFV AK**LVSETLRE PQIH**VWIGLR DQSERQQCSS

81 HWTDGSAVSY EKVVRFTKCF GLNKKTDYRT WVGLRCG

Supporting Peptides:

Peptide	Uniq	-10lgP	Mass	Length	ppm	m/z	z	RT	Fraction	Scan	Source File	Area F1	Area F2	Area F3	Area F4	A
K.HLASIEGLGK.A	Y	68.91	1023.5713	10	-0.9	512.7925	2	32.04	7	F7:8674	17112021_RID_2349_DP7.raw			3.0778E5	5.5199E5	2.(
K.LVSETLREPQIH.V	Y	46.38	1420.7673	12	1.1	711.3917	2	35.06	6	F6:9422	17112021_RID_2349_DP6.raw					

total 2 peptides

AUS82489.1
back to list

| Protein Coverage | Supporting Peptides |
Protein Coverage:

1 MVLIRVLANL LILQLSYAQK SSELVVGGDE CNINEHRSLV VLFNSSSFLC AGTLINEEWV LTAAHCDSKN FQMQLGVHSK

81 **KVLNEDEQTR** DPKEKFICPN KKKDDEN**DKD IMLIR**LDSPV SNSEHIAPLS LPSSPPSVGS VCR**IMGWGTI TPTK**ETYPDV

161 PRCANINILD HAVCRAAYPW NPVASTTLCA GTQQGGKDTC RGDSGGPLIC NGQFQGIVSW GGHPCGQAHE PGVYTKVFDY

241 TDWIQSIIAG NTDATCPP

Supporting Peptides:

Peptide	Uniq	-10lgP	Mass	Length	ppm	m/z	z	RT	Fraction	Scan	Source File	Area F1	Area F2	Area F3	Area F
K.VLNEDEQTR.D	N	63.83	1102.5254	9	0.5	552.2703	2	19.87	4	F4:4313	17112021_RID_2349_DP4.raw			2.2851E5	9.6629E
K.KVLNEDEQTR.D	N	55.31	1230.6204	10	0.8	616.3179	2	16.73	4	F4:3273	17112021_RID_2349_DP4.raw			2.4946E4	2.7539E
R.IMGWGTITPTK.E	Y	45.91	1203.6322	11	1.2	602.8241	2	49.08	4	F4:14280	17112021_RID_2349_DP4.raw				1.0911E
N.DKDIMLIR.L	N	44.22	1002.5532	8	-0.5	502.2836	2	37.38	3	F3:10341	17112021_RID_2349_DP3.raw			5.8616E6	
N.DKDIM(+15.99)LIR.L	N	39.37	1018.5481	8	0.3	510.2815	2	31.54	3	F3:8358	17112021_RID_2349_DP3.raw			1.7097E6	

total 5 peptic

JAA98031.1
back to list

| Protein Coverage | Supporting Peptides |
Protein Coverage:

1 MVLIRVLAHL LILQLSYAQK SSELVVGGHP CNINEHRSLV VLFNSSGFLC AGTLINEEWV LTAAHCDSKN FQMQLGVHSK

81 **KVLNEDEQTR** DPKEKFICPN KKKDDEK**DKD IMLIR**LDSPV SNSEHIAPLS LPSSPPSVGS VCR**IMGWGTI TPTK**ETYPDV

161 PHCANINILD HAVCRAAYPW NPVASTTLCA GTQQGGKDTC RADSGGPLIC NGEIQGIVSW GGHPCGQARE PGVYTKVFDY

241 TDWIQSIIAG KKTVNCPP

Supporting Peptides:

Peptide	Uniq	-10lgP	Mass	Length	ppm	m/z	z	RT	Fraction	Scan	Source File	Area F1	Area F2	Area F3	Area F
K.VLNEDEQTR.D	N	63.83	1102.5254	9	0.5	552.2703	2	19.87	4	F4:4313	17112021_RID_2349_DP4.raw			2.2851E5	9.6629E
K.KVLNEDEQTR.D	N	55.31	1230.6204	10	0.8	616.3179	2	16.73	4	F4:3273	17112021_RID_2349_DP4.raw			2.4946E4	2.7539E
R.IMGWGTITPTK.E	Y	45.91	1203.6322	11	1.2	602.8241	2	49.08	4	F4:14280	17112021_RID_2349_DP4.raw				1.0911E
K.DKDIMLIR.L	N	44.22	1002.5532	8	-0.5	502.2836	2	37.38	3	F3:10341	17112021_RID_2349_DP3.raw			5.8616E6	

total 5 peptic

Peptide	Uniq	-10lgP	Mass	Length	ppm	m/z	z	RT	Fraction	Scan	Source File	Area F1	Area F2	Area F3	Area F[illegible]
K.DKDIM(+15.99)LIR.L	N	39.37	1018.5481	8	0.3	510.2815	2	31.54	3	F3:8358	17112021_RID_2349_DP3.raw			1.7097E6	

total 5 pepti[illegible]

TRINITY_DN471
[back to list]

| [Protein Coverage] | [Supporting Peptides] |
Protein Coverage:

```
 1  INVTSAANVT LKSFGEWRER DLLSRKRHDN AQLLTDIDFD GPTIGMAHMF SMCHSERSVG IIQDYSPIDR VVATIMAHEM
81  GHNLGLNHDE KQCNCGANSC IMSAVLSDP
```

Supporting Peptides:

Peptide	Uniq	-10lgP	Mass	Length	ppm	m/z	z	RT	Fraction	Scan	Source File	Area F1	Area F2	Area F3	A[illegible]
R.SVGIIQDYSPIDR.V	Y	83.80	1461.7463	13	0.4	731.8807	2	48.90	2	F2:13984	12112021_RID_2349_DP2.raw	1.3286E6	4.833E6	1.0924E7	2
K.SFGEWRER.D	N	55.07	1065.4991	8	0.1	533.7569	2	31.26	2	F2:7954	12112021_RID_2349_DP2.raw	6.0265E6	2.4122E6		

total 2 peptides

ADN04917.1
[back to list]

| [Protein Coverage] | [Supporting Peptides] |
Protein Coverage:

```
  1  MVLIRVLANL LLLQLSHAQK SSELVVGGDE CNINEHRSLV FLYNSSFGCG GTLINQEWVL SAAHCDMENM RIYLGWHNFS
 81  LPNMNQKRRV AKEKFFCLSS KNYTEWDKDI MLIKMNRPVT YSTHVAPLSL PSSPPSVGSV CRIMGWGAIT SPNETYPDVP
161  HCANINILNY TVCRAAHPWL PAQSRTLCAG ILQGGIDTCK GDSGGPLICN GQIQGIVSWG DNPCAQPLKP GHYTNVFDYT
241  DWIQSIIAGN TTATCPP
```

Supporting Peptides:

Peptide	Uniq	-10lgP	Mass	Length	ppm	m/z	z	RT	Fraction	Scan	Source File	Area F1	Area F2	Area [illegible]
R.AAHPWLPAQSR.T	Y	82.07	1232.6414	11	-1.1	617.3273	2	33.26	1	F1:9553	12112021_RID_2349_DP1.raw	6.4186E7	1.0631E7	3.0723
E.WDKDIMLIK.M	N	57.12	1160.6263	9	-0.1	581.3204	2	46.64	2	F2:13338	12112021_RID_2349_DP2.raw	2.1169E5	9.915E6	1.5104
E.WDKDIM(+15.99)LIK.M	N	52.20	1176.6212	9	-0.2	589.3177	2	41.27	2	F2:11445	12112021_RID_2349_DP2.raw		6.6323E6	2.1157

total 3 pe[illegible]

E0Y419.1
[back to list]

| [Protein Coverage] | [Supporting Peptides] |
Protein Coverage:

```
  1  MVLIRVLANL LLLQLSHAQK SSELVVGGDE CNINEHRSLV FLYNSSFGCG GTLINQEWVL SAAHCDMENM RIYLGWHNFS
 81  LPNMNQKRRV AKEKFFCLSS KNYTEWDKDI MLIKMNRPVT YSTHVAPLSL PSSPPSVGSV CRIMGWGAIT SPNETYPDVP
161  HCANINILNY TVCRAAHPWL PAQSRTLCAG ILQGGIDTCK GDSGGPLICN GQIQGIVSWG DNPCAQPLKP GHYTNVFDYT
241  DWIQSIIAGN TTATCPP
```

Supporting Peptides:

Peptide	Uniq	-10lgP	Mass	Length	ppm	m/z	z	RT	Fraction	Scan	Source File	Area F1	Area F2	Area [illegible]
R.AAHPWLPAQSR.T	Y	82.07	1232.6414	11	-1.1	617.3273	2	33.26	1	F1:9553	12112021_RID_2349_DP1.raw	6.4186E7	1.0631E7	3.0723
E.WDKDIMLIK.M	N	57.12	1160.6263	9	-0.1	581.3204	2	46.64	2	F2:13338	12112021_RID_2349_DP2.raw	2.1169E5	9.915E6	1.5104
E.WDKDIM(+15.99)LIK.M	N	52.20	1176.6212	9	-0.2	589.3177	2	41.27	2	F2:11445	12112021_RID_2349_DP2.raw		6.6323E6	2.1157

total 3 pe[illegible]

AEH59582.1
[back to list]

| [Protein Coverage] | [Supporting Peptides] |
Protein Coverage:

```
1   MSMLCYTLII AFLIGIWAAP KSEDNVSLGS LATPDLSDTS CAKTHEALKT SRNTDQHYPA PKKAEDQEFG SAANIIVDPK
81  LFQKRRFQSP RVLFSTQPPP LSRDEQSMEF LDNADSLNRN IRAKRGTHPV HNRGEFSVCD SVSVWVANKT TATDIRGNVV
161 TVMVDVNLNN NVYRQYFFET KCKNPNPVPS GCRGIDAKHW NSYCTTTDTF VRALTMEGNQ ASWRFIRIDT ACVCVISRKN
241 DNL
```

Supporting Peptides:

Peptide	Uniq	-10lgP	Mass	Length	ppm	m/z	z	RT	Fraction	Scan	Source File	Area F1	Area F2	Area F3
R.ALTMEGNQASWR.F	N	80.22	1362.6350	12	0.4	682.3251	2	38.77	5	F5:10781	17112021_RID_2349_DP5.raw			4.2979E5
R.ALTM(+15.99)EGNQASWR.F	N	77.11	1378.6299	12	0.1	690.3223	2	36.09	5	F5:10910	17112021_RID_2349_DP5.raw			
R.QYFFETK.C	N	55.57	961.4545	7	0.5	481.7347	2	40.77	5	F5:11527	17112021_RID_2349_DP5.raw			
M.VDVNLNNNVYR.Q	Y	38.71	1318.6630	11	1.1	660.3395	2	37.96	5	F5:10607	17112021_RID_2349_DP5.raw			

total 4

QBF53420.1
back to list

| Protein Coverage | Supporting Peptides |
Protein Coverage:

```
1   MVHSVMSMLC YTLIIAFLIG IWAAPKSEDN VSLGSPATPD LSDTSCAKTH EALKTSRNTD QHYPAPKKAE DQEFGSAANI
81  IVDPKLFQKR RFQSPRVLFS TQPPPLSRDE QSVEFLDSAD SLNRNIRTKR GTHPVHNRGE FSVCDSVSVW VANKTTATDI
161 RGNVVTVMVD VNLNNNVYRQ YFFETKCKNP NPVPSGCRGI DAKHWNSYCT TTDTFVRALT MEGNQASWRF IRIDTACV
```

Supporting Peptides:

Peptide	Uniq	-10lgP	Mass	Length	ppm	m/z	z	RT	Fraction	Scan	Source File	Area F1	Area F2	Area F3
R.ALTMEGNQASWR.F	N	80.22	1362.6350	12	0.4	682.3251	2	38.77	5	F5:10781	17112021_RID_2349_DP5.raw			4.2979E5
R.ALTM(+15.99)EGNQASWR.F	N	77.11	1378.6299	12	0.1	690.3223	2	36.09	5	F5:10910	17112021_RID_2349_DP5.raw			
R.QYFFETK.C	N	55.57	961.4545	7	0.5	481.7347	2	40.77	5	F5:11527	17112021_RID_2349_DP5.raw			
M.VDVNLNNNVYR.Q	Y	38.71	1318.6630	11	1.1	660.3395	2	37.96	5	F5:10607	17112021_RID_2349_DP5.raw			

total 4

QHR82782.1
back to list

| Protein Coverage | Supporting Peptides |
Protein Coverage:

```
1   MSMLCYTLII AFLIGIWAAP KSEDNVSLGS PATPDLSDTS CAKTHEALKT SRNTDQHYPA PKKAEDQEFG SAANIIVDPK
81  LFQKRRFQSP RVLFSTQPPP LSRDEQSMEF LDNADSLNRN IRAKRGTHPV HNRGEFSVCD SVSVWVANKT TATDIRGNVV
161 TVMVDVNLNN NVYRQYFFET KCKNPNPVPS GCRGIDAKHW NSYCTTTDTF VRALTMEGNQ ASWRFIRIDT ACVCVISRKN
241 DNLG
```

Supporting Peptides:

Peptide	Uniq	-10lgP	Mass	Length	ppm	m/z	z	RT	Fraction	Scan	Source File	Area F1	Area F2	Area F3
R.ALTMEGNQASWR.F	N	80.22	1362.6350	12	0.4	682.3251	2	38.77	5	F5:10781	17112021_RID_2349_DP5.raw			4.2979E5
R.ALTM(+15.99)EGNQASWR.F	N	77.11	1378.6299	12	0.1	690.3223	2	36.09	5	F5:10910	17112021_RID_2349_DP5.raw			
R.QYFFETK.C	N	55.57	961.4545	7	0.5	481.7347	2	40.77	5	F5:11527	17112021_RID_2349_DP5.raw			
M.VDVNLNNNVYR.Q	Y	38.71	1318.6630	11	1.1	660.3395	2	37.96	5	F5:10607	17112021_RID_2349_DP5.raw			

total 4

TRINITY_DN1356
back to list

| Protein Coverage | Supporting Peptides |
Protein Coverage:

1 MLIRWAGGWG QLGKAYVKRW EAIAPCANFV CCQFAVSVTE RLPERSFIKA SHLSTLLLVS CWSQGMVHSV MSMLCYTLII
81 AFLIGIWAAP KSEDNVSLGS PATPDLSDTS CAKTHEALKT SRNTDQHYPA PKKAEDQEFG SAANIIVDPK LFQKRRFQSP
161 RVLFSTQPPP LSRDEQSLEF LDNADSLNRN IRAKRETHPV HNRGEFSVCD SVSVWVANKT TATDIRGNVV TVM**VDVNLNN**
241 **NVYRQYFFET K**CKNPNPVPS GCRGIDAKHW NSYCTTTDTF VR**ALTMEGNQ ASWR**FIRIDT ACVCVISRKK

Supporting Peptides:

Peptide	Uniq	-10lgP	Mass	Length	ppm	m/z	z	RT	Fraction	Scan	Source File	Area F1	Area F2	Area F3
R.ALTMEGNQASWR.F	N	80.22	1362.6350	12	0.4	682.3251	2	38.77	5	F5:10781	17112021_RID_2349_DP5.raw			4.2979E5
R.ALTM(+15.99)EGNQASWR.F	N	77.11	1378.6299	12	0.1	690.3223	2	36.09	5	F5:10910	17112021_RID_2349_DP5.raw			
R.QYFFETK.C	N	55.57	961.4545	7	0.5	481.7347	2	40.77	5	F5:11527	17112021_RID_2349_DP5.raw			
M.VDVNLNNNVYR.Q	Y	38.71	1318.6630	11	1.1	660.3395	2	37.96	5	F5:10607	17112021_RID_2349_DP5.raw			

total 4

TRINITY_DN1356
back to list

| Protein Coverage | Supporting Peptides |
Protein Coverage:

1 MLIRWAGGWG QLGKAYVKRW EAIAPCANFV CCQFAVSVTE RLPERSFIKA SHLSTLLLVS CWSQGMVHSV MSMLCYTLII
81 AFLIGIWAAP KSEDNVSLGS PATPDLSDTS CAKTHEALKT SRNTDQHYPA PKKAEDQEFG SAANIIVDPK LFQKRRFQSP
161 RVLFSTQPPP LSRDEQSLEF LDNADSLNRN IRAKRETHPV HNRGEFSVCD SVSVWVANKT TATDIRGNVV TVM**VDVNLNN**
241 **NVYRQYFFET K**CKNPNPVPS GCRGIDAKHW NSYCTTTDTF VR**ALTMEGNQ ASWR**FIRIDT ACVCVISRKK

Supporting Peptides:

Peptide	Uniq	-10lgP	Mass	Length	ppm	m/z	z	RT	Fraction	Scan	Source File	Area F1	Area F2	Area F3
R.ALTMEGNQASWR.F	N	80.22	1362.6350	12	0.4	682.3251	2	38.77	5	F5:10781	17112021_RID_2349_DP5.raw			4.2979E5
R.ALTM(+15.99)EGNQASWR.F	N	77.11	1378.6299	12	0.1	690.3223	2	36.09	5	F5:10910	17112021_RID_2349_DP5.raw			
R.QYFFETK.C	N	55.57	961.4545	7	0.5	481.7347	2	40.77	5	F5:11527	17112021_RID_2349_DP5.raw			
M.VDVNLNNNVYR.Q	Y	38.71	1318.6630	11	1.1	660.3395	2	37.96	5	F5:10607	17112021_RID_2349_DP5.raw			

total 4

TRINITY_DN1356
back to list

| Protein Coverage | Supporting Peptides |
Protein Coverage:

1 MLIRWAGGWG QLGKAYVKRW EAIAPCANFV CCQFAVSVTE RLPERSFIKA SHLSTLLLVS CWSQGMVHSV MSMLCYTLII
81 AFLIGIWAAP KSEDNVSLGS PATPDLSDTS CAKTHEALKT SRNTDQHYPA PKKAEDQEFG SAANIIVDPK LFQKRRFQSP
161 RVLFSTQPPP LSRDEQSLEF LDNADSLNRN IRAKRETHPV HNRGEFSVCD SVSVWVANKT TATDIRGNVV TVM**VDVNLNN**
241 **NVYRQYFFET K**CKNPNPVPS GCRGIDAKHW NSYCTTTDTF VR**ALTMEGNQ ASWR**FIRIDT ACVCVISRKK

Supporting Peptides:

Peptide	Uniq	-10lgP	Mass	Length	ppm	m/z	z	RT	Fraction	Scan	Source File	Area F1	Area F2	Area F3
R.ALTMEGNQASWR.F	N	80.22	1362.6350	12	0.4	682.3251	2	38.77	5	F5:10781	17112021_RID_2349_DP5.raw			4.2979E5
R.ALTM(+15.99)EGNQASWR.F	N	77.11	1378.6299	12	0.1	690.3223	2	36.09	5	F5:10910	17112021_RID_2349_DP5.raw			
R.QYFFETK.C	N	55.57	961.4545	7	0.5	481.7347	2	40.77	5	F5:11527	17112021_RID_2349_DP5.raw			
M.VDVNLNNNVYR.Q	Y	38.71	1318.6630	11	1.1	660.3395	2	37.96	5	F5:10607	17112021_RID_2349_DP5.raw			

total 4

TRINITY_DN1356
back to list

| Protein Coverage | Supporting Peptides |
Protein Coverage:

1 MLIRWAGGWG QLGKAYVKRW EAIAPCANFV CCQFAVSVTE RLPERSFIKA SHLSTLLLVS CWSQGMVHSV MSMLCYTLII
81 AFLIGIWAAP KSEDNVSLGS PATPDLSDTS CAKTHEALKT SRNTDQHYPA PKKAEDQEFG SAANIIVDPK LFQKRRFQSP
161 RVLFSTQPPP LSRDEQSLEF LDNADSLNRN IRAKRETHPV HNRGEFSVCD SVSVWVANKT TATDIRGNVV TVM**VDVNLNN**
241 **NVYRQYFFET K**CKNPNPVPS GCRGIDAKHW NSYCTTTDTF VR**ALTMEGNQ ASWR**FIRIDT ACVCVISRKK

Supporting Peptides:

Peptide	Uniq	-10lgP	Mass	Length	ppm	m/z	z	RT	Fraction	Scan	Source File	Area F1	Area F2	Area F3
R.ALTMEGNQASWR.F	N	80.22	1362.6350	12	0.4	682.3251	2	38.77	5	F5:10781	17112021_RID_2349_DP5.raw			4.2979E5
R.ALTM(+15.99)EGNQASWR.F	N	77.11	1378.6299	12	0.1	690.3223	2	36.09	5	F5:10910	17112021_RID_2349_DP5.raw			
R.QYFFETK.C	N	55.57	961.4545	7	0.5	481.7347	2	40.77	5	F5:11527	17112021_RID_2349_DP5.raw			
M.VDVNLNNNVYR.Q	Y	38.71	1318.6630	11	1.1	660.3395	2	37.96	5	F5:10607	17112021_RID_2349_DP5.raw			

total 4

TRINITY_DN180
back to list

| Protein Coverage | Supporting Peptides |
Protein Coverage:

1 MSMNGSHPRL NTLGRMVRAE SGPDMRYDMN SHMMMGGGGG GGGGSGHTHK MYYVQKSYVG DVNSEGYTQN GTATVSRRQN
81 TIQDLLQVCS DCLMR**AELIV QPELK**YGDGI QISRNRELEE CFTQATEHME MLEALIREMR QMGQPCELYQ KRLLQLQEQM
161 RALYKAISVP RVRRASSKGG GYSSQSASGW DEYTRRVTSE CLNWMRQQKT EMELIKWGFD AAAIEQQIAE HRRFHNSIGD
241 YRWHLDKVKT DLREKAAVHQ LEEEYEYLLR FSFERMDQLR **QLQNIIQATS R**EIMWINDCE EEELLYDWSD RNTDIARKQE
321 AFSKRMSQLE VKEKELNKLK QECDQLVLSQ HPASDKIEAY MDTLQTQWSW ILQITKCIDV HLKENAAYFQ FFEEAQSTEN
401 YLKNLQDVIR KRFICDKNMS LQVLLEQIKE LEAEREKILE YKRQVQNLVN KSKKIVQLKP RNPEYK**SNKP IILR**ALCDYK
481 QDLKTMRKGD ECILKDNSER SKWHVTGPGG LDMLVPSVSL IIPPPNPLAV DLATKIEQYY DAIMALWNQL YINMKSLVSW
561 HYCLIDIEKI KVMTIAKLKT MRREDYQRII SDLEIHYQEF IRNSQGSEMF GDEDKRTMQT RITDAQKHYQ TLVIQLPDLQ
641 TQPLPPQPTI VTEVINIEKQ EAWLLMELQK LRRQIENCEL RMVQRTPFQL DQGATHNLCV RISELESIQN ESQSLAEILN
721 RQNDYLPNFR GSEKYHYLQS EISALFQKLE NINGISACYL DSLNALRSLL QAILQTDDMI RVFEIRLTEE DTLSLDPDKV
801 EAYRACLKKM KADLSMKKSL LGTLEAELQK ALQVHSQSSQ TYPHYELDLG KFADRVSQLT DRWQRLEKQL DDRSWDLEKQ
881 VKQLRIYRDL YQALNKWICD ARRRQETIEA MKLGDVSTVM RYLQEQKNLH SEVTSKRDRV EEVIKHAEVC SLAIKDYELQ
961 AAAYSSGLET LLNIPVKRSM VQSPSGLILQ EAGEIQSRYI ELLTRSGDYY KFLSEMLKSL EDIKMKSTRI ELLEEELRLA
1041 KDANSDSNNK HKFLEQNLQK YQVECSQLKA KFISLEEMKR QVEMDSSTSK QNLDKCYAQI KDLNDRITRL TYEIEDEKRK
1121 RKLLEDRYEQ QKADFDQLQK TRQNEKETLG WQKIESEKII KEKEYELERL RVLLQDEGTR KRECENELAK VRNQFNEEMS
1201 NIKNKYETEI NIKKTTIHQI AAQKEDDAKN LRTQIDRLQR ENRDLKDEIV RLNEAILDTT EQRRRAEENI LQQKACGSEV
1281 LQQKQQIELE LKQIIQLRND DNTRYKQALE DATSTIHEKV KEIERLKIQL KEEAKSRWEL ENELAKTSHR IQESKNQCNH
1361 VMQERETLLI KIKTLEQDKA RLLRLEEDLN RTKGMLESES RLKVRLENEK QQILNDLNQW KSQYSRKEET IRKTESERDK
1441 SEREKSALLI EIERLQAEIK RIEERYRCRL EETSRKSQAE METERLRLQR EIEKLKQRPK GAPRGTQTEE DLMIDPSKLL
1521 FSGLRKKITA VQLYECQLID KCTLDKLLRG QKSVEEVAAE LEPYLRGAGA IAGASLNTKV KFSLVEAKRK QLLTPENTVL
1601 LLEAQAATGG VIDPHRNELL SVDSAVARDL IDFDDREQIY MAEKAITGFK DPFSGKMMPV AEAMKKNLVD RETGLRLLEA
1681 QLASGGIVDP INSIFLPKDI ALSRGLIDRD LYRALNNPSK PLIDPLSKVA ISYMKLREKC RVEQHTGLLL LPVQKRNTSF
1761 QGIRKQISVS ELVESGIIRE STANELETGA ITVEEVTDRI KDFLQGSSCI AGIYNEATGE KLGVYQAMKI GLVRPGTALE
1841 LLEAQAATGF IVDPVNNLRL PVEEAYKRGL VGIEFKEKLL SAERAVTGYK DPETGNIISL FQAMNKELIE KGHGIR**LLEA**
1921 **QIATGGIIDP K**ESHRLPVHT AYQRGYFNEE LNDILSDPSD DTKGFFDPNT EENLTYLQLK GRCKIDESTR LCLLPLREKK
2001 KVVQTSQKNT LRKRRVVIVD PDTNKEMSVQ EAYNKGLIDY STYMELSDQE CEWEEITTTG SDGSTRVVLV DRKTGHQYDI
2081 EDALDKGLVD RKFFDQYRSG SLSLTQFADM IASRNGGDEV FRQESIIRSP TVVSVKSSSI LLKSSSGSFS DSPEETTPIA
2161 AIFDTENLEK ISISEAVQRG IVDTITAQRL LEAQTCTGGI LCPTTGQRLS LQDAVAQGII DHEMATRLKP AQKAFLGFEG
2241 IKGRKRMSAA EAVKEKWLPY EAGQRFLEFQ FITGGLIDPE VHGRISTEEA IRKGLIDGRT SQRLQDISNY AKILTCPKTK
2321 LKISYREAMN RSMVEDQTGL KLLEASSVSS KGISSPYNVS SAPGSRSGSR SGSRSGSRSG SRRGSFDAGT SSSYSYSYTA
2401 VSSNSSLGR

Supporting Peptides:

Peptide	Uniq	-10lgP	Mass	Length	ppm	m/z	z	RT	Fraction	Scan	Source File	Area F1	Area F2	Area F3	Area [illegible]
R.LLEAQIATGGIIDPK.E	Y	62.51	1537.8715	15	0.8	769.9436	2	51.54	6	F6:15197	17112021_RID_2349_DP6.raw				
R.QLQNIIQATSR.E	Y	51.48	1270.6993	11	0.2	636.3571	2	38.60	2	F2:10594	12112021_RID_2349_DP2.raw		1.0785E4		1.2228[illegible]

total 4 peptides

Peptide	Uniq	-10lgP	Mass	Length	ppm	m/z	z	RT	Fraction	Scan	Source File	Area F1	Area F2	Area F3	Area I
K.SNKPIILR.A	Y	44.33	939.5865	8	-0.1	470.8005	2	24.97	6	F6:6034	17112021_RID_2349_DP6.raw				
R.AELIVQPELK.Y	Y	42.71	1138.6597	10	-0.2	570.3370	2	43.74	6	F6:12471	17112021_RID_2349_DP6.raw				

total 4 peptides

TRINITY_DN180
back to list

| Protein Coverage | Supporting Peptides |
Protein Coverage:

```
   1 MSMNGSHPRL NTLGRMVRAE SGPDMRYDMN SHMMMGGGGG GGGGSGHTHK MYYVQKSYVG DVNSEGYTQN GTATVSRRQN
  81 TIQDLLQVCS DCLMRAELIV QPELKYGDGI QISRNRELEE CFTQATEHME MLEALIREMR QMGQPCELYQ KRLLQLQEQM
 161 RALYKAISVP RVRRASSKGG GYSSQSASGW DEYTRRVTSE CLNWMRQQKT EMELIKWGFD AAAIEQQIAE HRRFHNSIGD
 241 YRWHLDKVKT DLREKAAVHQ LEEEYEYLLR FSFERMDQLR QLQNIIQATS REIMWINDCE EEELLYDWSD RNTDIARKQE
 321 AFSKRMSQLE VKEKELNKLK QECDQLVLSQ HPASDKIEAY MDTLQTQWSW ILQITKCIDV HLKENAAYFQ FFEEAQSTEN
 401 YLKNLQDVIR KRFICDKNMS LQVLLEQIKE LEAEREKILE YKRQVQNLVN KSKKIVQLKP RNPEYKSNKP IILRALCDYK
 481 QDLKTMRKGD ECILKDNSER SKWHVTGPGG LDMLVPSVSL IIPPPNPLAV DLATKIEQYY DAIMALWNQL YINMKSLVSW
 561 HYCLIDIEKI KVMTIAKLKT MRREDYQRII SDLEIHYQEF IRNSQGSEMF GDEDKRTMQT RITDAQKHYQ TLVIQLPDLQ
 641 TQPLPPQPTI VTEVINIEKQ EAWLLMELQK LRRQIENCEL RMVQRTPFQL DQGATHNLCV RISELESIQN ESQSLAEILN
 721 RQNDYLPNFR GSEKYHYLQS EISALFQKLE NINGISACYL DSLNALRSLL QAILQTDDMI RVFEIRLTEE DTLSLDPDKV
 801 EAYRACLKKM KADLSMKKSL LGTLEAELQK ALQVHSQSSQ TYPHYELDLG KFADRVSQLT DRWQRLEKQL DDRSWDLEKQ
 881 VKQLRIYRDL YQALNKWICD ARRRQETIEA MKLGDVSTVM RYLQEQKNLH SEVTSKRDRV EEVIKHAEVC SLAIKDYELQ
 961 AAAYSSGLET LLNIPVKRSM VQSPSGLILQ EAGEIQSRYI ELLTRSGDYY KFLSEMLKSL EDIKMKSTRI ELLEEELRLA
1041 KDANSDSNNK HKFLEQNLQK YQVECSQLKA KFISLEEMKR QVEMDSSTSK QNLDKCYAQI KDLNDRITRL TYEIEDEKRK
1121 RKLLEDRYEQ QKADFDQLQK TRQNEKETLG WQKIESEKII KEKEYELERL RVLLQDEGTR KRECENELAK VRNQFNEEMS
1201 NIKNKYETEI NIKKTTIHQI AAQKEDDAKN LRTQIDRLQR ENRDLKDEIV RLNEAILDTT EQRRRAEENI LQQKACGSEV
1281 LQQKQQIELE LKQIIQLRND DNTRYKQALE DATSTIHEKV KEIERLKIQL KEEAKSRWEL ENELAKVRNS YDEEIISLKN
1361 KYETEINITK TTINQVTVKK EEEASNYRAQ LDNAVRENRS LCEELRRLKN TLSQTTENLR KVEESVHQQK SIGSEISQKK
1441 QQLEKELKQT VQKFTEEIAH YKQSLDEAGR TIKEKNKEIE KNRKLLDAEI SQRKSMEEEN IHLQRIQYDL EKANINAKET
1521 ISRLKVQEQE LGRLKSEYER LAQDKKGKDQ ESTRLQSTVK ELQHQKHMLE EELNRQSRIV ADETSKRKNA EEELEAMRRT
1601 LRDQAVKITN LTQQIEEISI VKKRSEDDLR HQREVLDNHM REKQRYMEEI RKYTSEVESL RRQMVQEQEN LKQAHIRNEH
1681 LQKAIEDKSK SLNECKIEIE RLQCLTENLT KEHLMLEEEL RNLRLEYDDL RRGRSEVDEE KNAAILDLKN QLQTSNKHAL
1761 ELQGVINGLQ KERENLRQEI EKFQKQALET SHRIQESKNQ CNHVMQERET LLIKIKTLEQ DKARLLRLEE DLNRTKGMLE
1841 SESRLKVRLE NEKQQILNDL NQWKSQYSRK EETIRKTESE RDKSEREKSA LLIEIERLQA EIKRIEERYR CRLEETSRKS
1921 QAEMETERLR LQREIEKLKQ RPKGAPRGTQ TEEDLMIDPS KLLFSGLRKK ITAVQLYECQ LIDKCTLDKL LRGQKSVEEV
2001 AAELEPYLRG AGAIAGASLN TKVKFSLVEA KRKQLLTPEN TVLLLEAQAA TGGVIDPHRN ELLSVDSAVA RDLIDFDDRE
2081 QIYMAEKAIT GFKDPFSGKM MPVAEAMKKN LVDRETGLRL LEAQLASGGI VDPINSIFLP KDIALSRGLI DRDLYRALNN
2161 PSKPLIDPLS KVAISYMKLR EKCRVEQHTG LLLLPVQKRN TSFQGIRKQI SVSELVESGI IRESTANELE TGAITVEEVT
2241 DRIKDFLQGS SCIAGIYNEA TGEKLGVYQA MKIGLVRPGT ALELLEAQAA TGFIVDPVNN LRLPVEEAYK RGLVGIEFKE
2321 KLLSAERAVT GYKDPETGNI ISLFQAMNKE LIEKGHGIRL LEAQIATGGI IDPKESHRLP VHTAYQRGYF NEELNDILSD
2401 PSDDTKGFFD PNTEENLTYL QLKGRCKIDE STRLCLLPLR EKKKVVQTSQ KNTLRKRRVV IVDPDTNKEM SVQEAYNKGL
2481 IDYSTYMELS DQECEWEEIT TTGSDGSTRV VLVDRKTGHQ YDIEDALDKG LVDRKFFDQY RSGSLSLTQF ADMIASRNGG
2561 DEVFRQESII RSPTVVSVKS SSILLKSSSG SFSDSPEETT PIAAIFDTEN LEKISISEAV QRGIVDTITA QRLLEAQTCT
2641 GGILCPTTGQ RLSLQDAVAQ GIIDHEMATR LKPAQKAFLG FEGIKGRKRM SAAEAVKEKW LPYEAGQRFL EFQFITGGLI
2721 DPEVHGRIST EEAIRKGLID GRTSQRLQDI SNYAKILTCP KTKLKISYRE AMNRSMVEDQ TGLKLLEASS VSSKGISSPY
2801 NVSSAPGSRS GSRSGSRSGS RSGSRRGSFD AGTSSSYSYS YTAVSSNSSL GR
```

Supporting Peptides:

Peptide	Uniq	-10lgP	Mass	Length	ppm	m/z	z	RT	Fraction	Scan	Source File	Area F1	Area F2	Area F3	Area I
R.LLEAQIATGGIIDPK.E	Y	62.51	1537.8715	15	0.8	769.9436	2	51.54	6	F6:15197	17112021_RID_2349_DP6.raw				
R.QLQNIIQATSR.E	Y	51.48	1270.6993	11	0.2	636.3571	2	38.60	2	F2:10594	12112021_RID_2349_DP2.raw		1.0785E4		1.2228I
K.SNKPIILR.A	Y	44.33	939.5865	8	-0.1	470.8005	2	24.97	6	F6:6034	17112021_RID_2349_DP6.raw				
R.AELIVQPELK.Y	Y	42.71	1138.6597	10	-0.2	570.3370	2	43.74	6	F6:12471	17112021_RID_2349_DP6.raw				

total 4 peptides

XP_034285002.1
back to list

| Protein Coverage | Supporting Peptides |

Protein Coverage:

```
   1 MSINGSHPRL NTLGRMVRSE SGPDMRYDMN SHMMMGGGGG GGGGGGSSHA HKMYYVQKCY VGDINSEGYT QNGTATVSRR
  81 QNTIQDLLQA CSDCLMRAEL IVQPELKYGD GIQISRNREL EDCFGQANEH MDILDGLIRE MRQMGQPCDL YQKRLLQLQE
 161 QMRALYKAIS VPRVRRASSK GGGYSSQSAS GWDEYTRRVT SECLNWMRQQ KTEMELVKWG FDAAAIEQQI AEHRRFHNSI
 241 GDYRWHLDKV KTDLREKAAV HQLEEEYEYL LRFSFERMDQ LRQLQNIIQA TSREIMWIND CEEEELLYDW SDRNTDIARK
 321 QEVFSKRMSQ LEVKEKELNK LKQECDQLVL SQHPASDKIE AYMDTLQTQW SWILQITKCI DVHLKENAAY FQFFEEAHST
 401 ENYLKNLQDA IRKRFICDKN MSLQVLLEQI KELEAEREKI LEYKRQVQNL VNKSKKIVQL KPRNPEYKSN KPIILRALCD
 481 YKQDLKTMRK GDECILKDNS ERSKWHVTGP GGLDMLVPSV SLIIPPPNPL AVDLATKIEQ YYDAIMALWN QLYINMKSLV
 561 SWHYCMIDVE KIRAMTIAKL KTMRREDYQR IIADLEIHYQ EFIRNSQGSE MFGDDDKRNM QTQITDAQKH YQTLVMTLPR
 641 QTQTGPPIVT EVITIEKEKQ EAWLLMELQK LRRQIENCEF RMVQRTPFQL DQGASHNLSV RISELESIQI ESQSMAEALN
 721 RQKDYLPNFR GSEKYHYLQS EISALFQKLE NINGVSACYL DSLNALRSLL QAILQTDDVI RVFEIRLTEE DTLSLDPDKV
 801 EGYRVCLKKM KADLTMKKSL LGTLEAELQK ALQVHSQSSQ TYPHYDLDLG KFADRVSQLR DRWQRLEKQL DDRSWDLEKQ
 881 VKQLRIYREL YQALNKWICD ARRRQDTIEA MKLGDVSTVM RYLQEQKNLH SEITSKRDRV EDVVKNAEVC SLAIKDYELQ
 961 AAAYSSGLET LLNIPVKRSM VQSPSGLILQ EAGEIQSRYI ELLTRSGDYY KFLSEMLKSL EDIKMKSTRI ELLEEELRLA
1041 KDANSDSNNK HKFLEQNLQK YQVECSQLKA KFISLEEMKR QVEMDGSTAK QNLDKCYAQI KDLNERITRL TYEIEDEKRK
1121 RKLLEDRYDQ QKSDFDQLQK TRHNEKETLG WQKIESEKII KEKEYEIERL RVLLQDEGTR KRECENELAK TSHRIQESKS
1201 QCNHIMQERE TLLVKIKTLE QDKARLLRLE EDLNRTKGML ESESRLKARL ENEKQQILND LNQWKSQYSR KEETIRKTEC
1281 ERDKSEREKS SLLIEIERLQ AEIKRIEERY RCRLEETSRK SQAEMETERS RWQKEIDKLK KRPYGAHRST QTEEDLMIDP
1361 SKLLFNGLRK KITAVQLYEC QLIDKCTLDK LLRGQKSVEE VAAELEPYLK GAGAIAGASL NTKEKYSLVE AKRKQLLTPE
1441 NTILLLEAQA ATGGVIDPHR NELLSVDSAV ARDLVDFDDR EQIYMAEKAI TGFKDPFSGK TMSVAEAIKK NLVDRETGMR
1521 LLEAQLASGG IVDPINSIFL PKDIALSRGL IDRDLYRALN NPSKPLIDPL SKNAISYMKL RERCRIEPHT GLLLLPVQKR
1601 SMSFQGIRKL ITVSELVDSG IIRESTANEL ETGAITVEEV TDRIKDFLQG SSCIAGIYNE ATGEKLGVYQ AMKIGLVRPG
1681 TALELLEAQA ATGFIVDPVN NLRLPVEEGY KRGLVGIEFK EKLLSAERAV TGYRDPETGN IISLFQAMNK ELIEKGHGVR
1761 LLEAQIATGG IIDPKESHRL PVHTAYQRGY FNEELNDILS DPSDDTKGFF DPNTEENLTY LQLKGRCKID ELTGLCLLPL
1841 REKKKVVQTS QKNTLRKRRV VIVDPDTSKE MSVQEAYNKG LIDYSTYMEL SDQECEWEEI TTTGSDGSTR VVLVDRKTGH
1921 QYDIEDAIEK GLVDRKFFEQ YRSGSLSLTQ FADMIASRNG GDEVFKHESI IRSPTVLSVK SSSILLKSSS GSFSDSPEEA
2001 TPIAAIFDTE NLEKISIPEA VQRGIVDTIT AQRLLEAQSC TGGIICPTTG QRLSLQDAVA QGIIDHEMAT RLKPAQKAFL
2081 GFEGIKGRKR MSAAEAVKEK WLPYEAGQRF LEFQFITGGL VDPEVHGRIS TEEAIRKGLI DGRTSQRLQD IGNYAKILTC
2161 PKTKLKISYR EAMNRSMVED QTGLRLLEAS SVSSKGISSP YNVSSAPGSR SGSRSGSRSG SRSGSRRGSF DAGTSSSYSY
2241 SYTTVSSGSL GR
```

Supporting Peptides:

Peptide	Uniq	-10lgP	Mass	Length	ppm	m/z	z	RT	Fraction	Scan	Source File	Area F1	Area F2	Area F3	Area I
R.LLEAQIATGGIIDPK.E	Y	62.51	1537.8715	15	0.8	769.9436	2	51.54	6	F6:15197	17112021_RID_2349_DP6.raw				
R.QLQNIIQATSR.E	Y	51.48	1270.6993	11	0.2	636.3571	2	38.60	2	F2:10594	12112021_RID_2349_DP2.raw		1.0785E4		1.2228[illegible]
K.SNKPIILR.A	Y	44.33	939.5865	8	-0.1	470.8005	2	24.97	6	F6:6034	17112021_RID_2349_DP6.raw				
R.AELIVQPELK.Y	Y	42.71	1138.6597	10	-0.2	570.3370	2	43.74	6	F6:12471	17112021_RID_2349_DP6.raw				

total 4 peptides

XP_034285001.1

back to list

| Protein Coverage | Supporting Peptides |

Protein Coverage:

1 MSINGSHPRL NTLGRMVRSE SGPDMRYDMN SHMMMGGGGG GGGGGGSSHA HKMYYVQKCY VGDINSEGYT QNGTATVSRR
81 QNTIQDLLQA CSDCLMR**AEL IVQPELK**YGD GIQISRNREL EDCFGQANEH MDILDGLIRE MRQMGQPCDL YQKRLLQLQE
161 QMRALYKAIS VPRVRRASSK GGGYSSQSAS GWDEYTRRVT SECLNWMRQQ KTEMELVKWG FDAAAIEQQI AEHRRFHNSI
241 GDYRWHLDKV KTDLREKAAV HQLEEEYEYL LRFSFERMDQ LR**QLQNIIQA TSR**EIMWIND CEEEELLYDW SDRNTDIARK
321 QEVFSKRMSQ LEVKEKELNK LKQECDQLVL SQHPASDKIE AYMDTLQTQW SWILQITKCI DVHLKENAAY FQFFEEAHST
401 ENYLKNLQDA IRKRFICDKN MSLQVLLEQI KELEAEREKI LEYKRQVQNL VNKSKKIVQL KPRNPEYK**SN KPIILR**ALCD
481 YKQDLKTMRK GDECILKDNS ERSKWHVTGP GGLDMLVPSV SLIIPPPNPL AVDLATKIEQ YYDAIMALWN QLYINMKSLV
561 SWHYCMIDVE KIRAMTIAKL KTMRREDYQR IIADLEIHYQ EFIRNSQGSE MFGDDDKRNM QTQITDAQKH YQTLVMTLPR
641 QTQTGPPIVT EVITIEKEKQ EAWLLMELQK LRRQIENCEF RMVQRTPFQL DQGASHNLSV RISELESIQI ESQSMAEALN
721 RQKDYLPNFR GSEKYHYLQS EISALFQKLE NINGVSACYL DSLNALRSLL QAILQTDDVI RVFEIRLTEE DTLSLDPDKV
801 EGYRVCLKKM KADLTMKKSL LGTLEAELQK ALQVHSQSSQ TYPHYDLDLG KFADRVSQLR DRWQRLEKQL DDRSWDLEKQ
881 VKQLRIYREL YQALNKWICD ARRRQDTIEA MKLGDVSTVM RYLQEQKNLH SEITSKRDRV EDVVKNAEVC SLAIKDYELQ
961 AAAYSSGLET LLNIPVKRSM VQSPSGLILQ EAGEIQSRYI ELLTRSGDYY KFLSEMLKSL EDIKMKSTRI ELLEEELRLA
1041 KDANSDSNNK HKFLEQNLQK YQVECSQLKA KFISLEEMKR QVEMDGSTAK QNLDKCYAQI KDLNERITRL TYEIEDEKRK
1121 RKLLEDRYDQ QKSDFDQLQK TRHNEKETLG WQKIESEKII KEKEYEIERL RVLLQDEGTR KRECENELAK VRNQFNEEMS
1201 NIKNKYETEI NIKKTTIHQI AAQKEDDAKN LRTQIERLQR ENRDLKDEIV RLNEAILDTT EQRRRAEENV LQQKACGSEV
1281 IQQKQQIELE LKQIIQLRND DNTRYKQALE DATSTIHEKV KELERLKIQL QEEAKSRWEL ENELAKTSHR IQESKSQCNH
1361 IMQERETLLV KIKTLEQDKA RLLRLEEDLN RTKGMLESES RLKARLENEK QQILNDLNQW KSQYSRKEET IRKTECERDK
1441 SEREKSSLLI EIERLQAEIK RIEERYRCRL EETSRKSQAE METERSRWQK EIDKLKKRPY GAHRSTQTEE DLMIDPSKLL
1521 FNGLRKKITA VQLYECQLID KCTLDKLLRG QKSVEEVAAE LEPYLKGAGA IAGASLNTKE KYSLVEAKRK QLLTPENTIL
1601 LLEAQAATGG VIDPHRNELL SVDSAVARDL VDFDDREQIY MAEKAITGFK DPFSGKTMSV AEAIKKNLVD RETGMRLLEA
1681 QLASGGIVDP INSIFLPKDI ALSRGLIDRD LYRALNNPSK PLIDPLSKNA ISYMKLRERC RIEPHTGLLL LPVQKRSMSF
1761 QGIRKLITVS ELVDSGIIRE STANELETGA ITVEEVTDRI KDFLQGSSCI AGIYNEATGE KLGVYQAMKI GLVRPGTALE
1841 LLEAQAATGF IVDPVNNLRL PVEEGYKRGL VGIEFKEKLL SAERAVTGYR DPETGNIISL FQAMNKELIE KGHGVR**LLEA**
1921 **QIATGGIIDP K**ESHRLPVHT AYQRGYFNEE LNDILSDPSD DTKGFFDPNT EENLTYLQLK GRCKIDELTG LCLLPLREKK
2001 KVVQTSQKNT LRKRRVVIVD PDTSKEMSVQ EAYNKGLIDY STYMELSDQE CEWEEITTTG SDGSTRVVLV DRKTGHQYDI
2081 EDAIEKGLVD RKFFEQYRSG SLSLTQFADM IASRNGGDEV FKHESIIRSP TVLSVKSSSI LLKSSSGSFS DSPEEATPIA
2161 AIFDTENLEK ISIPEAVQRG IVDTITAQRL LEAQSCTGGI ICPTTGQRLS LQDAVAQGII DHEMATRLKP AQKAFLGFEG
2241 IKGRKRMSAA EAVKEKWLPY EAGQRFLEFQ FITGGLVDPE VHGRISTEEA IRKGLIDGRT SQRLQDIGNY AKILTCPKTK
2321 LKISYREAMN RSMVEDQTGL RLLEASSVSS KGISSPYNVS SAPGSRSGSR SGSRSGSRSG SRRGSFDAGT SSSYSYSYTT
2401 VSSGSLGR

Supporting Peptides:

Peptide	Uniq	-10lgP	Mass	Length	ppm	m/z	z	RT	Fraction	Scan	Source File	Area F1	Area F2	Area F3	Area I
R.LLEAQIATGGIIDPK.E	Y	62.51	1537.8715	15	0.8	769.9436	2	51.54	6	F6:15197	17112021_RID_2349_DP6.raw				
R.QLQNIIQATSR.E	Y	51.48	1270.6993	11	0.2	636.3571	2	38.60	2	F2:10594	12112021_RID_2349_DP2.raw		1.0785E4		1.2228I
K.SNKPIILR.A	Y	44.33	939.5865	8	-0.1	470.8005	2	24.97	6	F6:6034	17112021_RID_2349_DP6.raw				
R.AELIVQPELK.Y	Y	42.71	1138.6597	10	-0.2	570.3370	2	43.74	6	F6:12471	17112021_RID_2349_DP6.raw				

total 4 peptides

XP_034285000.1
back to list

| Protein Coverage | Supporting Peptides |
Protein Coverage:

1 MSINGSHPRL NTLGRMVRSE SGPDMRYDMN SHMMMGGGGG GGGGGGSSHA HKMYYVQKCY VGDINSEGYT QNGTATVSRR
81 QNTIQDLLQA CSDCLMR**AEL IVQPELK**YGD GIQISRNREL EDCFGQANEH MDILDGLIRE MRQMGQPCDL YQKRLLQLQE
161 QMRALYKAIS VPRVRRASSK GGGYSSQSAS GWDEYTRRVT SECLNWMRQQ KTEMELVKWG FDAAAIEQQI AEHRRFHNSI
241 GDYRWHLDKV KTDLREKAAV HQLEEEYEYL LRFSFERMDQ LR**QLQNIIQA TSR**EIMWIND CEEEELLYDW SDRNTDIARK
321 QEVFSKRMSQ LEVKEKELNK LKQECDQLVL SQHPASDKIE AYMDTLQTQW SWILQITKCI DVHLKENAAY FQFFEEAHST
401 ENYLKNLQDA IRKRFICDKN MSLQVLLEQI KELEAEREKI LEYKRQVQNL VNKSKKIVQL KPRNPEYK**SN KPIILR**ALCD
481 YKQDLKTMRK GDECILKDNS ERSKWHVTGP GGLDMLVPSV SLIIPPPNPL AVDLATKIEQ YYDAIMALWN QLYINMKSLV
561 SWHYCMIDVE KIRAMTIAKL KTMRREDYQR IIADLEIHYQ EFIRNSQGSE MFGDDDKRNM QTQITDAQKH YQTLVMTLPR
641 QTQTGPPIVT EVITIEKEKQ EAWLLMELQK LRRQIENCEF RMVQRTPFQL DQGASHNLSV RISELESIQI ESQSMAEALN
721 RQKDYLPNFR GSEKYHYLQS EISALFQKLE NINGVSACYL DSLNALRSLL QAILQTDDVI RVFEIRLTEE DTLSLDPDKV
801 EGYRVCLKKM KADLTMKKSL LGTLEAELQK ALQVHSQSSQ TYPHYDLDLG KFADRVSQLR DRWQRLEKQL DDRSWDLEKQ
881 VKQLRIYREL YQALNKWICD ARRRQDTIEA MKLGDVSTVM RYLQEQKNLH SEITSKRDRV EDVVKNAEVC SLAIKDYELQ
961 AAAYSSGLET LLNIPVKRSM VQSPSGLILQ EAGEIQSRYI ELLTRSGDYY KFLSEMLKSL EDIKMKSTRI ELLEEELRLA
1041 KDANSDSNNK HKFLEQNLQK YQVECSQLKA KFISLEEMKR QVEMDGSTAK QNLDKCYAQI KDLNERITRL TYEIEDEKRK
1121 RKLLEDRYDQ QKSDFDQLQK TRHNEKETLG WQKIESEKII KEKEYEIERL RVLLQDEGTR KRECENELAK VRNQFNEEMS
1201 NIKNKYETEI NIKKTTIHQI AAQKEDDAKN LRTQIERLQR ENRDLKDEIV RLNEAILDTT EQRRRAEENV LQQKACGSEV
1281 IQQKQQIELE LKQIIQLRND DNTRYKQALE DATSTIHEKV KELERLKIQL QEEAKSRWEL ENELAKVRNS YDEEIISLKN
1361 KYETEINITK TTINQVTVKK EEEASNYRAQ LDNAVRENRS LCEEIRRLKN TLSQTTENLR KVEENAHQQK AIGSEISQKK
1441 QHLEKELKQT VQKFTEEIAH YKQSLDEAGR TLKEKNKEIE KNRKLLDAEI CQRKSLEEEN IRLQRIQYDL EKANINATET
1521 ISRLKVQEQD LGRLKSEYER LAQDKKGKDQ ESTRLQSTIK EMQHQKHMLE EELNRQSRIA ADETSKRKNA EEELEAMRRT
1601 LREQAVKITN LTQQIEEISI VKKRSEDDLR HQREVLDSQM RDKQRYMEEI RKYTSEVEAL RRQMVQEQEN LKQAHIRNEH
1681 LQKAIEDKSK SLNECKIEIE RLQCLTENLT KEHLMLEEEL RNLRLEYDDL RRGRSEVDEE KNATILDLKN QLQTSNKHAL
1761 ELQGVINGLQ KERENLRQEI EKFQKQALET SHRIQESKSQ CNHIMQERET LLVKIKTLEQ DKARLLRLEE DLNRTKGMLE
1841 SESRLKARLE NEKQQILNDL NQWKSQYSRK EETIRKTECE RDKSEREKSS LLIEIERLQA EIKRIEERYR CRLEETSRKS
1921 QAEMETERSR WQKEIDKLKK RPYGAHRSTQ TEEDLMIDPS KLLFNGLRKK ITAVQLYECQ LIDKCTLDKL LRGQKSVEEV
2001 AAELEPYLKG AGAIAGASLN TKEKYSLVEA KRKQLLTPEN TILLLEAQAA TGGVIDPHRN ELLSVDSAVA RDLVDFDDRE
2081 QIYMAEKAIT GFKDPFSGKT MSVAEAIKKN LVDRETGMRL LEAQLASGGI VDPINSIFLP KDIALSRGLI DRDLYRALNN
2161 PSKPLIDPLS KNAISYMKLR ERCRIEPHTG LLLLPVQKRS MSFQGIRKLI TVSELVDSGI IRESTANELE TGAITVEEVT
2241 DRIKDFLQGS SCIAGIYNEA TGEKLGVYQA MKIGLVRPGT ALELLEAQAA TGFIVDPVNN LRLPVEEGYK RGLVGIEFKE
2321 KLLSAERAVT GYRDPETGNI ISLFQAMNKE LIEKGHGVR**L LEAQIATGGI IDPK**ESHRLP VHTAYQRGYF NEELNDILSD
2401 PSDDTKGFFD PNTEENLTYL QLKGRCKIDE LTGLCLLPLR EKKKVVQTSQ KNTLRKRRVV IVDPDTSKEM SVQEAYNKGL
2481 IDYSTYMELS DQECEWEEIT TTGSDGSTRV VLVDRKTGHQ YDIEDAIEKG LVDRKFFEQY RSGSLSLTQF ADMIASRNGG
2561 DEVFKHESII RSPTVLSVKS SSILLKSSSG SFSDSPEEAT PIAAIFDTEN LEKISIPEAV QRGIVDTITA QRLLEAQSCT
2641 GGIICPTTGQ RLSLQDAVAQ GIIDHEMATR LKPAQKAFLG FEGIKGRKRM SAAEAVKEKW LPYEAGQRFL EFQFITGGLV
2721 DPEVHGRIST EEAIRKGLID GRTSQRLQDI GNYAKILTCP KTKLKISYRE AMNRSMVEDQ TGLRLLEASS VSSKGISSPY
2801 NVSSAPGSRS GSRSGSRSGS RSGSRRGSFD AGTSSSYSYS YTTVSSGSLG R

Supporting Peptides:

Peptide	Uniq	-10lgP	Mass	Length	ppm	m/z	z	RT	Fraction	Scan	Source File	Area F1	Area F2	Area F3	Area F
R.LLEAQIATGGIIDPK.E	Y	62.51	1537.8715	15	0.8	769.9436	2	51.54	6	F6:15197	17112021_RID_2349_DP6.raw				
R.QLQNIIQATSR.E	Y	51.48	1270.6993	11	0.2	636.3571	2	38.60	2	F2:10594	12112021_RID_2349_DP2.raw		1.0785E4		1.2228
K.SNKPIILR.A	Y	44.33	939.5865	8	-0.1	470.8005	2	24.97	6	F6:6034	17112021_RID_2349_DP6.raw				
R.AELIVQPELK.Y	Y	42.71	1138.6597	10	-0.2	570.3370	2	43.74	6	F6:12471	17112021_RID_2349_DP6.raw				

total 4 peptides

JAG67762.1
back to list

| Protein Coverage | Supporting Peptides |
Protein Coverage:

1 M**VHWSAEEK**H MITSLWSKVD VAEVGGATLG K**LMVVYPWTQ R**FFAHFGNLS GPSAICGNPQ VKAHGK**KVLT SFGEAIK**HLD C
81 NVKETFAKLS ELHCDKLHVD PENFKLLGNI LIIVLAGHHG KEFTPCVHAT FQKLTNVVAH SLARRYH

Supporting Peptides:

Peptide	Uniq	-10lgP	Mass	Length	ppm	m/z	z	RT	Fraction	Scan	Source File	Area F1	Area F2	Area F3	Area F4	A
K.VLTSFGEAIK.H	N	61.56	1063.5913	10	-0.1	532.8029	2	41.86	7	F7:11920	17112021_RID_2349_DP7.raw					

total 5 peptides

Peptide	Uniq	-10lgP	Mass	Length	ppm	m/z	z	RT	Fraction	Scan	Source File	Area F1	Area F2	Area F3	Area F4	A
K.LMVVYPWTQR.F	Y	58.78	1291.6747	10	0.4	646.8448	2	54.62	7	F7:16340	17112021_RID_2349_DP7.raw					
M.VHWSAEEK.H	N	52.31	984.4665	8	0.7	493.2408	2	21.02	7	F7:4832	17112021_RID_2349_DP7.raw					
K.LM(+15.99)VVYPWTQR.F	Y	50.62	1307.6697	10	0.1	654.8422	2	51.02	7	F7:15084	17112021_RID_2349_DP7.raw					
K.KVLTSFGEAIK.H	N	48.51	1191.6863	11	1.5	596.8513	2	36.27	7	F7:10106	17112021_RID_2349_DP7.raw					

total 5 peptides

JAG67761.1
back to list

| Protein Coverage | Supporting Peptides |
Protein Coverage:

1 MVHWSAEEKH MITSLWSKVD VAEVGGATLG KLMVVYPWTQ RFFAHFGNLS GPSAICGNPQ VKAHGKKVLT SFGEAIKHLD

81 NVKETFAKLS ELHCDKLHVD PENFKLLGNI LIIVLAGHHG KEFTPCVHAT FQKLTNVVAH SLARRYH

Supporting Peptides:

Peptide	Uniq	-10lgP	Mass	Length	ppm	m/z	z	RT	Fraction	Scan	Source File	Area F1	Area F2	Area F3	Area F4	A
K.VLTSFGEAIK.H	N	61.56	1063.5913	10	-0.1	532.8029	2	41.86	7	F7:11920	17112021_RID_2349_DP7.raw					
K.LMVVYPWTQR.F	Y	58.78	1291.6747	10	0.4	646.8448	2	54.62	7	F7:16340	17112021_RID_2349_DP7.raw					
M.VHWSAEEK.H	N	52.31	984.4665	8	0.7	493.2408	2	21.02	7	F7:4832	17112021_RID_2349_DP7.raw					
K.LM(+15.99)VVYPWTQR.F	Y	50.62	1307.6697	10	0.1	654.8422	2	51.02	7	F7:15084	17112021_RID_2349_DP7.raw					
K.KVLTSFGEAIK.H	N	48.51	1191.6863	11	1.5	596.8513	2	36.27	7	F7:10106	17112021_RID_2349_DP7.raw					

total 5 peptides

XP_015679695.1
back to list

| Protein Coverage | Supporting Peptides |
Protein Coverage:

1 MKGRLKGAWI LSLLLAGLFQ PNLGQEQQEI LTGLQLSHQH DEARSCLHAQ PIPDRAKVDC YDSVQGSAHD YGALSLSGEY

81 IPFRNYAGKY ILFVNVATYU GLTGQYLELN ALQTSLGIDR LVILGFPCNQ FGKQEPGQNS EILQGIKHVR PGGGFVPNFQ

161 LFQKGDVNGE NEQRIYTFLK NSCPPVVENF GDPAKLFWSP LKTHDIKWNF EKFLVNPQGK PVMRWFQRTN VSSVKNDIIR

241 YMRKNG

Supporting Peptides:

Peptide	Uniq	-10lgP	Mass	Length	ppm	m/z	z	RT	Fraction	Scan	Source File	Area F1	Area F2	Area F3	Area F
K.QEPGQNSEILQGIK.H	Y	85.68	1539.7893	14	0.2	770.9021	2	41.31	5	F5:11775	17112021_RID_2349_DP5.raw				1.0455E
R.TNVSSVKNDIIR.Y	Y	68.80	1344.7361	12	0.4	673.3756	2	32.75	1	F1:9508	12112021_RID_2349_DP1.raw	2.0856E5			

total 2 peptides

ADP88559.1
back to list

| Protein Coverage | Supporting Peptides |
Protein Coverage:

1 MVLIRVLANL LVLQLSYAQK SSELVVGGHP CNIYEHHFLA FMYNSSGFMC SGTLINQQWV LSAAHCDMEN MHIYLGLHSF

81 KLPNKDQKKR VAKEKFFCLS SKSYTKWDKD IMLIKLNKPV TYSTHIASLS LPSNPPRVGS VCRIMGWGSI TSPKKILPFV

161 PHCANINIVP YTVCRVIYRP LPEQSRTLCA GVSGRRIGSC LGDSGGPLIC NGQIQGIVSW GSDPCVNRGA PSIYTKVFDY

241 TDWIHSIIAG NTAATCPS

Supporting Peptides:

Peptide	Uniq	-10lgP	Mass	Length	ppm	m/z	z	RT	Fraction	Scan	Source File	Area F1	Area F2	Are
K.WDKDIMLIK.L	N	57.12	1160.6263	9	-0.1	581.3204	2	46.64	2	F2:13338	12112021_RID_2349_DP2.raw	2.1169E5	9.915E6	1.51
K.WDKDIM(+15.99)LIK.L	N	52.20	1176.6212	9	-0.2	589.3177	2	41.27	2	F2:11445	12112021_RID_2349_DP2.raw		6.6323E6	2.11
R.IM(+15.99)GWGSITSPK.K	Y	51.75	1191.5958	11	1.1	596.8058	2	41.44	7	F7:11830	17112021_RID_2349_DP7.raw			
S.LSLPSNPPR.V	N	41.33	979.5450	9	-0.2	490.7797	2	34.94	7	F7:9564	17112021_RID_2349_DP7.raw			

total 5

Peptide	Uniq	-10lgP	Mass	Length	ppm	m/z	z	RT	Fraction	Scan	Source File	Area F1	Area F2	Are
R.IMGWGSITSPK.K	Y	40.20	1175.6008	11	-0.1	588.8076	2	47.84	4	F4:13831	17112021_RID_2349_DP4.raw			

total 5

E5L0E3.1
[back to list](#)

| [Protein Coverage](#) | [Supporting Peptides](#) |
Protein Coverage:

```
  1  MVLIRVLANL LVLQLSYAQK SSELVVGGHP CNIYEHHFLA FMYNSSGFMC SGTLINQQWV LSAAHCDMEN MHIYLGLHSF
                                   112
 81  KLPNKDQKKR VAKEKFFCLS SKSYTKWDKD IMLIKLNKPV TYSTHIASLS LPSNPPRVGS VCRIMGWGSI TSPKKILPFV
161  PHCANINIVP YTVCRVIYRP LPEQSRTLCA GVSGRRIGSC LGDSGGPLIC NGQIQGIVSW GSDPCVNRGA PSIYTKVFDY
241  TDWIHSIIAG NTAATCPS
```

Supporting Peptides:

Peptide	Uniq	-10lgP	Mass	Length	ppm	m/z	z	RT	Fraction	Scan	Source File	Area F1	Area F2	Are
K.WDKDIMLIK.L	N	57.12	1160.6263	9	-0.1	581.3204	2	46.64	2	F2:13338	12112021_RID_2349_DP2.raw	2.1169E5	9.915E6	1.51[illegible]
K.WDKDIM(+15.99)LIK.L	N	52.20	1176.6212	9	-0.2	589.3177	2	41.27	2	F2:11445	12112021_RID_2349_DP2.raw		6.6323E6	2.11[illegible]
R.IM(+15.99)GWGSITSPK.K	Y	51.75	1191.5958	11	1.1	596.8058	2	41.44	7	F7:11830	17112021_RID_2349_DP7.raw			
S.LSLPSNPPR.V	N	41.33	979.5450	9	-0.2	490.7797	2	34.94	7	F7:9564	17112021_RID_2349_DP7.raw			
R.IMGWGSITSPK.K	Y	40.20	1175.6008	11	-0.1	588.8076	2	47.84	4	F4:13831	17112021_RID_2349_DP4.raw			

total 5

P0DJL5.1
[back to list](#)

| [Protein Coverage](#) | [Supporting Peptides](#) |
Protein Coverage:

```
                                                                         68
  1  DQDCLPGWSY FEKYCYKVFQ VKKNWEDAEK FCTEEVKDGH LISLHSNEEV EFMTSLAFPI LKYDIVWMGL RNFWRDCPWK
 81  WSDDAKLDYK AWSDEPNCYG AMTTDYQWLR WNCNDPRYFV CKSPA
```

Supporting Peptides:

Peptide	Uniq	-10lgP	Mass	Length	ppm	m/z	z	RT	Fraction	Scan	Source File	Area F1	Area F2	Area F3	Area F4
K.YDIVWMGLR.N	Y	67.55	1151.5797	9	0.6	576.7975	2	60.05	7	F7:18181	17112021_RID_2349_DP7.raw			1.1179E5	
K.YDIVWM(+15.99)GLR.N	Y	64.95	1167.5747	9	0.9	584.7952	2	53.11	7	F7:15854	17112021_RID_2349_DP7.raw				
K.WSDDAKLDYK.A	Y	58.85	1239.5771	10	0.7	620.7963	2	32.61	7	F7:8789	17112021_RID_2349_DP7.raw				
M.TTDYQWLR.W	Y	57.81	1081.5193	8	0.1	541.7670	2	42.38	7	F7:12141	17112021_RID_2349_DP7.raw				

total 4 pep

AMB36341.1
[back to list](#)

| [Protein Coverage](#) | [Supporting Peptides](#) |
Protein Coverage:

```
  1  MGRLISISFG LLVVFLSLSG TGADQDCLPG WSYFEKYCYK VFKVKKNWEE AEKFCTEEVK DGHLISLHSN EEVEFMTSLA
                  91
 81  FPILKYDIVW MGLRNFWRDC PWKWSDDAKL DYKAWSDEPN CYGAKTTDYQ WLRWNCNDPR YFVCKSLA
```

Supporting Peptides:

Peptide	Uniq	-10lgP	Mass	Length	ppm	m/z	z	RT	Fraction	Scan	Source File	Area F1	Area F2	Area F3	Area F4
K.YDIVWMGLR.N	Y	67.55	1151.5797	9	0.6	576.7975	2	60.05	7	F7:18181	17112021_RID_2349_DP7.raw			1.1179E5	
K.YDIVWM(+15.99)GLR.N	Y	64.95	1167.5747	9	0.9	584.7952	2	53.11	7	F7:15854	17112021_RID_2349_DP7.raw				
K.WSDDAKLDYK.A	Y	58.85	1239.5771	10	0.7	620.7963	2	32.61	7	F7:8789	17112021_RID_2349_DP7.raw				
K.TTDYQWLR.W	Y	57.81	1081.5193	8	0.1	541.7670	2	42.38	7	F7:12141	17112021_RID_2349_DP7.raw				

total 4 pep

TRINITY_DN554
[back to list](#)

| Protein Coverage | Supporting Peptides |

Protein Coverage:

```
  1  LSAGKEGRKT MGRFISVSFS LLVVFLSLSG TEAGFCCPNG WSSFGQHCYK VIEPLKNWTD AEKFCTEQHK GSHLASIHSS
                                   101
 81  EEEAFVVKLA FQTLKSEILW MGLSKVWNEC NWGWSNGAKL KYEAWAEESY CVYFSS
```

Supporting Peptides:

Peptide	Uniq	-10lgP	Mass	Length	ppm	m/z	z	RT	Fraction	Scan	Source File	Area F1	Area F2	Area F3
K.SEILWMGLSK.V	Y	72.18	1162.6056	10	0.3	582.3102	2	55.68	7	F7:16745	17112021_RID_2349_DP7.raw		9.069E4	8.6025E5
K.SEILWM(+15.99)GLSK.V	Y	64.35	1178.6006	10	-0.5	590.3073	2	48.60	7	F7:14258	17112021_RID_2349_DP7.raw		8.1789E3	
H.SSEEEAFVVK.L	N	41.08	1123.5397	10	-0.5	562.7769	2	34.73	6	F6:9332	17112021_RID_2349_DP6.raw			
K.LAFQTLK.S	N	39.89	819.4854	7	0.6	410.7502	2	37.72	7	F7:10543	17112021_RID_2349_DP7.raw			

total 4

TRINITY_DN554

back to list

| Protein Coverage | Supporting Peptides |

Protein Coverage:

```
  1  LSAGKEGRKT MGRFISVSFS LLVVFLSLSG TEAGFCCPNG WSSFGQHCYK VIEPLKNWTD AEKFCTEQHK GSHLASIHSS
                                   101
 81  EEEAFVVKLA FQTLKSEILW MGLSKVWNVG CL
```

Supporting Peptides:

Peptide	Uniq	-10lgP	Mass	Length	ppm	m/z	z	RT	Fraction	Scan	Source File	Area F1	Area F2	Area F3
K.SEILWMGLSK.V	Y	72.18	1162.6056	10	0.3	582.3102	2	55.68	7	F7:16745	17112021_RID_2349_DP7.raw		9.069E4	8.6025E5
K.SEILWM(+15.99)GLSK.V	Y	64.35	1178.6006	10	-0.5	590.3073	2	48.60	7	F7:14258	17112021_RID_2349_DP7.raw		8.1789E3	
H.SSEEEAFVVK.L	N	41.08	1123.5397	10	-0.5	562.7769	2	34.73	6	F6:9332	17112021_RID_2349_DP6.raw			
K.LAFQTLK.S	N	39.89	819.4854	7	0.6	410.7502	2	37.72	7	F7:10543	17112021_RID_2349_DP7.raw			

total 4

XP_015666085.1

back to list

| Protein Coverage | Supporting Peptides |

Protein Coverage:

```
  1  MCKITTALLG LLSLSFFLEL SSCHVRRNAM GSIFRRLKHA LPSQGDPGQP LFLTPYIESG KIEEGRQLSL VEHLPGPHVK
 81  SYSGYLTVNK THNSNLFFWF FPAQVQPEKT PVLLWLQGGP GGTSMFGLFV EHGPYVVQKN LSLTERKFPW TSKFSMLYID
161  NPVGTGFSFT DDPKGYAKNE DDVGRDLYSA IIQFFQLFPH YQKNDFYATG ESYAGKYVPA IGYYIHSNNP TAKIKINFKG
241  VAIGDGLCDP EVMLGGYAEF LYQIGLVDEN QKIYVQNQSN LGQQYIQQKK WKEAFEVFDI LLNSDKTGTP SYIQTVSGCS
321  NYFNFMQCQE PEDQEYFGKL LSLPNIRKSI HVGNLTFHDG SVVEEYLLED VMKTIKPWLA VLMDHYRVLL YNGQLDIIVA
401  APLTERFLPT VPWGKVKEYK NAERIVWRIH DKDPEVAGYV RQVGEFYQVI VRGGGHILPY DQPERALDMM DRFIMGKGWK
```

Supporting Peptides:

Peptide	Uniq	-10lgP	Mass	Length	ppm	m/z	z	RT	Fraction	Scan	Source File	Area F1	Area F2	Area F3
K.NDFYATGESYAGK.Y	Y	72.10	1421.6099	13	-0.1	711.8121	2	37.11	2	F2:10004	12112021_RID_2349_DP2.raw	1.9882E5	1.3335E5	
R.QVGEFYQVIVR.G	Y	42.38	1336.7139	11	0.4	669.3645	2	51.49	1	F1:15706	12112021_RID_2349_DP1.raw	1.4401E5		
R.GGGHILPYDQPER.A	Y	40.27	1437.7001	13	1.0	719.8580	2	31.99	3	F3:8551	17112021_RID_2349_DP3.raw			8.9043E4

total 3 peptides

XP_015666086.1

back to list

| Protein Coverage | Supporting Peptides |

Protein Coverage:

1 MCKITTALLG LLSLSFFLEL SSCHVRRNAM GSIFRRLKHA LPSQGDPGQP LFLTPYIESG KIEEGRQLSL VEHLPGPHVK
81 SYSGYLTVNK THNSNLFFWF FPAQVQPEKT PVLLWLQGGP GGTSMFGLFV EHGPYVVQKN LSLTERKFPW TSKFSMLYID
161 NPVGTGFSFT DDPKGYAKNE DDVGRDLYSA IIQFFQLFPH YQKNDFYATG ESYAGKYVPA IGYYIHSNNP TAKIKINFKG
241 VAIGDGLCDP EVMLGGYAEF LYQIGLVDEN QKIYVQNQSN LGQQYIQQKK WKEAFEVFDI LLNSDKTGTP SYIQTVSGCS
321 NYFNFMQCQE PEDQEYFGKL LSLPNIRKSI HVGNLTFHDG SVVEEYLLED VMKTIKPWLA VLMDHYRVLL YNGQLDIIVA
401 APLTERFLPT VPWGKVKEYK NAERIVWRIH DKDPEVAGYV RQVGEFYQVI VRGGGHILPY DQPERALDMM DRFIMGKGWK

Supporting Peptides:

Peptide	Uniq	-10lgP	Mass	Length	ppm	m/z	z	RT	Fraction	Scan	Source File	Area F1	Area F2	Area F3
K.NDFYATGESYAGK.Y	Y	72.10	1421.6099	13	-0.1	711.8121	2	37.11	2	F2:10004	12112021_RID_2349_DP2.raw	1.9882E5	1.3335E5	
R.QVGEFYQVIVR.G	Y	42.38	1336.7139	11	0.4	669.3645	2	51.49	1	F1:15706	12112021_RID_2349_DP1.raw	1.4401E5		
R.GGGHILPYDQPER.A	Y	40.27	1437.7001	13	1.0	719.8580	2	31.99	3	F3:8551	17112021_RID_2349_DP3.raw			8.9043E4

total 3 peptides

XP_034258446.1
back to list

| Protein Coverage | Supporting Peptides |
Protein Coverage:

1 MCKITTVLLA LLSLSFSLEL SSCHVRWSAM GNIFRRLKYA LPSQGDPGQP LFLTPYIESG KTEEGRQLSL VEHLPGPNVK
81 SYSGYLTVNK TYNNNLFFWF FPAQVQPEKA PVLLWLQGGP GGTSMFGLFV EHGPYVVQKN LSLTERKFPW TSKFSMLYID
161 NPVGTGFSFT DDPKGYAKNE VDVGRDLYSA IVQFFQLFPD YQKNDFYATG ESYAGKYVPA IGYYIHSNNP TAKIKINFKG
241 VAIGDGLCDP EVMLGGYAEF LYQIGLVDEK QRIYVQNQTN LGQKYIQQKK WKEAFEVFDT LLNGDKTGSS PYIQTVTGCS
321 NYFNFLQCEE PEDQEYFGKL LSLPNIRKSI HVGNLTFHDG SIVEEHLIED VMKTIKPWLA VLMDHYRVLL YNGQLDIIVA
401 APLTERFLPT VPWGKVKEYK NAERIVWRIH DKDPEVAGYV RQVGEFYQVI VRGGGHILPY DQPERALDMM DRFIMGKEWK
481 QSRYGK

Supporting Peptides:

Peptide	Uniq	-10lgP	Mass	Length	ppm	m/z	z	RT	Fraction	Scan	Source File	Area F1	Area F2	Area F3
K.NDFYATGESYAGK.Y	Y	72.10	1421.6099	13	-0.1	711.8121	2	37.11	2	F2:10004	12112021_RID_2349_DP2.raw	1.9882E5	1.3335E5	
R.QVGEFYQVIVR.G	Y	42.38	1336.7139	11	0.4	669.3645	2	51.49	1	F1:15706	12112021_RID_2349_DP1.raw	1.4401E5		
R.GGGHILPYDQPER.A	Y	40.27	1437.7001	13	1.0	719.8580	2	31.99	3	F3:8551	17112021_RID_2349_DP3.raw			8.9043E4

total 3 peptides

XP_034258445.1
back to list

| Protein Coverage | Supporting Peptides |
Protein Coverage:

1 MCKITTVLLA LLSLSFSLEL SSCHVRWSAM GNIFRRLKYA LPSQGDPGQP LFLTPYIESG KTEEGRQLSL VEHLPGPNVK
81 SYSGYLTVNK TYNNNLFFWF FPAQVQPEKA PVLLWLQGGP GGTSMFGLFV EHGPYVVQKN LSLTERKFPW TSKFSMLYID
161 NPVGTGFSFT DDPKGYAKNE VDVGRDLYSA IVQFFQLFPD YQKNDFYATG ESYAGKYVPA IGYYIHSNNP TAKIKINFKG
241 VAIGDGLCDP EVMLGGYAEF LYQIGLVDEK QRIYVQNQTN LGQKYIQQKK WKEAFEVFDT LLNGDKTGSS PYIQTVTGCS
321 NYFNFLQCEE PEDQEYFGKL LSLPNIRKSI HVGNLTFHDG SIVEEHLIED VMKTIKPWLA VLMDHYRVLL YNGQLDIIVA
401 APLTERFLPT VPWGKVKEYK NAERIVWRIH DKDPEVAGYV RQVGEFYQVI VRGGGHILPY DQPERALDMM DRFIMGKEWK
481 QSRYGK

Supporting Peptides:

Peptide	Uniq	-10lgP	Mass	Length	ppm	m/z	z	RT	Fraction	Scan	Source File	Area F1	Area F2	Area F3
K.NDFYATGESYAGK.Y	Y	72.10	1421.6099	13	-0.1	711.8121	2	37.11	2	F2:10004	12112021_RID_2349_DP2.raw	1.9882E5	1.3335E5	
R.QVGEFYQVIVR.G	Y	42.38	1336.7139	11	0.4	669.3645	2	51.49	1	F1:15706	12112021_RID_2349_DP1.raw	1.4401E5		
R.GGGHILPYDQPER.A	Y	40.27	1437.7001	13	1.0	719.8580	2	31.99	3	F3:8551	17112021_RID_2349_DP3.raw			8.9043E4

total 3 peptides

XP_034258444.1
back to list

| Protein Coverage | Supporting Peptides |
Protein Coverage:

1 MCKITTVLLA LLSLSFSLEL SSCHVRWSAM GNIFRRLKYA LPSQGDPGQP LFLTPYIESG KTEEGRQLSL VEHLPGPNVK
81 SYSGYLTVNK TYNNNLFFWF FPAQVQPEKA PVLLWLQGGP GGTSMFGLFV EHGPYVVQKN LSLTERKFPW TSKFSMLYID
161 NPVGTGFSFT DDPKGYAKNE VDVGRDLYSA IVQFFQLFPD YQK**NDFYATG ESYAGK**YVPA IGYYIHSNNP TAKIKINFKG
241 VAIGDGLCDP EVMLGGYAEF LYQIGLVDEK QRIYVQNQTN LGQKYIQQKK WKEAFEVFDT LLNGDKTGSS PYIQTVTGCS
321 NYFNFLQCEE PEDQEYFGKL LSLPNIRKSI HVGNLTFHDG SIVEEHLIED VMKTIKPWLA VLMDHYRVLL YNGQLDIIVA
401 APLTERFLPT VPWGKVKEYK NAERIVWRIH DKDPEVAGYV R**QVGEFYQVI VRGGGHILPY DQPER**ALDMM DRFIMGKEWK
481 QSRYGK

Supporting Peptides:

Peptide	Uniq	-10lgP	Mass	Length	ppm	m/z	z	RT	Fraction	Scan	Source File	Area F1	Area F2	Area F3
K.NDFYATGESYAGK.Y	Y	72.10	1421.6099	13	-0.1	711.8121	2	37.11	2	F2:10004	12112021_RID_2349_DP2.raw	1.9882E5	1.3335E5	
R.QVGEFYQVIVR.G	Y	42.38	1336.7139	11	0.4	669.3645	2	51.49	1	F1:15706	12112021_RID_2349_DP1.raw	1.4401E5		
R.GGGHILPYDQPER.A	Y	40.27	1437.7001	13	1.0	719.8580	2	31.99	3	F3:8551	17112021_RID_2349_DP3.raw			8.9043E4

total 3 peptides

XP_032092626.1
back to list

| Protein Coverage | Supporting Peptides |
Protein Coverage:

1 MCKITALLAL LSLIFSLELS SCHVRWSAMG SIFRRLKYAL PSQGDPGQPL FLTPYIESGE TEEGRQLSLV EHLPGPNVKS
81 YSGYLTVNKT YNSNLFFWFF PAQFQPEKAP VLLWLQGGPG GTSMFGLFVE HGPYVVQKNL SLTERKFPWT SKFSMLYIDN
161 PVGTGFSFTD DPKGYAKNED DVGRDLYSAI VQFFQLFPDY QK**NDFYATGE SYAGK**YVPAI GYYIHSNNPT AKIKINFKGV
241 AIGDGLCDPE VMLGGYAEFL YQIGLLDEKQ KIYVQNQTNL GQQYIQQKKW KEAFEVFDTL LNGDKTGSPP YVDTVTGCSN
321 YFNFLKCEEP EDQKYFGKLL SLPNIRKSIH VGNLTFHDGS MVEEHLVEDI MKTIKPWLAV LMDHYKVLLY NGQLDIIVAA
401 PLTERFLPTV PWGKVKEYKN AERIIWRIHD KDPEVAGYVR **QVGEFYQVIV RGGGHILPYD QPER**ALDMMD RFIMGKEWK

Supporting Peptides:

Peptide	Uniq	-10lgP	Mass	Length	ppm	m/z	z	RT	Fraction	Scan	Source File	Area F1	Area F2	Area F3
K.NDFYATGESYAGK.Y	Y	72.10	1421.6099	13	-0.1	711.8121	2	37.11	2	F2:10004	12112021_RID_2349_DP2.raw	1.9882E5	1.3335E5	
R.QVGEFYQVIVR.G	Y	42.38	1336.7139	11	0.4	669.3645	2	51.49	1	F1:15706	12112021_RID_2349_DP1.raw	1.4401E5		
R.GGGHILPYDQPER.A	Y	40.27	1437.7001	13	1.0	719.8580	2	31.99	3	F3:8551	17112021_RID_2349_DP3.raw			8.9043E4

total 3 peptides

XP_032092625.1
back to list

| Protein Coverage | Supporting Peptides |
Protein Coverage:

1 MCKITALLAL LSLIFSLELS SCHVRWSAMG SIFRRLKYAL PSQGDPGQPL FLTPYIESGE TEEGRQLSLV EHLPGPNVKS
81 YSGYLTVNKT YNSNLFFWFF PAQFQPEKAP VLLWLQGGPG GTSMFGLFVE HGPYVVQKNL SLTERKFPWT SKFSMLYIDN
161 PVGTGFSFTD DPKGYAKNED DVGRDLYSAI VQFFQLFPDY QK**NDFYATGE SYAGK**YVPAI GYYIHSNNPT AKIKINFKGV
241 AIGDGLCDPE VMLGGYAEFL YQIGLLDEKQ KIYVQNQTNL GQQYIQQKKW KEAFEVFDTL LNGDKTGSPP YVDTVTGCSN
321 YFNFLKCEEP EDQKYFGKLL SLPNIRKSIH VGNLTFHDGS MVEEHLVEDI MKTIKPWLAV LMDHYKVLLY NGQLDIIVAA
401 PLTERFLPTV PWGKVKEYKN AERIIWRIHD KDPEVAGYVR **QVGEFYQVIV RGGGHILPYD QPER**ALDMMD RFIMGKEWK

Supporting Peptides:

Peptide	Uniq	-10lgP	Mass	Length	ppm	m/z	z	RT	Fraction	Scan	Source File	Area F1	Area F2	Area F3
K.NDFYATGESYAGK.Y	Y	72.10	1421.6099	13	-0.1	711.8121	2	37.11	2	F2:10004	12112021_RID_2349_DP2.raw	1.9882E5	1.3335E5	
R.QVGEFYQVIVR.G	Y	42.38	1336.7139	11	0.4	669.3645	2	51.49	1	F1:15706	12112021_RID_2349_DP1.raw	1.4401E5		
R.GGGHILPYDQPER.A	Y	40.27	1437.7001	13	1.0	719.8580	2	31.99	3	F3:8551	17112021_RID_2349_DP3.raw			8.9043E4

total 3 peptides

XP_032092628.1
back to list

| Protein Coverage | Supporting Peptides |
Protein Coverage:

1 MCKITALLAL LSLIFSLELS SCHVRWSAMG SIFRRLKYAL PSQGDPGQPL FLTPYIESGE TEEGRQLSLV EHLPGPNVKS
81 YSGYLTVNKT YNSNLFFWFF PAQFQPEKAP VLLWLQGGPG GTSMFGLFVE HGPYVVQKNL SLTERKFPWT SKFSMLYIDN
161 PVGTGFSFTD DPKGYAKNED DVGRDLYSAI VQFFQLFPDY QK**NDFYATGE SYAGK**YVPAI GYYIHSNNPT AKIKINFKGV
241 AIGDGLCDPE VMLGGYAEFL YQIGLLDEKQ KIYVQNQTNL GQQYIQQKKW KEAFEVFDTL LNGDKTGSPP YVDTVTGCSN
321 YFNFLKCEEP EDQKYFGKLL SLPNIRKSIH VGNLTFHDGS MVEEHLVEDI MKTIKPWLAV LMDHYKVLLY NGQLDIIVAA
401 PLTERFLPTV PWGKVKEYKN AERIIWRIHD KDPEVAGYVR **QVGEFYQVIV RGGGHILPYD QPER**ALDMMD RFIMGKEWK

Supporting Peptides:

Peptide	Uniq	-10lgP	Mass	Length	ppm	m/z	z	RT	Fraction	Scan	Source File	Area F1	Area F2	Area F3
K.NDFYATGESYAGK.Y	Y	72.10	1421.6099	13	-0.1	711.8121	2	37.11	2	F2:10004	12112021_RID_2349_DP2.raw	1.9882E5	1.3335E5	
R.QVGEFYQVIVR.G	Y	42.38	1336.7139	11	0.4	669.3645	2	51.49	1	F1:15706	12112021_RID_2349_DP1.raw	1.4401E5		
R.GGGHILPYDQPER.A	Y	40.27	1437.7001	13	1.0	719.8580	2	31.99	3	F3:8551	17112021_RID_2349_DP3.raw			8.9043E4

total 3 peptides

XP_013922774.1

back to list

| Protein Coverage | Supporting Peptides |

Protein Coverage:

1 MCKITALLAL LSLIFSLELS SCHVRWSAMG SIFRRLKYAL PSQGDPGQPL FLTPYIESGE TEEGRQLSLV EHLPGPNVKS
81 YSGYLTVNKT YNSNLFFWFF PAQFQPEKAP VLLWLQGGPG GTSMFGLFVE HGPYVVQKNL SLTERKFPWT SKFSMLYIDN
161 PVGTGFSFTD DPKGYAKNED DVGRDLYSAI VQFFQLFPDY QK**NDFYATGE SYAGK**YVPAI GYYIHSNNPT AKIKINFKGV
241 AIGDGLCDPE VMLGGYAEFL YQIGLLDEKQ KIYVQNQTNL GQQYIQQKKW KEAFEVFDTL LNGDKTGSPP YVHTVTGCSN
321 YFNFLKCEEP EDQEYFGNLL SLPNIRKSIH VGNLTFHDGS MVEEHLVEDI MKTIKPWLAV LMDHYRVLLY NGQLDIIVAA
401 PLTERFLPTV PWGKVKEYKD AERIIWRIHD KDPEVAGYVR **QVGEFYQVIV RGGGHILPYD QPER**ALDMMD RFIMGKEWK

Supporting Peptides:

Peptide	Uniq	-10lgP	Mass	Length	ppm	m/z	z	RT	Fraction	Scan	Source File	Area F1	Area F2	Area F3
K.NDFYATGESYAGK.Y	Y	72.10	1421.6099	13	-0.1	711.8121	2	37.11	2	F2:10004	12112021_RID_2349_DP2.raw	1.9882E5	1.3335E5	
R.QVGEFYQVIVR.G	Y	42.38	1336.7139	11	0.4	669.3645	2	51.49	1	F1:15706	12112021_RID_2349_DP1.raw	1.4401E5		
R.GGGHILPYDQPER.A	Y	40.27	1437.7001	13	1.0	719.8580	2	31.99	3	F3:8551	17112021_RID_2349_DP3.raw			8.9043E4

total 3 peptides

XP_032092627.1

back to list

| Protein Coverage | Supporting Peptides |

Protein Coverage:

1 MCKITALLAL LSLIFSLELS SCHVRWSAMG SIFRRLKYAL PSQGDPGQPL FLTPYIESGE TEEGRQLSLV EHLPGPNVKS
81 YSGYLTVNKT YNSNLFFWFF PAQFQPEKAP VLLWLQGGPG GTSMFGLFVE HGPYVVQKNL SLTERKFPWT SKFSMLYIDN
161 PVGTGFSFTD DPKGYAKNED DVGRDLYSAI VQFFQLFPDY QK**NDFYATGE SYAGK**YVPAI GYYIHSNNPT AKIKINFKGV
241 AIGDGLCDPE VMLGGYAEFL YQIGLLDEKQ KIYVQNQTNL GQQYIQQKKW KEAFEVFDTL LNGDKTGSPP YVDTVTGCSN
321 YFNFLKCEEP EDQKYFGKLL SLPNIRKSIH VGNLTFHDGS MVEEHLVEDI MKTIKPWLAV LMDHYKVLLY NGQLDIIVAA
401 PLTERFLPTV PWGKVKEYKN AERIIWRIHD KDPEVAGYVR **QVGEFYQVIV RGGGHILPYD QPER**ALDMMD RFIMGKEWK

Supporting Peptides:

Peptide	Uniq	-10lgP	Mass	Length	ppm	m/z	z	RT	Fraction	Scan	Source File	Area F1	Area F2	Area F3
K.NDFYATGESYAGK.Y	Y	72.10	1421.6099	13	-0.1	711.8121	2	37.11	2	F2:10004	12112021_RID_2349_DP2.raw	1.9882E5	1.3335E5	
R.QVGEFYQVIVR.G	Y	42.38	1336.7139	11	0.4	669.3645	2	51.49	1	F1:15706	12112021_RID_2349_DP1.raw	1.4401E5		
R.GGGHILPYDQPER.A	Y	40.27	1437.7001	13	1.0	719.8580	2	31.99	3	F3:8551	17112021_RID_2349_DP3.raw			8.9043E4

total 3 peptides

JAV50864.1

back to list

| Protein Coverage | Supporting Peptides |

Protein Coverage:

1 MCKITTALLG LLSLSFFLEL SSCHVRWNAI GSIFRRLKHA LPSQGDPGQP LFLTPYIESG KIEEGRQLSL VEHLPGPHVK
81 SYSGYLTVNK THNSNLFFWF FPAQVQPEKT PVLLWLQGGP GGTSMFGLFV EHGPYVVQKN LSLTERKFPW TSKFSMLYID
161 NPVGTGFSFT DDPKGYAKNE DDVGRDLYSA IIQFFQLFPH YQK**NDFYATG ESYAGK**YVPA IGYYIHSNNP TAKIKINFKG
241 VAIGDGLCDP EVMLGGYAEF LYQIGLVDEN QRIYVQNQSN LGQQYIQQKK WKEAFEVFDI LLNGDKTGTP SYIQTVSGCS
321 NYFNFMQCQE PEDQEYFGKL LSLPNIRKSI HVGNLTFHDG SMVEEYLLED IMKTIKPWLA VLMDHYRVLL YNGQLDIIVA
401 APLTERFLPT VPWGKVKEYK NAERIVWRIH DKDPEVAGYV R**QVGEFYQVI VRGGGHILPY DQPER**ALDMM DRFIMGKGWK
481 QSRYGK

Supporting Peptides:

Peptide	Uniq	-10lgP	Mass	Length	ppm	m/z	z	RT	Fraction	Scan	Source File	Area F1	Area F2	Area F3
K.NDFYATGESYAGK.Y	Y	72.10	1421.6099	13	-0.1	711.8121	2	37.11	2	F2:10004	12112021_RID_2349_DP2.raw	1.9882E5	1.3335E5	
R.QVGEFYQVIVR.G	Y	42.38	1336.7139	11	0.4	669.3645	2	51.49	1	F1:15706	12112021_RID_2349_DP1.raw	1.4401E5		
R.GGGHILPYDQPER.A	Y	40.27	1437.7001	13	1.0	719.8580	2	31.99	3	F3:8551	17112021_RID_2349_DP3.raw			8.9043E4

total 3 peptides

JAI13760.1
back to list

| Protein Coverage | Supporting Peptides |
Protein Coverage:

1 MCKITTALLC LLSLSFFLEL SSCHVRRNAI GSIFRRLKHA LPSQGDPGQP LFLTPYIESG KIEEGRQLSL VEHLPGPHVK
81 SYSGYLTVNK THNSNLFFWF FPAQVQPEKT PVLLWLQGGP GGTSMFGLFV EHGPYVVQKN LSLTERKFPW TSKFSMLYID
161 NPVGTGFSFT DDPKGYAKNE DDVGRDLYSA IIQFFQLFPH YQK**NDFYATG ESYAGK**YVPA IGYYIHSNNP TAKIKINFKG
241 VAIGDGLCDP EVMLGGYADF LYQIGLVDEN QRIYVQNQSN LGQQYIQQKK WKEAFEVFDI LLNDDKTGTP SYIQTISGCS
321 NYFNFMQCQE PEDQEYFGKL LSLPNIRKSI HVGNLTFHDG SMVEEYLLED IMKTIKPWLA VLMDHYRVLI YNGQLDIIVA
401 APLTERFLPT VPWGKVKEYK NAERIVWRIH DKDPEVAGYV R**QVGEFYQVI VRGGGHILPY DQPER**ALDMM DRFIMGKGWK
481 QSRYGK

Supporting Peptides:

Peptide	Uniq	-10lgP	Mass	Length	ppm	m/z	z	RT	Fraction	Scan	Source File	Area F1	Area F2	Area F3
K.NDFYATGESYAGK.Y	Y	72.10	1421.6099	13	-0.1	711.8121	2	37.11	2	F2:10004	12112021_RID_2349_DP2.raw	1.9882E5	1.3335E5	
R.QVGEFYQVIVR.G	Y	42.38	1336.7139	11	0.4	669.3645	2	51.49	1	F1:15706	12112021_RID_2349_DP1.raw	1.4401E5		
R.GGGHILPYDQPER.A	Y	40.27	1437.7001	13	1.0	719.8580	2	31.99	3	F3:8551	17112021_RID_2349_DP3.raw			8.9043E4

total 3 peptides

AFJ49766.1
back to list

| Protein Coverage | Supporting Peptides |
Protein Coverage:

1 MCKITTALLC LLSLSFFLEL SSCHVRRNAI GSIFRRLKHA LPSQGDPGQP LFLTPYIESG KIEEGRQLSL VEHLPGPHVK
81 SYSGYLTVNK THNSNLFFWF FPAQVQPEKT PVLLWLQGGP GGTSMFGLFV EHGPYVVQKN LSLTERKFPW TSKFSMLYID
161 NPVGTGFSFT DDPKGYAKNE DDVGRDLYSA IIQFFQLFPH YQK**NDFYATG ESYAGK**YVPA IGYYIHSNNP TAKIKINFKG
241 VAIGDGLCDP EVMLGGYADF LYQIGLVDEN QRIYVQNQSN LGQQYIQQKK WKEAFEVFDI LLNDDKTGTP SYIQTISGCS
321 NYFNFMQCQE PEDQEYFGKL LSLPNIRKSI HVGNLTFHDG SMVEEYLLED IMKTIKPWLA VLMDHYRVLI YNGQLDIIVA
401 APLTERFLPT VPWGKVKEYK NAERIVWRIH DKDPEVAGYV R**QVGEFYQVI VRGGGHILPY DQPER**ALDMM DRFIMGKGWK
481 QSRYGK

Supporting Peptides:

Peptide	Uniq	-10lgP	Mass	Length	ppm	m/z	z	RT	Fraction	Scan	Source File	Area F1	Area F2	Area F3
K.NDFYATGESYAGK.Y	Y	72.10	1421.6099	13	-0.1	711.8121	2	37.11	2	F2:10004	12112021_RID_2349_DP2.raw	1.9882E5	1.3335E5	
R.QVGEFYQVIVR.G	Y	42.38	1336.7139	11	0.4	669.3645	2	51.49	1	F1:15706	12112021_RID_2349_DP1.raw	1.4401E5		
R.GGGHILPYDQPER.A	Y	40.27	1437.7001	13	1.0	719.8580	2	31.99	3	F3:8551	17112021_RID_2349_DP3.raw			8.9043E4

total 3 peptides

JAG47096.1
back to list

| Protein Coverage | Supporting Peptides |
Protein Coverage:

1 MCKITTALLG LLSLSFFLEL SSCHVRRNAI GSIFRRLKHA LPSQGDPGQP LFLTPYIESG KIEEGRQLSL VEHLPGPHVK
81 SYSGYLTVNK THNSNLFFWF FPAQVQPEKT PVLLWLQGGP GGTSMFGLFV EHGPYVVQKN LSLTERKFPW TSKFSMLYVD
161 NPVGTGFSFT DDPKGYAKNE DDVGRDLYSA IIQFFQLFPH YQK**NDFYATG ESYAGK**YVPA IGYYIHSNNP TAKIKINFKG
241 VAIGDGLCDP EVMLGGYADF LYQIGLVDEN QRIYVQNQSN LGQQYIQQKK WKEAVEVFDI LLNDDKTGTP SYIQTISGCS
321 NYFNFMQCQE PEDQEYFGKL LSLPNIRKSI HVGNLTFHDG SMVEEYLLED IMKTIKPWLA VLMDHYRVLI YNGQLDIIVA
401 APLTERFLPT VPWGKVKEYK NAERIVWRIH DKDPEVAGYV R**QVGEFYQVI VRGGGHILPY DQPER**ALDMM DRFIMGKGWK
481 QSRYGK

Supporting Peptides:

Peptide	Uniq	-10lgP	Mass	Length	ppm	m/z	z	RT	Fraction	Scan	Source File	Area F1	Area F2	Area F3
K.NDFYATGESYAGK.Y	Y	72.10	1421.6099	13	-0.1	711.8121	2	37.11	2	F2:10004	12112021_RID_2349_DP2.raw	1.9882E5	1.3335E5	
R.QVGEFYQVIVR.G	Y	42.38	1336.7139	11	0.4	669.3645	2	51.49	1	F1:15706	12112021_RID_2349_DP1.raw	1.4401E5		
R.GGGHILPYDQPER.A	Y	40.27	1437.7001	13	1.0	719.8580	2	31.99	3	F3:8551	17112021_RID_2349_DP3.raw			8.9043E4

total 3 peptides

TRINITY_DN8397
back to list

| Protein Coverage | Supporting Peptides |
Protein Coverage:

1 MCKITTALLG LLSLSFFLEL SSCHVRRGAM GSIFRRLKYA LPSQGDPGQP LFLTPYIESG KIEEGRQLSL VEHLPGPHVK
81 SYSGYLTVNK THNSNLFFWF FPAQVQPEKT PVLLWLQGGP GGTSMFGLFV EHGPYVVQKN LSLTERKFPW TSKFSMLYID
161 NPVGTGFSFT DDPKGYAKNE DDVGRDLYSA IVQFFQLFPQ YQK**NDFYATG ESYAGK**YVPA IGYYIHSNNP TAKIKINFKG
241 VAIGDGLCDP EVMLGGYADF LYQIGLVDEK QRIYVQNQSD LGQQYIQQKK WKEAFEVFDI LLNGDKTGSP PYIETVSGCS
321 NYFNFLQCQD PEDQKYFGKL LSLPNIRKSI HVGNLTFHDG SMVEEYLIED IMKTIKPWLA VLMDHYRVLL YNGQLDIIVA
401 APLTERFLPT VPWGKVKEYK NAERIVWRIH DKDPEVAGYV R**QVGEFYQVI VRGGGHILPY DQPER**ALDMM DRFIMGKGWK
481 QSGYGK

Supporting Peptides:

Peptide	Uniq	-10lgP	Mass	Length	ppm	m/z	z	RT	Fraction	Scan	Source File	Area F1	Area F2	Area F3
K.NDFYATGESYAGK.Y	Y	72.10	1421.6099	13	-0.1	711.8121	2	37.11	2	F2:10004	12112021_RID_2349_DP2.raw	1.9882E5	1.3335E5	
R.QVGEFYQVIVR.G	Y	42.38	1336.7139	11	0.4	669.3645	2	51.49	1	F1:15706	12112021_RID_2349_DP1.raw	1.4401E5		
R.GGGHILPYDQPER.A	Y	40.27	1437.7001	13	1.0	719.8580	2	31.99	3	F3:8551	17112021_RID_2349_DP3.raw			8.9043E4

total 3 peptides

ETE69371.1
back to list

| Protein Coverage | Supporting Peptides |
Protein Coverage:

1 MCKITTALLA LLSLSFFLEL SSYHVRWSAM GSIFRRRKYT LPSQGDPGQP LFLTPYIESG KTEEGRQLSL VEHFPGPNVK
81 SYSGYLTVNM TYNSNLFFWF FPAQVGTGFS FTDDLKGYAK NEDDVGRDLY SAIVQFFQLF PDYQK**NDFYA TGESYAGK**YV
161 PAIGYYIHSN NPTAKIKINF KGVAIGDGLC DPEVMLGGYA EFLYQIGLVD EKQRLYVQNQ TNLGQQYIQQ KKWKEAFEVF
241 DILLNGDKTG SPYIQNVTGC SNYFNFLQCN EPEDQEYFGK LLSLPNIRKS IHVGNLTFHD GSMVEEHLIE DIMKTIKPWL
321 AVLMDNYRVL LYNGQLDIIV AASLTERFLP TVPWGKAKEY KNAERIVWRI HDKDPEVAGY VR**QVGEFYQV IVRGGGHILP**
401 **YDQPER**ALDM MDRFTMGKGW KQSRYGK

Supporting Peptides:

Peptide	Uniq	-10lgP	Mass	Length	ppm	m/z	z	RT	Fraction	Scan	Source File	Area F1	Area F2	Area F3
K.NDFYATGESYAGK.Y	Y	72.10	1421.6099	13	-0.1	711.8121	2	37.11	2	F2:10004	12112021_RID_2349_DP2.raw	1.9882E5	1.3335E5	
R.QVGEFYQVIVR.G	Y	42.38	1336.7139	11	0.4	669.3645	2	51.49	1	F1:15706	12112021_RID_2349_DP1.raw	1.4401E5		
R.GGGHILPYDQPER.A	Y	40.27	1437.7001	13	1.0	719.8580	2	31.99	3	F3:8551	17112021_RID_2349_DP3.raw			8.9043E4

total 3 peptides

XP_026529384.1
back to list

| Protein Coverage | Supporting Peptides |
Protein Coverage:

1 MCKITTALLA LLSLSFFLEL SSYHVQWSAM GSIFRRLKYT LPSQGDPGQP LFLTPYIESG KTEEGRQLSL VEHFPGPNVK
81 SYSGYLTVNM TYNSNLFFWF FPAQDQPEKA PVLLWLQGGP GGTSMFGLFV EHGPYVVQKN LSLTERKFPW TSKFSMLYID
161 NPVGTGFSFT DDLKGYAKNE DDVGRDLYSA IVQFFQLFPD YQK**NDFYATG ESYAGK**YVPA IGYYIHSNNP TAKIKINFKG
241 VAIGDGLCDP EVMLGGYAEF LYQIGLVDEK QRIYVQNQTN LGQQYIQQKK WKEAFEVFDI LLNGDKTGNP YIQNVTGCSN
321 YFNFLQCNEP EDQEYFGKLL SLPNIRKSIH VGNLTFHDGS MVEEHLIEDI MKTIKPWLAV LMDNYRVLLY NGQLDIIVAA
401 SLTERFLPTV PWSKAKEYKN AERIVWRIHD KDPEVAGYVH **QVGEFYQVIV RGGGHILPYD QPER**ALDMMD RFIMGKGWKQ
481 SRYGK

Supporting Peptides:

Peptide	Uniq	-10lgP	Mass	Length	ppm	m/z	z	RT	Fraction	Scan	Source File	Area F1	Area F2	Area F3
K.NDFYATGESYAGK.Y	Y	72.10	1421.6099	13	-0.1	711.8121	2	37.11	2	F2:10004	12112021_RID_2349_DP2.raw	1.9882E5	1.3335E5	
H.QVGEFYQVIVR.G	Y	42.38	1336.7139	11	0.4	669.3645	2	51.49	1	F1:15706	12112021_RID_2349_DP1.raw	1.4401E5		
R.GGGHILPYDQPER.A	Y	40.27	1437.7001	13	1.0	719.8580	2	31.99	3	F3:8551	17112021_RID_2349_DP3.raw			8.9043E4

total 3 peptides

XP_026563995.1
back to list

| Protein Coverage | Supporting Peptides |
Protein Coverage:

1 MCKITTTLLA LLSLSFFLEL SSYHVRWSAM GSIFRRLKYT LPSQGDPGQP LFLTPYIESG KTEEGRQLSL VEHFPGPNVK
81 SYSDYLTVNM TYNSNLFFWF FPAQDQPEKA PVLLWLQGGP GGTSMFGLFV EHGPYVVQKN LSLTERKFPW TSKFSMLYID
161 NPVGTGFSFT DELKGYAKNE DDVGRDLYSA IVQFFQLFPD YQK**NDFYATG ESYAGK**YVPA VGYYIHSNNP TAKIKINFKG
241 VAIGDGLCDP EVMLGGYAEF LYQIGLVDEK QRIYVQNQTN LGQQYIQQKK WKKAFEVFDI LLNGDKTGNP YIQNVTGCSN
321 YFNFLQCNEP EDQEYFGKLL SLPNIRKSIH VGNLTFHDGS IVEEHLIEDI MKTIKPWLAV LMDNYRVLLY NGQLDIIVAA
401 SLTERFLPTV PWSKAKEYKN AERIVWRIHD KDPDVAGYVR **QVGEFYQVIV RGGGHILPYD QPER**ALDMMD RFIMGKGWKQ
481 SRYGK

Supporting Peptides:

Peptide	Uniq	-10lgP	Mass	Length	ppm	m/z	z	RT	Fraction	Scan	Source File	Area F1	Area F2	Area F3
K.NDFYATGESYAGK.Y	Y	72.10	1421.6099	13	-0.1	711.8121	2	37.11	2	F2:10004	12112021_RID_2349_DP2.raw	1.9882E5	1.3335E5	
R.QVGEFYQVIVR.G	Y	42.38	1336.7139	11	0.4	669.3645	2	51.49	1	F1:15706	12112021_RID_2349_DP1.raw	1.4401E5		
R.GGGHILPYDQPER.A	Y	40.27	1437.7001	13	1.0	719.8580	2	31.99	3	F3:8551	17112021_RID_2349_DP3.raw			8.9043E4

total 3 peptides

P86497.1
back to list

| Protein Coverage | Supporting Peptides |
Protein Coverage:

1 VIGGAECDIN EHPSLALIYS TSMRFHCAGT LLNQEWV**SFT MWDKDIMLIR** TLCAGVLEGG KDTCLAHPCA QPLLPAFYTK
81 VFDYIPWIK

Supporting Peptides:

Peptide	Uniq	-10lgP	Mass	Length	ppm	m/z	z	RT	Fraction	Scan	Source File	Area F1	Area I[illegible]
M.WDKDIMLIR.T	N	59.98	1188.6324	9	0.8	595.3240	2	47.61	2	F2:13483	12112021_RID_2349_DP2.raw		2.0105[illegible]
M.WDKDIM(+15.99)LIR.T	N	54.14	1204.6274	9	0.6	603.3214	2	42.22	2	F2:11819	12112021_RID_2349_DP2.raw		1.4264[illegible]
V.SFTM(+15.99)WDKDIMLIR.T	Y	52.39	1670.8160	13	0.4	836.4156	2	57.93	2	F2:16952	12112021_RID_2349_DP2.raw		1.0184[illegible]
F.TMWDKDIMLIR.T	N	50.40	1420.7207	11	0.6	711.3680	2	53.82	3	F3:15851	17112021_RID_2349_DP3.raw		
W.DKDIMLIR.T	N	44.22	1002.5532	8	-0.5	502.2836	2	37.38	3	F3:10341	17112021_RID_2349_DP3.raw		
V.SFTM(+15.99)WDKDIM(+15.99)LIR.T	Y	42.80	1686.8109	13	0.4	844.4131	2	54.20	2	F2:15770	12112021_RID_2349_DP2.raw		4.4011[illegible]
W.DKDIM(+15.99)LIR.T	N	39.37	1018.5481	8	0.3	510.2815	2	31.54	3	F3:8358	17112021_RID_2349_DP3.raw		

JAG46182.1
back to list

| Protein Coverage | Supporting Peptides |
Protein Coverage:

```
  1 MYWHFPLLFA ALISTYLCFP QNHLSLEELG SDIGIQVFNQ LVKTRAGENV VVSPHGIASV LGMLQLGADS KTKKQLTTVM
 81 RYSVNGAGKV LKKINKAIVS RKNKDIVKIA NVVFANSGLK MEASFISRNS EVFQCPVKNI NFKDPNAASD TMNQWVKNKT
161 EGMIDSVLTP DDIDSALTRL VVINAVYFKG LWRSRFQPEN TKKRTFNEGD GKTYQVPMLA QLSVFRCGTT STPNDLWYNT
241 IELPYHGESI SMLIALPAES STPLSAIIPH ISTKTIQSWM TTMVQKRVQV ILPKFTAEAE TDLKEPLKEL GIRDMFDQSR
321 ANFAKITRSE NIYVSQLLQK AKIEVSEDGT KASAATTAIV TARSSPPWFV VDRPFLFFIR HNPTGAVLFM GQITKP
```

Supporting Peptides:

Peptide	Uniq	-10lgP	Mass	Length	ppm	m/z	z	RT	Fraction	Scan	Source File	Area F1	Area F2	Area F3	Area F4	Ar F5
K.IANVVFANSGLK.M	Y	77.60	1231.6924	12	0.3	616.8536	2	44.26	2	F2:12431	12112021_RID_2349_DP2.raw		7.2043E4			
R.SENIYVSQLLQK.A	Y	41.15	1420.7561	12	0.3	711.3856	2	49.58	2	F2:14262	12112021_RID_2349_DP2.raw		4.7741E4			
													**total 2 peptides**			

XP_015674504.1
back to list

| Protein Coverage | Supporting Peptides |
Protein Coverage:

```
  1 MYWHFPLLFA ALISTYACFP QNHLSLEELG SDIGIQVFNQ LVKTRAGDNV VVSPHGIASV LGMLQLGADS KTKKQLTTVM
 81 RYSVNGAGKV LKKINKAIVS RKNKDIVKIA NVVFANSGLK MEAPFISRNS EVFQCPVKNI NFKDPNAASD TMNQWVKNKT
161 EGMIDSVLTP GDIDSALTRL VVINAVYFKG LWRSRFQPEN TKKRTFNEGD GKTYQVPMLA QLSVFRCGTT STPNDLWYNT
241 IELPYHGESI SMLIALPAES STPLSAIIPH ISTKTIQSWM TTMVQKRVQV ILPKFTAEAE TDLKEPLKEL GIRDMFDQSR
321 ANFAKITRSE NIYVSQLLQK AKIEVSEDGT KASAATTAIV TARSSPPWFV VDRPFLFFIR HNPTGAVLFM GQITKP
```

Supporting Peptides:

Peptide	Uniq	-10lgP	Mass	Length	ppm	m/z	z	RT	Fraction	Scan	Source File	Area F1	Area F2	Area F3	Area F4	Ar F5
K.IANVVFANSGLK.M	Y	77.60	1231.6924	12	0.3	616.8536	2	44.26	2	F2:12431	12112021_RID_2349_DP2.raw		7.2043E4			
R.SENIYVSQLLQK.A	Y	41.15	1420.7561	12	0.3	711.3856	2	49.58	2	F2:14262	12112021_RID_2349_DP2.raw		4.7741E4			
													**total 2 peptides**			

JAG43702.1
back to list

| Protein Coverage | Supporting Peptides |
Protein Coverage:

```
  1 MYWHFPLLFA ALISTYLCFP QNHLSLEELG SDIGIQVFNQ LVKTRAGENV VVSPHGIASV LGMLQLGADS KTKKQLTTVM
 81 RYSVNGAGKV LKKINKAIVS RKNKDIVKIA NVVFANSGLK MEASFISRNS EVFQCPVKNI NFKDPNAASD TMNQWVKNKT
161 EGMIDSVLTP DDIDSALTRL VVINAVYFKG LWRSRFQPEN TKKRTFNEGD GKTYQVPMLA QLSVFRCGTT STPNDLWYNT
241 IELPYHGESI SMLIALPAES STPLSAIIPH ISTKTIQSWM TTMVQKRVQV ILPKFTAEAE TDLKEPLKEL GIRDMFDQSR
321 ANFAKITRSE NIYVSQLLQK AKIEVSEDGT KASAATTAIV TARSSPPWFV VDRPFLFFIR HNPTGAVLFM GQITKP
```

Supporting Peptides:

Peptide	Uniq	-10lgP	Mass	Length	ppm	m/z	z	RT	Fraction	Scan	Source File	Area F1	Area F2	Area F3	Area F4	Ar F5
K.IANVVFANSGLK.M	Y	77.60	1231.6924	12	0.3	616.8536	2	44.26	2	F2:12431	12112021_RID_2349_DP2.raw		7.2043E4			
R.SENIYVSQLLQK.A	Y	41.15	1420.7561	12	0.3	711.3856	2	49.58	2	F2:14262	12112021_RID_2349_DP2.raw		4.7741E4			
													**total 2 peptides**			

JAA96331.1
back to list

| Protein Coverage | Supporting Peptides |
Protein Coverage:

```
  1 MYWHFPLLFA ALISTYLCFP QNHLSLEELG SDIGIQVFNQ LVKTRAGENV VVSPHGIASV LGMLQLGADS KTKKQLTTVM
 81 RYSVNGAGKV LKKINKAIVS RKNKDIVKIA NVVFANSGLK MEASFISRNS EVFQCPVKNI NFKDPNAASD TMNQWVKNKT
161 EGMIDSVLTP DDIDSALTRL VVINAVYFKG LWRSRFQPEN TKKRTFNEGD GKTYQVPMLA QLSVFRCGTT STPNDLWYNT
241 IELPYHGESI SMLIALPAES STPLSAIIPH ISTKTIQSWM TTMVQKRVQV ILPKFTAEAE TDLKEPLKEL GIRDMFDQSR
321 ANFAKITRSE NIYVSQLLQK AKIEVSEDGT KASAATTAIV TARSSPPWFV VDRPFLFFIR HNPTGAVLFM GQITKP
```

Supporting Peptides:

Peptide	Uniq	-10lgP	Mass	Length	ppm	m/z	z	RT	Fraction	Scan	Source File	Area F1	Area F2	Area F3	Area F4	Ar F5

Peptide	Uniq	-10lgP	Mass	Length	ppm	m/z	z	RT	Fraction	Scan	Source File	Area F1	Area F2	Area F3	Area F4	Ar F5
K.IANVVFANSGLK.M	Y	77.60	1231.6924	12	0.3	616.8536	2	44.26	2	F2:12431	12112021_RID_2349_DP2.raw		7.2043E4			
R.SENIYVSQLLQK.A	Y	41.15	1420.7561	12	0.3	711.3856	2	49.58	2	F2:14262	12112021_RID_2349_DP2.raw		4.7741E4			

total 2 peptides

XP_015674505.1
back to list

| Protein Coverage | Supporting Peptides |
Protein Coverage:

1 MYWHFPLLFA ALISTYACFP QNHLSLEELG SDIGIQVFNQ LVKTRAGDNV VVSPHGIASV LGMLQLGADS KTKKQLTTVM
81 RYSVNGAGKV LKKINKAIVS RKNKDIVK**IA NVVFANSGLK** MEAPFISRNS EVFQCPVKNI NFKDPNAASD TMNQWVKNKT
161 EGMIDSVLTP GDIDSALTRL VVINAVYFKG LWRSRFQPEN TKKRTFNEGD GKTYQVPMLA QLSVFRCGTT STPNDLWYNT
241 IELPYHGESI SMLIALPAES STPLSAIIPH ISTKTIQSWM TTMVQKRVQV ILPKFTAEAE TDLKEPLKEL GIRDMFDQSR
321 ANFAKITR**SE NIYVSQLLQK** AKIEVSEDGT KASAATTAIV TARSSPPWFV VDRPFLFFIR HNPTGAVLFM GQITKP

Supporting Peptides:

Peptide	Uniq	-10lgP	Mass	Length	ppm	m/z	z	RT	Fraction	Scan	Source File	Area F1	Area F2	Area F3	Area F4	Ar F5
K.IANVVFANSGLK.M	Y	77.60	1231.6924	12	0.3	616.8536	2	44.26	2	F2:12431	12112021_RID_2349_DP2.raw		7.2043E4			
R.SENIYVSQLLQK.A	Y	41.15	1420.7561	12	0.3	711.3856	2	49.58	2	F2:14262	12112021_RID_2349_DP2.raw		4.7741E4			

total 2 peptides

JAV49839.1
back to list

| Protein Coverage | Supporting Peptides |
Protein Coverage:

1 MYWHFPLLFA ALISTYFCFP QNHLSLEELG SDIGIQVFNQ LVKTRAGDNV VVSPHGIASV LGMLQLGADS KTKKQLTTVM
81 RYSVNGAGKV LKKINKAIVS RKNKDIVK**IA NVVFANSGLK** MEAPFISRNS EVFQCPVKNI NFKDPNAASD TMNQWVKNKT
161 EGMIDSVLTP DDIDSALTRL VVINAVYFKG LWRSRFQPEN TKKRTFNEGD GKTYQVPMLA QLSVFRCGTT STPNDLWYNT
241 IELPYHGESI SMLIALPAES STPLSAIIPH ISTKTIQSWM TTMVQKRVQV ILPKFTAEAE TDLKEPLKEL GIRDMFDQSR
321 ANFAKITR**SE NIYVSQLLQK** AKIEVSEDGT KASAATTAIV TARSSPPWFV VDRPFLFFIR HNPTGAVLFM GQITKP

Supporting Peptides:

Peptide	Uniq	-10lgP	Mass	Length	ppm	m/z	z	RT	Fraction	Scan	Source File	Area F1	Area F2	Area F3	Area F4	Ar F5
K.IANVVFANSGLK.M	Y	77.60	1231.6924	12	0.3	616.8536	2	44.26	2	F2:12431	12112021_RID_2349_DP2.raw		7.2043E4			
R.SENIYVSQLLQK.A	Y	41.15	1420.7561	12	0.3	711.3856	2	49.58	2	F2:14262	12112021_RID_2349_DP2.raw		4.7741E4			

total 2 peptides

TRINITY_DN3815
back to list

| Protein Coverage | Supporting Peptides |
Protein Coverage:

1 MYWHFPLLFA ALISTYVCFP QNHLSLEELG SDIGIQVFNQ LVKTRADDNV VVSPHGIASV LGMLQLGADS KTKKQLTTVM
81 RYSVNGAGKV LKKINKAIVS RKNKDIVK**IA NVVFANSGLK** MEAPFISRNS EVFQCPVKNI NFKDPNAASD TMNQWVKNKT
161 QGMIDSVLTP DDIDSALTRL VVINAVYFKG LWRSRFQPEN TKKRTFNGGD GKTYQVPMLA QLSVFRCGTT STPNDLWYNT
241 IELPYHGESI SMLIALPAQI STPLSAIIPH ISTKTIQSWM TTMVQKRVQV ILPKFTAEAE TDLKEPLKEL GIRDMFDQSR
321 ANFAKITR**SE NIYVSQLLQK** AKIEVSEDGT KASAATTAIV TARSSPPWFV VDRPFLFFIR HNPTGAVLFM GQITKP

Supporting Peptides:

Peptide	Uniq	-10lgP	Mass	Length	ppm	m/z	z	RT	Fraction	Scan	Source File	Area F1	Area F2	Area F3	Area F4	Ar F5
K.IANVVFANSGLK.M	Y	77.60	1231.6924	12	0.3	616.8536	2	44.26	2	F2:12431	12112021_RID_2349_DP2.raw		7.2043E4			
R.SENIYVSQLLQK.A	Y	41.15	1420.7561	12	0.3	711.3856	2	49.58	2	F2:14262	12112021_RID_2349_DP2.raw		4.7741E4			

total 2 peptides

TRINITY_DN3815
back to list

| Protein Coverage | Supporting Peptides |
Protein Coverage:

1 MYWHFPLLFA ALISTYVCFP QNHLSLEELG SDIGIQVFNQ LVKTRADDNV VVSPHGIASV LGMLQLGADS KTKKQLTTVM
81 RYSVNGAGKV LKKINKAIVS RKNKDIVK**IA NVVFANSGLK** MEAPFISRNS EVFQCPVKNI NFKDPNAASD TMNQWVKNKT
161 QGMIDSVLTP DDIDSALTRL VVINAVYFKG LWRSRFQPEN TKKRTFNGGD GKTYQVPMLA QLSVFRCGTT STPNDLWYNT
241 IELPYHGESI SMLIALPAQI STPLSAIIPH ISTKTIQSWM TTMVQKRVQV ILPKFTAEAE TDLKEPLKEL GIRDMFDQSR
321 ANFAKITR**SE NIYVSQLLQK** AKIEVSEDGT KASAATTAIV TARSSPPWFV VDRPFLFFIR HNPTGAVLFM GQITKP

Supporting Peptides:

Peptide	Uniq	-10lgP	Mass	Length	ppm	m/z	z	RT	Fraction	Scan	Source File	Area F1	Area F2	Area F3	Area F4	Ar F5
K.IANVVFANSGLK.M	Y	77.60	1231.6924	12	0.3	616.8536	2	44.26	2	F2:12431	12112021_RID_2349_DP2.raw		7.2043E4			
R.SENIYVSQLLQK.A	Y	41.15	1420.7561	12	0.3	711.3856	2	49.58	2	F2:14262	12112021_RID_2349_DP2.raw		4.7741E4			

total 2 peptides

TRINITY_DN3815
back to list

| Protein Coverage | Supporting Peptides |
Protein Coverage:

1 MYWHFPLLFA ALISTYVCFP QNHLSLEELG SDIGIQVFNQ LVKTRADDNV VVSPHGIASV LGMLQLGADS KTKKQLTTVM
81 RYSVNGAGKV LKKINKAIVS RKNKDIVK**IA NVVFANSGLK** MEAPFISRNS EVFQCPVKNI NFKDPNAASD TMNQWVKNKT
161 QGMIDSVLTP DDIDSALTRL VVINAVYFKG LWRSRFQPEN TKKRTFNGGD GKTYQVPMLA QLSVFRCGTT STPNDLWYNT
241 IELPYHGESI SMLIALPAQI STPLSAIIPH ISTKTIQSWM TTMVQKRVQV ILPKFTAEAE TDLKEPLKEL GIRDMFDQSR
321 ANFAKITR**SE NIYVSQLLQK** AKIEVSEDGT KASAATTAIV TARSSPPWFV VDRPFLFFIR HNPTGAVLFM GQITKP

Supporting Peptides:

Peptide	Uniq	-10lgP	Mass	Length	ppm	m/z	z	RT	Fraction	Scan	Source File	Area F1	Area F2	Area F3	Area F4	Ar F5
K.IANVVFANSGLK.M	Y	77.60	1231.6924	12	0.3	616.8536	2	44.26	2	F2:12431	12112021_RID_2349_DP2.raw		7.2043E4			
R.SENIYVSQLLQK.A	Y	41.15	1420.7561	12	0.3	711.3856	2	49.58	2	F2:14262	12112021_RID_2349_DP2.raw		4.7741E4			

total 2 peptides

TRINITY_DN3815
back to list

| Protein Coverage | Supporting Peptides |
Protein Coverage:

1 MYWHFPLLFA ALISTYVCFP QNHLSLEELG SDIGIQVFNQ LVKTRADDNV VVSPHGIASV LGMLQLGADS KTKKQLTTVM
81 RYSVNGAGKV LKKINKAIVS RKNKDIVK**IA NVVFANSGLK** MEAPFISRNS EVFQCPVKNI NFKDPNAASD TMNQWVKNKT
161 QGMIDSVLTP DDIDSALTRL VVINAVYFKG LWRSRFQPEN TKKRTFNGGD GKTYQVPMLA QLSVFRCGTT STPNDLWYNT
241 IELPYHGESI SMLIALPAQI STPLSAIIPH ISTKTIQSWM TTMVQKRVQV ILPKFTAEAE TDLKEPLKEL GIRDMFDQSR
321 ANFAKITR**SE NIYVSQLLQK** AKIEVSEDGT KASAATTAIV TARSSPPWFV VDRPFLFFIR HNPTGAVLFM GQITKP

Supporting Peptides:

Peptide	Uniq	-10lgP	Mass	Length	ppm	m/z	z	RT	Fraction	Scan	Source File	Area F1	Area F2	Area F3	Area F4	Ar F5
K.IANVVFANSGLK.M	Y	77.60	1231.6924	12	0.3	616.8536	2	44.26	2	F2:12431	12112021_RID_2349_DP2.raw		7.2043E4			
R.SENIYVSQLLQK.A	Y	41.15	1420.7561	12	0.3	711.3856	2	49.58	2	F2:14262	12112021_RID_2349_DP2.raw		4.7741E4			

total 2 peptides

TRINITY_DN3815
back to list

| Protein Coverage | Supporting Peptides |
Protein Coverage:

1 MYWHFPLLFA ALISTYVCFP QNHLSLEELG SDIGIQVFNQ LVKTRADDNV VVSPHGIASV LGMLQLGADS KTKKQLTTVM
81 RYSVNGAGKV LKKINKAIVS RKNKDIVK**IA NVVFANSGLK** MEAPFISRNS EVFQCPVKNI NFKDPNAASD TMNQWVKNKT
161 QGMIDSVLTP DDIDSALTRL VVINAVYFKG LWRSRFQPEN TKKRTFNGGD GKTYQVPMLA QLSVFRCGTT STPNDLWYNT
241 IELPYHGESI SMLIALPAQI STPLSAIIPH ISTKTIQSWM TTMVQKRVQV ILPKFTAEAE TDLKEPLKEL GIRDMFDQSR
321 ANFAKITR**SE NIYVSQLLQK** AKIEVSEDGT KASAATTAIV TARSSPPWFV VDRPFLFFIR HNPTGAVLFM GQITKP

Supporting Peptides:

Peptide	Uniq	-10lgP	Mass	Length	ppm	m/z	z	RT	Fraction	Scan	Source File	Area F1	Area F2	Area F3	Area F4	Ar F5
K.IANVVFANSGLK.M	Y	77.60	1231.6924	12	0.3	616.8536	2	44.26	2	F2:12431	12112021_RID_2349_DP2.raw		7.2043E4			

total 2 peptides

Peptide	Uniq	-10lgP	Mass	Length	ppm	m/z	z	RT	Fraction	Scan	Source File	Area F1	Area F2	Area F3	Area F4	Area F5
R.SENIYVSQLLQK.A	Y	41.15	1420.7561	12	0.3	711.3856	2	49.58	2	F2:14262	12112021_RID_2349_DP2.raw		4.7741E4			

total 2 peptides

TRINITY_DN28984
back to list

| Protein Coverage | Supporting Peptides |
Protein Coverage:

1 ETKPRELYGR RLPLREYLVL VPVVVPTETS RKICVQLNHL NESVTLSITL EYGLQNRSLL REEVSKKDLF QCTEFQIPKW
81 ERSFNSHIAL FTVEVTGATL RYLDRKRVYI ENQESLTFVQ TDKPIYKPGH KVLFRAVSLT KEFIPVTEKV PLIYIEDPKR
161 NRLFHWKDVE LKGGLVQLEF PLSSEPALGT YKVVVQKDSG RKVEHPYTVD EYELPKFEVT VKSTPVITIL DNKLDVTVCG
241 KYTFGKPVPG LVNIRVCRKF SFFGSTCYGE ESKAICEEFH GKADIHGCFS QEVDLKIFQL KRNGFQMQIQ TEGTITEEGT
321 EIVLTGSGTT QITSTLSVVS FENVDAYYKP GLPLSGEIKL VDGRTKPIAN QVVELQVSGV KNSTYHNTDD QGRARFSIDT
401 SDFTNDAVQL TATYKQLDYC YDSNWISPQH QTAFTSLSRF YSPSQSYLKI ERHLGSLTCG EHAVIPVHYI LKPELIKDKE
481 VVFYYLVVAR GDIVKTGTHP LKGKESKGTF NLNLLVDIKS APLARVLLYT ILDNGELVVH SADFPVEPCF ANKVNLRFAE
561 PEGLPGSSIN LQLSAAQNSL CAIHAVDQSV FLLKPEAELS PRTVYDLLPV KDLRGYYYKS ENLEDPDTNP CVPPRKIIVD
641 GIRYQTDTYS YGEGDAYTIL KDIGFKTFTS TQIHKPNICS SPDVYYSRAY DVAGGMAAPL AAELKRES**AE AVETTIR**SFF
721 PETWLWSIKS LSSETEVTLP VTIPDTITEW RAGAFCLSPV VGFGLAKTTF LKAMQPFFVE VTQPYSVVRG EAFNLK**ATVF**
801 **SYQK**RPIQVS LSLLLSPDYD ASPVEKEQDS YCLPVDGRKT VSWKVTPKTL GKVNFTITAE ALKSAQLCGN EIVEVPTKGQ
881 RDIVIKSLLV EPEGIEKEVV INNLLCGDKG TPSATISLKV PEKVVEKSAR AFFSVLGDIL SSAIQNVHQL IRLPTGCGEQ
961 NMALLAPNIY ILDYLNATGQ LNEDMKSKAI GFLVAGYQRQ LNYKHADGSY STFGERNNEP GNVWLTAFVL KCLSQMKRYI
1041 PVEERHLIDA QHFLALKQKP NGCFQITGTL LNNALKGGVD DEVTLSTYIA IALLEMSLPV THSVLRNALF CLETASQQKD
1121 IPVYNLALLG YAFALAHKQD KVAEILHTLQ GKAVKGEDGS IHWERPAKPE AKKPLYYESR ASSAEVELNS YMLLTYLKR**N**
1201 **PSPTQDDLAM ASK**IVTWLTK QQNPSGGFSS TQDTVVALQA LSLYAAATFS NSAEGIRVTL KSGENNLKEF QVDNSNRILL
1281 QTQDLPTVPG EYSAVVTGKG CTYVQTTLRY NVLPHPEDDP FAVTVDTIPE TCVGVKAHKE FEIVMNVSYT GKRPASNMAI
1361 INVKMVSGFV PNAPSLKKLK KQAPIQQVEV TATNVMLYLE KVTNVTIPIS FTVERSFDIQ NQRPAFVHMY DYYEDESTVV
1441 PYSAPCSKAK FGNA

Supporting Peptides:

Peptide	Uniq	-10lgP	Mass	Length	ppm	m/z	z	RT	Fraction	Scan	Source File	Area F1	Area F2	Area F3	Area F4	Area F5
R.NPSPTQDDLAMASK.I	Y	58.60	1473.6769	14	1.1	737.8466	2	32.86	7	F7:8878	17112021_RID_2349_DP7.raw					
S.AEAVETTIR.S	Y	52.94	988.5189	9	-0.1	495.2667	2	26.18	7	F7:6550	17112021_RID_2349_DP7.raw					
K.ATVFSYQK.R	Y	50.25	942.4811	8	-0.2	472.2477	2	31.69	7	F7:8446	17112021_RID_2349_DP7.raw					

total 3 peptides

TRINITY_DN28984
back to list

| Protein Coverage | Supporting Peptides |
Protein Coverage:

1 MGKNRLLAGP CLFLLGLFLL PGGTSASTPK PEYLVLVPVV VPTETSRKIC VQLNHLNESV TLSITLEYGL QNRSLLREEV
81 SKKDLFQCTE FQIPKWERSF NSHIALFTVE VTGATLRYLD RKRVYIENQE SLTFVQTDKP IYKPGHKVLF RAVSLTKEFI
161 PVTEKVPLIY IEDPKRNRLF HWKDVELKGG LVQLEFPLSS EPALGTYKVV VQKDSGRKVE HPYTVDEYEL PKFEVTVKST
241 PVITILDNKL DVTVCGKYTF GKPVPGLVNI RVCRKFSFFG STCYGEESKA ICEEFHGKAD IHGCFSQEVD LKIFQLKRNG
321 FQMQIQTEGT ITEEGTEIVL TGSGTTQITS TLSVVSFENV DAYYKPGLPL SGEIKLVDGR TKPIANQVVE LQVSGVKNST
401 YHNTDDQGRA RFSIDTSDFT NDAVQLTATY KQLDYCYDSN WISPQHQTAF TSLSRFYSPS QSYLKIERHL GSLTCGEHAV
481 IPVHYILKPE LIKDKEVVFY YLVVARGDIV KTGTHPLKGK ESKGTFNLNL LVDIKSAPLA RVLLYTILDN GELVVHSADF
561 PVEPCFANKV NLRFAEPEGL PGSSINLQLS AAQNSLCAIH AVDQSVFLLK PEAELSPRTV YDLLPVKDLR GYYYKSENLE
641 DPDTNPCVPP RKIIVDGIRY QTDTYSYGEG DAYTILKDIG FKTFTSTQIH KPNICSSPDV YYSRAYDVAG GMAAPLAAEL
721 KRES**AEAVET TIR**SFFPETW LWSIKSLSSE TEVTLPVTIP DTITEWRAGA FCLSPVVGFG LAKTTFLKAM QPFFVEVTQP
801 YSVVRGEAFN LK**ATVFSYQK** RPIQVSLSLL LSPDYDASPV EKEQDSYCLP VDGRKTVSWK VTPKTLGKVN FTITAEALKS
881 AQLCGNEIVE VPTKGQRDIV IKSLLVEPEG IEKEVVINNL LCGDKGTPSA TISLKVPEKV VEKSARAFFS VLGDILSSAI
961 QNVHQLIRLP TGCGEQNMAL LAPNIYILDY LNATGQLNED MKSKAIGFLV AGYQRQLNYK HADGSYSTFG ERNNEPGNVW
1041 LTAFVLKCLS QMKRYIPVEE RHLIDAQHFL ALKQKPNGCF QITGTLLNNA LKGGVDDEVT LSTYIAIALL EMSLPVTHSV
1121 LRNALFCLET ASQQKDIPVY NLALLGYAFA LAHKQDKVAE ILHTLQGKAV KGEDGSIHWE RPAKPEAKKP LYYESRASSA
1201 EVELNSYMLL TYLKR**NPSPT QDDLAMASK**I VTWLTKQQNP SGGFSSTQDT VVALQALSLY AAATFSNSAE GIRVTLKSGE
1281 NNLKEFQVDN SNRILLQTQD LPTVPGEYSA VVTGKGCTYV QTTLRYNVLP HPEDDPFAVT VDTIPETCVG VKAHKEFEIV
1361 MNVSYTGKRP ASNMAIINVK MVSGFVPNAP SLKKLKKQAP IQQVEVTATN VMLYLEKVTN VTIPISFTVE RSFDIQNQRP
1441 AFVHMYDYYE DESTVVPYSA PCSKAKFGNA

Supporting Peptides:

Peptide	Uniq	-10lgP	Mass	Length	ppm	m/z	z	RT	Fraction	Scan	Source File	Area F1	Area F2	Area F3	Area F4	Area F5	Area
R.NPSPTQDDLAMASK.I	Y	58.60	1473.6769	14	1.1	737.8466	2	32.86	7	F7:8878	17112021_RID_2349_DP7.raw						
S.AEAVETTIR.S	Y	52.94	988.5189	9	-0.1	495.2667	2	26.18	7	F7:6550	17112021_RID_2349_DP7.raw						
K.ATVFSYQK.R	Y	50.25	942.4811	8	-0.2	472.2477	2	31.69	7	F7:8446	17112021_RID_2349_DP7.raw						

total 3 peptides

JAV50910.1
back to list

| Protein Coverage | Supporting Peptides |
Protein Coverage:

1 MAEEKPQVEL FVKAGSDGAK IGNCPFSQRL FMVLWLK**GVT FNVTTVDTK**R RTEAVQKLCP GGQLPFLMYG TEVHTDTNKI
81 EEFLEEVLCP PKYPK**LAANN PESNTAGLDV FAK**FSAYIKN SNPSLNANLE KGLLKALKVL DVYLMTPQPE EVDENSAEDE
161 GQSNRKFLDG DELTLADCNL LPKLHIVKVV CKKYRNFTIP EEFCGIHRYL KNAYAREEFA STCPDDEEIE LAYELVAKAL
241 K

Supporting Peptides:

Peptide	Uniq	-10lgP	Mass	Length	ppm	m/z	z	RT	Fraction	Scan	Source File	Area F1	Area F2	Area F3	Are
K.LAANNPESNTAGLDVFAK.F	Y	72.78	1830.9111	18	1.0	916.4637	2	47.50	4	F4:13719	17112021_RID_2349_DP4.raw				4.70
K.GVTFNVTTVDTK.R	Y	48.38	1280.6613	12	1.0	641.3386	2	41.97	4	F4:11922	17112021_RID_2349_DP4.raw				6.49

total 2 peptides

JAI10138.1
back to list

| Protein Coverage | Supporting Peptides |
Protein Coverage:

1 MAEEKPQVEL FVKAGSDGAK IGNCPFSQRL FMVLWLK**GVT FNVTTVDTK**R RTEAVQKLCP GGQLPFLMYG TEVHTDTNKV
81 EEFLEEVLCP PKYPK**LAANN PESNTAGLDV FAK**FSAYIKN SNPSLNANLE KGLLKALKVL DVYLMTPQPE EVDENSAEDE
161 GQSNRKFLDG NELTLADCNL LPKLHIVKVV CKKYRDFTIP EEFRGIHRYL KNAYAREEFS STCPDDEEIE LAYELVAKAL
241 K

Supporting Peptides:

Peptide	Uniq	-10lgP	Mass	Length	ppm	m/z	z	RT	Fraction	Scan	Source File	Area F1	Area F2	Area F3	Are
K.LAANNPESNTAGLDVFAK.F	Y	72.78	1830.9111	18	1.0	916.4637	2	47.50	4	F4:13719	17112021_RID_2349_DP4.raw				4.70
K.GVTFNVTTVDTK.R	Y	48.38	1280.6613	12	1.0	641.3386	2	41.97	4	F4:11922	17112021_RID_2349_DP4.raw				6.49

total 2 peptides

JAB54484.1
back to list

| Protein Coverage | Supporting Peptides |
Protein Coverage:

```
  1 MAEEKPQVEL FVKAGSDGAK IGNCPFSQRL FMVLWLKGVT FNVTTVDTKR RTEAVQKLCP GGQLPFLMYG TEVHTDTNKV
 81 EEFLEEVLCP PKYPKLAANN PESNTAGLDV FAKFSAYIKN SNPSLNANLE KGLLKALKVL DVYLMTPQPE EVDENSAEDE
161 GQSNRKFLDG NELTLADCNL LPKLHIVKVV CKKYRDFTIP EEFRGIHRYL KNAYAREEFS STCPDDEEIE LAYELVAKAL
241 K
```

Supporting Peptides:

Peptide	Uniq	-10lgP	Mass	Length	ppm	m/z	z	RT	Fraction	Scan	Source File	Area F1	Area F2	Area F3	Are
K.LAANNPESNTAGLDVFAK.F	Y	72.78	1830.9111	18	1.0	916.4637	2	47.50	4	F4:13719	17112021_RID_2349_DP4.raw				4.70
K.GVTFNVTTVDTK.R	Y	48.38	1280.6613	12	1.0	641.3386	2	41.97	4	F4:11922	17112021_RID_2349_DP4.raw				6.49

total 2 peptides

XP_026541233.1
back to list

| Protein Coverage | Supporting Peptides |
Protein Coverage:

```
  1 MAEEKPQVEL FVKAGSDGAK IGNCPFSQRL FMVLWLKGVT FNVTTVDTKR RTEAVQKLCP GGQLPFLMYG TEVHTDTNKV
 81 EEFLEEVLCP PKYPKLAANN PESNTAGLDV FAKFSAYIKN SNPSLNANLE KGLLKALKVL DVYLMTPQPE EVDENSAEDE
161 GQSNRKFLDG NELTLADCNL LPKLHIVKVV CKKYRDFTIP EEFRGIHRYL KNAYAREEFS STCPDDEEIE LAYELVAKAL
241 KA
```

Supporting Peptides:

Peptide	Uniq	-10lgP	Mass	Length	ppm	m/z	z	RT	Fraction	Scan	Source File	Area F1	Area F2	Area F3	Are
K.LAANNPESNTAGLDVFAK.F	Y	72.78	1830.9111	18	1.0	916.4637	2	47.50	4	F4:13719	17112021_RID_2349_DP4.raw				4.70
K.GVTFNVTTVDTK.R	Y	48.38	1280.6613	12	1.0	641.3386	2	41.97	4	F4:11922	17112021_RID_2349_DP4.raw				6.49

total 2 peptides

XP_026576189.1
back to list

| Protein Coverage | Supporting Peptides |
Protein Coverage:

```
  1 MAEEKPQVEL FVKAGSDGAK IGNCPFSQRL FMVLWLKGVT FNVTTVDTKR RTEAVQKLCP GGQLPFLMYG TEVHTDTNKI
 81 EEFLEEVLCP PKYPKLAANN PESNTAGLDV FAKFSAYIKN SNPSLNANLE KGLLKALKVL DVYLMTPQPE EVDENSAEDE
161 GQSNRKFLDG NELTLADCNL LPKLHIVKVV CKKYRDFTIP EEFRGIHRYL KNAYAREEFS STCPDDEEIE LAYELVAKAL
241 KA
```

Supporting Peptides:

Peptide	Uniq	-10lgP	Mass	Length	ppm	m/z	z	RT	Fraction	Scan	Source File	Area F1	Area F2	Area F3	Are
K.LAANNPESNTAGLDVFAK.F	Y	72.78	1830.9111	18	1.0	916.4637	2	47.50	4	F4:13719	17112021_RID_2349_DP4.raw				4.70
K.GVTFNVTTVDTK.R	Y	48.38	1280.6613	12	1.0	641.3386	2	41.97	4	F4:11922	17112021_RID_2349_DP4.raw				6.49

total 2 peptides

LAB45561.1
back to list

| Protein Coverage | Supporting Peptides |
Protein Coverage:

```
  1 TWGPPAARLS FAAPWVHASL PRLNHAPSVE SSSSSSAMAE EKPQVELFVK AGSDGAKIGN CPFSQRLFMV LWLKGVTFNV
 81 TTVDTKRRTE AVQKLCPGGQ LPFLMYGTEV HTDTNKVEEF LEEVLCPPKY PKLAANNPES NTAGLDVFAK FSAYIKNSNP
161 SLNANLEKGL LKALKVLDVY LMTPQPEEVD ENSAEDEGQS NRKFLDGNEL TLADCNLLPK LHIVKVVCKK YRDFTIPEEF
241 RGIHRYLKNA YAREEFSSTC PDDEEIELAY ELVAKALK
```

Supporting Peptides:

Peptide	Uniq	-10lgP	Mass	Length	ppm	m/z	z	RT	Fraction	Scan	Source File	Area F1	Area F2	Area F3	Are
K.LAANNPESNTAGLDVFAK.F	Y	72.78	1830.9111	18	1.0	916.4637	2	47.50	4	F4:13719	17112021_RID_2349_DP4.raw				4.70
K.GVTFNVTTVDTK.R	Y	48.38	1280.6613	12	1.0	641.3386	2	41.97	4	F4:11922	17112021_RID_2349_DP4.raw				6.49

total 2 peptides

TRINITY_DN178
back to list

| Protein Coverage | Supporting Peptides |

Protein Coverage:

1 SSTGGSRVEA SRPEPRGGRL FPRGPSQQGP LAGWLGSVPG RGRSSGGKLR RSCESRLLGA APGSPPALPR RPLGPREPPL
81 PSQSVQASSS SSSSSAMAEE KPQVELFVKA GSDGAKIGNC PFSQRLFMVL WLK**GVTFNVT TVDTK**RRTEA VQKLCPGGQL
161 PFLMYGTEVH TDTNKIEEFL EEVLCPPRYP K**LAANNPESN TAGLDVFAK**F SAYIKNSNPS LNANLEKGLL KALKVLDVYL
241 MTPQPEEVDE NSAEDEGQSN RKFLDGNELT LADCNLLPKL HIVKVVCKKY RNFTIPEEFC GIHRYLKNAY AREEFASTCP
321 DDEEIELAYE LVAKALK

Supporting Peptides:

Peptide	Uniq	-10lgP	Mass	Length	ppm	m/z	z	RT	Fraction	Scan	Source File	Area F1	Area F2	Area F3	Are
K.LAANNPESNTAGLDVFAK.F	Y	72.78	1830.9111	18	1.0	916.4637	2	47.50	4	F4:13719	17112021_RID_2349_DP4.raw				4.70
K.GVTFNVTTVDTK.R	Y	48.38	1280.6613	12	1.0	641.3386	2	41.97	4	F4:11922	17112021_RID_2349_DP4.raw				6.49

total 2 peptides

LAB63521.1

back to list

| Protein Coverage | Supporting Peptides |

Protein Coverage:

1 MAEEKPQVEL FVKAGSDGAK IGNCPFSQRL FMVLWLK**GVT FNVTTVDTK**R RTEAVQKLCP GGQLPFLMYG TEVHTDTNKV
81 EEFLEEVLCP PKYPK**LAANN PESNTAGLDV FAK**FSAYIKN SNPSLNANLE KGLLKALKVL DLYLMTPQPE EVDENSAEDE
161 GQSNRKFLDG NELTLADCNL LPKLHIVKVV CKKYRDFTIP EEFRGIHRYL KNAYAREEFS STCPDDEEIE LAYELVAKAL
241 K

Supporting Peptides:

Peptide	Uniq	-10lgP	Mass	Length	ppm	m/z	z	RT	Fraction	Scan	Source File	Area F1	Area F2	Area F3	Are
K.LAANNPESNTAGLDVFAK.F	Y	72.78	1830.9111	18	1.0	916.4637	2	47.50	4	F4:13719	17112021_RID_2349_DP4.raw				4.70
K.GVTFNVTTVDTK.R	Y	48.38	1280.6613	12	1.0	641.3386	2	41.97	4	F4:11922	17112021_RID_2349_DP4.raw				6.49

total 2 peptides

JAG68347.1

back to list

| Protein Coverage | Supporting Peptides |

Protein Coverage:

1 MAEEKPQVEL FVKAGSDGAK IGNCPFSQRL FMVLWLK**GVT FNVTTVDTK**R RTEAVQKLCP GGQLPFLMYG TEVHTDTNKV
81 EEFLEEVLCP PKYPK**LAANN PESNTAGLDV FAK**FSAYIKN SNPSLNANLE KGLLKALKVL BVYLMTPQPE EVDENSAEDE
161 GQSNRKFLDG NELTLADCNL LPKLHIVKVV CKKYRDFTIP EEFRGIHRYL KNAYAREEFS STCPDDEEIE LAYELVAKAL
241 K

Supporting Peptides:

Peptide	Uniq	-10lgP	Mass	Length	ppm	m/z	z	RT	Fraction	Scan	Source File	Area F1	Area F2	Area F3	Are
K.LAANNPESNTAGLDVFAK.F	Y	72.78	1830.9111	18	1.0	916.4637	2	47.50	4	F4:13719	17112021_RID_2349_DP4.raw				4.70
K.GVTFNVTTVDTK.R	Y	48.38	1280.6613	12	1.0	641.3386	2	41.97	4	F4:11922	17112021_RID_2349_DP4.raw				6.49

total 2 peptides

ETE58466.1

back to list

| Protein Coverage | Supporting Peptides |

Protein Coverage:

1 MVELVEILGI LEEARRGEVK VKRKGVRKAL CERGKGGGER EGRKKEGKRK RKKEGKERRK EGEKERREKR EKRERKKKKK
81 ERPFPIAKKE RKKERQAIWL FLAGSDGAKI GNCPFSQRLF MVLWLK**GVTF NVTTVDTK**RY PK**LAANNPES NTAGLDVFAK**
161 FSAYIKNSNP SLNANLEKGL LKALKVLDIY LMTPQPEEVD ENSAEDEGQS NRKFLDGNEL TLADCNLLPK LHIVKVVCKK
241 YRDFTIPEEF RGIHRYLKNA YAREEFSSTC PDDEEIELAY ELVAKALK

Supporting Peptides:

Peptide	Uniq	-10lgP	Mass	Length	ppm	m/z	z	RT	Fraction	Scan	Source File	Area F1	Area F2	Area F3	Are
K.LAANNPESNTAGLDVFAK.F	Y	72.78	1830.9111	18	1.0	916.4637	2	47.50	4	F4:13719	17112021_RID_2349_DP4.raw				4.70
K.GVTFNVTTVDTK.R	Y	48.38	1280.6613	12	1.0	641.3386	2	41.97	4	F4:11922	17112021_RID_2349_DP4.raw				6.49

total 2 peptides

TRINITY_DN7999

back to list

| Protein Coverage | Supporting Peptides |

Protein Coverage:

```
  1 MTPHFLILIM LFAFSKGGLS QIVLTQSDPV LKKPGEAHKL TCAVTGFNVN SYWMGWIRQK PGQGLEWLVR YYDTSNTIYY
 81 SPTIQGRFTP SKDSSNFYLQ MNNLKAEDTA VYYCARDRQV RDFYYWGKGT TVTVTSAGKT APTVFPLSPC NQDIATASLV
161 SIGCFIKGYF PEPATVQWNS GAITSGIQNF PPVLLASNHY SHTSLLTIPA SQWQSESFQC NVEHAATNTR INKIIERSAI
241 RSPVGPEITV FHSCDSRDSD GTVQLWCHIS GFYPKQLDID WKVGSRSGQL HPYNYPPWQN PGTYTFSTAS VVNITQDDWL
321 EGNSYYCEAT HAASQTKVKG KAKKCKDGSS CPSGDIKVDI LPPRPKALYI DRNSKISCVV SNLQNEQGLK FTWFREKSGH
401 LTPDAIETTE GSNGGYIVLS RLGIFSEDWD SGETFTCVVE HPTFVSPVTK KISKSRGKHS APKVYVFPPH QEELNPGRSV
481 SLTCLVTGFS PEAADVQWLE DHNPLSEDNS VTTPPMKNKH DDSYFLYSKL LVPKTDWNTK SFTCMVVHED LPMKFTQRNI
561 SRTQGKK
```

Supporting Peptides:

Peptide	Uniq	-10lgP	Mass	Length	ppm	m/z	z	RT	Fraction	Scan	Source File	Area F1	Area F2	Area F3	Ar F
K.DSSNFYLQMNNLK.A	Y	63.76	1572.7242	13	0.8	787.3700	2	52.45	7	F7:15573	17112021_RID_2349_DP7.raw				
K.DSSNFYLQM(+15.99)NNLK.A	Y	48.08	1588.7191	13	1.4	795.3679	2	43.09	7	F7:12421	17112021_RID_2349_DP7.raw				
S.NTIYYSPTIQGR.F	Y	42.97	1411.7096	12	1.2	706.8629	2	39.01	7	F7:11021	17112021_RID_2349_DP7.raw				
S.QIVLTQSDPVLK.K	Y	42.47	1339.7711	12	0.7	670.8933	2	59.10	7	F7:17836	17112021_RID_2349_DP7.raw				

total 4 peptide

JAV50725.1

back to list

| Protein Coverage | Supporting Peptides |

Protein Coverage:

```
  1 MVSPRTCINK LGSTLPLLLV LWDLHYEGVQ CGINAEVEKQ LELGKKLLAA GQLADALSHF HAAIEGDADN YLAYYRRATV
 81 YLAMGKSKAA IRDLSRVVEL KQDFTSARLQ RGHLLLKQGK LNEAVEDFEG VLLSNPNDKE EGEARAQLLK SDEARNLRSQ
161 AQSAFLESDY YTAITLLDKI LEVCVWDVDM RELRAESYIK EGEPGKAISD LKAAAKLKSD NTEAFYKIST IYYELGDHEM
241 SLSEVRECLK LDQDHKECFS HYKRIKKLNK QIVSAEDLIQ ERRYQEATEK YEAVMKMETN VPVYTTRAKE RICHCLSKNQ
321 QSTEAIKVCT EVLQLEPENV NALKDRAEAY LLEEMYEEAI QDYETAKKHS DNDQQILESL EKAQRMLKQS QKRDYYKILG
401 VKRNARKQEI IKAYRKLAMQ WHPDNFQDEE EKKKAEKKFI DIAAAKEVLT DPEMRKKFDA GEDPLDAESQ QGGGHPFHRS
481 WNSWQGFDPF SSGGPFQFKF HFN
```

Supporting Peptides:

Peptide	Uniq	-10lgP	Mass	Length	ppm	m/z	z	RT	Fraction	Scan	Source File	Area F1	Area F2	Area F3	Area F4	Area F5
K.QIVSAEDLIQER.R	Y	58.67	1399.7307	12	0.2	700.8728	2	46.91	3	F3:13615	17112021_RID_2349_DP3.raw			9.5286E4		
K.LKSDNTEAFYK.I	N	47.39	1314.6455	11	1.2	658.3308	2	27.25	7	F7:6950	17112021_RID_2349_DP7.raw					
K.EVLTDPEMR.K	N	42.20	1088.5172	9	0.9	545.2664	2	34.55	7	F7:9479	17112021_RID_2349_DP7.raw					

total 3 peptides

ADP88560.1

back to list

| Protein Coverage | Supporting Peptides |

Protein Coverage:

```
  1 MVLIKVLANL LVLQLSYAQK SSELVVGGDE CNINEHRSLV FLYNNSFGCS GTLINQQWVL SAVHCDMENV RIYLGVHNLT
 81 LRNNAEIRLP EERFFCLSNK NYTKWDKDIM LIKLDRPVKT STYIAPLSLP SSPPRVGSVC RIMGWGAITS PNETFPGVTH
161 CANINILPYS VCRAAYKGLP AQSRTLCGGI LEGGIGSCMG DSGGPLICNG EMHGIVAWGD DTCAQPHKPV HYTKVYDYTD
241 WIQSIIAGNT AATCPP
```

Supporting Peptides:

Peptide	Uniq	-10lgP	Mass	Length	ppm	m/z	z	RT	Fraction	Scan	Source File	Area F1	Area F2	Area F
K.TSTYIAPLSLPSSPPR.V	Y	65.05	1685.8988	16	0.6	843.9572	2	51.64	7	F7:15270	17112021_RID_2349_DP7.raw	3.9743E5	3.772E5	
K.WDKDIMLIK.L	N	57.12	1160.6263	9	-0.1	581.3204	2	46.64	2	F2:13338	12112021_RID_2349_DP2.raw	2.1169E5	9.915E6	1.5104I
K.WDKDIM(+15.99)LIK.L	N	52.20	1176.6212	9	-0.2	589.3177	2	41.27	2	F2:11445	12112021_RID_2349_DP2.raw		6.6323E6	2.1157I

total

E5L0E4.1

back to list

| Protein Coverage | Supporting Peptides |

Protein Coverage:

1 MVLIKVLANL LVLQLSYAQK SSELVVGGDE CNINEHRSLV FLYNNSFGCS GTLINQQWVL SAVHCDMENV RIYLGVHNLT

81 LRNNAEIRLP EERFFCLSNK NYTK**WDKDIM LIK**LDRPVK**T STYIAPLSLP SSPPR**VGSVC RIMGWGAITS PNETFPGVTH

161 CANINILPYS VCRAAYKGLP AQSRTLCGGI LEGGIGSCMG DSGGPLICNG EMHGIVAWGD DTCAQPHKPV HYTKVYDYTD

241 WIQSIIAGNT AATCPP

Supporting Peptides:

Peptide	Uniq	-10lgP	Mass	Length	ppm	m/z	z	RT	Fraction	Scan	Source File	Area F1	Area F2	Area F
K.TSTYIAPLSLPSSPPR.V	Y	65.05	1685.8988	16	0.6	843.9572	2	51.64	7	F7:15270	17112021_RID_2349_DP7.raw	3.9743E5	3.772E5	
K.WDKDIMLIK.L	N	57.12	1160.6263	9	-0.1	581.3204	2	46.64	2	F2:13338	12112021_RID_2349_DP2.raw	2.1169E5	9.915E6	1.5104I
K.WDKDIM(+15.99)LIK.L	N	52.20	1176.6212	9	-0.2	589.3177	2	41.27	2	F2:11445	12112021_RID_2349_DP2.raw		6.6323E6	2.1157I

total

XP_026535564.1

back to list

| Protein Coverage | Supporting Peptides |

Protein Coverage:

1 MSLASGDSSS SSPGEEPRGT EPVKSLISHK ASHNSYVELH ELVMDKNNEF RWMEASRWIK LEEDFEEMGQ WGRPHLSYLT

81 FQSLLEVRKA FSK**GTVLLDL QER**SLAGIAN QVLSQMIYEG KLKPDDQEVL LRTLLLQHSQ PSESTSLNTL LPARVQRTDA

161 KDTTEPLLQE QHQIEMKTFF NGSEQGESSH SGFRKQLLEK IPPDAEATLG LVGCATFLDR PILAFVRLKE AVELDSVLEV

241 PVPVRFLFVV LGPDSPHISY HEIGR**AISTM MSER**VFR**NDS YLAEGR**QDLI KSLDEFLDCC IVLPPSEIQS ERLLKMLLPV

321 QKDFLRKRYL PVEKKLEEPL KDLGHKLKDI TAEDSGDSLR RTGNIFGGLV KDIKRRYPKY LSDIKDALNP QCLAAVIFIY

401 FAALSPAITF GGLLAEKTDR LIGVSELLLS TAVQSGLFCL LGAQPLLVVG FSGPLLVFEE AFYAFCTANG IEYIVGRVWI

481 GFWLILIVIL VVAFEGSFLV RFISRYTQEI FSILISLIFI LETFFKLIKI FKEHPLKEAY KNVTFSRDED PREPVPNTAL

561 LSLVLMGGTF FIAFFLRKFK NSSFFPGRLR RVIGDFGVPI AILIMTLADN LIKDTYTQKL KVPKGLQVSN PEKRDWFIHP

641 LGNEFPKWMM FASLVPALLV FILIFLETQI TTLIVSKPER KMVKGSGFHL DLLLIVGMGG IAPLLGMPWL SATTVRTVTH

721 ANALTVMSKT TLPGEKACIQ EVKEQRVTGF LVAILVGLSI LMEPILKLIP LAVLFGIFLY MGVTSLNGIQ LYDRILLMLK

801 PPKYHPDVPY VTKVKTWHMH IFTLTQILCL GLLWGVKTSP ASLALPFILI LTVPLRYFLL PVIFTDIELK CLDAEDTELT

881 FDETEGQDVY DEVQMPV

Supporting Peptides:

Peptide	Uniq	-10lgP	Mass	Length	ppm	m/z	z	RT	Fraction	Scan	Source File	Area F1	Area F2	Area F3	Area F4	Area F5	Area F6
R.AISTMMSER.V	Y	54.88	1024.4681	9	0.2	513.2415	2	31.14	7	F7:8285	17112021_RID_2349_DP7.raw						
K.GTVLLDLQER.S	Y	50.80	1142.6295	10	0.8	572.3225	2	45.59	7	F7:13262	17112021_RID_2349_DP7.raw						
R.NDSYLAEGR.Q	Y	47.45	1023.4621	9	0.6	512.7386	2	26.44	7	F7:6652	17112021_RID_2349_DP7.raw						

total 3 peptides

XP_026535563.1

back to list

| Protein Coverage | Supporting Peptides |

Protein Coverage:

1 MAFQEDYESG IKEKLDLEAY GDSSLKQSNM SLASGDSSSS SPGEEPRGTE PVKSLISHKA SHNSYVELHE LVMDKNNEFR

81 WMEASRWIKL EEDFEEMGQW GRPHLSYLTF QSLLEVRKAF SK**GTVLLDLQ ER**SLAGIANQ VLSQMIYEGK LKPDDQEVLL

161 RTLLLQHSQP SESTSLNTLL PARVQRTDAK DTTEPLLQEQ HQIEMKTFFN GSEQGESSHS GFRKQLLEKI PPDAEATLGL

241 VGCATFLDRP ILAFVRLKEA VELDSVLEVP VPVRFLFVVL GPDSPHISYH EIGR**AISTMM SER**VFR**NDSY LAEGR**QDLIK

321 SLDEFLDCCI VLPPSEIQSE RLLKMLLPVQ KDFLRKRYLP VEKKLEEPLK DLGHKLKDIT AEDSGDSLRR TGNIFGGLVK

401 DIKRRYPKYL SDIKDALNPQ CLAAVIFIYF AALSPAITFG GLLAEKTDRL IGVSELLLST AVQSGLFCLL GAQPLLVVGF

481 SGPLLVFEEA FYAFCTANGI EYIVGRVWIG FWLILIVILV VAFEGSFLVR FISRYTQEIF SILISLIFIL ETFFKLIKIF

561 KEHPLKEAYK NVTFSRDEDP REPVPNTALL SLVLMGGTFF IAFFLRKFKN SSFFPGRLRR VIGDFGVPIA ILIMTLADNL

641 IKDTYTQKLK VPKGLQVSNP EKRDWFIHPL GNEFPKWMMF ASLVPALLVF ILIFLETQIT TLIVSKPERK MVKGSGFHLD

721 LLLIVGMGGI APLLGMPWLS ATTVRTVTHA NALTVMSKTT LPGEKACIQE VKEQRVTGFL VAILVGLSIL MEPILKLIPL

801 AVLFGIFLYM GVTSLNGIQL YDRILLMLKP PKYHPDVPYV TKVKTWHMHI FTLTQILCLG LLWGVKTSPA SLALPFILIL

881 TVPLRYFLLP VIFTDIELKC LDAEDTELTF DETEGQDVYD EVQMPV

Supporting Peptides:

Peptide	Uniq	-10lgP	Mass	Length	ppm	m/z	z	RT	Fraction	Scan	Source File	Area F1	Area F2	Area F3	Area F4	Area F5	Area F6

Peptide	Uniq	-10lgP	Mass	Length	ppm	m/z	z	RT	Fraction	Scan	Source File	Area F1	Area F2	Area F3	Area F4	Area F5	Area F6
R.AISTMMSER.V	Y	54.88	1024.4681	9	0.2	513.2415	2	31.14	7	F7:8285	17112021_RID_2349_DP7.raw						
K.GTVLLDLQER.S	Y	50.80	1142.6295	10	0.8	572.3225	2	45.59	7	F7:13262	17112021_RID_2349_DP7.raw						
R.NDSYLAEGR.Q	Y	47.45	1023.4621	9	0.6	512.7386	2	26.44	7	F7:6652	17112021_RID_2349_DP7.raw						

total 3 peptides

XP_013918193.1
back to list

| Protein Coverage | Supporting Peptides |
Protein Coverage:

```
  1 MERVSMGKAG SYQRHMDTSS SSSSEEEPRG TEPAKPLISH KASHNSYVEL HELVMDKNNE FRWMEASHWI KLEEDFEEMG
 81 HWGRPHLSYL TFQSLLEVRK AFSKGTVLLD LQERSLAGIA NQVLSQMIYE GKLKPDDQEV LLRTLLLQHS HPSESASLNT
161 LLPALVQRTD TKDSTQPLLQ EHQQIEMKTF SNGSEQGESS HSDFKKQLLD KIPPDAEATL GLVGCATFLD RPTVAFVRLK
241 EAVELDSVLE VPVPVRFLFV VLGPDSPHIS YHEIGRAIST MMSERVFRND SYLAEGRQDL IKSLDEFLDC CIVLPPSEIQ
321 NERLLKMLLP VQKDFLRKRY LPVEKKLEEP LKDLGHKLKD HDITAEDSDD SLRRTGKIFG GMVKDIKRRY PKYLSDIKDA
401 LNPQCLAAVI FIYFAALSPA ITFGGLLAEK TERLIGVSEL LLSTAVQSGL FCLLGAQPLL VVGFSGPLLV FEEAFYAFCV
481 NKNYEYLVGR VWIGFWLILI VVVVVAFEGS FLVRFISRYT QEIFSILISL IFILETFFKL IKIFKEHPLK EVYKNITYPP
561 DEEPRYPVPN TALLSLVLMG GTFFIAFFLR KFKNSSFFPG KLRRVIGDFG VPIAIFIMTL ADYLIKDTYT QKLKVPKGLQ
641 VSNPEQRNWF IHPLGSSLGN PFPIWMMFAT ALPALLVFIL IFLETQITTL IVSKPERKML KGSGFHLDLL LIVGMGGIAP
721 LLGMPWLSAT TVRTVTHANA LTVMSKTTLP GEKACIQEVK EQRVTGFLVA ILVGLSILME PILKLIPLAV LFGIFLYMGV
801 TSLNGIQLYD RILLLLKPPK YHPDVSYVTK VKTWRMHIFT FTQMLCLGLL WGVKSSPASL ALPFILILTV PLRLFLLPFI
881 FTDVELKCLD ADDAEVTFDE TEGQDVYDEV QMPV
```

Supporting Peptides:

Peptide	Uniq	-10lgP	Mass	Length	ppm	m/z	z	RT	Fraction	Scan	Source File	Area F1	Area F2	Area F3	Area F4	Area F5	Area F6
R.AISTMMSER.V	Y	54.88	1024.4681	9	0.2	513.2415	2	31.14	7	F7:8285	17112021_RID_2349_DP7.raw						
K.GTVLLDLQER.S	Y	50.80	1142.6295	10	0.8	572.3225	2	45.59	7	F7:13262	17112021_RID_2349_DP7.raw						
R.NDSYLAEGR.Q	Y	47.45	1023.4621	9	0.6	512.7386	2	26.44	7	F7:6652	17112021_RID_2349_DP7.raw						

total 3 peptides

XP_034271810.1
back to list

| Protein Coverage | Supporting Peptides |
Protein Coverage:

```
  1 MSICQHICIS FSQYSCPGHQ GIYHLSKRRR MERVSMGKAG SYQRHVDTSS SSSSEEEPRG TEPAKPLISH KASHNSYVEL
 81 HELVMDKNNE FRWMEASHWI KLEEDFEEMG HWGRPHLSYL TFQSLLEVRK AFSKGTVLLD LQERSLAGIA NQVLSQMIYE
161 GKLKPDDQEV LLRTLLLQHS HPSESASLNT LLPARVQRTD AKDSTQPLLQ EHHQIEMKTF SNGSEQGESS HSGFKKQLLD
241 KIPPDAEATL GLVGCATFLD RPTLAFVRLK EAVELDSVLE VPVPVRFLFV VLGPDSPHIN YHEIGRAIST MMSERVFRND
321 SYLAEGRQDL IKSLDEFLDC CIVLPPSEIQ NERLLKMLLP VQKDFLRKRY LPVEKKLEEP LKDLAHKLKD HDITAEDSDD
401 PLRRTGRIFG GLVKDIKRRY PKYLSDIKDA LNPQCLAAVI FIYFAALSPA ITFGGLLAEE TERLIGVSEL LLSTAVQSGL
481 FCLLGAQPLL VVGFSGPLLV FEKAFYAFCT NYNIEYLVGR VWIGFWLILI VILVVAFEGS FLVRYISRYT QEIFSILISL
561 IFIQETFSNL IKIFKAHPLK GVYKNITFSG EEEPREPVPN TALLSLVLMG GTFFIAFFLR KFKNSSFFPG RLRRVIGDFG
641 VPIAIFIMTL ADNLIKDTYT QKLKVPKGLQ VSNTDKRDWF IHPLKEPIPK WFLFAAALPA LLVFILIFLE TQITTLIVSK
721 PERKLLKGSG FHLDLLLIVG MGGIAPLLGM PWLSATTVRT VTHANALTIM SKTTLPGEKA CIQEVKEQRI TGFLVALLVG
801 LSILMEPILK LIPLAVLFGI FLYMGVTSLS GIQLYDRILL MLKPPKYHPD VSYVTKVKSW RMHIFTFSQI LCLGLLWGVK
881 SSPASLALPF ILILTVPLRR FLLPVIFTDI ELKCLDADDA EVTFDETEGQ DVYDEVQMPV
```

Supporting Peptides:

Peptide	Uniq	-10lgP	Mass	Length	ppm	m/z	z	RT	Fraction	Scan	Source File	Area F1	Area F2	Area F3	Area F4	Area F5	Area F6
R.AISTMMSER.V	Y	54.88	1024.4681	9	0.2	513.2415	2	31.14	7	F7:8285	17112021_RID_2349_DP7.raw						
K.GTVLLDLQER.S	Y	50.80	1142.6295	10	0.8	572.3225	2	45.59	7	F7:13262	17112021_RID_2349_DP7.raw						
R.NDSYLAEGR.Q	Y	47.45	1023.4621	9	0.6	512.7386	2	26.44	7	F7:6652	17112021_RID_2349_DP7.raw						

total 3 peptides

XP_013918195.1
back to list

| Protein Coverage | Supporting Peptides |

Protein Coverage:

```
  1 MQKKSYVELH ELVMDKNNEF RWMEASHWIK LEEDFEEMGH WGRPHLSYLT FQSLLEVRKA FSKGTVLLDL QERSLAGIAN
 81 QVLSQMIYEG KLKPDDQEVL LRTLLLQHSH PSESASLNTL LPALVQRTDT KDSTQPLLQE HQQIEMKTFS NGSEQGESSH
161 SDFKKQLLDK IPPDAEATLG LVGCATFLDR PTVAFVRLKE AVELDSVLEV PVPVRFLFVV LGPDSPHISY HEIGRAISTM
241 MSERVFRNDS YLAEGRQDLI KSLDEFLDCC IVLPPSEIQN ERLLKMLLPV QKDFLRKRYL PVEKKLEEPL KDLGHKLKDH
321 DITAEDSDDS LRRTGKIFGG MVKDIKRRYP KYLSDIKDAL NPQCLAAVIF IYFAALSPAI TFGGLLAEKT ERLIGVSELL
401 LSTAVQSGLF CLLGAQPLLV VGFSGPLLVF EEAFYAFCVN KNYEYLVGRV WIGFWLILIV VVVVAFEGSF LVRFISRYTQ
481 EIFSILISLI FILETFFKLI KIFKEHPLKE VYKNITYPPD EEPRYPVPNT ALLSLVLMGG TFFIAFFLRK FKNSSFFPGK
561 LRRVIGDFGV PIAIFIMTLA DYLIKDTYTQ KLKVPKGLQV SNPEQRNWFI HPLGSSLGNP FPIWMMFATA LPALLVFILI
641 FLETQITTLI VSKPERKMLK GSGFHLDLLL IVGMGGIAPL LGMPWLSATT VRTVTHANAL TVMSKTTLPG EKACIQEVKE
721 QRVTGFLVAI LVGLSILMEP ILKLIPLAVL FGIFLYMGVT SLNGIQLYDR ILLLLKPPKY HPDVSYVTKV KTWRMHIFTF
801 TQMLCLGLLW GVKSSPASLA LPFILILTVP LRLFLLPFIF TDVELKCLDA DDAEVTFDET EGQDVYDEVQ MPV
```

Supporting Peptides:

Peptide	Uniq	-10lgP	Mass	Length	ppm	m/z	z	RT	Fraction	Scan	Source File	Area F1	Area F2	Area F3	Area F4	Area F5	Area F6
R.AISTMMSER.V	Y	54.88	1024.4681	9	0.2	513.2415	2	31.14	7	F7:8285	17112021_RID_2349_DP7.raw						
K.GTVLLDLQER.S	Y	50.80	1142.6295	10	0.8	572.3225	2	45.59	7	F7:13262	17112021_RID_2349_DP7.raw						
R.NDSYLAEGR.Q	Y	47.45	1023.4621	9	0.6	512.7386	2	26.44	7	F7:6652	17112021_RID_2349_DP7.raw						

total 3 peptides

XP_013918194.1

back to list

| Protein Coverage | Supporting Peptides |

Protein Coverage:

```
  1 MASQEDYESG IKAKLDVEAY GDSSLKQSNM SLASGNSSSS SSEEEPRGTE PAKPLISHKA SHNSYVELHE LVMDKNNEFR
 81 WMEASHWIKL EEDFEEMGHW GRPHLSYLTF QSLLEVRKAF SKGTVLLDLQ ERSLAGIANQ VLSQMIYEGK LKPDDQEVLL
161 RTLLLQHSHP SESASLNTLL PALVQRTDTK DSTQPLLQEH QQIEMKTFSN GSEQGESSHS DFKKQLLDKI PPDAEATLGL
241 VGCATFLDRP TVAFVRLKEA VELDSVLEVP VPVRFLFVVL GPDSPHISYH EIGRAISTMM SERVFRNDSY LAEGRQDLIK
321 SLDEFLDCCI VLPPSEIQNE RLLKMLLPVQ KDFLRKRYLP VEKKLEEPLK DLGHKLKDHD ITAEDSDDSL RRTGKIFGGM
401 VKDIKRRYPK YLSDIKDALN PQCLAAVIFI YFAALSPAIT FGGLLAEKTE RLIGVSELLL STAVQSGLFC LLGAQPLLVV
481 GFSGPLLVFE EAFYAFCVNK NYEYLVGRVW IGFWLILIVV VVVAFEGSFL VRFISRYTQE IFSILISLIF ILETFFKLIK
561 IFKEHPLKEV YKNITYPPDE EPRYPVPNTA LLSLVLMGGT FFIAFFLRKF KNSSFFPGKL RRVIGDFGVP IAIFIMTLAD
641 YLIKDTYTQK LKVPKGLQVS NPEQRNWFIH PLGSSLGNPF PIWMMFATAL PALLVFILIF LETQITTLIV SKPERKMLKG
721 SGFHLDLLLI VGMGGIAPLL GMPWLSATTV RTVTHANALT VMSKTTLPGE KACIQEVKEQ RVTGFLVAIL VGLSILMEPI
801 LKLIPLAVLF GIFLYMGVTS LNGIQLYDRI LLLLKPPKYH PDVSYVTKVK TWRMHIFTFT QMLCLGLLWG VKSSPASLAL
881 PFILILTVPL RLFLLPFIFT DVELKCLDAD DAEVTFDETE GQDVYDEVQM PV
```

Supporting Peptides:

Peptide	Uniq	-10lgP	Mass	Length	ppm	m/z	z	RT	Fraction	Scan	Source File	Area F1	Area F2	Area F3	Area F4	Area F5	Area F6
R.AISTMMSER.V	Y	54.88	1024.4681	9	0.2	513.2415	2	31.14	7	F7:8285	17112021_RID_2349_DP7.raw						
K.GTVLLDLQER.S	Y	50.80	1142.6295	10	0.8	572.3225	2	45.59	7	F7:13262	17112021_RID_2349_DP7.raw						
R.NDSYLAEGR.Q	Y	47.45	1023.4621	9	0.6	512.7386	2	26.44	7	F7:6652	17112021_RID_2349_DP7.raw						

total 3 peptides

XP_034271811.1

back to list

| Protein Coverage | Supporting Peptides |

Protein Coverage:

1 MASQEDYESG IKAKLDLEAY GDSSLKQSNM SLASGDSSSS SSEEEPRGTE PAKPLISHKA SHNSYVELHE LVMDKNNEFR
81 WMEASHWIKL EEDFEEMGHW GRPHLSYLTF QSLLEVRKAF SK**GTVLLDLQ ER**SLAGIANQ VLSQMIYEGK LKPDDQEVLL
161 RTLLLQHSHP SESASLNTLL PARVQRTDAK DSTQPLLQEH HQIEMKTFSN GSEQGESSHS GFKKQLLDKI PPDAEATLGL
241 VGCATFLDRP TLAFVRLKEA VELDSVLEVP VPVRFLFVVL GPDSPHINYH EIGR**AISTMM SER**VFR**NDSY LAEGR**QDLIK
321 SLDEFLDCCI VLPPSEIQNE RLLKMLLPVQ KDFLRKRYLP VEKKLEEPLK DLAHKLKDHD ITAEDSDDPL RRTGRIFGGL
401 VKDIKRRYPK YLSDIKDALN PQCLAAVIFI YFAALSPAIT FGGLLAEETE RLIGVSELLL STAVQSGLFC LLGAQPLLVV
481 GFSGPLLVFE KAFYAFCTNY NIEYLVGRVW IGFWLILIVI LVVAFEGSFL VRYISRYTQE IFSILISLIF IQETFSNLIK
561 IFKAHPLKGV YKNITFSGEE EPREPVPNTA LLSLVLMGGT FFIAFFLRKF KNSSFFPGRL RRVIGDFGVP IAIFIMTLAD
641 NLIKDTYTQK LKVPKGLQVS NTDKRDWFIH PLKEPIPKWF LFAAALPALL VFILIFLETQ ITTLIVSKPE RKLLKGSGFH
721 LDLLLIVGMG GIAPLLGMPW LSATTVRTVT HANALTIMSK TTLPGEKACI QEVKEQRITG FLVALLVGLS ILMEPILKLI
801 PLAVLFGIFL YMGVTSLSGI QLYDRILLML KPPKYHPDVS YVTKVKSWRM HIFTFSQILC LGLLWGVKSS PASLALPFIL
881 ILTVPLRRFL LPVIFTDIEL KCLDADDAEV TFDETEGQDV YDEVQMPV

Supporting Peptides:

Peptide	Uniq	-10lgP	Mass	Length	ppm	m/z	z	RT	Fraction	Scan	Source File	Area F1	Area F2	Area F3	Area F4	Area F5	Area F6
R.AISTMMSER.V	Y	54.88	1024.4681	9	0.2	513.2415	2	31.14	7	F7:8285	17112021_RID_2349_DP7.raw						
K.GTVLLDLQER.S	Y	50.80	1142.6295	10	0.8	572.3225	2	45.59	7	F7:13262	17112021_RID_2349_DP7.raw						
R.NDSYLAEGR.Q	Y	47.45	1023.4621	9	0.6	512.7386	2	26.44	7	F7:6652	17112021_RID_2349_DP7.raw						

total 3 peptides

XP_029143180.1
back to list

| Protein Coverage | Supporting Peptides |
Protein Coverage:

1 MSLASGDSSS SSPEEEPRGT EPVKPLISHK ASHNSYVELH ELVMDKNNEF RWMEASHWIK LEEDFEEMGH WGRPHLSYLT
81 FQSLLEVRKA FNK**GTVLLDL QER**SLAGIAN QVLSQMTNEG KLKPDDQEVL LRTLLLQHSH PNESTSLNTL LPARVQQTDA
161 KDSTQPLLQE HHQIEMKTFF NGSEQGESSH SDFKKQLLDK IPSDAEATLG LVGCATFLDR PTLAFVRLKE AVELDSVLEV
241 PVPVRFLFVV LGPDSPHISY HEIGR**AISTM MSER**VFR**NDS YLAEGR**QDLI KSLDEFLDCC IVLPPSEIQN ERLLKMLLPV
321 QKDFLRKRYL PVEKKLEESL KDLGHKLKDH EVKAEDSDDS LRRTGRIFGG VVKDIKRRYP KYLSDIKDAL NPQCLAAVIF
401 IYFAALSPAI TFGGLLAEKT ERLIGVSELL LSTAVQSGLF CLLGAQPLLV VGFSGPLLVF EEAFYTFCTK NNIEYIVGRV
481 WIGFWLILIV IVVVAFEGSF LVRYISRYTQ EIFSILISLI FILETFFKLI KIFKEHPLKE VYTNITFSRD EEPRGPAPNT
561 ALLSLVLMGG TFFIAFFLRK FKNSSFFPGR LRRVIGDFGV PIAIFIMTLA DNLIKDTYTQ KLKVPKGLQV SNPEKRGWFI
641 YPTGIAFPIW MMFAAALPAL LVFILIFLET QITTLIVSKP ERKMLKGSGF HLDLLLIVGM GGIAPLLGMP WLSATTVRTV
721 THANALTVMS KTTLPGEKAC IQEVKEQRVT GFLVAILVGL SILMEPILKL IPLAVLFGIF LYMGVTSLNG IQLYDRILLM
801 LKPPKYHPDI SYVTKVKTWR MHIFTLTQIL CLGLLWGVKS SPASLALPFI LILTVPLRRF LLPVIFRDIE LKCLDSDDAE
881 VTFDETEGQD VYDEVQMPI

Supporting Peptides:

Peptide	Uniq	-10lgP	Mass	Length	ppm	m/z	z	RT	Fraction	Scan	Source File	Area F1	Area F2	Area F3	Area F4	Area F5	Area F6
R.AISTMMSER.V	Y	54.88	1024.4681	9	0.2	513.2415	2	31.14	7	F7:8285	17112021_RID_2349_DP7.raw						
K.GTVLLDLQER.S	Y	50.80	1142.6295	10	0.8	572.3225	2	45.59	7	F7:13262	17112021_RID_2349_DP7.raw						
R.NDSYLAEGR.Q	Y	47.45	1023.4621	9	0.6	512.7386	2	26.44	7	F7:6652	17112021_RID_2349_DP7.raw						

total 3 peptides

JAC95757.1
back to list

| Protein Coverage | Supporting Peptides |
Protein Coverage:

1 MVSPRTCVHK LGSTLPLLLV LWDLHYEGVQ CGINAEVEKQ LELGKKLLAA GQLADALSHF HAAIEGDXDN YLAYYRRATV
81 YLAMGKSKAA IRDLSRVVEL KQDFTSARLQ RGHLLLKQGK LDEAVEDFEG VLLSNPSDKE EGEARAQLLK SDEARRLC**SQ**
161 **AQSAFLEK**DY YTAITLLDKI LEVCVWDADM RELRAESYIK EGEPGKAISD LKAAAK**LKSD NTEAFYK**IST IYYELGDHEM
241 SLSEVRECLK LDQDHKECFS HYKRVKKLNK HIVSAEELIQ EGRYQEATEK YEAVMKMETN VPVYTTRAKE RICHCFSKNQ
321 QTTEAIKVCT EVLQLEPENV NALKDRAEAY LLEEMYEEAI QDYETAKK**YS DNDQQIQESL EK**AQRMLKQS QKRDYYKILG
401 VKRNARKQEI IKAYRKLAMQ WHPDNFQDEE EKKKAEKKFI DIAAAK**EVLT DPEMR**RKFDA GEDPLDAESQ QGGGHPFHRT
481 WNSWQGFDPF SSGGPFQFKF HFN

Supporting Peptides:

Peptide	Uniq	-10lgP	Mass	Length	ppm	m/z	z	RT	Fraction	Scan	Source File	Area F1	Area F2	Area F3	Area F
K.YSDNDQQIQESLEK.A	Y	49.17	1695.7588	14	0.5	848.8871	2	35.45	3	F3:9753	17112021_RID_2349_DP3.raw			1.1168E5	
K.LKSDNTEAFYK.I	N	47.39	1314.6455	11	1.2	658.3308	2	27.25	7	F7:6950	17112021_RID_2349_DP7.raw				
K.EVLTDPEMR.R	N	42.20	1088.5172	9	0.9	545.2664	2	34.55	7	F7:9479	17112021_RID_2349_DP7.raw				
C.SQAQSAFLEK.D	Y	41.16	1107.5560	10	0.3	554.7855	2	29.85	4	F4:7720	17112021_RID_2349_DP4.raw				8.8606[illegible]

total 4 peptides

ADI47590.1
back to list

| Protein Coverage | Supporting Peptides |
Protein Coverage:

```
  1 MMQVLLATIC LAVFPYQGSS IILESGNVND YEVVYPQKVT ALPQGAIQQP EQKYEDTMQY EIEVNGEPVV LHLEKNKDLF   C
 81 SEDYSETHYS PDGRKITTNP PVQDHCYYHG RIQNDAHSTA SMSACNGLKG YFKLRGETYF IEPLKIPDSE AHAVYKYENL
161 EKEDETPKIC GVTQTNWESD EPIKKASQLV ATSEQQRYYD RFRYVXXIVV VDHRMCKKYN GNLRRIRRRL YQLINFLNEM
241 YLPWNLRVPL VGMEFWNQRD LINVTSSPKV TLDLFGEWRE SDLLNRKEHD YAQLLTAIDF DGPTIGMAHI ATMCHSKLSV
321 GIVEDYRPAE SVVAAIMAHE MGHNLGLIHD ENQCNCDADS CIMYHQINNP PPMYFSNCSW NYYQNFLTNY KPDCTLIRPS
401 RTDIVSPPVC GNALLEKGEE CDCGSPANCQ DPCCDAASCK LHSWVECEIG ECCDQCRFKP AGTECRAIRS ECDLPEYCTG
481 QSVDCPIDRF HRNGKPCQSN NGYCYNGACP IMQNQCYALF GPDATVGQDA CFEENKKGKS YFYCRKENDV KIPCAPEDVK
561 CGRLLCEIVK NTCKYDYSED PNYGMVDEGT KCADGKVCRN RHCVDVTTAY
```

Supporting Peptides:

Peptide	Uniq	-10lgP	Mass	Length	ppm	m/z	z	RT	Fraction	Scan	Source File	Area F1	Area I
R.TDIVSPPVC(+57.02)GNALLEK.G	Y	60.69	1711.8815	16	0.8	856.9487	2	49.01	1	F1:14875	12112021_RID_2349_DP1.raw	1.4223E6	4.9549[illegible]
V.TQTNWESDEPIKK.A	N	44.98	1574.7576	13	0.5	788.3865	2	30.75	1	F1:8710	12112021_RID_2349_DP1.raw	3.3503E5	

E0Y421.1
back to list

| Protein Coverage | Supporting Peptides |
Protein Coverage:

```
  1 MVLIRVLANL LLLQLSYAQK SSELVVGGDE CNINEHRSLV FLYNSSFGCG GTLINQQWVL SAAHCDMENV QIYLGLHNLR   O C
 81 LRNQDEQIRV AEEKFFCLSN KSYTKWDKDI MLIRLNSSVT YNTHIAPLSL PSSPPRVGSV CRIMGWGAIT SPNETFPNVP
161 HCANINILRY SVCRAAYRGL PAQSRTLCAG ILQGGIGSCM GDSGGPLICN GEIQGIVSWG DDICAQPHKP VHYTKVFDYS
241 DWIQSIIAGN TTATCPL
```

Supporting Peptides:

Peptide	Uniq	-10lgP	Mass	Length	ppm	m/z	z	RT	Fraction	Scan	Source File	Area F1	Area F2	Area F
K.WDKDIMLIR.L	N	59.98	1188.6324	9	0.8	595.3240	2	47.61	2	F2:13483	12112021_RID_2349_DP2.raw		2.0105E7	1.3706[illegible]
R.AAYRGLPAQSR.T	Y	57.92	1188.6364	11	0.5	397.2196	3	24.28	1	F1:6637	12112021_RID_2349_DP1.raw	1.8692E7		
K.WDKDIM(+15.99)LIR.L	N	54.14	1204.6274	9	0.6	603.3214	2	42.22	2	F2:11819	12112021_RID_2349_DP2.raw		1.4264E7	3.0085[illegible]
W.DKDIMLIR.L	N	44.22	1002.5532	8	-0.5	502.2836	2	37.38	3	F3:10341	17112021_RID_2349_DP3.raw			5.8616[illegible]
W.DKDIM(+15.99)LIR.L	N	39.37	1018.5481	8	0.3	510.2815	2	31.54	3	F3:8358	17112021_RID_2349_DP3.raw			1.7097[illegible]

total 5 pept

ADN04919.1
back to list

| Protein Coverage | Supporting Peptides |
Protein Coverage:

```
  1 MVLIRVLANL LLLQLSYAQK SSELVVGGDE CNINEHRSLV FLYNSSFGCG GTLINQQWVL SAAHCDMENV QIYLGLHNLR   O C
 81 LRNQDEQIRV AEEKFFCLSN KSYTKWDKDI MLIRLNSSVT YNTHIAPLSL PSSPPRVGSV CRIMGWGAIT SPNETFPNVP
161 HCANINILRY SVCRAAYRGL PAQSRTLCAG ILQGGIGSCM GDSGGPLICN GEIQGIVSWG DDICAQPHKP VHYTKVFDYS
241 DWIQSIIAGN TTATCPL
```

Supporting Peptides:

Peptide	Uniq	-10lgP	Mass	Length	ppm	m/z	z	RT	Fraction	Scan	Source File	Area F1	Area F2	Area F
K.WDKDIMLIR.L	N	59.98	1188.6324	9	0.8	595.3240	2	47.61	2	F2:13483	12112021_RID_2349_DP2.raw		2.0105E7	1.3706I
R.AAYRGLPAQSR.T	Y	57.92	1188.6364	11	0.5	397.2196	3	24.28	1	F1:6637	12112021_RID_2349_DP1.raw	1.8692E7		
K.WDKDIM(+15.99)LIR.L	N	54.14	1204.6274	9	0.6	603.3214	2	42.22	2	F2:11819	12112021_RID_2349_DP2.raw		1.4264E7	3.0085I
W.DKDIMLIR.L	N	44.22	1002.5532	8	-0.5	502.2836	2	37.38	3	F3:10341	17112021_RID_2349_DP3.raw			5.8616I
W.DKDIM(+15.99)LIR.L	N	39.37	1018.5481	8	0.3	510.2815	2	31.54	3	F3:8358	17112021_RID_2349_DP3.raw			1.7097I

total 5 pept

QBF53413.1
back to list

| Protein Coverage | Supporting Peptides |
Protein Coverage:

```
  1 MVLIRVLANL LVLQLSYAQK SSELVVGGDE CNINEHRSLV FLYNSSFGCG GILINQEWVL SAAHCNMENV QIYLGMHNLR
 81 PRNKDEQIRV AEEKFFCLSS KNYTKWDKDI MLIRLNSSVT YNTHIAPLSL PSSPPRVGSV CRIMGWGAIT SPNETFPNVP
161 HCANINILHY SVCRAAYRGL PAQSRTLCAG ILQGGIGSCM GDSGGPLICN GEIQGIVSWG DDICAQPHKP VHYTKVFDYS
241 DWIQSIIAGN TTATCPP
```

Supporting Peptides:

Peptide	Uniq	-10lgP	Mass	Length	ppm	m/z	z	RT	Fraction	Scan	Source File	Area F1	Area F2	Area F
K.WDKDIMLIR.L	N	59.98	1188.6324	9	0.8	595.3240	2	47.61	2	F2:13483	12112021_RID_2349_DP2.raw		2.0105E7	1.3706I
R.AAYRGLPAQSR.T	Y	57.92	1188.6364	11	0.5	397.2196	3	24.28	1	F1:6637	12112021_RID_2349_DP1.raw	1.8692E7		
K.WDKDIM(+15.99)LIR.L	N	54.14	1204.6274	9	0.6	603.3214	2	42.22	2	F2:11819	12112021_RID_2349_DP2.raw		1.4264E7	3.0085I
W.DKDIMLIR.L	N	44.22	1002.5532	8	-0.5	502.2836	2	37.38	3	F3:10341	17112021_RID_2349_DP3.raw			5.8616I
W.DKDIM(+15.99)LIR.L	N	39.37	1018.5481	8	0.3	510.2815	2	31.54	3	F3:8358	17112021_RID_2349_DP3.raw			1.7097I

total 5 pept

XP_007435739.1
back to list

| Protein Coverage | Supporting Peptides |
Protein Coverage:

```
  1 MIKSQRNASS TVRSFSSRSY TPGPVSSSRI STSYVSSGFG GSRYGGGGSF RGASAGSLGG ITAVSVNKSL LAPLNLEIDP
 81 TIHQVKTKEK EEIKTLNNKF ANLIDKVRFL EQQNKMLETK WSLLQNQKTT RSNMDSMFEA YISNLRRQLE NLGQERARLD
161 AELANMQGLV EDFKNRYEDE INRRTEKENE FVLLKKDVDE AYMNKVELES RLECLTDEIN FLRQLYDEEL RVLQSQISDT
241 SVILSMDNNR SLDLDGIIAE VRAQYEEIAN RSRSEAEKMY QIKYEELQTV AGKHGDDLRN TKNEINELNR MITRIQSEID
321 GLKGQRASLE AAITEAEERG ELAVKDANNK LAELEAALQR AKQDMARQLR EYQELMNVKL ALDIEIATYR KLLEGEESRL
401 ESGMQNLSIQ TKTTGYSGGL SSGFSSYGGG YSSSYSSGGY SINALPLESS AISKTKAIVI KKIETRDGKL VSESSDVLSN
```

Supporting Peptides:

Peptide	Uniq	-10lgP	Mass	Length	ppm	m/z	z	RT	Fraction	Scan	Source File	Area F1	Area F2	Area F3	Are
R.AQYEEIANR.S	Y	73.65	1092.5199	9	0.5	547.2675	2	27.32	1	F1:7637	12112021_RID_2349_DP1.raw	6.6638E5	2.0438E5	3.7003E5	1.43
K.LALDIEIATYR.K	N	59.31	1276.7026	11	1.2	639.3594	2	57.61	4	F4:17049	17112021_RID_2349_DP4.raw				0

total 2 peptide

XP_015672252.1
back to list

| Protein Coverage | Supporting Peptides |
Protein Coverage:

1 MMRKNYRNTS STVRSFSSRS SGPVASSRIS TSYSSAGSGF GGSRYGGGAS FRGTSAGSLG GITAVSVNKS LLAPLNLEID
81 PAIQQVRIKE KEEIKTLNNK FANLIDKVRF LEQQNKMLET KWSLLQNQKT TRSNMDSMFE AYINNLRRQL ENLGQERARL
161 DAELGNMQGL VEEFKNKYED EINHRTEKEN EFVLLKKDVD EAYMNKVELE SRMECLTDEI NFLRQFYDEE LRVLQSQISD
241 TSVILSMDNN RSLDLDGIIT DVK**AQYEEIA NR**SRAEAERM YQIKYEELQT VAGKHGDDLR STKNEINELS RMINRIQSEI
321 DGLKGQRASL EAAITEAEER GELAVKDANN KLAELESAMQ RAKQDMARQL REYQELLNTK **LALDIEIATY R**KLLEGEEIR
401 LESGMQNLSI QTKTTGYSGG LSSGFSGYGG GYNSSYSSGG YSMNMLPMES SAISKTKAIV IKKIETRDGK LVSESSDVLS
481 K

Supporting Peptides:

Peptide	Uniq	-10lgP	Mass	Length	ppm	m/z	z	RT	Fraction	Scan	Source File	Area F1	Area F2	Area F3	Are
K.AQYEEIANR.S	Y	73.65	1092.5199	9	0.5	547.2675	2	27.32	1	F1:7637	12112021_RID_2349_DP1.raw	6.6638E5	2.0438E5	3.7003E5	1.43
K.LALDIEIATYR.K	N	59.31	1276.7026	11	1.2	639.3594	2	57.61	4	F4:17049	17112021_RID_2349_DP4.raw				0

total 2 peptide

JAG44290.1
back to list

| Protein Coverage | Supporting Peptides |
Protein Coverage:

1 MMRKNYRNTS NTVRTFSSRS SGPIASSRIS TSYSCSGFGG GRYGSGASFR GPSAGSLGGI TAVSVNKSLL APLNLEIDPA
81 IQQVRTKEKE EIKILNNKFA NLIDKVRFLE QQNKMLETKW SLLQNQKTTR SNMDSMFEAY INTLRRQLEN LGQERARLGA
161 ELGNMQGLVE EFKNKYEDEI NHRTEKENEF VLLKKDVDEA YMNKVELESR MECLTDEINF LRQFYDEELR VLQSQISDTS
241 VILSMDNNRS LDLDGIITDV K**AQYEEIANR** SRAEAERMYQ IKYEELQTVA GKHGDDLRCT KNEINELSRM ITRIQSEIDG
321 LKGQRASLEA AITEAEERGE LAVKDANTKL AELECAMQRA KQDMARQLRE YQELLNTK**LA LDIEIATYR**K LLEGEEIRLE
401 SGMQNLSIQT KTTGYSGGLS CGFSGYGGGY NPSYSSGGYS MNMLPMESSA VSKTKAIVIK KIETRDGKLV SESSDVLSN

Supporting Peptides:

Peptide	Uniq	-10lgP	Mass	Length	ppm	m/z	z	RT	Fraction	Scan	Source File	Area F1	Area F2	Area F3	Are
K.AQYEEIANR.S	Y	73.65	1092.5199	9	0.5	547.2675	2	27.32	1	F1:7637	12112021_RID_2349_DP1.raw	6.6638E5	2.0438E5	3.7003E5	1.43
K.LALDIEIATYR.K	N	59.31	1276.7026	11	1.2	639.3594	2	57.61	4	F4:17049	17112021_RID_2349_DP4.raw				0

total 2 peptide

TRINITY_DN6008
back to list

| Protein Coverage | Supporting Peptides |
Protein Coverage:

1 MKNYRNTSST VRSFSSRSYT PGPVASSRIS SSYSSSGFGG GSRYGGGASF RGASAGSLGG ITAVSVNKSL LAPLNLEIDP
81 AMQQVRTKEK EEIKTLNNKF ANLIDKVRFL EQQNKMLETK WSLMQNQKTT RSNMDSMFEA YINTLRRQLD NLGQERARLD
161 AELANMQGLV EEFKNKYEDE INHRTEKENE FVLLKKDVDE AYMNKVELES RMECMTDEVN FLRQLYDEEL RVLQSQISDT
241 SVILSMDNNR SLNLEGIIAD VK**AQYEEIAN R**SRAEAERMY QIKYEELQNV AGKHGDDLRS TKNEINELNR MISRIQSEID
321 GLKGQRASLE AAITEAEERG ELAVKDANAK LAELESAMQR AKQDMARQLR EYQELLNTK**L ALDIEIATYR** KLLEGEEIRL
401 ESGMQNLSIQ TKTTGYSGGL SSGFSGYGGG YSSSYSGGGY SINMLPMESS AIGKTKAIVI KKIETRDGKL VSESSDVLSN

Supporting Peptides:

Peptide	Uniq	-10lgP	Mass	Length	ppm	m/z	z	RT	Fraction	Scan	Source File	Area F1	Area F2	Area F3	Are
K.AQYEEIANR.S	Y	73.65	1092.5199	9	0.5	547.2675	2	27.32	1	F1:7637	12112021_RID_2349_DP1.raw	6.6638E5	2.0438E5	3.7003E5	1.43
K.LALDIEIATYR.K	N	59.31	1276.7026	11	1.2	639.3594	2	57.61	4	F4:17049	17112021_RID_2349_DP4.raw				0

total 2 peptide

AFJ50411.1
back to list

| Protein Coverage | Supporting Peptides |
Protein Coverage:

1 MMRKNYRNTS NTVRTFSSRS SGPIASSRIS TSYSCSGFGG GRYGSGASFR GPSAGSLGGI TAVSVNKSLL APLNLEIDPA
81 IQQVRTKEKE EIKILNNKFA NLIDKVRFLE QQNKMLETKW SLLQNQKTTR SNMDSMFEAY INTLRRQLEN LGQERARLGA
161 ELGNMQGLVE EFKNKYEDEI NHRTEKENEF VLLKKDVDEA YMNKVELESR MECLTDEINF LRQFYDEELR VLQSQISDTS
241 VILSMDNNRS LDLDGIITDV K**AQYEEIANR** SRAEAERMYQ IKYEELQTVA GKHGDDLRCT KNEINELSRM ITRIQSEIDG
321 LKGQRASLEA AITEAEERGE LAVKDANTKL AELECAMQRA KQDMARQLRE YQELLNTK**LA LDIEIATYR**K LLEGEEIRLE
401 SGMQNLSIQT KTTGYSGGLS CGFSGYGGGY NSSYSSGGYS MNMLPMESSA VSKTKAIVIK KIETRDGKLV SESSDVLSN

Supporting Peptides:

Peptide	Uniq	-10lgP	Mass	Length	ppm	m/z	z	RT	Fraction	Scan	Source File	Area F1	Area F2	Area F3	Are
K.AQYEEIANR.S	Y	73.65	1092.5199	9	0.5	547.2675	2	27.32	1	F1:7637	12112021_RID_2349_DP1.raw	6.6638E5	2.0438E5	3.7003E5	1.43
K.LALDIEIATYR.K	N	59.31	1276.7026	11	1.2	639.3594	2	57.61	4	F4:17049	17112021_RID_2349_DP4.raw				0

total 2 peptide

TRINITY_DN2736
back to list

| Protein Coverage | Supporting Peptides |
Protein Coverage:

1 AASVTLDLFG DWRDKNLLRR KR**HDNAQLLT GIDLNR**QTLG IAHVEGMCNG KLSVGVIQDY CKSYLLVAIT MAHEMGHNLG

81 MKHDNANCNC PDTSCIMS

Supporting Peptides:

Peptide	Uniq	-10lgP	Mass	Length	ppm	m/z	z	RT	Fraction	Scan	Source File	Area F1	Area F2	Area F
R.HDNAQLLTGIDLNR.Q	Y	77.18	1578.8114	14	0.9	790.4137	2	46.31	2	F2:13153	12112021_RID_2349_DP2.raw	1.8674E5	1.5439E6	7.1135E
D.NAQLLTGIDLNR.Q	Y	51.88	1326.7256	12	1.0	664.3707	2	48.90	2	F2:13982	12112021_RID_2349_DP2.raw		1.8287E4	

total 2 peptides

TRINITY_DN471
back to list

| Protein Coverage | Supporting Peptides |
Protein Coverage:

1 MCQPLR**SVAI VEDYSPIDR**V VAAIMAHEMG HNLGITHDEN PCSCGANSCI MAPQISDPPP MKFSDCSWDQ YQNFLTNDKA

81 NCTLIRPSRT DIVSPPACGN ELLEVGEECD CGSP

Supporting Peptides:

Peptide	Uniq	-10lgP	Mass	Length	ppm	m/z	z	RT	Fraction	Scan	Source File	Area F1	Area F2	Area F3	
R.SVAIVEDYSPIDR.V	Y	83.77	1462.7303	13	0.2	732.3726	2	47.47	2	F2:13537	12112021_RID_2349_DP2.raw	4.0439E6	6.1514E6	1.3556E7	4

total 1 peptides

TRINITY_DN1497
back to list

| Protein Coverage | Supporting Peptides |
Protein Coverage:

1 MTDLEREMGL PPIEQKLGLQ IWTINNMKMD PIPAKAYGSF FEGDCYIVLN NSKRQHSFAT DLHYWIGRTA TQDEQGAAAF

81 YVTQMDDSLG GRAIQHREVQ GHESAAFKSY FKKGIIYKKG GLASGFKHVE TNMYNIKRLL HVKGKKHVTA TEVDLSWDSF

161 NKGDVFLMDL GKVLIQWNGP FCNVAEKSKG MILARSIRDG ERGGRAQIAI IDNEKDCPEL MQIMKATLGE RTQEIREPLT

241 DEKADEHQKA NVRLYHVYEN GKDLVVQEIA TRPLTQDLLS HEDCHIIDQG GLKIYVWRGK ESSKEERKAA FTRAVGFIQA

321 KGYPPSTNVE VVNDGAESAM FKQLFQKWTE KYTTQGLGKA YTLGKIAKVD QAKFDITQLY VTPEVAAEER MPDDASGKVE

401 VWRIENREMV PVNPKTYGQF YSGDCYLVLY TYTKSGRPHY IIYMWQGRHA SVDEITACAF NAVELDKK**YG DEPVQVR**VTM

481 GKEPRHFLAI FKGNLIIYEG GISHGQKMGP EPAIQLFQVR GTDEFNTKTV EVPARASSLN SNDVFILKTN QVCYLWCGKG

561 CSGDEREMAK TVADVISKWD KQTILEGQEP AEFWVALGGK APYASDKRFQ ERVPHYQSRL FECSNQTGRF IMTEIVDFGQ

641 DDLDEEDVML LDTWEEVFLW VGKAANSYEK TESISAAKEY LKNHPAGR**DA ATPIIMVK**QG HEPLNFTGWF TAWDPYKWSD

721 EKSYEQMKSS LGDVSAVSEI TMDLKNANFN HRSTSNKPVT ANADISEARS SMQDDQTFTN SSNCYNANTP CQEPNETGTF

801 PRDLLINKTV DELPDGVDPT KKEYYLSDAE FCDIFGKTKD AFYQMPAWKQ QNEKKQYGLF

Supporting Peptides:

Peptide	Uniq	-10lgP	Mass	Length	ppm	m/z	z	RT	Fraction	Scan	Source File	Area F1	Area F2	Area F3	Area F4	Area F5	A l
K.YGDEPVQVR.V	Y	57.76	1061.5142	9	0.2	531.7645	2	29.19	1	F1:8248	12112021_RID_2349_DP1.raw	4.705E4					
R.DAATPIIMVK.Q	Y	48.71	1057.5841	10	0.8	529.7997	2	45.65	1	F1:13790	12112021_RID_2349_DP1.raw	8.7907E4					

total 2 peptides

TRINITY_DN1497
back to list

| Protein Coverage | Supporting Peptides |
Protein Coverage:

1 MYNIKRLLHV KGKKHVTATE VDLSWDSFNK GDVFLMDLGK VLIQWNGPFC NVAEKSKGMI LARSIRDGER GGRAQIAIID
81 NEKDCPELMQ IMKATLGERT QEIREPLTDE KADEHQKANV RLYHVYENGK DLVVQEIATR PLTQDLLSHE DCHIIDQGGL
161 KIYVWRGKES SKEERKAAFT RAVGFIQAKG YPPSTNVEVV NDGAESAMFK QLFQKWTEKY TTQGLGKAYT LGKIAKVDQA
241 KFDITQLYVT PEVAAEERMP DDASGKVEVW RIENREMVPV NPKTYGQFYS GDCYLVLYTY TKSGRPHYII YMWQGRHASV
321 DEITACAFNA VELDKK**YGDE PVQVR**VTMGK EPRHFLAIFK GNLIIYEGGI SHGQKMGPEP AIQLFQVRGT DEFNTKTVEV
401 PARASSLNSN DVFILKTNQV CYLWCGKGCS GDEREMAKTV ADVISKWDKQ TILEGQEPAE FWVALGGKAP YASDKRFQER
481 VPHYQSRLFE CSNQTGRFIM TEIVDFGQDD LDEEDVMLLD TWEEVFLWVG KAANSYEKTE SISAAKEYLK NHPAGR**DAAT**
561 **PIIMVK**QGHE PLNFTGWFTA WDPYKWSDEK SYEQMKSSLG DVSAVSEITM DLKNANFNHR STSNKPVTAN ADISEARSSM
641 QDDQTFTNSS NCYNANTPCQ EPNETGTFPR DLLINKTVDE LPDGVDPTKK EYYLSDAEFC DIFGKTKDAF YQMPAWKQQN
721 EKKQYGLF

Supporting Peptides:

Peptide	Uniq	-10lgP	Mass	Length	ppm	m/z	z	RT	Fraction	Scan	Source File	Area F1	Area F2	Area F3	Area F4	Area F5	A
K.YGDEPVQVR.V	Y	57.76	1061.5142	9	0.2	531.7645	2	29.19	1	F1:8248	12112021_RID_2349_DP1.raw	4.705E4					
R.DAATPIIMVK.Q	Y	48.71	1057.5841	10	0.8	529.7997	2	45.65	1	F1:13790	12112021_RID_2349_DP1.raw	8.7907E4					

total 2 peptides

ETE68218.1
back to list

| Protein Coverage | Supporting Peptides |
Protein Coverage:

1 MEVMNIIDQP IKVTEWQQTY TYDSGIHSGM NTQVPSVSSK GLMDEDDLYG KQYTFKKTTY SQPGSGQTQT QAELESQLAM
81 TRAQRIRAAM YPETAEDHTL LLTTQVEGQQ TNVQRLAEPS QMLKSAIVHL INYQDDAELA TRAIPELTKL LNDEDPVVVS
161 KAAMIVNQLS KKEASRRALM QSAQIVAAVV R**TMQNTSDLD TAR**CTTSILH NLSHHREGLL SIFKSGGIPA LVRMLSSPVE
241 SVLFYAITTL HNLLLYQEGA KMAVRLADGL QKMVPLLTKN NPKFLAITTD CLQLLAYGNQ ESKLIILANG GPQALVQIMR
321 SYTYEKLLWT TSRVLKVLSV CPSNKPAIVE AGGMQALGKH LTSSSPRLVQ NCLWTLRNLS DVATKQEGLD GVLKILVNQL
401 SSDDIHVLTC ATGTLSNLTC NNSKNKTLVT QSNGVEALIH TILRAGDKED ITEPAVCALR HLTSRHPEAE MAQNSVR**LNY**
481 **GIPAIVK**LLN QPNQWPLIKA TIGLIRNLAL CPANHAPLQE AAVIPRLVQL LVKAHQDAQR HAAAGTQQPY TDGVKMEEIV
561 EGCTGALHIL ARDPMNRMEI FRLNTIPLFV QVRISEWMEV LLYSPVENIQ RVAAGVLCEL AQDKEAADAI DAEGASAPLM
641 ELLHSRNEGT ATYAAAVLFR **ISEDKNPDYR** KRVSVELTNS LFKHDPAAWE AAQSMIPIHE PYADELDGGY RGMYSGEIPM
721 DPMDMHMDMD GDYPMDTYSD GVRAPYPDHM LA

Supporting Peptides:

Peptide	Uniq	-10lgP	Mass	Length	ppm	m/z	z	RT	Fraction	Scan	Source File	Area F1	Area F2	Area F3	Area F4
R.LNYGIPAIVK.L	Y	54.39	1086.6437	10	0.2	544.3292	2	49.42	6	F6:14436	17112021_RID_2349_DP6.raw	5.5682E4			1.1942E5
R.TMQNTSDLDTAR.C	Y	50.31	1351.6038	12	0.9	676.8098	2	26.03	6	F6:6391	17112021_RID_2349_DP6.raw				
R.ISEDKNPDYR.K	Y	46.51	1235.5781	10	1.3	618.7971	2	18.97	6	F6:3975	17112021_RID_2349_DP6.raw				

total 3 peptides

XP_025022318.1
back to list

| Protein Coverage | Supporting Peptides |
Protein Coverage:

1 MDVMNLIEQP IKVTEWQQTY TYDSGIHSGM NTQVPSVSSK GLMDEDDLYG KQYTIKKTTT YSQAGPGQAQ AQAQELESQL
81 AMTRAQRIRA AMYPETVEDH SLLVTTQVEG QQTNVQRLAE PSQMLKSAIV HLINYQDDAE LATRAIPELT KLLNDEDPVV
161 VSKAAMIVNQ LSKKEASRRA LMQSAQIVAA VVR**TMQNTSD LDTAR**CTTSI LHNLSHHREG LLSIFKSGGI PALVRMLSSP
241 VESVLFYAIT TLHNLLLYQE GAKMAVRLAD GLQKMVPLLT KNNPKFLAIT TDCLQLLAYG NQESKLIILA NGGPQALVQI
321 MRSYTYEKLL WTTSRVLKVL SVCPSNKPAI VEAGGMQALG KHLTSSSPRL VQNCLWTLRN LSDVATKQEG LDGVLKILVN
401 QLSSDDINVL TCATGTLSNL TCNNSKNKTL VTQSNGVEAL IHTILRAGDK EDITEPAVCA LRHLTSRHPE AEMAQNSVR**L**
481 **NYGIPAIVK**L LNQPNQWPLI KATIGLIRNL ALCPANHAPL QEAAVIPRLV QLLVKAHQDA QRHAAAGTQQ PYTDGVKMEE
561 IVEGCTGALH ILARDPMNRM EIFRLNTIPL FVQLLYSPVE NIQRVAAGVL CELAQDKEAA DAIDAEGASA PLMELLHSRN
641 EGTATYAAAV LFR**ISEDKNP DYR**KRVSVEL TNSLFKHDPA AWEAAQSMIP IHEPYADELD GGYRGMYPGE IPMDPMDMHM
721 DMDGDYPMDT YSDGVRAPYP DHMLA

Supporting Peptides:

Peptide	Uniq	-10lgP	Mass	Length	ppm	m/z	z	RT	Fraction	Scan	Source File	Area F1	Area F2	Area F3	Area F4

Peptide	Uniq	-10lgP	Mass	Length	ppm	m/z	z	RT	Fraction	Scan	Source File	Area F1	Area F2	Area F3	Area F4
R.LNYGIPAIVK.L	Y	54.39	1086.6437	10	0.2	544.3292	2	49.42	6	F6:14436	17112021_RID_2349_DP6.raw	5.5682E4			1.1942E5
R.TMQNTSDLDTAR.C	Y	50.31	1351.6038	12	0.9	676.8098	2	26.03	6	F6:6391	17112021_RID_2349_DP6.raw				
R.ISEDKNPDYR.K	Y	46.51	1235.5781	10	1.3	618.7971	2	18.97	6	F6:3975	17112021_RID_2349_DP6.raw				

total 3 peptides

JAG67632.1
back to list

| Protein Coverage | Supporting Peptides |
Protein Coverage:

1 MDMMNIIDQP IKVTEWQQTY TYDSGIHSGM NTQVPSVSSK GLMDEDDLYG KQYTIKKTTT YSQPGSGQGQ TQAELESQLA
81 MTRAQRIRAA MYPETAEDHT LLLTTQVEGQ QTNVQRLAEP SQMLKSAIVH LINYQDDAEL ATRAIPELTK LLNDEDPVVV
161 SKAAMIVNQL SKKEASRRAL MQSAQIVAAV VR**TMQNTSDL DTAR**CTTSIL HNLSHHREGL LSIFKSGGIP ALVRMLSSPV
241 ESVLFYAITT LHNLLLYQEG AKMAVRLADG LQKMVPLLTK NNPKFLAITT DCLQLLAYGN QESKLIILAN GGPQALVQIM
321 RSYTYEKLLW TTSRVLKVLS VCPSNKPAIV EAGGMQALGK HLTSSSPRLV QNCLWTLRNL SDVATKQEGL DGVLKILVNQ
401 LSSDDINVLT CATGTLSNLT CNNSKNKTLV TQSNGVEALI HTILRAGDKE DITEPAVCAL RHLTSRHPEA EMAQNSVR**LN**
481 **YGIPAIVK**LL NQPNQWPLIK ATIGLIRNLA LCPANHAPLQ EAAVIPRLVQ LLVKAHQDAQ RHAAAGTQQP YTDGVKMEEI
561 VEGCTGALHI LARDPMNRME IFRLNTIPLF VQLLYSPVEN IQRVAAGVLC ELAQDKEAAD AIDAEGASAP LMELLHSRNE
641 GTATYAAAVL FR**ISEDKNPD YR**KRVSVELT NSLFKHDPAA WEAAQSMIPI HEPYADELDG GYRGMYSGEI PIDPMDLHMD
721 MDGDYPMDTY SDGVRAPYPD HMLA

Supporting Peptides:

Peptide	Uniq	-10lgP	Mass	Length	ppm	m/z	z	RT	Fraction	Scan	Source File	Area F1	Area F2	Area F3	Area F4
R.LNYGIPAIVK.L	Y	54.39	1086.6437	10	0.2	544.3292	2	49.42	6	F6:14436	17112021_RID_2349_DP6.raw	5.5682E4			1.1942E5
R.TMQNTSDLDTAR.C	Y	50.31	1351.6038	12	0.9	676.8098	2	26.03	6	F6:6391	17112021_RID_2349_DP6.raw				
R.ISEDKNPDYR.K	Y	46.51	1235.5781	10	1.3	618.7971	2	18.97	6	F6:3975	17112021_RID_2349_DP6.raw				

total 3 peptides

JAG44306.1
back to list

| Protein Coverage | Supporting Peptides |
Protein Coverage:

1 MEVMNIIDQP IKVTEWQQTY TYDSGIHSGM NTQVPSVSSK GLMDEDDLYG KQYTIKKTTT YSQPGSGQAQ TQAELESQLA
81 MTRAQRIRAA MYPETAEDHT LLLTTQVEGQ QTNVQRLAEP SQMLKSAIVH LINYQDDAEL ATRAIPELTK LLNDEDPVVV
161 SKAAMIVNQL SKKEASRRAL MQSAQIVAAV VR**TMQNTSDL DTAR**CTTSIL HNLSHHREGL LSIFKSGGIP ALVRMLSSPV
241 ESVLFYAITT LHNLLLYQEG AKMAVRLADG LQKMVPLLTK NNPKFLAITT DCLQLLAYGN QESKLIILAN GGPQALVQIM
321 RSYTYEKLLW TTSRVLKVLS VCPSNKPAIV EAGGMQALGK HLTSSSPRLV QNCLWTLRNL SDVATKQEGL DGVLKILVNQ
401 LSSDDINVLT CATGTLSNLT CNNSKNKTLV TQSNGVEALI HTILRAGDKE DITEPAVCAL RHLTSRHPEA EMAQNSVR**LN**
481 **YGIPAIVK**LL NQPNQWPLIK ATIGLIRNLA LCPANHAPLQ EAAVIPRLVQ LLVKAHQDAQ RHAAAGTQQP YTDGVKMEEI
561 VEGCTGALHI LARDPMNRME IFRLNTIPLF VQLLYSPVEN IQRVAAGVLC ELAQDKEAAD AIDAEGASAP LMELLHSRNE
641 GTATYAAAVL FR**ISEDKNPD YR**KRVSVELT NSLFKHDPAA WEAAQSMIPI HEPYADELDA GYRGMYSAEI PMDPMDMHLD
721 MDGDYPMDTY SDGVRAPYPD HMLA

Supporting Peptides:

Peptide	Uniq	-10lgP	Mass	Length	ppm	m/z	z	RT	Fraction	Scan	Source File	Area F1	Area F2	Area F3	Area F4
R.LNYGIPAIVK.L	Y	54.39	1086.6437	10	0.2	544.3292	2	49.42	6	F6:14436	17112021_RID_2349_DP6.raw	5.5682E4			1.1942E5
R.TMQNTSDLDTAR.C	Y	50.31	1351.6038	12	0.9	676.8098	2	26.03	6	F6:6391	17112021_RID_2349_DP6.raw				
R.ISEDKNPDYR.K	Y	46.51	1235.5781	10	1.3	618.7971	2	18.97	6	F6:3975	17112021_RID_2349_DP6.raw				

total 3 peptides

JAA96758.1
back to list

| Protein Coverage | Supporting Peptides |
Protein Coverage:

```
  1 MEVMNIIDQP IKVTEWQQTY TYDSGIHSGM NTQVPSVSSK GLMDEDDLYG KQYTIKKTTT YSQPGSGQAQ TQAELESQLA
 81 MTRAQRIRAA MYPETAEDHT LLLTTQVEGQ QTNVQRLAEP SQMLKSAIVH LINYQDDAEL ATRAIPELTK LLNDEDPVVV
161 SKAAMIVNQL SKKEASRRAL MQSAQIVAAV VRTMQNTSDL DTARCTTSIL HNLSHHREGL LSIFKSGGIP ALVRMLSSPV
241 ESVLFYAITT LHNLLLYQEG AKMAVRLADG LQKMVPLLTK NNPKFLAITT DCLQLLAYGN QESKLIILAN GGPQALVQIM
321 RSYTYEKLLW TTSRVLKVLS VCPSNKPAIV EAGGMQALGK HLTSSSPRLV QNCLWTLRNL SDVATKQEGL DGVLKILVNQ
401 LSSDDINVLT CATGTLSNLT CNNSKNKTLV TQSNGVEALI HTILRAGDKE DITEPAVCAL RHLTSRHPEA EMAQNSVRLN
481 YGIPAIVKLL NQPNQWPLIK ATIGLIRNLA LCPANHAPLQ EAAVIPRLVQ LLVKAHQDAQ RHAAAGTQQP YTDGVKMEEI
561 VEGCTGALHI LARDPMNRME IFRLNTIPLF VQLLYSPVEN IQRVAAGVLC ELAQDKEAAD AIDAEGASAP LMELLHSRNE
641 GTATYAAAVL FRISEDKNPD YRKRVSVELT NSLFKHDPAA WEAAQSMIPI HEPYADELDA GYRGMYSAEI PMDPMDMHLD
721 MDGDYPMDTY SDGVRAPYPD HMLA
```

Supporting Peptides:

Peptide	Uniq	-10lgP	Mass	Length	ppm	m/z	z	RT	Fraction	Scan	Source File	Area F1	Area F2	Area F3	Area F4
R.LNYGIPAIVK.L	Y	54.39	1086.6437	10	0.2	544.3292	2	49.42	6	F6:14436	17112021_RID_2349_DP6.raw	5.5682E4			1.1942E5
R.TMQNTSDLDTAR.C	Y	50.31	1351.6038	12	0.9	676.8098	2	26.03	6	F6:6391	17112021_RID_2349_DP6.raw				
R.ISEDKNPDYR.K	Y	46.51	1235.5781	10	1.3	618.7971	2	18.97	6	F6:3975	17112021_RID_2349_DP6.raw				

total 3 peptides

JAG46538.1
back to list

| Protein Coverage | Supporting Peptides |

Protein Coverage:

```
  1 MEVMNIIDQP IKVTEWQQTY TYDSGIHSGM NTQVPSVSSK GLMDEDDLYG KQYTIKKTTT YSQPGSGQAQ TQAELESQLA
 81 MTRAQRIRAA MYPETAEDHT LLLTTQVEGQ QTNVQRLAEP SQMLKSAIVH LINYQDDAEL ATRAIPELTK LLNDEDPVVV
161 SKAAMIVNQL SKKEASRRAL MQSAQIVAAV VRTMQNTSDL DTARCTTSIL HNLSHHREGL LSIFKSGGIP ALVRMLSSPV
241 ESVLFYAITT LHNLLLYQEG AKMAVRLADG LQKMVPLLTK NNPKFLAITT DCLQLLAYGN QESKLIILAN GGPQALVQIM
321 RSYTYEKLLW TTSRVLKVLS VCPSNKPAIV EAGGMQALGK HLTSSSPRLV QNCLWTLRNL SDVATKQEGL DGVLKILVNQ
401 LSSDDINVLT CATGTLSNLT CNNSKNKTLV TQSNGVEALI HTILRAGDKE DITEPAVCAL RHLTSRHPEA EMAQNSVRLN
481 YGIPAIVKLL NQPNQWPLIK ATIGLIRNLA LCPANHAPLQ EAAVIPRLVQ LLVKAHQDAQ RHAAAGTQQP YTDGVKMEEI
561 VEGCTGALHI LARDPMNRME IFRLNTIPLF VQLLYSPVEN IQRVAAGVLC ELAQDKEAAD AIDAEGASAP LMELLHSRNE
641 GTATYAAAVL FRISEDKNPD YRKRVSVELT NSLFKHDPAA WEAAQSMIPI HEPYADELDA GYRGMYSAEI PMDPMDMHLD
721 MDGDYPMDTY SDGVRAPYPD HMLA
```

Supporting Peptides:

Peptide	Uniq	-10lgP	Mass	Length	ppm	m/z	z	RT	Fraction	Scan	Source File	Area F1	Area F2	Area F3	Area F4
R.LNYGIPAIVK.L	Y	54.39	1086.6437	10	0.2	544.3292	2	49.42	6	F6:14436	17112021_RID_2349_DP6.raw	5.5682E4			1.1942E5
R.TMQNTSDLDTAR.C	Y	50.31	1351.6038	12	0.9	676.8098	2	26.03	6	F6:6391	17112021_RID_2349_DP6.raw				
R.ISEDKNPDYR.K	Y	46.51	1235.5781	10	1.3	618.7971	2	18.97	6	F6:3975	17112021_RID_2349_DP6.raw				

total 3 peptides

LAB43566.1
back to list

| Protein Coverage | Supporting Peptides |

Protein Coverage:

```
  1 STWGERGGVE RGNQGGGDGA GRIAMEVVNI IDQPIKVTEW QQTYTYDSGI HSGMNTQVPS VSSKGLMDED DLYGNQYTIK
 81 KTTTYSQPGS GKAQTQAELE SQLAMTRAQR IRAAMYPETT EDHSLLVTTQ VEGQQTNVQR LAEPSQMLKS AIVHLINYQD
161 DAELATRAIP ELTKLLNDED PVVVSKAAMI VNQLSKKEAS RRALMQSAQI VAAVVRTMQN TSDLDTARCT TSILHNLSHH
241 REGLLSIFKS GGIPALVRML SSPVESVLFY AITTLHNLLL YQEGAKMAVR LADGLQKMVP LLTKNNPKFL AITTDCLQLL
321 AYGNQESKLI ILANGGPQAL VQIMRSYTYE KLLWTTSRVL KVLSVCPSNK PAIVEAGGMQ ALGKHLTSSS PRLVQNCLWT
401 LRNLSDVATK QEGLDGVLKI LVNQLSSDDI HVLTCATGTL SNLTCNNSKN KTLVTQSNGV EALIHTILRA GDKEDITEPA
481 VCALRHLTSR HPEAEMAQNS VRLNYGIPAI VKLLNQPNQW PLIKATIGLI RNLALCPANH APLQEAAVIP RLVQLLVKAH
561 AAAGTQQPYT DGVKMEEIVE GCTGALHILA RDPMNRMEIF RLNTIPLFVQ LLYSPVENIQ RVAAGVLCEL AQDKEAADAI
641 DAEGASAPLM ELLHSRNEGT ATYAAAVLFR ISEDKNPDYR KRVSVELTNS LFKHDPAAWE AAQSMIPIHE PYADELDGGY
721 RGMYSGEIPM DPMDMHMDMD GDYPMDTYSD GVRAPYPDHM LA
```

Supporting Peptides:

Peptide	Uniq	-10lgP	Mass	Length	ppm	m/z	z	RT	Fraction	Scan	Source File	Area F1	Area F2	Area F3	Area F4
R.LNYGIPAIVK.L	Y	54.39	1086.6437	10	0.2	544.3292	2	49.42	6	F6:14436	17112021_RID_2349_DP6.raw	5.5682E4			1.1942E5
R.TMQNTSDLDTAR.C	Y	50.31	1351.6038	12	0.9	676.8098	2	26.03	6	F6:6391	17112021_RID_2349_DP6.raw				
R.ISEDKNPDYR.K	Y	46.51	1235.5781	10	1.3	618.7971	2	18.97	6	F6:3975	17112021_RID_2349_DP6.raw				

total 3 peptides

JAV50234.1
back to list

| Protein Coverage | Supporting Peptides |
Protein Coverage:

```
  1 MEVMNIIDQP IKVTEWQQTY TYDSGIHSGM NTQVPSVSSK GLMDEDDLYG KQYTIKKTTT YSQPGSGQAQ TQAELESQLA
 81 MTRAQRIRAA MYPETAEDHT LLLTTQVEGQ QTNVQRLAEP SQMLKSAIVH LINYQDDAEL ATRAIPELTK LLNDEDPVVV
161 SKAAMIVNQL SKKEASRRAL MQSAQIVAAV VRTMQNTSDL DTARCTTSIL HNLSHHREGL LSIFKSGGIP ALVRMLSSPV
241 ESVLFYAITT LHNLLLYQEG AKMAVRLADG LQKMVPLLTK NNPKFLAITT DCLQLLAYGN QESKLIILAN GGPQALVQIM
321 RSYTYEKLLW TTSRVLKVLS VCPSNKPAIV EAGGMQALGK HLTSSSPRLV QNCLWTLRNL SDVATKQEGL DGVLKILVNQ
401 LSSDDINVLT CATGTLSNLT CNNSKNKTLV TQSNGVEALI HTILRAGDKE DITEPAVCAL RHLTSRHPEA EMAQNSVRLN
481 YGIPAIVKLL NQPNQWPLIK ATIGLIRNLA LCPANHAPLQ EAAVIPRLVQ LLVKAHQDAQ RHAAAGTQQP YTDGVKMEEI
561 VEGCTGALHI LARDPMNRLE IFRLNTIPLF VQLLYSPVEN IQRVAAGVLC ELAQDKEAAD AIDAEGASAP LMELLHSRNE
641 GTATYAAAVL FRISEDKNPD YRKRVSVELT NSLFKHDPAA WEAAQSMIPI HEPYADELDA GYRAMYSAEI PMDALDMDGD
721 YPMDTYSDGV RAPYPDHMLA
```

Supporting Peptides:

Peptide	Uniq	-10lgP	Mass	Length	ppm	m/z	z	RT	Fraction	Scan	Source File	Area F1	Area F2	Area F3	Area F4
R.LNYGIPAIVK.L	Y	54.39	1086.6437	10	0.2	544.3292	2	49.42	6	F6:14436	17112021_RID_2349_DP6.raw	5.5682E4			1.1942E5
R.TMQNTSDLDTAR.C	Y	50.31	1351.6038	12	0.9	676.8098	2	26.03	6	F6:6391	17112021_RID_2349_DP6.raw				
R.ISEDKNPDYR.K	Y	46.51	1235.5781	10	1.3	618.7971	2	18.97	6	F6:3975	17112021_RID_2349_DP6.raw				

total 3 peptides

XP_026576301.1
back to list

| Protein Coverage | Supporting Peptides |
Protein Coverage:

```
  1 MEVMNIIDQP IKVTEWQQTY TYDSGIHSGM NTQVPSVSSK GLMDEDDLYG KQYTIKKTTT YSQPGSGQAQ TQELESQLAM
 81 TRAQRIRAAM YPETAEDHTL LLTTQVEGQQ TNVQRLAEPS QMLKSAIVHL INYQDDAELA TRAIPELTKL LNDEDPVVVS
161 KAAMIVNQLS KKEASRRALM QSAQIVAAVV RTMQNTSDLD TARCTTSILH NLSHHREGLL SIFKSGGIPA LVRMLSSPVE
241 SVLFYAITTL HNLLLYQEGA KMAVRLADGL QKMVPLLTKN NPKFLAITTD CLQLLAYGNQ ESKLIILANG GPQALVQIMR
321 SYTYEKLLWT TSRVLKVLSV CPSNKPAIVE AGGMQALGKH LTSSSPRLVQ NCLWTLRNLS DVATKQEGLD GVLKILVNQL
401 SSDDIHVLTC ATGTLSNLTC NNSKNKTLVT QSNGVEALIH TILRAGDKED ITEPAVCALR HLTSRHPEAE MAQNSVRLNY
481 GIPAIVKLLN QPNQWPLIKA TIGLIRNLAL CPANHAPLQE AAVIPRLVQL LVKAHQDAQR HAAAGTQQPY TDGVKMEEIV
561 EGCTGALHIL ARDPMNRMEI FRLNTIPLFV QLLYSPVENI QRVAAGVLCE LAQDKEAADA IDAEGASAPL MELLHSRNEG
641 TATYAAAVLF RISEDKNPDY RKRVSVELTN SLFKHDPAAW EAAQSMIPIH EPYADELDGG YRGMYSGEIP MDAMDMDGDY
721 PMDTYSDGVR APYPDHMLA
```

Supporting Peptides:

Peptide	Uniq	-10lgP	Mass	Length	ppm	m/z	z	RT	Fraction	Scan	Source File	Area F1	Area F2	Area F3	Area F4
R.LNYGIPAIVK.L	Y	54.39	1086.6437	10	0.2	544.3292	2	49.42	6	F6:14436	17112021_RID_2349_DP6.raw	5.5682E4			1.1942E5
R.TMQNTSDLDTAR.C	Y	50.31	1351.6038	12	0.9	676.8098	2	26.03	6	F6:6391	17112021_RID_2349_DP6.raw				
R.ISEDKNPDYR.K	Y	46.51	1235.5781	10	1.3	618.7971	2	18.97	6	F6:3975	17112021_RID_2349_DP6.raw				

total 3 peptides

XP_026525656.1
back to list

| Protein Coverage | Supporting Peptides |
Protein Coverage:

1 MEVMNIIDQP IKVTEWQQTY TYDSGIHSGM NTQVPSVSSK GLMDEDDLYG KQYTIKKTTT YSQPGSGQAQ TQELESQLAM
81 TRAQRIRAAM YPETAEDHTL LLTTQVEGQQ TNVQRLAEPS QMLKSAIVHL INYQDDAELA TRAIPELTKL LNDEDPVVVS
161 KAAMIVNQLS KKEASRRALM QSAQIVAAVV R**TMQNTSDLD TAR**CTTSILH NLSHHREGLL SIFKSGGIPA LVRMLSSPVE
241 SVLFYAITTL HNLLLYQEGA KMAVRLADGL QKMVPLLTKN NPKFLAITTD CLQLLAYGNQ ESKLIILANG GPQALVQIMR
321 SYTYEKLLWT TSRVLKVLSV CPSNKPAIVE AGGMQALGKH LTSSSPRLVQ NCLWTLRNLS DVATKQEGLD GVLKILVNQL
401 SSDDIHVLTC ATGTLSNLTC NNSKNKTLVT QSNGVEALIH TILRAGDKED ITEPAVCALR HLTSRHPEAE MAQNSVR**LNY**
481 **GIPAIVK**LLN QPNQWPLIKA TIGLIRNLAL CPANHAPLQE AAVIPRLVQL LVKAHQDAQR HAAAGTQQPY TDGVKMEEIV
561 EGCTGALHIL ARDPMNRMEI FRLNTIPLFV QLLYSPVENI QRVAAGVLCE LAQDKEAADA IDAEGASAPL MELLHSRNEG
641 TATYAAAVLF R**ISEDKNPDY R**KRVSVELTN SLFKHDPAAW EAAQSMIPIH EPYADELDGG YRGMYSGEIP MDAMDMDGDY
721 PMDTYSDGVR APYPDHMLA

Supporting Peptides:

Peptide	Uniq	-10lgP	Mass	Length	ppm	m/z	z	RT	Fraction	Scan	Source File	Area F1	Area F2	Area F3	Area F4
R.LNYGIPAIVK.L	Y	54.39	1086.6437	10	0.2	544.3292	2	49.42	6	F6:14436	17112021_RID_2349_DP6.raw	5.5682E4			1.1942E5
R.TMQNTSDLDTAR.C	Y	50.31	1351.6038	12	0.9	676.8098	2	26.03	6	F6:6391	17112021_RID_2349_DP6.raw				
R.ISEDKNPDYR.K	Y	46.51	1235.5781	10	1.3	618.7971	2	18.97	6	F6:3975	17112021_RID_2349_DP6.raw				

total 3 peptides

XP_026576299.1
back to list

| Protein Coverage | Supporting Peptides |

Protein Coverage:

1 MEVMNIIDQP IKVTEWQQTY TYDSGIHSGM NTQVPSVSSK GLMDEDDLYG KQYTIKKTTT YSQPGSGQAQ TQAELESQLA
81 MTRAQRIRAA MYPETAEDHT LLLTTQVEGQ QTNVQRLAEP SQMLKSAIVH LINYQDDAEL ATRAIPELTK LLNDEDPVVV
161 SKAAMIVNQL SKKEASRRAL MQSAQIVAAV VR**TMQNTSDL DTAR**CTTSIL HNLSHHREGL LSIFKSGGIP ALVRMLSSPV
241 ESVLFYAITT LHNLLLYQEG AKMAVRLADG LQKMVPLLTK NNPKFLAITT DCLQLLAYGN QESKLIILAN GGPQALVQIM
321 RSYTYEKLLW TTSRVLKVLS VCPSNKPAIV EAGGMQALGK HLTSSSPRLV QNCLWTLRNL SDVATKQEGL DGVLKILVNQ
401 LSSDDIHVLT CATGTLSNLT CNNSKNKTLV TQSNGVEALI HTILRAGDKE DITEPAVCAL RHLTSRHPEA EMAQNSVR**LN**
481 **YGIPAIVK**LL NQPNQWPLIK ATIGLIRNLA LCPANHAPLQ EAAVIPRLVQ LLVKAHQDAQ RHAAAGTQQP YTDGVKMEEI
561 VEGCTGALHI LARDPMNRME IFRLNTIPLF VQLLYSPVEN IQRVAAGVLC ELAQDKEAAD AIDAEGASAP LMELLHSRNE
641 GTATYAAAVL FR**ISEDKNPD YR**KRVSVELT NSLFKHDPAA WEAAQSMIPI HEPYADELDG GYRGMYSGEI PMDAMDMDGD
721 YPMDTYSDGV RAPYPDHMLA

Supporting Peptides:

Peptide	Uniq	-10lgP	Mass	Length	ppm	m/z	z	RT	Fraction	Scan	Source File	Area F1	Area F2	Area F3	Area F4
R.LNYGIPAIVK.L	Y	54.39	1086.6437	10	0.2	544.3292	2	49.42	6	F6:14436	17112021_RID_2349_DP6.raw	5.5682E4			1.1942E5
R.TMQNTSDLDTAR.C	Y	50.31	1351.6038	12	0.9	676.8098	2	26.03	6	F6:6391	17112021_RID_2349_DP6.raw				
R.ISEDKNPDYR.K	Y	46.51	1235.5781	10	1.3	618.7971	2	18.97	6	F6:3975	17112021_RID_2349_DP6.raw				

total 3 peptides

XP_026525655.1
back to list

| Protein Coverage | Supporting Peptides |

Protein Coverage:

1 MEVMNIIDQP IKVTEWQQTY TYDSGIHSGM NTQVPSVSSK GLMDEDDLYG KQYTIKKTTT YSQPGSGQAQ TQAELESQLA
81 MTRAQRIRAA MYPETAEDHT LLLTTQVEGQ QTNVQRLAEP SQMLKSAIVH LINYQDDAEL ATRAIPELTK LLNDEDPVVV
161 SKAAMIVNQL SKKEASRRAL MQSAQIVAAV VR**TMQNTSDL DTAR**CTTSIL HNLSHHREGL LSIFKSGGIP ALVRMLSSPV
241 ESVLFYAITT LHNLLLYQEG AKMAVRLADG LQKMVPLLTK NNPKFLAITT DCLQLLAYGN QESKLIILAN GGPQALVQIM
321 RSYTYEKLLW TTSRVLKVLS VCPSNKPAIV EAGGMQALGK HLTSSSPRLV QNCLWTLRNL SDVATKQEGL DGVLKILVNQ
401 LSSDDIHVLT CATGTLSNLT CNNSKNKTLV TQSNGVEALI HTILRAGDKE DITEPAVCAL RHLTSRHPEA EMAQNSVR**LN**
481 **YGIPAIVK**LL NQPNQWPLIK ATIGLIRNLA LCPANHAPLQ EAAVIPRLVQ LLVKAHQDAQ RHAAAGTQQP YTDGVKMEEI
561 VEGCTGALHI LARDPMNRME IFRLNTIPLF VQLLYSPVEN IQRVAAGVLC ELAQDKEAAD AIDAEGASAP LMELLHSRNE
641 GTATYAAAVL FR**ISEDKNPD YR**KRVSVELT NSLFKHDPAA WEAAQSMIPI HEPYADELDG GYRGMYSGEI PMDAMDMDGD
721 YPMDTYSDGV RAPYPDHMLA

Supporting Peptides:

Peptide	Uniq	-10lgP	Mass	Length	ppm	m/z	z	RT	Fraction	Scan	Source File	Area F1	Area F2	Area F3	Area F4
R.LNYGIPAIVK.L	Y	54.39	1086.6437	10	0.2	544.3292	2	49.42	6	F6:14436	17112021_RID_2349_DP6.raw	5.5682E4			1.1942E5
R.TMQNTSDLDTAR.C	Y	50.31	1351.6038	12	0.9	676.8098	2	26.03	6	F6:6391	17112021_RID_2349_DP6.raw				
R.ISEDKNPDYR.K	Y	46.51	1235.5781	10	1.3	618.7971	2	18.97	6	F6:3975	17112021_RID_2349_DP6.raw				

total 3 peptides

XP_026525654.1
back to list

| Protein Coverage | Supporting Peptides |
Protein Coverage:

```
  1 MEVMNIIDQP IKVTEWQQTY TYDSGIHSGM NTQVPSVSSK GLMDEDDLYG KQYTIKKTTT YSQPGSGQAQ TQAELESQLA
 81 MTRAQRIRAA MYPETAEDHT LLLTTQVEGQ QTNVQRLAEP SQMLKSAIVH LINYQDDAEL ATRAIPELTK LLNDEDPVVV
161 SKAAMIVNQL SKKEASRRAL MQSAQIVAAV VRTMQNTSDL DTARCTTSIL HNLSHHREGL LSIFKSGGIP ALVRMLSSPV
241 ESVLFYAITT LHNLLLYQEG AKMAVRLADG LQKMVPLLTK NNPKFLAITT DCLQLLAYGN QESKLIILAN GGPQALVQIM
321 RSYTYEKLLW TTSRVLKVLS VCPSNKPAIV EAGGMQALGK HLTSSSPRLV QNCLWTLRNL SDVATKQEGL DGVLKILVNQ
401 LSSDDIHVLT CATGTLSNLT CNNSKNKTLV TQSNGVEALI HTILRAGDKE DITEPAVCAL RHLTSRHPEA EMAQNSVRLN
481 YGIPAIVKLL NQPNQWPLIK ATIGLIRNLA LCPANHAPLQ EAAVIPRLVQ LLVKAHQDAQ RHAAAGTQQP YTDGVKMEEI
561 VEGCTGALHI LARDPMNRME IFRLNTIPLF VQLLYSPVEN IQRVAAGVLC ELAQDKEAAD AIDAEGASAP LMELLHSRNE
641 GTATYAAAVL FRISEDKNPD YRKRVSVELT NSLFKHDPAA WEAAQSMIPI HEPYADELDG GYRGMYSGEI PMDAMDMDGD
721 YPMDTYSDGV RAPYPDHMLA
```

Supporting Peptides:

Peptide	Uniq	-10lgP	Mass	Length	ppm	m/z	z	RT	Fraction	Scan	Source File	Area F1	Area F2	Area F3	Area F4
R.LNYGIPAIVK.L	Y	54.39	1086.6437	10	0.2	544.3292	2	49.42	6	F6:14436	17112021_RID_2349_DP6.raw	5.5682E4			1.1942E5
R.TMQNTSDLDTAR.C	Y	50.31	1351.6038	12	0.9	676.8098	2	26.03	6	F6:6391	17112021_RID_2349_DP6.raw				
R.ISEDKNPDYR.K	Y	46.51	1235.5781	10	1.3	618.7971	2	18.97	6	F6:3975	17112021_RID_2349_DP6.raw				

total 3 peptides

XP_026576298.1
back to list

| Protein Coverage | Supporting Peptides |
Protein Coverage:

```
  1 MEVMNIIDQP IKVTEWQQTY TYDSGIHSGM NTQVPSVSSK GLMDEDDLYG KQYTIKKTTT YSQPGSGQAQ TQAELESQLA
 81 MTRAQRIRAA MYPETAEDHT LLLTTQVEGQ QTNVQRLAEP SQMLKSAIVH LINYQDDAEL ATRAIPELTK LLNDEDPVVV
161 SKAAMIVNQL SKKEASRRAL MQSAQIVAAV VRTMQNTSDL DTARCTTSIL HNLSHHREGL LSIFKSGGIP ALVRMLSSPV
241 ESVLFYAITT LHNLLLYQEG AKMAVRLADG LQKMVPLLTK NNPKFLAITT DCLQLLAYGN QESKLIILAN GGPQALVQIM
321 RSYTYEKLLW TTSRVLKVLS VCPSNKPAIV EAGGMQALGK HLTSSSPRLV QNCLWTLRNL SDVATKQEGL DGVLKILVNQ
401 LSSDDIHVLT CATGTLSNLT CNNSKNKTLV TQSNGVEALI HTILRAGDKE DITEPAVCAL RHLTSRHPEA EMAQNSVRLN
481 YGIPAIVKLL NQPNQWPLIK ATIGLIRNLA LCPANHAPLQ EAAVIPRLVQ LLVKAHQDAQ RHAAAGTQQP YTDGVKMEEI
561 VEGCTGALHI LARDPMNRME IFRLNTIPLF VQLLYSPVEN IQRVAAGVLC ELAQDKEAAD AIDAEGASAP LMELLHSRNE
641 GTATYAAAVL FRISEDKNPD YRKRVSVELT NSLFKHDPAA WEAAQSMIPI HEPYADELDG GYRGMYSGEI PMDAMDMDGD
721 YPMDTYSDGV RAPYPDHMLA
```

Supporting Peptides:

Peptide	Uniq	-10lgP	Mass	Length	ppm	m/z	z	RT	Fraction	Scan	Source File	Area F1	Area F2	Area F3	Area F4
R.LNYGIPAIVK.L	Y	54.39	1086.6437	10	0.2	544.3292	2	49.42	6	F6:14436	17112021_RID_2349_DP6.raw	5.5682E4			1.1942E5
R.TMQNTSDLDTAR.C	Y	50.31	1351.6038	12	0.9	676.8098	2	26.03	6	F6:6391	17112021_RID_2349_DP6.raw				
R.ISEDKNPDYR.K	Y	46.51	1235.5781	10	1.3	618.7971	2	18.97	6	F6:3975	17112021_RID_2349_DP6.raw				

total 3 peptides

XP_013919280.1
back to list

| Protein Coverage | Supporting Peptides |
Protein Coverage:

```
  1 MDMMNIIDQP IKVTEWQQTY TYDSGIHSGM NTQVPSVSSK GLMDEDDLYG KQYTIKKTTT YSQPGSGQAQ TQAELESQIA
 81 MTRAQRIRAA MYPETAEDHT LLLTTQVEGQ QTNVQRLAEP SQMLKSAIVH LINYQDDAEL ATRAIPELTK LLNDEDPVVV
161 SKAAMIVNQL SKKEASRRAL MQSAQIVAAV VRTMQNTSDL DTARCTTSIL HNLSHHREGL LSIFKSGGIP ALVRMLSSPV
241 ESVLFYAITT LHNLLLYQEG AKMAVRLADG LQKMVPLLTK NNPKFLAITT DCLQLLAYGN QESKLIILAN GGPQALVQIM
321 RSYTYEKLLW TTSRVLKVLS VCPSNKPAIV EAGGMQALGK HLTSSSPRLV QNCLWTLRNL SDVATKQEGL DGVLKILVNQ
401 LSSDDINVLT CATGTLSNLT CNNSKNKTLV TQSNGVEALI HTILRAGDKE DITEPAVCAL RHLTSRHPEA EMAQNSVRLN
481 YGIPAIVKLL NQPNQWPLIK ATIGLIRNLA LCPANHAPLQ EAAVIPRLVQ LLVKAHQDAQ RHAAAGTQQP YTDGVKMEEI
561 VEGCTGALHI LARDPMNRME IFRLNTIPLF VQLLYSPVEN IQRVAAGVLC ELAQDKEAAD AIDAEGASAP LMELLHSRNE
641 GTATYAAAVL FRISEDKNPD YRKRVSVELT NSLFKHDPAA WEAAQSMIPI HEPYADELDG GYRAMYSGEI PMDHMDMHMD
721 MDGDYPMDTY SDGVRAPYPD HMLA
```

Supporting Peptides:

Peptide	Uniq	-10lgP	Mass	Length	ppm	m/z	z	RT	Fraction	Scan	Source File	Area F1	Area F2	Area F3	Area F4
R.LNYGIPAIVK.L	Y	54.39	1086.6437	10	0.2	544.3292	2	49.42	6	F6:14436	17112021_RID_2349_DP6.raw	5.5682E4			1.1942E5
R.TMQNTSDLDTAR.C	Y	50.31	1351.6038	12	0.9	676.8098	2	26.03	6	F6:6391	17112021_RID_2349_DP6.raw				
R.ISEDKNPDYR.K	Y	46.51	1235.5781	10	1.3	618.7971	2	18.97	6	F6:3975	17112021_RID_2349_DP6.raw				

total 3 peptides

TRINITY_DN206
back to list

| Protein Coverage | Supporting Peptides |
Protein Coverage:

```
  1 PLFAGTSHLE RGGVERGKQG GGDGAGRIAM DMMNIIDQPI KVTEWQQTYT YDSGIHSGMN TQVPSVSSKG LMDEDDLYGK
 81 QYTIKKTTTY SQPGSGQAQS QAELESQLAM TRAQRIRAAM YPETAEDHTL LLTTQVEGQQ TNVQRLAEPS QMLKSAIVHL
161 INYQDDAELA TRAIPELTKL LNDEDPVVVS KAAMIVNQLS KKEASRRALM QSAQIVAAVV RTMQNTSDLD TARCTTSILH
241 NLSHHREGLL SIFKSGGIPA LVRMLSSPVE SVLFYAITTL HNLLLYQEGA KMAVRLADGL QKMVPLLTKN NPKFLAITTD
321 CLQLLAYGNQ ESKLIILANG GPQALVQIMR SYTYEKLLWT TSRVLKVLSV CPSNKPAIVE AGGMQALGKH LTSSSPRLVQ
401 NCLWTLRNLS DVATKQEGLD GVLKILVNQL SSDDINVLTC ATGTLSNLTC NNSKNKTLVT QSNGVEALIH TILRAGDKED
481 ITEPAVCALR HLTSRHPEAE MAQNSVRLNY GIPAIVKLLN QPNQWPLIKA TIGLIRNLAL CPANHVPLQE AAVIPRLVQL
561 LVKAHQDAQR HAAAGTQQPY TDGVKMEEIV EGCTGALHIL ARDPMNRMEI FRLNTIPLFV QLLYSPVENI QRVAAGVLCE
641 LAQDKEAADA IDAEGASAPL MELLHSRNEG TATYAAAVLF RISEDKNPDY RKRVSVELTN SLFKHDPAAW EAAQSMIPIH
721 EPYADDVDAG YRGMYSGEIP MDPMDMHLDM DGDYPMDTYS DGVRAPYPGH MLA
```

Supporting Peptides:

Peptide	Uniq	-10lgP	Mass	Length	ppm	m/z	z	RT	Fraction	Scan	Source File	Area F1	Area F2	Area F3	Area F4
R.LNYGIPAIVK.L	Y	54.39	1086.6437	10	0.2	544.3292	2	49.42	6	F6:14436	17112021_RID_2349_DP6.raw	5.5682E4			1.1942E5
R.TMQNTSDLDTAR.C	Y	50.31	1351.6038	12	0.9	676.8098	2	26.03	6	F6:6391	17112021_RID_2349_DP6.raw				
R.ISEDKNPDYR.K	Y	46.51	1235.5781	10	1.3	618.7971	2	18.97	6	F6:3975	17112021_RID_2349_DP6.raw				

total 3 peptides

TRINITY_DN206
back to list

| Protein Coverage | Supporting Peptides |
Protein Coverage:

```
  1 PLFAGTSHLE RGGVERGKQG GGDGAGRIAM DMMNIIDQPI KVTEWQQTYT YDSGIHSGMN TQVPSVSSKG LMDEDDLYGK
 81 QYTIKKTTTY SQPGSGQAQS QAELESQLAM TRAQRIRAAM YPETAEDHTL LLTTQVEGQQ TNVQRLAEPS QMLKSAIVHL
161 INYQDDAELA TRAIPELTKL LNDEDPVVVS KAAMIVNQLS KKEASRRALM QSAQIVAAVV RTMQNTSDLD TARCTTSILH
241 NLSHHREGLL SIFKSGGIPA LVRMLSSPVE SVLFYAITTL HNLLLYQEGA KMAVRLADGL QKMVPLLTKN NPKFLAITTD
321 CLQLLAYGNQ ESKLIILANG GPQALVQIMR SYTYEKLLWT TSRVLKVLSV CPSNKPAIVE AGGMQALGKH LTSSSPRLVQ
401 NCLWTLRNLS DVATKQEGLD GVLKILVNQL SSDDINVLTC ATGTLSNLTC NNSKNKTLVT QSNGVEALIH TILRAGDKED
481 ITEPAVCALR HLTSRHPEAE MAQNSVRLNY GIPAIVKLLN QPNQWPLIKA TIGLIRNLAL CPANHVPLQE AAVIPRLVQL
561 LVKAHQDAQR HAAAGTQQPY TDGVKMEEIV EGCTGALHIL ARDPMNRMEI FRLNTIPLFV QLLYSPVENI QRVAAGVLCE
641 LAQDKEAADA IDAEGASAPL MELLHSRNEG TATYAAAVLF RISEDKNPDY RKRVSVELTN SLFKHDPAAW EAAQSMIPIH
721 EPYADDVDAG YRGMYSGEIP MDPMDMHLDM DGDYPMDTYS DGVRAPYPGH MLA
```

Supporting Peptides:

Peptide	Uniq	-10lgP	Mass	Length	ppm	m/z	z	RT	Fraction	Scan	Source File	Area F1	Area F2	Area F3	Area F4
R.LNYGIPAIVK.L	Y	54.39	1086.6437	10	0.2	544.3292	2	49.42	6	F6:14436	17112021_RID_2349_DP6.raw	5.5682E4			1.1942E5
R.TMQNTSDLDTAR.C	Y	50.31	1351.6038	12	0.9	676.8098	2	26.03	6	F6:6391	17112021_RID_2349_DP6.raw				
R.ISEDKNPDYR.K	Y	46.51	1235.5781	10	1.3	618.7971	2	18.97	6	F6:3975	17112021_RID_2349_DP6.raw				

total 3 peptides

XP_032091066.1
back to list

| Protein Coverage | Supporting Peptides |
Protein Coverage:

1 MGTRDGLCQA RCSPPLFAES SHLERGGVER SNQGGGDGAS RIAMDMMNII DQPIKVTEWQ QTYTYDSGIH SGMNTQVPSV
81 SSKGLMDEDD LYGKQYTIKK TTTYSQPGSG QAQTQAELES QLAMTRAQRI RAAMYPETAE DHTLLLTTQV EGQQTNVQRL
161 AEPSQMLKSA IVHLINYQDD AELATRAIPE LTKLLNDEDP VVVSKAAMIV NQLSKKDASR RALMQSAQIV AAVVR**TMQNT**
241 **SDLDTAR**CTT SILHNLSHHR EGLLSIFKSG GIPALVRMLS SPVESVLFYA ITTLHNLLLY QEGAKMAVRL ADGLQKMVPL
321 LTKNNPKFLA ITTDCLQLLA YGNQESKLII LANGGPQALV QIMRSYTYEK LLWTTSRVLK VLSVCPSNKP AIVEAGGMQA
401 LGKHLTSSSP RLVQNCLWTL RNLSDVATKQ EGLDGVLKIL VNQLSSDDIN VLTCATGTLS NLTCNNSKNK TLVTQSNGVE
481 ALIHTILRAG DKEDITEPAV CALRHLTSRH PEAEMAQNSV R**LNYGIPAIV K**LLNQPNQWP LIKATIGLIR NLALCPANHA
561 PLQEAAVIPR LVQLLVKAHQ DAQRHAAAGT QQPYTDGVKM EEIVEGCTGA LHILARDPMN RMEIFRLNTI PLFVQLLYSP
641 VENIQRVAAG VLCELAQDKE AADAIDAEGA SAPLMELLHS RNEGTATYAA AVLFR**ISEDK NPDYR**KRVSV ELTNSLFKHD
721 PAAWEAAQSM IPIHEPYADE LDGGYRAMYS GEIPMDHMDM HMDMDGDYPM DTYSDGVRAP YPDHMLA

Supporting Peptides:

Peptide	Uniq	-10lgP	Mass	Length	ppm	m/z	z	RT	Fraction	Scan	Source File	Area F1	Area F2	Area F3	Area F4
R.LNYGIPAIVK.L	Y	54.39	1086.6437	10	0.2	544.3292	2	49.42	6	F6:14436	17112021_RID_2349_DP6.raw	5.5682E4			1.1942E5
R.TMQNTSDLDTAR.C	Y	50.31	1351.6038	12	0.9	676.8098	2	26.03	6	F6:6391	17112021_RID_2349_DP6.raw				
R.ISEDKNPDYR.K	Y	46.51	1235.5781	10	1.3	618.7971	2	18.97	6	F6:3975	17112021_RID_2349_DP6.raw				

total 3 peptides

XP_032091065.1
back to list

| Protein Coverage | Supporting Peptides |
Protein Coverage:

1 MGTRDGLCQA RCSPPLFAES SHLERGGVER SNQGGGDGAS RIAMDMMNII DQPIKVTEWQ QTYTYDSGIH SGMNTQVPSV
81 SSKGLMDEDD LYGKQYTIKK TTTYSQPGSG QAQTQAELES QLAMTRAQRI RAAMYPETAE DHTLLLTTQV EGQQTNVQRL
161 AEPSQMLKSA IVHLINYQDD AELATRAIPE LTKLLNDEDP VVVSKAAMIV NQLSKKDASR RALMQSAQIV AAVVR**TMQNT**
241 **SDLDTAR**CTT SILHNLSHHR EGLLSIFKSG GIPALVRMLS SPVESVLFYA ITTLHNLLLY QEGAKMAVRL ADGLQKMVPL
321 LTKNNPKFLA ITTDCLQLLA YGNQESKLII LANGGPQALV QIMRSYTYEK LLWTTSRVLK VLSVCPSNKP AIVEAGGMQA
401 LGKHLTSSSP RLVQNCLWTL RNLSDVATKQ EGLDGVLKIL VNQLSSDDIN VLTCATGTLS NLTCNNSKNK TLVTQSNGVE
481 ALIHTILRAG DKEDITEPAV CALRHLTSRH PEAEMAQNSV R**LNYGIPAIV K**LLNQPNQWP LIKATIGLIR NLALCPANHA
561 PLQEAAVIPR LVQLLVKAHQ DAQRHAAAGT QQPYTDGVKM EEIVEGCTGA LHILARDPMN RMEIFRLNTI PLFVQLLYSP
641 VENIQRVAAG VLCELAQDKE AADAIDAEGA SAPLMELLHS RNEGTATYAA AVLFR**ISEDK NPDYR**KRVSV ELTNSLFKHD
721 PAAWEAAQSM IPIHEPYADE LDGGYRAMYS GEIPMDHMDM HMDMDGDYPM DTYSDGVRAP YPDHMLA

Supporting Peptides:

Peptide	Uniq	-10lgP	Mass	Length	ppm	m/z	z	RT	Fraction	Scan	Source File	Area F1	Area F2	Area F3	Area F4
R.LNYGIPAIVK.L	Y	54.39	1086.6437	10	0.2	544.3292	2	49.42	6	F6:14436	17112021_RID_2349_DP6.raw	5.5682E4			1.1942E5
R.TMQNTSDLDTAR.C	Y	50.31	1351.6038	12	0.9	676.8098	2	26.03	6	F6:6391	17112021_RID_2349_DP6.raw				
R.ISEDKNPDYR.K	Y	46.51	1235.5781	10	1.3	618.7971	2	18.97	6	F6:3975	17112021_RID_2349_DP6.raw				

total 3 peptides

XP_034271617.1
back to list

| Protein Coverage | Supporting Peptides |
Protein Coverage:

1 MDMMNIIDQP IKVTEWQQTY TYDSGIHSGM NTQVPSVSSK GLMDEDDLYG KQYTIKKTTT YSQPGSGQAQ TQELESQLAM
81 TRAQRIRAAM YPETTEDHTL LLTTQVEGQQ TNVQRLAEPS QMLKSAIVHL INYQDDAELA TRAIPELTKL LNDEDPVVVS
161 KAAMIVNQLS KKEASRRALM QSAQIVAAVV R**TMQNTSDLD TAR**CTTSILH NLSHHREGLL SIFKSGGIPA LVRMLSSPVE
241 SVLFYAITTL HNLLLYQEGA KMAVRLADGL QKMVPLLTKN NPKFLAITTD CLQLLAYGNQ ESKLIILANG GPQALVQIMR
321 SYTYEKLLWT TSRVLKVLSV CPSNKPAIVE AGGMQALGKH LTSSSPRLVQ NCLWTLRNLS DVATKQEGLD GVLKILVNQL
401 SSDDINVLTC ATGTLSNLTC NNSKNKTLVT QSNGVEALIH TILRAGDKED ITEPAVCALR HLTSRHPEAE MAQNSVR**LNY**
481 **GIPAIVK**LLN QPNQWPLIKA TIGLIRNLAL CPANHAPLQE AAVIPRLVQL LVKAHQDAQR HAAAGTQQPY TDGVKMEEIV
561 EGCTGALHIL ARDPMNRMEI FRLNTIPLFV QLLYSPVENI QRVAAGVLCE LAQDKEAADA IDAEGASAPL MELLHSRNEG
641 TATYAAAVLF R**ISEDKNPDY R**KRVSVELTN SLFKHDPAAW EAAQSMIPIH EPYADELDGG YRGMYSGEIP MDPMDMHMDM
721 DGDYPMDTYS DGVRAPYPDH MLA

Supporting Peptides:

Peptide	Uniq	-10lgP	Mass	Length	ppm	m/z	z	RT	Fraction	Scan	Source File	Area F1	Area F2	Area F3	Area F4
R.LNYGIPAIVK.L	Y	54.39	1086.6437	10	0.2	544.3292	2	49.42	6	F6:14436	17112021_RID_2349_DP6.raw	5.5682E4			1.1942E5
R.TMQNTSDLDTAR.C	Y	50.31	1351.6038	12	0.9	676.8098	2	26.03	6	F6:6391	17112021_RID_2349_DP6.raw				
R.ISEDKNPDYR.K	Y	46.51	1235.5781	10	1.3	618.7971	2	18.97	6	F6:3975	17112021_RID_2349_DP6.raw				

total 3 peptides

XP_034271615.1
back to list

| Protein Coverage | Supporting Peptides |
Protein Coverage:

1 MDMMNIIDQP IKVTEWQQTY TYDSGIHSGM NTQVPSVSSK GLMDEDDLYG KQYTIKKTTT YSQPGSGQAQ TQAELESQLA
81 MTRAQRIRAA MYPETTEDHT LLLTTQVEGQ QTNVQRLAEP SQMLKSAIVH LINYQDDAEL ATRAIPELTK LLNDEDPVVV
161 SKAAMIVNQL SKKEASRRAL MQSAQIVAAV VR**TMQNTSDL DTAR**CTTSIL HNLSHHREGL LSIFKSGGIP ALVRMLSSPV
241 ESVLFYAITT LHNLLLYQEG AKMAVRLADG LQKMVPLLTK NNPKFLAITT DCLQLLAYGN QESKLIILAN GGPQALVQIM
321 RSYTYEKLLW TTSRVLKVLS VCPSNKPAIV EAGGMQALGK HLTSSSPRLV QNCLWTLRNL SDVATKQEGL DGVLKILVNQ
401 LSSDDINVLT CATGTLSNLT CNNSKNKTLV TQSNGVEALI HTILRAGDKE DITEPAVCAL RHLTSRHPEA EMAQNSVR**LN**
481 **YGIPAIVK**LL NQPNQWPLIK ATIGLIRNLA LCPANHAPLQ EAAVIPRLVQ LLVKAHQDAQ RHAAAGTQQP YTDGVKMEEI
561 VEGCTGALHI LARDPMNRME IFRLNTIPLF VQLLYSPVEN IQRVAAGVLC ELAQDKEAAD AIDAEGASAP LMELLHSRNE
641 GTATYAAAVL FR**ISEDKNPD YR**KRVSVELT NSLFKHDPAA WEAAQSMIPI HEPYADELDG GYRGMYSGEI PMDPMDMHMD
721 MDGDYPMDTY SDGVRAPYPD HMLA

Supporting Peptides:

Peptide	Uniq	-10lgP	Mass	Length	ppm	m/z	z	RT	Fraction	Scan	Source File	Area F1	Area F2	Area F3	Area F4
R.LNYGIPAIVK.L	Y	54.39	1086.6437	10	0.2	544.3292	2	49.42	6	F6:14436	17112021_RID_2349_DP6.raw	5.5682E4			1.1942E5
R.TMQNTSDLDTAR.C	Y	50.31	1351.6038	12	0.9	676.8098	2	26.03	6	F6:6391	17112021_RID_2349_DP6.raw				
R.ISEDKNPDYR.K	Y	46.51	1235.5781	10	1.3	618.7971	2	18.97	6	F6:3975	17112021_RID_2349_DP6.raw				

total 3 peptides

XP_034271618.1
back to list

| Protein Coverage | Supporting Peptides |
Protein Coverage:

1 MDMMNIIDQP IKVTEWQQTY TYDSGIHSGM NTQVPSVSSK GLMDEDDLYG KQYTIKKTTT YSQPGSGQAQ TQAELESQLA
81 MTRAQRIRAA MYPETTEDHT LLLTTQVEGQ QTNVQRLAEP SQMLKSAIVH LINYQDDAEL ATRAIPELTK LLNDEDPVVV
161 SKAAMIVNQL SKKEASRRAL MQSAQIVAAV VR**TMQNTSDL DTAR**CTTSIL HNLSHHREGL LSIFKSGGIP ALVRMLSSPV
241 ESVLFYAITT LHNLLLYQEG AKMAVRLADG LQKMVPLLTK NNPKFLAITT DCLQLLAYGN QESKLIILAN GGPQALVQIM
321 RSYTYEKLLW TTSRVLKVLS VCPSNKPAIV EAGGMQALGK HLTSSSPRLV QNCLWTLRNL SDVATKQEGL DGVLKILVNQ
401 LSSDDINVLT CATGTLSNLT CNNSKNKTLV TQSNGVEALI HTILRAGDKE DITEPAVCAL RHLTSRHPEA EMAQNSVR**LN**
481 **YGIPAIVK**LL NQPNQWPLIK ATIGLIRNLA LCPANHAPLQ EAAVIPRLVQ LLVKAHQDAQ RHAAAGTQQP YTDGVKMEEI
561 VEGCTGALHI LARDPMNRME IFRLNTIPLF VQLLYSPVEN IQRVAAGVLC ELAQDKEAAD AIDAEGASAP LMELLHSRNE
641 GTATYAAAVL FR**ISEDKNPD YR**KRVSVELT NSLFKHDPAA WEAAQSMIPI HEPYADELDG GYRGMYSGEI PMDPMDMHMD
721 MDGDYPMDTY SDGVRAPYPD HMLA

Supporting Peptides:

Peptide	Uniq	-10lgP	Mass	Length	ppm	m/z	z	RT	Fraction	Scan	Source File	Area F1	Area F2	Area F3	Area F4
R.LNYGIPAIVK.L	Y	54.39	1086.6437	10	0.2	544.3292	2	49.42	6	F6:14436	17112021_RID_2349_DP6.raw	5.5682E4			1.1942E5
R.TMQNTSDLDTAR.C	Y	50.31	1351.6038	12	0.9	676.8098	2	26.03	6	F6:6391	17112021_RID_2349_DP6.raw				
R.ISEDKNPDYR.K	Y	46.51	1235.5781	10	1.3	618.7971	2	18.97	6	F6:3975	17112021_RID_2349_DP6.raw				

total 3 peptides

XP_034271616.1
back to list

| Protein Coverage | Supporting Peptides |
Protein Coverage:

```
  1 MDMMNIIDQP IKVTEWQQTY TYDSGIHSGM NTQVPSVSSK GLMDEDDLYG KQYTIKKTTT YSQPGSGQAQ TQAELESQLA
 81 MTRAQRIRAA MYPETTEDHT LLLTTQVEGQ QTNVQRLAEP SQMLKSAIVH LINYQDDAEL ATRAIPELTK LLNDEDPVVV
161 SKAAMIVNQL SKKEASRRAL MQSAQIVAAV VRTMQNTSDL DTARCTTSIL HNLSHHREGL LSIFKSGGIP ALVRMLSSPV
241 ESVLFYAITT LHNLLLYQEG AKMAVRLADG LQKMVPLLTK NNPKFLAITT DCLQLLAYGN QESKLIILAN GGPQALVQIM
321 RSYTYEKLLW TTSRVLKVLS VCPSNKPAIV EAGGMQALGK HLTSSSPRLV QNCLWTLRNL SDVATKQEGL DGVLKILVNQ
401 LSSDDINVLT CATGTLSNLT CNNSKNKTLV TQSNGVEALI HTILRAGDKE DITEPAVCAL RHLTSRHPEA EMAQNSVRLN
481 YGIPAIVKLL NQPNQWPLIK ATIGLIRNLA LCPANHAPLQ EAAVIPRLVQ LLVKAHQDAQ RHAAAGTQQP YTDGVKMEEI
561 VEGCTGALHI LARDPMNRME IFRLNTIPLF VQLLYSPVEN IQRVAAGVLC ELAQDKEAAD AIDAEGASAP LMELLHSRNE
641 GTATYAAAVL FRISEDKNPD YRKRVSVELT NSLFKHDPAA WEAAQSMIPI HEPYADELDG GYRGMYSGEI PMDPMDMHMD
721 MDGDYPMDTY SDGVRAPYPD HMLA
```

Supporting Peptides:

Peptide	Uniq	-10lgP	Mass	Length	ppm	m/z	z	RT	Fraction	Scan	Source File	Area F1	Area F2	Area F3	Area F4
R.LNYGIPAIVK.L	Y	54.39	1086.6437	10	0.2	544.3292	2	49.42	6	F6:14436	17112021_RID_2349_DP6.raw	5.5682E4			1.1942E5
R.TMQNTSDLDTAR.C	Y	50.31	1351.6038	12	0.9	676.8098	2	26.03	6	F6:6391	17112021_RID_2349_DP6.raw				
R.ISEDKNPDYR.K	Y	46.51	1235.5781	10	1.3	618.7971	2	18.97	6	F6:3975	17112021_RID_2349_DP6.raw				

total 3 peptides

XP_025022317.1
back to list

| Protein Coverage | Supporting Peptides |
Protein Coverage:

```
  1 MDVMNLIEQP IKVTEWQQTY TYDSGIHSGM NTQVPSVSSK GLMDEDDLYG KQYTIKKTTT YSQAGPGQAQ AQAQAELESQ
 81 LAMTRAQRIR AAMYPETVED HSLLVTTQVE GQQTNVQRLA EPSQMLKSAI VHLINYQDDA ELATRAIPEL TKLLNDEDPV
161 VVSKAAMIVN QLSKKEASRR ALMQSAQIVA AVVRTMQNTS DLDTARCTTS ILHNLSHHRE GLLSIFKSGG IPALVRMLSS
241 PVESVLFYAI TTLHNLLLYQ EGAKMAVRLA DGLQKMVPLL TKNNPKFLAI TTDCLQLLAY GNQESKLIIL ANGGPQALVQ
321 IMRSYTYEKL LWTTSRVLKV LSVCPSNKPA IVEAGGMQAL GKHLTSSSPR LVQNCLWTLR NLSDVATKQE GLDGVLKILV
401 NQLSSDDINV LTCATGTLSN LTCNNSKNKT LVTQSNGVEA LIHTILRAGD KEDITEPAVC ALRHLTSRHP EAEMAQNSVR
481 LNYGIPAIVK LLNQPNQWPL IKATIGLIRN LALCPANHAP LQEAAVIPRL VQLLVKAHQD AQRHAAAGTQ QPYTDGVKME
561 EIVEGCTGAL HILARDPMNR MEIFRLNTIP LFVQLLYSPV ENIQRVAAGV LCELAQDKEA ADAIDAEGAS APLMELLHSR
641 NEGTATYAAA VLFRISEDKN PDYRKRVSVE LTNSLFKHDP AAWEAAQSMI PIHEPYADEL DGGYRGMYPG EIPMDPMDMH
721 MDMDGDYPMD TYSDGVRAPY PDHMLA
```

Supporting Peptides:

Peptide	Uniq	-10lgP	Mass	Length	ppm	m/z	z	RT	Fraction	Scan	Source File	Area F1	Area F2	Area F3	Area F4
R.LNYGIPAIVK.L	Y	54.39	1086.6437	10	0.2	544.3292	2	49.42	6	F6:14436	17112021_RID_2349_DP6.raw	5.5682E4			1.1942E5
R.TMQNTSDLDTAR.C	Y	50.31	1351.6038	12	0.9	676.8098	2	26.03	6	F6:6391	17112021_RID_2349_DP6.raw				
R.ISEDKNPDYR.K	Y	46.51	1235.5781	10	1.3	618.7971	2	18.97	6	F6:3975	17112021_RID_2349_DP6.raw				

total 3 peptides

XP_007428111.1
back to list

| Protein Coverage | Supporting Peptides |
Protein Coverage:

1 MDVMNLIEQP IKVTEWQQTY TYDSGIHSGM NTQVPSVSSK GLMDEDDLYG KQYTIKKTTT YSQAGPGQAQ AQAQAELESQ
81 LAMTRAQRIR AAMYPETVED HSLLVTTQVE GQQTNVQRLA EPSQMLKSAI VHLINYQDDA ELATRAIPEL TKLLNDEDPV
161 VVSKAAMIVN QLSKKEASRR ALMQSAQIVA AVVR**TMQNTS DLDTAR**CTTS ILHNLSHHRE GLLSIFKSGG IPALVRMLSS
241 PVESVLFYAI TTLHNLLLYQ EGAKMAVRLA DGLQKMVPLL TKNNPKFLAI TTDCLQLLAY GNQESKLIIL ANGGPQALVQ
321 IMRSYTYEKL LWTTSRVLKV LSVCPSNKPA IVEAGGMQAL GKHLTSSSPR LVQNCLWTLR NLSDVATKQE GLDGVLKILV
401 NQLSSDDINV LTCATGTLSN LTCNNSKNKT LVTQSNGVEA LIHTILRAGD KEDITEPAVC ALRHLTSRHP EAEMAQNSVR
481 **LNYGIPAIVK** LLNQPNQWPL IKATIGLIRN LALCPANHAP LQEAAVIPRL VQLLVKAHQD AQRHAAAGTQ QPYTDGVKME
561 EIVEGCTGAL HILARDPMNR MEIFRLNTIP LFVQLLYSPV ENIQRVAAGV LCELAQDKEA ADAIDAEGAS APLMELLHSR
641 NEGTATYAAA VLFR**ISEDKN PDYR**KRVSVE LTNSLFKHDP AAWEAAQSMI PIHEPYADEL DGGYRGMYPG EIPMDPMDMH
721 MDMDGDYPMD TYSDGVRAPY PDHMLA

Supporting Peptides:

Peptide	Uniq	-10lgP	Mass	Length	ppm	m/z	z	RT	Fraction	Scan	Source File	Area F1	Area F2	Area F3	Area F4
R.LNYGIPAIVK.L	Y	54.39	1086.6437	10	0.2	544.3292	2	49.42	6	F6:14436	17112021_RID_2349_DP6.raw	5.5682E4			1.1942E5
R.TMQNTSDLDTAR.C	Y	50.31	1351.6038	12	0.9	676.8098	2	26.03	6	F6:6391	17112021_RID_2349_DP6.raw				
R.ISEDKNPDYR.K	Y	46.51	1235.5781	10	1.3	618.7971	2	18.97	6	F6:3975	17112021_RID_2349_DP6.raw				

total 3 peptides

TRINITY_DN459
back to list

| Protein Coverage | Supporting Peptides |
Protein Coverage:

1 QSADCPIDRY HRNGQPCLQN YGYCYNGKCP IMHHQCYYLF GANSTKGQDG CFDWNKKGTG DFYCRKENDV PIPCAPEDVK
81 CGRLFCEILP NTCK**HAPEDN GMVDPGTK**CE DKKVCMNRKC VDVNTAY

Supporting Peptides:

Peptide	Uniq	-10lgP	Mass	Length	ppm	m/z	z	RT	Fraction	Scan	Source File	Area F1	Area F2
K.HAPEDNGMVDPGTK.C	Y	76.97	1466.6460	14	-0.6	734.3298	2	23.50	4	F4:5677	17112021_RID_2349_DP4.raw	1.8448E6	3.4934E5
K.HAPEDNGM(+15.99)VDPGTK.C	Y	63.27	1482.6409	14	0.1	742.3278	2	23.52	4	F4:5529	17112021_RID_2349_DP4.raw	1.8341E6	

total

TRINITY_DN459
back to list

| Protein Coverage | Supporting Peptides |
Protein Coverage:

1 CEQCRFKRAG TECRRASECD LPEYCTGQSA ECPTDVFHSN GKPCLNNFGY CYNGKCPIMY HQCYALFGAN ATVGQDGCFE
81 WNKKGESYFY CRKENGVPIP CAPEDGYVKC GRLFCEILPN TCK**HAPEDNG MVDPGTK**CED KKVCMNRKCV DVNTAY

Supporting Peptides:

Peptide	Uniq	-10lgP	Mass	Length	ppm	m/z	z	RT	Fraction	Scan	Source File	Area F1	Area F2
K.HAPEDNGMVDPGTK.C	Y	76.97	1466.6460	14	-0.6	734.3298	2	23.50	4	F4:5677	17112021_RID_2349_DP4.raw	1.8448E6	3.4934E5
K.HAPEDNGM(+15.99)VDPGTK.C	Y	63.27	1482.6409	14	0.1	742.3278	2	23.52	4	F4:5529	17112021_RID_2349_DP4.raw	1.8341E6	

total

TRINITY_DN3041
back to list

| Protein Coverage | Supporting Peptides |
Protein Coverage:

1 RLFCEILPNT CK**HAPEDNGM VDPGTK**CEDK KVCMNRKCVD VNTAY

Supporting Peptides:

Peptide	Uniq	-10lgP	Mass	Length	ppm	m/z	z	RT	Fraction	Scan	Source File	Area F1	Area F2
K.HAPEDNGMVDPGTK.C	Y	76.97	1466.6460	14	-0.6	734.3298	2	23.50	4	F4:5677	17112021_RID_2349_DP4.raw	1.8448E6	3.4934E5
K.HAPEDNGM(+15.99)VDPGTK.C	Y	63.27	1482.6409	14	0.1	742.3278	2	23.52	4	F4:5529	17112021_RID_2349_DP4.raw	1.8341E6	

total

TRINITY_DN2

back to list

| Protein Coverage | Supporting Peptides |

Protein Coverage:

```
  1 RAASHMKERL PRVGITCRRS LFNPRSNWIW SAGRTMRCWV FLAVVAATLL AVRARDPFAE DCRIHEYPPA GPTYKGTIPE
 81 YVINLDLPPS ERWVQVIHDK KNELKAVIKT VRDIVLSFIH EKFVASMESK IGWLCSTLPY PFNEEIKGIA SAIDGPLGDV
161 VLFNIFYEIF TVCTSIIAED KTGKLYHARN LDFGLFLGWD IQNNSWDTTQ KLKPLMVSLD FQKNNKTVFK SANFAGYVGI
241 VSGVKQGLFT LTMNERFSLD GGYIGIFEWL IGKRDGWWMS FLTRAVLENS TSYEDAKDKL VNTKLLAPAY FIVGGNKLDE
321 GCIITRSRTA TLNIKNLDAK NGNWFVIETN YDPWKAPLII DDRRHPAMNC LNQTGQEKIA LPGLYNVLST KPILNKLTVS
401 TTLLEVSEGH IETYLRDCPD PCSPW
```

Supporting Peptides:

Peptide	Uniq	-10lgP	Mass	Length	ppm	m/z	z	RT	Fraction	Scan	Source File	Area F1	Area F2	Area F3	Area F4
K.LLAPAYFIVGGNK.L	Y	56.67	1361.7706	13	0.3	681.8928	2	57.51	4	F4:17066	17112021_RID_2349_DP4.raw				3.8785E:
R.DGWWMSFLTR.A	Y	52.96	1297.5913	10	3.2	649.8050	2	74.68	3	F3:22606	17112021_RID_2349_DP3.raw			7.0205E4	

total 2 peptides

TRINITY_DN7999

back to list

| Protein Coverage | Supporting Peptides |

Protein Coverage:

```
  1 KSQMYLQMNH LRSGDTTVYH CSGDGNYYAF DVWGPGTVVT VTNDPPKAPS VFPLTPGHET QVAKALSTAS NVTIGCLIKN
 81 YFPEPATVQW NSGAITSGIQ NFPPVLLASN HYSHTSLLTI PASQWQSESF QCNVEHAATN TRINKIIERS AIRSPVGPEI
161 TVFHSCDSRD SDGTVQLWCH ISGFYPKQLD IDWKVGSRSG QLHPYNYPPW QNPGTYTFST ASVVNITQDD WLEGNSYYCE
241 ATHAASQTKV KGKAKKCKDG SSCPSGDIKV DILPPRPKAL YIDRNSKISC VVSNLQNEQG LKFTWFREKS GHLTPDAIET
321 TEGSNGGYIV LSRLGIFSED WDSGETFTCV VEHPTFVSPV TKKISKSRGK HSAPKVYVFP PHQEELNPGR SVSLTCLVTG
401 FSPEAADVQW LEDHNPLSED NSVTTPPMKN KHDDSYFLYS KLLVPKTDWN TKSFTCMVVH EDLPMKFTQR NISRTQGKK
```

Supporting Peptides:

Peptide	Uniq	-10lgP	Mass	Length	ppm	m/z	z	RT	Fraction	Scan	Source File	Area F1	Area F2	Area F3	Area F4	Ar F
K.APSVFPLTPGHETQVA.K	Y	55.91	1649.8413	16	0.2	825.9281	2	48.79	7	F7:14307	17112021_RID_2349_DP7.raw					
K.APSVFPLTPGHETQ.V	Y	50.42	1479.7357	14	0.6	740.8756	2	45.66	7	F7:13339	17112021_RID_2349_DP7.raw					

total 2 peptides

TRINITY_DN7999

back to list

| Protein Coverage | Supporting Peptides |

Protein Coverage:

```
  1 AKSQLYLQMS SLKAEDTAVY YCTRHIYGLG WLYYAFDVWG PGTVVTVTND PPKAPSVFPL TPGHETQVAK ALSTASNVTI
 81 GCLIKNYFPE PATVQWNSGA ITSGIQNFPP VLLASNHYSH TSLLTIPASQ WQSESFQCNV EHAATNTRIN KIIERSAIRS
161 PVGPEITVFH SCDSRDSDGT VQLWCHISGF YPKQLDIDWK VGSRSGQLHP YNYPPWQNPG TYTFSTASVV NITQDDWLEG
241 NSYYCEATHA ASQTKVKGKA KKCKDGSSCP SGDIKVDILP PRPKALYIDR NSKISCVVSN LQNEQGLKFT WFREKSGHLT
321 PDAIETTEGS NGGYIVLSRL GIFSEDWDSG ETFTCVVEHP TFVSPVTKKI SKSRGKHSAP KVYVFPPHQE ELNPGRSVSL
401 TCLVTGFSPE AADVQWLEDH NPLSEDNSVT TPPMKNKHDD SYFLYSKLLV PKTDWNTKSF TCMVVHEDLP MKFTQRNISR
481 TQGKK
```

Supporting Peptides:

Peptide	Uniq	-10lgP	Mass	Length	ppm	m/z	z	RT	Fraction	Scan	Source File	Area F1	Area F2	Area F3	Area F4	Ar F
K.APSVFPLTPGHETQVA.K	Y	55.91	1649.8413	16	0.2	825.9281	2	48.79	7	F7:14307	17112021_RID_2349_DP7.raw					
K.APSVFPLTPGHETQ.V	Y	50.42	1479.7357	14	0.6	740.8756	2	45.66	7	F7:13339	17112021_RID_2349_DP7.raw					

total 2 peptides

TRINITY_DN13494

back to list

| Protein Coverage | Supporting Peptides |

Protein Coverage:

```
  1 MEGLRGGLSL AGLLSLLLVA GVMKEASTEV SCGVPALGRI VGGSDARNGQ WPWQANLNFN GVHACGGTLI TLQWLVTAAH
 81 CFPKANQIDM YEVALGSYQL KNPSPDIQVI LVEEVIKYPD YSEDESSQGD IALVKLKVPA TYTRTVRPIC LPASSVTFPP
161 GMRCTVTGWG NVLADTSLPS PMTLQQLEVP IIGLENCKCL YGRNPDPDGS HTLHNDMMCA GFAEGKKDAC QGDSGGPLSC
241 LVDNAWFLAG VVSWGDNCGE PNRPGVYIRL PVYADWIKQR VPEVQLQQVT VPPGPTPEES FCPNSTSGGG PYDDIQPSPG
321 WSRPLNPDKS TPSGSVSIAC MVGLPLLLLL LQSCLW
```

Supporting Peptides:

Peptide	Uniq	-10lgP	Mass	Length	ppm	m/z	z	RT	Fraction	Scan	Source File	Area F1	Area F2	Area F3	A
R.LPVYYADWIK.Q	Y	65.35	1103.6014	9	0.5	552.8083	2	54.90	2	F2:15975	12112021_RID_2349_DP2.raw		6.1755E5		
K.ANQIDMYEVALGSYQLK.N	Y	54.76	1941.9506	17	1.7	971.9843	2	60.47	2	F2:17731	12112021_RID_2349_DP2.raw		1.1688E5		

total 2 peptides

A8Y7P1.1
back to list

| Protein Coverage | Supporting Peptides |
Protein Coverage:

1 MSSGGLLLLL GLLTLWAELT PISGHDRPKF CYLPADPGEC LAHMR**SFYYD SESKK**CKEFI YGGCHGNANK FPSRDKCRQT

81 CGGK

Supporting Peptides:

Peptide	Uniq	-10lgP	Mass	Length	ppm	m/z	z	RT	Fraction	Scan	Source File	Area F1	Area F2	Area F3	Area F4	Area F5	Area F6	A
R.SFYYDSESKK.C	Y	70.58	1252.5612	10	0.4	627.2881	2	25.22	7	F7:6242	17112021_RID_2349_DP7.raw							3
R.SFYYDSESK.K	Y	43.22	1124.4662	9	0.1	563.2404	2	31.78	7	F7:8493	17112021_RID_2349_DP7.raw							4

total 2 peptides

CAL69604.1
back to list

| Protein Coverage | Supporting Peptides |
Protein Coverage:

1 MSSGGLLLLL GLLTLWAELT PISGHDRPKF CYLPADPGEC MAYIR**SFYYD SESKK**CKEFI YGGCHGNANN FPTRDKCRQT

81 CRGK

Supporting Peptides:

Peptide	Uniq	-10lgP	Mass	Length	ppm	m/z	z	RT	Fraction	Scan	Source File	Area F1	Area F2	Area F3	Area F4	Area F5	Area F6	A
R.SFYYDSESKK.C	Y	70.58	1252.5612	10	0.4	627.2881	2	25.22	7	F7:6242	17112021_RID_2349_DP7.raw							3
R.SFYYDSESK.K	Y	43.22	1124.4662	9	0.1	563.2404	2	31.78	7	F7:8493	17112021_RID_2349_DP7.raw							4

total 2 peptides

A8Y7N6.1
back to list

| Protein Coverage | Supporting Peptides |
Protein Coverage:

1 MSSGGLLLLL GLLTLWAELT PISGHDRPKF CYLPADPGEC MAYIR**SFYYD SESKK**CKEFI YGGCHGNANN FPTRDKCRQT

81 CRGK

Supporting Peptides:

Peptide	Uniq	-10lgP	Mass	Length	ppm	m/z	z	RT	Fraction	Scan	Source File	Area F1	Area F2	Area F3	Area F4	Area F5	Area F6	A
R.SFYYDSESKK.C	Y	70.58	1252.5612	10	0.4	627.2881	2	25.22	7	F7:6242	17112021_RID_2349_DP7.raw							3
R.SFYYDSESK.K	Y	43.22	1124.4662	9	0.1	563.2404	2	31.78	7	F7:8493	17112021_RID_2349_DP7.raw							4

total 2 peptides

CAL69609.1
back to list

| Protein Coverage | Supporting Peptides |
Protein Coverage:

1 MSSGGLLLLL GLLTLWAELT PISGHDRPKF CYLPADPGEC LAHMR**SFYYD SESKK**CKEFI YGGCHGNANK FPSRDKCRQT

81 CGGK

Supporting Peptides:

Peptide	Uniq	-10lgP	Mass	Length	ppm	m/z	z	RT	Fraction	Scan	Source File	Area F1	Area F2	Area F3	Area F4	Area F5	Area F6	A
R.SFYYDSESKK.C	Y	70.58	1252.5612	10	0.4	627.2881	2	25.22	7	F7:6242	17112021_RID_2349_DP7.raw							3
R.SFYYDSESK.K	Y	43.22	1124.4662	9	0.1	563.2404	2	31.78	7	F7:8493	17112021_RID_2349_DP7.raw							4

total 2 peptides

A8Y7P5.1
back to list

| Protein Coverage | Supporting Peptides |

Protein Coverage:

1 MSSGGLLLLL GLLTLWAELT PISGHDRPKF CYLPADPGEC LAHMR**SFYYD SESKK**CKEFI YGGCHGNANK FPSRDKCRQT

81 CGASAKGRPT

Supporting Peptides:

Peptide	Uniq	-10lgP	Mass	Length	ppm	m/z	z	RT	Fraction	Scan	Source File	Area F1	Area F2	Area F3	Area F4	Area F5	Area F6	[illegible]
R.SFYYDSESKK.C	Y	70.58	1252.5612	10	0.4	627.2881	2	25.22	7	F7:6242	17112021_RID_2349_DP7.raw							3
R.SFYYDSESK.K	Y	43.22	1124.4662	9	0.1	563.2404	2	31.78	7	F7:8493	17112021_RID_2349_DP7.raw							4

total 2 peptides

AFD04724.1
back to list

| Protein Coverage | Supporting Peptides |

Protein Coverage:

1 MSSGGLLLLL GLLALWAELT PISGHDRPKF CYLPADPGEC MAYIR**SFYYD SESKK**CKEFI YGGCHGNANN FPTRDKCRQT

81 CRAPRKGRHT

Supporting Peptides:

Peptide	Uniq	-10lgP	Mass	Length	ppm	m/z	z	RT	Fraction	Scan	Source File	Area F1	Area F2	Area F3	Area F4	Area F5	Area F6	[illegible]
R.SFYYDSESKK.C	Y	70.58	1252.5612	10	0.4	627.2881	2	25.22	7	F7:6242	17112021_RID_2349_DP7.raw							3
R.SFYYDSESK.K	Y	43.22	1124.4662	9	0.1	563.2404	2	31.78	7	F7:8493	17112021_RID_2349_DP7.raw							4

total 2 peptides

CAL69613.1
back to list

| Protein Coverage | Supporting Peptides |

Protein Coverage:

1 MSSGGLLLLL GLLTLWAELT PISGHDRPKF CYLPADPGEC LAHMR**SFYYD SESKK**CKEFI YGGCHGNANK FPSRDKCRQT

81 CGASAKGRPT

Supporting Peptides:

Peptide	Uniq	-10lgP	Mass	Length	ppm	m/z	z	RT	Fraction	Scan	Source File	Area F1	Area F2	Area F3	Area F4	Area F5	Area F6	[illegible]
R.SFYYDSESKK.C	Y	70.58	1252.5612	10	0.4	627.2881	2	25.22	7	F7:6242	17112021_RID_2349_DP7.raw							3
R.SFYYDSESK.K	Y	43.22	1124.4662	9	0.1	563.2404	2	31.78	7	F7:8493	17112021_RID_2349_DP7.raw							4

total 2 peptides

A8Y7N7.1
back to list

| Protein Coverage | Supporting Peptides |

Protein Coverage:

1 MSSGGLLLLL GLLTLWAELT PISGHDRPKF CYLPADPGEC MAYIR**SFYYD SESKK**CKEFI YGGCHGNANN FPTRDKCRQT

81 CRAPRKGRHT

Supporting Peptides:

Peptide	Uniq	-10lgP	Mass	Length	ppm	m/z	z	RT	Fraction	Scan	Source File	Area F1	Area F2	Area F3	Area F4	Area F5	Area F6	[illegible]
R.SFYYDSESKK.C	Y	70.58	1252.5612	10	0.4	627.2881	2	25.22	7	F7:6242	17112021_RID_2349_DP7.raw							3
R.SFYYDSESK.K	Y	43.22	1124.4662	9	0.1	563.2404	2	31.78	7	F7:8493	17112021_RID_2349_DP7.raw							4

total 2 peptides

CAL69605.1
back to list

| Protein Coverage | Supporting Peptides |

Protein Coverage:

1 MSSGGLLLLL GLLTLWAELT PISGHDRPKF CYLPADPGEC MAYIR**SFYYD SESKK**CKEFI YGGCHGNANN FPTRDKCRQT

81 CRAPRKGRHT

Supporting Peptides:

Peptide	Uniq	-10lgP	Mass	Length	ppm	m/z	z	RT	Fraction	Scan	Source File	Area F1	Area F2	Area F3	Area F4	Area F5	Area F6	[illegible]
R.SFYYDSESKK.C	Y	70.58	1252.5612	10	0.4	627.2881	2	25.22	7	F7:6242	17112021_RID_2349_DP7.raw							3

total 2 peptides

Peptide	Uniq	-10lgP	Mass	Length	ppm	m/z	z	RT	Fraction	Scan	Source File	Area F1	Area F2	Area F3	Area F4	Area F5	Area F6	A
R.SFYYDSESK.K	Y	43.22	1124.4662	9	0.1	563.2404	2	31.78	7	F7:8493	17112021_RID_2349_DP7.raw							4

total 2 peptides

TRINITY_DN960
back to list

| Protein Coverage | Supporting Peptides |
Protein Coverage:

```
1   AWDGLPRPWC PAVRGSRARK ARSEEEEEEA PAELRPQSAP SGPVPSPPPR PGEGGPASDG WLDRDGSPLL LPPPPPPAGA
81  EDAVGASPAG RLYKARTAMP PLRANPARQA NAAPFGSAVR AFEDSLSLSL ARSRPEALPA PAHSGMFVSR LAASGLLLLS
161 LLALSLDGKP LKQKWPMPGP EIPPLEQRWP GPKVPPLEQR WPGPEIPPLE QRWPGPQIPP LQQKWRGPLQ PEAPPLMEPH
241 QLSAGGTTAL REELSPRAEA AQHPGGSGGG WRDENEPPKK LPPSSCFGHK IDRIGSVSGL GCNKFKPGH
```

Supporting Peptides:

Peptide	Uniq	-10lgP	Mass	Length	ppm	m/z	z	RT	Fraction	Scan	Source File	Area F1	Area F2	Area F3	Area F4	Area F
R.WPGPQIPPLQQK.W	Y	61.81	1387.7612	12	0.7	694.8884	2	49.77	5	F5:14595	17112021_RID_2349_DP5.raw					3.7975E
R.WPGPEIPPLEQR.W	Y	57.32	1417.7354	12	0.2	709.8751	2	53.12	5	F5:15698	17112021_RID_2349_DP5.raw					2.0576E

total 2 peptides

TRINITY_DN960
back to list

| Protein Coverage | Supporting Peptides |
Protein Coverage:

```
1   AWDGLPRPWC PAVRGSRARK ARSEEEEEEA PAELRPQSAP SGPVPSPPPR PGEGGPASDG WLDRDGSPLL LPPPPPPAGA
81  EDAVGASPAG RLYKARTAMP PLRANPARQA NAAPFGSAVR AFEDSLSLSL ARSRPEALPA PAHSGMFVSR LAASGLLLLS
161 LLALSLDGKP LKQKWPMPGP EIPPLEQRWP GPKVPPLEQR WPGPEIPPLE QRWPGPKVPP LEQRWPGPEI PPLEQRWPGP
241 KVPPLEQRWP GPQIPPLQQK WRGPLQPEAP PLMEPHQLSA GGTTALREEL SPRAEAAQHP GGSGGGWRDE NEPPKKLPPS
321 SCFGHKIDRI GSVSGLGCNK FKPGH
```

Supporting Peptides:

Peptide	Uniq	-10lgP	Mass	Length	ppm	m/z	z	RT	Fraction	Scan	Source File	Area F1	Area F2	Area F3	Area F4	Area F
R.WPGPQIPPLQQK.W	Y	61.81	1387.7612	12	0.7	694.8884	2	49.77	5	F5:14595	17112021_RID_2349_DP5.raw					3.7975E
R.WPGPEIPPLEQR.W	Y	57.32	1417.7354	12	0.2	709.8751	2	53.12	5	F5:15698	17112021_RID_2349_DP5.raw					2.0576E

total 2 peptides

TRINITY_DN960
back to list

| Protein Coverage | Supporting Peptides |
Protein Coverage:

```
1   KKERKKERKK ERKKERKKER KKERKKEAGW GGRRGFSTPT FCQPSPTQSV CKAWDGLPRP WCPAVRGSRA RKARSEEEEE
81  EAPAELRPQS APSGPVPSPP PRPGEGGPAS DGWLDRDGSP LLLPPPPPPA GAEDAVGASP AGRLYKARTA MPPLRANPAR
161 QANAAPFGSA VRAFEDSLSL SLARSRPEAL PAPAHSGMFV SRLAASGLLL LSLLALSLDG KPLKQKWPMP GPEIPPLEQR
241 WPGPKVPPLE QRWPGPEIPP LEQRWPGPQI PPLQQKWRGP LQPEAPPLME PHQVSRRRAP PARLAFPRRG SRACFGSVLG
321 GGEETGGPFA ALGGSGDGDV GLGSRGGESR ARSGEGTTHP IGPSSLGHCS RGAPGWSEGA GGWWERTAGP PLAFFPPCTR
401 PV
```

Supporting Peptides:

Peptide	Uniq	-10lgP	Mass	Length	ppm	m/z	z	RT	Fraction	Scan	Source File	Area F1	Area F2	Area F3	Area F4	Area F
R.WPGPQIPPLQQK.W	Y	61.81	1387.7612	12	0.7	694.8884	2	49.77	5	F5:14595	17112021_RID_2349_DP5.raw					3.7975E
R.WPGPEIPPLEQR.W	Y	57.32	1417.7354	12	0.2	709.8751	2	53.12	5	F5:15698	17112021_RID_2349_DP5.raw					2.0576E

total 2 peptides

TRINITY_DN960
back to list

| Protein Coverage | Supporting Peptides |
Protein Coverage:

1 KKERKKERKK ERKKERKKER KKERKKEAGW GGRRGFSTPT FCQPSPTQSV CKAWDGLPRP WCPAVRGSRA RKARSEEEEE
81 EAPAELRPQS APSGPVPSPP PRPGEGGPAS DGWLDRDGSP LLLPPPPPPA GAEDAVGASP AGRLYKARTA MPPLRANPAR
161 QANAAPFGSA VRAFEDSLSL SLARSRPEAL PAPAHSGMFV SRLAASGLLL LSLLALSLDG KPLKQKWPMP GPEIPPLEQR
241 WPGPKVPPLE QR**WPGPEIPP LEQR**WPGPKV PPLEQRWPGP EIPPLEQRWP GPKVPPLEQR **WPGPQIPPLQ QK**WRGPLQPE
321 APPLMEPHQV SRRRAPPARL AFPRRGSRAC FGSVLGGGEE TGGPFAALGG SGDGDVGLGS RGGESRARSG EGTTHPIGPS
401 SLGHCSRGAP GWSEGAGGWW ERTAGPPLAF FPPCTRPV

Supporting Peptides:

Peptide	Uniq	-10lgP	Mass	Length	ppm	m/z	z	RT	Fraction	Scan	Source File	Area F1	Area F2	Area F3	Area F4	Area F
R.WPGPQIPPLQQK.W	Y	61.81	1387.7612	12	0.7	694.8884	2	49.77	5	F5:14595	17112021_RID_2349_DP5.raw					3.7975E
R.WPGPEIPPLEQR.W	Y	57.32	1417.7354	12	0.2	709.8751	2	53.12	5	F5:15698	17112021_RID_2349_DP5.raw					2.0576E

total 2 peptides

TRINITY_DN960
back to list

| Protein Coverage | Supporting Peptides |
Protein Coverage:

1 AWDGLPRPWC PAVRGSRARK ARSEEEEEEA PAELRPQSAP SGPVPSPPPR PGEGGPASDG WLDRDGSPLL LPPPPPPAGA
81 EDAVGASPAG RLYKARTAMP PLRANPARQA NAAPFGSAVR AFEDSLSLSL ARSRPEALPA PAHSGMFVSR LAASGLLLLS
161 LLALSLDGKP LKQKWPMPGP EIPPLEQRWP GPKVPPLEQR **WPGPEIPPLE QRWPGPQIPP LQQK**WRGPLQ PEAPPLMEPH
241 QVSRRRAPPA RLAFPRRGSR ACFGSVLGGG EETGGPFAAL GGSGDGDVGL GSRGGGESRA RSGEGTTHPI GPSSLGHCSR
321 GAPGWSEGAG GWWERTAGPP LAFFPPCTRP V

Supporting Peptides:

Peptide	Uniq	-10lgP	Mass	Length	ppm	m/z	z	RT	Fraction	Scan	Source File	Area F1	Area F2	Area F3	Area F4	Area F
R.WPGPQIPPLQQK.W	Y	61.81	1387.7612	12	0.7	694.8884	2	49.77	5	F5:14595	17112021_RID_2349_DP5.raw					3.7975E
R.WPGPEIPPLEQR.W	Y	57.32	1417.7354	12	0.2	709.8751	2	53.12	5	F5:15698	17112021_RID_2349_DP5.raw					2.0576E

total 2 peptides

TRINITY_DN960
back to list

| Protein Coverage | Supporting Peptides |
Protein Coverage:

1 MGGQKGFLNP NFLPTFPNSK RLQAWDGLPR PWCPAVRGSR ARKARSEEEE EEAPAELRPQ SAPSGPVPSP PPRPGEGGPA
81 SDGWLDRDGS PLLLPPPPPP AGAEDAVGAS PAGRLYKART AMPPLRANPA RQANAAPFGS AVRAFEDSLS LSLARSRPEA
161 LPAPAHSGMF VSRLAASGLL LLSLLALSLD GKPLKQKWPM PGPEIPPLEQ RWPGPKVPPL EQR**WPGPEIP PLEQRWPGPQ**
241 **IPPLQQK**WRG PLQPEAPPLM EPHQVSRRRA PPARLAFPRR GSRACFGSVL GGGEETGGPF AALGGSGDGD VGLGSRGGES
321 RARSGEGTTH PIGPSSLGHC SRGAPGWSEG AGGWWERTAG PPLAFFPPCT RPV

Supporting Peptides:

Peptide	Uniq	-10lgP	Mass	Length	ppm	m/z	z	RT	Fraction	Scan	Source File	Area F1	Area F2	Area F3	Area F4	Area F
R.WPGPQIPPLQQK.W	Y	61.81	1387.7612	12	0.7	694.8884	2	49.77	5	F5:14595	17112021_RID_2349_DP5.raw					3.7975E
R.WPGPEIPPLEQR.W	Y	57.32	1417.7354	12	0.2	709.8751	2	53.12	5	F5:15698	17112021_RID_2349_DP5.raw					2.0576E

total 2 peptides

TRINITY_DN960
back to list

| Protein Coverage | Supporting Peptides |
Protein Coverage:

1 MGGQKGFLNP NFLPTFPNSK RLQAWDGLPR PWCPAVRGSR ARKARSEEEE EEAPAELRPQ SAPSGPVPSP PPRPGEGGPA
81 SDGWLDRDGS PLLLPPPPPP AGAEDAVGAS PAGRLYKART AMPPLRANPA RQANAAPFGS AVRAFEDSLS LSLARSRPEA
161 LPAPAHSGMF VSRLAASGLL LLSLLALSLD GKPLKQKWPM PGPEIPPLEQ RWPGPKVPPL EQR**WPGPEIP PLEQR**WPGPK
241 VPPLEQRWPG PEIPPLEQRW PGPKVPPLEQ R**WPGPQIPPL QQK**WRGPLQP EAPPLMEPHQ VSRRRAPPAR LAFPRRGSRA
321 CFGSVLGGGE ETGGPFAALG GSGDGDVGLG SRGGESRARS GEGTTHPIGP SSLGHCSRGA PGWSEGAGGW WERTAGPPLA
401 FFPPCTRPV

Supporting Peptides:

Peptide	Uniq	-10lgP	Mass	Length	ppm	m/z	z	RT	Fraction	Scan	Source File	Area F1	Area F2	Area F3	Area F4	Area F
R.WPGPQIPPLQQK.W	Y	61.81	1387.7612	12	0.7	694.8884	2	49.77	5	F5:14595	17112021_RID_2349_DP5.raw					3.7975E

total 2 peptides

Peptide	Uniq	-10lgP	Mass	Length	ppm	m/z	z	RT	Fraction	Scan	Source File	Area F1	Area F2	Area F3	Area F4	Area F
R.WPGPEIPPLEQR.W	Y	57.32	1417.7354	12	0.2	709.8751	2	53.12	5	F5:15698	17112021_RID_2349_DP5.raw					2.0576E

total 2 peptides

TRINITY_DN672
back to list

| Protein Coverage | Supporting Peptides |
Protein Coverage:

1 QSVAMALNCY GGLTVLLFCF GAFSQAQVTQ NGPVSVDIAG TTVLTCALQG VEFISPTNPS WYQHKEGKSP RLLIYKSRER
81 ASGTPQRFSA DTYGNTAKLT ISSVEVGDEG DYYCAVWHNN GFVFGGGTRV TVTGQPTVSP S**VQVFAPSPE EIR**SPNPHTV
161 VCLLSEFYPR SFVLQWKCDD KVMTSGVETT KATKQGDKYL ASSYLKLSAS EYEKYGTFTC EVTHERKTIV KSVSKSGSC

Supporting Peptides:

Peptide	Uniq	-10lgP	Mass	Length	ppm	m/z	z	RT	Fraction	Scan	Source File	Area F1	Area F2	Area F3	Area F4	Area F5	Are F6
S.VQVFAPSPEEIR.S	Y	51.69	1370.7194	12	1.2	686.3678	2	44.58	7	F7:12865	17112021_RID_2349_DP7.raw						
F.APSPEEIR.S	Y	39.94	897.4556	8	-0.4	449.7349	2	24.57	7	F7:5992	17112021_RID_2349_DP7.raw						

total 2 peptides

1OQS
back to list

| Protein Coverage | Supporting Peptides |
Protein Coverage:

1 **NLFQFAR**MIN GKLGAFSVWN YISYGCYCGW GGQGTPKDAT DRCCFVHDCC YGGVKGCNPK **LAIYSYSFQR** GNIVCGRNNG
81 CLRTICECDR VAANCFHQNK NTYNKEYKFL SSSKCRQRSE QC

Supporting Peptides:

Peptide	Uniq	-10lgP	Mass	Length	ppm	m/z	z	RT	Fraction	Scan	Source File	Area F1	Area F2	Area F3	Area F4	Area F5	Area F6	
K.LAIYSYSFQR.G	Y	68.53	1246.6345	10	0.4	624.3248	2	48.21	7	F7:14191	17112021_RID_2349_DP7.raw							[illegible]
NLFQFAR.M	Y	42.54	894.4711	7	0.0	448.2429	2	47.18	7	F7:13859	17112021_RID_2349_DP7.raw							[illegible]

total 2 peptides

1OQS
back to list

| Protein Coverage | Supporting Peptides |
Protein Coverage:

1 **NLFQFAR**MIN GKLGAFSVWN YISYGCYCGW GGQGTPKDAT DRCCFVHDCC YGGVKGCNPK **LAIYSYSFQR** GNIVCGRNNG
81 CLRTICECDR VAANCFHQNK NTYNKEYKFL SSSKCRQRSE QC

Supporting Peptides:

Peptide	Uniq	-10lgP	Mass	Length	ppm	m/z	z	RT	Fraction	Scan	Source File	Area F1	Area F2	Area F3	Area F4	Area F5	Area F6	
K.LAIYSYSFQR.G	Y	68.53	1246.6345	10	0.4	624.3248	2	48.21	7	F7:14191	17112021_RID_2349_DP7.raw							[illegible]
NLFQFAR.M	Y	42.54	894.4711	7	0.0	448.2429	2	47.18	7	F7:13859	17112021_RID_2349_DP7.raw							[illegible]

total 2 peptides

1OQS
back to list

| Protein Coverage | Supporting Peptides |
Protein Coverage:

1 **NLFQFAR**MIN GKLGAFSVWN YISYGCYCGW GGQGTPKDAT DRCCFVHDCC YGGVKGCNPK **LAIYSYSFQR** GNIVCGRNNG
81 CLRTICECDR VAANCFHQNK NTYNKEYKFL SSSKCRQRSE QC

Supporting Peptides:

Peptide	Uniq	-10lgP	Mass	Length	ppm	m/z	z	RT	Fraction	Scan	Source File	Area F1	Area F2	Area F3	Area F4	Area F5	Area F6	
K.LAIYSYSFQR.G	Y	68.53	1246.6345	10	0.4	624.3248	2	48.21	7	F7:14191	17112021_RID_2349_DP7.raw							[illegible]
NLFQFAR.M	Y	42.54	894.4711	7	0.0	448.2429	2	47.18	7	F7:13859	17112021_RID_2349_DP7.raw							[illegible]

total 2 peptides

1OQS
back to list

| Protein Coverage | Supporting Peptides |
Protein Coverage:

1 **NLFQFAR**MIN GKLGAFSVWN YISYGCYCGW GGQGTPKDAT DRCCFVHDCC YGGVKGCNPK **LAIYSYSFQR** GNIVCGRNNG

81 CLRTICECDR VAANCFHQNK NTYNKEYKFL SSSKCRQRSE QC

Supporting Peptides:

Peptide	Uniq	-10lgP	Mass	Length	ppm	m/z	z	RT	Fraction	Scan	Source File	Area F1	Area F2	Area F3	Area F4	Area F5	Area F6
K.LAIYSYSFQR.G	Y	68.53	1246.6345	10	0.4	624.3248	2	48.21	7	F7:14191	17112021_RID_2349_DP7.raw						
NLFQFAR.M	Y	42.54	894.4711	7	0.0	448.2429	2	47.18	7	F7:13859	17112021_RID_2349_DP7.raw						

total 2 peptides

CAA48456.1
back to list

| Protein Coverage | Supporting Peptides |
Protein Coverage:

1 MRTLWIVAVC LIGVEG**NLFQ FAR**MINGKLG AFSVWNYISY GCYCGWGGQG TPKDATDRCC FVHDCCYGGV KGCNPK**LAIY**

81 **SYSFQR**GNIV CGRNNGCLRT ICECDRVAAN CFHQNKNTYN KEYKFLSSSK CRQRSEQC

Supporting Peptides:

Peptide	Uniq	-10lgP	Mass	Length	ppm	m/z	z	RT	Fraction	Scan	Source File	Area F1	Area F2	Area F3	Area F4	Area F5	Area F6
K.LAIYSYSFQR.G	Y	68.53	1246.6345	10	0.4	624.3248	2	48.21	7	F7:14191	17112021_RID_2349_DP7.raw						
G.NLFQFAR.M	Y	42.54	894.4711	7	0.0	448.2429	2	47.18	7	F7:13859	17112021_RID_2349_DP7.raw						

total 2 peptides

AAZ53185.1
back to list

| Protein Coverage | Supporting Peptides |
Protein Coverage:

1 MRTLWILAVC LIGVEG**NLFQ FAR**MINGKLG AFSVWNYISY GCYCGWGGQG TPKDATDRCC FVHDCCYGGV KGCNPK**LAIY**

81 **SYSFQR**GNIV CGRNNGCLRT ICECDRVAAN CFHQNKNTYN KRYKFLSSYY CRQTSEKC

Supporting Peptides:

Peptide	Uniq	-10lgP	Mass	Length	ppm	m/z	z	RT	Fraction	Scan	Source File	Area F1	Area F2	Area F3	Area F4	Area F5	Area F6
K.LAIYSYSFQR.G	Y	68.53	1246.6345	10	0.4	624.3248	2	48.21	7	F7:14191	17112021_RID_2349_DP7.raw						
G.NLFQFAR.M	Y	42.54	894.4711	7	0.0	448.2429	2	47.18	7	F7:13859	17112021_RID_2349_DP7.raw						

total 2 peptides

Q02471.1
back to list

| Protein Coverage | Supporting Peptides |
Protein Coverage:

1 MRTLWIVAVC LIGVEG**NLFQ FAR**MINGKLG AFSVWNYISY GCYCGWGGQG TPKDATDRCC FVHDCCYGGV KGCNPK**LAIY**

81 **SYSFQR**GNIV CGRNNGCLRT ICECDRVAAN CFHQNKNTYN KEYKFLSSSK CRQRSEQC

Supporting Peptides:

Peptide	Uniq	-10lgP	Mass	Length	ppm	m/z	z	RT	Fraction	Scan	Source File	Area F1	Area F2	Area F3	Area F4	Area F5	Area F6
K.LAIYSYSFQR.G	Y	68.53	1246.6345	10	0.4	624.3248	2	48.21	7	F7:14191	17112021_RID_2349_DP7.raw						
G.NLFQFAR.M	Y	42.54	894.4711	7	0.0	448.2429	2	47.18	7	F7:13859	17112021_RID_2349_DP7.raw						

total 2 peptides

S29298
back to list

| Protein Coverage | Supporting Peptides |
Protein Coverage:

1 MRTLWIVAVC LIGVEG**NLFQ FAR**MINGKLG AFSVWNYISY GCYCGWGGQG TPKDATDRCC FVHDCCYGGV KGCNPK**LAIY**

81 **SYSFQR**GNIV CGRNNGCLRT ICECDRVAAN CFHQNKNTYN KEYKFLSSSK CRQRSEQC

Supporting Peptides:

Peptide	Uniq	-10lgP	Mass	Length	ppm	m/z	z	RT	Fraction	Scan	Source File	Area F1	Area F2	Area F3	Area F4	Area F5	Area F6
K.LAIYSYSFQR.G	Y	68.53	1246.6345	10	0.4	624.3248	2	48.21	7	F7:14191	17112021_RID_2349_DP7.raw						
G.NLFQFAR.M	Y	42.54	894.4711	7	0.0	448.2429	2	47.18	7	F7:13859	17112021_RID_2349_DP7.raw						

total 2 peptides

ACD43467.1
back to list

| Protein Coverage | Supporting Peptides |
Protein Coverage:

1 MRTLWIVAVC LIGVEG**NLFQ FAR**MINGKLG AFSVWNYISY GCYCGWGGQG TPKDATDRCC FVHDCCYGGV KGCNPK**LAIY**

81 **SYSFQR**GNIV CGRNNGCLRT ICECDRVAAN CFHQNKNTYN KEYKFLSSSK CRQRSEQC

Supporting Peptides:

Peptide	Uniq	-10lgP	Mass	Length	ppm	m/z	z	RT	Fraction	Scan	Source File	Area F1	Area F2	Area F3	Area F4	Area F5	Area F6	
K.LAIYSYSFQR.G	Y	68.53	1246.6345	10	0.4	624.3248	2	48.21	7	F7:14191	17112021_RID_2349_DP7.raw							[illegible]
G.NLFQFAR.M	Y	42.54	894.4711	7	0.0	448.2429	2	47.18	7	F7:13859	17112021_RID_2349_DP7.raw							[illegible]

total 2 peptides

ACD43465.1
back to list

| Protein Coverage | Supporting Peptides |
Protein Coverage:

1 MRTLWIVAVC LIGVEG**NLFQ FAR**MINGKLG AFSVWNYISY GCYCGWGGQG TPKDATDRCC FVHDCCYGGV KGCNPK**LAIY**

81 **SYSFQR**GNIV CGRNNGCLRT ICECDRVAAN CFHQNKNTYN KEYKFLSSSK CRQRSEQC

Supporting Peptides:

Peptide	Uniq	-10lgP	Mass	Length	ppm	m/z	z	RT	Fraction	Scan	Source File	Area F1	Area F2	Area F3	Area F4	Area F5	Area F6	
K.LAIYSYSFQR.G	Y	68.53	1246.6345	10	0.4	624.3248	2	48.21	7	F7:14191	17112021_RID_2349_DP7.raw							[illegible]
G.NLFQFAR.M	Y	42.54	894.4711	7	0.0	448.2429	2	47.18	7	F7:13859	17112021_RID_2349_DP7.raw							[illegible]

total 2 peptides

AFD04723.1
back to list

| Protein Coverage | Supporting Peptides |
Protein Coverage:

1 MSSGGLLLLL GLLTLWAELT PISGQDRPKF CNLAPESGRC RGHLR**RIYYN PDSNK**CEVFF YGGCGGNDNN FETRKKCRQT

81 CGAPRKGRPT

Supporting Peptides:

Peptide	Uniq	-10lgP	Mass	Length	ppm	m/z	z	RT	Fraction	Scan	Source File	Area F1	Area F2	Area F3	Area F4	Area F[illegible]
R.IYYNPDSNK.C	Y	52.18	1112.5138	9	-0.5	557.2639	2	26.99	7	F7:6866	17112021_RID_2349_DP7.raw				8.1126E5	8.8307E[illegible]
R.RIYYNPDSNK.C	Y	51.68	1268.6149	10	-0.6	635.3143	2	23.49	7	F7:5623	17112021_RID_2349_DP7.raw					

total 2 peptides

AFB74191.1
back to list

| Protein Coverage | Supporting Peptides |
Protein Coverage:

1 MSSGGLLLLL GLLTLWAELT PISGQDRPKF CNLAPESGRC RGHLR**RIYYN PDSNK**CEVFF YGGCGGNDNN FETRKKCRQT

81 CGAPRKGRPT

Supporting Peptides:

Peptide	Uniq	-10lgP	Mass	Length	ppm	m/z	z	RT	Fraction	Scan	Source File	Area F1	Area F2	Area F3	Area F4	Area F[illegible]
R.IYYNPDSNK.C	Y	52.18	1112.5138	9	-0.5	557.2639	2	26.99	7	F7:6866	17112021_RID_2349_DP7.raw				8.1126E5	8.8307E[illegible]
R.RIYYNPDSNK.C	Y	51.68	1268.6149	10	-0.6	635.3143	2	23.49	7	F7:5623	17112021_RID_2349_DP7.raw					

total 2 peptides

A8Y7N4.1
back to list

| Protein Coverage | Supporting Peptides |
Protein Coverage:

1 MSSGGLLLLL GLLTLWAELT PISGQDRPKF CNLAPESGRC RGHLR**RIYYN PDSNK**CEVFF YGGCGGNDNN FETRKKCRQT

81 CGAPRKGRPT

Supporting Peptides:

Peptide	Uniq	-10lgP	Mass	Length	ppm	m/z	z	RT	Fraction	Scan	Source File	Area F1	Area F2	Area F3	Area F4	Area F[illegible]

Peptide	Uniq	-10lgP	Mass	Length	ppm	m/z	z	RT	Fraction	Scan	Source File	Area F1	Area F2	Area F3	Area F4	Area F5
R.IYYNPDSNK.C	Y	52.18	1112.5138	9	-0.5	557.2639	2	26.99	7	F7:6866	17112021_RID_2349_DP7.raw				8.1126E5	8.8307E
R.RIYYNPDSNK.C	Y	51.68	1268.6149	10	-0.6	635.3143	2	23.49	7	F7:5623	17112021_RID_2349_DP7.raw					

total 2 peptides

H6VC05.1
back to list

| Protein Coverage | Supporting Peptides |
Protein Coverage:

1 MSSGGLLLLL GLLTLWAELT PISGQDRPKF CNLAPESGRC RGHLR**RIYYN PDSNK**CEVFF YGGCGGNDNN FETRKKCRQT

81 CGAPRKGRPT

Supporting Peptides:

Peptide	Uniq	-10lgP	Mass	Length	ppm	m/z	z	RT	Fraction	Scan	Source File	Area F1	Area F2	Area F3	Area F4	Area F5
R.IYYNPDSNK.C	Y	52.18	1112.5138	9	-0.5	557.2639	2	26.99	7	F7:6866	17112021_RID_2349_DP7.raw				8.1126E5	8.8307E
R.RIYYNPDSNK.C	Y	51.68	1268.6149	10	-0.6	635.3143	2	23.49	7	F7:5623	17112021_RID_2349_DP7.raw					

total 2 peptides

CAL69602.1
back to list

| Protein Coverage | Supporting Peptides |
Protein Coverage:

1 MSSGGLLLLL GLLTLWAELT PISGQDRPKF CNLAPESGRC RGHLR**RIYYN PDSNK**CEVFF YGGCGGNDNN FETRKKCRQT

81 CGAPRKGRPT

Supporting Peptides:

Peptide	Uniq	-10lgP	Mass	Length	ppm	m/z	z	RT	Fraction	Scan	Source File	Area F1	Area F2	Area F3	Area F4	Area F5
R.IYYNPDSNK.C	Y	52.18	1112.5138	9	-0.5	557.2639	2	26.99	7	F7:6866	17112021_RID_2349_DP7.raw				8.1126E5	8.8307E
R.RIYYNPDSNK.C	Y	51.68	1268.6149	10	-0.6	635.3143	2	23.49	7	F7:5623	17112021_RID_2349_DP7.raw					

total 2 peptides

ABD24041.1
back to list

| Protein Coverage | Supporting Peptides |
Protein Coverage:

1 MSSGGLLLLL GLLTLWAEPT PISGQDRPKF CFLRPDFGRY GHPRPR**FYYN PATNQ**CQGFL AQR**SRENTNN FDTRDK**CRQT

81 CGRK

Supporting Peptides:

Peptide	Uniq	-10lgP	Mass	Length	ppm	m/z	z	RT	Fraction	Scan	Source File	Area F1	Area F2	Area F3	Area F4
R.FYYNPATNQ.C	Y	53.01	1116.4875	9	0.4	559.2513	2	42.16	7	F7:12041	17112021_RID_2349_DP7.raw	1.5234E5			
R.SRENTNNFDTRDK.C	Y	41.21	1595.7288	13	0.2	532.9170	3	18.70	1	F1:4666	12112021_RID_2349_DP1.raw	1.8585E5			
R.SRENTNNFDTR.D	Y	39.27	1352.6069	11	-0.4	451.8761	3	17.84	2	F2:3446	12112021_RID_2349_DP2.raw		1.6087E5		

total 3 peptides

Q2ES49.1
back to list

| Protein Coverage | Supporting Peptides |
Protein Coverage:

1 MSSGGLLLLL GLLTLWAEPT PISGQDRPKF CFLRPDFGRY GHPRPR**FYYN PATNQ**CQGFL AQR**SRENTNN FDTRDK**CRQT

81 CGRK

Supporting Peptides:

Peptide	Uniq	-10lgP	Mass	Length	ppm	m/z	z	RT	Fraction	Scan	Source File	Area F1	Area F2	Area F3	Area F4
R.FYYNPATNQ.C	Y	53.01	1116.4875	9	0.4	559.2513	2	42.16	7	F7:12041	17112021_RID_2349_DP7.raw	1.5234E5			
R.SRENTNNFDTRDK.C	Y	41.21	1595.7288	13	0.2	532.9170	3	18.70	1	F1:4666	12112021_RID_2349_DP1.raw	1.8585E5			
R.SRENTNNFDTR.D	Y	39.27	1352.6069	11	-0.4	451.8761	3	17.84	2	F2:3446	12112021_RID_2349_DP2.raw		1.6087E5		

total 3 peptides

TRINITY_DN157

back to list

| Protein Coverage | Supporting Peptides |

Protein Coverage:

```
 1 MVHWTAEEKS TITAVWSKVD VPAVGAEALS NLLIVCPWTQ RFFPNFGNLS NAAAIQSNAQ VKAHGKKVFT AFGDAVKNPE
81 GVKDTFAKLS ELHCDKLHVD PVNFKLLGDS LITVLAAHFG KEFTPHVQAC FQKLVAVVAH ALAHRYH
```

Supporting Peptides:

Peptide	Uniq	-10lgP	Mass	Length	ppm	m/z	z	RT	Fraction	Scan	Source File	Area F1	Area F2	Area F3	Area F4	Area F5	Area F6
K.STITAVWSK.V	Y	55.57	991.5338	9	0.2	496.7743	2	39.29	7	F7:11035	17112021_RID_2349_DP7.raw						
K.VFTAFGDAVK.N	N	40.99	1053.5494	10	0.9	527.7825	2	44.38	7	F7:12863	17112021_RID_2349_DP7.raw						

total 2 peptides

AAB36402.1

back to list

| Protein Coverage | Supporting Peptides |

Protein Coverage:

```
 1 DCSSGWTAYG KHCYKVFDEP KTWEDAEKFC SEQANGGHLV SFRSSKEADF VVTLTAQTKE SEIVWMGLSK IWNQCDWGWT
81 NGAKLNYEAW AEAESYCVWF SSTNKEWKSR PCSLFGHFVC KSPAW
```

Supporting Peptides:

Peptide	Uniq	-10lgP	Mass	Length	ppm	m/z	z	RT	Fraction	Scan	Source File	Area F1	Area F2	Area F3	Area F4	Area F5	Area F6	A
K.TWEDAEK.F	N	42.92	877.3817	7	0.0	439.6982	2	23.16	7	F7:5532	17112021_RID_2349_DP7.raw							0
N.GGHLVSFR.S	Y	42.19	871.4664	8	0.1	436.7405	2	27.12	7	F7:6878	17112021_RID_2349_DP7.raw							3.9
K.TWEDAEKF.C	N	40.38	1024.4502	8	-0.9	513.2319	2	40.86	7	F7:11654	17112021_RID_2349_DP7.raw							9.4

total 3 peptides

Q9PSM8.1

back to list

| Protein Coverage | Supporting Peptides |

Protein Coverage:

```
 1 DCSSGWTAYG KHCYKVFDEP KTWEDAEKFC SEQANGGHLV SFRSSKEADF VVTLTAQTKE SEIVWMGLSK IWNQCDWGWT
81 NGAKLNYEAW AEAESYCVWF SSTNKEWKSR PCSLFGHFVC KSPAW
```

Supporting Peptides:

Peptide	Uniq	-10lgP	Mass	Length	ppm	m/z	z	RT	Fraction	Scan	Source File	Area F1	Area F2	Area F3	Area F4	Area F5	Area F6	A
K.TWEDAEK.F	N	42.92	877.3817	7	0.0	439.6982	2	23.16	7	F7:5532	17112021_RID_2349_DP7.raw							0
N.GGHLVSFR.S	Y	42.19	871.4664	8	0.1	436.7405	2	27.12	7	F7:6878	17112021_RID_2349_DP7.raw							3.9
K.TWEDAEKF.C	N	40.38	1024.4502	8	-0.9	513.2319	2	40.86	7	F7:11654	17112021_RID_2349_DP7.raw							9.4

total 3 peptides

XP_029141923.1

back to list

| Protein Coverage | Supporting Peptides |

Protein Coverage:

```
  1 MVPWLNLMSL LVVLRGVQSE VQLVESGGEG RRPGESLHLS CQGSGFTFGN YWMVWVRQAP GKGLECVLVF KGYQYGCHMV
 81 RMERIRSVST ESTEFYFTNS SFIRAELHIT PYEYLLDLRK QVEIMLSACL NIFPSVRILH TFERAKMALW LNIMFLLVVL
161 GGAQSEVQLV ETGGDVRRPG ESLRLSCQAS GFTFSSSWMS WVRQAPGKGL EWVARIRYDS ATIYYPDKVK GRFTISRDNA
       248
241 KSQLYLQMNS LKAEDTAVYY CVRDTMVYPD VYWFSSQGNT NMRWAIFSMS AMEDEGTLET QSSVYLFYPE GYVNSVTASC
```

Supporting Peptides:

Peptide	Uniq	-10lgP	Mass	Length	ppm	m/z	z	RT	Fraction	Scan	Source File	Area F1	Area F2	Area F3	Area F4	Area
K.SQLYLQMNSLK.A	N	55.00	1323.6857	11	0.6	662.8505	2	45.98	7	F7:13334	17112021_RID_2349_DP7.raw					
S.EVQLVETGGDVR.R	Y	53.24	1300.6622	12	0.4	651.3386	2	35.98	7	F7:9917	17112021_RID_2349_DP7.raw					
K.SQLYLQM(+15.99)NSLK.A	N	41.82	1339.6805	11	0.6	670.8480	2	37.34	7	F7:10474	17112021_RID_2349_DP7.raw					

total 3 peptides

TRINITY_DN1424

back to list

| Protein Coverage | Supporting Peptides |

Protein Coverage:

1 GRLARGRGRM LAARRALWPG WRGWSCERRK MKLLAAAALL GLGGGLLMLL LPGTRAEERK KGPKVTVKVQ FDVKIGEEEV
81 GRIVIGLFGK TVPK**TVENFV ALTTGEK**GFG FKGSKFHRII KDFMIQGGDF TRGDGTGGKS IYGDRFPDEN FKLKHYGPGW
161 VSMANAGKDT NGSQFFITTV KTSWLDGKHV VFGKVLEGMD VVRKMESTKT DSRDKPLKDV IIADCNVIEV EKPFAIAKE

Supporting Peptides:

Peptide	Uniq	-10lgP	Mass	Length	ppm	m/z	z	RT	Fraction	Scan	Source File	Area F1	Area F2	Area F3	Area F4	Area
K.TVENFVALTTGEK.G	Y	65.95	1407.7245	13	0.5	704.8699	2	47.57	7	F7:13936	17112021_RID_2349_DP7.raw					6.0446

total 1 peptides

ART88739.1
back to list

| Protein Coverage | Supporting Peptides |

Protein Coverage:

1 MVLIRVLANL LILQLSYAQR SSELVAGGDE CNINEHRSLV VLFNSSGFLC GGTLINQEWV LTAAHCDSKN FQMQLGVHSK
81 KVLNDDEQTR DPKEKFLCLG RKNEK**DKDIM LIR**LNSPVHE STHIAPVSLP SSSPTVGSVC R**IMGWGLTTP TK**ETYPDVPN
161 CANINILDHA VCQRAYPGLL VKSRTLCAGI LKGGKDTCVG DSGGPLICNG QFQGIVSWGG HPCAEPLRPG VYTTVFDYND
241 WIKSIIAGNT AATCPP

Supporting Peptides:

Peptide	Uniq	-10lgP	Mass	Length	ppm	m/z	z	RT	Fraction	Scan	Source File	Area F1	Area F2	Area F3	Area F4
R.IMGWGLTTPTK.E	Y	45.69	1203.6322	11	0.2	602.8235	2	49.32	2	F2:14064	12112021_RID_2349_DP2.raw		1.254E5		
K.DKDIMLIR.L	N	44.22	1002.5532	8	-0.5	502.2836	2	37.38	3	F3:10341	17112021_RID_2349_DP3.raw			5.8616E6	
K.DKDIM(+15.99)LIR.L	N	39.37	1018.5481	8	0.3	510.2815	2	31.54	3	F3:8358	17112021_RID_2349_DP3.raw			1.7097E6	

total 3 peptides

TRINITY_DN1526
back to list

| Protein Coverage | Supporting Peptides |

Protein Coverage:

1 LGLSREQLQG GVSAKKGGRG RKGRREKGRG ECTKQTPTPG YLAVRAPVER RQTFSGSPGL HSRVMDPCRR KQIRWRTTLL
81 QQCVLLLTYV VIILEAVPID VDKTKVQEQE KDSATVDNPE TGLYYDAYLR QVIEVLETDA HFREKLQTTN VEDIKNGKLS
161 K**ELDLVGHK**V RTKLDELKRQ EVARLRMLIK AK**MDAYQDSG IDHQALLK**QF EHLNHHNPHT FEVKDLDMLI KAATHDLENY
241 DNTRHEEFKK YEMMKEHERR EYLKTLDEDK RRQEEAKYEE MKKKHNDHPK VNHPGSKDQL KEVWEEADGL DPNDFDAKTF
321 FKLHDVNADG FLDEQELEAL FTREVNMCNW IISESYFIWL SA

Supporting Peptides:

Peptide	Uniq	-10lgP	Mass	Length	ppm	m/z	z	RT	Fraction	Scan	Source File	Area F1	Area F2	Area F3	Area F4
K.MDAYQDSGIDHQALLK.Q	Y	59.48	1803.8461	16	1.3	902.9315	2	39.75	7	F7:11265	17112021_RID_2349_DP7.raw				
K.ELDLVGHK.V	Y	39.13	909.4919	8	0.8	455.7536	2	27.94	7	F7:7151	17112021_RID_2349_DP7.raw				

total 2 peptides

TRINITY_DN1526
back to list

| Protein Coverage | Supporting Peptides |

Protein Coverage:

1 LGLSREQLQG GVSAKKGGRG RKGRREKGRG ECTKQTPTPG YLAVRAPVER RQTFSGSPGL HSRVMDPCRR KQIRWRTTLL
81 QQCVLLLTYV VIILEAVPID VDKTKVQEQE KDSATVDNPE TGLYYDAYLR QVIEVLETDA HFREKLQTTN VEDIKNGKLS
161 K**ELDLVGHK**V RTKLDELKRQ EVARLRMLIK AK**MDAYQDSG IDHQALLK**QF EHLNHHNPHT FEVKDLDMLI KAATHDLENY
241 DNTRHEEFKK YEMMKEHERR EYLKTLDEDK RRQEEAKYEE MKKKHNDHPK VNHPGSKDQL KEVWEEADGL DPNDFDAKTF
321 FKLHDVNADG FLDEQELEAL FTRELEKVYD PKNEEDDMIE MEEERLRMRE HVMNEVDINK DRLITLEEFM RITEKREFSE
401 PESWEVQMAL LN

Supporting Peptides:

Peptide	Uniq	-10lgP	Mass	Length	ppm	m/z	z	RT	Fraction	Scan	Source File	Area F1	Area F2	Area F3	Area F4
K.MDAYQDSGIDHQALLK.Q	Y	59.48	1803.8461	16	1.3	902.9315	2	39.75	7	F7:11265	17112021_RID_2349_DP7.raw				
K.ELDLVGHK.V	Y	39.13	909.4919	8	0.8	455.7536	2	27.94	7	F7:7151	17112021_RID_2349_DP7.raw				

total 2 peptides

TRINITY_DN1526
back to list

| Protein Coverage | Supporting Peptides |

Protein Coverage:

1 LGLSREQLQG GVSAKKGGRG RKGRREKGRG ECTKQTPTPG YLAVRAPVER RQTFSGSPGL HSRVMDPCRR KQIRWRTTLL
81 QQCVLLLTYV VIILEAVPID VDKTKVQEQE KDSATVDNPE TGLYYDAYLR QVIEVLETDA HFREKLQTTN VEDIKNGKLS
161 K**ELDLVGHK**V RTKLDELKRQ EVARLRMLIK AK**MDAYQDSG IDHQALLK**QF EHLNHHNPHT FEVKDLDMLI KAATHDLENY
241 DNTRHEEFKK YEMMKEHERR EYLKTLDEDK RRQEEAKYEE MKKKHNDHPK VNHPGSKDQL KEVWEEADGL DPNDFDAKTF
321 FKLHDVNADG FLDEQELEAL FTRELEKVYD PKNEEDDMIE MEEERLRMRE HVMNEVDINK DRLITLEEFM RITEKREFSE
401 PESWETLDQQ EVFTEEELKQ FESHIFQQED ELQKKALELQ KKREELEQQQ DFLQAQKQEL EMAFKQMQQK KLQQGHPPSG
481 PGGELKFQPS VSQLDGNVQN HPVSSNQPLA PGNIPVQEAA ARSDHAQSHP

Supporting Peptides:

Peptide	Uniq	-10lgP	Mass	Length	ppm	m/z	z	RT	Fraction	Scan	Source File	Area F1	Area F2	Area F3	Area F4	Area /
K.MDAYQDSGIDHQALLK.Q	Y	59.48	1803.8461	16	1.3	902.9315	2	39.75	7	F7:11265	17112021_RID_2349_DP7.raw					
K.ELDLVGHK.V	Y	39.13	909.4919	8	0.8	455.7536	2	27.94	7	F7:7151	17112021_RID_2349_DP7.raw					

total 2 peptides

TRINITY_DN2452
back to list

| Protein Coverage | Supporting Peptides |

Protein Coverage:

1 LPDSEAHAIY KYENIEKEDE GPKKCGVTQT NWESDEPIKA SQFILTPEQR AYMNANKYIK LVIVVDNVMF R**KYTGDLIAI**
81 **R**TRIYEIVNT LNLIYTILNI HIALVY

Supporting Peptides:

Peptide	Uniq	-10lgP	Mass	Length	ppm	m/z	z	RT	Fraction	Scan	Source File	Area F1	Area F2	Area F3	Area F4	Ar
K.YTGDLIAIR.T	Y	54.91	1020.5604	9	0.2	511.2876	2	43.73	5	F5:12611	17112021_RID_2349_DP5.raw		3.2643E4		4.1422E4	3.8
R.KYTGDLIAIR.T	Y	45.94	1148.6553	10	0.5	575.3352	2	37.96	5	F5:10653	17112021_RID_2349_DP5.raw					1.57

total 2 peptides

P00990.1
back to list

| Protein Coverage | Supporting Peptides |

Protein Coverage:

1 HDRPTFCNLA PESGRCRGHL R**RIYYNLESN K**CKVFFYGGC GGNANNFETR DECRETCGGK

Supporting Peptides:

Peptide	Uniq	-10lgP	Mass	Length	ppm	m/z	z	RT	Fraction	Scan	Source File	Area F1	Area F2	Area F3	Area F4	Area
R.IYYNLESNK.C	Y	53.68	1142.5608	9	-0.1	572.2876	2	34.45	7	F7:9462	17112021_RID_2349_DP7.raw			4.065E5	8.0118E5	1.074
R.RIYYNLESNK.C	Y	46.56	1298.6619	10	0.4	650.3384	2	30.29	7	F7:8008	17112021_RID_2349_DP7.raw					

total 2 peptides

TIVRV2
back to list

| Protein Coverage | Supporting Peptides |

Protein Coverage:

1 HDRPTFCNLA PESGRCRGHL R**RIYYNLESN K**CKVFFYGGC GGNANNFETR DECRETCGGK

Supporting Peptides:

Peptide	Uniq	-10lgP	Mass	Length	ppm	m/z	z	RT	Fraction	Scan	Source File	Area F1	Area F2	Area F3	Area F4	Area
R.IYYNLESNK.C	Y	53.68	1142.5608	9	-0.1	572.2876	2	34.45	7	F7:9462	17112021_RID_2349_DP7.raw			4.065E5	8.0118E5	1.074
R.RIYYNLESNK.C	Y	46.56	1298.6619	10	0.4	650.3384	2	30.29	7	F7:8008	17112021_RID_2349_DP7.raw					

total 2 peptides

CAL69611.1
back to list

| Protein Coverage | Supporting Peptides |

Protein Coverage:

1 MSSGGLLLLL GLLTLWAELT PISGHDRPTF CNLAPESGRC RGHLR**RIYYN LESNK**CEVFF YGGCGGNDNN FSTRDECRHT
81 CVGK

Supporting Peptides:

Peptide	Uniq	-10lgP	Mass	Length	ppm	m/z	z	RT	Fraction	Scan	Source File	Area F1	Area F2	Area F3	Area F4	Area
R.IYYNLESNK.C	Y	53.68	1142.5608	9	-0.1	572.2876	2	34.45	7	F7:9462	17112021_RID_2349_DP7.raw			4.065E5	8.0118E5	1.074
R.RIYYNLESNK.C	Y	46.56	1298.6619	10	0.4	650.3384	2	30.29	7	F7:8008	17112021_RID_2349_DP7.raw					

total 2 peptides

A8Y7P3.1
back to list

| Protein Coverage | Supporting Peptides |
Protein Coverage:

1 MSSGGLLLLL GLLTLWAELT PISGHDRPTF CNLAPESGRC RGHLR**RIYYN LESNK**CEVFF YGGCGGNDNN FSTRDECRHT

81 CVGK

Supporting Peptides:

Peptide	Uniq	-10lgP	Mass	Length	ppm	m/z	z	RT	Fraction	Scan	Source File	Area F1	Area F2	Area F3	Area F4	Area
R.IYYNLESNK.C	Y	53.68	1142.5608	9	-0.1	572.2876	2	34.45	7	F7:9462	17112021_RID_2349_DP7.raw			4.065E5	8.0118E5	1.074
R.RIYYNLESNK.C	Y	46.56	1298.6619	10	0.4	650.3384	2	30.29	7	F7:8008	17112021_RID_2349_DP7.raw					

total 2 peptides

CAL69610.1
back to list

| Protein Coverage | Supporting Peptides |
Protein Coverage:

1 MSSGGLLLLL GLLTLWAELT PISGHDRPTF CNLAPESGRC RGHLR**RIYYN LESNK**CNVFF YGGCGGNDNN FETRDECRQT

81 CGGK

Supporting Peptides:

Peptide	Uniq	-10lgP	Mass	Length	ppm	m/z	z	RT	Fraction	Scan	Source File	Area F1	Area F2	Area F3	Area F4	Area
R.IYYNLESNK.C	Y	53.68	1142.5608	9	-0.1	572.2876	2	34.45	7	F7:9462	17112021_RID_2349_DP7.raw			4.065E5	8.0118E5	1.074
R.RIYYNLESNK.C	Y	46.56	1298.6619	10	0.4	650.3384	2	30.29	7	F7:8008	17112021_RID_2349_DP7.raw					

total 2 peptides

A8Y7P2.1
back to list

| Protein Coverage | Supporting Peptides |
Protein Coverage:

1 MSSGGLLLLL GLLTLWAELT PISGHDRPTF CNLAPESGRC RGHLR**RIYYN LESNK**CNVFF YGGCGGNDNN FETRDECRQT

81 CGGK

Supporting Peptides:

Peptide	Uniq	-10lgP	Mass	Length	ppm	m/z	z	RT	Fraction	Scan	Source File	Area F1	Area F2	Area F3	Area F4	Area
R.IYYNLESNK.C	Y	53.68	1142.5608	9	-0.1	572.2876	2	34.45	7	F7:9462	17112021_RID_2349_DP7.raw			4.065E5	8.0118E5	1.074
R.RIYYNLESNK.C	Y	46.56	1298.6619	10	0.4	650.3384	2	30.29	7	F7:8008	17112021_RID_2349_DP7.raw					

total 2 peptides

CAL69615.1
back to list

| Protein Coverage | Supporting Peptides |
Protein Coverage:

1 MSSGGLLLLL GLLTLWAELT PISGHDRPTF CNLAPESGRC RGHLR**RIYYN LESNK**CKVFF YGGCGGNANN FETRDECRQT

81 CGGK

Supporting Peptides:

Peptide	Uniq	-10lgP	Mass	Length	ppm	m/z	z	RT	Fraction	Scan	Source File	Area F1	Area F2	Area F3	Area F4	Area
R.IYYNLESNK.C	Y	53.68	1142.5608	9	-0.1	572.2876	2	34.45	7	F7:9462	17112021_RID_2349_DP7.raw			4.065E5	8.0118E5	1.074
R.RIYYNLESNK.C	Y	46.56	1298.6619	10	0.4	650.3384	2	30.29	7	F7:8008	17112021_RID_2349_DP7.raw					

total 2 peptides

AFE83617.1
back to list

| Protein Coverage | Supporting Peptides |

Protein Coverage:

1 MSSGGLLLLL GLLTLWAELT PISGHDRPTF CNLAPESGRC RGHLR**RIYYN LESNK**CKVFF YGGCGGNDNN FETRDECRQT

81 CGGK

Supporting Peptides:

Peptide	Uniq	-10lgP	Mass	Length	ppm	m/z	z	RT	Fraction	Scan	Source File	Area F1	Area F2	Area F3	Area F4	Area
R.IYYNLESNK.C	Y	53.68	1142.5608	9	-0.1	572.2876	2	34.45	7	F7:9462	17112021_RID_2349_DP7.raw			4.065E5	8.0118E5	1.074
R.RIYYNLESNK.C	Y	46.56	1298.6619	10	0.4	650.3384	2	30.29	7	F7:8008	17112021_RID_2349_DP7.raw					

total 2 peptides

A8Y7P4.1

back to list

| Protein Coverage | Supporting Peptides |

Protein Coverage:

1 MSSGGLLLLL GLLTLWAELT PISGHDRPTF CNLAPESGRC RGHLR**RIYYN LESNK**CEVFF YGGCGGNDNN FSTWDECRHT

81 CVGK

Supporting Peptides:

Peptide	Uniq	-10lgP	Mass	Length	ppm	m/z	z	RT	Fraction	Scan	Source File	Area F1	Area F2	Area F3	Area F4	Area
R.IYYNLESNK.C	Y	53.68	1142.5608	9	-0.1	572.2876	2	34.45	7	F7:9462	17112021_RID_2349_DP7.raw			4.065E5	8.0118E5	1.074
R.RIYYNLESNK.C	Y	46.56	1298.6619	10	0.4	650.3384	2	30.29	7	F7:8008	17112021_RID_2349_DP7.raw					

total 2 peptides

AFE83616.1

back to list

| Protein Coverage | Supporting Peptides |

Protein Coverage:

1 MSSGGLLLLL GLLTLWAELT PISGHDRPTF CNLAPESGRC RGHLR**RIYYN LESNK**CEVFF YGGCGGNDNN FSTWDECRHT

81 CVGK

Supporting Peptides:

Peptide	Uniq	-10lgP	Mass	Length	ppm	m/z	z	RT	Fraction	Scan	Source File	Area F1	Area F2	Area F3	Area F4	Area
R.IYYNLESNK.C	Y	53.68	1142.5608	9	-0.1	572.2876	2	34.45	7	F7:9462	17112021_RID_2349_DP7.raw			4.065E5	8.0118E5	1.074
R.RIYYNLESNK.C	Y	46.56	1298.6619	10	0.4	650.3384	2	30.29	7	F7:8008	17112021_RID_2349_DP7.raw					

total 2 peptides

AFB74192.1

back to list

| Protein Coverage | Supporting Peptides |

Protein Coverage:

1 MSSGGLLLLL GLLTLWAELT PISGHDRPTF CNLAPESGRC RAHLR**RIYYN LESNK**CEVFF YGGCGGNDNN FSTWDECRHT

81 CVGK

Supporting Peptides:

Peptide	Uniq	-10lgP	Mass	Length	ppm	m/z	z	RT	Fraction	Scan	Source File	Area F1	Area F2	Area F3	Area F4	Area
R.IYYNLESNK.C	Y	53.68	1142.5608	9	-0.1	572.2876	2	34.45	7	F7:9462	17112021_RID_2349_DP7.raw			4.065E5	8.0118E5	1.074
R.RIYYNLESNK.C	Y	46.56	1298.6619	10	0.4	650.3384	2	30.29	7	F7:8008	17112021_RID_2349_DP7.raw					

total 2 peptides

CAL69614.1

back to list

| Protein Coverage | Supporting Peptides |

Protein Coverage:

1 MSSGGLLLLL GLLTLWAELT PISSHDRPKF CNLAPESGRC RGHLR**RIYYN LESNK**CKVFF YGGCGGNANN FETRDECRQT

81 CGGK

Supporting Peptides:

Peptide	Uniq	-10lgP	Mass	Length	ppm	m/z	z	RT	Fraction	Scan	Source File	Area F1	Area F2	Area F3	Area F4	Area
R.IYYNLESNK.C	Y	53.68	1142.5608	9	-0.1	572.2876	2	34.45	7	F7:9462	17112021_RID_2349_DP7.raw			4.065E5	8.0118E5	1.074

total 2 peptides

Peptide	Uniq	-10lgP	Mass	Length	ppm	m/z	z	RT	Fraction	Scan	Source File	Area F1	Area F2	Area F3	Area F4	Area
R.RIYYNLESNK.C	Y	46.56	1298.6619	10	0.4	650.3384	2	30.29	7	F7:8008	17112021_RID_2349_DP7.raw					

total 2 peptides

A8Y7P6.1
back to list

| Protein Coverage | Supporting Peptides |
Protein Coverage:

1 MSSGGLLLLL GLLTLWAELT PISSHDRPKF CNLAPESGRC RGHLR**RIYYN LESNK**CKVFF YGGCGGNANN FETRDECRQT

81 CGGK

Supporting Peptides:

Peptide	Uniq	-10lgP	Mass	Length	ppm	m/z	z	RT	Fraction	Scan	Source File	Area F1	Area F2	Area F3	Area F4	Area
R.IYYNLESNK.C	Y	53.68	1142.5608	9	-0.1	572.2876	2	34.45	7	F7:9462	17112021_RID_2349_DP7.raw			4.065E5	8.0118E5	1.074
R.RIYYNLESNK.C	Y	46.56	1298.6619	10	0.4	650.3384	2	30.29	7	F7:8008	17112021_RID_2349_DP7.raw					

total 2 peptides

A8Y7N5.1
back to list

| Protein Coverage | Supporting Peptides |
Protein Coverage:

1 MSSGGLLLLL ALLTLWAELT PISGHDRPTF CNLAPESGRC RAHLR**RIYYN LESNK**CEVFF YGGCGGNDNN FSSWDECRHT

81 CVGK

Supporting Peptides:

Peptide	Uniq	-10lgP	Mass	Length	ppm	m/z	z	RT	Fraction	Scan	Source File	Area F1	Area F2	Area F3	Area F4	Area
R.IYYNLESNK.C	Y	53.68	1142.5608	9	-0.1	572.2876	2	34.45	7	F7:9462	17112021_RID_2349_DP7.raw			4.065E5	8.0118E5	1.074
R.RIYYNLESNK.C	Y	46.56	1298.6619	10	0.4	650.3384	2	30.29	7	F7:8008	17112021_RID_2349_DP7.raw					

total 2 peptides

CAL69608.1
back to list

| Protein Coverage | Supporting Peptides |
Protein Coverage:

1 MSSGGLLLLL GLLTLWAELT PISGHDRPTF CNLAPESGRC RGHLR**RIYYN LESNK**CKVFF YGGCGGNANN FETRDECRQT

81 CGGK

Supporting Peptides:

Peptide	Uniq	-10lgP	Mass	Length	ppm	m/z	z	RT	Fraction	Scan	Source File	Area F1	Area F2	Area F3	Area F4	Area
R.IYYNLESNK.C	Y	53.68	1142.5608	9	-0.1	572.2876	2	34.45	7	F7:9462	17112021_RID_2349_DP7.raw			4.065E5	8.0118E5	1.074
R.RIYYNLESNK.C	Y	46.56	1298.6619	10	0.4	650.3384	2	30.29	7	F7:8008	17112021_RID_2349_DP7.raw					

total 2 peptides

ABD24040.1
back to list

| Protein Coverage | Supporting Peptides |
Protein Coverage:

1 MSSGGLLLLL GLLTLWAELT PISGHDRPTF CNLAPESGRC RGHLR**RIYYN LESNK**CKVFF YGGCGGNANN FETRDECRQT

81 CGGK

Supporting Peptides:

Peptide	Uniq	-10lgP	Mass	Length	ppm	m/z	z	RT	Fraction	Scan	Source File	Area F1	Area F2	Area F3	Area F4	Area
R.IYYNLESNK.C	Y	53.68	1142.5608	9	-0.1	572.2876	2	34.45	7	F7:9462	17112021_RID_2349_DP7.raw			4.065E5	8.0118E5	1.074
R.RIYYNLESNK.C	Y	46.56	1298.6619	10	0.4	650.3384	2	30.29	7	F7:8008	17112021_RID_2349_DP7.raw					

total 2 peptides

CAL69612.1
back to list

| Protein Coverage | Supporting Peptides |
Protein Coverage:

1 MSSGGLLLLL GLLTLWAELT PISGHDRPTF CNLAPESGRC RGHLR**RIYYN LESNK**CEVFF YGGCGGNDNN FSTWDECRHT

81 CVGK

Supporting Peptides:

Peptide	Uniq	-10lgP	Mass	Length	ppm	m/z	z	RT	Fraction	Scan	Source File	Area F1	Area F2	Area F3	Area F4	Area
R.IYYNLESNK.C	Y	53.68	1142.5608	9	-0.1	572.2876	2	34.45	7	F7:9462	17112021_RID_2349_DP7.raw			4.065E5	8.0118E5	1.074
R.RIYYNLESNK.C	Y	46.56	1298.6619	10	0.4	650.3384	2	30.29	7	F7:8008	17112021_RID_2349_DP7.raw					

total 2 peptides

A8Y7P0.1
back to list

| Protein Coverage | Supporting Peptides |
Protein Coverage:

1 MSSGGLLLLL GLLTLWAELT PISGHDRPTF CNLAPESGRC RGHLR**RIYYN LESNK**CKVFF YGGCGGNANN FETRDECRQT

81 CGGK

Supporting Peptides:

Peptide	Uniq	-10lgP	Mass	Length	ppm	m/z	z	RT	Fraction	Scan	Source File	Area F1	Area F2	Area F3	Area F4	Area
R.IYYNLESNK.C	Y	53.68	1142.5608	9	-0.1	572.2876	2	34.45	7	F7:9462	17112021_RID_2349_DP7.raw			4.065E5	8.0118E5	1.074
R.RIYYNLESNK.C	Y	46.56	1298.6619	10	0.4	650.3384	2	30.29	7	F7:8008	17112021_RID_2349_DP7.raw					

total 2 peptides

AFE83615.1
back to list

| Protein Coverage | Supporting Peptides |
Protein Coverage:

1 MSSGGLLLLL GLLTLWAELT PISGHDRPTF CNLAPESGRC RGHLR**RIYYN LESNK**CKVFF YGGCGGNANN FETRDECRQT

81 CGGK

Supporting Peptides:

Peptide	Uniq	-10lgP	Mass	Length	ppm	m/z	z	RT	Fraction	Scan	Source File	Area F1	Area F2	Area F3	Area F4	Area
R.IYYNLESNK.C	Y	53.68	1142.5608	9	-0.1	572.2876	2	34.45	7	F7:9462	17112021_RID_2349_DP7.raw			4.065E5	8.0118E5	1.074
R.RIYYNLESNK.C	Y	46.56	1298.6619	10	0.4	650.3384	2	30.29	7	F7:8008	17112021_RID_2349_DP7.raw					

total 2 peptides

Q2ES50.1
back to list

| Protein Coverage | Supporting Peptides |
Protein Coverage:

1 MSSGGLLLLL GLLTLWAELT PISGHDRPTF CNLAPESGRC RGHLR**RIYYN LESNK**CKVFF YGGCGGNANN FETRDECRQT

81 CGGK

Supporting Peptides:

Peptide	Uniq	-10lgP	Mass	Length	ppm	m/z	z	RT	Fraction	Scan	Source File	Area F1	Area F2	Area F3	Area F4	Area
R.IYYNLESNK.C	Y	53.68	1142.5608	9	-0.1	572.2876	2	34.45	7	F7:9462	17112021_RID_2349_DP7.raw			4.065E5	8.0118E5	1.074
R.RIYYNLESNK.C	Y	46.56	1298.6619	10	0.4	650.3384	2	30.29	7	F7:8008	17112021_RID_2349_DP7.raw					

total 2 peptides

CAL69603.1
back to list

| Protein Coverage | Supporting Peptides |
Protein Coverage:

1 MSSGGLLLLL ALLTLWAELT PISGHDRPTF CNLAPESGRC RAHLR**RIYYN LESNK**CEVFF YGGCGGNDNN FSSWDECRHT

81 CVGK

Supporting Peptides:

Peptide	Uniq	-10lgP	Mass	Length	ppm	m/z	z	RT	Fraction	Scan	Source File	Area F1	Area F2	Area F3	Area F4	Area
R.IYYNLESNK.C	Y	53.68	1142.5608	9	-0.1	572.2876	2	34.45	7	F7:9462	17112021_RID_2349_DP7.raw			4.065E5	8.0118E5	1.074
R.RIYYNLESNK.C	Y	46.56	1298.6619	10	0.4	650.3384	2	30.29	7	F7:8008	17112021_RID_2349_DP7.raw					

total 2 peptides

H6VC06.1
back to list

| Protein Coverage | Supporting Peptides |
Protein Coverage:

1 MSSGGLLLLL GLLTLWAELT PISGHDRPTF CNLAPESGRC RAHLRRIYYN LESNKCEVFF YGGCGGNDNN FSTWDECRHT

81 CVGK

Supporting Peptides:

Peptide	Uniq	-10lgP	Mass	Length	ppm	m/z	z	RT	Fraction	Scan	Source File	Area F1	Area F2	Area F3	Area F4	Area
R.IYYNLESNK.C	Y	53.68	1142.5608	9	-0.1	572.2876	2	34.45	7	F7:9462	17112021_RID_2349_DP7.raw			4.065E5	8.0118E5	1.074
R.RIYYNLESNK.C	Y	46.56	1298.6619	10	0.4	650.3384	2	30.29	7	F7:8008	17112021_RID_2349_DP7.raw					

total 2 peptides

TRINITY_DN5102
back to list

| Protein Coverage | Supporting Peptides |
Protein Coverage:

1 MAPPALLCFG LGLCLCAAVA GSRRDSATGE QLFKAWASRH RRAYRSEGEF RHRMQIFLDN KQKIDKHNAG NYSFRMGLNQ

81 FSDMTFAEFK KTYLWQEPQN CSATTGNFPR SAGPCPRAID WRKKGRFVSP VKNQGPCGSC WTFSTTGCLE SAIAIKTGKL

161 LNLAEQQLID CAQNFNNFGC NGGLPSQAFE YILYNKGLMD EEAYPYRAQN GTCKFQPQKA VAFIKDVVNI SLYDEHGLMQ

241 AVGTYNPVSI AFEVKEDFVH YQEGVYTSTD CDKTPDKVNH AVLAVGYGEE DGVPFWIVKN SWGPSWGLDG YFNIERGKNT

321 CGLADCASFP VPLL

Supporting Peptides:

Peptide	Uniq	-10lgP	Mass	Length	ppm	m/z	z	RT	Fraction	Scan	Source File	Area F1	Area F2	Area F3	Area F4	Area F5	A
R.DSATGEQLFK.A	Y	60.36	1094.5244	10	-0.1	548.2694	2	37.40	2	F2:10112	12112021_RID_2349_DP2.raw		8.6583E4				

total 1 peptides

CAE47141.1
back to list

| Protein Coverage | Supporting Peptides |
Protein Coverage:

1 MRILWIVAVC LIGAEGHLSQ FGDMINKKTG IFGIMSYIYY GCYCGWGGKG KPLDATDRCC FVHDCCYGRV NGCDPKMGTY

81 SYSFQNGDIV CGGDDPCLRA VCECDRVAAI CFGENMNTYD RKYMLYSLFD CKEESEQC

Supporting Peptides:

Peptide	Uniq	-10lgP	Mass	Length	ppm	m/z	z	RT	Fraction	Scan	Source File	Area F1	Area F2	Area F3	Area F4	Area F5	Area F6
R.KYMLYSLFD.C	N	43.04	1178.5681	9	0.2	590.2914	2	60.72	7	F7:18383	17112021_RID_2349_DP7.raw						
G.ENMNTYDRK.Y	Y	39.03	1169.5135	9	-9.0	585.7588	2	23.22	7	F7:5585	17112021_RID_2349_DP7.raw						

total 2 peptides

ABB76279.1
back to list

| Protein Coverage | Supporting Peptides |
Protein Coverage:

1 MVLIRVLANL LILQLSYAQK SSELVIGGDE CNINEHRSLA LVYITSGFLC GGTLINQQWV LTAAHCDRGN MLIFFDVHSL

81 KGLNKDVQSR VAKEKFICPN RKKDDEKDKD IMLIKLDSPV SNSEHIAPLS LPSNPPSVGS VCRIMGWGAI TSPNVTLPGV

161 PHCANINILD YEVCRKAYTG LPATSRTLCA GILEGGKDSC KGDSGGPLIC NGQFQGIVSW GAHPCGQSLK PGVYTKVFDY

241 TEWIQSILAG NADATCPP

Supporting Peptides:

Peptide	Uniq	-10lgP	Mass	Length	ppm	m/z	z	RT	Fraction	Scan	Source File	Area F1	Ar F
K.VFDYTEWIQSILAGNADATC(+57.02)PP	Y	43.47	2467.1365	22	0.5	1234.5762	2	83.71	4	F4:25719	17112021_RID_2349_DP4.raw		

ADI47595.1
back to list

| Protein Coverage | Supporting Peptides |
Protein Coverage:

1 DLTAMRTRVY EMINTVNEIY IYMDIRVPLV GIEFWCNGDL ITVTSSAENT LNSFGDWRAS DLLSHKTHDN AQLLTTITLS
81 GSVVGLAHVS SMCQATRSVG VIEDHSPIVR EVAVTMAHEM GHNLGMSHDG NQCKCGDNSC IMYPYLSEPL PMFFSNCSKD
161 QYQKFLTNFK PDCTLIRPSR **TDIVSPPVCG NDLLER**GEEC DCGSPENCEN PCCDAASCKL HSWIECEFGE CCETCRLKPA
241 GTECRGIRNE CDLPEYCTGQ SGDCPTDHFH

Supporting Peptides:

Peptide	Uniq	-10lgP	Mass	Length	ppm	m/z	z	RT	Fraction	Scan	Source File	Area F1	Area A F2
R.TDIVSPPVC(+57.02)GNDLLER.G	Y	54.09	1783.8774	16	0.3	892.9462	2	47.68	1	F1:14421	12112021_RID_2349_DP1.raw	4.3022E6	

ADI47604.1
back to list

| Protein Coverage | Supporting Peptides |
Protein Coverage:

1 CIMYPYLSEP LPMFFSNCSK DQYQKFLTNF KPDCTLIRPS R**TDIVSPPVC GNDLLER**GEE CDCGSPENCE NPCCDAASCK
81 LHSWIECEFG ECCEQCRLKP AGTECRGIRN ECDLPEYCTG QSGDCPTDHF HRNGKPCLYN NGYCYNGACP IMEYQCYDHF
161 GPDAVVSQDA CFEKNKEGKG DFYCRNENYV KTPCAPEDVK CGRLFCDIGP NRCIFPYGDT GMVNPGTKCG DKKVCXHRKC
241 IDVNTTY

Supporting Peptides:

Peptide	Uniq	-10lgP	Mass	Length	ppm	m/z	z	RT	Fraction	Scan	Source File	Area F1	Area A F2
R.TDIVSPPVC(+57.02)GNDLLER.G	Y	54.09	1783.8774	16	0.3	892.9462	2	47.68	1	F1:14421	12112021_RID_2349_DP1.raw	4.3022E6	

TRINITY_DN722
back to list

| Protein Coverage | Supporting Peptides |
Protein Coverage:

1 MLLILALSLA HTYVVYGSIS CYDDSGKPVD WFIVYKLPKS HQSSLAEGMQ YMYQDGRTQG WVQGRSLMNS TEGAVGR**TLN**
81 **ELYLEAANR**Q REKEVAYVLY NDEPPQDSSS GSKGHTKGVI FLDRTQGFWL VHSTPRFPPK LDEKYAWPHT ALTYGQSFLC
161 VTYPYSQFKA IGNQLLFTEP HVYDYQVPVA FARDLPALLN ASEGQHVQEE PWNSRVTLTS KGGKEFVSFA KFRFFHDDLY
241 SGWLAQALAT DLYVEFWLKS HGVLASNCSG KYKVYNVEQL AFQDPATTFF STSDHSKWCV GIESDSEWAC VGDMNRNRAE
321 EHRGGGTICC RDVTIWKSFH SLVQNYRDCD TTGYGL

Supporting Peptides:

Peptide	Uniq	-10lgP	Mass	Length	ppm	m/z	z	RT	Fraction	Scan	Source File	Area F1	Area F2	Area F3	Area F4	Are F5
R.TLNELYLEAANR.Q	Y	72.61	1405.7201	12	0.9	703.8679	2	46.94	2	F2:13276	12112021_RID_2349_DP2.raw		3.0191E5			
													**total 1 peptides**			

TRINITY_DN722
back to list

| Protein Coverage | Supporting Peptides |
Protein Coverage:

1 MLLILALSLA HTYVVYGSIS CYDDSGKPVD WFIVYKLPKS HQSSLAEGMQ YMYQDGRTQG WVQGRSLMNS TEGAVGR**TLN**
81 **ELYLEAANR**Q VSLQKEQFQF LLVSKIVVMG

Supporting Peptides:

Peptide	Uniq	-10lgP	Mass	Length	ppm	m/z	z	RT	Fraction	Scan	Source File	Area F1	Area F2	Area F3	Area F4	Are F5
R.TLNELYLEAANR.Q	Y	72.61	1405.7201	12	0.9	703.8679	2	46.94	2	F2:13276	12112021_RID_2349_DP2.raw		3.0191E5			
													**total 1 peptides**			

TRINITY_DN722
back to list

| Protein Coverage | Supporting Peptides |
Protein Coverage:

1 FCKILMLLIL ALSLAHTYVV YGSISCYDDS GKPVDWFIVY KLPKSHQSSL AEGMQYMYQD GRTQGWVQGR SLMNSTEGAV
81 GR**TLNELYLE AANR**QREKEV AYVLYNDEPP QDSSSGSKGH TKGSALASNV FKERG

Supporting Peptides:

Peptide	Uniq	-10lgP	Mass	Length	ppm	m/z	z	RT	Fraction	Scan	Source File	Area F1	Area F2	Area F3	Area F4	Are F5
R.TLNELYLEAANR.Q	Y	72.61	1405.7201	12	0.9	703.8679	2	46.94	2	F2:13276	12112021_RID_2349_DP2.raw		3.0191E5			
													**total 1 peptides**			

TRINITY_DN86540
back to list

| Protein Coverage | Supporting Peptides |
Protein Coverage:

1 GGRQSERLGS PPEKARQRLY SPPREEGVCC FGRDGEGVLP SASPGLIKGQ PGCRGHEPAK MVLSEDDKSK VRASWSPVCK
81 NPELYGSETL TRMFAAHPTT KTYFPHFDLS PGSSDLKAHG KKVIDALTEA **VNNLDDVAGA LSK**LSDLHAH K**LRVDPVNFK**
161 LLAHCLEVTI AAHNGGPLKP EVILALDKFL CLVAKVLVSR YR

Supporting Peptides:

Peptide	Uniq	-10lgP	Mass	Length	ppm	m/z	z	RT	Fraction	Scan	Source File	Area F1	Area F2	Area F3	Area F4	Area
A.VNNLDDVAGALSK.L	Y	49.66	1314.6779	13	1.5	658.3472	2	43.46	7	F7:12535	17112021_RID_2349_DP7.raw					
K.LRVDPVNFK.L	N	42.14	1086.6185	9	0.0	544.3165	2	36.89	7	F7:10234	17112021_RID_2349_DP7.raw					4.637

total 2 peptides

P0C0U6.1
back to list

| Protein Coverage | Supporting Peptides |
Protein Coverage:

1 VLTEEDKSRV RAAWGPVSKN AELYGAETLT RLFTAYPATK TYFHHFDLSP GSSNLKTHGK KVIDAITEA**V NNLDDVAGAL**
81 **SK**LSDLHAQK **LRVDPVNFK**L LGHCLEVTIA AHNGGPLKPE VILSLDKFLC LVAKTLVSRY R

Supporting Peptides:

Peptide	Uniq	-10lgP	Mass	Length	ppm	m/z	z	RT	Fraction	Scan	Source File	Area F1	Area F2	Area F3	Area F4	Area
A.VNNLDDVAGALSK.L	Y	49.66	1314.6779	13	1.5	658.3472	2	43.46	7	F7:12535	17112021_RID_2349_DP7.raw					
K.LRVDPVNFK.L	N	42.14	1086.6185	9	0.0	544.3165	2	36.89	7	F7:10234	17112021_RID_2349_DP7.raw					4.637

total 2 peptides

XP_026532450.1
back to list

| Protein Coverage | Supporting Peptides |
Protein Coverage:

1 MVLTEEDKAR VRACWVPVSK NAELYGAETI TRLFAAHPTT KTYFPHFDVS PGSNDLKAHG KKVIDALTEA **VNNLDDVAGA**
81 **LSK**LSDLHAQ K**LRVDPVNFK** FLAHCLEVTI AAHNGGPLKP EVILSLDKFM DIVSKVLVSR YR

Supporting Peptides:

Peptide	Uniq	-10lgP	Mass	Length	ppm	m/z	z	RT	Fraction	Scan	Source File	Area F1	Area F2	Area F3	Area F4	Area
A.VNNLDDVAGALSK.L	Y	49.66	1314.6779	13	1.5	658.3472	2	43.46	7	F7:12535	17112021_RID_2349_DP7.raw					
K.LRVDPVNFK.F	N	42.14	1086.6185	9	0.0	544.3165	2	36.89	7	F7:10234	17112021_RID_2349_DP7.raw					4.637

total 2 peptides

XP_026570824.1
back to list

| Protein Coverage | Supporting Peptides |
Protein Coverage:

1 MVLTEEDKAR VRASWVPVSK NAELYGAETL TRLFSAHPTT KTYFPHFDLS PGSHDLKAHG KKVIDALTEA **VNNLDDVAGA**
81 **LSK**LSDLHAQ K**LRVDPVNFK** LLGQCLEVTI AAHNGGPLKP EVILSLDKFL DLASKLLVSR YR

Supporting Peptides:

Peptide	Uniq	-10lgP	Mass	Length	ppm	m/z	z	RT	Fraction	Scan	Source File	Area F1	Area F2	Area F3	Area F4	Area
A.VNNLDDVAGALSK.L	Y	49.66	1314.6779	13	1.5	658.3472	2	43.46	7	F7:12535	17112021_RID_2349_DP7.raw					
K.LRVDPVNFK.L	N	42.14	1086.6185	9	0.0	544.3165	2	36.89	7	F7:10234	17112021_RID_2349_DP7.raw					4.637

total 2 peptides

XP_034284997.1
back to list

| Protein Coverage | Supporting Peptides |
Protein Coverage:

1 MVLTEEDKAR VRASWGTVSK NAELYGSETL SRLFTVYPAT KTYFHHFDVS PGSSDLKSHG KKVIDAITEA **VNNLDDVAGA**
81 **LSK**LSDLHAQ K**LRVDPVNFK** LLAHCLEVTI AAHNGGPLKP EVILSVDKFL DKVAKVLVSR YR

Supporting Peptides:

Peptide	Uniq	-10lgP	Mass	Length	ppm	m/z	z	RT	Fraction	Scan	Source File	Area F1	Area F2	Area F3	Area F4	Area

total 2 peptides

Peptide	Uniq	-10lgP	Mass	Length	ppm	m/z	z	RT	Fraction	Scan	Source File	Area F1	Area F2	Area F3	Area F4	Area
A.VNNLDDVAGALSK.L	Y	49.66	1314.6779	13	1.5	658.3472	2	43.46	7	F7:12535	17112021_RID_2349_DP7.raw					
K.LRVDPVNFK.L	N	42.14	1086.6185	9	0.0	544.3165	2	36.89	7	F7:10234	17112021_RID_2349_DP7.raw					4.637

total 2 peptides

XP_032079120.1
back to list

| Protein Coverage | Supporting Peptides |
Protein Coverage:

1 MVLTEEDKAR VRACWGPVSK NAELYGSEAL TRLFAAHPAA KTYFHHFDVS PGSSDLKTHG KKVIDAITEA **VNNLDDVAGA**

81 **LSK**LSDLHAQ K**LRVDPVNFK** LLGHCLEVTI AAHNGGPLKA EVLLSLDKFL CKVAKVLVSR YR

Supporting Peptides:

Peptide	Uniq	-10lgP	Mass	Length	ppm	m/z	z	RT	Fraction	Scan	Source File	Area F1	Area F2	Area F3	Area F4	Area
A.VNNLDDVAGALSK.L	Y	49.66	1314.6779	13	1.5	658.3472	2	43.46	7	F7:12535	17112021_RID_2349_DP7.raw					
K.LRVDPVNFK.L	N	42.14	1086.6185	9	0.0	544.3165	2	36.89	7	F7:10234	17112021_RID_2349_DP7.raw					4.637

total 2 peptides

JAC95434.1
back to list

| Protein Coverage | Supporting Peptides |
Protein Coverage:

1 MVLTEEDKAR VRACWGPVCK NAEVYGAESL TRMFAAHPTT KTYFHHLDVS PGSNDLKTHG KKVIDAITEA **VNNLDDVAGA**

81 **LSK**LSDLHAQ K**LRVDPVNFK** LLAHCLEITI AAHNGGPLKP EVILSLDKFL CLVAKVLVSR YR

Supporting Peptides:

Peptide	Uniq	-10lgP	Mass	Length	ppm	m/z	z	RT	Fraction	Scan	Source File	Area F1	Area F2	Area F3	Area F4	Area
A.VNNLDDVAGALSK.L	Y	49.66	1314.6779	13	1.5	658.3472	2	43.46	7	F7:12535	17112021_RID_2349_DP7.raw					
K.LRVDPVNFK.L	N	42.14	1086.6185	9	0.0	544.3165	2	36.89	7	F7:10234	17112021_RID_2349_DP7.raw					4.637

total 2 peptides

LAA66838.1
back to list

| Protein Coverage | Supporting Peptides |
Protein Coverage:

1 VHGEGSPAVG GHQPAKMVLT DEDKARVRAC WGPVSKNAEL YGSEALTRLF AAYPTTKTYF HHFDLSPGSN DLKSHGKKVI

81 DALTEA**VNNL DDVAGALSK**L SDLHAQK**LRV DPVNFK**LLGH SLEVTIAAHN GGPLKAEVLL SLDKFLDRVA KVLVSRYR

Supporting Peptides:

Peptide	Uniq	-10lgP	Mass	Length	ppm	m/z	z	RT	Fraction	Scan	Source File	Area F1	Area F2	Area F3	Area F4	Area
A.VNNLDDVAGALSK.L	Y	49.66	1314.6779	13	1.5	658.3472	2	43.46	7	F7:12535	17112021_RID_2349_DP7.raw					
K.LRVDPVNFK.L	N	42.14	1086.6185	9	0.0	544.3165	2	36.89	7	F7:10234	17112021_RID_2349_DP7.raw					4.637

total 2 peptides

XP_029140879.1
back to list

| Protein Coverage | Supporting Peptides |
Protein Coverage:

1 MGHPQLWPLL LLLLLSNEAL TRDATRFKPF RKYIYHYETE ITSGVTGTAD SHSGSKIKCK AELEVPQLHN FVLKINQCTL
81 EEVYGIDVQG KALLKKSKNS DEFAAAMSQY ELKVKIQNEK NVELYPVDSE ATHILNIKRG IISALLVPTE REDNIQTLSM
161 DTVYGKCDSS IEVKNRKTTA NLADIEITRN LKSCENFNPI RDYVSPIAFI KGLNAPLSTL ISSNQHCHYT VDTKKKHISE
241 AICTEKHLFL PSSYKNQYGM MAKVIQTLKY ESIANSNTRS MDVTGLIKRE LSLENVEAKI LRHGDAVLKS LQELKKLSVS
321 QQNHKRASLF YRFVTGLRSL HNSTLGPMVP KLMETSSSIT IQALIQCGTP ECFGAILQIL RTSNIDPLVA DAVTFSLGLL
401 PSPCTKTIRE ILNMAEYHPS RASFYALSHS VNRFYSQKKI ITPELTDVAN FVTSLISNEC SGNEDLTYLT LRVIGNMGNI
481 LENVNPNVKQ SLKLCIRSNA ASLDIQKAAI QALRKMDLND EDRGMLIRVF QEASSPADKR LAAYLLLMKN PSTSDLSKII
561 KVSLRDKNEQ VKNFVASHIA NILDTIDFSN EPYLPMLRIK IQEALKGNQM PTVKDFRRFS QNYQVSKTMT VPWSQDPFTA
641 KLEGNLLFDP SSYIAKEAML KTTLELFGLR PLDVFEIGLD GKNLEPTVEA MFGPEGFFPD AATKALYWID GKVPEQFSQA
721 LFNYFGYSKD ATQDQDIMKG IMLNFEKLVK EISNKPTPDV KAYFRLFQEE LGYLKFSDIK LLGNVILKSF KTLQSIPQKI
801 VQTISQGTEN DLMIHYIFMD NEFELPTSAG LQLQVALSGV ATPGAKVGMK ISQRNIHADL IVKPSMAVEF ITHLGVNIPE
881 FARNGVQMNT NIYHESGIEA NVALKAGHLK FTIPAPKAPI KLFSISNVLH LVTPTRNEVI PPLMENRVTQ NNCSPFFTGL
961 KFCTIIEYSN ASSTVAAPYY PLTGETRYEI ELQPTGRVKE YIASANYELN KEGKDLVDTL KFIAQAKGVK ESEAKIVFTF
1041 NRGKKIFISD FQAPGFNINF GTNLKITDSS NQEKKAVAFI LDILNEK**SPE ITITGR**LSSA FGRESSLEAA ISIPRLQTQA
1121 KTDISLRRSS NAMIFQFDSS ATSYGSSVSE RIVLRYDTEK VELQWNSGTS AALKRICANL PGDLEGYSKA LQNKANGLLN
1201 HKVANTDMTL RHIVSHFIVA TNTWLQKTSK DIPYAQNLQD KLSKLQQLDF QIVDVITIPE ELFFKSDGKI KYVWNKESTF
1281 ITIPMPFGGQ SSQVISIPKI IRAPAMVVES LGLNIPSQEY KIPQFTIPND YTLRLPLLGT FEITSNLYNN YYNWSGSYSL
1361 ANTTKDAYGL KTRYFMKADS VLDLLSYNVQ GQAEASYDGN GLTCDYENSL RHSLLNSNFK FSKVNRYGLN PTVKYSMTFD
1441 TSSPVGVMFS LSAHQDSTED DNSHVGVTNL EGQLNVATLF AKTTYLQSIT SKKKSLDLVW DSTLKFDSSL FGATNQMIQK
1521 ITEDAWTINS ITNIQNYILT NKVSLRYQNS QLKLSSEAIG TYTNFETINK FDLTLDKQQA ILHSKLKATY FKNVYYALVS
1601 GSVNNQGVEL IGDLSWENER NKAEHKSALS IKQDGLVSSA TTKLQLLLLI LENEMNARIG TSGASVKIDT NGRAGTHNAR
1681 FSLDGKLALT EIALLSTYQG SILEVDSKNI LNFRVNNGEL RFSNSLTGSH KEMQFEHTHE LNIVGLSLTY VSNLDHTINP
1761 DVSHKHHVDL QLQPSSLTAN LNNDLKYSSA NVNNKARLQL ELLKVNLDGN IKGAYSGNEI KHTYIFTYGD LAAHMKTDTA
1841 ASIQGAAVTH KVNLNVAGLS STINVNTNCD SKFLTFSNEI RSMAAPFTVT FDMHTNGDGQ LGEQSGRLYS KFLLKAEPLA
1921 IVFSHDYQGS TNHNINSGER YNTGLDSKVT VVLTPSEQSS AWKMKSQLNN HVHTQDINMF NNAENIGLEL TGQTLADLSA
2001 LDFPMKIPFT NDRRLNLIDT LDLRENIAEP QEFGLSLSLK YDKNADMHVI NLPFLERLPA YYEQFRRSIL TAMKSIQKNL
2081 KNINIDQLMR KYKIALDKFP QQVNDYVNSF DLERKVNKLK ENLDAFTKDY SITVEDLQNT LENAKANLLK ALTQLQVFLT
2161 ETEKYMKENY DLRPAIMQLI EKIVEKLKVL DDQYEISVTM INTIQQLQSF ISQYDPSEIG SNAAAWVQNV DTTYKIRAQI
2241 QEKLDQLKSQ IQNFDAQHIA ETLKQQIETI DTKILIEKLK IQFPFEKINQ LLEQIKNILL NFMEDYEISE KINAFRVRMH
2321 ELIVKYNIDE QAKVLMDKVI ELYKQQKITE TVLRLTNTLK QIDIKPFFNQ ILKFIDDAIK KLQTVDYKKL VDGINDFLDI
2401 MIKKLKSFDY NKFVDETNNK IRQATQKINE EIKALELPQK IEAAKQYIKE VYTVVSRYIE KLKDSRLAAT VNWLNDLISS
2481 TAIDELKSKI RDFLEDARKR IYEMDIPAEC QRFLHKASQL YRVIVSFLVD KWDIISKKIS VSAEKYNVKN MADHLNQFVK
2561 TGFIVPAIKL GVINIPAFEV SLRALQDATF KTPDFILPLT DLHVPSYQIN FKKLNEIRIP IRFSTPEFTI LNTIKVPSYT
2641 IDFNEIKLKI VRIIDQIMTS DFQLPATDTY FQEIKIKDLY FSDFSLPEIN LPQLQISELL IPKLNLNEFQ FPDIKIPEFQ
2721 LPRIPHTVTI PTFGKLSGTC RITSPFFTLT TNAGFQNVTA HPYSPEFVAS LSAIAVSKID YLAFTLTANT HLLAPELKKL
2801 ILKENLKFTH LYLKADHGSE VVFLTASVQG NAETTASFRS TRNAIELHNK LTVNLNKIIS MDSKTLYTHR LNIPKAQFTS
2881 QAELSNEIKT ALEAGHISVT STGKGDWKWT AHDYSDEGTH ESKVSFTIER SIAAFIAENR INAKYLRVNQ RMVYEYNLPS
2961 SSKLQVESTI ESPQLGQSVL NVQGTGNLVQ LKMELSGTHN AKVIGRTSGT INNDVSFSVQ PFEIKASANN NMNLKVSFPF
3041 KIVGKIESLN NYGLVLSPST QQVSWVAEGR FNQYRYTHNL SAGNNEDSTE AIISMNGEAN LEFLNIPISF PEQFVPYLGI
3121 KTPQVQAYSL WEETGLKGLL KTPKQSFDLN VKLQYKKNKD VHSFPIPLDR VHKTLNSYIV NFNKYFERNR DHTLAFLTES
3201 YNQAKVKFDK YKVDTSVNQS PQIFTIPSYT IPLLNIEVSP FTAELPTFGY VLPKEMRTPD FTVPFVQYSM PSYTVVLPSL
3281 ELPVLHIPQG LRTLKLPNYR VSPLPNQIYI PAFGNVTYDF SFKSSVITLN TNAGFFNQSD IIARLSSSST SVIEALQYQL
3361 TGTTSITRKR GLKLATALSL KNKFVEGNHD STISFTKKNI EALVVTTAKI MAPGLKVNYG QELKGNAKTM PIISSKINLN
3441 YDVDTNYVNA KGAIVHQANL ESIISYISAD TSTSGFINGD FYEGKPFSGK LVHEANTYLN SKGIRSAMKF ETSSNSGDFW
3521 NFDAKENVAI EASTNRLYAL WDHSGENYLN YFPLPRTRGN QICRVTLELA SDIMSAALQI QVAQPNNFLE ETSVNQAFSL
3601 TVNNGNQKIN WKSESQIHNF FLGHDLELAN DNVEARVGLS ASLGGHINFL REFTLPVYER SLWHILKLDL TTRPEEKQFL
3681 NASTNIVYTK NQDGYFFPIS ISQLADRFII KFPAIKAVGI HVQFFTIGLH EFNVLERVNN TSFDINLPLL PKMQLDEITK
3761 YNNLGQNKIP YFELTVPKHP KLVTIPQFTL PKRLFLGNIN IDLDVVANKI ADFDLPTITI PQQQIEIPVF TFTLPAGIYF
3841 PK**FGALSSSF K**IASPLYSAT WKTDLKNTKE AFEHSIDFTA NSPLQFLEYD LDGVTTYQYE GNRFTLNRQY DLAHPELSGK
3921 YKAVYITEGD RIVRDKASLD ITSPTFTDVQ MRYQGDDSRV STMISSSSAG TLRFLISLDS SILNSKIFYQ TQSNPQNEVE
4001 ILKSEISFKN PELIQIKTNW KEDAVTELLE GLKEKVPKMA DALYYTANKY HREHTGMEIS SAASKLKDTL RSNIDQTYVN
4081 TVNSISELEQ QLHEVTNQVT GKYEQIKTES QKLYHKAADQ ASQIEYDQIR TQFLDAIMDS IVEYHKQIKR LIDSAIEFLK
4161 VTRFQVPGLP GKHTGEELYV IITEKVAKAV DQSIARVQEY FDSLITFVNE AEIKIPASSE IIKGSDILAE IKTFLTHVQK
4241 KVSQIFVGLR EIDFAQHLRD LKERIQQVFQ KIEELIKNLQ AQNYDHLKEQ TKQLFVNFLN ALNSLAGDVK YFSPRIENII
4321 QNTFSEIYNK LEELLHSIRD LRKEYFDPNV VGWSLKYYEV EEKFINWLKT FFNTILEWHI KWINEAADLI TNLTDQAKEA
4401 LENQEKLAEF SKTAHEKIQY WSKVAKQSAA EQNQQVKAKL QETYNHLFNS YEMLISETKR LIDLTIEKYT TFFQYLQKLL
4481 DWLEKVTADA LRPYIAVRQG ELRIDIPKPF NLSAIYQGPL SDEDLQQQQT ILANQ

Supporting Peptides:

Peptide	Uniq	-10lgP	Mass	Length	ppm	m/z	z	RT	Fraction	Scan	Source File	Area F1	Area F2	Area F3	Area F4	Area F5	Area F6	Are
K.SPEITITGR.L	Y	49.73	972.5240	9	0.1	487.2693	2	33.18	7	F7:9009	17112021_RID_2349_DP7.raw							1.38
K.FGALSSSFK.I	Y	40.73	942.4810	9	-0.4	472.2476	2	38.95	7	F7:10968	17112021_RID_2349_DP7.raw							5.80

total 2 peptides

AJO70722.1
back to list

| Protein Coverage | Supporting Peptides |

Protein Coverage:

1 MGRFISVSFG CLVVFLSLSG TGADQDCPSD WSSHEGHCYK VFNLRMNWAD AEKFCTEVVS GGHLISLNSA EEVDFMIKLV

81 FPILK**FDFIW IGLR**DFWRDC HWGWSDGVKL DYKAWSDEPN CYVAKTVDYQ WLFRDCNRTS RFICKSRVPR

Supporting Peptides:

Peptide	Uniq	-10lgP	Mass	Length	ppm	m/z	z	RT	Fraction	Scan	Source File	Area F1	Area F2	Area F3	Area F4	Area F5	Area F6	A
K.FDFIWIGLR.D	Y	51.10	1165.6284	9	0.3	583.8217	2	71.27	7	F7:21945	17112021_RID_2349_DP7.raw							0

total 1 peptides

XP_029142225.1
back to list

| Protein Coverage | Supporting Peptides |

Protein Coverage:

1 MSEILMGVFG IWTKSSIFML SCLIEGGKNC NGPPRIENGD IITLSEKRYR SGSSVEFRCQ RFYAMEGQKI SFCNNGTWTK

81 VPICLDPCRI PTAELERQKI EVKDGMDASE NIFVQHGHSI ELICRTGYVL AADSSQSASI IHCDGTTPVI PKCKEIICNS

161 PRISNGSFRP KRNIYHVGDL IQIQCYSGFT FEPDNRGQVV ECTKNGWSPP SKCVLVNPLI VCEEPDDIAF GEIVTMEKAK

241 YLENDRVQYR CYPGYVLEGL EWIQCKGQQW TPRPPKCLAP CSITRQQLVT KNLFVFGSQR KSRFIQSNHS LQFQCSEGYV

321 LVVPSVRKCV DGYMDLPLCI SERGKNCSDP PR**IENGDITT LSEK**QYRSGS SVEFRCQRYY TIEGQNRSFC DNGTWTKVPI

401 CLDPCMIPRA ELETQKIEVK GGMDASENIF VPRGHSIELT CRTGYVLAAD SSQLACDGTP PVIPKCKEIT CSSPRILKGS

481 FRPQRNIYND GDLIRIQCDN GFTLEQDNGG QVAECTRNGW LPSPKCVQVE RTCKVSLLHG SFVPEQQSIQ INDKAKYICQ

561 DGYTTSKGET EVETQCLTEG WSPEPECIRT CLKPPADNFI FNTTKPVFLP GDVLHYECKE EFEITKNSIS DTVVCTEEGW

641 EPTPPRCVAF VCQAPFLENG AIDPRENVFQ HKMVVRFNCD TGFTRVGSES AQCYHFGWSP QPPVCKGIAS VCHAPFLENG

721 AINPREDVFQ HKMVVRFNCD RGFIRVGSES AQCYHFGWSP QPPVCKENVK SCQASPRISH GMVTDERKAV HQHGDLLEVQ

801 CDISFALRGS KTIECVDGEW APLPSCIEEV KTCGPPRKIT RGIPVDAMSS IYRHGETVAY QCQQPSVSIG TNPAKCLHGQ

881 WELPSCLETC PPPPQLPNAI NIAEMRIYRS EEEISFKCQG NFLLRGPPKI KCEDGKWQTP PRCLACEEPE DIDFGEIVTT

961 EKAKYLENER VQYRCNPAYV LEGPEWIQCK GQKWTPHPPK CLAPCSITRQ QLATKNLFVF GSQRKALLIQ SNHSRQFQCN

1041 EGYVLVAPSV RMCVDGYMEL PLCISERGRN CSGPPRIENG DITTLSEKRY RSGSSVEFRC QKDYAMEDQN RSFCDNGTWT

1121 KVPICLDPCV IPRAELESQM IELKDGIDVP ENTFVQRGHS IELTCRTGYV LAVDSSQSAS IIHCDGTTPV IPKCKEITCN

1201 SPRISNGSFL PQRTIYHDGD LIRIRCESGF TFKPDNGEKV VECTKNGWSP PPKCVSRGSQ PPAMAHYHAV ACLKPGHVSG

1281 WPVHTCVRAA QVA

Supporting Peptides:

Peptide	Uniq	-10lgP	Mass	Length	ppm	m/z	z	RT	Fraction	Scan	Source File	Area F1	Area F2	Area F3	Area F4	Area F5
R.IENGDITTLSEK.Q	Y	64.45	1318.6616	12	0.5	660.3384	2	34.80	3	F3:9504	17112021_RID_2349_DP3.raw			9.5811E4		

total 1 peptides

XP_015677107.1
back to list

| Protein Coverage | Supporting Peptides |

Protein Coverage:

1 MKSWLLLVVV FLLWPGCSSQ NVCEEPDDID FGEIVTTEKA KYLENDRVQY RCYPGYVLEG PEWIQCKGQQ WTPHPPKCLA

81 PCIITRQQLA TKNLFVFGGQ RKTWIIQNNH SVQFQCNERH VLVVPSVRKC VDGYMDLPLC LSERGGNCSG PPR**IENGDIT**

161 **TLSEK**RYRSG SSVEFKCQKY YAMEGQKRSF CDNGTWTKVP ICLDPCMIPT AELERQKIEV KDGMDASENI FVQRGHSIEL

241 ICRTGYVLAA DSSQSASIIH CDGTPPVIPK CKEITCNSPR ILNGSFRSQR TIYHDGDLIR IQCDSGFTLE PDNGEKVVEC

321 TRNGWSPSPK CVEIQCSPPE VRKGRIEPRR SQYMYNDEIE IICHRRIFFT AIQRKYISKC TANGWSPSPL CEEIRCSPPE

401 VRNGYIRPTQ SQYMHNDEIE ITCFRWPFFK GDRKSRSNCT ANGWDPPPSC

Supporting Peptides:

Peptide	Uniq	-10lgP	Mass	Length	ppm	m/z	z	RT	Fraction	Scan	Source File	Area F1	Area F2	Area F3	Area F4	Area F5
R.IENGDITTLSEK.R	Y	64.45	1318.6616	12	0.5	660.3384	2	34.80	3	F3:9504	17112021_RID_2349_DP3.raw			9.5811E4		

total 1 peptides

TRINITY_DN1914
back to list

| Protein Coverage | Supporting Peptides |
Protein Coverage:

```
  1 MKNWLLLVAA FLLWPCCSSQ NACEEPEDID FGEIVSIEKA KYLENDRVQY RCNPAYVLEG PEWIQCKGQK WTPHPPKCLA
 81 PCSITRQQLT TKNLFVFGSQ RKARLIQSNH SLQFQCNEGY VLVAPSVRKC VDGYMEFPLC ISERAGNCRG PPRIENGDIT
161 TLSEKQYRSG SSVEFRCQKH YAMEGQNRSF CDNGTWTKVP ICLDPCVIPR VELESQMIEL EDGIDVPENI FVQRGHSIEL
241 TCRTGYVLAV NSSQSASIIH CDGTRPVIPK CKEITCHSPR ISNGSFRPQR TIYHDGDLIR IQCDSGFTFE RDNGEEVVEC
321 TKNGWSPPPK CVTACTTSEE DMREHNIKLK WAIGNKIYST DGNIIEFQCI RGHKLHPSYR TLRINCVNGK FDYPRCIPI
```

Supporting Peptides:

Peptide	Uniq	-10lgP	Mass	Length	ppm	m/z	z	RT	Fraction	Scan	Source File	Area F1	Area F2	Area F3	Area F4	Area F5
R.IENGDITTLSEK.Q	Y	64.45	1318.6616	12	0.5	660.3384	2	34.80	3	F3:9504	17112021_RID_2349_DP3.raw			9.5811E4		

total 1 peptides

TRINITY_DN1889
back to list

| Protein Coverage | Supporting Peptides |
Protein Coverage:

```
  1 MFRCRLLLCL AGLLVLSEAG GRLPALQAAL EQVLEDFHGR SLVQAAFLGE TARELVEVLP MGTYVQLEVD LVQTVCRKHQ
 81 WRSQNCQIKA GGRRQKCLAC FKFDDNNNPG RLLNKSLRCL SEQNPLFQEA RRRQEAECQA IKAANEDRYL PGKFAFSVGL
161 PS
```

Supporting Peptides:

Peptide	Uniq	-10lgP	Mass	Length	ppm	m/z	z	RT	Fraction	Scan	Source File	Area F1	Area F2	Area F3	Area F4	Area F5
R.SLVQAAFLGETAR.E	Y	63.54	1361.7302	13	1.1	681.8732	2	50.30	7	F7:14849	17112021_RID_2349_DP7.raw					

total 1 peptides

CCH15044.1
back to list

| Protein Coverage | Supporting Peptides |
Protein Coverage:

```
  1 MSSGGLLLLL GLLTLWAELT PVSSQDRPKF CYLPADPAEC NAYMPRFYYD SASNKCKEFI YGGCRGNANN FKNRAECRHT
 81 CVASRKGIQP RIASN
```

Supporting Peptides:

Peptide	Uniq	-10lgP	Mass	Length	ppm	m/z	z	RT	Fraction	Scan	Source File	Area F1	Area F2	Area F3	Area
R.FYYDSASNK.C	Y	63.13	1093.4716	9	0.3	547.7432	2	28.93	5	F5:7517	17112021_RID_2349_DP5.raw	8.2206E5	5.5535E5	2.9889E6	2.3037

total 1 peptides

I2G9B4.1
back to list

| Protein Coverage | Supporting Peptides |
Protein Coverage:

```
  1 MSSGGLLLLL GLLTLWAELT PVSSQDRPKF CYLPADPAEC NAYMPRFYYD SASNKCKEFI YGGCRGNANN FKNRAECRHT
 81 CVASRKGIQP RIASN
```

Supporting Peptides:

Peptide	Uniq	-10lgP	Mass	Length	ppm	m/z	z	RT	Fraction	Scan	Source File	Area F1	Area F2	Area F3	Area
R.FYYDSASNK.C	Y	63.13	1093.4716	9	0.3	547.7432	2	28.93	5	F5:7517	17112021_RID_2349_DP5.raw	8.2206E5	5.5535E5	2.9889E6	2.3037

total 1 peptides

AMH40736.1
back to list

| Protein Coverage | Supporting Peptides |
Protein Coverage:

```
  1 MSSGGLLLLL GLLTLWAELT PISGRDRPKF CHLPADPGIC KAHTPRFYYD SASNKCKQFS FGGCPGNANN SKTWDECRHT
 81 CVASGKGIQP
```

Supporting Peptides:

Peptide	Uniq	-10lgP	Mass	Length	ppm	m/z	z	RT	Fraction	Scan	Source File	Area F1	Area F2	Area F3	Area
R.FYYDSASNK.C	Y	63.13	1093.4716	9	0.3	547.7432	2	28.93	5	F5:7517	17112021_RID_2349_DP5.raw	8.2206E5	5.5535E5	2.9889E6	2.3037

total 1 peptides

AMH40741.1
back to list

| Protein Coverage | Supporting Peptides |

Protein Coverage:

1 MSSGGLLLLL GLLTLWAELT PVSGQDHPKF CYLPADPGRC KAHIPR**FYYD SASNK**CNKFI YGGCPGNANN FKTWDECRQT

81 CGASAMGRPT

Supporting Peptides:

Peptide	Uniq	-10lgP	Mass	Length	ppm	m/z	z	RT	Fraction	Scan	Source File	Area F1	Area F2	Area F3	Area I
R.FYYDSASNK.C	Y	63.13	1093.4716	9	0.3	547.7432	2	28.93	5	F5:7517	17112021_RID_2349_DP5.raw	8.2206E5	5.5535E5	2.9889E6	2.3037

total 1 peptides

P00991.2

back to list

| Protein Coverage | Supporting Peptides |

Protein Coverage:

1 MSSGGLLLLL GLLTLWAELT PVSGQDHPKF CYLPADPGRC KAHIPR**FYYD SASNK**CNKFI YGGCPGNANN FKTWDECRQT

81 CGASAMGRPT

Supporting Peptides:

Peptide	Uniq	-10lgP	Mass	Length	ppm	m/z	z	RT	Fraction	Scan	Source File	Area F1	Area F2	Area F3	Area I
R.FYYDSASNK.C	Y	63.13	1093.4716	9	0.3	547.7432	2	28.93	5	F5:7517	17112021_RID_2349_DP5.raw	8.2206E5	5.5535E5	2.9889E6	2.3037

total 1 peptides

AAP04484.1

back to list

| Protein Coverage | Supporting Peptides |

Protein Coverage:

1 MSSGGLLLLL GLLTLWAELT PVSGQDHPKF CYLPADPGRC KAHIPR**FYYD SASNK**CNKFI YGGCPGNANN FKTWDECRQT

81 CGASAMGRPT

Supporting Peptides:

Peptide	Uniq	-10lgP	Mass	Length	ppm	m/z	z	RT	Fraction	Scan	Source File	Area F1	Area F2	Area F3	Area I
R.FYYDSASNK.C	Y	63.13	1093.4716	9	0.3	547.7432	2	28.93	5	F5:7517	17112021_RID_2349_DP5.raw	8.2206E5	5.5535E5	2.9889E6	2.3037

total 1 peptides

AAP04485.1

back to list

| Protein Coverage | Supporting Peptides |

Protein Coverage:

1 MSSGGLLLLL GLLTLWAELT PVSTRDRPKF CYLPADPGRC LAYMPS**FYYD SASNK**CKKFI YGGCRGNANN FKTWDECRHT

81 CVASGIQPRI ASN

Supporting Peptides:

Peptide	Uniq	-10lgP	Mass	Length	ppm	m/z	z	RT	Fraction	Scan	Source File	Area F1	Area F2	Area F3	Area I
S.FYYDSASNK.C	Y	63.13	1093.4716	9	0.3	547.7432	2	28.93	5	F5:7517	17112021_RID_2349_DP5.raw	8.2206E5	5.5535E5	2.9889E6	2.3037

total 1 peptides

P00992.2

back to list

| Protein Coverage | Supporting Peptides |

Protein Coverage:

1 MSSGGLLLLL GLLTLWAELT PVSTRDRPKF CYLPADPGRC LAYMPS**FYYD SASNK**CKKFI YGGCRGNANN FKTWDECRHT

81 CVASGIQPRI ASN

Supporting Peptides:

Peptide	Uniq	-10lgP	Mass	Length	ppm	m/z	z	RT	Fraction	Scan	Source File	Area F1	Area F2	Area F3	Area I
S.FYYDSASNK.C	Y	63.13	1093.4716	9	0.3	547.7432	2	28.93	5	F5:7517	17112021_RID_2349_DP5.raw	8.2206E5	5.5535E5	2.9889E6	2.3037

total 1 peptides

AMH40733.1

back to list

| Protein Coverage | Supporting Peptides |

Protein Coverage:

1 MSSGGLLLLL GLLTLWAELT PVSTRDRPKF CYLPADPGRC LAYIPS**FYYD SASNK**CKKFI YGGCRGNANN FKTWDECRHT

81 CVASGIQPRI ASN

Supporting Peptides:

Peptide	Uniq	-10lgP	Mass	Length	ppm	m/z	z	RT	Fraction	Scan	Source File	Area F1	Area F2	Area F3	Area I

total 1 peptides

Peptide	Uniq	-10lgP	Mass	Length	ppm	m/z	z	RT	Fraction	Scan	Source File	Area F1	Area F2	Area F3	Area I
S.FYYDSASNK.C	Y	63.13	1093.4716	9	0.3	547.7432	2	28.93	5	F5:7517	17112021_RID_2349_DP5.raw	8.2206E5	5.5535E5	2.9889E6	2.3037I

total 1 peptides

AMH40738.1
back to list

| Protein Coverage | Supporting Peptides |
Protein Coverage:

1 MSSGGLLLLL GLLTLWAELT PVSTRDRPKF CYLPADPGRC LAYIPS**FYYD SASNK**CKKFI YGGCRGNANN FKTWDECRHT

81 CVASGIQPRI ASN

Supporting Peptides:

Peptide	Uniq	-10lgP	Mass	Length	ppm	m/z	z	RT	Fraction	Scan	Source File	Area F1	Area F2	Area F3	Area I
S.FYYDSASNK.C	Y	63.13	1093.4716	9	0.3	547.7432	2	28.93	5	F5:7517	17112021_RID_2349_DP5.raw	8.2206E5	5.5535E5	2.9889E6	2.3037I

total 1 peptides

AMH40740.1
back to list

| Protein Coverage | Supporting Peptides |
Protein Coverage:

1 MSSGGLLLLL GLLTLWAELT PVSTRDRPKF CYLPADPGRC LAYIPS**FYYD SASNK**CKKFI YGGCRGNANN FKTWDECRHT

81 CVASGIQPRI ASN

Supporting Peptides:

Peptide	Uniq	-10lgP	Mass	Length	ppm	m/z	z	RT	Fraction	Scan	Source File	Area F1	Area F2	Area F3	Area I
S.FYYDSASNK.C	Y	63.13	1093.4716	9	0.3	547.7432	2	28.93	5	F5:7517	17112021_RID_2349_DP5.raw	8.2206E5	5.5535E5	2.9889E6	2.3037I

total 1 peptides

E5AJX3.1
back to list

| Protein Coverage | Supporting Peptides |
Protein Coverage:

1 MSSGGLLLLL GLLTLWAELT PVSSRDRPKF CYLPAEPGEC NAYMPS**FYYD SASNK**CKKFI YGGCRGNANN FKTRDECHHT

81 CVASGKGIQP RIGSN

Supporting Peptides:

Peptide	Uniq	-10lgP	Mass	Length	ppm	m/z	z	RT	Fraction	Scan	Source File	Area F1	Area F2	Area F3	Area I
S.FYYDSASNK.C	Y	63.13	1093.4716	9	0.3	547.7432	2	28.93	5	F5:7517	17112021_RID_2349_DP5.raw	8.2206E5	5.5535E5	2.9889E6	2.3037I

total 1 peptides

CBW30779.1
back to list

| Protein Coverage | Supporting Peptides |
Protein Coverage:

1 MSSGGLLLLL GLLTLWAELT PVSSRDRPKF CYLPAEPGEC NAYMPS**FYYD SASNK**CKKFI YGGCRGNANN FKTRDECHHT

81 CVASGKGIQP RIGSN

Supporting Peptides:

Peptide	Uniq	-10lgP	Mass	Length	ppm	m/z	z	RT	Fraction	Scan	Source File	Area F1	Area F2	Area F3	Area I
S.FYYDSASNK.C	Y	63.13	1093.4716	9	0.3	547.7432	2	28.93	5	F5:7517	17112021_RID_2349_DP5.raw	8.2206E5	5.5535E5	2.9889E6	2.3037I

total 1 peptides

TRINITY_DN151
back to list

| Protein Coverage | Supporting Peptides |
Protein Coverage:

1 MQQQNPEFKE QDPSFSHLVQ PRKLNKTSQM ALDHVQTEHA RRAPEFCYLP ADPAECNAYM PR**FYYDSASN K**CKEFIYGGC

81 RGNANNFKNR AECRHTCVAS RKGIQPRIAS N

Supporting Peptides:

Peptide	Uniq	-10lgP	Mass	Length	ppm	m/z	z	RT	Fraction	Scan	Source File	Area F1	Area F2	Area F3	Area I
R.FYYDSASNK.C	Y	63.13	1093.4716	9	0.3	547.7432	2	28.93	5	F5:7517	17112021_RID_2349_DP5.raw	8.2206E5	5.5535E5	2.9889E6	2.3037

total 1 peptides

Q6T269.1
back to list

| Protein Coverage | Supporting Peptides |

Protein Coverage:

1 PLRPGTEKHT FPFPAEFCNL PADLGPCKNY TGR**FYYDSAS NK**CEVFIYGG CPGNANNFKT REECRKTCVE ICILPAELGP

81 CDEYTGRFYY DSASNKCEVF IYGGCQGNAN NFKTRDECRK TCVEICILPA ELGPCDEYTG RLLLRLGIKQ M

Supporting Peptides:

Peptide	Uniq	-10lgP	Mass	Length	ppm	m/z	z	RT	Fraction	Scan	Source File	Area F1	Area F2	Area F3	Area
R.FYYDSASNK.C	Y	63.13	1093.4716	9	0.3	547.7432	2	28.93	5	F5:7517	17112021_RID_2349_DP5.raw	8.2206E5	5.5535E5	2.9889E6	2.3037

total 1 peptides

AAR19275.1

back to list

| Protein Coverage | Supporting Peptides |

Protein Coverage:

1 PLRPGTEKHT FPFPAEFCNL PADLGPCKNY TGR**FYYDSAS NK**CEVFIYGG CPGNANNFKT REECRKTCVE ICILPAELGP

81 CDEYTGRFYY DSASNKCEVF IYGGCQGNAN NFKTRDECRK TCVEICILPA ELGPCDEYTG RLLLRLGIKQ M

Supporting Peptides:

Peptide	Uniq	-10lgP	Mass	Length	ppm	m/z	z	RT	Fraction	Scan	Source File	Area F1	Area F2	Area F3	Area
R.FYYDSASNK.C	Y	63.13	1093.4716	9	0.3	547.7432	2	28.93	5	F5:7517	17112021_RID_2349_DP5.raw	8.2206E5	5.5535E5	2.9889E6	2.3037

total 1 peptides

LAA93895.1

back to list

| Protein Coverage | Supporting Peptides |

Protein Coverage:

1 YEELQDTAGR HNDDVRNAKH EISELNRHVQ RLKAEIEGVK KQCGNLKGKI AEAEDRGELA LKDARQK**LAE LEEALQK**AKQ

81 EMAKQLKEYQ QLLNVKISLD IEIATYKKLL EGEEYRLAND AGDAVNMSVI SSHISRGSAA GSGLGYGSGL SMGGGSCFAV

161 GGGGGCSNIV DLGNGSCSSM KFIS

Supporting Peptides:

Peptide	Uniq	-10lgP	Mass	Length	ppm	m/z	z	RT	Fraction	Scan	Source File	Area F1	Area F2	Area F3	Area F4	A
K.LAELEEALQK.A	Y	61.86	1142.6183	10	0.8	572.3169	2	41.52	4	F4:11731	17112021_RID_2349_DP4.raw	2.8419E4			7.8314E5	1.(

total 1 peptides

CAJ34939.1

back to list

| Protein Coverage | Supporting Peptides |

Protein Coverage:

1 MIQVLLVIIC LAVFPFQGSS KTLK**SGNVND YEVVNPGTVT GLPK**GAVEEK HEPMKGNTLQ KFPLCTTGPC CRQCKLKPAG

81 TTCWKTSRTS HYCTGKSCDC PSYPGNG

Supporting Peptides:

Peptide	Uniq	-10lgP	Mass	Length	ppm	m/z	z	RT	Fraction	Scan	Source File	Area F1	Area F2	Area F3
K.SGNVNDYEVVNPGTVTGLPK.G	Y	61.72	2059.0222	20	-0.3	1030.5181	2	48.96	7	F7:14386	17112021_RID_2349_DP7.raw			

total 1 peptides

Q3BK14.1

back to list

| Protein Coverage | Supporting Peptides |

Protein Coverage:

1 MIQVLLVIIC LAVFPFQGSS KTLK**SGNVND YEVVNPGTVT GLPK**GAVEEK HEPMKGNTLQ KFPLCTTGPC CRQCKLKPAG

81 TTCWKTSRTS HYCTGKSCDC PSYPGNG

Supporting Peptides:

Peptide	Uniq	-10lgP	Mass	Length	ppm	m/z	z	RT	Fraction	Scan	Source File	Area F1	Area F2	Area F3
K.SGNVNDYEVVNPGTVTGLPK.G	Y	61.72	2059.0222	20	-0.3	1030.5181	2	48.96	7	F7:14386	17112021_RID_2349_DP7.raw			

total 1 peptides

TRINITY_DN160

back to list

| Protein Coverage | Supporting Peptides |

Protein Coverage:

1 LASHSQREKN SGWLESRKRL PVFKPNPASK MIQVLLVIIC LAVFPFQGSS KTLK**SGNVND YEVVNPGTVT GLPK**GAVEEK

81 HEPMKGNTLQ KFPLCTTGPC CRQCKLKPAG TTCWKTSRTS HYCTGKSCDC PMYPGNG

Supporting Peptides:

Peptide	Uniq	-10lgP	Mass	Length	ppm	m/z	z	RT	Fraction	Scan	Source File	Area F1	Area F2	Are[illegible] F3
K.SGNVNDYEVVNPGTVTGLPK.G	Y	61.72	2059.0222	20	-0.3	1030.5181	2	48.96	7	F7:14386	17112021_RID_2349_DP7.raw			

total 1 peptides

JAS05406.1
back to list

| Protein Coverage | Supporting Peptides |
Protein Coverage:

```
  1 MIQVLLVTIC LAVFPYQGSS IILESGNVND YEVVYPRKVT AFPKGAVQPK YEDAMQYEFK VNGESVVLHL EKNKGLFSKD
 81 YSETHYSPDG REITTYPPAE DHCYYHGRIE NDADSTASIS ACNGLKGHFK LQGKIYLIEP LKLSDSEAHA VYKYENVEKE
161 DEAPKMCGVT QNWKSYEPIK KPSQLVVTAE QQRIAKRYVE FVIVVDHGMV TKHNETLRTW VFQIVNTINE IYIPLNIRFA
241 LVGLEIWSNG DKINVQSSAH NTLHLFGNWR RRVLLKRKRH DNAQLLTSID FDGQTVGLAH VSSMCNQKLS TGVIQDHSAI
321 NLLVAVTMAH ELGHNLGMDH DGNQCHCDAP SCVMSARLRE RPSYGFSNCS QNEYQMFIIN YNPQCILNEP LPTDIVSPPV
401 CGNELLEVGE ECDCGSPNNC QDPCCDATTC KQHSWVECES GECCEQCRFK GAGTECRAAS SECDIAESCT GQSADCPTDD
481 LHRNGQPCLN NHGYCYNGMC PIMDHQCIAL YGADATVAED ACFDSNLQGQ GNFYCRRENG RIFPCAPQDV KCGRLFCKFD
561 HYSCQYQYSG DPDFGMVDHG TKCADGKVCN NRRCVDVTTA Y
```

Supporting Peptides:

Peptide	Uniq	-10lgP	Mass	Length	ppm	m/z	z	RT	Fraction	Scan	Source File	Area F1	Area F2	Area F3	Area F4	A[illegible]
G.DPDFGMVDHGTK.C	Y	60.18	1317.5659	12	1.0	659.7909	2	34.72	4	F4:9473	17112021_RID_2349_DP4.raw		2.6453E5		1.04E6	

total 1 peptides

JAS05289.1
back to list

| Protein Coverage | Supporting Peptides |
Protein Coverage:

```
  1 MIEVLLVTIC LAVFPYQGSS IILESGNVND YEVVYPRKVT ALPKGAVQPN YEDAMQYEFK VNGESVVLHL EKNKELFSEN
 81 YSETHYSPDG REITTYPPAE DHCYYHGRIE NDADSTASIS ACNGLKGHFK LQGEMYLIEP LELPDSEAHA VFKYENVEKE
161 DEAPKMCGVT QTNWKSYEPI KKPSHLVVTA EQQRLTKRYV ELVIVADHGM FMKHNETLRT WVFQMVNTIN EIYIPLNIRF
241 ALVGLEIWSN GDKITVQSSA HNTLHLFGNW RKRVLLKRKK HDNAQLLTSI DFDGRTVGLA HVSSMCNQKL STGVIQDHSA
321 INLVMAVTMA HELGHNLGMD HDIKYCTCGA PACVMGNTLS EQPSYGFSNC SQNQYQTFII NYNPQCILNE PLPTDIVSPP
401 VCGNELLEVG EECDCGSPNT CRDPCCDATT CKQHSWVECE SGECCEQCKF KGAGTECRAA RSECDIAESC TGQSADCPTD
481 DLHRNGQPCL NNHGYCYNGM CPIMDHQCIA LYGADATVAP DACFDFNLKG QDNFYCRREK ARIFPCAPQD VKCGRLFCEF
561 GHYSCQYQYS GDPDFGMVDH GTKCADGKVC KNRQCVDVTT AY
```

Supporting Peptides:

Peptide	Uniq	-10lgP	Mass	Length	ppm	m/z	z	RT	Fraction	Scan	Source File	Area F1	Area F2	Area F3	Area F4	A[illegible]
G.DPDFGMVDHGTK.C	Y	60.18	1317.5659	12	1.0	659.7909	2	34.72	4	F4:9473	17112021_RID_2349_DP4.raw		2.6453E5		1.04E6	

total 1 peptides

JAS05287.1
back to list

| Protein Coverage | Supporting Peptides |
Protein Coverage:

```
  1 MIEVLLVTIC LAVFPYQGSS IILESGNVND YEVVYPRKVT ALPKGAVQPN YEDAMQYEFK VNGESVVLHL EKNKELFSEN
 81 YSETHYSPDG REITTYPPAE DHCYYHGRIE NDADSTASIS ACNGLKGHFK LQGEMYLIEP LELPDSEAHA VFKYENVEKE
161 DEAPKMCGVT QTNWKSYEPI KKPSHLVVTA EQQRLTKRYV ELVIVADHGM FMKHNETLRT WVFQMVNTIN EIYIPLNIRF
241 ALVGLEIWSN GDKITVQSSA HNTLHLFGNW RKRVLLKRKK HDNAQLLTSI DFDGRTVGLA HVSSMCNQKL STGVIQDHSA
321 INLVMAVTMA HELGHNLGMD HDIKYCTCGA PACVMGNTLS EQPSYGFSNC SQNQYQTFII NYNPQCILNE PLPTDIVSPP
401 VCGNELLEVG EECDCGSPNT CRDPCCDATT CKQHSWVECE SGECCEQCKF KGAGTECRAA RSECDIAESC TGQSADCPTD
481 DLHRNGQPCL NNHGYCYNGM CPIMDHQCIA LYGADATVAP DACFDFNLKG QDNFYCRREK ARIFPCAPQD VKCGRLFCEF
561 GHYSCQYQYS GDPDFGMVDH GTKCADGKVC KNRQCVDVTT AY
```

Supporting Peptides:

Peptide	Uniq	-10lgP	Mass	Length	ppm	m/z	z	RT	Fraction	Scan	Source File	Area F1	Area F2	Area F3	Area F4	A[illegible]
G.DPDFGMVDHGTK.C	Y	60.18	1317.5659	12	1.0	659.7909	2	34.72	4	F4:9473	17112021_RID_2349_DP4.raw		2.6453E5		1.04E6	

total 1 peptides

ABG26980.1
back to list

| Protein Coverage | Supporting Peptides |
Protein Coverage:

1 MIEVLLVTIC LAVFPYQGSS IILESGNVND YEVVYPRKVT ALPKGAVQPN YEDAMQYEFK VNGESVVLHL EKNKELFSEN
81 YSETHYSPDG REITTYPPAE DHCYYHGRIE NDADSTASIS ACNGLKGHFK LQGEMYLIEP LELPDSEAHA VFKYENVEKE
161 DEAPKMCGVT QTNWKSYEPI KKPSHLVVTA EQQRLTKRYV ELVIVADHGM FMKHNETLRT WVFQMVNTIN EIYIPLNIRF
241 ALVGLEIWSN GDKITVQSSA HNTLHLFGNW RKRVLLKRKK HDNAQLLTSI DFDGRTVGLA HVSSMCNQKL STGVIQDHSA
321 INLVMAVTMA HELGHNLGMD HDIKYCTCGA PACVMGNTLS EQPSYGFSNC SQNQYQTFII NYNPQCILNE PLPTDIVSPP
401 VCGNELLEVG EECDCGSPNT CRDPCCDATT CKQHSWVECE SGECCEQCKF KGAGTECRAA RSECDIAESC TGQSADCPTD
481 DLHRNGQPCL NNHGYCYNGM CPIMDHQCIA LYGADATVAP DACFDFNLKG QDNFYCRREK ARIFPCAPQD VKCGRLFCEF
561 GHYSCQYQYS G**DPDFGMVDH GTK**CADGKVC KNRQCVDVTT AY

Supporting Peptides:

Peptide	Uniq	-10lgP	Mass	Length	ppm	m/z	z	RT	Fraction	Scan	Source File	Area F1	Area F2	Area F3	Area F4	A
G.DPDFGMVDHGTK.C	Y	60.18	1317.5659	12	1.0	659.7909	2	34.72	4	F4:9473	17112021_RID_2349_DP4.raw		2.6453E5		1.04E6	

total 1 peptides

JAS05286.1
back to list

| Protein Coverage | Supporting Peptides |
Protein Coverage:

1 MIEVLLVTIC LAVFPYQGSS IILESGNVND YEVVYPRKVT ALPKGAVQPN YEDAMQYEFK VNGESVVLHL EKNKELFSEN
81 YSETHYSPDG REITTYPPAE DHCYYHGRIE NDADSTASIS ACNGLKGHFK LQGEMYLIEP LELPDSEAHA VFKYENVEKE
161 DEAPKMCGVT QTNWKSYEPI KKPSHLVVTA EQQRLTKRYV ELVIVADHGM FMKHNETLRT WVFQMVNTIN EIYIPLNIRF
241 ALVGLEIWSN GDKITVQSSA HNTLHLFGNW RKRVLLKRKK HDNAQLLTSI DFDGRTVGLA HVSSMCNQKL STGVIQDHSA
321 INLVMAVTMA HELGHNLGMD HDIKYCTCGA PACVMGNTLS EQPSYGFSNC SQNQYQTFII NYNPQCILNE PLPTDIVSPP
401 VCGNELLEVG EECDCGSPNT CRDPCCDATT CKQHSWVECE SGECCEQCKF KGAGTECRAA RSECDIAESC TGQSADCPTD
481 DLHRNGQPCL NNHGYCYNGM CPIMDHQCIA LYGADATVAP DACFDFNLKG QDNFYCRREK ARIFPCAPQD VKCGRLFCEF
561 GHYSCQYQYS G**DPDFGMVDH GTK**CADGKVC KNRQCVDVTT AY

Supporting Peptides:

Peptide	Uniq	-10lgP	Mass	Length	ppm	m/z	z	RT	Fraction	Scan	Source File	Area F1	Area F2	Area F3	Area F4	A
G.DPDFGMVDHGTK.C	Y	60.18	1317.5659	12	1.0	659.7909	2	34.72	4	F4:9473	17112021_RID_2349_DP4.raw		2.6453E5		1.04E6	

total 1 peptides

JAS05288.1
back to list

| Protein Coverage | Supporting Peptides |
Protein Coverage:

1 MIEVLLLTIC LAVFPYQGSS IILESGNVND YEVVYPRKVT ALPKGAVQPN YEDAMQYEFK VNGESVVLHL EKNKELFSEN
81 YSETHYSPDG REITTYPPAE DHCYYHGRIE NDADSTASIS ACNGLKGHFK LQGEMYLIEP LELPDSEAHA VFKYENVEKE
161 DEAPKMCGVT QTNWKSYEPI KKPSHLVVTA EQQRLTKRYV ELVIVADHGM FMKHNETLRT WVFQMVNTIN EIYIPLNIRF
241 ALVGLEIWSN GDKITVQSSA HNTLHLFGNW RKRVLLKRKK HDNAQLLTSI DFDGRTVGLA HVSSMCNQKL STGVIQDHSA
321 INLVMAVTMA HELGHNLGMD HDIKYCTCGA PACVMGNTLS EQPSYGFSNC SQNQYQTFII NYNPQCILNE PLPTDIVSPP
401 VCGNELLEVG EECDCGSPNT CRDPCCDATT CKQHSWVECE SGECCEQCKF KGAGTECRAA RSECDIAESC TGQSADCPTD
481 DLHRNGQPCL NNHGYCYNGM CPIMDHQCIA LYGADATVAP DACFDFNLKG QDNFYCRREK ARIFPCAPQD VKCGRLFCEF
561 GHYSCQYQYS G**DPDFGMVDH GTK**CADGKVC KNRQCVDVTT AY

Supporting Peptides:

Peptide	Uniq	-10lgP	Mass	Length	ppm	m/z	z	RT	Fraction	Scan	Source File	Area F1	Area F2	Area F3	Area F4	A
G.DPDFGMVDHGTK.C	Y	60.18	1317.5659	12	1.0	659.7909	2	34.72	4	F4:9473	17112021_RID_2349_DP4.raw		2.6453E5		1.04E6	

total 1 peptides

JAS05285.1
back to list

| Protein Coverage | Supporting Peptides |
Protein Coverage:

1 MIEVLLLTIC LAVFPYQGSS IILESGNVND YEVVYPRKVT ALPKGAVQPN YEDAMQYEFK VNGESVVLHL EKNKELFSEN
81 YSETHYSPDG REITTYPPAE DHCYYHGRIE NDADSTASIS ACNGLKGHFK LQGEMYLIEP LELPDSEAHA VFKYENVEKE
161 DEAPKMCGVT QTNWKSYEPI KKPSHLVVTA EQQRLTKRYV ELVIVADHGM FMKHNETLRT WVFQMVNTIN EIYIPLNIRF
241 ALVGLEIWSN GDKITVQSSA HNTLHLFGNW RKRVLLKRKK HDNAQLLTSI DFDGRTVGLA HVSSMCNQKL STGVIQDHSA
321 INLVMAVTMA HELGHNLGMD HDIKYCTCGA PACVMGNTLS EQPSYGFSNC SQNQYQTFII NYNPQCILNE PLPTDIVSPP
401 VCGNELLEVG EECDCGSPNT CRDPCCDATT CKQHSWVECE SGECCEQCKF KGAGTECRAA RSECDIAESC TGQSADCPTD
481 DLHRNGQPCL NNHGYCYNGM CPIMDHQCIA LYGADATVAP DACFDFNLKG QDNFYCRREK ARIFPCAPQD VKCGRLFCEF
561 GHYSCQYQYS G**DPDFGMVDH GTK**CADGKVC KNRQCVDVTT AY

Supporting Peptides:

Peptide	Uniq	-10lgP	Mass	Length	ppm	m/z	z	RT	Fraction	Scan	Source File	Area F1	Area F2	Area F3	Area F4	A 1
G.DPDFGMVDHGTK.C	Y	60.18	1317.5659	12	1.0	659.7909	2	34.72	4	F4:9473	17112021_RID_2349_DP4.raw		2.6453E5		1.04E6	

total 1 peptides

JAS05407.1
back to list

| Protein Coverage | Supporting Peptides |
Protein Coverage:

```
  1 MIQVLLVTIC LAVFPYQGSS IILESGNVND YEVVYPRKVT ALPKGAVQPK YEDAMQYEFK VNGEPVVLHL EKNKELFSEN
 81 YSETHYSPDG REITTYPPAE DHCYYHGRIE NDADSTASIS ACNGLKGHFK LQGKIYLIEP LKLSDSEAHA VYKYENVEKE
161 DEAPKMCGVT QNWKSYEPIK KASQLVVTAE QQRIAKRYVE FVIVVDHGMV TKHNETLRTW VFQIVNTINE IYIPLNIRFA
241 LVGLEIWSNG DKINVQSSAH NTLHLFGNWR RRVLLKRKRH DNAQLLTSID FDGQTVGLAH VSSMCNQKLS TGVIQDHSAI
321 NLLVAVTMAH ELGHNLGMDH DGNQCHCDAP SCVMSARLRE RPSYGFSNCS QNEYQMFIIN YNPQCILNEP LPTDIVSPPV
401 CGNELLEVGE ECDCGSPNNC QDPCCDATTC KQHSWVECES GECCEQCRFK GAGTECRAAS SECDIAESCT GQSADCPTDD
481 LHRNGQPCLN NHGYCYNGMC PIMDHQCIAL YGADATVAED ACFDSNLQGQ GNFYCRRENG RIFPCAPQDV KCGRLFCKFD
561 HYSCQYQYSG DPDFGMVDHG TKCADGKVCN NRRCVDVTTA Y
```

Supporting Peptides:

Peptide	Uniq	-10lgP	Mass	Length	ppm	m/z	z	RT	Fraction	Scan	Source File	Area F1	Area F2	Area F3	Area F4	A 1
G.DPDFGMVDHGTK.C	Y	60.18	1317.5659	12	1.0	659.7909	2	34.72	4	F4:9473	17112021_RID_2349_DP4.raw		2.6453E5		1.04E6	

total 1 peptides

JAS05408.1
back to list

| Protein Coverage | Supporting Peptides |
Protein Coverage:

```
  1 MIEVLLVTIF LAVFPYQGSS IILESGNVND YEVVYPRKVT ALPKGAVQPK YEDAMQYEFK VNGEPVVLHL EKNKELFSEN
 81 YSETHYSPDG REITTYPPAE DHCYYHGRIE NDADSTASIS ACNGLKGHFK LQGKIYLIEP LKLSDSEAHA VYKYENVEKE
161 DEAPKMCGVT QNWKSYEPIK KPSQLVVTAE QQRIAKRYVE FVIVVDHGMV TKHNETLRTW VFQIVNTINE IYIPLNIRFA
241 LVGLEIWSNG DKINVQSSAH NTLHLFGNWR RRVLLKRKRH DNAQLLTSID FDGQTVGLAH VSSMCNQKLS TGVIQDHSAI
321 NLLVAVTMAH ELGHNLGMDH DGNQCHCDAP SCVMSARLRE RPSYGFSNCS QNEYQMFIIN YNPQCILNEP LPTDIVSPPV
401 CGNELLEVGE ECDCGSPNNC QDPCCDATTC KQHSWVECES GECCEQCRFK GAGTECRAAS SECDIAESCT GQSADCPTDD
481 LHRNGQPCLN NHGYCYNGMC PIMDHQCIAL YGADATVAED ACFDSNLQGQ GNFYCRRENG RIFPCAPQDV KCGRLFCKFD
561 HYSCQYQYSG DPDFGMVDHG TKCADGKVCN NRRCVDVTTA Y
```

Supporting Peptides:

Peptide	Uniq	-10lgP	Mass	Length	ppm	m/z	z	RT	Fraction	Scan	Source File	Area F1	Area F2	Area F3	Area F4	A 1
G.DPDFGMVDHGTK.C	Y	60.18	1317.5659	12	1.0	659.7909	2	34.72	4	F4:9473	17112021_RID_2349_DP4.raw		2.6453E5		1.04E6	

total 1 peptides

QIV64953.1
back to list

| Protein Coverage | Supporting Peptides |
Protein Coverage:

```
  1 TIDFDGQTVG LAHISSMCNS KLSTGVIQDH SPINLLMAVT MAHELGHNLG MDHDTKYCTC GAPACIISDT LSHQPSKQFS
 81 NCSKEDYRTY LINRRPQCIL NEPLPTDIIS PPVCGNELLE VGEECDCGSP NTCQDPCCDA TTCKQHSWVE CESGECCEQC
161 KFKGAGTECQ AAKDDCDMAE SCTGRSAYCP TDDLHRNGQP CLNNHGYCYN GMCPIMDHQC IALYGADATV AEDACFDLNL
241 QGQGNFYCRR EKARIVPCAP QDVKCGRLFC KFDHYSCQYQ YSGDPDFGMV DHGTKCADGK VCKNRQCVDV TTAY
```

Supporting Peptides:

Peptide	Uniq	-10lgP	Mass	Length	ppm	m/z	z	RT	Fraction	Scan	Source File	Area F1	Area F2	Area F3	Area F4	A 1
G.DPDFGMVDHGTK.C	Y	60.18	1317.5659	12	1.0	659.7909	2	34.72	4	F4:9473	17112021_RID_2349_DP4.raw		2.6453E5		1.04E6	

total 1 peptides

ETE57622.1
back to list

| Protein Coverage | Supporting Peptides |
Protein Coverage:

1 MSHVSMWRTS SSTLLEKLSS LHNIKSNVVH PNDGSGIFGG SHGITGLHYI FFSLPLCKGI TSQPTLTQPV SQSGSPGGTV
81 KLSCAISSNP NNVRWVQQKS GEAPRFVHCD ACSSRGDGIP DRFMATRSGN NGYLTITNLQ AEDEADYFCC MWYSSGNMFH
161 IISHSGPLRG TIKLSCAISS DEMAISWLQQ RSGGAPHFVH CDGCNRGPGI PGRFTGTRSG NNGYLTITTL QAEDKANYYC
241 YMWYSSGGRF HRSFAQYVLT QPASVSVPLR QNARITCSAN NIASKNVHWY QQKPGDPPVL IIYNDNQRPT GISDQ**FSGTN**
321 **SGNTATLSIS R**AQAQDEAVY YCQVFDSNQC TKAQFSIMAW SLLLAFFFLG CTRATAQFTL TQPAAQSFPL GQPAKISCTM
401 SRDSQVGSYD ISWHQQKAGE SPKYLLSNMG SNGRFKGSKD ASANAAYLTI TNLQAEEEAD YYCGGAYGSG NNWWFPQ

Supporting Peptides:

Peptide	Uniq	-10lgP	Mass	Length	ppm	m/z	z	RT	Fraction	Scan	Source File	Area F1	Area F2	Area F3	Area F4	Ar[illegible] F5
Q.FSGTNSGNTATLSISR.A	Y	55.32	1611.7853	16	0.3	806.9001	2	36.24	7	F7:10113	17112021_RID_2349_DP7.raw					

total 1 peptides

XP_015672332.1
back to list

| Protein Coverage | Supporting Peptides |
Protein Coverage:

1 MPKPVNVRVT TMDAELEFAI QPNTTGKQLF DQVVKTIGLR EVWYFGLQCT DNKGFPTWLK LEKKVSAQEI RKENPLQFKF
81 RAKFFPEDVF EELIQDITQK LFFLQVKEGI LSDEIYCPPE TAVLLGSYAV QAKFGDYNKE LHKAGYLSSE RLVPQRVMNQ
161 HKLSREQWEE RIQVWHAEHG GMLKENAMLE YLKIAQDLEM YGINYFEIKN KKGTDLWLGV DALGLNIYEK EDKLTPKIGF
241 PWSEIRNISF NDKKFVIKPI DKK**SPDFIFY APR**LRINKRI LQLCMGNHEL YMRRRKPDTI EVQQMKAQAR EEKNQKELER
321 KMLEKEKKKR ETIEKEKEQM MREKEELMIR LQEYEVKTQK AEKELSDQIE KARQLEDERT RAQEEAERLE NERLMALQAK
401 EELEKQAVDQ MKSQEQLATE LAEYTAKISL LEEARKHKET EVEEWQHRAK EAQDDLVKTK EELHLVMTAP PPPPPPAYEP
481 VNYHVHDPLQ DEGIESSGYS AEFSSEGILN DRNEEKRITE TEKNERVQRQ LKTLTDELAQ ARDDEKKTQN DLIHTENMRQ
561 GRDKYKTLRQ IRQGNTKQRI DEFESM

Supporting Peptides:

Peptide	Uniq	-10lgP	Mass	Length	ppm	m/z	z	RT	Fraction	Scan	Source File	Area F1	Area F2	Area F3	Area F4	Area F5	Are[illegible] F6
K.SPDFIFYAPR.L	Y	52.38	1211.5974	10	0.5	606.8063	2	55.07	1	F1:16773	12112021_RID_2349_DP1.raw	7.951E4					

total 1 peptides

TRINITY_DN27458
back to list

| Protein Coverage | Supporting Peptides |
Protein Coverage:

1 MTQGSVLLFI LYMSHHVFAQ GQNSYLIQEC LNNNYTEKSC EKVFCQPWQT CIEGTCHCKI PYQCPRNGTS VCSTMRRSFQ
81 TYCQLKSYEC RQRLSKFLSR GVCNREAHFN VLFSHENASS EGIIQVELSN SEKSFLCGKG LTIHEANVIC RHLGFAEGAD
161 GKESVEPDED QNSHECLKAT CRGTETSLAE CGLKKLRLTT CKLAKVICHT EQRDCSPREF RCVNGKCISS AKTCDGLNDC
241 GDLSDELCCK ACKRKHFHCN SDVCIPSSFV CNNELDCLTG EDELNCNKTG GSKKVETEGR RTSNKEESNQ VIQSMDEERK
321 RTKSLFPVLS CGITNHTVTR RKRIIGGFTA KPHEFPWQVA IRGEGQIMNC GGVYIGGCWI LTAAHCVRRS RLHIYKIWTG
401 IVDSMKYSTR VDTFSLKDMI IHEHYNAK**TY QNDIALLEMK** TKNEGTPCSP RHTVPVCIPW SQYMFGSGHQ CKVSGWGLDE
481 ESTRQYILKW GYVTLMDNCS EIYKNRYFKG MECAGTDDGS VDACKGDSGG PLVCFDHNNV GYVWGVVSWG ENCGEKGHPG
561 VYTKVALYFD WIAQHTGISL ISKYNL

Supporting Peptides:

Peptide	Uniq	-10lgP	Mass	Length	ppm	m/z	z	RT	Fraction	Scan	Source File	Area F1	Area F2	Area F3	Area F4	Ar[illegible] F5
K.TYQNDIALLEMK.T	Y	51.48	1437.7173	12	1.1	719.8667	2	52.54	4	F4:15401	17112021_RID_2349_DP4.raw				1.3912E5	

total 1 peptides

XP_015683432.1
back to list

| Protein Coverage | Supporting Peptides |
Protein Coverage:

1 MTQGSVLLFI LYLSHHVFAQ GQNSYLIEEC LENNYTEQSC EKVFCQPWQT CIEGTCHCKI PYQCPRNGTL VCSTTRRSFQ
81 TYCQLKSYEC RQRSSKFLSR GLCNREARFN VLFSHKNASS EGIIQVELSN SEKSFLCEKG LTIHEANVIC KHLGFAEGAD
161 GKESVEPDED QNSHECLKAT CRGTETSLAE CGLKKMRLTI GKLAKVICHT EQRDCSPREF RCVNGKCISS TKTCDGLNDC
241 GDLSDELCCK ACKRKTFHCN SDVCIPSSSV CNNELDCLTG EDELNCNKTG RSKKGDSNQV IESVDEERKR TKTLLPVISC
321 GITNHTVTRR KRVIGGFTAK PHDFPWQVAI RGEGQTMNCG GVYIGGCWIL TAAHCVRRSR LHVYKIWTGV VDSIKFNSRV
401 ETFSLKDMII HEHYNSK**TYQ NDIALLEMK**T KNEGTPCSPP DTVPVCIPWS QYMFGGGHQC KVSGWGLDEG STRQYILKWG
481 YVTLMDNCSE IYKNRYFKGM ECAGTDDGSV DACKGDSGGP LVCFDHNNVG YLWGVVSWGE NCGERGHPGV YTKVANYFDW
561 IAQRTGIALI SKYNL

Supporting Peptides:

Peptide	Uniq	-10lgP	Mass	Length	ppm	m/z	z	RT	Fraction	Scan	Source File	Area F1	Area F2	Area F3	Area F4	Area F5
K.TYQNDIALLEMK.T	Y	51.48	1437.7173	12	1.1	719.8667	2	52.54	4	F4:15401	17112021_RID_2349_DP4.raw				1.3912E5	

total 1 peptides

XP_007421015.1
back to list

| Protein Coverage | Supporting Peptides |
Protein Coverage:

1 MGFPVALVFF LGGAVLRGAA VRCGYQSCPV TEPDMLNIHL IPHTHDDVGW LKTVDQYFFG VRNDIQHAGV QYILDSVIPQ
81 LLADPSKRFI YVEVAFFARW WGLQTEAMRQ AVRQLVAEGR LEFVNGGWCM NDEASVHYNA VIDQMSLGLH FLKETFGECG
161 RPRVAWHIDP FGHSREQASL FAQMGYDGFF LGRIDYQDKS HRESLREMEQ LWRASRNLDP PVADLFTGIL PNVYNPPVSL
241 CWDQICSDNP IVDDDSDENN VENIVAYFLG TATAQAKHYR TNHIVMTMGS DFQYENALLW YKNMDKLIHH VNAQQLRGSR
321 INVFYSTPSC YLWELNKANM SWSVKYDDFF PYADGPHQFW TGYFTSRPAL KRYERLSNNF LQICNQLEVL VGPKANLGPY
401 GSANSSVLRQ AMGVAQHHDA VSGTAKQHVT NDYAKQLSVG WDACQIVMSN ALASLVGSKE NFIYCTYLNI SVCPLTEAAP
481 TFT**VIIYNPL AR**SVDWNVRL PVNGSHYSVV APDGQNVLNE IIPVSSFTRA VRHNQGSAIN ELVFQASLPP LGYSTYSISR
561 LSARDPVRSR LLKRLRPQPA AEYLMLAIEN EHLQVLFDPR TGLMKEIRNL DKNISLPLLQ SFLWYNASVG NEEHSQPSGA
641 YIFRPDTSKP FPIAQEARIY QMKTQVVQEL YQNFSDWCSQ VVRLYAGQPY VELEWTVGPI PIWDHRGKEI ISRFETNLQT
721 EGLFYTDSNG REILERRRDY RVTWNLSQTE PVAGNYYPVN SRIYIKDRKT QLTVLTDRSQ GGSSLTDGSL ELMVHRRLLH
801 DDNRGVGEPL LEPGDYHNGL VVRGRHLVFL DTVESSAEQH RLRAQQEYMA PQLVLAPGGG PPYQPGQGIV KEFSALHHAL
881 PSNIHLLTLA QWEPDAVLIR LEHQFEHGEA VNGSLPVVIN LLNLFSSFNI VTVQEMNLSA NQKREDVSRL LWQPAEGVAA
961 EQRSGTGLDP AHIELFPMEI RTFLLQIRYQ DKPRQEM

Supporting Peptides:

Peptide	Uniq	-10lgP	Mass	Length	ppm	m/z	z	RT	Fraction	Scan	Source File	Area F1	Area F2	Area F3	Area F4	Area F5	Area F6
T.VIIYNPLAR.S	Y	50.81	1057.6284	9	0.8	529.8219	2	50.08	1	F1:15258	12112021_RID_2349_DP1.raw	1.4791E5					

total 1 peptides

TRINITY_DN13676
back to list

| Protein Coverage | Supporting Peptides |
Protein Coverage:

1 MAVAQHHDAV SGTAKQHVTN DYAKQLSIGW DACQIVMNNA FANLLGSTEN FIYCTYLNIS ACPLTEATPT FR**VIIYNPLA**
81 **R**SVDWNVRLP VNGSHYSVVA PDGQNILNEI IPVSSFTRAV RHNRGSAINE LVFQASLPPL GYKTYSVSRL SLRDPAHPKL
161 LKRMRPQPAA AHLTPSIENE HLQVLFDPST GLMKEIRNLD KSISLPLLQS FLWYNASDGN GEYSQASGAY IFHPDPSKAF
241 PIAHGAHVYQ IKTQVVQELY QNFSDWCSQV VRLYAGQPYV ELEWTVGPIP IGDQQGKEII SRFETNLQTE GLFYTDSNGR
321 EILERRRDYR FTWNLSQTEP VAGNYYPVNS RIYIKDRKTQ LTVLTDRSQG GSSLTDGSLE LMVHRRLLHD DNRGVQEPLL
401 EPGEYHNGLV VRGRHLIFLD TVESSAEQHR LWAQQEYMAP QVVLTPDSHD VVKEFSALRY ALPLNVHLLT LAQWEPDAVL
481 IRLEHQFEYG EAVNGSLPVV INLLDLFSSF NIIAVQETNL SANQKRENVS RLIWQSTEGI AAERRSGTGL DPAHIELFPM
561 QIRTFLLQIR S

Supporting Peptides:

Peptide	Uniq	-10lgP	Mass	Length	ppm	m/z	z	RT	Fraction	Scan	Source File	Area F1	Area F2	Area F3	Area F4	Area F5	Area F6
R.VIIYNPLAR.S	Y	50.81	1057.6284	9	0.8	529.8219	2	50.08	1	F1:15258	12112021_RID_2349_DP1.raw	1.4791E5					

total 1 peptides

TRINITY_DN13676
back to list

| Protein Coverage | Supporting Peptides |
Protein Coverage:

1 MRFPVALVFF LGGAVLRGSA VRCGYQSCPV TKPDMLNVHL IPHTHDDVGW LKTVDQYFFG VRNDIQHAGV QYILDSVIPQ
81 LLADPSKRFI YVEVAFFARW WGLQTEAMRQ AVQQLVAEGR LEFVNGGWCM NDEASVHYNA VIDQMSLGLH FLKETFGECG
161 RPRVAWHIDP FGHSREQASL FAQMGYDGFF LGRIDYQDKF HRESLREMEQ LWRASRNLDP PAADLFTGIL PNGYDPPMAL
241 CWDQICSDNP IVDDDSDENN VEKIVAYFLG IAAAQAKHYR TNHIVMTMGS DFQYENAMLW YKNLDKLIHH VNTQQLKGSR
321 VNVLYSTPSC YLWELNKANM TWSLKYDDFF PYANGPHQFW TGYYTSRPAL KRYERLSNNF LQICNQLEVI VGTKANFGPY
401 GNANSSVLRQ TMAVAQHHDA VSGTAKQHVT NDYAKQLSIG WDACQIVMNN AFANLLGSTE NFIYCTYLNI SACPLTEATP
481 TFR**VIIYNPL AR**SVDWNVRL PVNGSHYSVV APDGQNILNE IIPVSSFTRA VRHNRGSAIN ELVFQASLPP LGYKTYSVSR
561 LSLRDPAHPK LLKRMRPQPA AAHLTPSIEN EHLQVLFDPS TGLMKEIRNL DKSISLPLLQ SFLWYNASDG NGEYSQASGA
641 YIFHPDPSKA FPIAHGAHVY QIKVGKAQRH LLQIDLFAQ

Supporting Peptides:

Peptide	Uniq	-10lgP	Mass	Length	ppm	m/z	z	RT	Fraction	Scan	Source File	Area F1	Area F2	Area F3	Area F4	Area F5	Area F6

total 1 peptides

Peptide	Uniq	-10lgP	Mass	Length	ppm	m/z	z	RT	Fraction	Scan	Source File	Area F1	Area F2	Area F3	Area F4	Area F5	Are: F6
R.VIIYNPLAR.S	Y	50.81	1057.6284	9	0.8	529.8219	2	50.08	1	F1:15258	12112021_RID_2349_DP1.raw	1.4791E5					

total 1 peptides

TRINITY_DN13676
back to list

| Protein Coverage | Supporting Peptides |
Protein Coverage:

1 MRFPVALVFF LGGAVLRGSA VRCGYQSCPV TKPDMLNVHL IPHTHDDVGW LKTVDQYFFG VRNDIQHAGV QYILDSVIPQ
81 LLADPSKRFI YVEVAFFARW WGLQTEAMRQ AVQQLVAEGR LEFVNGGWCM NDEASVHYNA VIDQMSLGLH FLKETFGECG
161 RPRVAWHIDP FGHSREQASL FAQMGYDGFF LGRIDYQDKF HRESLREMEQ LWRASRNLDP PAADLFTGIL PNGYDPPMAL
241 CWDQICSDNP IVDDDSDENN VEKIVAYFLG IAAAQAKHYR TNHIVMTMGS DFQYENAMLW YKNLDKLIHH VNTQQLKGSR
321 VNVLYSTPSC YLWELNKANM TWSLKYDDFF PYANGPHQFW TGYYTSRPAL KRYERLSNNF LQICNQLEVI VGTKANFGPY
401 GNANSSVLRQ TMAVAQHHDA VSGTAKQHVT NDYAKQLSIG WDACQIVMNN AFANLLGSTE NFIYCTYLNI SACPLTEATP
481 TFR**VIIYNPL AR**SVDWNVRL PVNGSHYSVV APDGQNILNE IIPVSSFTRA VRHNRGSAIN ELVFQASLPP LGYKTYSVSR
561 LSLRDPAHPK LLKRMRPQPA AAHLTPSIEN EHLQVLFDPS TGLMKEIRNL DKSISLPLLQ SFLWYNASDG NGEYSQASGA
641 YIFHPDPSKA FPIAHGAHVY QIKVGKAQRH LLQIDLFAQ

Supporting Peptides:

Peptide	Uniq	-10lgP	Mass	Length	ppm	m/z	z	RT	Fraction	Scan	Source File	Area F1	Area F2	Area F3	Area F4	Area F5	Are: F6
R.VIIYNPLAR.S	Y	50.81	1057.6284	9	0.8	529.8219	2	50.08	1	F1:15258	12112021_RID_2349_DP1.raw	1.4791E5					

total 1 peptides

TRINITY_DN13676
back to list

| Protein Coverage | Supporting Peptides |
Protein Coverage:

1 MRFPVALVFF LGGAVLRGSA VRCGYQSCPV TKPDMLNVHL IPHTHDDVGW LKTVDQYFFG VRNDIQHAGV QYILDSVIPQ
81 LLADPSKRFI YVEVAFFARW WGLQTEAMRQ AVQQLVAEGR LEFVNGGWCM NDEASVHYNA VIDQMSLGLH FLKETFGECG
161 RPRVAWHIDP FGHSREQASL FAQMGYDGFF LGRIDYQDKF HRESLREMEQ LWRASRNLDP PAADLFTGIL PNGYDPPMAL
241 CWDQICSDNP IVDDDSDENN VEKIVAYFLG IAAAQAKHYR TNHIVMTMGS DFQYENAMLW YKNLDKLIHH VNTQQLKGSR
321 VNVLYSTPSC YLWELNKANM TWSLKYDDFF PYANGPHQFW TGYYTSRPAL KRYERLSNNF LQICNQLEVI VGTKANFGPY
401 GNANSSVLRQ TMAVAQHHDA VSGTAKQHVT NDYAKQLSIG WDACQIVMNN AFANLLGSTE NFIYCTYLNI SACPLTEATP
481 TFR**VIIYNPL AR**SVDWNVRL PVNGSHYSVV APDGQNILNE IIPVSSFTRA VRHNRGSAIN ELVFQASLPP LGYKTYSVSR
561 LSLRDPAHPK LLKRMRPQPA AAHLTPSIEN EHLQVLFDPS TGLMKEIRNL DKSISLPLLQ SFLWYNASDG NGEYSQASGA
641 YIFHPDPSKA FPIAHGAHVY QIKTQVVQEL YQNFSDWCSQ VVRLYAGQPY VELEWTVGPI PIGDQQGKEI ISRFETNLQT
721 EGLFYTDSNG REILERRRDY RFTWNLSQTE PVAGNYYPVN SRIYIKVTIS HGSAQTWLFL VSYRTACPHK A

Supporting Peptides:

Peptide	Uniq	-10lgP	Mass	Length	ppm	m/z	z	RT	Fraction	Scan	Source File	Area F1	Area F2	Area F3	Area F4	Area F5	Are: F6
R.VIIYNPLAR.S	Y	50.81	1057.6284	9	0.8	529.8219	2	50.08	1	F1:15258	12112021_RID_2349_DP1.raw	1.4791E5					

total 1 peptides

TRINITY_DN13676
back to list

| Protein Coverage | Supporting Peptides |
Protein Coverage:

1 MRFPVALVFF LGGAVLRGSA VRCGYQSCPV TKPDMLNVHL IPHTHDDVGW LKTVDQYFFG VRNDIQHAGV QYILDSVIPQ
81 LLADPSKRFI YVEVAFFARW WGLQTEAMRQ AVQQLVAEGR LEFVNGGWCM NDEASVHYNA VIDQMSLGLH FLKETFGECG
161 RPRVAWHIDP FGHSREQASL FAQMGYDGFF LGRIDYQDKF HRESLREMEQ LWRASRNLDP PAADLFTGIL PNGYDPPMAL
241 CWDQICSDNP IVDDDSDENN VEKIVAYFLG IAAAQAKHYR TNHIVMTMGS DFQYENAMLW YKNLDKLIHH VNTQQLKGSR
321 VNVLYSTPSC YLWELNKANM TWSLKYDDFF PYANGPHQFW TGYYTSRPAL KRYERLSNNF LQICNQLEVI VGTKANFGPY
401 GNANSSVLRQ TMAVAQHHDA VSGTAKQHVT NDYAKQLSIG WDACQIVMNN AFANLLGSTE NFIYCTYLNI SACPLTEATP
481 TFR**VIIYNPL AR**SVDWNVRL PVNGSHYSVV APDGQNILNE IIPVSSFTRA VRHNRGSAIN ELVFQASLPP LGYKTYSVSR
561 LSLRDPAHPK LLKRMRPQPA AAHLTPSIEN EHLQVLFDPS TGLMKEIRNL DKSISLPLLQ SFLWYNASDG NGEYSQASGA
641 YIFHPDPSKA FPIAHGAHVY QIKTQVVQEL YQNFSDWCSQ VVRLYAGQPY VELEWTVGPI PIGDQQGKEI ISRFETNLQT
721 EGLFYTDSNG REILERRRDY RFTWNLSQTE PVAGNYYPVN SRIYIKDRKT QLTVLTDRSQ GGSSLTDGSL ELMVHRRLLH
801 DDNRGVQEPL LEPGEYHNGL VVRGRHLIFL DTVESSAEQH RLWAQQEYMA PQVVLTPDSH DVVKEFSALR YALPLNVHLL
881 TLAQWEPDAV LIRLEHQFEY GEAVNGSLPV VINLLDLFSS FNIIAVQETN LSANQKRENV SRLIWQSTEG IAAERRSGTG
961 LDPAHIELFP MQIRTFLLQI RS

Supporting Peptides:

Peptide	Uniq	-10lgP	Mass	Length	ppm	m/z	z	RT	Fraction	Scan	Source File	Area F1	Area F2	Area F3	Area F4	Area F5	Area F6
R.VIIYNPLAR.S	Y	50.81	1057.6284	9	0.8	529.8219	2	50.08	1	F1:15258	12112021_RID_2349_DP1.raw	1.4791E5					

total 1 peptides

JAV50100.1
back to list

| Protein Coverage | Supporting Peptides |
Protein Coverage:

```
  1 MRFPVALVFF LGGAVLRGSA VRCGYQSCPV TKPDMLNVHL IPHTHDDVGW LKTVDQYFFG VRNDIQHAGV QYILDSVIPQ
 81 LLADPSKRFI YVEVAFFARW WGLQTEAIRQ AVQQLVAEGR LEFVNGGWCM NDEASVHYSA VIDQMSLGLH FLKETFGECG
161 RPRVAWHIDP FGHSREQASL FAQMGYDGFF LGRIDYQDKF HRESLREMEQ LWRASRNLDP PAADLFTGIL PNVYNPPMSL
241 CWDQICSDNP IVDDDSDENN VENIVAYFLG IAAFQAKHYR TNHIVMTMGS DFQYENALLW YKNLDKLIHH VNAQQLKGSR
321 VNVLYSTPSC YLWELNKANM TWSLKYDDFF PYADGPHQFW TGYYTSRPAL KRYERLSNNF LQICNQLEVI VGTKANFGPY
401 GNANSSVLRQ AMAVAQHHDA VSGTAKQHVT NDYAKQLSIG WDACQIVMNN AFANLLGSTE NFVYCTYLNI SACLLTEADP
481 TFRVIIYNPL ARPVDWNVRL PVNGSHYSVV APDGQNILNE IIPVSSFTRA VRHNRGSAIN ELVFQASLPP LGYKTYSVSR
561 LSLRDPAHPR LLNRMRPQPA AAHLTPSIKN EHLQVLFDPS TGLMKEIRNL DKSISLPLLQ SFLWYKASDG NAEHSQASGA
641 YIFRPDTFKA FPIAYGADVY QIKNRVVQEL YQNFSDWCSQ VVRLYAGQPY VELEWTVGPI PIGDQQGKEI ISRFETNLQT
721 EGLFYTDSNG REILERRRDY RFTWNLSQTE PVAGNYYPVN SRIYIKDRKT QLTVLTDRSQ GGSSLTDGSL ELMVHRRLLH
801 DDNRGVQEPL LEPGEYHNGL VVRGRHLIFL DTVESSAEQH RLWAQQEYMA PQVVLTPDSH DIVKEFSALR HALPLNVHLL
881 TLAQWEPDAV LIRLEHQFEY GEAVNGSLPV VINLLNLFSS FNIITVQEMN LSANQKRENV SRLIWQSTEG IAAKRRSGTG
961 LDPAHIELFP MQIRTFLVQI RY
```

Supporting Peptides:

Peptide	Uniq	-10lgP	Mass	Length	ppm	m/z	z	RT	Fraction	Scan	Source File	Area F1	Area F2	Area F3	Area F4	Area F5	Area F6
R.VIIYNPLAR.P	Y	50.81	1057.6284	9	0.8	529.8219	2	50.08	1	F1:15258	12112021_RID_2349_DP1.raw	1.4791E5					

total 1 peptides

LAB58574.1
back to list

| Protein Coverage | Supporting Peptides |
Protein Coverage:

```
  1 AMAVAQHHDA VSGTAKQHVT NDYAKQLSIG WDACQIVMNN AFANLLGSTE NFIYCTYLNI SACSLTEVAP AFRVIIYNPL
 81 ARSVDWNIRL PVNGSHYSVV APDG
```

Supporting Peptides:

Peptide	Uniq	-10lgP	Mass	Length	ppm	m/z	z	RT	Fraction	Scan	Source File	Area F1	Area F2	Area F3	Area F4	Area F5	Area F6
R.VIIYNPLAR.S	Y	50.81	1057.6284	9	0.8	529.8219	2	50.08	1	F1:15258	12112021_RID_2349_DP1.raw	1.4791E5					

total 1 peptides

XP_007421016.1
back to list

| Protein Coverage | Supporting Peptides |
Protein Coverage:

```
  1 MLNIHLIPHT HDDVGWLKTV DQYFFGVRND IQHAGVQYIL DSVIPQLLAD PSKRFIYVEV AFFARWWGLQ TEAMRQAVRQ
 81 LVAEGRLEFV NGGWCMNDEA SVHYNAVIDQ MSLGLHFLKE TFGECGRPRV AWHIDPFGHS REQASLFAQM GYDGFFLGRI
161 DYQDKSHRES LREMEQLWRA SRNLDPPVAD LFTGILPNVY NPPVSLCWDQ ICSDNPIVDD DSDENNVENI VAYFLGTATA
241 QAKHYRTNHI VMTMGSDFQY ENALLWYKNM DKLIHHVNAQ QLRGSRINVF YSTPSCYLWE LNKANMSWSV KYDDFFPYAD
321 GPHQFWTGYF TSRPALKRYE RLSNNFLQIC NQLEVLVGPK ANLGPYGSAN SSVLRQAMGV AQHHDAVSGT AKQHVTNDYA
401 KQLSVGWDAC QIVMSNALAS LVGSKENFIY CTYLNISVCP LTEAAPTFTV IIYNPLARSV DWNVRLPVNG SHYSVVAPDG
481 QNVLNEIIPV SSFTRAVRHN QGSAINELVF QASLPPLGYS TYSISRLSAR DPVRSRLLKR LRPQPAAEYL MLAIENEHLQ
561 VLFDPRTGLM KEIRNLDKNI SLPLLQSFLW YNASVGNEEH SQPSGAYIFR PDTSKPFPIA QEARIYQMKT QVVQELYQNF
641 SDWCSQVVRL YAGQPYVELE WTVGPIPIWD HRGKEIISRF ETNLQTEGLF YTDSNGREIL ERRRDYRVTW NLSQTEPVAG
721 NYYPVNSRIY IKDRKTQLTV LTDRSQGGSS LTDGSLELMV HRRLLHDDNR GVGEPLLEPG DYHNGLVVRG RHLVFLDTVE
801 SSAEQHRLRA QQEYMAPQLV LAPGGGPPYQ PGQGIVKEFS ALHHALPSNI HLLTLAQWEP DAVLIRLEHQ FEHGEAVNGS
881 LPVVINLLNL FSSFNIVTVQ EMNLSANQKR EDVSRLLWQP AEGVAAEQRS GTGLDPAHIE LFPMEIRTFL LQIRYQDKPR
961 QEM
```

Supporting Peptides:

Peptide	Uniq	-10lgP	Mass	Length	ppm	m/z	z	RT	Fraction	Scan	Source File	Area F1	Area F2	Area F3	Area F4	Area F5	Area F6
T.VIIYNPLAR.S	Y	50.81	1057.6284	9	0.8	529.8219	2	50.08	1	F1:15258	12112021_RID_2349_DP1.raw	1.4791E5					

total 1 peptides

XP_026552070.1

back to list

| Protein Coverage | Supporting Peptides |

Protein Coverage:

```
  1 MRFPVALVFF LGGAVLRGSA VKCGYQSCPV TKPDMLNVHL IPHTHDDVGW LKTVDQYFFG VRNDIQHAGV QYILDSVIPQ
 81 LLADPNKRFI YVEVVFFARW WELQTEAMRQ AVRQLVAEGR LEFVNGGWCM NDEASVHYNA VIDQMSLGLH FLKETFGECS
161 RPRVAWHIDP FGHSREQASL FAQMGYDGFF LGRIDYQDKF HRESLREMEQ LWRASRNLDP PSADLFTGIL PNGYNPPMSL
241 CWDQICSDNP IVDDNSDENN VESIVAYFLG TAAAQAKHYR TNHIVMTMGS DFQYENALLW YKNMDKLIYH VNAQQLRGSR
321 VNVLYSTPSC YLWELNKANM TWSMKYDDFF PYADGPHQFW TGYFTSRPAL KRYERLSNNF LQICNQLEVL VGTKANFGPY
401 GNANSSVLRQ AMAVAQHHDA VSGTAKQHVT NDYAKQLSIG WDACQIVMNN AFANLLGSTE NFIYCTYLNI SVCSLTEFAP
481 TFRVIIYNPL ARSVDWNIRL PVNGSHYSVV APDGQNILNE IIPVSSFTRA VRHNQGSATN ELVFQASLPP LGYRTYSVSR
561 LSGKDSTHPR LLKRLRPRPA AARLTPSIEN EHLQVLFDPN TGLMKEIRNL DKNISLPLSQ SFLWYNASDG NAAFSQASGA
641 YIFRPDTSKA FPIAQKVRVY QIKTQVVQEL YQNFSNWCSQ VVRLYAGQPY VELEWTVGPI PIADHYGKEI ISRFETNLQT
721 GGLFYTDSNG REILERRRDY RVTWNLSQTE PVAGNYYPVN TRMYIKDQKT QLTVLTDRSQ GGSSLTDGSL ELMVHRRLLH
801 DDNRGVQEPL LEPGEYHNGL VVRGRHLIFL DTVESSAEQH RLWAQQEYMA PQVVLTPDSH DIVKEFSALR HALPSNVHLL
881 TLAQWEPNAV LIRLEHQFEY GEAVNGSLPV AINLLNLFSS FNIITVQEMN LSANQKRENV SRLIWRSTQG IAAERQSGTR
961 LDPAHIELSP MQIRTFLLQI RY
```

Supporting Peptides:

Peptide	Uniq	-10lgP	Mass	Length	ppm	m/z	z	RT	Fraction	Scan	Source File	Area F1	Area F2	Area F3	Area F4	Area F5	Area F6
R.VIIYNPLAR.S	Y	50.81	1057.6284	9	0.8	529.8219	2	50.08	1	F1:15258	12112021_RID_2349_DP1.raw	1.4791E5					

total 1 peptides

JAA96625.1

back to list

| Protein Coverage | Supporting Peptides |

Protein Coverage:

```
  1 MRFPVALVFF LGGAVLRGSA VRCGYQSCPV TKPDMLNVHL IPHTHDDVGW LKTVDQYFFG VRNDIQHAGV QYILDSVIPQ
 81 LLADPSKRFI YVEVAFFARW WGLQTEAMRQ AVQQLVAEGR LEFVNGGWCM NDEASVHYSA VIDQMSLGLH FLKETFGECG
161 RPRVAWHIDP FGHSREQASL FAQMGYDGFF LGRIDYQDKF HRESLREMEQ LWRASRNLDP PAADLFTGIL PNVYNPPMSL
241 CWDQICSDNP IVDDDSDENN VENIVAYFLG IAAFQAKHYR TNHIVMTMGS DFQYENALLW YKNLDKLIHH VNAQQLKGSR
321 VNVLYSTPSC YLWELNKANM TWSLKYDDFF PYADGPHQFW TGYYTSRPAL KRYERLSNNF LQICNQLEVI VGRKANFGPY
401 GNANSSVLRQ AMAVAQHHDA VSGTAKQHVT NDYAKQLSIG WDACQIVMSN AFANLLGSTE NFVYCTYLNI SACPLTEADP
481 TFRVIIYNPL ARPVDWNVRL PVNGSHYSVV APDGQNILNE IIPVSSFTRA VRHNRGNAIN ELVFQASLPP LGYKTYSVSR
561 LSLRDPAHPR LLNRMRPQPV AAPLTPSIEN EHLQVLFDPS TGLMKEIRNL DKSISLPLLQ SFLWYNASDG NAEHSQASGA
641 YIFRPDTFKA FPIAYGADVY QIKNQVVQEL YQNFSDWCSQ VVRLYAGQPY VELEWTVGPI PIGDQQGKEI ISRFETNLQT
721 EGLFYTDSNG REILERRRDY RFTWNLSQTE PVAGNYYPVN SRIYIKDRKT QLTVLTDRSQ GGSSLTDGSL ELMVHRRLLR
801 DDNRGVQEPL LEPGEYHNGL VVRGRHLIFL DTVESSAEQH RLWAQQEYMA PQVVLTPDSH DIVKEFSALQ HALPLNVHLL
881 TLAQWEPDAV LIRLEHQFEY GEAVNGSLPV VVNLLNLFSS FNIITVQEMN LSANQKRENV SRLIWQSIEG IAAKRRSGTG
961 LDPAHVELFP MQIRTFLLQI RY
```

Supporting Peptides:

Peptide	Uniq	-10lgP	Mass	Length	ppm	m/z	z	RT	Fraction	Scan	Source File	Area F1	Area F2	Area F3	Area F4	Area F5	Area F6
R.VIIYNPLAR.P	Y	50.81	1057.6284	9	0.8	529.8219	2	50.08	1	F1:15258	12112021_RID_2349_DP1.raw	1.4791E5					

total 1 peptides

XP_026537394.1

back to list

| Protein Coverage | Supporting Peptides |

Protein Coverage:

1 MRFPVALVFF LGGAVLRGSA VKCGYQSCPV TKPDMLNVHL IPHTHDDVGW LKTVDQYFFG VRNDIQHAGV QYILDSVIPQ
81 LLADPSKRFI YVEVVFFARW WELQTEAMRQ AVRQLVAEGR LEFVNGGWCM NDEASVHYNA VIDQMSLGLH FLKETFGECS
161 RPRVAWHIDP FGHSREQASL FAQMGYDGFF LGRIDYQDKF HRESLREMEQ LWRASRNLDP PSADLFTGIL PNGYNPPMSL
241 CWDQICSDNP IVDNNSDENN VESIVAYFLG TAAAQAKHYR TNHIVMTMGS DFQYENALLW YKNMDKLIYH VNAQQLRGSR
321 VNVLYSTPSC YLWELNKANM TWSMKYDDFF PYADGPHQFW TGYFTSRPAL KRYERLSNNF LQICNQLEVL VGTKANFGPY
401 GNANSSVLRQ AMAVAQHHDA VSGTAKQHVT NDYAKQLSIG WDACQIVMNN AFANLLGSTE NFIYCTYLNI SVCSLTEVAP
481 TFR**VIIYNPL AR**SVDWNIRL PVNGSHYSVV APDGQNILNE IIPVSSFTRA VRHNQGSATN ELVFQASLPP LGYRTYSVSR
561 LSGKDSTRPR LLKRLRPQPA AARLTPSIEN EHLQVLFDPN TGLMKEIRNL DKNISLPLSQ SFLWYNASVG NAAFPQASGA
641 YIFRPDTSKA FPIAQKVRVY QIKTQVVQEL YQNFSNWCSQ VVRLYAGQPY VELEWTVGPI PIADHYGKEI ISRFETNLQT
721 GGLFYTDANG REILERRRDY RVTWNLSQTE PVAGNYYPVN TRMYIKDQKT QLTVLTDRSQ GGSSLTDGSL ELMVHRRLLH
801 DDNRGVQEPL LEPGEYHNGL VVRGRHLIFL DTVESSAEQH RLWAQQEYMA PQVVLTPDSH DIVKEFSALR HALPSNVHLL
881 TLAQWEPDAV LIRLEHQFEY GEAVNGSLPV AINLLNLFSS FNIITVQEMN LSANQKRENV SRLIWQSTQG IAAERQSGTR
961 LDPAHIELSP MQIRTFLLQI RY

Supporting Peptides:

Peptide	Uniq	-10lgP	Mass	Length	ppm	m/z	z	RT	Fraction	Scan	Source File	Area F1	Area F2	Area F3	Area F4	Area F5	Are F6
R.VIIYNPLAR.S	Y	50.81	1057.6284	9	0.8	529.8219	2	50.08	1	F1:15258	12112021_RID_2349_DP1.raw	1.4791E5					

total 1 peptides

JAG44103.1
back to list

| Protein Coverage | Supporting Peptides |
Protein Coverage:

1 MRFPVALVFF LGGAVLRGSA VRCGYQSCPV TKPDMLNVHL IPHTHDDVGW LKTVDQYFFG VRNDIQHAGV QYILDSVIPQ
81 LLADPSKRFI YVEVAFFARW WGLQTEAMRQ AVQQLVAEGR LEFVNGGWCM NDEASVHYSA VIDQMSLGLH FLKETFGECG
161 RPRVAWHIDP FGHSREQASL FAQMGYDGFF LGRIDYQDKF HRESLREMEQ LWRASRNLDP PAADLFTGIL PNVYNPPMSL
241 CWDQICSDNP IVDDDSDENN VENIVAYFLG IAAFQAKHYR TNHIVMTMGS DFQYENALLW YKNLDKLIHH VNAQQLKGSR
321 VNVLYSTPSC YLWELNKANM TWSLKYDDFF PYADGPHQFW TGYYTSRPAL KRYERLSNNF LQICNQLEVI VGRKANFGPY
401 GNANSSVLRQ AMAVAQHHDA VSGTAKQHVT NDYAKQLSIG WDACQIVMSN AFANLLGSTE NFVYCTYLNI SACPLTEADP
481 TFR**VIIYNPL AR**PVDWNVRL PVNGSHYSVV APDGQNILNE IIPVSSFTRA VRHNRGNAIN ELVFQASLPP LGYKTYSVSR
561 LSLRDPAHPR LLNRMRPQPV AAPLTPSIEN EHLQVLFDPS TGLMKEIRNL DKSISLPLLQ SFLWYNASDG NAEHSQASGA
641 YIFRPDTFKA FPIAYGADVY QIKNQVVQEL YQNFSDWCSQ VVRLYAGQPY VELEWTVGPI PIGDQQGKEI ISRFETNLQT
721 EGLFYTDSNG REILERRRDY RFTWNLSQTE PVAGNYYPVN SRIYIKDRKT QLTVLTDRSQ GGSSLTDGSL ELMVHRRLLR
801 DDNRGVQEPL LEPGEYHNGL VVRGRHLIFL DTVESSAEQH RLWAQQEYMA PQVVLTPDSH DIVKEFSALQ HALPLNVHLL
881 TLAQWEPDAV LIRLEHQFEY GEAVNGSLPV VVNLLNLFSS FNIITVQEMN LSANQKRENV SRLIWQSIEG IAAKRRSGTG
961 LDPAHVELFP MQIRTFLLQI RY

Supporting Peptides:

Peptide	Uniq	-10lgP	Mass	Length	ppm	m/z	z	RT	Fraction	Scan	Source File	Area F1	Area F2	Area F3	Area F4	Area F5	Are F6
R.VIIYNPLAR.P	Y	50.81	1057.6284	9	0.8	529.8219	2	50.08	1	F1:15258	12112021_RID_2349_DP1.raw	1.4791E5					

total 1 peptides

XP_026537257.1
back to list

| Protein Coverage | Supporting Peptides |
Protein Coverage:

1 MRFPVALVFF LGGAVLRGSA VKCGYQSCPV TKPDMLNVHL IPHTHDDVGW LKTVDQYFFG VRNDIQHAGV QYILDSVIPQ
81 LLADPSKRFI YVEVVFFARW WELQTEAMRQ AVRQLVAEGR LEFVNGGWCM NDEASVHYNA VIDQMSLGLH FLKETFGECS
161 RPRVAWHIDP FGHSREQASL FAQMGYDGFF LGRIDYQDKF HRESLREMEQ LWRASRNLDP PSADLFTGIL PNGYNPPMSL
241 CWDQICSDNP IVDNNSDENN VESIVAYFLG TAAAQAKHYR TNHIVMTMGS DFQYENALLW YKNMDKLIYH VNAQQLRGSR
321 VNVLYSTPSC YLWELNKANM TWSMKYDDFF PYADGPHQFW TGYFTSRPAL KRYERLSNNF LQICNQLEVL VGTKANFGPY
401 GNANSSVLRQ AMAVAQHHDA VSGTAKQHVT NDYAKQLSIG WDACQIVMNN AFANLLGSTE NFIYCTYLNI SVCSLTEVAP
481 TFR**VIIYNPL AR**SVDWNIRL PVNGSHYSVV APDGQNILNE IIPVSSFTRA VRHNQGSATN ELVFQASLPP LGYRTYSVSR
561 LSGKDSTRPR LLKRLRPQPA AARLTLSIEN EHLQVLFDPN TGLMKEIRNL DKNISLPLSQ SFLWYNASVG NAAFPQASGA
641 YIFRPDTSKA FPIAQKVRVY QIKTQVVQEL YQNFSNWCSQ VVRLYAGQPY VELEWTVGPI PIADHYGKEI ISRFETNLQT
721 GGLFYTDANG REILERRRDY RVTWNLSQTE PVAGNYYPVN TRMYIKDQKT QLTVLTDRSQ GGSSLTDGSL ELMVHRRLLH
801 DDNRGVQEPL LEPGEYHNGL VVRGRHLIFL DTVESSAEQH RLWAQQEYMA PQVVLTPDSH DIVKEFSALR HALPSNVHLL
881 TLAQWEPDAV LIRLEHQFEY GEAVNGSLPV AINLLNLFSS FNIFTVQEMN LSANQKRENV SRLIWQSTQG IAAERQSGTR
961 LDPAHIELSP MQIRTFLLQI RY

Supporting Peptides:

Peptide	Uniq	-10lgP	Mass	Length	ppm	m/z	z	RT	Fraction	Scan	Source File	Area F1	Area F2	Area F3	Area F4	Area F5	Area F6
R.VIIYNPLAR.S	Y	50.81	1057.6284	9	0.8	529.8219	2	50.08	1	F1:15258	12112021_RID_2349_DP1.raw	1.4791E5					

total 1 peptides

LAB27223.1
back to list

| Protein Coverage | Supporting Peptides |
Protein Coverage:

1 STWGSLSCRN RVGVMGFPVA LVFFLGGAVL RGSAVKCGYQ SCPVTKPDML NVHLIPHTHD DVGWLKTVDQ YFFGVRNDIQ

81 HAGVQYILDS VIPQLLADPS KRFIYVEVVF FARWWELQTE AMHQAVRQLV AEGRLEFVNG GWCMNDEASV HYNAVIDQMS

161 LGLHFLKETF GECSRPRVAW HIDPFGHSRE QASLFAQMGY DGFFLGRIDY QDKFHRESLR EMEQLWRASR NLDPPSADLF

241 TGILPNGYNP PMSLCWDQIC SDNPIVDDDS DENNVENIVA YFLGTAAAQA KHYRTNHIVM TMGSDFQYEN ALLWYKNMDK

321 LIYHVNAQQV RGSRVNVLYS TPSCYLWELN KANMTWSMKY DDFFPYADGP HQFWTGYFTS RPALKRYERL SNNFLQICNQ

401 LEVLVGTKAN FGPYGNANSS VLRKAMAVAQ HHDAVSGTAK QHVTNDYAKQ LSIGWDACQI VMNNAFANLL GSTENFIYCT

481 YLNISACSLT EVAPAFR**VII YNPLAR**SVDW NIRLPVNGSH YSVVAPDGQN IMNEIIPVSS FTRAVRHNRG SATNELVFQA

561 SLPPLGYRTY SIARLSDKDS AHPRLLKRLR PQPAAAHLTP LIENEHLQVL FDPNTGLMKE IRNLKKNISL PLSQSFLWYN

641 ASVGNAAFSQ ASGAYIFRPD TSKAFPIAQK VGVYQIKTQV VQELYQNFSN WCSQVVRLYA GQPYVELEWT VGPIPIADHY

721 GKEIISRFET NLQTGGLFYT DSNGREILER RRDYRVTWNL NQTEPVAGNY YPVNTRMYIK DQKTQLTVLT DRSQGGSSLT

801 DGSLELMVHR RLLHDDNRGV QEPLLEPGDY HNGLVVRGRH LIFLDTVESS AEQHRLWAQQ EYMAPQVVLT PDSHDIVKEF

881 SALRHALPSN VHLLTLAQWE PDAVLIRLEH QFEYGEAVNG SLPVTINLLN LFSSFNIITV QEMNLSANQK RENVSRLIWQ

961 STQGIAAERQ SGTRLDPAHI ELSPMQIRTF LLQIRY

Supporting Peptides:

Peptide	Uniq	-10lgP	Mass	Length	ppm	m/z	z	RT	Fraction	Scan	Source File	Area F1	Area F2	Area F3	Area F4	Area F5	Area F6
R.VIIYNPLAR.S	Y	50.81	1057.6284	9	0.8	529.8219	2	50.08	1	F1:15258	12112021_RID_2349_DP1.raw	1.4791E5					

total 1 peptides

XP_032065032.1
back to list

| Protein Coverage | Supporting Peptides |
Protein Coverage:

1 MIIAVRMSCW KGVGTMGFHV ALVFFLGGAV LGGSAVRCGY QSCPVTKPDM LNVHLIPHTH DDVGWLKTVD QYFFGVRNDI

81 QHAGVQYILD SVIPQLLADP SKRFIYVEVV FFARWWGLQT EAMRQAVRQL VAEGRLEFVN GGWCMNDEAS VHYSAVIDQM

161 SLGLQFLKET FGECARPRVA WHIDPFGHSR EQASLFAQMG YDGFFLGRID YQDKSHRESL REMEQLWRAS RNLDPPAADL

241 FTGILPNGYN PPMSLCWDQI CSDNPIVDDD SDENNVENIV AYFLGTAAVQ AKHYRTNHIV MTMGSDFQYE NALLWYKNMD

321 KLIYHVNAQQ LKGSHVNVFY STPSCYLWEL NKANMTWSIK YDDFFPYADG PHQFWTGYFT SRPAFKRYER LSNNFLQICN

401 QLEVLVGTKA NFGPYGNANS SVLRQAMAVA QHHDAVSGTA KQHVTNDYAK QLSIGWDTCQ IVMNNALANL LGSTENFIYC

481 TYLNISACPL TEATPAFR**VI IYNPLAR**SVD WNVRLPVNGS HYSVVAPDGQ NILNELIPVS SFTRAVRHNR GSAINELVFQ

561 ASLPPLGYRT YSISRLSGRD SARPKLLKRL RPQQAGAHFT PLIENEHLQV LFDPNTGLMK EIRNLDKNIS LPLFQSFLWY

641 NASVGDASYS QASGAYIFRP DTSKAFPIAQ KVGVYQIKTQ VVQELYQNFS NWCSQVVRLY AGQPYVELEW TVGPIPIQDQ

721 YGKEIISRFE TNLQTGGHFY TDSNGREILE RRRDYRATWN LSQTEPVAGN YYPVNTRIYI KDQKTQLTVL TDRSQGGSSL

801 ADGSLELMVH RRLLHDDNRG VQEPLLEFGE YHNGLVVRGR HLVFLDTVES SAEKHRLWAQ QEYMAPQVVL TPDSHDIVKE

881 FSALRHALPL NVHLLTLAQW EPDAVLIRLE HQFEYGEAVN GSLPVAINLL NLFSSFNIIS VQEVNLSANQ KRENVSRLTW

961 QSTQGMAAER RSGTVLDPAH IELFPMQIRT FLVQIRY

Supporting Peptides:

Peptide	Uniq	-10lgP	Mass	Length	ppm	m/z	z	RT	Fraction	Scan	Source File	Area F1	Area F2	Area F3	Area F4	Area F5	Area F6
R.VIIYNPLAR.S	Y	50.81	1057.6284	9	0.8	529.8219	2	50.08	1	F1:15258	12112021_RID_2349_DP1.raw	1.4791E5					

total 1 peptides

XP_034262785.1
back to list

| Protein Coverage | Supporting Peptides |
Protein Coverage:

1 MIAAVSLSCW NGVGAMGIPV ALVFLLGGAV LRGSAVRCGY QSCPVTKPDM LNVHLIPHTH DDVGWLKTVD QYFFGVRNDI
81 QHAGVQYILD SVVPQLLADP SKRFIYVEVV FFARWWGLQT EAMRQAVRQL VAEGRLEFVN GGWCMNDEAS VHYNAVIDQM
161 SLGLHFLKET FGECGRPRVA WHIDPFGHSR EQASLFAQMG YDGFFLGRID YQDKFHRESL REMEQLWRAS SNLDPPAADL
241 FTGILPNGYN PPMSLCWDQI CSDNPIVDDD SDENNVENIV AYFLGVAAAQ AKHYRTNHIV MTMGSDFQYE NALLWYKNMD
321 KLIYHVNAQQ LKGSRVNVFY STPSCYLWEL NKANITWTMK YDDFFPYADG PHQFWTGYFT SRPALKRYER LSNNFLQICN
401 QLEVLVGTKA NFGPYGNANS SVLRQAMAVA QHHDAVSGTA KQHVTNDYAK QLSIGWDACQ IVMNNAFANL LGSTENFIYC
481 TYLNISACPL TEATPAFR**VI IYNPLAR**SVD WNVRLPVNGS HYSVVAPDGQ NILNEIIPVS SFTRAVRHNR GSAINELVFQ
561 ASLPPLGYRT YSISRLSGRD SARPRLQKRL RPRPAAAHLT PSIENEHLQV LFDPKTGLMK EIRNLDKNIS LPLFQSFLWY
641 NASDGNAAYS QASGAYIFRP DTSKAFPIAQ KVRVYQIKTQ VVQELYQNFS DWCSQVVRLY AGQPYVELEW TVGPIPIEDL
721 YGKEIISRFE TNLQTGGLFY TDSNGREILE RRRDYRVTWN LSQTEPVAGN YDPVNTRIYI KDQKTQLTVL TDRSQGGSSL
801 TDGSLELMVH RRLLHDDNRG VQEPLLEPGE YHNGLVVRGR HLVFLDTVES SAEQHRLWAQ QEYMAPQVVL TPDSHDIVKE
881 FSALRHALPL NVHLLTLAQW EPDAVLIRLE HQFEYGEAVN GSLPVAINLL NLFSSFNIIA VQEMNLSANQ KRENVSRLIW
961 QSTQGIAAER RSGTRLDPAH IELSPMQIRT FLVQIRY

Supporting Peptides:

Peptide	Uniq	-10lgP	Mass	Length	ppm	m/z	z	RT	Fraction	Scan	Source File	Area F1	Area F2	Area F3	Area F4	Area F5	Are[illegible] F6
R.VIIYNPLAR.S	Y	50.81	1057.6284	9	0.8	529.8219	2	50.08	1	F1:15258	12112021_RID_2349_DP1.raw	1.4791E5					

total 1 peptides

XP_013919874.1
back to list

| Protein Coverage | Supporting Peptides |
Protein Coverage:

1 MIIAIRMSCW KGVGTMGFHV ALVFFLGGAV LGGSAVRCGY QSCPVTKPDM LNVHLIPHTH DDVGWLKTVD QYFFGVRNDI
81 QHAGVQYILD SVIPQLLADP SKRFIYVEVV FFARWWGLQT EAMRQAVRQL VAEGRLEFVN GGWCMNDEAS VHYSAVIDQM
161 SLGLQFLKET FGECARPRVA WHIDPFGHSR EQASLFAQMG YDGFFLGRID YQDKSHRESL REMEQLWRAS RNLDPPAADL
241 FTGILPNGYN PPMSLCWDQI CSDNPIVDDD SDENNVENIV AYFLGTAAVQ AKHYRTNHIV MTMGSDFQYE NALLWYKNMD
321 KLIYHVNAQQ LKGSHVNVFY STPSCYLWEL NKANMTWSIK YDDFFPYADG PHQFWTGYFT SRPAFKRYER LSNNFLQICN
401 QLEVLVGTKA NFGPYGNANS SVLRQAMAVA QHHDAVSGTA KQHVTNDYAK QLSIGWDTCQ IVMNNALANL LGSTENFIYC
481 TYLNISACPL TEATPAFR**VI IYNPLAR**SVD WNVRLPVNGN HYSVVAPDGQ NILNELIPVS SFTRAVRHNR GNAINELVFQ
561 ASLPPLGYRT YSISRLSGRD SARPRLLKRL RPQQAGAHFT PLIENEHLQV LFDPNTGLMK EIRNLDKNIS LPLFQSFLWY
641 NASVGDASYS QASGAYIFRP DTSKAFPIAQ KVGVYQIKTQ VVQELYQNFS NWCSQVVRLY AGQPYVELEW TVGPIPIQDQ
721 YGKEIISRFE TNLQTGGHFY TDSNGREILE RRRDYRATWN LSQTEPVAGN YYPVNTRIYI KDQKTQLTVL TDRSQGGSSL
801 ADGSLELMVH RRLLHDDNRG VQEPLLEFGE YHNGLVVRGR HLVFLDTVES SAEKHRLWAQ QEYMAPQVVL TPDSHDIVKE
881 FSALRHALPL NVHLLTLAQW EPDAVLIRLE HQFEYGEAVN GSLPVAINLL NLFSSFNIVS VQEVNLSANQ KRENVSRLTW
961 QSTQGMAAER RSGTGLDPAH IELFPMQIRT FLVQIRY

Supporting Peptides:

Peptide	Uniq	-10lgP	Mass	Length	ppm	m/z	z	RT	Fraction	Scan	Source File	Area F1	Area F2	Area F3	Area F4	Area F5	Are[illegible] F6
R.VIIYNPLAR.S	Y	50.81	1057.6284	9	0.8	529.8219	2	50.08	1	F1:15258	12112021_RID_2349_DP1.raw	1.4791E5					

total 1 peptides

JAV49704.1
back to list

| Protein Coverage | Supporting Peptides |
Protein Coverage:

1 MKVLRFFAPV LCLLWLGQVC LAVDIEEEEG VLVLKSANFD QALEQYPNIL VEFYAPWCGH CKALAPEYVK AAAK**LLSENS**
81 **EIR**LAKVDAT EESELAQQFG VRGYPTIKFF KNGDKSSPKE YTAGREADDI LNWLKKRTGP AATTLADVAA VEELVESNEV
161 AVIGFFKDVE SDVAKEFLLA AEAIDDIPFG ITSKSDIFAK YQLKKDGVVL FKKFDEGRNN FDGEITKDNL LNFIKSNQLP
241 LVIEFTEQTA PKIFGGEIKT HILLFLPKSV EEYQGKLDNF KTAAEDFRGK ILFIYIDSDH SDNQRILEFF GLKKEECPAI
321 RLITLEEEMT KYKPESDELT PENIRDFCHK FLEGKVKPHL MSQEISDDWD KQPVKVLVGK NFEEVAFDEN KNVFVEFYAP
401 WCGHCKQLAP IWDKLGETYK DHENIIIAKM DSTVNEVEVV KVHSFPTLKY FPAGPDRTVV DYNGERTLEG FKKFLESGGK
481 DGGVDENDLE DLEDAEEEPD VEEEEEETGA KKDEL

Supporting Peptides:

Peptide	Uniq	-10lgP	Mass	Length	ppm	m/z	z	RT	Fraction	Scan	Source File	Area F1	Area F2	Area F3	Area F4	Area F5	Area F6	Ar[illegible]
K.LLSENSEIR.L	Y	50.02	1059.5560	9	0.3	530.7855	2	30.07	7	F7:7892	17112021_RID_2349_DP7.raw							1.33

total 1 peptides

JAA96208.1
back to list

| Protein Coverage | Supporting Peptides |

Protein Coverage:

```
  1 MKVLRFFAPV LCLLWLGQVC LAVDIEEEEG VLVLKSANFD QALEQYPNIL VEFYAPWCGH CKALAPEYVK AAAKLLSENS
 81 EIRLAKVDAT EESELAQQFG VRGYPTIKFF KNGDKSSPKE YTAGREADDI LNWLKKRTGP AATTLADVAA AEELVESNEV
161 AVIGFFKDAE SDVAKEFLLA AEAIDDIPFG ITSKSDIFAK YQLKKDGVAL FKKFDEGRNN FDGEITKDNL LNFIKSNQLP
241 LVIEFTEQTA PKIFGGEIKT HILLFLPKSV EEYQGKLDNF KAAAEDFRGK ILFIYIDSDH SDNQRILEFF GLKKEECPAI
321 RLITLEEEMT KYKPESDELT PENIRDFCHK FLEGKVKPHL MSQEISDDWD KQPVKVLVGK NFEEVAFDEN KNVFVEFYAP
401 WCGHCKQLAP IWDKLGETYK DHENIIIAKM DSTVNEVEVV KVHSFPTLKY FPAGPDRTVV DYNGERTLEG FKKFLESGGK
481 DGGVDENDLE DLEDAEEEPD VEEEEEEETG AKKDEL
```

Supporting Peptides:

Peptide	Uniq	-10lgP	Mass	Length	ppm	m/z	z	RT	Fraction	Scan	Source File	Area F1	Area F2	Area F3	Area F4	Area F5	Area F6	Are
K.LLSENSEIR.L	Y	50.02	1059.5560	9	0.3	530.7855	2	30.07	7	F7:7892	17112021_RID_2349_DP7.raw							1.33

total 1 peptides

TRINITY_DN159
back to list

| Protein Coverage | Supporting Peptides |
Protein Coverage:

```
  1 MKLLRFFAPV LCLLWLGQVC LAVDIEEEEG VLVLKAANFD QALEQYPNIL VEFYAPWCGH CKALAPEYVK AAAKLLSENS
 81 EIRLAKVDAT EESELAQQFG VRGYPTIKFF KNGDKSSPKE YTAGREADDI LNWLKKRTGP AATTLADVAA AEELVESNEV
161 AVIGFFKDVE SDMAKEFLLA AEAIDDIPFG ITSKSDIFAK YQLKKDGVAL FKKFDEGRNN FDGEITKDNL LNFIKSNQLP
241 LVIEFTEQTA PKIFGGEIKT HILLFLPKSV EEYQGKLDNF KKAAEDFKGK ILFIYIDGDH SDNQRILEFF GLKKEECPAI
321 RLITLEEEMT KYKPESDELT PENIKDFCHK FLEGKVKVIV LFFPNGVFGA GSHVGKS
```

Supporting Peptides:

Peptide	Uniq	-10lgP	Mass	Length	ppm	m/z	z	RT	Fraction	Scan	Source File	Area F1	Area F2	Area F3	Area F4	Area F5	Area F6	Are
K.LLSENSEIR.L	Y	50.02	1059.5560	9	0.3	530.7855	2	30.07	7	F7:7892	17112021_RID_2349_DP7.raw							1.33

total 1 peptides

TRINITY_DN159
back to list

| Protein Coverage | Supporting Peptides |
Protein Coverage:

```
  1 MKLLRFFAPV LCLLWLGQVC LAVDIEEEEG VLVLKAANFD QALEQYPNIL VEFYAPWCGH CKALAPEYVK AAAKLLSENS
 81 EIRLAKVDAT EESELAQQFG VRGYPTIKFF KNGDKSSPKE YTAGREADDI LNWLKKRTGP AATTLADVAA AEELVESNEV
161 AVIGFFKDVE SDMAKEFLLA AEAIDDIPFG ITSKSDIFAK YQLKKDGVAL FKKFDEGRNN FDGEITKDNL LNFIKSNQLP
241 LVIEFTEQTA PKIFGGEIKT HILLFLPKSV EEYQGKLDNF KKAAEDFKGK ILFIYIDGDH SDNQRILEFF GLKKEECPAI
321 RLITLEEEMT KYKPESDELT PENIKDFCHK FLEGKVKPHL MSQEISDDWD KHPVKVLVGN NFEEVAFDEN KNVFVEFYAP
401 WCGHCKQLAP IWDKLGETYK DHENIIIAKM DATVNEVEAV KVHSFPTLKY FPAGPDRTVS QLYIET
```

Supporting Peptides:

Peptide	Uniq	-10lgP	Mass	Length	ppm	m/z	z	RT	Fraction	Scan	Source File	Area F1	Area F2	Area F3	Area F4	Area F5	Area F6	Are
K.LLSENSEIR.L	Y	50.02	1059.5560	9	0.3	530.7855	2	30.07	7	F7:7892	17112021_RID_2349_DP7.raw							1.33

total 1 peptides

TRINITY_DN159
back to list

| Protein Coverage | Supporting Peptides |
Protein Coverage:

```
  1 MEGIGRKYTI HLMLCLLDVQ FFPPTDAPWC GHCKALAPEY VKAAAKLLSE NSEIRLAKVD ATEESELAQQ FGVRGYPTIK
 81 FFKNGDKSSP KEYTAGREAD DILNWLKKRT GPAATTLADV AAAEELVESN EVAVIGFFKD VESDMAKEFL LAAEAIDDIP
161 FGITSKSDIF AKYQLKKDGV ALFKKFDEGR NNFDGEITKD NLLNFIKSNQ LPLVIEFTEQ TAPKIFGGEI KTHILLFLPK
241 SVEEYQGKLD NFKKAAEDFK GKILFIYIDG DHSDNQRILE FFGLKKEECP AIRLITLEEE MTKYKPESDE LTPENIKDFC
321 HKFLEGKVKP HLMSQEISDD WDKHPVKVLV GNNFEEVAFD ENKNVFVEFY APWCGHCKQL APIWDKLGET YKDHENIIIA
401 KMDATVNEVE AVKVHSFPTL KYFPAGPDRT VVDYNGDRTL EGFKKFLESG GKDGGVDEND LEDLEDAEAE PDVEEEDEET
481 GAKKDEL
```

Supporting Peptides:

Peptide	Uniq	-10lgP	Mass	Length	ppm	m/z	z	RT	Fraction	Scan	Source File	Area F1	Area F2	Area F3	Area F4	Area F5	Area F6	Are
K.LLSENSEIR.L	Y	50.02	1059.5560	9	0.3	530.7855	2	30.07	7	F7:7892	17112021_RID_2349_DP7.raw							1.33

total 1 peptides

AFJ50884.1

back to list

| Protein Coverage | Supporting Peptides |

Protein Coverage:

1 MKVLRFFAPV LCLLWLGQVC LAVDIEEEEG VLVLKSANFD QALEQYPNIL VEFYAPWCGH CKALAPEYVK AAAK**LLSENS**

81 **EIR**LAKVDAT EESELAQQFG VRGYPTIKFF KNGDKSSPKE YTAGREADDI LNWLKKRTGP AATTLADVAA AEELVESNEV

161 AVIGFFKDVE SDVAKEFLLA AEAIDDIPFG ITSKSDIFAK YQLKKDGVAL FKKFDEGRNN FDGEITKDNL LNFIKSNQLP

241 LVIEFTEQTA PKIFGGEIKT HILLFLPKSV EEYQGKLDNF KTAAEDFRGK ILFIYIDSDH SDNQRILEFF GLKKEECPAI

321 RLITLEEEMT KYKPESDELT PENIRDFCHK FLEGKVKPHL MSQEISDDWD KQPVKVLVGK NFEEVAFDEN KNVFVEFYAP

401 WCGHCKQLAP IWDKLGETYK DHENIIIAKM DSTVNEVEVV KVHSFPTLKY FPAGPDRTVV DYNGERTLEG FKKFLESGGK

481 DGGVDENDLE DLEDAEEEPD VEEEEEEEET GAKKDEL

Supporting Peptides:

Peptide	Uniq	-10lgP	Mass	Length	ppm	m/z	z	RT	Fraction	Scan	Source File	Area F1	Area F2	Area F3	Area F4	Area F5	Area F6	Are
K.LLSENSEIR.L	Y	50.02	1059.5560	9	0.3	530.7855	2	30.07	7	F7:7892	17112021_RID_2349_DP7.raw							1.33

total 1 peptides

JAI12151.1

back to list

| Protein Coverage | Supporting Peptides |

Protein Coverage:

1 MKVLRFFAPV LCLLWLGQVC LAVDIEEEEG VLVLKSANFD QALEQYPNIL VEFYAPWCGH CKALAPEYVK AAAK**LLSENS**

81 **EIR**LAKVDAT EESELAQQFG VRGYPTIKFF KNGDKSSPKE YTAGREADDI LNWLKKRTGP AATTLADVAA AEELVESNEV

161 AVIGFFKDVE SDVAKEFLLA AEAIDDIPFG ITSKSDIFAK YQLKKDGVAL FKKFDEGRNN FDGEITKDNL LNFIKSNQLP

241 LVIEFTEQTA PKIFGGEIKT HILLFLPKSV EEYQGKLDNF KTAAEDFRGK ILFIYIDSDH SDNQRILEFF GLKKEECPAI

321 RLITLEEEMT KYKPESDELT PENIRDFCHK FLEGKVKPHL MSQEISDDWD KQPVKVLVGK NFEEVAFDEN KNVFVEFYAP

401 WCGHCKQLAP IWDKLGETYK DHENIIIAKM DSTVNEVEVV KVHSFPTLKY FPAGPDRTVV DYNGERTLEG FKKFLESGGK

481 DGGVDENDLE DLEDAEEEPD VEEEEEEEET GAKKDEL

Supporting Peptides:

Peptide	Uniq	-10lgP	Mass	Length	ppm	m/z	z	RT	Fraction	Scan	Source File	Area F1	Area F2	Area F3	Area F4	Area F5	Area F6	Are
K.LLSENSEIR.L	Y	50.02	1059.5560	9	0.3	530.7855	2	30.07	7	F7:7892	17112021_RID_2349_DP7.raw							1.33

total 1 peptides

JAG67907.1

back to list

| Protein Coverage | Supporting Peptides |

Protein Coverage:

1 MVK**VGVNGFG R**IGRLVTRAA FVSGKVEVVA INDPFIDLNY MTYMFKYDST HGRFHGTVHA ENGKLVINGH AITVFQERDP

81 ANIKWGEAGA DYVVESTGVF TTTEKASAHL KGGAKRVIIS APSADAPMFV MGVNHEKYDK SLKVVSNASC TTNCLAPLAK

161 VIHDNYGIVE GLMTTVHAIT ATQKTVDGPS GKLWRDGRGA AQNIIPASTG AAKAVGKVIP ELNGKLTGMA FRVPTPNVSV

241 VDLTCRLEKP AKYDDIKKVV KAASEGPMKG ILGYTEDQVV SSDFNGDTHS SIFDAAAGIA LNDHFVKLIS WYDNEYGYSN

321 RVVDLMVHMA SKE

Supporting Peptides:

Peptide	Uniq	-10lgP	Mass	Length	ppm	m/z	z	RT	Fraction	Scan	Source File	Area F1	Area F2	Area F3	Area F4	Area F5	Area F
K.VGVNGFGR.I	Y	49.83	804.4242	8	0.7	403.2197	2	30.24	4	F4:7860	17112021_RID_2349_DP4.raw				1.365E5		1.3546E

total 1 peptides

TRINITY_DN3991

back to list

| Protein Coverage | Supporting Peptides |

Protein Coverage:

1 MVK**VGVNGFG R**IGRLVTRAA ILRGNVEVVA INDPFIDLNY MVYMFKYDST HGRWKGDVKA EGGKLVIDGH AITIFQERDP

81 SNIKWGEAGA TYVVESTGVF TTVEKASTHL KGGAKKVIIS APSADAPMFV VGVNHQKYDP AVNVVSNASC TTNCLAPLAK

161 VINDNFVILE GLMTTVHAYT ATQKTVDGPS GKLWRDGRGA HQNIIPASTG AAKAVGKVIP ELNGKITGMA FRVPTPNVSV

241 VDLTVRLEKP ATYDAIKAKV KEAANGPMKG ILGYTEDQVV STDFNGDTRS SIFDAGAGIA LNDHFVKLVT WYDNEFGYSN

321 RVVDLVLYMV SKDK

Supporting Peptides:

Peptide	Uniq	-10lgP	Mass	Length	ppm	m/z	z	RT	Fraction	Scan	Source File	Area F1	Area F2	Area F3	Area F4	Area F5	Area F
K.VGVNGFGR.I	Y	49.83	804.4242	8	0.7	403.2197	2	30.24	4	F4:7860	17112021_RID_2349_DP4.raw				1.365E5		1.3546E

total 1 peptides

CAJ01683.1
back to list

| Protein Coverage | Supporting Peptides |
Protein Coverage:

1 MMQVLLVTIC LAVFPYEGSS IILESGNVND YEIVYPQKVT AMPIEAILQP EQKYEDVMQY EFEVNGEPVV LHLEKNKDLF
81 SEDYSETYYT PDGREITTNL PVEDHCYYHG RLQNDAHSSA IISACNGLKG HFKLRGETYL IEPLKVPDSE AHAVYKYENI
161 EKEDEAPKMC GVTQTNWESD EPIKEASQLF ATSEQKSYYD RFRHIELVIV VDHRMVEKYN GNLRKIRRRI YQIVNILNQI
241 YIFMNIRVPL VGVEFWTNGD LINVTSEAEA TLNSFGEWRA SYLLSRKNHD HAQLFTAIDL EGLTIGMAPM AGMCQSERSV
321 GIFEDYSPVD HVVAVIIAHE MGHNLGIFHD VHQCNCGANS CIMYAKISNP PPMYFSDCSQ EQYQFFLDNY KPDCTLIRPP
401 R**TDIVSPPVC GNDLLEK**GEE CDCGSPENCQ NPCCDAASCK LHSWIECEFG ECCEQCRFKP AGTECRGIRN ECDLPEYCTG
481 QSAECPIDRS HRNGKPCLNN YGYCYNGTCP IMYHQCYALF GPKAVVGQDV CFEENKRGES YFYCRKENDV KIPCAPEDIK
561 CGRLFCRHDI YECRYDYSEN PNYGMVEEGT KCGDGKVCSK RHCVDVTTAY

Supporting Peptides:

Peptide	Uniq	-10lgP	Mass	Length	ppm	m/z	z	RT	Fraction	Scan	Source File	Area F1	Area F2	Are F3
R.TDIVSPPVC(+57.02)GNDLLEK.G	Y	48.76	1755.8712	16	0.4	878.9432	2	46.31	3	F3:13400	17112021_RID_2349_DP3.raw			2.178

ADW54336.1
back to list

| Protein Coverage | Supporting Peptides |
Protein Coverage:

1 MMQVLLVTIC LAVFPYEGSS IILESGNVND YEIVYPQKVT AMPIEAILQP EQKYEDVMQY EFEVNGEPVV LHLEKNKDLF
81 SEDYSETYYT PDGREITTNL PVEDHCYYHG RLQNDAHSSA IISAXHGLKG HFKLRGETYL IEPLKVPDSX XXXXXXXXXX
161 XXXXXXXXXX XXXXXXXXXX XXXXXXXXXX XXXXXXXXXX XXXXXXXXXX XXXXXXXXXX XXXXXXXXXX XXXXXXXXXX
241 XXXXXXXXXX XXXXXXXIRR RIYQIVNILN QIYIFMNIRV PLVGVEFWTN GDLINVTSEA EATLNSFGEW RASYLLSRKN
321 HDHAQLFTAI DLEGLTIGMA PMAGMCQSER SVGIFEDYSP VDHVVAVIIA HEMGHNLGIF HDVHQCNCGA NSCIMYAKIS
401 NPPPMYFSDC SQEQYQFFLD NYKPDCTLIR PPR**TDIVSPP VCGNDLLEK**G EECDCGSPEN CQNPCCDAAS CKLHSWIECE
481 FGECCEQCRF KPAGTECRGI RSECDLPEYC TGQSLDCPID HFHRNGKPCQ NNNGYCYNGI CPTMQNQCLA HFGPDAVMAQ
561 DGCFEWNKKG EGDFYCRKEN NVTIPCASED IKCGRLFCEI VPNICRRAYG DKGMVDPGTK CEDGKVCINR KCTNVNTAY

Supporting Peptides:

Peptide	Uniq	-10lgP	Mass	Length	ppm	m/z	z	RT	Fraction	Scan	Source File	Area F1	Area F2	Are F3
R.TDIVSPPVC(+57.02)GNDLLEK.G	Y	48.76	1755.8712	16	0.4	878.9432	2	46.31	3	F3:13400	17112021_RID_2349_DP3.raw			2.178

ADW54337.1
back to list

| Protein Coverage | Supporting Peptides |
Protein Coverage:

1 QLFATSEQKS YYDRFRHIEL VIVVDHRMVE KYNGNLRKIR RRIYQIVNIL NQIYIFMNIR VPLVGVEFWT NGDLINVTSE
81 AEATLNSFGE WRASYLLSRK NHDHAQLFTA IDLEGLTIGM APMAGMCQSE RSVGIFEDYS PVDHVVAVII AHEMGHNLGI
161 FHDVHQCNCG ANSCIMYAKI SNPPPMYFSD CSQEQYQFFL DNYKPDCTLI RPPR**TDIVSP PVCGNDLLEK** GEECDCGSPE
241 NCQNPCCDAA SCKLHSWIEC EFGECCEPCR FKPAGTECRG IRSECDLPEY CTGQSLDCPI DHFHRNGKPC QNNNGYCYNG
321 ICPTMQNQCL AHFGPDAVMA QDGCFEWNKK GEGDFYCRKE NNVTIPCASE DIKCGRLFCE IVPNICRRAY GDKGMVDPGT
401 KCEDGKVCIN RKCTNVNTAY

Supporting Peptides:

Peptide	Uniq	-10lgP	Mass	Length	ppm	m/z	z	RT	Fraction	Scan	Source File	Area F1	Area F2	Are F3
R.TDIVSPPVC(+57.02)GNDLLEK.G	Y	48.76	1755.8712	16	0.4	878.9432	2	46.31	3	F3:13400	17112021_RID_2349_DP3.raw			2.178

ADW54335.1
back to list

| Protein Coverage | Supporting Peptides |
Protein Coverage:

1 MVEKYNGNLR KIRRRIYQIV NILNQIYIFM NIRVPLVGVE FWTNGDLINV TSEAEATLNS FGEWRASYLL SRKNHDHAQL
81 FTAIDLEGLT IGMAPMAGMC QSERSVGIFE DYSPVDHVVA VIIAHEMGHN LGIFHDVHQC NCGANSCIMY AKIREPPPMY
161 FSDCSQEQYQ FFLDNYKPDC TLIRPPR**TDI VSPPVCGNDL LEK**GEECDCG SPENCQNPCC DAASCKLHSW IECEFGECCE
241 QCRFKPAGTE CRGIRSECDL PEHCTGQSLD CPIDHFHRXG KPCQTNXGYX YNGICPTMXN QCLXHFGPXA VXAQXGXFEW
321 XXKGXGDFXC RKENXVTLPX ASEDIKRGRL XCEIVPNICR

Supporting Peptides:

Peptide	Uniq	-10lgP	Mass	Length	ppm	m/z	z	RT	Fraction	Scan	Source File	Area F1	Area F2	Are F3

Peptide	Uniq	-10lgP	Mass	Length	ppm	m/z	z	RT	Fraction	Scan	Source File	Area F1	Area F2	Are F3
R.TDIVSPPVC(+57.02)GNDLLEK.G	Y	48.76	1755.8712	16	0.4	878.9432	2	46.31	3	F3:13400	17112021_RID_2349_DP3.raw			2.178

P86541.2
back to list

| Protein Coverage | Supporting Peptides |
Protein Coverage:

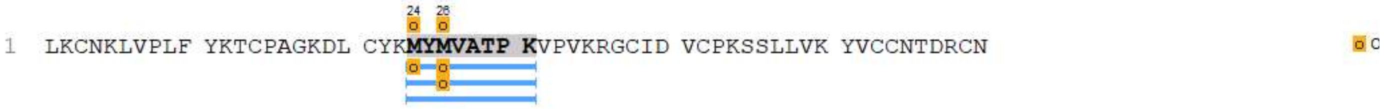

Supporting Peptides:

Peptide	Uniq	-10lgP	Mass	Length	ppm	m/z	z	RT	Fraction	Scan	Source File	Area F1	Area F2	A 1
K.MYMVATPK.V	Y	47.76	939.4558	8	0.4	470.7354	2	34.94	7	F7:9610	17112021_RID_2349_DP7.raw			
K.M(+15.99)YM(+15.99)VATPK.V	Y	46.26	971.4456	8	0.1	486.7301	2	25.61	6	F6:6222	17112021_RID_2349_DP6.raw	1.4689E5		
K.MYM(+15.99)VATPK.V	Y	45.65	955.4507	8	0.4	478.7328	2	30.13	2	F2:7655	12112021_RID_2349_DP2.raw	4.4599E4	2.2353E4	

P01440.1
back to list

| Protein Coverage | Supporting Peptides |
Protein Coverage:

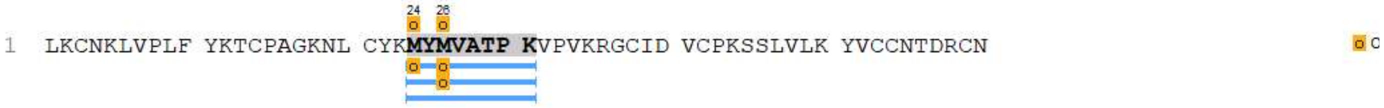

Supporting Peptides:

Peptide	Uniq	-10lgP	Mass	Length	ppm	m/z	z	RT	Fraction	Scan	Source File	Area F1	Area F2	A 1
K.MYMVATPK.V	Y	47.76	939.4558	8	0.4	470.7354	2	34.94	7	F7:9610	17112021_RID_2349_DP7.raw			
K.M(+15.99)YM(+15.99)VATPK.V	Y	46.26	971.4456	8	0.1	486.7301	2	25.61	6	F6:6222	17112021_RID_2349_DP6.raw	1.4689E5		
K.MYM(+15.99)VATPK.V	Y	45.65	955.4507	8	0.4	478.7328	2	30.13	2	F2:7655	12112021_RID_2349_DP2.raw	4.4599E4	2.2353E4	

AAC61315.1
back to list

| Protein Coverage | Supporting Peptides |
Protein Coverage:

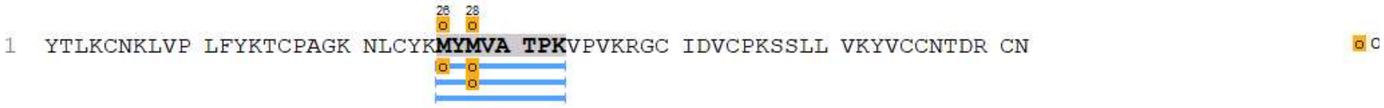

Supporting Peptides:

Peptide	Uniq	-10lgP	Mass	Length	ppm	m/z	z	RT	Fraction	Scan	Source File	Area F1	Area F2	A 1
K.MYMVATPK.V	Y	47.76	939.4558	8	0.4	470.7354	2	34.94	7	F7:9610	17112021_RID_2349_DP7.raw			
K.M(+15.99)YM(+15.99)VATPK.V	Y	46.26	971.4456	8	0.1	486.7301	2	25.61	6	F6:6222	17112021_RID_2349_DP6.raw	1.4689E5		
K.MYM(+15.99)VATPK.V	Y	45.65	955.4507	8	0.4	478.7328	2	30.13	2	F2:7655	12112021_RID_2349_DP2.raw	4.4599E4	2.2353E4	

BAU24665.1
back to list

| Protein Coverage | Supporting Peptides |
Protein Coverage:

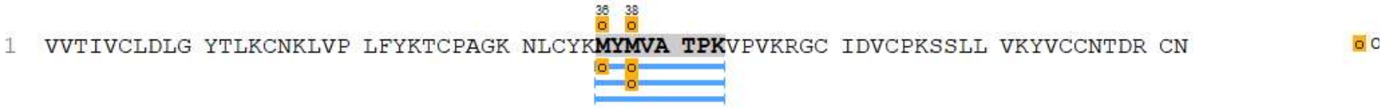

Supporting Peptides:

Peptide	Uniq	-10lgP	Mass	Length	ppm	m/z	z	RT	Fraction	Scan	Source File	Area F1	Area F2	A 1

Peptide	Uniq	-10lgP	Mass	Length	ppm	m/z	z	RT	Fraction	Scan	Source File	Area F1	Area F2	A 1
K.MYMVATPK.V	Y	47.76	939.4558	8	0.4	470.7354	2	34.94	7	F7:9610	17112021_RID_2349_DP7.raw			
K.M(+15.99)YM(+15.99)VATPK.V	Y	46.26	971.4456	8	0.1	486.7301	2	25.61	6	F6:6222	17112021_RID_2349_DP6.raw	1.4689E5		
K.MYM(+15.99)VATPK.V	Y	45.65	955.4507	8	0.4	478.7328	2	30.13	2	F2:7655	12112021_RID_2349_DP2.raw	4.4599E4	2.2353E4	

O93472.1
back to list

| Protein Coverage | Supporting Peptides |
Protein Coverage:

45 47
1 MKTLLLTLVL VTIVCLDLGY TLKCNKLVPL FYKTCPAGKN LCYK**MYMVAT PK**VPVKRGCI DVYPKSSLLV KYVCCNTDRC

81 N

Supporting Peptides:

Peptide	Uniq	-10lgP	Mass	Length	ppm	m/z	z	RT	Fraction	Scan	Source File	Area F1	Area F2	A 1
K.MYMVATPK.V	Y	47.76	939.4558	8	0.4	470.7354	2	34.94	7	F7:9610	17112021_RID_2349_DP7.raw			
K.M(+15.99)YM(+15.99)VATPK.V	Y	46.26	971.4456	8	0.1	486.7301	2	25.61	6	F6:6222	17112021_RID_2349_DP6.raw	1.4689E5		
K.MYM(+15.99)VATPK.V	Y	45.65	955.4507	8	0.4	478.7328	2	30.13	2	F2:7655	12112021_RID_2349_DP2.raw	4.4599E4	2.2353E4	

Q9PST3.1
back to list

| Protein Coverage | Supporting Peptides |
Protein Coverage:

45 47
1 MKTLLLTLVV VTTVCLDLGY TLKCNKLVPL FYKTCPAGKN LCYK**MYMVAT PK**VPVKRGCI DVCPKSSLLV KYVCCNTDRC

81 N

Supporting Peptides:

Peptide	Uniq	-10lgP	Mass	Length	ppm	m/z	z	RT	Fraction	Scan	Source File	Area F1	Area F2	A 1
K.MYMVATPK.V	Y	47.76	939.4558	8	0.4	470.7354	2	34.94	7	F7:9610	17112021_RID_2349_DP7.raw			
K.M(+15.99)YM(+15.99)VATPK.V	Y	46.26	971.4456	8	0.1	486.7301	2	25.61	6	F6:6222	17112021_RID_2349_DP6.raw	1.4689E5		
K.MYM(+15.99)VATPK.V	Y	45.65	955.4507	8	0.4	478.7328	2	30.13	2	F2:7655	12112021_RID_2349_DP2.raw	4.4599E4	2.2353E4	

AAK49439.1
back to list

| Protein Coverage | Supporting Peptides |
Protein Coverage:

45 47
1 MKTLLLTLVV VTIVCLDLGY TLKCNKLVPL FYKTCPAGKN LCYK**MYMVAT PK**VPVKRGCI DVCPKSSLLV KYVCCNTDRC

81 N

Supporting Peptides:

Peptide	Uniq	-10lgP	Mass	Length	ppm	m/z	z	RT	Fraction	Scan	Source File	Area F1	Area F2	A 1
K.MYMVATPK.V	Y	47.76	939.4558	8	0.4	470.7354	2	34.94	7	F7:9610	17112021_RID_2349_DP7.raw			
K.M(+15.99)YM(+15.99)VATPK.V	Y	46.26	971.4456	8	0.1	486.7301	2	25.61	6	F6:6222	17112021_RID_2349_DP6.raw	1.4689E5		
K.MYM(+15.99)VATPK.V	Y	45.65	955.4507	8	0.4	478.7328	2	30.13	2	F2:7655	12112021_RID_2349_DP2.raw	4.4599E4	2.2353E4	

AAC27686.1
back to list

| Protein Coverage | Supporting Peptides |
Protein Coverage:

45 47

1 MKTLLLTLVV VTTVCLDLGY TLKCNKLVPL FYKTCPAGKN LCYK**MYMVAT PK**VPVKRGCI DVCPKSSLLV KYVCCNTDRC C

81 N

Supporting Peptides:

Peptide	Uniq	-10lgP	Mass	Length	ppm	m/z	z	RT	Fraction	Scan	Source File	Area F1	Area F2	A
K.MYMVATPK.V	Y	47.76	939.4558	8	0.4	470.7354	2	34.94	7	F7:9610	17112021_RID_2349_DP7.raw			
K.M(+15.99)YM(+15.99)VATPK.V	Y	46.26	971.4456	8	0.1	486.7301	2	25.61	6	F6:6222	17112021_RID_2349_DP6.raw	1.4689E5		
K.MYM(+15.99)VATPK.V	Y	45.65	955.4507	8	0.4	478.7328	2	30.13	2	F2:7655	12112021_RID_2349_DP2.raw	4.4599E4	2.2353E4	

Q9PST4.1
back to list

| Protein Coverage | Supporting Peptides |
Protein Coverage:

45 47

1 MKTLLLTLVV VTIVCLDLGY TLKCNKLVPL FYKTCPAGKN LCYK**MYMVAT PK**VPVKRGCI DVCPKSSLLV KYVCCNTDRC C

81 N

Supporting Peptides:

Peptide	Uniq	-10lgP	Mass	Length	ppm	m/z	z	RT	Fraction	Scan	Source File	Area F1	Area F2	A
K.MYMVATPK.V	Y	47.76	939.4558	8	0.4	470.7354	2	34.94	7	F7:9610	17112021_RID_2349_DP7.raw			
K.M(+15.99)YM(+15.99)VATPK.V	Y	46.26	971.4456	8	0.1	486.7301	2	25.61	6	F6:6222	17112021_RID_2349_DP6.raw	1.4689E5		
K.MYM(+15.99)VATPK.V	Y	45.65	955.4507	8	0.4	478.7328	2	30.13	2	F2:7655	12112021_RID_2349_DP2.raw	4.4599E4	2.2353E4	

AAC27687.1
back to list

| Protein Coverage | Supporting Peptides |
Protein Coverage:

45 47

1 MKTLLLTLVL VTIVCLDLGY TLKCNKLVPL FYKTCPAGKN LCYK**MYMVAT PK**VPVKRGCI DVYPKSSLLV KYVCCNTDRC C

81 N

Supporting Peptides:

Peptide	Uniq	-10lgP	Mass	Length	ppm	m/z	z	RT	Fraction	Scan	Source File	Area F1	Area F2	A
K.MYMVATPK.V	Y	47.76	939.4558	8	0.4	470.7354	2	34.94	7	F7:9610	17112021_RID_2349_DP7.raw			
K.M(+15.99)YM(+15.99)VATPK.V	Y	46.26	971.4456	8	0.1	486.7301	2	25.61	6	F6:6222	17112021_RID_2349_DP6.raw	1.4689E5		
K.MYM(+15.99)VATPK.V	Y	45.65	955.4507	8	0.4	478.7328	2	30.13	2	F2:7655	12112021_RID_2349_DP2.raw	4.4599E4	2.2353E4	

AAC27685.1
back to list

| Protein Coverage | Supporting Peptides |
Protein Coverage:

45 47

1 MKTLLLTLVV VTIVCLDLGY TLKCNKLVPL FYKTCPAGKN LCYK**MYMVAT PK**VPVKRGCI DVCPKSSLLV KYVCCNTDRC C

81 N

Supporting Peptides:

Peptide	Uniq	-10lgP	Mass	Length	ppm	m/z	z	RT	Fraction	Scan	Source File	Area F1	Area F2	A
K.MYMVATPK.V	Y	47.76	939.4558	8	0.4	470.7354	2	34.94	7	F7:9610	17112021_RID_2349_DP7.raw			
K.M(+15.99)YM(+15.99)VATPK.V	Y	46.26	971.4456	8	0.1	486.7301	2	25.61	6	F6:6222	17112021_RID_2349_DP6.raw	1.4689E5		

Peptide	Uniq	-10lgP	Mass	Length	ppm	m/z	z	RT	Fraction	Scan	Source File	Area F1	Area F2	A 1
K.MYM(+15.99)VATPK.V	Y	45.65	955.4507	8	0.4	478.7328	2	30.13	2	F2:7655	12112021_RID_2349_DP2.raw	4.4599E4	2.2353E4	

ETE69494.1
back to list

| Protein Coverage | Supporting Peptides |
Protein Coverage:

```
  1 MAGGKAGKDS GKTKTKTVSR SQRAGLQFPV GRIHRHLKSR TTSHGRVGAT AAVYSAAILE YLTAEVLELA GNASKDLKVK
 81 RITPRHLQLA IRGDEELDSL IKATIAGGVL LEAIMKNGSA AGSGTHCRKP ASKNDKSKTN SMKDSSASAA GEIGKTYTKE
161 QIEGVQSIKK CKNYYEVLGV SKDAGEEDLK KAYRKLALKF HPDKNHAPGA TEAFKKIGNA YAVLSNPEKR KQYDLTGSED
241 PCNQPGNGRF NFHRGCEADI TPEDLFNMFF GGAFPTGSVH SFSNGRSGFS HHNQHRHSGH EREEERGDGG FSMFIQLMPI
321 IVLIFVSLLS QLMVSNPPYS LYPRSSSGQT IQMQTENLGV VYYVNKDFKN EYKGPNLQKV EKSVEEDYVS NIRNNCWKER
401 QQKTDLLYAA KVYRDDRLRK KAEAISMDNC KELERLTSIY RGG
```

Supporting Peptides:

Peptide	Uniq	-10lgP	Mass	Length	ppm	m/z	z	RT	Fraction	Scan	Source File	Area F1	Area F2	Area F3	Area F4	Area F5	A
R.AGLQFPVGR.I	Y	47.54	943.5239	9	0.3	472.7694	2	42.70	5	F5:12209	17112021_RID_2349_DP5.raw				4.76E4	5.9883E4	9.1

total 1 peptides

ETE65060.1
back to list

| Protein Coverage | Supporting Peptides |
Protein Coverage:

```
  1 MSGRGKQGGK ARAKAKSRSS RAGLQFPVGR VHRLLRKGNY AERVGAGAPV YLAAVLEYLT AEILELAGNA ARDNKKTRII
 81 PRHLQLAVRN DEELNKLLGG VTIAQGGVLP NIQAVLLPKK TESHKAKGK
```

Supporting Peptides:

Peptide	Uniq	-10lgP	Mass	Length	ppm	m/z	z	RT	Fraction	Scan	Source File	Area F1	Area F2	Area F3	Area F4	Area F5	A
R.AGLQFPVGR.V	Y	47.54	943.5239	9	0.3	472.7694	2	42.70	5	F5:12209	17112021_RID_2349_DP5.raw				4.76E4	5.9883E4	9.

total 1 peptides

XP_013915314.1
back to list

| Protein Coverage | Supporting Peptides |
Protein Coverage:

```
  1 MSGRGKQGGK ARAKAKSRSS RAGLQFPVGR VHRLLRKGNY AERVGAGAPV YLAAVLEYLT AEILELAGNA ARDNKKTRII
 81 PRHLQLAVRN DEELNKLLGG VTIAQGGVLP NIQAVLLPKK TESHKAKGK
```

Supporting Peptides:

Peptide	Uniq	-10lgP	Mass	Length	ppm	m/z	z	RT	Fraction	Scan	Source File	Area F1	Area F2	Area F3	Area F4	Area F5	A
R.AGLQFPVGR.V	Y	47.54	943.5239	9	0.3	472.7694	2	42.70	5	F5:12209	17112021_RID_2349_DP5.raw				4.76E4	5.9883E4	9.

total 1 peptides

XP_013919189.1
back to list

| Protein Coverage | Supporting Peptides |
Protein Coverage:

```
  1 MSGRGKSGGK ARAKAKSRSS RAGLQFPVGR VHRLLRKGNY AERVGAGAPV YLAAVLEYLS AEILELAGNA ARDNKKTRII
 81 PRHLQLAIRN DEELNKLLGG VTIAQGGVLP NIQAVLLPKK TQSSKK
```

Supporting Peptides:

Peptide	Uniq	-10lgP	Mass	Length	ppm	m/z	z	RT	Fraction	Scan	Source File	Area F1	Area F2	Area F3	Area F4	Area F5	A
R.AGLQFPVGR.V	Y	47.54	943.5239	9	0.3	472.7694	2	42.70	5	F5:12209	17112021_RID_2349_DP5.raw				4.76E4	5.9883E4	9.

total 1 peptides

XP_013919188.1
back to list

| Protein Coverage | Supporting Peptides |
Protein Coverage:

```
  1 MSGRGKSGGK ARAKAKSRSS RAGLQFPVGR VHRLLRKGNY AERVGAGAPV YLAAVLEYLS AEILELAGNA ARDNKKTRII
 81 PRHLQLAIRN DEELNKLLGG VTIAQGGVLP NIQAVLLPKK TQSSKK
```

Supporting Peptides:

Peptide	Uniq	-10lgP	Mass	Length	ppm	m/z	z	RT	Fraction	Scan	Source File	Area F1	Area F2	Area F3	Area F4	Area F5	A

total 1 peptides

Peptide	Uniq	-10lgP	Mass	Length	ppm	m/z	z	RT	Fraction	Scan	Source File	Area F1	Area F2	Area F3	Area F4	Area F5	A
R.AGLQFPVGR.V	Y	47.54	943.5239	9	0.3	472.7694	2	42.70	5	F5:12209	17112021_RID_2349_DP5.raw				4.76E4	5.9883E4	9.

total 1 peptides

ETE65301.1
back to list

| Protein Coverage | Supporting Peptides |
Protein Coverage:

1 MSGRGKSGGK ARAKAKSRSS R**AGLQFPVGR** VHRLLRKGNY AERVGAGAPV YLAAVLEYLS AEILELAGNA ARDNKKTRII

81 PRHLQLAIRN DEELNKLLGG VTIAQGGVLP NIQAVLLPKK TQSSKK

Supporting Peptides:

Peptide	Uniq	-10lgP	Mass	Length	ppm	m/z	z	RT	Fraction	Scan	Source File	Area F1	Area F2	Area F3	Area F4	Area F5	A
R.AGLQFPVGR.V	Y	47.54	943.5239	9	0.3	472.7694	2	42.70	5	F5:12209	17112021_RID_2349_DP5.raw				4.76E4	5.9883E4	9.

total 1 peptides

JAG46644.1
back to list

| Protein Coverage | Supporting Peptides |
Protein Coverage:

1 MAGGKAGKDS GKAKAKAVSR SQR**AGLQFPV GR**IHRHLKTR TTSHGRVGAT AAVYSAAILE YLTAEVLELA GNASKDLKVK

81 RITPRHLQLA IRGDEELDSL IKATIAGGGV IPHIHKSLIG KKGQQKTA

Supporting Peptides:

Peptide	Uniq	-10lgP	Mass	Length	ppm	m/z	z	RT	Fraction	Scan	Source File	Area F1	Area F2	Area F3	Area F4	Area F5	A
R.AGLQFPVGR.I	Y	47.54	943.5239	9	0.3	472.7694	2	42.70	5	F5:12209	17112021_RID_2349_DP5.raw				4.76E4	5.9883E4	9.1

total 1 peptides

JAI09932.1
back to list

| Protein Coverage | Supporting Peptides |
Protein Coverage:

1 MAGGKAGKDS GKTKTKTVSR SQR**AGLQFPV GR**IHRHLKSR TTSHGRVGAT AAVYSAAILE YLTAEVLELA GNASKDLKVK

81 RITPRHLQLA IRGDEELDSL IKATIAGGGV IPHIHKSLIG KKGQQKTV

Supporting Peptides:

Peptide	Uniq	-10lgP	Mass	Length	ppm	m/z	z	RT	Fraction	Scan	Source File	Area F1	Area F2	Area F3	Area F4	Area F5	A
R.AGLQFPVGR.I	Y	47.54	943.5239	9	0.3	472.7694	2	42.70	5	F5:12209	17112021_RID_2349_DP5.raw				4.76E4	5.9883E4	9.1

total 1 peptides

XP_007430054.1
back to list

| Protein Coverage | Supporting Peptides |
Protein Coverage:

1 MAGGKAGKDS GKAKAKAVSR SQR**AGLQFPV GR**IHRHLKTR TTSHGRVGAT AAVYSAAILE YLTAEVLELA GNASKDLKVK

81 RITPRHLQLA IRGDEELDSL IKATIAGGGV IPHIHKSLIG KKGQQKTA

Supporting Peptides:

Peptide	Uniq	-10lgP	Mass	Length	ppm	m/z	z	RT	Fraction	Scan	Source File	Area F1	Area F2	Area F3	Area F4	Area F5	A
R.AGLQFPVGR.I	Y	47.54	943.5239	9	0.3	472.7694	2	42.70	5	F5:12209	17112021_RID_2349_DP5.raw				4.76E4	5.9883E4	9.1

total 1 peptides

JAI13025.1
back to list

| Protein Coverage | Supporting Peptides |
Protein Coverage:

1 MAGGKAGKDS GKTKTKTVSR SQR**AGLQFPV GR**IHRHLKSR TTSHGRVGAT AAVYSAAILE YLTAEVLELA GNASKDLKVK

81 RITPRHLQLA IRGDEELDSL IKATIAGGGV IPHIHKSLIG KKGQQKTV

Supporting Peptides:

Peptide	Uniq	-10lgP	Mass	Length	ppm	m/z	z	RT	Fraction	Scan	Source File	Area F1	Area F2	Area F3	Area F4	Area F5	A
R.AGLQFPVGR.I	Y	47.54	943.5239	9	0.3	472.7694	2	42.70	5	F5:12209	17112021_RID_2349_DP5.raw				4.76E4	5.9883E4	9.1

total 1 peptides

AFJ50305.1
back to list

| Protein Coverage | Supporting Peptides |
Protein Coverage:

1 MAGGKAGKDS GKTKTKTVSR SQRAGLQFPV GRIHRHLKSR TTSHGRVGAT AAVYSAAILE YLTAEVLELA GNASKDLKVK

81 RITPRHLQLA IRGDEELDSL IKATIAGGGV IPHIHKSLIG KKGQQKTV

Supporting Peptides:

Peptide	Uniq	-10lgP	Mass	Length	ppm	m/z	z	RT	Fraction	Scan	Source File	Area F1	Area F2	Area F3	Area F4	Area F5	A
R.AGLQFPVGR.I	Y	47.54	943.5239	9	0.3	472.7694	2	42.70	5	F5:12209	17112021_RID_2349_DP5.raw				4.76E4	5.9883E4	9.1

total 1 peptides

JAG44453.1
back to list

| Protein Coverage | Supporting Peptides |
Protein Coverage:

1 MAGGKAGKDS GKAKAKAVSR SQRAGLQFPV GRIHRHLKTR TTSHGRVGAT AAVYSAAILE YLTAEVLELA GNASKDLKVK

81 RITPRHLQLA IRGDEELDSL IKATIAGGGV IPHIHKSLIG KKGQQKTA

Supporting Peptides:

Peptide	Uniq	-10lgP	Mass	Length	ppm	m/z	z	RT	Fraction	Scan	Source File	Area F1	Area F2	Area F3	Area F4	Area F5	A
R.AGLQFPVGR.I	Y	47.54	943.5239	9	0.3	472.7694	2	42.70	5	F5:12209	17112021_RID_2349_DP5.raw				4.76E4	5.9883E4	9.1

total 1 peptides

JAI09933.1
back to list

| Protein Coverage | Supporting Peptides |
Protein Coverage:

1 MAGGKAGKDS GKAKAKAVSR SQRAGLQFPV GRIHRHLKTR TTSHGRVGAT AAVYSAAILE YLTAEVLELA GNASKDLKVK

81 RITPRHLQLA IRGDEELDSL IKATIAGGGV IPHIHKSLIG KKGQQKTA

Supporting Peptides:

Peptide	Uniq	-10lgP	Mass	Length	ppm	m/z	z	RT	Fraction	Scan	Source File	Area F1	Area F2	Area F3	Area F4	Area F5	A
R.AGLQFPVGR.I	Y	47.54	943.5239	9	0.3	472.7694	2	42.70	5	F5:12209	17112021_RID_2349_DP5.raw				4.76E4	5.9883E4	9.1

total 1 peptides

JAG46643.1
back to list

| Protein Coverage | Supporting Peptides |
Protein Coverage:

1 MAGGKAGKDS GKTKTKTVSR SQRAGLQFPV GRIHRHLKSR TTSHGRVGAT AAVYSAAILE YLTAEVLELA GNASKDLKVK

81 RITPRHLQLA IRGDEELDSL IKATIAGGGV IPHIHKSLIG KKGQQKTV

Supporting Peptides:

Peptide	Uniq	-10lgP	Mass	Length	ppm	m/z	z	RT	Fraction	Scan	Source File	Area F1	Area F2	Area F3	Area F4	Area F5	A
R.AGLQFPVGR.I	Y	47.54	943.5239	9	0.3	472.7694	2	42.70	5	F5:12209	17112021_RID_2349_DP5.raw				4.76E4	5.9883E4	9.1

total 1 peptides

AFJ50304.1
back to list

| Protein Coverage | Supporting Peptides |
Protein Coverage:

1 MAGGKAGKDS GKAKAKAVSR SQRAGLQFPV GRIHRHLKTR TTSHGRVGAT AAVYSAAILE YLTAEVLELA GNASKDLKVK

81 RITPRHLQLA IRGDEELDSL IKATIAGGGV IPHIHKSLIG KKGQQKTA

Supporting Peptides:

Peptide	Uniq	-10lgP	Mass	Length	ppm	m/z	z	RT	Fraction	Scan	Source File	Area F1	Area F2	Area F3	Area F4	Area F5	A
R.AGLQFPVGR.I	Y	47.54	943.5239	9	0.3	472.7694	2	42.70	5	F5:12209	17112021_RID_2349_DP5.raw				4.76E4	5.9883E4	9.1

total 1 peptides

JAG67733.1
back to list

| Protein Coverage | Supporting Peptides |
Protein Coverage:

1 MAGGKAGKDS GKAKAKAVSR SQRAGLQFPV GRIHRHLKTR TTSHGRVGAT AAVYSAAILE YLTAEVLELA GNASKDLKVK

81 RITPRHLQLA IRGDEELDSL IKATIAGGGV IPHIHKSLIG KKGQQKTA

Supporting Peptides:

Peptide	Uniq	-10lgP	Mass	Length	ppm	m/z	z	RT	Fraction	Scan	Source File	Area F1	Area F2	Area F3	Area F4	Area F5	A

Peptide	Uniq	-10lgP	Mass	Length	ppm	m/z	z	RT	Fraction	Scan	Source File	Area F1	Area F2	Area F3	Area F4	Area F5	A
R.AGLQFPVGR.I	Y	47.54	943.5239	9	0.3	472.7694	2	42.70	5	F5:12209	17112021_RID_2349_DP5.raw				4.76E4	5.9883E4	9.1

total 1 peptides

JAB54108.1
back to list

| Protein Coverage | Supporting Peptides |
Protein Coverage:

1 MAGGKAGKDS GKAKAKAVSR SQRAGLQFPV GRIHRHLKTR TTSHGRVGAT AAVYSAAILE YLTAEVLELA GNASKDLKVK

81 RITPRHLQLA IRGDEELDSL IKATIAGGGV IPHIHKSLIG KKGQQKTA

Supporting Peptides:

Peptide	Uniq	-10lgP	Mass	Length	ppm	m/z	z	RT	Fraction	Scan	Source File	Area F1	Area F2	Area F3	Area F4	Area F5	A
R.AGLQFPVGR.I	Y	47.54	943.5239	9	0.3	472.7694	2	42.70	5	F5:12209	17112021_RID_2349_DP5.raw				4.76E4	5.9883E4	9.1

total 1 peptides

JAC95409.1
back to list

| Protein Coverage | Supporting Peptides |
Protein Coverage:

1 MAGGKAGKDS GKAKAKAVSR SQRAGLQFPV GRIHRHLKTR TTSHGRVGAT AAVYSAAILE YLTAEVLELA GNASKDLKVK

81 RITPRHLQLA IRGDEELDSL IKATIAGGGV IPHIHKSLIG KKGQQKTA

Supporting Peptides:

Peptide	Uniq	-10lgP	Mass	Length	ppm	m/z	z	RT	Fraction	Scan	Source File	Area F1	Area F2	Area F3	Area F4	Area F5	A
R.AGLQFPVGR.I	Y	47.54	943.5239	9	0.3	472.7694	2	42.70	5	F5:12209	17112021_RID_2349_DP5.raw				4.76E4	5.9883E4	9.1

total 1 peptides

JAI13026.1
back to list

| Protein Coverage | Supporting Peptides |
Protein Coverage:

1 MAGGKAGKDS GKAKAKAVSR SQRAGLQFPV GRIHRHLKTR TTSHGRVGAT AAVYSAAILE YLTAEVLELA GNASKDLKVK

81 RITPRHLQLA IRGDEELDSL IKATIAGGGV IPHIHKSLIG KKGQQKTA

Supporting Peptides:

Peptide	Uniq	-10lgP	Mass	Length	ppm	m/z	z	RT	Fraction	Scan	Source File	Area F1	Area F2	Area F3	Area F4	Area F5	A
R.AGLQFPVGR.I	Y	47.54	943.5239	9	0.3	472.7694	2	42.70	5	F5:12209	17112021_RID_2349_DP5.raw				4.76E4	5.9883E4	9.1

total 1 peptides

JAA96854.1
back to list

| Protein Coverage | Supporting Peptides |
Protein Coverage:

1 MAGGKAGKDS GKTKTKTVSR SQRAGLQFPV GRIHRHLKSR TTSHGRVGAT AAVYSAAILE YLTAEVLELA GNASKDLKVK

81 RITPRHLQLA IRGDEELDSL IKATIAGGGV IPHIHKSLIG KKGQQKTV

Supporting Peptides:

Peptide	Uniq	-10lgP	Mass	Length	ppm	m/z	z	RT	Fraction	Scan	Source File	Area F1	Area F2	Area F3	Area F4	Area F5	A
R.AGLQFPVGR.I	Y	47.54	943.5239	9	0.3	472.7694	2	42.70	5	F5:12209	17112021_RID_2349_DP5.raw				4.76E4	5.9883E4	9.1

total 1 peptides

JAG44452.1
back to list

| Protein Coverage | Supporting Peptides |
Protein Coverage:

1 MAGGKAGKDS GKTKTKTVSR SQRAGLQFPV GRIHRHLKSR TTSHGRVGAT AAVYSAAILE YLTAEVLELA GNASKDLKVK

81 RITPRHLQLA IRGDEELDSL IKATIAGGGV IPHIHKSLIG KKGQQKTV

Supporting Peptides:

Peptide	Uniq	-10lgP	Mass	Length	ppm	m/z	z	RT	Fraction	Scan	Source File	Area F1	Area F2	Area F3	Area F4	Area F5	A
R.AGLQFPVGR.I	Y	47.54	943.5239	9	0.3	472.7694	2	42.70	5	F5:12209	17112021_RID_2349_DP5.raw				4.76E4	5.9883E4	9.1

total 1 peptides

JAA96855.1
back to list

| Protein Coverage | Supporting Peptides |

Protein Coverage:

```
 1  MAGGKAGKDS GKAKAKAVSR SQRAGLQFPV GRIHRHLKTR TTSHGRVGAT AAVYSAAILE YLTAEVLELA GNASKDLKVK

81  RITPRHLQLA IRGDEELDSL IKATIAGGGV IPHIHKSLIG KKGQQKTA
```

Supporting Peptides:

Peptide	Uniq	-10lgP	Mass	Length	ppm	m/z	z	RT	Fraction	Scan	Source File	Area F1	Area F2	Area F3	Area F4	Area F5	A
R.AGLQFPVGR.I	Y	47.54	943.5239	9	0.3	472.7694	2	42.70	5	F5:12209	17112021_RID_2349_DP5.raw				4.76E4	5.9883E4	9.1

total 1 peptides

XP_013930707.1

back to list

| Protein Coverage | Supporting Peptides |

Protein Coverage:

```
 1  MAGGKAGKDS GKTKTKTVSR SQRAGLQFPV GRIHRHLKSR TTSHGRVGAT AAVYSAAILE YLTAEVLELA GNASKDLKVK

81  RITPRHLQLA IRGDEELDSL IKATIAGGGV IPHIHKSLIG KKGQQKTV
```

Supporting Peptides:

Peptide	Uniq	-10lgP	Mass	Length	ppm	m/z	z	RT	Fraction	Scan	Source File	Area F1	Area F2	Area F3	Area F4	Area F5	A
R.AGLQFPVGR.I	Y	47.54	943.5239	9	0.3	472.7694	2	42.70	5	F5:12209	17112021_RID_2349_DP5.raw				4.76E4	5.9883E4	9.1

total 1 peptides

XP_013931116.1

back to list

| Protein Coverage | Supporting Peptides |

Protein Coverage:

```
 1  MSGRGKQGGK ARAKAKSRSS RAGLQFPVGR VHRLLRKGNY AERVGAGAPV YLAAVLEYLT AEILELAGNA ARDNKKTRII

81  PRHLQLAIRN DEELNKLLGK VTIAQGGVLP NIQAVLLPKK TESHKAKGK
```

Supporting Peptides:

Peptide	Uniq	-10lgP	Mass	Length	ppm	m/z	z	RT	Fraction	Scan	Source File	Area F1	Area F2	Area F3	Area F4	Area F5	A
R.AGLQFPVGR.V	Y	47.54	943.5239	9	0.3	472.7694	2	42.70	5	F5:12209	17112021_RID_2349_DP5.raw				4.76E4	5.9883E4	9.

total 1 peptides

XP_013908942.1

back to list

| Protein Coverage | Supporting Peptides |

Protein Coverage:

```
 1  MSGRGKQGGK TRAKAKTRSS RAGLQFPVGR VHRLLRKGNY AERVGAGAPV YLAAVLEYLT AEILELAGNA ARDNKKTRII

81  PRHLQLAIRN DEELNKLLGR VTIAQGGVLP NIQAVLLPKK TESHKAKAK
```

Supporting Peptides:

Peptide	Uniq	-10lgP	Mass	Length	ppm	m/z	z	RT	Fraction	Scan	Source File	Area F1	Area F2	Area F3	Area F4	Area F5	A
R.AGLQFPVGR.V	Y	47.54	943.5239	9	0.3	472.7694	2	42.70	5	F5:12209	17112021_RID_2349_DP5.raw				4.76E4	5.9883E4	9.

total 1 peptides

XP_013911300.1

back to list

| Protein Coverage | Supporting Peptides |

Protein Coverage:

```
 1  MSGRGKVGGG KTRSKAKSRS SRAGLQFPVG RVHRLLRRGR YAERVGAGAP VYLAAVLEYL TAEILELAGN AARDNRKTRI

81  VPRHVQLAVR NDEELSRLLG RVTIAQGGVL PNIPAALLPR KAAAQGGGAA GRKKGALQAS QEY
```

Supporting Peptides:

Peptide	Uniq	-10lgP	Mass	Length	ppm	m/z	z	RT	Fraction	Scan	Source File	Area F1	Area F2	Area F3	Area F4	Area F5	A
R.AGLQFPVGR.V	Y	47.54	943.5239	9	0.3	472.7694	2	42.70	5	F5:12209	17112021_RID_2349_DP5.raw				4.76E4	5.9883E4	9.

total 1 peptides

ETE60792.1

back to list

| Protein Coverage | Supporting Peptides |

Protein Coverage:

```
 1  MSGRGKTGGK ARAKAKSRSS RAGLQFPVGR VHRLLRKGNY AERIGGGAPV YLAAVLEYLS AEILELAGNA ARDNKKSRII

81  PRHLQLAVRN DEELNKLLGG VTIAQGGVLP NIHAVLLPKK TEILHSASSS KPTTSVSKST KVPKVAVKPT TAPVSV
```

Supporting Peptides:

Peptide	Uniq	-10lgP	Mass	Length	ppm	m/z	z	RT	Fraction	Scan	Source File	Area F1	Area F2	Area F3	Area F4	Area F5	A

Peptide	Uniq	-10lgP	Mass	Length	ppm	m/z	z	RT	Fraction	Scan	Source File	Area F1	Area F2	Area F3	Area F4	Area F5	A
R.AGLQFPVGR.V	Y	47.54	943.5239	9	0.3	472.7694	2	42.70	5	F5:12209	17112021_RID_2349_DP5.raw				4.76E4	5.9883E4	9.

total 1 peptides

XP_013924759.1
back to list

| Protein Coverage | Supporting Peptides |
Protein Coverage:

1 MSGRGKTGGK ARAKAKSRSS R**AGLQFPVGR** VHRLLRKGNY AERIGGGAPV YLAAVLEYLS AEILELAGNA ARDNKKSRII

81 PRHLQLAVRN DEELNKLLGG VTIAQGGVLP NIHAVLLPKK TEILHSASSS KPTTSVSKSA KVPKVAVKPT TAPVAVQ

Supporting Peptides:

Peptide	Uniq	-10lgP	Mass	Length	ppm	m/z	z	RT	Fraction	Scan	Source File	Area F1	Area F2	Area F3	Area F4	Area F5	A
R.AGLQFPVGR.V	Y	47.54	943.5239	9	0.3	472.7694	2	42.70	5	F5:12209	17112021_RID_2349_DP5.raw				4.76E4	5.9883E4	9.

total 1 peptides

XP_026542761.1
back to list

| Protein Coverage | Supporting Peptides |
Protein Coverage:

1 MSGRGKTGGK ARAKAKSRSS R**AGLQFPVGR** VHRLLRKGNY AERIGGGAPV YLAAVLEYLS AEILELAGNA ARDNKKSRII

81 PRHLQLAVRN DEELNKLLGG VTIAQGGVLP NIHAVLLPKK TEILHSASSS KPTTSVSKST KVPKVAVKPT TAPVSVQ

Supporting Peptides:

Peptide	Uniq	-10lgP	Mass	Length	ppm	m/z	z	RT	Fraction	Scan	Source File	Area F1	Area F2	Area F3	Area F4	Area F5	A
R.AGLQFPVGR.V	Y	47.54	943.5239	9	0.3	472.7694	2	42.70	5	F5:12209	17112021_RID_2349_DP5.raw				4.76E4	5.9883E4	9.

total 1 peptides

ETE59971.1
back to list

| Protein Coverage | Supporting Peptides |
Protein Coverage:

1 GRGRPGSLLA PRRVIVSRLS QAALARNRDR TSPRSDANMA GGKAGKDSGK AKAKAVSRSQ R**AGLQFPVGR** IHRHLKTRTT

81 SHGRVGATAA VYSAAILEYL TAEVLELAGN ASKDLKVKRI TPRHLQLAIR GDEELDSLIK ATIAGGGVIP HIHKSLIGKK

161 GQQKTA

Supporting Peptides:

Peptide	Uniq	-10lgP	Mass	Length	ppm	m/z	z	RT	Fraction	Scan	Source File	Area F1	Area F2	Area F3	Area F4	Area F5	A
R.AGLQFPVGR.I	Y	47.54	943.5239	9	0.3	472.7694	2	42.70	5	F5:12209	17112021_RID_2349_DP5.raw				4.76E4	5.9883E4	9.1

total 1 peptides

XP_013921022.1
back to list

| Protein Coverage | Supporting Peptides |
Protein Coverage:

1 MLFHLTILFK KFHISPYFFC PNYSTMTGRN RDRTSPRSDA NMAGGKAGKD SGKAKAKAVS RSQR**AGLQFP VGR**IHRHLKT

81 RTTSHGRVGA TAAVYSAAIL EYLTAEVLEL AGNASKDLKV KRITPRHLQL AIRGDEELDS LIKATIAGGG VIPHIHKSLI

161 GKKGQQKTA

Supporting Peptides:

Peptide	Uniq	-10lgP	Mass	Length	ppm	m/z	z	RT	Fraction	Scan	Source File	Area F1	Area F2	Area F3	Area F4	Area F5	A
R.AGLQFPVGR.I	Y	47.54	943.5239	9	0.3	472.7694	2	42.70	5	F5:12209	17112021_RID_2349_DP5.raw				4.76E4	5.9883E4	9.1

total 1 peptides

LAB40587.1
back to list

| Protein Coverage | Supporting Peptides |
Protein Coverage:

1 MPDVSLEERL PSILSTHSGA GGKYKSRSSW QHGLQFQVVS RWLRIRMSGR GKQDSKARAK AKTRSSR**AGL QFPVGR**VHRL

81 LRRGHYAERV GAGAPVYLAA VLEYLTAEIL ELAGNAARDN RKTRIVPRHV QLAVRNDEEL SRLLGRVTIA QGGVLPNIPA

161 ALLPRKAAAA GGGTAGRQKG THQASQEN

Supporting Peptides:

Peptide	Uniq	-10lgP	Mass	Length	ppm	m/z	z	RT	Fraction	Scan	Source File	Area F1	Area F2	Area F3	Area F4	Area F5	A

total 1 peptides

Peptide	Uniq	-10lgP	Mass	Length	ppm	m/z	z	RT	Fraction	Scan	Source File	Area F1	Area F2	Area F3	Area F4	Area F5	A
R.AGLQFPVGR.V	Y	47.54	943.5239	9	0.3	472.7694	2	42.70	5	F5:12209	17112021_RID_2349_DP5.raw				4.76E4	5.9883E4	9.

total 1 peptides

TRINITY_DN1911
back to list

| Protein Coverage | Supporting Peptides |
Protein Coverage:

1 **VAQLASGFIQ K**PGIYVWIGL RDRRKEQVVL NFMTTTESRI SVAK

Supporting Peptides:

Peptide	Uniq	-10lgP	Mass	Length	ppm	m/z	z	RT	Fraction	Scan	Source File	Area F1	Area F2	Area F3	Area F4	Area F5	Ar F
VAQLASGFIQK.P	Y	46.25	1160.6553	11	0.8	581.3354	2	40.26	5	F5:11370	17112021_RID_2349_DP5.raw					2.6109E5	

total 1 peptides

TRINITY_DN1911
back to list

| Protein Coverage | Supporting Peptides |
Protein Coverage:

1 **VAQLASGFIQ K**PGIYVWIGL RDRRKEQVVL NFMTTTESRI SVAK

Supporting Peptides:

Peptide	Uniq	-10lgP	Mass	Length	ppm	m/z	z	RT	Fraction	Scan	Source File	Area F1	Area F2	Area F3	Area F4	Area F5	Ar F
VAQLASGFIQK.P	Y	46.25	1160.6553	11	0.8	581.3354	2	40.26	5	F5:11370	17112021_RID_2349_DP5.raw					2.6109E5	

total 1 peptides

XP_026548138.1
back to list

| Protein Coverage | Supporting Peptides |
Protein Coverage:

1 MAQLDQLSPY VKKVDRSAQA DPPQQASSTP VEKWPDPIPI ERLQRSLSLN LGEGTSSATE PVAARTRAKT EQASLVAPLR
81 QVDQLAINPK KPSESVTLSL FQHVPFTSSD LLNWKLHYGP FSEKPMEVAD LVKTIMDAHN PNWLDVQQLM GTLFSPEERE
161 KIKNAISEIL KPDVTLYHSL TDLMDEKYPS SNPDWDLYTD EGKTRLRDYQ SLVVRAIRLA GKPVINMSKP ALVLQEPTES
241 PEAFYTRLVE SYRMYTPIDP SAPENARMMT MAFISQSALD IRRKLQRLEG ALGKPMSELM EVARKVFANR DKEEDKKKDH
321 KMQRKAELLA AAIAWPSRGR VNPLRGMGPG PQRGRGNSLQ RGVSWDLGPN QCAFCKQQGH WKGECPERND RGRSGDRGDS
401 PRNLTFAVEE EWDXGRPGSE SLPEPMVSLK VGGKPVTFLV DTGATRSVLT QPMAQASRQT INIQGATGNT LQRPFLEPLE
481 CTVKGQAITH QFVYMPDCPV PLLGRDLLCK LRAQISFAED GSMHLGYGKL GFSIPIEESW RLMVARTTGQ ETEETWRQFE
561 VPYLWAEDNP PGFAAHHPPI LVEEVPTKPP VRIRQRAYPR HVMQAIQDII DIYLQHHILV PTESPWNTQI LPIPKGDGRF
641 RPVQDLRPVN LATVTIHPVV PNPYVILGLI PQAAQWFSVI DLKDAFFTIA IHELSQHLFA FEWENPATGR KQQYTWTRLP
721 QGFKNSPTLF GTALAKDLEN LHIPAPDLVL QYVDDLLVTG LTEDRCWENT KDLLSLLQTL GYKASRNKAQ LVLQKVRYLG
801 YDIERGKRTL GHERKEAICQ LPRPINKKDL RGFLGAAGFC RIWIPKFSLI AKPLYEATKG ANRDPLHWGP EEEASFCALK
881 QALLQAPSLG LPDLDKPFQL FVDTKKEVAV GVLTQTIGSW HRPVAYLSKQ LDNVAKGWPA CLKAVAGTAL LTQEANKLTF
961 GQQLDIFTPH ALKSVLDMKG HLWLTNPRML KYQGLLTHNP MITLTQSTLL NPATLLPEPD MGVHHDCIHT IEETYASRPD
1041 MRDTPLPAPD YSLFTDGTSF LQEGMRKAGY AVVTLDEVWE AKPLPPGTSA QLAELHALTR ALELAKGLAV NIYTDSKYAF
1121 LTVQVHGALY KERGLITAGG KDIK**YGPHIL R**LLDSVWSPS KVAVMHCRGH QKVVTDIHRG NERADKFARK AAMLEFIPEE
1201 RNLAVWLPDD NPKPHYSSED RDRLDALEAH PDGHWWVLPD NRVAEIKSWI HWSRVKLAAP TSWKVETCPD SLLKVILKRA
1281 KKDISPGGDT PEAVPLPTGD EEDLPSPSGD TPEAVPQPTG GVERQTPAGV TPDADAHRTG LPWRLRSSAK RQ

Supporting Peptides:

Peptide	Uniq	-10lgP	Mass	Length	ppm	m/z	z	RT	Fraction	Scan	Source File	Area F1	Area F2	Area F3	Area F4	Area F5	Area F6	Area F
K.YGPHILR.L	Y	45.78	854.4763	7	1.0	428.2458	2	29.13	7	F7:7565	17112021_RID_2349_DP7.raw							1.3984E

total 1 peptides

XP_032092456.1
back to list

| Protein Coverage | Supporting Peptides |
Protein Coverage:

1 MILWLNLVSL LVSLGGVRSD VQLVESGGDV RRPGESLRLS CQASGFTFSS YYMNWVRQAP GKGLEWVASI SESSGTYTYY
81 SDKVKGRFTI SRDNAKSQLY LQMNNLKEED TAVYYCARGT VRGSLSEEFS LKLLFLGIQS **EVQLVESGGG VR**RPGESLRL
161 SCQGSGFTFS CYYMNWVRQA PGKRPEWVVY ISISSSNIYY SDKVKGRFTI SRDDSNNLLY LQMKNLKPED TAVYYCARDA
241 Q

Supporting Peptides:

Peptide	Uniq	-10lgP	Mass	Length	ppm	m/z	z	RT	Fraction	Scan	Source File	Area F1	Area F2	Area F3	Area F4	Area F5	Are F(

Peptide	Uniq	-10lgP	Mass	Length	ppm	m/z	z	RT	Fraction	Scan	Source File	Area F1	Area F2	Area F3	Area F4	Area F5	Ar[illegible] F[illegible]
S.EVQLVESGGGVR.R	Y	42.06	1228.6411	12	-0.8	615.3273	2	33.46	7	F7:9095	17112021_RID_2349_DP7.raw						

total 1 peptides

XP_007437884.2
back to list

| Protein Coverage | Supporting Peptides |
Protein Coverage:

1 MFCGKLNMHV NIQTGKWETD PSGTKSCFGR KEEILEYCQE IYPDLRITNV VELNEPVCIK NWCKKGKKQC KGHTHIVVPF
81 KCLVGEFVSD VLLVPEKCRF FHRERMVVCK SHQYWHIVAK EACLTEGMIL HSYGMLLPCG VDKFHGTEYV CCPQTKIVDE
161 ALSKEKEDED EEEDEDEDDD GIKSELPTEA DTDDFTVGVM EMNEEDEEEQ EENEEEVVVD QDYYYDSYKD YNEELPSEPS
241 NGRSFSQKEI ISDVKAVCSQ EAMTGPCRAM MPRWYFDMYK KKCIRFIYGG CGGNRNNFES EEYCMAVCKK MIPPTPLPTD
321 DVDVYFETPA DDNEHARFQK AKEQLEIRHR NRMDRVK**KEW EEAEK**QAKNL PKTERQALMQ RFQTMAKSLE KEAASEKQQL
401 VETHLARVEA MLDDRHRIAL ENYLVALQTD PPRPHRILQA LKRYVRAENK DRLHTIHHYQ HVLAVDPEKA AQMKSQVMTH
481 LHVIEERMNQ SLSLLYKVPY VAEEIQNEID ELLQEQRTDM DQFTSSISES QVDVHVSSEE SEESPLFDGK PFRPFQVKTF
561 PASSKSEGSG LADLDGLIGA EEKVINSKNK LDENVKYPVP TMPR

Supporting Peptides:

Peptide	Uniq	-10lgP	Mass	Length	ppm	m/z	z	RT	Fraction	Scan	Source File	Area F1	Area F2	Area F3	Area F4	Area F5	Area F6
K.KEWEEAEK.Q	Y	40.82	1047.4873	8	0.1	524.7510	2	26.98	6	F6:6676	17112021_RID_2349_DP6.raw						2.3088E[illegible]

total 1 peptides

XP_007437883.2
back to list

| Protein Coverage | Supporting Peptides |
Protein Coverage:

1 MFCGKLNMHV NIQTGKWETD PSGTKSCFGR KEEILEYCQE IYPDLRITNV VELNEPVCIK NWCKKGKKQC KGHTHIVVPF
81 KCLVGEFVSD VLLVPEKCRF FHRERMVVCK SHQYWHIVAK EACLTEGMIL HSYGMLLPCG VDKFHGTEYV CCPQTKIVDE
161 ALSKEKEDED EEEDEDEDDD GIKSELPTEA DTDDFTVGVM EMNEEDEEEQ EENEEEVVVD QDYYYDSYKD YNEELPSEPS
241 NGRSFSQKEI ISDVKAVCSQ EAMTGPCRAM MPRWYFDMYK KKCIRFIYGG CGGNRNNFES EEYCMAVCKK MIPPTPLPTD
321 DVDVYFETPA DDNEHARFQK AKEQLEIRHR NRMDRVK**KEW EEAEK**QAKNL PKTERQALMQ RFQTMAKSLE KEAASEKQQL
401 VETHLARVEA MLDDRHRIAL ENYLVALQTD PPRPHRILQA LKRYVRAENK DRLHTIHHYQ HVLAVDPEKA AQMKSQVMTH
481 LHVIEERMNQ SLSLLYKVPY VAEEIQNEID ELLQEQRTDM DQFTSSISES QVDVHVSSEE SEESPLFDGK PFRPFQVKTF
561 PASSKSEDSQ PDLYHPEKKG SGLADLDGLI GAEEKVINSK NKLDENVKYP VPTMPR

Supporting Peptides:

Peptide	Uniq	-10lgP	Mass	Length	ppm	m/z	z	RT	Fraction	Scan	Source File	Area F1	Area F2	Area F3	Area F4	Area F5	Area F6
K.KEWEEAEK.Q	Y	40.82	1047.4873	8	0.1	524.7510	2	26.98	6	F6:6676	17112021_RID_2349_DP6.raw						2.3088E[illegible]

total 1 peptides

XP_007437886.2
back to list

| Protein Coverage | Supporting Peptides |
Protein Coverage:

1 MFCGKLNMHV NIQTGKWETD PSGTKSCFGR KEEILEYCQE IYPDLRITNV VELNEPVCIK NWCKKGKKQC KGHTHIVVPF
81 KCLVGEFVSD VLLVPEKCRF FHRERMVVCK SHQYWHIVAK EACLTEGMIL HSYGMLLPCG VDKFHGTEYV CCPQTKIVDE
161 ALSKEKEDED EEEDEDEDDD GIKSELPTEA DTDDFTVGVM EMNEEDEEEQ EENEEEVVVD QDYYYDSYKD YNEELPSEPS
241 NGRSFSQKEI ISDVKVPPTP LPTDDVDVYF ETPADDNEHA RFQKAKEQLE IRHRNRMDRV K**KEWEEAEK**Q AKNLPKTERQ
321 ALMQRFQTMA KSLEKEAASE KQQLVETHLA RVEAMLDDRH RIALENYLVA LQTDPPRPHR ILQALKRYVR AENKDRLHTI
401 HHYQHVLAVD PEKAAQMKSQ VMTHLHVIEE RMNQSLSLLY KVPYVAEEIQ NEIDELLQEQ RTDMDQFTSS ISESQVDVHV
481 SSEESEESPL FDGKPFRPFQ VKTFPASSKS EGSGLADLDG LIGAEEKVIN SKNKLDENVK YPVPTMPR

Supporting Peptides:

Peptide	Uniq	-10lgP	Mass	Length	ppm	m/z	z	RT	Fraction	Scan	Source File	Area F1	Area F2	Area F3	Area F4	Area F5	Area F6
K.KEWEEAEK.Q	Y	40.82	1047.4873	8	0.1	524.7510	2	26.98	6	F6:6676	17112021_RID_2349_DP6.raw						2.3088E[illegible]

total 1 peptides

XP_007437885.2
back to list

| Protein Coverage | Supporting Peptides |
Protein Coverage:

1 MFCGKLNMHV NIQTGKWETD PSGTKSCFGR KEEILEYCQE IYPDLRITNV VELNEPVCIK NWCKKGKKQC KGHTHIVVPF
81 KCLVGEFVSD VLLVPEKCRF FHRERMVVCK SHQYWHIVAK EACLTEGMIL HSYGMLLPCG VDKFHGTEYV CCPQTKIVDE
161 ALSKEKEDED EEEDEDEDDD GIKSELPTEA DTDDFTVGVM EMNEEDEEEQ EENEEEVVVD QDYYYDSYKD YNEELPSEPS
241 NGRSFSQKEI ISDVKVPPTP LPTDDVDVYF ETPADDNEHA RFQKAKEQLE IRHRNRMDRV K**KEWEEAEK**Q AKNLPKTERQ
321 ALMQRFQTMA KSLEKEAASE KQQLVETHLA RVEAMLDDRH RIALENYLVA LQTDPPRPHR ILQALKRYVR AENKDRLHTI
401 HHYQHVLAVD PEKAAQMKSQ VMTHLHVIEE RMNQSLSLLY KVPYVAEEIQ NEIDELLQEQ RTDMDQFTSS ISESQVDVHV
481 SSEESEESPL FDGKPFRPFQ VKTFPASSKS EDSQPDLYHP EKKGSGLADL DGLIGAEEKV INSKNKLDEN VKYPVPTMPR

Supporting Peptides:

Peptide	Uniq	-10lgP	Mass	Length	ppm	m/z	z	RT	Fraction	Scan	Source File	Area F1	Area F2	Area F3	Area F4	Area F5	Area F6
K.KEWEEAEK.Q	Y	40.82	1047.4873	8	0.1	524.7510	2	26.98	6	F6:6676	17112021_RID_2349_DP6.raw						2.3088E[illegible]

total 1 peptides

XP_007421854.1
back to list

| Protein Coverage | Supporting Peptides |

Protein Coverage:

1 MQRLRDADSS MFKPECIYTS GGGWTEGGGS ADSQSMPYFA KLMLLIFCGV LLGESRKHKR GRWTEEADVQ KTSHTYKKNY
81 DMTKAWSGNN KQLLRVEDHD FTMRPAFGGP AIPVGVDVQV ESLDSISEVN MDFTMTLYLR HYWKDERLSF PSHANKSMTF
161 DGRLVKKIWV PDIFFVHSKR SFIHDTTTDN IMLRVFPDGH VLYSMRITVT AMCNMDFSRF PLDSQTCSLE LESYAYTDED
241 LMLYWKNGSE SLKTDEKISL SQFLIQKFHT TSRLAFYSST GRYNRLYINF ILRRHIFFFL LQTYFPATLM VMLSWVSFWI
321 DRRAVPARVS LGITTVLTMS TIITGVNASM PGVSYIKAVD IYLWVSFVFV FLSVIEYAAV NYLTTVQERK QRKQQEK**FPC**
401 **VSGISSTK**RM TLDGIYNELD ASSLEGDPRS QMVAEEDKQE KMVIHLTMSN ESNSSKKKGL KEHVSLRIIQ NTHAIDKYSR
481 LIFPGTYIFF NIVYWSVFC

Supporting Peptides:

Peptide	Uniq	-10lgP	Mass	Length	ppm	m/z	z	RT	Fraction	Scan	Source File	Area F1	Area F2	Area F3	Area F4
K.FPC(+57.02)VSGISSTK.R	Y	39.14	1181.5751	11	-0.4	591.7946	2	42.28	5	F5:12814	17112021_RID_2349_DP5.raw				4.3217E[illegible]

Peptide List

Prepared with PEAKS ™ (bioinfor.com)
