# Supplementary material for: The Middle Eastern Cousin: Comparative Venomics of Daboia palaestinae and Daboia russelii
Source: Toxins (Basel). 2022 Oct 23;14(11):725. doi: 10.3390/toxins14110725 (PMC9696752; doi:10.3390/toxins14110725)
Supplement: Supplementary file 1 [file toxins-14-00725-s001.zip › Supplementary Tables.pdf]

Supplementary Table

Raw MS/MS spectra of each gel excised bands were searched against the National Center for Biotechnology Information's (NCBI) non-redundant (nr) database (Serpentes: 8570) using Peaks Studio X+ for the identification of toxin classes present in the venom. The key results of these searches, including the accession numbers, species, -10lgP values, coverage, number of high confidence peptides, unique peptides, percent abundance of each toxin hit, average molecular mass (kDa) and the toxin family of the matching NCBI entry was identified are listed here. The percentage indicated adjacent to the average mass column corresponds to its relative proportion in the venom of *D. palaestinae* **(Table S1)**.

Table S1. The proteomic composition of *D. palaestinae* venom.

Sr. No.	Accession	Species	-10lgP	Coverage (%)	#Peptides	#Unique	Relative abandance of toxin hit (%)	Avg. Mass (kda)	Toxin Type
**Snake venom serine protease (SVSP): 41.382%**									
1	TRINITY_DN441	*D. palaestinae* transcriptome	270.06	71	24	15	10.1632	13.35	SVSP
2	TRINITY_DN240	*D. palaestinae* transcriptome	245.05	44	15	5	11.9091	15.30	SVSP
3	Q9PT40.1	*Macrovipera lebetina*	215.17	26	11	5	1.5929	28.89	SVSP
4	ADN04916.1	*Macrovipera lebetina*	186.74	21	10	6	0.9888	28.70	SVSP
5	P18964.1	*Daboia siamensis*	181.11	18	8	7	0.0993	26.18	SVSP
6	TRINITY_DN441	*D. palaestinae* transcriptome	172.25	35	9	5	9.4037	16.51	SVSP
7	TRINITY_DN240	*D. palaestinae* transcriptome	153.29	22	4	2	0.1057	13.90	SVSP
8	TRINITY_DN0	*D. palaestinae* transcriptome	148.09	19	5	2	3.7123	19.01	SVSP
9	E5AJX2.1	*Vipera berus nikolskii*	137.19	13	5	1	0.0070	28.21	SVSP
10	AUS82489.1	*Crotalus atrox*	125.09	11	4	1	0.0001	28.08	SVSP
11	ADN04917.1	*Macrovipera lebetina*	122.9	8	2	1	0.6202	28.29	SVSP
12	ADP88559.1	*Daboia siamensis*	117.89	11	3	1	0.0005	28.49	SVSP
13	XP_015666085.1	*Protobothrops mucrosquamatus*	114.12	8	3	3	0.0038	54.74	SVSP
14	P86497.1	*Bitis rhinoceros*	114.04	15	4	1	0.1342	9.97	SVSP
15	ADP88560.1	*Daboia siamensis*	105.88	10	2	1	0.0060	28.03	SVSP
16	E0Y421.1	*Macrovipera lebetina*	103.68	8	3	1	0.1653	28.27	SVSP

17	TRINITY_DN13494	*D. palaestinae* transcriptome	92.73	7	2	2	0.0036	38.00	SVSP
18	ART88739.1	*Azemiops feae*	79.86	7	2	1	0.0006	27.74	SVSP
19	ABB76279.1	*Lachesis stenophrys*	73.14	9	1	1	0.0004	27.81	SVSP
20	TRINITY_DN233	*D. palaestinae* transcriptome	359.11	51	37	24	1.6643	76.82	SVSP
21	TRINITY_DN132	*D. palaestinae* transcriptome	293.5	30	29	29	0.7847	110.24	SVSP
22	TRINITY_DN91	*D. palaestinae* transcriptome	192.1	8	7	4	0.0046	110.85	SVSP
23	TRINITY_DN283	*D. palaestinae* transcriptome	165.03	10	8	8	0.0114	106.18	SVSP
24	XP_034291429.1	*Pantherophis guttatus*	148.76	5	4	1	0.0005	113.38	SVSP
**Lectins: 28.923%**									
25	TRINITY_DN59	*D. palaestinae* transcriptome	243.26	37	21	18	19.4976	17.72	Lectin
26	TRINITY_DN554	*D. palaestinae* transcriptome	226.4	16	8	8	3.6991	18.18	Lectin
27	ADK22823.1	*Daboia siamensis*	197.97	31	12	4	0.0244	17.50	Lectin
28	TRINITY_DN64	*D. palaestinae* transcriptome	193.05	37	8	1	0.0287	14.8	Lectin
29	ADK22827.1	*Daboia russelii*	188.3	28	8	1	0.0309	18.10	Lectin
30	ADK22824.1	*Daboia siamensis*	186.62	35	10	6	3.3302	18.06	Lectin
31	TRINITY_DN59	*D. palaestinae* transcriptome	169.25	23	5	1	0.0204	17.59	Lectin
32	TRINITY_DN278	*D. palaestinae* transcriptome	169.22	33	6	1	0.0095	14.04	Lectin
33	TRINITY_DN59	*D. palaestinae* transcriptome	166.98	37	9	1	0.0098	15.35	Lectin
34	APB93444.1	*Vipera ammodytes ammodytes*	156.68	34	8	1	0.0495	17.28	Lectin
35	TRINITY_DN743	*D. palaestinae* transcriptome	148.55	15	3	3	0.0390	21.19	Lectin
36	TRINITY_DN4675	*D. palaestinae* transcriptome	137.11	13	3	2	0.0957	21.08	Lectin
37	TRINITY_DN59	*D. palaestinae* transcriptome	137.06	22	3	1	0.3197	15.02	Lectin
38	TRINITY_DN12452	*D. palaestinae* transcriptome	126.04	19	2	2	1.5737	13.39	Lectin
39	P0DJL5.1	*Daboia palaestinae*	116.25	22	3	3	0.0094	15.15	Lectin
40	TRINITY_DN554	*D. palaestinae* transcriptome	114.68	20	3	1	0.1770	15.33	Lectin
41	AAB36402.1	*Echis carinatus*	85.29	13	3	1	0.0050	14.37	Lectin
42	TRINITY_DN1911	*D. palaestinae* transcriptome	46.25	25	1	1	0.0030	5.00	Lectin
**Phospholipase A_2 (PLA_2): 12.209%**									
43	AAZ53176.1	*Daboia siamensis*	195.25	40	13	9	10.9150	15.58	PLA2

44	CAR40186.1	*Macrovipera lebetina*	169.6	28	6	2	1.2759	15.39	PLA2
45	1OQS	*Daboia siamensis*	89.8	14	2	2	0.0136	13.81	PLA2
46	CAE47141.1	*Vipera aspis aspis*	75.31	12	2	1	0.0041	15.46	PLA2
**L-amino-acid oxidase (LAAO): 6.564%**									
47	TRINITY_DN9	*D. palaestinae* transcriptome	397.42	65	68	13	6.5640	57.04	LAAO
**Vascular endothelial growth factor (VEGF): 5.596%**									
48	P0DL42.1	*Daboia siamensis*	206.88	39	13	11	5.2859	12.55	VEGF
49	TRINITY_DN588	*D. palaestinae* transcriptome	135.41	20	5	1	0.0010	17.10	VEGF
50	P82475.2	*Macrovipera lebetina*	130.5	26	5	3	0.3094	12.57	VEGF
**Snake venom metalloproteinases (SVMP): 3.384%**									
51	TRINITY_DN30	*D. palaestinae* transcriptome	254.87	13	16	2	0.0211	69.49	SVMP
52	TRINITY_DN1385	*D. palaestinae* transcriptome	231.26	40	11	3	0.6196	15.27	SVMP
53	Q4VM07.1	*Macrovipera lebetina*	205.78	12	12	3	0.0351	68.84	SVMP
54	TRINITY_DN459	*D. palaestinae* transcriptome	199.41	11	7	2	2.3161	15.03	SVMP
55	TRINITY_DN459	*D. palaestinae* transcriptome	183.64	13	6	1	0.0182	14.96	SVMP
56	B8K1W0.1	*Daboia russelii*	178.02	10	10	7	0.1134	69.55	SVMP
57	AUF41660.1	*Daboia siamensis*	170.03	10	7	1	0.0005	69.15	SVMP
58	QBF53416.1	*Vipera ammodytes ammodytes*	167.52	9	4	1	0.0002	36.83	SVMP
59	TRINITY_DN714	*D. palaestinae* transcriptome	161.86	15	5	1	0.0044	38.79	SVMP
60	TRINITY_DN2452	*D. palaestinae* transcriptome	130.54	24	5	2	0.0236	24.82	SVMP
61	ADJ67475.1	*Daboia russelii*	127.31	5	3	1	0.0001	69.52	SVMP
62	TRINITY_DN471	*D. palaestinae* transcriptome	123.14	19	2	1	0.0631	12.05	SVMP
63	ADI47590.1	*Echis carinatus sochureki*	104.6	5	2	1	0.0173	69.52	SVMP
64	TRINITY_DN2736	*D. palaestinae* transcriptome	103.13	14	2	2	0.0105	10.84	SVMP
65	TRINITY_DN471	*D. palaestinae* transcriptome	102.97	11	1	1	0.0906	12.38	SVMP
66	TRINITY_DN2452	*D. palaestinae* transcriptome	77.88	9	2	2	0.0103	12.33	SVMP
67	ADI47595.1	*Echis carinatus sochureki*	73.04	6	1	1	0.0381	29.96	SVMP
68	JAS05406.1	*Sistrurus miliarius barbouri*	60.18	2	1	1	0.0018	67.26	SVMP
69	CAJ01683.1	*Echis ocellatus*	48.76	3	1	1	0.0003	69.59	SVMP

**5'-nucleotidase (5'-NT): 0.749%**									
70	AHJ80886.1	*Macrovipera lebetina*	337.24	49	30	12	0.6746	45.03	5'-NT
71	TRINITY_DN266	*D. palaestinae* transcriptome	248.99	36	9	1	0.0657	31.95	5'-NT
72	QHR82712.1	*Vipera anatolica senliki*	229.92	32	9	1	0.0089	32.55	5'-NT
**Phospholipase B (PLB): 0.56%**									
73	TRINITY_DN0	*D. palaestinae* transcriptome	307.8	38	25	12	0.5602	64.29	PLB
74	BAN82155.1	*Ovophis okinavensis*	251.91	22	14	1	0.0002	64.13	PLB
**Phosphodiesterase (PDE): 0.252%**									
75	TRINITY_DN1507	*D. palaestinae* transcriptome	257.03	24	16	9	0.2478	86.50	PDE
76	XP_034278116.1	*Pantherophis guttatus*	126.21	5	5	5	0.0038	100.97	PDE
**Kunitz-type serine protease (Kunitz): 0.218%**									
77	A8Y7P1.1	*Daboia siamensis*	92.2	12	2	2	0.0102	9.31	Kunitz
78	AFD04723.1	*Daboia russelii*	87.78	11	2	2	0.0671	10.01	Kunitz
79	ABD24041.1	*Daboia russelii*	86.71	26	3	3	0.0050	9.68	Kunitz
80	P00990.1	*Daboia russelii*	76.97	17	2	2	0.0602	6.85	Kunitz
81	CCH15044.1	*Macrovipera lebetina transmediterranea*	63.13	9	1	1	0.0756	10.56	Kunitz
**Disintegrin: 0.052%**									
82	TRINITY_DN459	*D. palaestinae* transcriptome	94.14	11	1	1	0.0415	14.40	Disintegrin
83	CAJ34939.1	*Macrovipera lebetina*	61.72	19	1	1	0.0103	11.49	Disintegrin
**Cysteine-rich secretory proteins (CRISP): 0.043%**									
84	ACE73567.1	*Daboia russelii*	175.01	15	4	2	0.0379	26.68	CRISP
85	TRINITY_DN1793	*D. palaestinae* transcriptome	155.08	21	4	1	0.0008	26.75	CRISP
86	CAP74088.1	*Vipera berus nikolskii*	127.36	14	3	1	0.0041	24.61	CRISP
**Nerve growth factor (NGF): 0.039%**									
87	JAS04521.1	*Agkistrodon conanti*	128.01	12	3	1	0.0375	27.10	NGF
88	AEH59582.1	*Vipera ursinii*	120.92	12	3	1	0.0014	27.28	NGF
**Hyaluronidases: 0.024%**									
88	TRINITY_DN3925	*D. palaestinae* transcriptome	157.17	14	6	6	0.0238	52.50	Hyaluronidase

**Cystatin: 0.005%**									
90	TRINITY_DN195	*D. palaestinae* transcriptome	156.4	23	5	5	0.0046	22.37	Cystatin
**Serpin: 0.001%**									
91	JAG46182.1	*Crotalus horridus*	109.91	6	2	2	0.0006	44.17	Serpin

Supplementary Table S2: Venom gland transcriptome assembly statistics

Reads				Assembly		
**Tissue**	**Raw**	**Filtered**	**Expressed**	**Annotated**	**Transcripts**	**N50**
Venom gland 1 (left)	41,875,666	38,323,871	32,975,671	67,980	1,91,425	1812
Venom gland 2 (right)	43,278,432	39,637,721	34,484,363			

Supplementary Table S3: Comparative transcriptomics of the left and right venom glands of *D. palaestinae*

Sr. no	Isoform	VG1 (left)	Abundance VG1	VG2 (right)	Abundance VG2	Family
1	TRINITY_DN266_c0_g1_i3	75918	0.5688%	56126	0.5114%	5'-nucleotidase
2	TRINITY_DN266_c0_g1_i8	75918		56126		5'-nucleotidase
3	TRINITY_DN266_c0_g1_i12	75918		56126		5'-nucleotidase
4	TRINITY_DN266_c0_g1_i11	75918		56126		5'-nucleotidase
5	TRINITY_DN1277_c0_g1_i6	1129		1172		5'-nucleotidase
6	TRINITY_DN1430_c0_g2_i3	253		205		5'-nucleotidase
7	TRINITY_DN1430_c0_g2_i2	253		205		5'-nucleotidase
8	TRINITY_DN3725_c0_g2_i4	163		157		5'-nucleotidase
9	TRINITY_DN3725_c0_g2_i11	163		157		5'-nucleotidase
10	TRINITY_DN1046_c0_g1_i1	127		144		5'-nucleotidase
11	TRINITY_DN1793_c0_g1_i7	445	0.0020%	501	0.0027%	CRISP
12	TRINITY_DN1793_c0_g1_i9	445		501		CRISP
13	TRINITY_DN11831_c0_g1_i1	186		190		CRISP
14	TRINITY_DN160_c1_g1_i8	785308	1.4632%	667403	1.5094%	Disintegrin
15	TRINITY_DN39_c1_g1_i1	800		723		Disintegrin
16	TRINITY_DN1910_c1_g1_i2	201		185		Disintegrin
17	TRINITY_DN10769_c0_g1_i2	127		144		Disintegrin
18	TRINITY_DN10769_c0_g1_i5	127		144		Disintegrin
19	TRINITY_DN3925_c0_g1_i6	9936	0.1109%	8753	0.1186%	Hyaluronidase
20	TRINITY_DN3925_c0_g1_i3	9936		8753		Hyaluronidase
21	TRINITY_DN3925_c0_g1_i1	9936		8753		Hyaluronidase
22	TRINITY_DN3925_c0_g1_i5	9936		8753		Hyaluronidase
23	TRINITY_DN3925_c0_g1_i2	9936		8753		Hyaluronidase
24	TRINITY_DN3925_c0_g1_i4	9936		8753		Hyaluronidase

25	TRINITY_DN2597_c0_g1_i2	1645	0.0122%	1595	0.0144%	Kazal
26	TRINITY_DN2597_c0_g1_i1	1645		1595		Kazal
27	TRINITY_DN2597_c0_g1_i4	1645		1595		Kazal
28	TRINITY_DN2597_c0_g1_i3	1645		1595		Kazal
29	TRINITY_DN151_c0_g1_i2	451774	6.7415%	347728	6.3030%	Kunitz
30	TRINITY_DN151_c0_g1_i6	451774		347728		Kunitz
31	TRINITY_DN151_c0_g1_i7	451774		347728		Kunitz
32	TRINITY_DN151_c0_g1_i9	451774		347728		Kunitz
33	TRINITY_DN151_c0_g1_i8	451774		347728		Kunitz
34	TRINITY_DN151_c0_g1_i10	451774		347728		Kunitz
35	TRINITY_DN151_c0_g1_i17	451774		347728		Kunitz
36	TRINITY_DN151_c0_g1_i3	451774		347728		Kunitz
37	TRINITY_DN395_c0_g1_i1	2640		2760		Kunitz
38	TRINITY_DN395_c0_g1_i3	2640		2760		Kunitz
39	TRINITY_DN395_c0_g1_i2	2640		2760		Kunitz
40	TRINITY_DN503_c1_g5_i1	1965		1829		Kunitz
41	TRINITY_DN9_c0_g2_i12	718296	5.3457%	577361	5.2150%	LAAO
42	TRINITY_DN9_c0_g2_i14	718296		577361		LAAO
43	TRINITY_DN9_c0_g2_i5	718296		577361		LAAO
44	TRINITY_DN9_c0_g2_i10	718296		577361		LAAO
45	TRINITY_DN9_c0_g1_i1	373		348		LAAO
46	TRINITY_DN9257_c0_g1_i1	157		223		LAAO
47	TRINITY_DN59_c0_g1_i8	962329	26.2005%	736863	24.4659%	Lectin
48	TRINITY_DN59_c0_g1_i18	962329		736863		Lectin
49	TRINITY_DN59_c0_g1_i6	962329		736863		Lectin
50	TRINITY_DN59_c0_g1_i14	962329		736863		Lectin
51	TRINITY_DN59_c0_g1_i7	962329		736863		Lectin

52	TRINITY_DN59_c0_g1_i2	962329		736863		Lectin
53	TRINITY_DN59_c0_g1_i5	962329		736863		Lectin
54	TRINITY_DN59_c0_g1_i12	962329		736863		Lectin
55	TRINITY_DN59_c0_g1_i4	962329		736863		Lectin
56	TRINITY_DN59_c0_g1_i10	962329		736863		Lectin
57	TRINITY_DN59_c0_g1_i9	962329		736863		Lectin
58	TRINITY_DN59_c0_g1_i3	962329		736863		Lectin
59	TRINITY_DN64_c2_g2_i1	153849		129673		Lectin
60	TRINITY_DN64_c2_g1_i12	155868		124797		Lectin
61	TRINITY_DN64_c2_g1_i2	155868		124797		Lectin
62	TRINITY_DN278_c0_g1_i2	132315		111782		Lectin
63	TRINITY_DN278_c0_g1_i1	132315		111782		Lectin
64	TRINITY_DN278_c0_g1_i13	132315		111782		Lectin
65	TRINITY_DN14197_c0_g1_i2	114301		93202		Lectin
66	TRINITY_DN14197_c0_g1_i6	114301		93202		Lectin
67	TRINITY_DN554_c0_g1_i9	108778		89229		Lectin
68	TRINITY_DN554_c0_g1_i8	108778		89229		Lectin
69	TRINITY_DN406_c1_g1_i9	55746		43058		Lectin
70	TRINITY_DN406_c1_g1_i6	55746		43058		Lectin
71	TRINITY_DN406_c1_g1_i4	55746		43058		Lectin
72	TRINITY_DN406_c1_g1_i13	55746		43058		Lectin
73	TRINITY_DN406_c1_g1_i12	55746		43058		Lectin
74	TRINITY_DN406_c1_g1_i7	55746		43058		Lectin
75	TRINITY_DN406_c1_g1_i11	55746		43058		Lectin
76	TRINITY_DN406_c1_g1_i1	55746		43058		Lectin
77	TRINITY_DN406_c1_g1_i8	55746		43058		Lectin
78	TRINITY_DN743_c0_g2_i6	57984		37922		Lectin

79	TRINITY_DN743_c0_g2_i8	57984		37922		Lectin
80	TRINITY_DN743_c0_g2_i4	57984		37922		Lectin
81	TRINITY_DN743_c0_g2_i7	57984		37922		Lectin
82	TRINITY_DN743_c0_g2_i5	57984		37922		Lectin
83	TRINITY_DN64_c0_g2_i1	34600		26239		Lectin
84	TRINITY_DN64_c0_g2_i7	34600		26239		Lectin
85	TRINITY_DN64_c0_g2_i5	34600		26239		Lectin
86	TRINITY_DN2895_c0_g2_i5	19892		14016		Lectin
87	TRINITY_DN12452_c0_g1_i2	16379		14453		Lectin
88	TRINITY_DN12452_c0_g1_i1	16379		14453		Lectin
89	TRINITY_DN712_c0_g1_i1	14673		10557		Lectin
90	TRINITY_DN712_c0_g1_i2	14673		10557		Lectin
91	TRINITY_DN1142_c0_g1_i3	14999		9308		Lectin
92	TRINITY_DN4209_c1_g1_i1	10672		8104		Lectin
93	TRINITY_DN5166_c0_g2_i3	7721		5562		Lectin
94	TRINITY_DN5166_c0_g2_i8	7721		5562		Lectin
95	TRINITY_DN5166_c0_g2_i7	7721		5562		Lectin
96	TRINITY_DN5166_c0_g2_i6	7721		5562		Lectin
97	TRINITY_DN5166_c0_g2_i4	7721		5562		Lectin
98	TRINITY_DN665_c0_g1_i2	6115		6340		Lectin
99	TRINITY_DN5315_c0_g1_i1	2539		2574		Lectin
100	TRINITY_DN292_c0_g1_i11	2066		1797		Lectin
101	TRINITY_DN292_c0_g1_i12	2066		1797		Lectin
102	TRINITY_DN292_c0_g1_i2	2066		1797		Lectin
103	TRINITY_DN404_c2_g1_i7	2168		1510		Lectin
104	TRINITY_DN404_c2_g1_i3	2168		1510		Lectin
105	TRINITY_DN404_c2_g1_i14	2168		1510		Lectin

106	TRINITY_DN2775_c0_g1_i3	1831		1433		Lectin
107	TRINITY_DN2775_c0_g1_i4	1831		1433		Lectin
108	TRINITY_DN8529_c0_g1_i2	1666		870		Lectin
109	TRINITY_DN9961_c0_g1_i3	819		690		Lectin
110	TRINITY_DN4429_c2_g1_i1	658		404		Lectin
111	TRINITY_DN4300_c0_g1_i2	416		344		Lectin
112	TRINITY_DN33170_c0_g1_i1	301		362		Lectin
113	TRINITY_DN823_c1_g1_i3	302		231		Lectin
114	TRINITY_DN1142_c0_g2_i1	296		181		Lectin
115	TRINITY_DN4039_c0_g1_i1	210		166		Lectin
116	TRINITY_DN3466_c0_g1_i7	162		142		Lectin
117	TRINITY_DN3466_c0_g1_i4	162		142		Lectin
118	TRINITY_DN3466_c0_g1_i9	162		142		Lectin
119	TRINITY_DN27565_c0_g1_i1	159		78		Lectin
120	TRINITY_DN27565_c0_g1_i2	159		78		Lectin
121	TRINITY_DN64_c2_g1_i9	155868		124797		Lectin
122	TRINITY_DN235_c0_g2_i2	737	0.0036%	800	0.0045%	Natriuretic peptide
123	TRINITY_DN22914_c0_g1_i6	593		602		Natriuretic peptide
124	TRINITY_DN22914_c0_g1_i8	593		602		Natriuretic peptide
125	TRINITY_DN300_c0_g2_i2	21283	0.0792%	19327	0.0873%	Neprilysin
126	TRINITY_DN300_c0_g2_i1	21283		19327		Neprilysin
127	TRINITY_DN1356_c0_g1_i2	34629	0.4517%	27064	0.4286%	NGF
128	TRINITY_DN1356_c0_g1_i4	34629		27064		NGF
129	TRINITY_DN1356_c0_g1_i17	34629		27064		NGF
130	TRINITY_DN1356_c0_g1_i10	34629		27064		NGF
131	TRINITY_DN1356_c0_g1_i20	34629		27064		NGF
132	TRINITY_DN1356_c0_g1_i13	34629		27064		NGF

133	TRINITY_DN1356_c0_g1_i14	34629		27064		NGF
134	TRINITY_DN2461_c0_g1_i1	303		289		NGF
135	TRINITY_DN10993_c0_g2_i1	124		107		NGF
136	TRINITY_DN1507_c0_g1_i3	10438	0.3500%	8762	0.3567%	PDE
137	TRINITY_DN1507_c0_g1_i5	10438		8762		PDE
138	TRINITY_DN1507_c0_g1_i21	10438		8762		PDE
139	TRINITY_DN1507_c0_g1_i24	10438		8762		PDE
140	TRINITY_DN1507_c0_g1_i17	10438		8762		PDE
141	TRINITY_DN1507_c0_g1_i22	10438		8762		PDE
142	TRINITY_DN1507_c0_g1_i10	10438		8762		PDE
143	TRINITY_DN1507_c0_g1_i9	10438		8762		PDE
144	TRINITY_DN1507_c0_g1_i20	10438		8762		PDE
145	TRINITY_DN1507_c0_g1_i25	10438		8762		PDE
146	TRINITY_DN1507_c0_g1_i15	10438		8762		PDE
147	TRINITY_DN1507_c0_g1_i13	10438		8762		PDE
148	TRINITY_DN1507_c0_g1_i19	10438		8762		PDE
149	TRINITY_DN1507_c0_g1_i18	10438		8762		PDE
150	TRINITY_DN1507_c0_g1_i1	10438		8762		PDE
151	TRINITY_DN1507_c0_g1_i7	10438		8762		PDE
152	TRINITY_DN1507_c0_g1_i6	10438		8762		PDE
153	TRINITY_DN1507_c0_g1_i26	10438		8762		PDE
154	TRINITY_DN24352_c0_g1_i1	163		182		PDE
155	TRINITY_DN7710_c0_g1_i2	106		94		PDE
156	TRINITY_DN0_c3_g2_i4	1250341	11.6991%	917455	10.4250%	PLA2
157	TRINITY_DN0_c3_g2_i1	1250341		917455		PLA2
158	TRINITY_DN0_c3_g2_i3	1250341		917455		PLA2
159	TRINITY_DN0_c3_g2_i5	1250341		917455		PLA2

160	TRINITY_DN0_c3_g2_i6	1250341		917455		PLA2
161	TRINITY_DN191_c0_g2_i2	7999		6715		PLA2
162	TRINITY_DN191_c0_g2_i1	7999		6715		PLA2
163	TRINITY_DN191_c0_g2_i3	7999		6715		PLA2
164	TRINITY_DN595_c0_g1_i2	5954		4747		PLA2
165	TRINITY_DN423_c1_g3_i1	562		462		PLA2
166	TRINITY_DN2075_c0_g1_i3	639		349		PLA2
167	TRINITY_DN2075_c0_g1_i2	639		349		PLA2
168	TRINITY_DN2075_c0_g1_i5	639		349		PLA2
169	TRINITY_DN2075_c0_g1_i1	639		349		PLA2
170	TRINITY_DN423_c1_g1_i3	471		302		PLA2
171	TRINITY_DN423_c1_g1_i4	471		302		PLA2
172	TRINITY_DN423_c1_g1_i1	471		302		PLA2
173	TRINITY_DN979_c0_g1_i6	324		330		PLA2
174	TRINITY_DN979_c0_g1_i10	324		330		PLA2
175	TRINITY_DN2209_c0_g1_i11	290		308		PLA2
176	TRINITY_DN2209_c0_g1_i1	290		308		PLA2
177	TRINITY_DN2209_c0_g1_i7	290		308		PLA2
178	TRINITY_DN2209_c0_g1_i8	290		308		PLA2
179	TRINITY_DN2209_c0_g1_i10	290		308		PLA2
180	TRINITY_DN4463_c0_g1_i1	203		158		PLA2
181	TRINITY_DN4463_c0_g1_i4	203		158		PLA2
182	TRINITY_DN4463_c0_g1_i3	203		158		PLA2
183	TRINITY_DN4463_c0_g1_i2	203		158		PLA2
184	TRINITY_DN0_c36_g2_i2	49906		40375		PLB
185	TRINITY_DN0_c36_g2_i1	49906	0.1859%	40375	0.1827%	PLB
186	TRINITY_DN12219_c0_g2_i1	137		157		PLB

187	TRINITY_DN30_c0_g1_i3	681168	23.5318%	577129	24.3709%	SVMP
188	TRINITY_DN30_c0_g1_i9	681168		577129		SVMP
189	TRINITY_DN30_c0_g1_i8	681168		577129		SVMP
190	TRINITY_DN30_c0_g1_i5	681168		577129		SVMP
191	TRINITY_DN30_c0_g1_i11	681168		577129		SVMP
192	TRINITY_DN30_c0_g1_i6	681168		577129		SVMP
193	TRINITY_DN30_c0_g1_i15	681168		577129		SVMP
194	TRINITY_DN30_c0_g1_i14	681168		577129		SVMP
195	TRINITY_DN30_c0_g1_i12	681168		577129		SVMP
196	TRINITY_DN30_c0_g1_i4	681168		577129		SVMP
197	TRINITY_DN30_c0_g1_i10	681168		577129		SVMP
198	TRINITY_DN30_c0_g1_i1	681168		577129		SVMP
199	TRINITY_DN3546_c0_g1_i1	171971		156100		SVMP
200	TRINITY_DN459_c0_g1_i5	155256		139360		SVMP
201	TRINITY_DN459_c0_g1_i6	155256		139360		SVMP
202	TRINITY_DN459_c0_g1_i20	155256		139360		SVMP
203	TRINITY_DN459_c0_g1_i2	155256		139360		SVMP
204	TRINITY_DN459_c0_g1_i17	155256		139360		SVMP
205	TRINITY_DN459_c0_g1_i13	155256		139360		SVMP
206	TRINITY_DN459_c0_g1_i10	155256		139360		SVMP
207	TRINITY_DN459_c0_g1_i18	155256		139360		SVMP
208	TRINITY_DN626_c0_g1_i7	120375		116184		SVMP
209	TRINITY_DN2177_c0_g1_i6	96954		79769		SVMP
210	TRINITY_DN2177_c0_g1_i10	96954		79769		SVMP
211	TRINITY_DN2177_c0_g1_i4	96954		79769		SVMP
212	TRINITY_DN2177_c0_g1_i8	96954		79769		SVMP
213	TRINITY_DN2177_c0_g1_i7	96954		79769		SVMP

214	TRINITY_DN2177_c0_g1_i2	96954		79769		SVMP
215	TRINITY_DN2177_c0_g1_i9	96954		79769		SVMP
216	TRINITY_DN2177_c0_g1_i3	96954		79769		SVMP
217	TRINITY_DN2177_c0_g1_i5	96954		79769		SVMP
218	TRINITY_DN2452_c0_g1_i4	71706		63531		SVMP
219	TRINITY_DN2452_c0_g1_i8	71706		63531		SVMP
220	TRINITY_DN2452_c0_g1_i7	71706		63531		SVMP
221	TRINITY_DN2452_c0_g1_i5	71706		63531		SVMP
222	TRINITY_DN2452_c0_g1_i2	71706		63531		SVMP
223	TRINITY_DN2452_c0_g1_i1	71706		63531		SVMP
224	TRINITY_DN2452_c0_g1_i3	71706		63531		SVMP
225	TRINITY_DN459_c2_g1_i1	71927		62740		SVMP
226	TRINITY_DN459_c2_g1_i2	71927		62740		SVMP
227	TRINITY_DN1004_c0_g1_i4	65759		52769		SVMP
228	TRINITY_DN1004_c0_g1_i3	65759		52769		SVMP
229	TRINITY_DN1004_c0_g1_i1	65759		52769		SVMP
230	TRINITY_DN1004_c0_g1_i5	65759		52769		SVMP
231	TRINITY_DN1004_c0_g1_i2	65759		52769		SVMP
232	TRINITY_DN1385_c0_g1_i4	58798		49893		SVMP
233	TRINITY_DN1385_c0_g1_i3	58798		49893		SVMP
234	TRINITY_DN1385_c0_g1_i5	58798		49893		SVMP
235	TRINITY_DN1385_c0_g1_i6	58798		49893		SVMP
236	TRINITY_DN1385_c0_g1_i2	58798		49893		SVMP
237	TRINITY_DN1385_c0_g1_i7	58798		49893		SVMP
238	TRINITY_DN30_c43_g1_i1	49185		42879		SVMP
239	TRINITY_DN714_c0_g2_i1	44070		35862		SVMP
240	TRINITY_DN714_c0_g2_i5	44070		35862		SVMP

241	TRINITY_DN714_c0_g2_i3	44070		35862		SVMP
242	TRINITY_DN714_c0_g2_i6	44070		35862		SVMP
243	TRINITY_DN714_c0_g2_i4	44070		35862		SVMP
244	TRINITY_DN714_c0_g2_i2	44070		35862		SVMP
245	TRINITY_DN714_c0_g2_i7	44070		35862		SVMP
246	TRINITY_DN3470_c0_g1_i1	31787		25009		SVMP
247	TRINITY_DN3470_c0_g1_i2	31787		25009		SVMP
248	TRINITY_DN714_c0_g3_i2	28623		24144		SVMP
249	TRINITY_DN714_c0_g3_i1	28623		24144		SVMP
250	TRINITY_DN217_c0_g1_i1	24424		21675		SVMP
251	TRINITY_DN5928_c0_g1_i1	23706		21041		SVMP
252	TRINITY_DN5928_c0_g1_i2	23706		21041		SVMP
253	TRINITY_DN5928_c0_g1_i3	23706		21041		SVMP
254	TRINITY_DN5928_c0_g1_i4	23706		21041		SVMP
255	TRINITY_DN5928_c0_g1_i5	23706		21041		SVMP
256	TRINITY_DN182_c0_g1_i1	17762		16883		SVMP
257	TRINITY_DN3041_c0_g1_i1	12812		10806		SVMP
258	TRINITY_DN30_c34_g1_i1	11503		9653		SVMP
259	TRINITY_DN5926_c2_g1_i5	9372		9611		SVMP
260	TRINITY_DN2736_c0_g1_i1	5906		4567		SVMP
261	TRINITY_DN471_c0_g3_i1	4099		4329		SVMP
262	TRINITY_DN2708_c0_g1_i1	4472		3907		SVMP
263	TRINITY_DN925_c0_g1_i10	3787		3434		SVMP
264	TRINITY_DN5016_c0_g1_i5	3606		3308		SVMP
265	TRINITY_DN3294_c0_g1_i2	2695		2691		SVMP
266	TRINITY_DN3294_c0_g1_i1	2695		2691		SVMP
267	TRINITY_DN3294_c0_g1_i4	2695		2691		SVMP

268	TRINITY_DN479_c1_g1_i7	2393		2266		SVMP
269	TRINITY_DN479_c1_g1_i2	2393		2266		SVMP
270	TRINITY_DN479_c1_g1_i15	2393		2266		SVMP
271	TRINITY_DN479_c1_g1_i13	2393		2266		SVMP
272	TRINITY_DN479_c1_g1_i12	2393		2266		SVMP
273	TRINITY_DN479_c1_g1_i11	2393		2266		SVMP
274	TRINITY_DN479_c1_g1_i16	2393		2266		SVMP
275	TRINITY_DN145_c0_g1_i7	1239		1339		SVMP
276	TRINITY_DN471_c0_g1_i1	1338		1053		SVMP
277	TRINITY_DN1550_c0_g1_i1	1272		861		SVMP
278	TRINITY_DN1550_c0_g1_i2	1272		861		SVMP
279	TRINITY_DN2736_c0_g2_i1	1104		987		SVMP
280	TRINITY_DN344_c0_g1_i1	923		974		SVMP
281	TRINITY_DN344_c0_g1_i5	923		974		SVMP
282	TRINITY_DN5025_c0_g1_i11	873		731		SVMP
283	TRINITY_DN5025_c0_g1_i3	873		731		SVMP
284	TRINITY_DN15772_c0_g3_i1	738		701		SVMP
285	TRINITY_DN15772_c0_g3_i2	738		701		SVMP
286	TRINITY_DN2280_c0_g1_i6	553		743		SVMP
287	TRINITY_DN225_c1_g1_i11	648		629		SVMP
288	TRINITY_DN225_c1_g1_i2	648		629		SVMP
289	TRINITY_DN10090_c0_g1_i2	476		490		SVMP
290	TRINITY_DN10090_c0_g1_i4	476		490		SVMP
291	TRINITY_DN2230_c0_g1_i2	408		464		SVMP
292	TRINITY_DN2230_c0_g1_i9	408		464		SVMP
293	TRINITY_DN2230_c0_g1_i7	408		464		SVMP
294	TRINITY_DN2230_c0_g1_i4	408		464		SVMP

295	TRINITY_DN2230_c0_g1_i5	408		464		SVMP
296	TRINITY_DN2230_c0_g1_i1	408		464		SVMP
297	TRINITY_DN2230_c0_g1_i8	408		464		SVMP
298	TRINITY_DN2230_c0_g1_i6	408		464		SVMP
299	TRINITY_DN3781_c0_g1_i4	310		316		SVMP
300	TRINITY_DN901_c1_g1_i1	274		346		SVMP
301	TRINITY_DN5467_c0_g1_i6	249		261		SVMP
302	TRINITY_DN5467_c0_g1_i5	249		261		SVMP
303	TRINITY_DN5467_c0_g1_i4	249		261		SVMP
304	TRINITY_DN5467_c0_g1_i7	249		261		SVMP
305	TRINITY_DN5467_c0_g1_i1	249		261		SVMP
306	TRINITY_DN471_c0_g2_i1	287		209		SVMP
307	TRINITY_DN2177_c1_g1_i2	179		242		SVMP
308	TRINITY_DN2177_c1_g1_i1	179		242		SVMP
309	TRINITY_DN1910_c1_g1_i3	201		185		SVMP
310	TRINITY_DN1910_c1_g1_i1	201		185		SVMP
311	TRINITY_DN1571_c1_g1_i2	158		159		SVMP
312	TRINITY_DN1571_c1_g1_i3	158		159		SVMP
313	TRINITY_DN12142_c0_g1_i7	134		149		SVMP
314	TRINITY_DN12142_c0_g1_i4	134		149		SVMP
315	TRINITY_DN12142_c0_g1_i2	134		149		SVMP
316	TRINITY_DN12142_c0_g1_i6	134		149		SVMP
317	TRINITY_DN12142_c0_g1_i1	134		149		SVMP
318	TRINITY_DN12142_c0_g1_i5	134		149		SVMP
319	TRINITY_DN12142_c0_g1_i3	134		149		SVMP
320	TRINITY_DN3629_c0_g1_i9	117		157		SVMP
321	TRINITY_DN5311_c0_g1_i5	136		134		SVMP

322	TRINITY_DN754_c2_g1_i1	117		110		SVMP
323	TRINITY_DN4004_c0_g1_i4	123		94		SVMP
324	TRINITY_DN3245_c0_g1_i7	85		125		SVMP
325	TRINITY_DN0_c0_g1_i3	1028266	19.0835%	985655	21.4228%	SVSP
326	TRINITY_DN0_c0_g1_i13	1028266		985655		SVSP
327	TRINITY_DN0_c0_g1_i8	1028266		985655		SVSP
328	TRINITY_DN0_c0_g1_i2	1028266		985655		SVSP
329	TRINITY_DN240_c0_g1_i5	260469		228576		SVSP
330	TRINITY_DN240_c0_g1_i6	260469		228576		SVSP
331	TRINITY_DN240_c0_g1_i8	260469		228576		SVSP
332	TRINITY_DN240_c0_g1_i7	260469		228576		SVSP
333	TRINITY_DN240_c0_g1_i3	260469		228576		SVSP
334	TRINITY_DN441_c0_g1_i15	244521		215565		SVSP
335	TRINITY_DN441_c0_g1_i20	244521		215565		SVSP
336	TRINITY_DN441_c0_g1_i1	244521		215565		SVSP
337	TRINITY_DN441_c0_g1_i8	244521		215565		SVSP
338	TRINITY_DN441_c0_g1_i9	244521		215565		SVSP
339	TRINITY_DN441_c0_g1_i13	244521		215565		SVSP
340	TRINITY_DN441_c0_g1_i11	244521		215565		SVSP
341	TRINITY_DN441_c0_g1_i5	244521		215565		SVSP
342	TRINITY_DN441_c0_g1_i16	244521		215565		SVSP
343	TRINITY_DN441_c0_g1_i19	244521		215565		SVSP
344	TRINITY_DN441_c0_g1_i6	244521		215565		SVSP
345	TRINITY_DN441_c0_g1_i3	244521		215565		SVSP
346	TRINITY_DN626_c0_g1_i4	120375		116184		SVSP
347	TRINITY_DN626_c0_g1_i5	120375		116184		SVSP
348	TRINITY_DN626_c0_g1_i3	120375		116184		SVSP

349	TRINITY_DN626_c0_g1_i13	120375		116184		SVSP
350	TRINITY_DN626_c0_g1_i9	120375		116184		SVSP
351	TRINITY_DN343_c0_g1_i3	104694		95680		SVSP
352	TRINITY_DN343_c0_g1_i8	104694		95680		SVSP
353	TRINITY_DN343_c0_g1_i6	104694		95680		SVSP
354	TRINITY_DN343_c0_g1_i1	104694		95680		SVSP
355	TRINITY_DN1328_c0_g1_i8	67012		62802		SVSP
356	TRINITY_DN1328_c0_g1_i4	67012		62802		SVSP
357	TRINITY_DN1328_c0_g1_i1	67012		62802		SVSP
358	TRINITY_DN1328_c0_g1_i7	67012		62802		SVSP
359	TRINITY_DN1328_c0_g1_i5	67012		62802		SVSP
360	TRINITY_DN1328_c0_g1_i6	67012		62802		SVSP
361	TRINITY_DN424_c0_g2_i2	38961		40631		SVSP
362	TRINITY_DN424_c0_g2_i3	38961		40631		SVSP
363	TRINITY_DN424_c0_g2_i4	38961		40631		SVSP
364	TRINITY_DN424_c0_g2_i1	38961		40631		SVSP
365	TRINITY_DN0_c16_g1_i2	37666		39022		SVSP
366	TRINITY_DN1959_c0_g1_i3	36961		35266		SVSP
367	TRINITY_DN1959_c0_g1_i4	36961		35266		SVSP
368	TRINITY_DN424_c0_g1_i5	28805		27219		SVSP
369	TRINITY_DN424_c0_g1_i6	28805		27219		SVSP
370	TRINITY_DN424_c0_g1_i2	28805		27219		SVSP
371	TRINITY_DN424_c0_g1_i4	28805		27219		SVSP
372	TRINITY_DN240_c1_g1_i4	14684		13638		SVSP
373	TRINITY_DN240_c1_g1_i15	14684		13638		SVSP
374	TRINITY_DN240_c1_g1_i3	14684		13638		SVSP
375	TRINITY_DN240_c1_g1_i14	14684		13638		SVSP

376	TRINITY_DN240_c1_g1_i1	14684		13638		SVSP
377	TRINITY_DN240_c1_g1_i12	14684		13638		SVSP
378	TRINITY_DN13494_c0_g1_i1	2364		2056		SVSP
379	TRINITY_DN5609_c0_g1_i9	1670		1436		SVSP
380	TRINITY_DN5609_c0_g1_i3	1670		1436		SVSP
381	TRINITY_DN5609_c0_g1_i2	1670		1436		SVSP
382	TRINITY_DN5609_c0_g1_i7	1670		1436		SVSP
383	TRINITY_DN5609_c0_g1_i4	1670		1436		SVSP
384	TRINITY_DN5609_c0_g1_i6	1670		1436		SVSP
385	TRINITY_DN1660_c0_g1_i3	439		429		SVSP
386	TRINITY_DN1660_c0_g1_i2	439		429		SVSP
387	TRINITY_DN1959_c0_g2_i1	372		424		SVSP
388	TRINITY_DN21404_c0_g1_i3	336		300		SVSP
389	TRINITY_DN21404_c0_g1_i1	336		300		SVSP
390	TRINITY_DN21404_c0_g1_i2	336		300		SVSP
391	TRINITY_DN21404_c0_g1_i5	336		300		SVSP
392	TRINITY_DN11879_c0_g1_i1	305		300		SVSP
393	TRINITY_DN10537_c1_g2_i1	145		148		SVSP
394	TRINITY_DN4899_c0_g1_i1	113		96		SVSP
395	TRINITY_DN4899_c0_g1_i3	113		96		SVSP
396	TRINITY_DN588_c0_g1_i5	633157	4.1692%	559413	4.5800%	VEGF
397	TRINITY_DN588_c0_g1_i6	633157		559413		VEGF
398	TRINITY_DN588_c0_g1_i3	633157		559413		VEGF
399	TRINITY_DN19_c0_g1_i12	48630		49815		VEGF
400	TRINITY_DN19_c0_g1_i11	48630		49815		VEGF
401	TRINITY_DN19_c0_g1_i1	48630		49815		VEGF
402	TRINITY_DN19_c0_g1_i3	48630		49815		VEGF

403	TRINITY_DN19_c0_g1_i8	48630		49815		VEGF
404	TRINITY_DN19_c0_g1_i2	48630		49815		VEGF
405	TRINITY_DN19_c0_g1_i9	48630		49815		VEGF
406	TRINITY_DN19_c0_g2_i1	1215		1598		VEGF
407	TRINITY_DN10045_c2_g1_i1	173		168		VEGF
408	TRINITY_DN101577_c0_g1_i1	249	0.0005%	112	0.0003%	Venom factor
409	TRINITY_DN3484_c0_g1_i1	89	0.0002%	123	0.0003%	Waprin
**Total Abundance**			**100.00%**		**100.00%**	
410	TRINITY_DN960_c0_g1_i1	7208426	12.5006%	6245792	12.5007%	SVMPI
411	TRINITY_DN3982_c0_g2_i1	258		258		SVMPI
412	TRINITY_DN89609_c0_g1_i1	134		141		SVMPI
413	TRINITY_DN960_c0_g1_i2	7208426	62.4996%	6245792	62.4995%	SVMPI, BPP
414	TRINITY_DN960_c0_g1_i9	7208426		6245792		SVMPI, BPP
415	TRINITY_DN960_c0_g1_i8	7208426		6245792		SVMPI, BPP
416	TRINITY_DN960_c0_g1_i5	7208426		6245792		SVMPI, BPP
417	TRINITY_DN960_c0_g1_i6	7208426		6245792		SVMPI, BPP
418	TRINITY_DN960_c0_g1_i3	7208426	24.9998%	6245792	24.9998%	SVMPI, BPP, Natriuretic peptide
419	TRINITY_DN960_c0_g1_i10	7208426		6245792		SVMPI, BPP, Natriuretic peptide
**Total Abundance**			**100.00%**		**100.00%**	

Each toxin family and the corresponding relative abundance is uniquely color coded.

Supplementary Table S4. Toxicity profiles of *Daboia* venoms.

**Sample ID**	**Venom Dose** (µg)					**Number of survivors**					**LD_{50}** (µg/mouse)	**LD_{50}** (mg/Kg)
D. palaestinae	3.84	4.8	6	7.5	9.37	5	5	4	1	0	6.71<br>6.19- 7.26	0.34<br>0.31-0.36
D. russelii	2.56	3.2	4	5	6.25	4	4	3	1	0	3.98<br>3.41- 4.65	0.20<br>0.171- 0.24

This table shows the median lethal dose and survivorship data for *Daboia* venoms.
